# Facile Synthesis of Nonalternant π‑Conjugated Azaborines via Boron-Deleting Annulation

**DOI:** 10.1021/jacs.5c23345

**Published:** 2026-03-17

**Authors:** Weiwen Zhuang, Farshad Shiri, Faan-Fung Hung, Zhen Wang, Dongdong Zhang, Lian Duan, Chi-Ming Che, Zhenyang Lin, Junzhi Liu

**Affiliations:** † Department of Chemistry, 25809 The University of Hong Kong, Pokfulam Road, Hong Kong 999077, China; ‡ Materials Innovation Institute for Life Sciences and Energy (MILES), HKU-SIRI, Shenzhen 518045, China; § Department of Chemistry, The Hong Kong University of Science and Technology, Hong Kong 999077, China; ∥ Key Lab of Organic Optoelectronics and Molecular Engineering of Ministry of Education, Department of Chemistry, 12442 Tsinghua University, Beijing 100084, China; ⊥ State Key Laboratory of Synthetic Chemistry, HKU-CAS Joint Laboratory on New Materials and Shanghai-Hong Kong Joint Laboratory on Chemical Synthesis, The University of Hong Kong, Pokfulam Road, Hong Kong 999077, China

## Abstract

Skeletal editing of aza-heteroarenes has become a powerful strategy for late-stage molecular diversification in medicinal and material chemistry. π-Conjugated azaborines have attracted increasing attention due to their exceptional optoelectronic properties and practical applications in organic light-emitting diodes (OLEDs). However, ring manipulation of boron-containing rings remains a long-standing challenge, and cascade skeletal editing beyond monocyclic frameworks is largely underdeveloped. Here, we report a boron-deleting annulation (BDA) reaction that enables the transformation of unstrained bicyclic 1,4-azaborines into a diaza-pentagon-heptagon pair in a one-pot reaction, affording a wide array of nonalternant π-conjugated azaborines (>35 examples) with unique optoelectronic properties. This late-stage modification approach thus balances synthetic simplicity with structural diversity and expands the accessible chemical space of functional π-systems for optoelectronic applications. Additionally, the BDA product can trigger consecutive cyclodehydrogenation to zipping-up sophisticated nanographenes via further periphery-to-core extension. Mechanistic studies reveal a series of unprecedented ring contraction–expansion processes. Remarkably, yellow OLEDs based on BDA product achieved a record 23.0% external quantum efficiency without delayed fluorescence, demonstrating the potential for high-performance electroluminescence.

## Graphical abstract

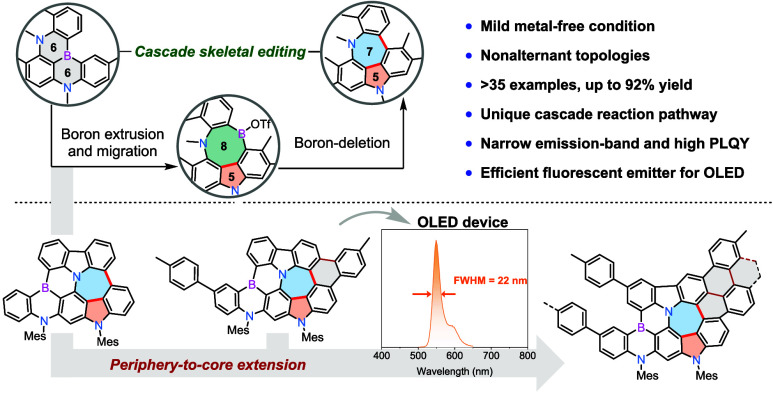

## Introduction

The precise bottom-up synthesis of polycyclic-conjugated hydrocarbon (PCH)-based functional molecules typically requires *de novo* design, while their finely tuned photophysical properties depend on heteroatom doping and π-electron density.^1,2^ Skeletal editing of pharmaceuticals or bioactive small molecules,^3–7^ such as cyclic amine,^8,9^ pyrrole,^10,11^ indole,^12–14^ quinoline,^15,16^ pyridine,^17,18^ pyrimidine,^19,20^ furan,^21^ thiazole,^22^ and thiophene,^23^ has experienced a renaissance over the past decade as a thought-after strategy for scaffold hopping. These transformations typically involve single-atom insertion/deletion (C, N, O)^10–16,18–21^ or atom-pair swap.^17^ By contrast, strategies modifying multiple rings simultaneously face greater challenges. In 2023, Glorius and co-workers reported photocatalytic insertion of bicyclobutane into thiophene, which allowed the facile construction of unique bicyclic frameworks (Figure 1a).^23^ Cascade ring remodeling of fused aromatic systems dates back to 1947 when thermal azulene-to-naphthalene (AN) rearrangement was reported.^24^ Although the AN rearrangement offers limited synthetic utility, simultaneous reconstruction of multiple rings in polycyclic systems, beyond the well-established periphery-to-core extension strategy by the Scholl reaction,^25^ could streamline the synthesis of PCHs with refined topologies and systematical modulation of photophysical properties.

**Figure 1:**
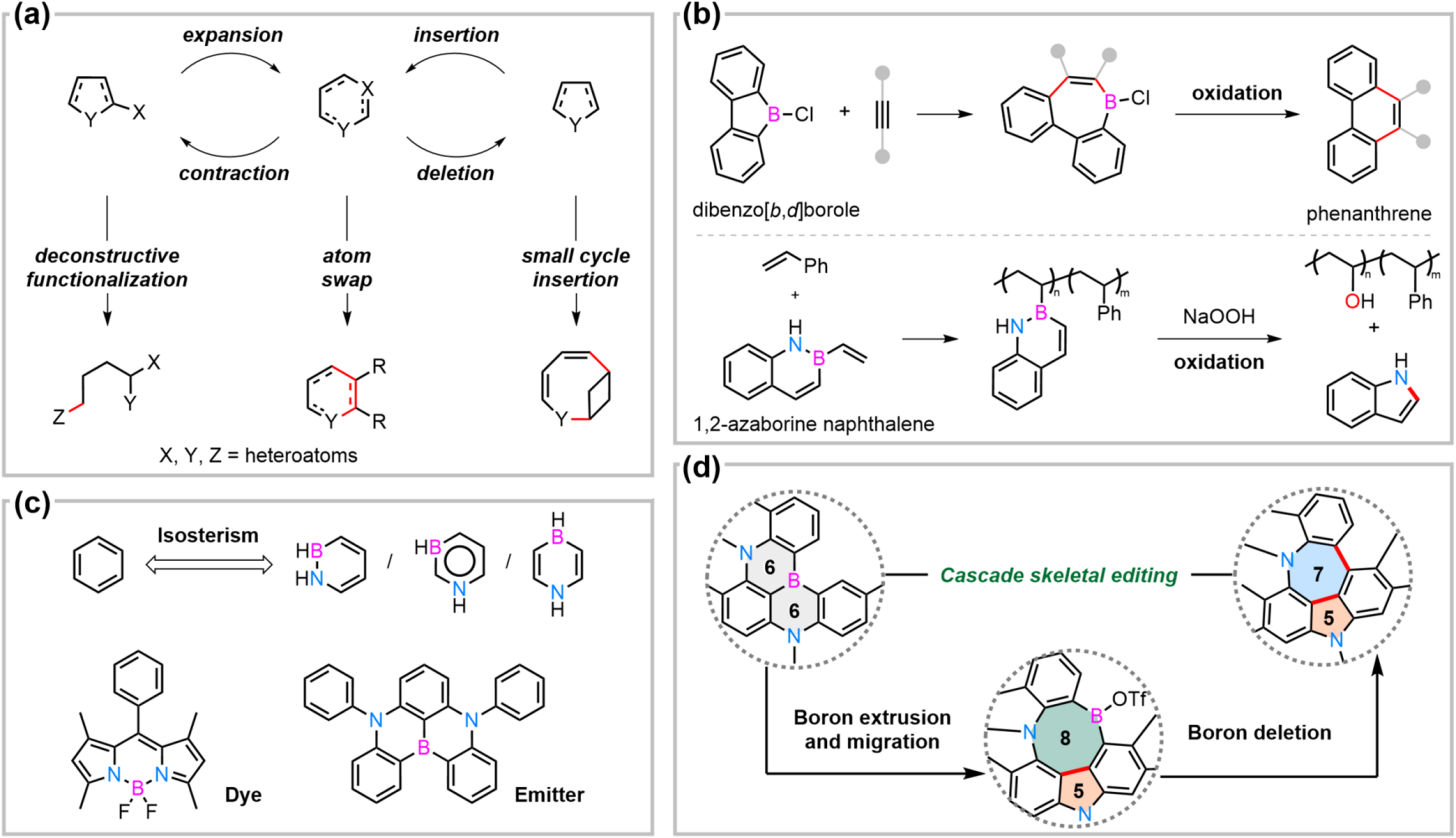
Classical skeletal editing strategies (a). Literature precedents for ring manipulation of boron-containing aromatic rings (b). Azaborines as isoteres of benzene and representative functional molecules with azaborine rings (c). Cascade skeletal editing of azaborines via boron-deleting annulation is shown in this work (d).

Direct ring manipulation of boracycles, especially for unsaturated ones, remains notably rare (Figure 1b). One of the few examples leverages the intrinsic high reactivity of dibenzo­[*b,d*]­boroles, which undergo [5 + 2] cycloaddition with alkynes to generate isolable seven-membered boracyclic intermediates. Subsequent chemical oxidation yields phenanthrenes.^26,27^ In 2018, Klausen and co-workers reported a polymerization–oxidation strategy for the preparation of poly­(vinyl alcohol) (PVA)-incorporated copolymers, employing 2-vinyl-1,2-azaborine naphthalene as a vinyl alcohol surrogate. Oxidative cleavage of the B–N and B–C bonds furnished indole as a boron-deleted side product.^28^

The stability-reactivity trade-off in boron-containing π-systems can be partially regulated through nitrogen-embedded backbone and constrain engineering in the azaborine ring systems.^29,30^ As unique benzene isosteres, azaborines offer compelling potential for both pharmaceutical development and materials science (Figure 1c).^31^ For instance, the prevailing boron dipyrromethenes (BODIPYs) dyes^32^ and 1,4-azaborine based multiresonant (MR) emitters^33–37^ have widespread applications owing to their excellent spectroscopic and photophysical properties. The geometrical diversity and versatility of those boron–nitrogen doped materials could be increased drastically by late-stage diversification.^38^

Inspired by recent advances in metal-free oxidative coupling of tetracoordinate borate,^39–41^ we aimed to find a system that could thermodynamically and kinetically address azaborine rings for cascade skeletal editing (Figure 1d). We envisaged that coordinating the activation of slightly constrained tricoordinate boron in the bicyclic azaborines could generate more reactive tetracoordinate borate for a sequential boron-deleting annulation (BDA) reaction. Specifically, removal of one boron atom in bicyclic azaborine rings of a triazadibora polycyclic system, namely, carbazole-based DABNA analogs^42^ would facilitate the formation of two new C­(sp^2^)–C­(sp^2^) bonds, generating a diaza-heptagon–pentagon pair. The resultant π-conjugated nonalternant^43,44^ azaborines exhibit exceptional photophysical properties, positioning them as promising functional molecules. Moreover, highly efficient yellow OLEDs were fabricated using the BDA product (**10k**) as the emitter, which achieved record-breaking external quantum efficiencies (EQEs) of up to 23.0% without relying on delayed fluorescence, demonstrating its significant potential for high-performance electroluminescence.

## Results and Discussion

### Reaction Development

Motivated by the coordination of fluorine anion^45^ and benzoquinone (BQ)^46^ to the novel MR-core, we aimed to identify mild conditions to activate the boron atoms in MR-core for the C­(sp^2^)–C­(sp^2^) bond formation. To assess the viability of BDA for the cascade ring manipulation of bicyclic azaborines, we selected compound **1** as the model substrate, using 2,3-dichloro-5,6-dicyano-1,4-benzoquinone (DDQ) as an oxidant and a series of Brønsted acids as activators (Scheme 1a and Tables S1–S5). After optimization, product **2** was obtained in 82% yield based on nuclear magnetic resonance (NMR) analysis, following a 12-h reaction in anhydrous dichloromethane (DCM) using trifluoromethanesulfonic acid (HOTf, triflic acid) as an activator (Scheme 1a, entry 1). The molecular structure of compound **2**, featuring a pentagon–heptagon pair, was unambiguously determined by single-crystal X-ray diffraction (SC-XRD) analysis (CCDC: no. 2431227). Other reaction parameters were systematically evaluated. Both DDQ and HOTf proved indispensable for the successful boron-deleting annulation of compound **1** (Scheme 1a, entries 2–4). Employing weaker Brønsted acid, such as trifluoroacetic acid (TFA), did not lead to the desired product (Scheme 1a, entry 5). Alternative oxidants, including *p*-chloranil, demonstrated low efficiency (Scheme 1a, entry 6). Notably, reducing the reaction temperature to 0 °C resulted in a significant decrease in the yield (Scheme 1a, entry 7). Scale-up studies were conducted using 1.5 mmol of substrate **1**.

**Scheme 1:**
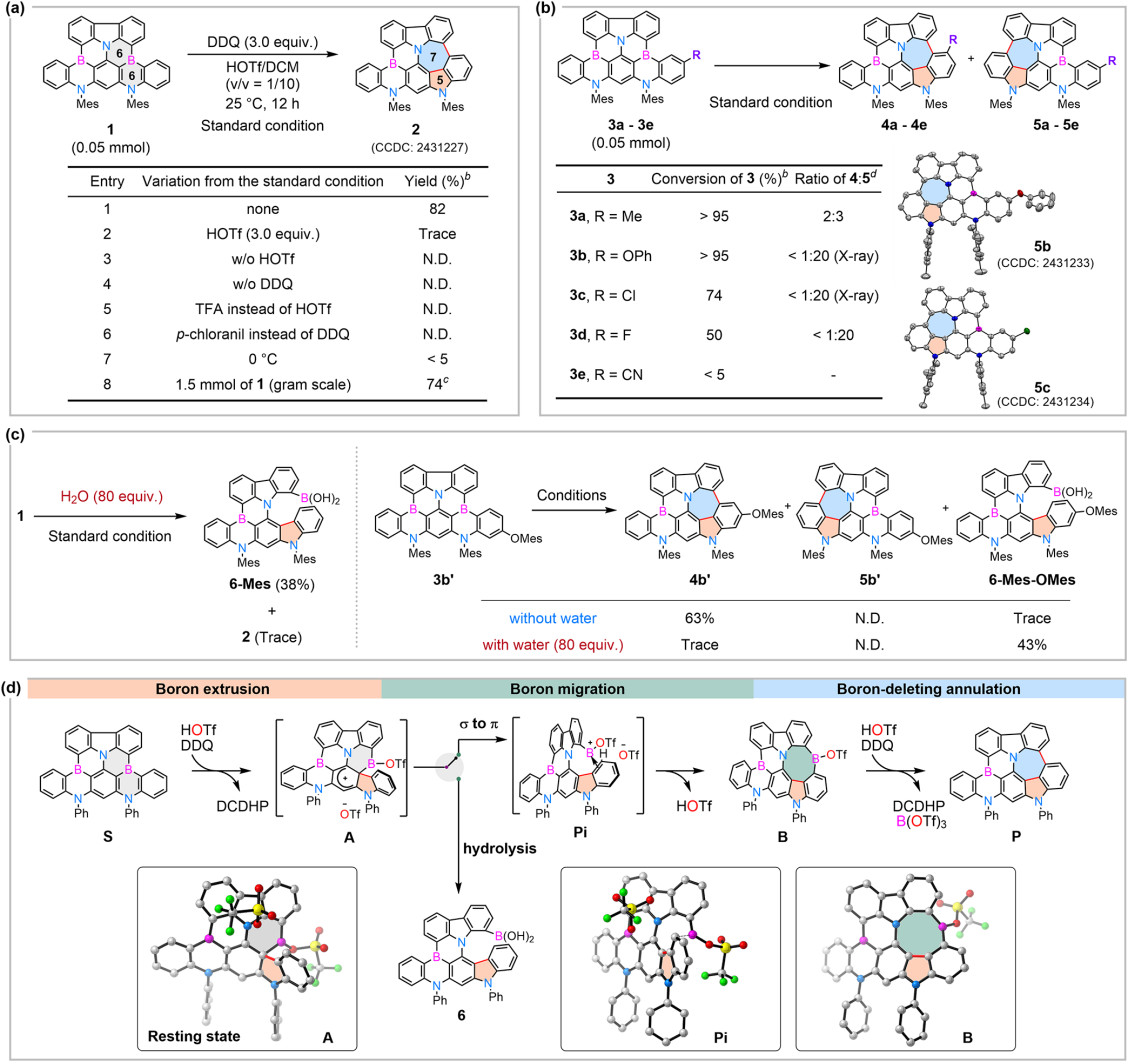
Reaction Development and Proposed Reaction Mechanism^d^

b Yield or conversion was determined by ^1^H NMR using 1,3,5-trimethoxybenzene as an internal standard.
c Gram-scale reaction with the isolated yield.
d Ratio based on isolated products. Mes, mesityl; OPh, phenol; OMes, mesityloxy; Ph, phenyl.
a Condition optimization (a). Evaluation of electronic effects on reactivity and regioselectivity (b). Water interrupted BDA reactions (c). Proposed mechanism with DFT optimized structures (d).

Optimization of the concentration and ratio of DCM/HOTf resulted in variable conversion rates for **1** (Figure S1). When 1.0 g of **1** (1.5 mmol) reacted with 3.0 equiv. DDQ in the presence of 1.0 mL HOTf and 50.0 mL DCM, the isolated yield of **2** was 74% (Scheme 1a, entry 8). To our surprise, subjecting the isolated product **2** to the standard condition did not lead to the deletion of the remaining boron atom, even at an elevated temperature (80 °C) in 1,2-dichloroethane (DCE) (Figure S2). The formation of the diaza-pentagon–heptagon pair resulted in a more rigid framework relative to the parent DABNA skeleton, which may suppress the coordination activation of the remaining tricoordinate boron atom.^29,30^ Consequently, the second BDA reaction is not feasible.

With the optimized BDA reaction conditions established, we next investigated the electronic effects on the reactivity and regioselectivity using unsymmetric substrates (**3a–3e)** (Scheme 1b). Substrates bearing electron-withdrawing groups (**3c**–**3e**) exhibited conversions lower than those with electron-donating groups (**3a**-**3b**). For **3a**, the methyl substituent yielded a 2:3 ratio of **4a**:**5a**. Other evaluated substituents (**3b**–**3d**) led to the formation of compounds **5b**-**5d**, respectively. The structures of **5b** and **5c** were confirmed by NMR spectroscopy and SC-XRD analysis (CCDC: 2431233 and 2431234 for **5b** and **5c**, respectively). The pronounced electron deficiency in **3e** led to an extremely low conversion. In addition, the presence of water in the reaction mixture resulted in the formation of interrupted BDA product **6-Mes** instead of expected **2** (Scheme 1c, left). Intriguingly, the positions of electron-rich aryloxy groups play a pivotal role in determining the regioselectivity of BDA reactions for asymmetrical substrates **3b** and **3b’**. Phenol group (OPh) attached to the *meta-*position of the boron atom should be considered an electron-withdrawing substituent exerting only inductive withdrawing effect, which led to the formation of **5b**. On the other hand, mesityloxy groups (OMes) located at the *para*-position to the boron atom showed an electron-donating effect and directed the boron deletion toward its side to generate **4b′**. Furthermore, water interrupted BDA reaction of **3b′** afforded **6-Mes-OMes** with excellent regioselectivity (Scheme 1c, right). Based on these experimental observations, we proposed a plausible mechanism for the BDA reaction of substrate **S** (Scheme 1d), which was supported by density functional theory (DFT) calculations (Figure 2a,2b).

**Figure 2:**
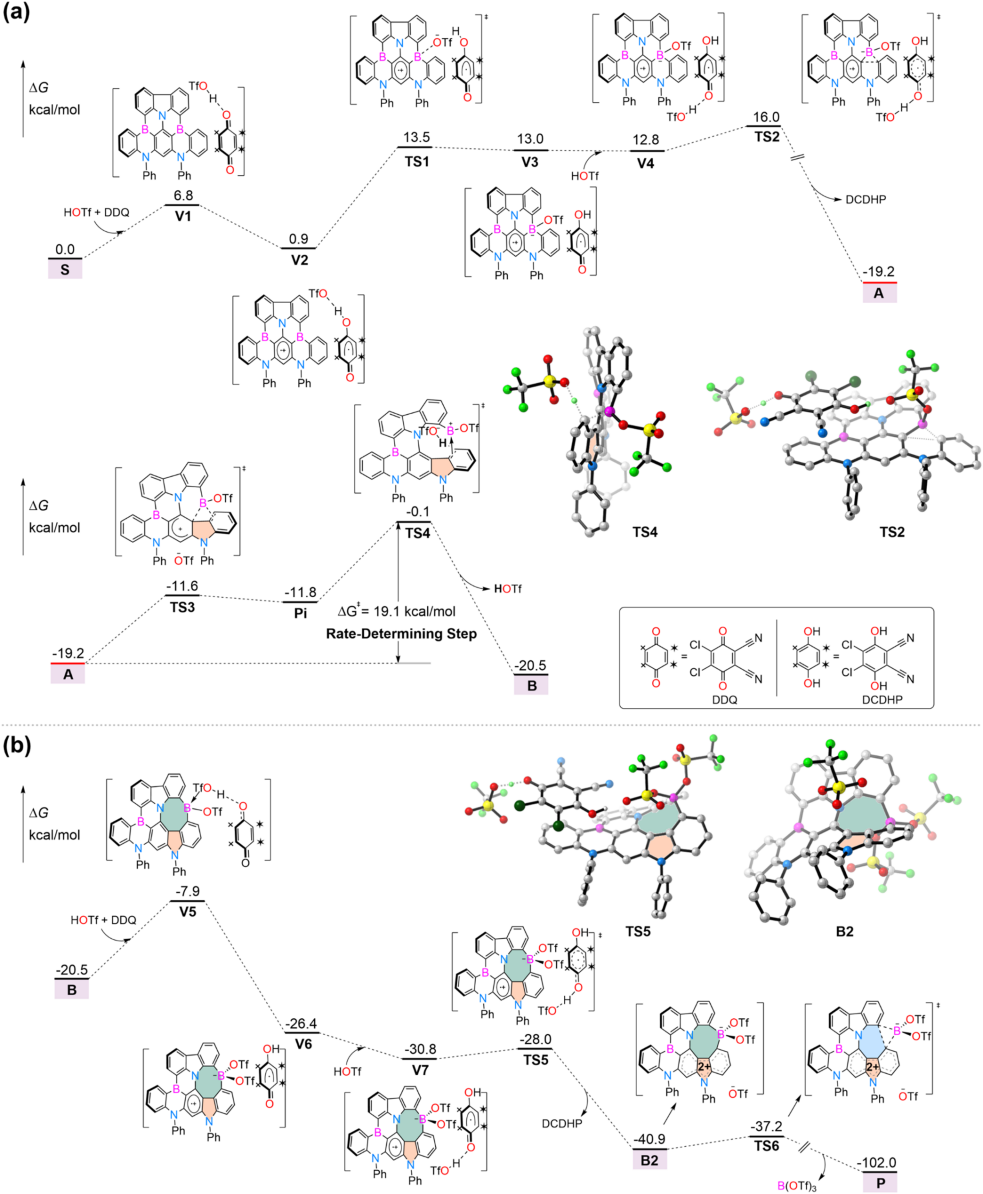
Gibbs free energy profiles calculated for **S** → **B** (a) and **B** → **P** (b). Gibbs free energy is given in kcal/mol. The optimized structures of **TS2**, **TS4**, **TS5**, and **B2** are shown. DFT calculations were conducted at the SMD/M06-2X/def2-TZVP//SMD/M06-2X/def2-SVP level of theory.

### Mechanistic Investigations

The DFT results indicate that the reaction begins with the formation of van der Waals complex **V1** among azaborine **S**, DDQ, and HOTf (Figure 2a). A single-electron transfer (SET) occurs from the π-system of **S** to DDQ, leading to the formation of radical ion pair **V2**. Then, the in situ generated triflate anion (TfO^–^) coordinates to the Lewis acidic boron center in the oxidized azaborine via the transition state **TS1**, forming the boron–triflate adduct **V3**. The remaining carbonyl group of the partially reduced DDQ is activated by another HOTf, facilitating a second SET through the transition state **TS2**. Meanwhile, the ring contraction is triggered to generate arenium intermediate **A** along with the release of 4,5-dichloro-3,6-dihydroxyphthalonitrile (DCDHP) (Figure 2a). The formation of **A** is highly exergonic (ΔG = – 19.2 kcal/mol), rendering it essentially irreversible and establishing **A** as the resting state of the process. This is consistent with experimental observations showing that, in the presence of water, **A** undergoes hydrolysis to give boronic acid derivative **6** (Figure 2a and Scheme 1d).

From **A**, a cationic boryl migration occurs via **TS3**, from which the electrons from the heterolytically cleaved B–C σ-bond are redistributed to the π-system, resulting in rearomatization of the central ring. This process leads to the B–C bond cleavage and the formation of π-complex **Pi** (Figure 2a), where the cationic boryl is coordinated by the adjacent phenyl π-system. Deprotonation of the C–H bond associated with the boryl-coordinated unit in **Pi** by the free triflate anion occurs via **TS4**, with an activation barrier of 19.1 kcal/mol relative to **A**, yielding intermediate **B**. We also explored several alternative rearrangement pathways originating from the π-complex, including triflate bonding to the π-bonded ring and π-bond rearrangement in **Pi** to generate Wheland intermediates. However, all such structures reverted to the π-complex **Pi** upon optimization, likely due to the extensive π-conjugation that stabilizes the π-complex geometry. The transformation from **A** to **B** via **TS4** is rate-determining.

Similar to the formation of **V4** from **S** with the first molecule of DDQ, the radical zwitterion **V7** (Figure 2b) is generated from **B** with a second molecule of DDQ. Then, the second SET via **TS5** leads to a new zwitterion **B2**, in which the +2 charge is delocalized across a triannular π-system as indicated by the lowest unoccupied molecular orbital (LUMO) plot (Figure S11). **B2** then undergoes the C–C bond formation, followed by the elimination of B­(OTf)_3_ via the low-energy transition state **TS6**, with Δ*G* ^‡^ = 3.7 kcal/mol. This facile step leads to the final π-extended product **P** bearing a pentagon–heptagon pair with an overall free energy change of −102.0 kcal/mol. Notably, the intermediate **B**, which contains an 8-membered ring, is more easily being oxidized by DDQ than azaborine **S**.

### Substrate Scope Investigation

With the optimized conditions in hand, the substrate scope, with respect to the substituents, was first evaluated. As shown in Scheme 2a, the BDA reaction tolerated a diverse range of substrates (**7a**–**7p**) bearing different functional groups. Critically, halogen atoms (Cl, Br; **8a**, **8h**, **8i**) remained intact, providing valuable handles for further derivatization. Alkyl chains and tethered aryl rings (**8b**-**8f**) likewise proved compatible, demonstrating the BDA reactions’ robustness. Notably, substrates **7k**–**7p** underwent complete conversion within 30 min, affording products **8k**–**8p** in moderate to excellent yields. This rapid transformation accommodated substrates featuring additional heteroatoms, including oxygen (**8k**), nitrogen (**8l**, **8m**), and sulfur (**8n**, **8o**) atoms, under such strong oxidative and acidic conditions. Finally, the structure of naphthalene-fused product **8p** was unambiguously confirmed by SC-XRD analysis.

**Scheme 2:**
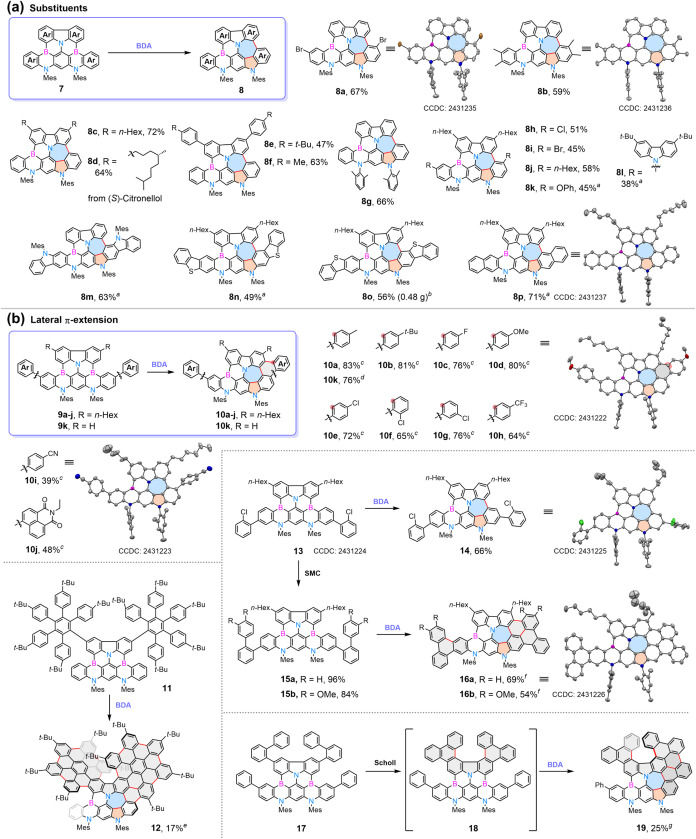
Evaluation of Substitutes and Lateral π-Extension following BDA Reaction[Fn s2fn8]

a Thirty minutes.
b 0.82 mmol scale, 30 min.
c DDQ (4.0 equiv) was used, 30 min.
d 0.40 mmol scale, DDQ (4.0 equiv) was used, 30 min.
e DDQ (18.0 equiv) was used, 12 h.
f DDQ (6.0 equiv) was used, 30 min.
g DDQ (7.5 equiv) was used, 12 h.
h
**Reaction condition**: substrate (0.05 mmol), DDQ (0.15 mmol), HOTf (150 μL), DCM (1.5 mL), rt, 12 h. Mes, mesityl; *n*-Hex, *n*-hexyl; *t*-Bu, *tert*-butyl; Ar, aromatic; BDA, boron-deleting annulation; SMC, Suzuki-Miyaura coupling; Scholl, Scholl reaction.

Given the reagent similarity between this newly developed BDA reaction and conventional Scholl reaction,^25,45,47–50^ we explored substrates **9a**–**9k** containing two peripheral aromatic rings to assess potential further cyclodehydrogenation (Scheme 2b). To our delight, when the amount of DDQ was increased to 4.0 equiv, the desired products **10a**-**10h** and **10k** were obtained in moderate to good yields. The formation of the aza-heptagon–pentagon pair and the additional hexagonal ring of **10d** was unambiguously confirmed by SC-XRD analysis. However, the further Scholl type cyclodehydrogenation reaction was inhibited by strong electron-withdrawing groups on the peripheral aromatic rings (**10i** and **10j**), even with excess DDQ.

When pentaaryl benzene rings were connected to the carbazole moiety of **11** (Scheme 2b), we envisioned that the primary cyclodehydrogenation reaction could lead to the formation of two hexa-*peri*-benzocoronene (HBC) blades. Subsequent BDA reaction of the azaborine moiety could generate 5–7 pairs, creating an additional reaction site for final cyclodehydrogenation. To our delight, this cascade yielded helical nanographene **12** featuring 15 newly formed C–C bonds in 17% yield. When this strategy was applied to biphenyl-tethered substrates **15a** and **15b**, consecutive Scholl and BDA reactions furnished **16a** and **16b** in 69% and 54% yield, respectively. The formation of the terphenyl moieties of **16a** was also confirmed by its crystal structure. Extending this approach, when biphenyl was connected to carbazole, the Scholl reaction of **17** generated [7]­helicene moiety in **18**, which underwent the BDA reaction to afford **19** as a cut-out of **12**.

### Synthetic Derivatization and Product Elaboration

To illustrate the BDA reaction’s capacity for expanding accessible chemical space, synthetic derivatization and product elaboration were further conducted (Scheme 3). First, alkyl chain was installed to the MR-core via the nitrogen-deletion reaction of **20**.^51,52^ Subsequent BDA reaction of **21** afforded a heptagon–pentagon-embedded backbone, which was further functionalized via Pd-catalyzed amination to attach a chiral dehydroabietylamine pendant. Notably, the BDA reaction provides a robust platform for efficient synthesis of π-conjugated azaborine nanographenes. As a proof-of-concept, BDA-triggered zipping-up cyclization smoothly furnished nanographenes **25a** and **25b**. Finally, the BDA products with chloride (**5c**, **10f**, and **10g**) were further transformed by transition metal-catalyzed coupling reactions to introduce alkene (**26**), (methylthio)­arene (**27**), alkyne (**33**, **35**), and amine (**29**-**32**) functionalities, which were incompatible with the direct BDA protocol. The electron-rich alkyne **35** underwent a click reaction with tetracyanoethylene (TCE) to afford the adduct **36.** ^53^

**Scheme 3:**
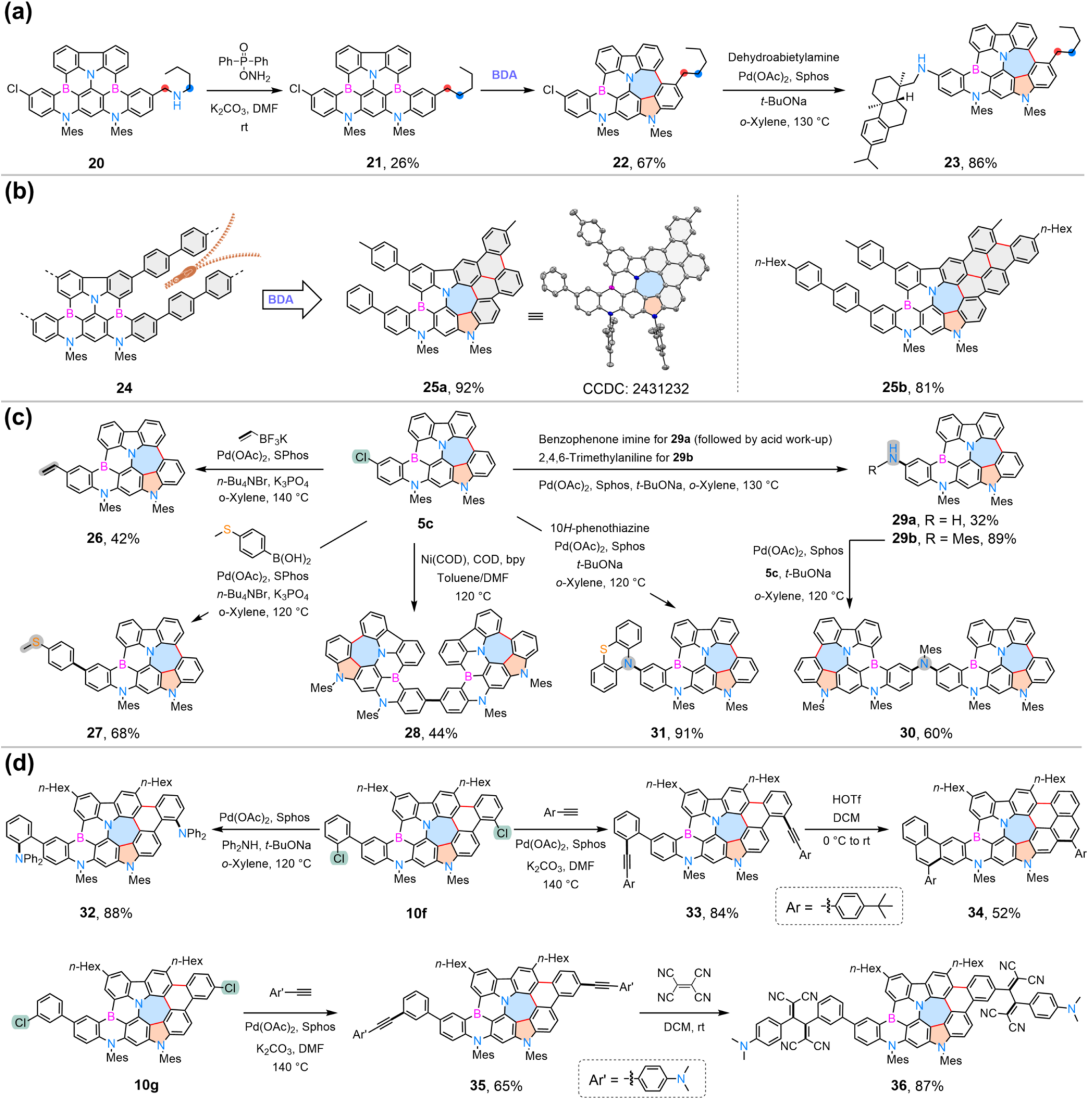
Synthetic Derivatization and Product Elaboration^a^

a Sequential nitrogen- and boron-deleting reaction (a). BDA reaction triggered fusion for zipping-up of nanographenes (b). Transformation of asymmetric BDA product (c). Product elaboration (d). BDA reactions with different amounts of DDQ: 3.0 equiv. toward **22**, 5.0 equiv. toward **25a**, 6.0 equiv. toward **25b**. Yields were given for isolated products. Sphos, 2-dicyclohexylphosphino-2′,6′-dimethoxybiphenyl; COD, 1,5-cyclooctadiene; bpy, 2,2′-bipyridine.

Building on the facile cascade reconstruction of π-conjugated nonalternant azaborines, we systematically investigated their photophysical properties. For selected examples, the structural and electronic properties of **2**, **10k**, and **25a** were further investigated using time-dependent DFT calculation on PBE0/6-311G­(d) level (Table S18). The highest occupied molecular orbital (HOMO) and LUMO wave functions are alternatively separated, resulting in the short-distance MR effect (Figure 3a), i.e., narrowband emission with small full width at half-maximum (fwhm) values (22 nm for **2**, 22 nm for **10k**, and 20 nm for **25a**). As the conjugation increased from **2** to **10k** and **25a**, narrowed HOMO–LUMO gaps and red-shifted emission peaks were observed (Figure 3b). We observed that all nonalternant azaborines displayed red-shifted fluorescence compared to their intact precursors (see Supporting Information Section 8.1). Most of the BDA products exhibited intense, narrowband fluorescence with a high photoluminescence quantum yield (PLQY, Table S19). For example, due to π-extension or peripheral functionalization of the model BDA product **2**, the emission bands gradually red-shifted (526, 538, 545, 549, 560, 570, 580, and 597 nm for **2**, **8e**, **8k**, **10k**, **25a**, **8o**, **8m**, and **19**, respectively), while maintaining small fwhm values (22, 22, 24, 22, 20, 26, 21, and 38, respectively) (Figure S63). Their emission colors spanned the visible spectrum from green to orange-red.

**Figure 3:**
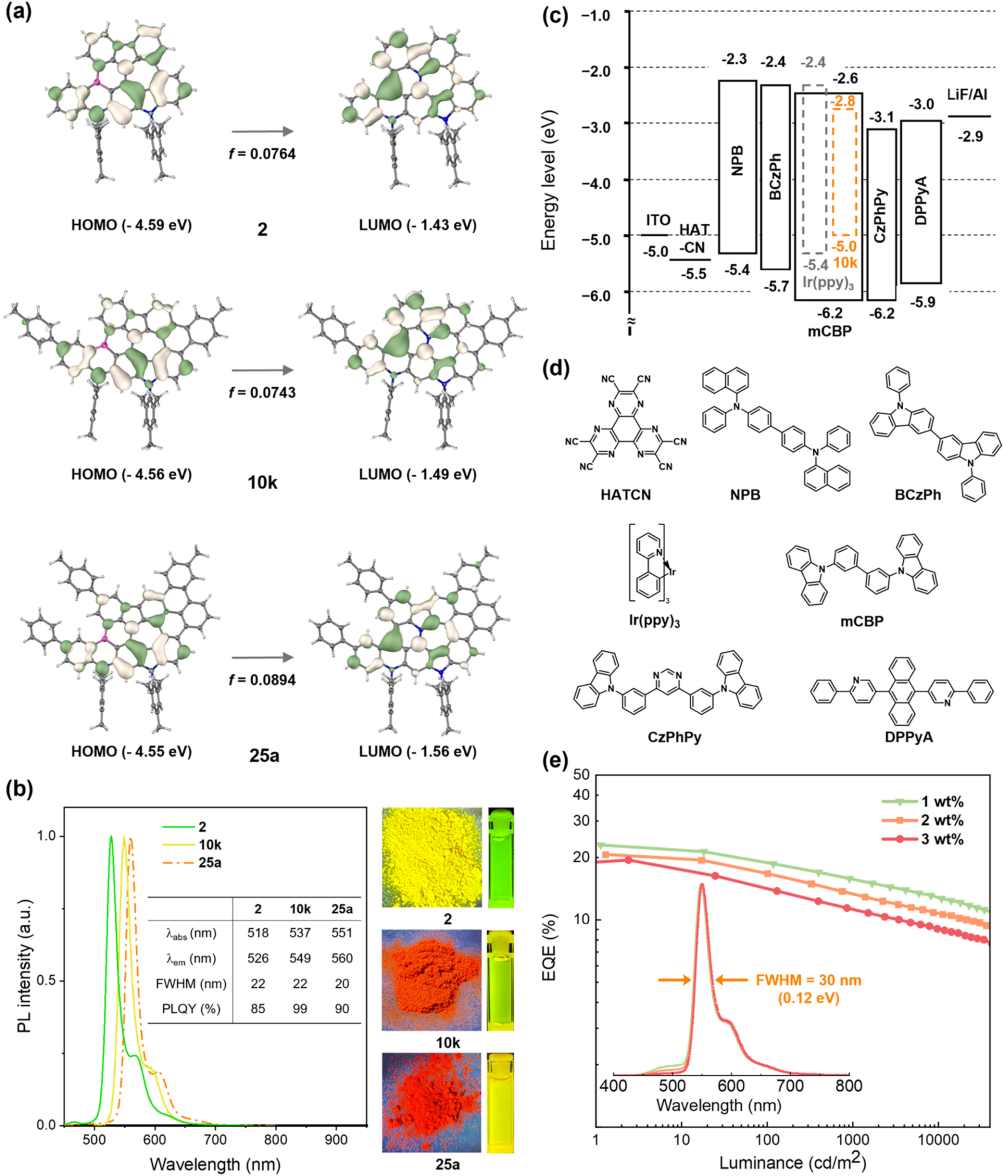
Frontier molecular orbital profiles, energies, and oscillation strength (*f*) for the HOMO → LUMO electronic transition of **2**, **10k**, and **25a** (a). Photophysical properties of **2**, **10k**, and **25a** were obtained with the images of solids and toluene solutions under 365 nm UV light irradiation (b). Device structure based on **10k** with energy-level diagrams (c). Chemical structures of materials used in the OLED devices (d). The EQE-luminance characteristics and EL spectra of **10k**-based devices (e).

### OLED Devices

Considering the near-unity PLQY and small fwhm of **10k** in toluene, we further evaluated the electroluminescence (EL) performance of **10k** as emitters. We fabricated phosphorescence-sensitized OLEDs with the following structure: ITO/HATCN (5 nm)/NPB (30 nm)/BCzPh (10 nm)/mCBP:30 wt % Ir­(ppy)_3_:1–3 wt % **10k** (30 nm)/CzPhPy (10 nm)/DPPyA (30 nm)/LiF (0.7 nm)/Al (150 nm) (Figure 3c,3d). In this architecture, mCBP served as the wide-energy-gap host; HATCN and LiF were the hole- and electron-injection layers, respectively; NPB and DPPyA functioned as the hole- and electron-transport layers; and BCzPh and CzPhPy acted as the electron- and hole-blocking layers. The phosphorescent sensitizer Ir­(ppy)_3_ was incorporated to facilitate triplet recycling to achieve high efficiency.^54^ The corresponding energy-level diagram is provided in Figure 3c. The devices exhibited EL spectra peaking at 547–551 nm with a fwhm of approximately 30 nm, suggesting high color purity yellow emission (Figure 3e). The slight bathochromic shift and broadening relative to the photoluminescence spectra are likely attributable to molecular interactions within the doped films, possibly influenced by the planar conjugated backbone of the emitter. Notably, the devices achieved high maximum EQEs of 19.5 to 23.0% and high power efficiencies (PEs) of 61.2 to 87.9 lm W^–1^ (Table S20). To the best of our knowledge, these values rank among the highest for yellow OLEDs based on conventional fluorescent emitters that do not rely on delayed fluorescence, underscoring the significant potential of BDA product **10k** for high-performance yellow electroluminescence.

## Conclusions

We present a facile synthetic approach toward nonalternant π-conjugated azaborines by cascade skeletal editing of bicyclic azaborines through a one-pot sequential boron extrusion, migration, and oxidative boron deletion process. This robust BDA protocol delivers diverse architectures (spanning planar, curved, and helical topologies) with a broad substrate scope. Through concerted boron-deleting annulation and peripheral functionalization, the photophysical properties of those π-conjugated azaborines featuring an aza-pentagon–heptagon pair have been systematically modulated. The practicality of the method has been further illustrated by consecutive Scholl reaction and zipping-up toward novel nanographenes. Furthermore, a high-performance yellow OLED was fabricated using the BDA product as a conventional fluorescent emitter in a phosphorescence-sensitized device structure, achieving remarkably high EQEs of up to 23.0%. This unprecedented reaction pathway could inspire the development of new ring manipulation methodologies, thereby expanding the accessible chemical space of functional π-systems for optoelectronic applications.

## Supplementary Material



## Notes

Experimental section, compound characterization data, computational details, and crystallographic information (PDF)
