# Supplementary material for: Facile Synthesis of Nonalternant π‑Conjugated Azaborines via Boron-Deleting Annulation
Source: J Am Chem Soc. 2026 Mar 17;148(12):13196–206. doi: 10.1021/jacs.5c23345 (PMC13047674; doi:10.1021/jacs.5c23345)
Supplement: Supplementary file 1 [file ja5c23345_si_001.pdf]

Supporting Information

Facile Synthesis of Nonalternant π-Conjugated Azaborines via Boron-Deleting Annulation

Weiwen Zhuang[1,2], Farshad Shiri[3], Faan-Fung Hung[1], Zhen Wang[1], Dongdong Zhang[4], Lian Duan[4], Chi-Ming Che[1,5], Zhenyang Lin[3*], and Junzhi Liu[1,2,5*]

[1]Department of Chemistry, The University of Hong Kong, Pokfulam Road, Hong Kong 999077, China

[2]Materials Innovation Institute for Life Sciences and Energy (MILES), HKU-SIRI, Shenzhen 518045, China

[3]Department of Chemistry, The Hong Kong University of Science and Technology, Hong Kong 999077, China

[4]Key Lab of Organic Optoelectronics and Molecular Engineering of Ministry of Education, Department of Chemistry, Tsinghua University, Beijing 100084, China

[5]State Key Laboratory of Synthetic Chemistry, HKU-CAS Joint Laboratory on New Materials and Shanghai-Hong Kong Joint Laboratory on Chemical Synthesis, The University of Hong Kong, Pokfulam Road, Hong Kong 999077, China

*Corresponding authors: chzlin@ust.hk; juliu@hku.hk

Contents

1. General information 3
2. Optimization of reaction conditions 4
3. Cascade skeletal editing of π-conjugated azaborines 7
3.1 General synthetic procedures for BDA reaction 7
3.2 Table of the unsuccessful substrates for BDA reaction 8
3.3 Characterization data for skeletal-edited π-conjugated azaborines 8
4. Synthetic derivatization and product elaboration 28
4.1 Sequential nitrogen-deletion, BDA reaction and amination reaction 28
4.2 Synthesis of nanographenes by BDA-triggered cyclodehydrogenation 29
4.3 Product elaboration 31
5. SC-XRD data 41
6. Mechanistic study 53
6.1 Control experiments 53
6.2 Electronic effects 53
6.3 Interrupted BDA reaction 56
7. DFT calculation 59
7.1 Computational details and Cartesian coordinates 59
7.2 Calculated mechanism 82
7.3 Spin natural orbitals and frontier molecular orbitals for selected species 84
8. Photophysical and electrochemical properties of skeletal-edited products 92
8.1 UV-Vis absorption and emission spectra 92
8.2 Quantum yields and lifetime 107
8.3 Electrochemical properties 113
9. Device performances 115
10. Preparation of substrates 116
10.1 Synthesis of substrates 116
10.2 Synthesis of precursors for the borylated substrates 138
11. NMR spectrum 151
12. Mass spectrum 301
13. Reference 331

1. General information

All chemicals were purchased from Dieckmann (Hong Kong), Aldrich Chemical Co. Bide Pharmatech Co., J&K Chemical, Energy Chemical. companies and used without further purification, unless otherwise stated. $Pd_2(dba)_3$, tris(dibenzylideneacetone)dipalladium(0); $Pd(OAc)_2$, palladium(II) acetate; Xantphos, (9,9-Dimethyl-9*H*-xanthene-4,5-diyl)bis(diphenylphosphane); Sphos, dicyclohexyl(2′,6′-dimethoxy[1,1′-biphenyl]-2-yl)phosphane; *t*-BuONa, sodium *tert*-butoxide; *o*-xylene, 1,2-dimethylbenzene; BBr_3, boron tribromide; DDQ, 2,3-dichloro-5,6-dicyano-1,4-benzoquinone; HOTf, trifluoromethanesulfonic acid, triflic acid; MSA, methanesulfonic acid; $BF_3{\cdot}OEt_2$, boron trifluoride diethyl etherate; TCB, 1,2,4-trichlorobenzene; Tol, toluene; Ph_2O, diphenyl ether; THF, tetrahydrofuran; DMF, dimethylformamide; DCM, dichloromethane.

Analytical thin layer chromatography (TLC) was carried out on silica-coated aluminium plates (silica gel GF254) using UV light as visualizing agent (254 or 365 nm). Nuclear magnetic resonance spectra (^{1}H NMR, ^{13}C NMR, ^{19}F NMR) were recorded with Bruker NEO 400/500/600 NMR Spectrometers. Chemical shifts were reported in parts per million (ppm, δ), downfield from tetramethyl silane (TMS, δ = 0.00 ppm), and referenced to residual solvent ($CDCl_3$, δ = 7.26 ppm for ^{1}H and 77.16 ppm for ^{13}C; acetone-*d6*, δ = 2.05 ppm for ^{1}H and 29.84 ppm for ^{13}C; DMSO-*d6*, δ = 2.50 ppm for ^{1}H and 39.52 ppm for ^{13}C); all the ^{19}F chemical shifts were not referenced. Coupling constants were reported in Hertz (Hz). Data for ^{1}H NMR spectra were reported as follows: chemical shift (ppm, referenced to protium; s = singlet, d = doublet, t = triplet, q = quartet, dd = doublet of doublets, m = multiplet, br = broad, coupling constant (Hz), and integration).

High resolution mass spectra (HRMS) were obtained on a Bruker Q-Tof Maxis II mass spectrometer, a DFS high resolution magnetic sector mass spectrometer or a Bruker Autoflex Speed MALDI TOF MS spectrometer, using *trans*-2-[3-(4-*tert*-butylphenyl)-2-methyl-2-propenylidene]malononitrile (DCTB) or tetracyanoquinodimethane (TCNQ) as a matrix. The electrochemical measurements were conducted in a conventional three-electrode cell on a CHI-760E electrochemical workstation (Shanghai Chenhua Instrument Co., Ltd, China). Absorption spectra were measured with Agilent Technologies Cary 60 UV-Vis spectrophotometer in a 1-cm quartz cell. Fluorescence measurements were measured with Agilent Technologies Cary Eclipse fluorescence spectrophotometer at room temperature. Photoluminescence quantum yields (Φ_{PL}) were determined by HAMAMATSU Quantaurus-QY instrument at room temperature. Time-resolved photoluminescence decays were measured with HAMAMATSU Quantaurus-Tau Fluorescence lifetime spectrometer C16361. Circular dichroism (CD) spectra and circularly polarized luminescence (CPL) spectra were collected on Applied Photophysics Chirascan-v100.

Purchased 2,3-dichloro-5,6-dicyano-1,4-benzoquinone (DDQ) was dissolved in anhydrous dichloromethane, and dried over sodium sulfate with vigorous stirring under nitrogen atmosphere. The suspension was filtered through a pad of oven-dried silica gel. The solvent in the filtrate was removed in vacuo. The obtained bright yellow solid was further dried under vacuum at 50 °C for 5 days and used for the BDA reaction.

Model compound **1**[S1], 3,6-di([1,1'-biphenyl]-2-yl)-9H-carbazole[S2], and (*S*)-4,8-dimethylnon-1-ene[S3] were prepared following the according references.

2. Optimization of reaction conditions

Reaction development:

Compound **1** (34.0 mg, 0.05 mmol, 1.0 equiv.) was added to a 20 mL vial, then dried under vacuum for 30 min. DDQ (34 mg, 0.15 mmol, 3.0 equiv.) (other oxidants if applicable) was added to the vial and further dried under vacuum. The vial was then sealed and purged with nitrogen flow. Anhydrous solvent (1.5 mL) was injected via a syringe. Acid (150 uL for liquid, 20.0 equiv. for solid) and additive (if applicable) were finally added. The vial was further sealed with electrical tape and stirred at room temperature (variation if applicable) for 12 h. The reaction mixture was poured into saturated Na_2CO_3 solution and extracted with DCM. The organic phase was dried over Na_2SO_4. After removal of solvent, the residue was analyzed by NMR spectroscopy with 1,3,5-trimethoxybenzene as the internal standard [Optimal result: 82% yield. Variations see Tables S1-S5].

Table S1. Evaluation of acids

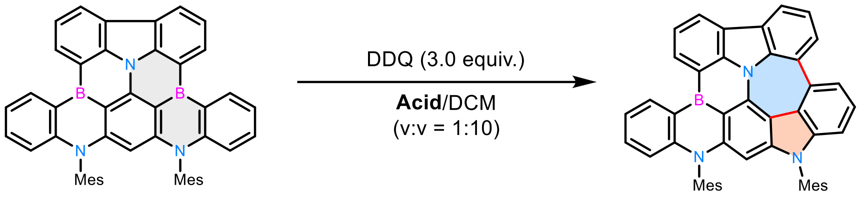

1 **2**

Entry	Acid	Yield (%)
1	MsOH	N.D.
2	TsOH*	N.D.
3	H_2SO_4	N.D.
4	AcOH	N.D.
5	CF_3CO_2H	N.D.
6	BF_3•OEt_2	N.D.
7	$Sc(OTf)_3$*	N.D.
8	Tf_2NH	< 5%

*20.0 equiv. of TsOH or $Sc(OTf)_3$ were used.

Table S2. Evaluation of solvents

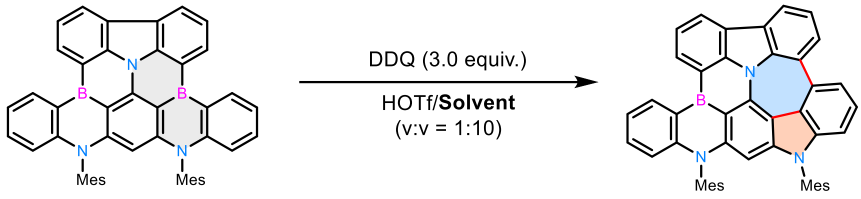

1 **2**

Entry	Solvent	Yield (%)
1	DCE	43
2	$CHCl_3$	11
3	Toluene	N.D.
4	PhCl	Trace
5	Hexane	N.D.
6	MeCN	N.D.
7	HFIP	N.D.

Table S3. Evaluation of oxidants

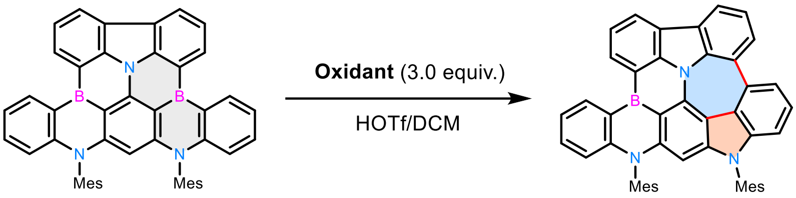

1 **2**

Oxidant	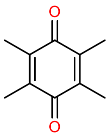	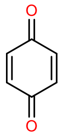	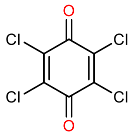	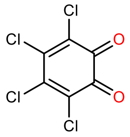
Result	N.D.	N.D.	N.D.	N.D.

Table S4. Evaluation of additives

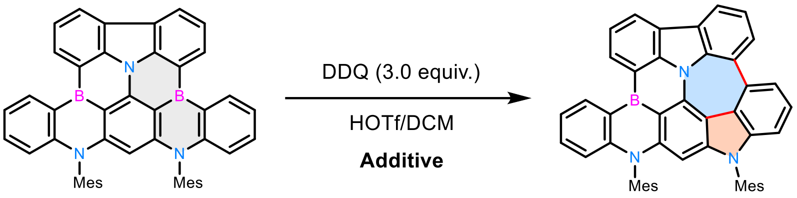

1 **2**

Additive (3.0 eq.)	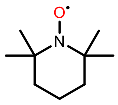	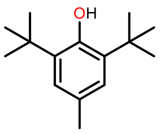	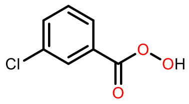
Result	Trace	Trace	Trace

Table S5. Evaluation of temperatures

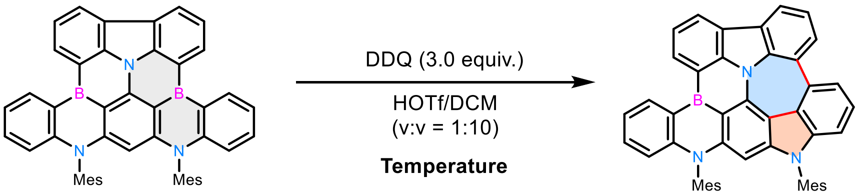

1 **2**

Entry	Temperature (°C)	Yield (%)
1	-10	N.D.
2	0	Trace
3	10	< 5%
4	30	73

3. Cascade skeletal editing of π-conjugated azaborines

3.1 General synthetic procedures for BDA reaction

General Procedure A

To a 20 mL vial was added the substrate (0.05 mmol, 1.0 equiv.), then dried under vacuum for 30 min. DDQ (0.15 mmol, 3.0 equiv.) was added to the vial and further dried under vacuum. The vial was then sealed and purged with nitrogen flow. Anhydrous DCM (1.5 mL) was injected via a syringe. HOTf (150 uL) was finally injected via a micro-syringe with stainless needle. The vial was further sealed with electrical tape and stirred at room temperature for 12 h (for **1**, **3a-3e**, **3b'**, **7a-7j**, **13** and **21**) or 30 min (**7k-7p**). The reaction mixture was poured into saturated Na_2CO_3 solution and extracted with DCM. The organic phase was dried over Na_2SO_4. After removal of solvent, the residue was purified by column chromatography using hexane and DCM as the eluents to give products **2**, **4a-4d** or **5a-5d**, **4b'**, **8a-8p**, **14**, **22**.

General Procedure B

To a 20 mL vial was added the substrate (0.05 mmol, 1.0 equiv.), then dried under vacuum for 30 min. DDQ (0.2 mmol, 4.0 equiv.) was added to the vial and further dried under vacuum. The vial was then sealed and purged with nitrogen flow. Anhydrous DCM (5.0 mL) was injected via a syringe. HOTf (250 uL) was finally injected via a micro-syringe with stainless needle. The vial was further sealed with electrical tape and stirred at room temperature for 30 min. The reaction mixture was poured into saturated Na_2CO_3 solution and extracted with DCM. The organic phase was dried over Na_2SO_4. After removal of solvent, the residue was purified by column chromatography using hexane and DCM as the eluents to give products **10a-10k**.

General Procedure C

To a 20 mL vial was added the substrate (0.05 mmol, 1.0 equiv.), then dried under vacuum for 30 min. DDQ (variable equiv.) was added to the vial and further dried under vacuum. The vial was then sealed and purged with nitrogen flow. Anhydrous DCM (5.0 mL) was injected via a syringe. HOTf (250 uL) was finally injected via a micro-syringe with stainless needle. The vial was further sealed with electrical tape and stirred at room temperature and monitored by TLC. The reaction mixture was poured into saturated Na_2CO_3 solution and extracted with DCM. The organic phase was dried over Na_2SO_4. After removal of solvent, the residue was purified by column chromatography using hexane and DCM as the eluents to give products **12** (18.0 equiv. DDQ), **16a** (6.0 equiv. DDQ), **16b** (6.0 equiv. DDQ), **19** (7.5 equiv. DDQ), **25a** (5.0 equiv. DDQ) or **25b** (6.0 equiv. DDQ).

3.2 Table of the unsuccessful substrates for BDA reaction

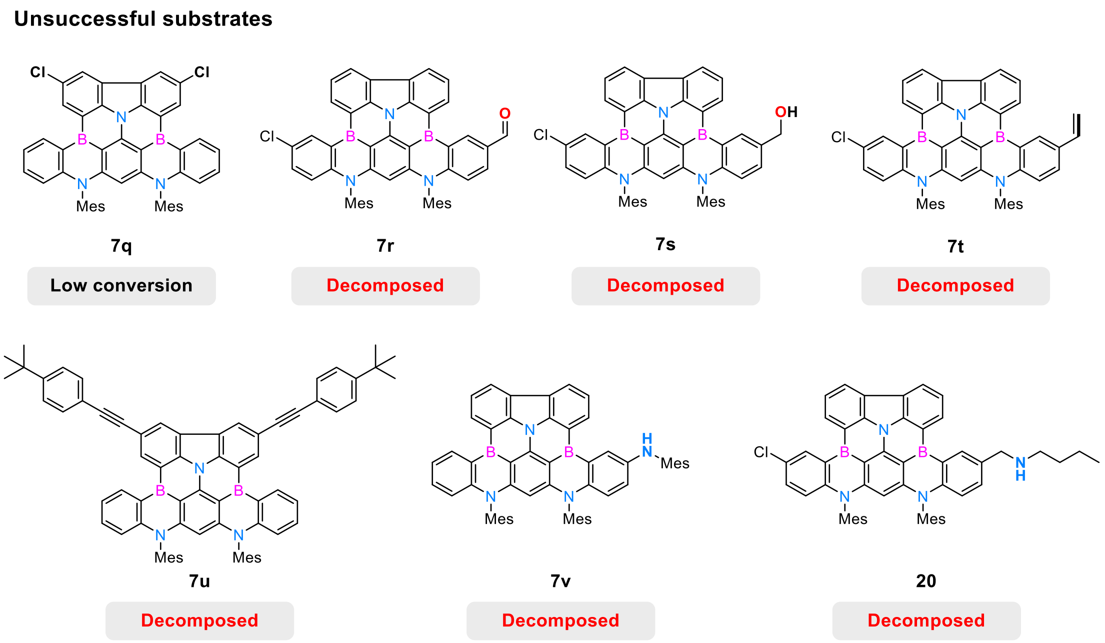

3.3 Characterization data for skeletal-edited π-conjugated azaborines

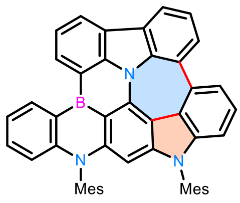

2

2 (26.1 mg, 78% yield, yellow solid) was synthesized from **1** (34.0 mg, 0.05 mmol, 1.0 equiv.) following **General Procedure A**. Purification by column chromatography on silica gel (hexane/DCM = 20/1 – 4/1). Single crystal was grown by slow diffusion of methanol to the toluene solution of **2**.

^{1}H NMR (600 MHz, $CDCl_3$) δ 9.00 (d, J = 7.6 Hz, 1H), 8.91 (d, J = 7.4 Hz, 1H), 8.16 (d, J = 7.4 Hz, 1H), 7.81 (t, J = 7.9 Hz, 2H), 7.59 (t, J = 7.4 Hz, 1H), 7.43 (d, J = 7.8 Hz, 1H), 7.41 – 7.37 (m, 1H), 7.22 (t, J = 7.2 Hz, 1H), 7.15 (t, J = 7.6 Hz, 1H), 7.04 (t, J = 7.8 Hz, 1H), 6.99 (s, 2H), 6.93 (s, 2H), 6.60 (d, J = 8.6 Hz, 1H), 6.49 (d, J = 7.9 Hz, 1H), 5.29 (s, 1H), 2.34 (s, 3H), 2.32 (s, 3H), 1.79 (d, J = 2.1 Hz, 12H).

^{13}C NMR (151 MHz, Acetone-*d6*/CS_2) δ 146.90, 146.72, 145.37, 142.72, 142.38, 141.95, 141.27, 139.30, 139.07, 137.51, 137.38, 137.25, 136.33, 133.89, 132.96, 131.80, 131.54, 131.00, 130.13, 129.60, 128.21, 127.44, 124.97, 124.59, 123.66, 123.15, 123.09, 121.84, 120.98, 116.92, 115.76, 110.25, 106.90, 88.45, 68.14, 21.56, 17.78, 17.65.

^{11}B NMR (128 MHz, $CDCl_3$) δ 37.6.

HRMS (MALDI-TOF) m/z: $[M]^+$ Calculated for $C_{48}H_{36}BN_3$ 665.3005; Found 665.3135 (19.5 ppm).

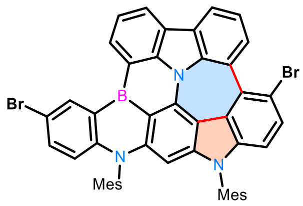

8a

8a (27.5 mg, 67% yield, brown solid) was synthesized from **7a** (41.8 mg, 0.05 mmol, 1.0 equiv.) following **General Procedure A**. Purification by column chromatography on silica gel (hexane/DCM = 15/1 − 4/1). Single crystal was grown by slow diffusion of methanol to the toluene solution of **8a**.

^{1}H NMR (400 MHz, $CDCl_3$) δ 9.05 (d, *J* = 2.4 Hz, 1H), 8.80 (d, *J* = 7.5 Hz, 1H), 8.32 (dd, *J* = 7.9, 1.2 Hz, 1H), 8.20 (d, *J* = 7.4 Hz, 1H), 7.87 (dd, *J* = 7.6, 1.3 Hz, 1H), 7.65 (t, *J* = 7.5 Hz, 1H), 7.44 (dd, *J* = 9.1, 2.4 Hz, 1H), 7.38 (d, *J* = 8.4 Hz, 1H), 7.29 (d, *J* = 7.7 Hz, 1H), 6.98 (s, 2H), 6.93 (s, 2H), 6.47 (d, *J* = 9.1 Hz, 1H), 6.38 (d, *J* = 8.5 Hz, 1H), 5.27 (s, 1H), 2.34 (s, 3H), 2.32 (s, 3H), 1.77 (s, 6H)1.76 (s, 6H).

^{13}C NMR (101 MHz, $CDCl_3$) δ 146.28, 145.37, 144.93, 142.59, 141.61, 139.61, 138.83, 138.58, 137.72, 137.05, 136.67, 136.31, 134.78, 134.05, 132.69, 132.19, 131.02, 130.50, 130.26, 129.29, 128.79, 127.71, 126.68, 123.75, 123.26, 122.73, 122.44, 121.50, 117.31, 113.40, 110.85, 110.22, 106.19, 87.74, 29.86, 21.20, 21.13, 17.42, 17.26.

^{11}B NMR (128 MHz, $CDCl_3$) δ 38.4.

HRMS (MALDI-TOF) m/z: $[M]^+$ Calculated for $C_{48}H_{34}BBr_2N_3$ 823.1200; Found 823.1255 (6.7 ppm).

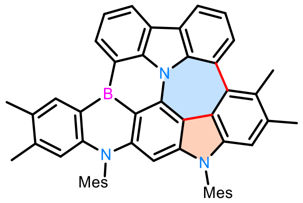

8b

8b (21.3 mg, 59% yield, yellow solid) was synthesized from **7b** (37.0 mg, 0.05 mmol, 1.0 equiv.) following **General Procedure A**. Purification by column chromatography on silica gel (hexane/DCM = 15/1 − 6/1). Single crystal was grown by slow diffusion of methanol to the toluene solution of **8b**.

^{1}H NMR (600 MHz, C_6D_6) δ 9.24 – 9.20 (m, 1H), 9.10 (s, 1H), 8.04 (dd, *J* = 7.5, 1.1 Hz, 1H), 7.68 (dd, *J* = 7.5, 1.2 Hz, 1H), 7.57 (t, *J* = 7.4 Hz, 1H), 7.44 (dd, *J* = 7.7, 1.3 Hz, 1H), 7.12 (t, *J* = 7.6 Hz, 1H), 6.72 (s, 2H), 6.68 – 6.67 (m, 3H), 6.51 (s, 1H), 5.74 (s, 1H), 2.42 (s, 3H), 2.28 (s, 3H), 2.08 (s, 3H), 2.06 (s, 3H), 2.04 (s, 3H), 1.99 (s, 3H), 1.89 (s, 6H), 1.84 (s, 6H).

^{13}C NMR (151 MHz, C_6D_6) δ 146.31, 145.67, 145.33, 143.77, 142.60, 142.57, 141.67, 139.00, 138.22, 137.93, 137.80, 137.74, 137.55, 137.25, 136.88, 133.54, 133.38, 132.07, 131.38, 130.60, 129.66, 129.34, 129.24, 128.62, 128.35, 126.88, 124.98, 124.42, 123.60, 123.21, 122.53, 122.09, 122.04, 120.28, 115.89, 110.31, 107.18, 87.51, 21.94, 21.04, 21.01, 20.85, 20.68, 19.41, 17.55, 17.48.

^{11}B NMR (128 MHz, $CDCl_3/CS_2$) δ 39.7.

HRMS (MALDI-TOF) m/z: $[M]^+$ Calculated for $C_{52}H_{44}BN_3$ 721.3631; Found 721.3681 (6.9 ppm).

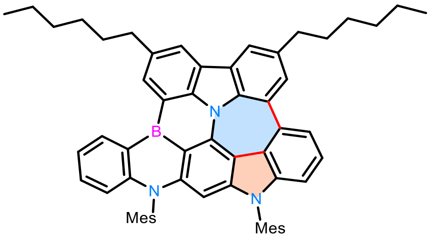

8c

8c (29.9 mg, 72% yield, yellow solid) was synthesized from **7c** (42.0 mg, 0.1 mmol, 1.0 equiv.) following **General Procedure A**. Purification by column chromatography on silica gel (hexane/DCM = 10/1 − 6/1).

^{1}H NMR (400 MHz, $CDCl_3$) δ 9.00 (d, *J* = 7.7 Hz, 1H), 8.69 (s, 1H), 7.96 (s, 1H), 7.58 (d, *J* = 7.0 Hz, 2H), 7.48 – 7.35 (m, 2H), 7.22 (d, *J* = 7.2 Hz, 1H), 7.02 (t, *J* = 7.8 Hz, 1H), 6.98 (s, 2H), 6.92 (s, 2H), 6.58 (d, *J* = 8.6 Hz, 1H), 6.47 (d, *J* = 7.9 Hz, 1H), 5.24 (s, 1H), 2.97 (t, *J* = 7.8 Hz, 2H), 2.70 (t, *J* = 7.7 Hz, 2H), 2.34 (s, 3H), 2.32 (s, 3H), 1.87 – 1.72 (m, 16H), 1.52 – 1.31 (m, 12H), 0.92 (t, *J* = 7.1Hz, 6H).

^{13}C NMR (101 MHz, $CDCl_3$) δ 146.37, 146.23, 144.71, 142.54, 142.05, 140.57, 140.31, 138.33, 138.18, 138.11, 137.15, 136.92, 136.89, 136.42, 135.66, 133.28, 132.01, 131.28, 131.09, 130.07, 129.13, 129.08, 127.13, 126.04, 124.73, 124.28, 123.13, 122.10, 120.29, 119.86, 115.65, 115.15, 109.47, 105.94, 87.33, 36.71, 35.85, 32.67, 32.05, 32.00, 31.65, 29.31, 29.21, 22.87, 22.83, 21.19, 21.12, 17.46, 17.34, 14.34, 14.32.

^{11}B NMR (128 MHz, $CDCl_3/CS_2$) δ 38.3.

HRMS (MALDI-TOF) m/z: $[M]^+$ Calculated for $C_{60}H_{60}BN_3$ 833.4885; Found 833.4868 (-2.0 ppm).

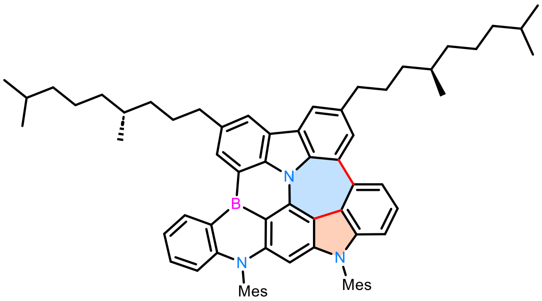

8d

8d (31.2 mg, 64% yield, orange-yellow solid) was synthesized from **7d** (49.5 mg, 0.05 mmol, 1.0 equiv.) following **General Procedure A**. Purification by column chromatography on silica gel (hexane/DCM = 10/1 − 8/1).

^{1}H NMR (400 MHz, $CDCl_3$) δ 9.00 (d, *J* = 7.8 Hz, 1H), 8.69 (s, 1H), 7.97 (s, 1H), 7.59 (d, *J* = 6.4 Hz, 2H), 7.45 – 7.35 (m, 2H), 7.03 – 6.89 (m, 5H), 6.58 (d, *J* = 8.6 Hz, 1H), 6.47 (d, *J* = 7.9 Hz, 1H), 5.24 (s, 1H), 2.96 (m, 2H), 2.68 (m, 2H), 2.33 (d, *J* = 7.6 Hz, 6H), 1.79 (s, 14H), 1.54 – 1.07 (m, 30H), 1.00 – 0.79 (m, 22H).

^{13}C NMR (101 MHz, $CDCl_3$) δ 146.20, 140.55, 138.32, 138.21, 138.11, 137.15, 136.92, 135.66, 133.25, 132.02, 131.09, 130.07, 129.14, 127.14, 124.27, 123.14, 122.10, 120.30, 119.88, 115.15, 109.44, 87.34, 39.50, 37.46, 37.44, 37.08, 36.96, 36.16, 34.90, 34.57, 32.97, 32.92, 30.26, 29.86, 29.51, 29.22, 28.49, 28.12, 25.00, 23.00, 22.89, 22.78, 21.19, 21.12, 19.92, 19.53, 19.40, 17.46,

17.34, 17.24.

^{11}B NMR (128 MHz, $CDCl_3/CS_2$) δ 38.3.

HRMS (MALDI-TOF) m/z: $[M]^+$ Calculated for $C_{70}H_{80}BN_3$ 973.6451; Found 973.6341 (-11.2 ppm).

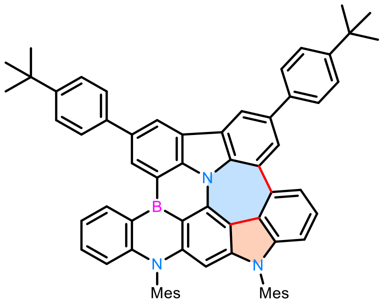

8e

8e (21.6 mg, 47% yield, orange solid) was synthesized from **7e** (47.0 mg, 0.05 mmol, 1.0 equiv.) following **General Procedure A**. Purification by column chromatography on silica gel (hexane/DCM = 10/1 − 4/1).

^{1}H NMR (400 MHz, $CDCl_3$) δ 9.13 (s, 1H), 9.05 (d, J = 7.7 Hz, 1H), 8.42 (s, 1H), 8.20 – 8.00 (m, 2H), 7.89 (d, J = 7.6 Hz, 1H), 7.79 (d, J = 7.4 Hz, 2H), 7.70 – 7.29 (m, 8H), 7.20 – 6.89 (m, 5H), 6.63 (d, J = 8.7 Hz, 1H), 6.53 (d, J = 7.9 Hz, 1H), 5.34 (s, 1H), 2.35 – 2.34 (m, 6H), 1.81 (s, 12H), 1.56 (s, 9H), 1.26 (s, 9H).

^{13}C NMR (101 MHz, $CDCl_3$) δ 146.33, 144.89, 142.59, 141.10, 140.77, 138.48, 138.26, 137.13, 136.87, 135.62, 132.56, 132.29, 130.96, 130.13, 129.72, 129.20, 129.08, 129.01, 127.90, 127.40, 127.01, 126.41, 123.57, 123.22, 121.16, 120.16, 119.24, 115.94, 115.33, 109.85, 106.17, 93.24, 87.93, 29.85, 21.20, 21.13, 17.48, 17.35.

^{11}B NMR (128 MHz, $CDCl_3/CS_2$) δ 40.3.

HRMS (MALDI-TOF) m/z: $[M]^+$ Calculated for $C_{68}H_{60}BN_3$ 929.4886; Found 929.4781 (-11.3 ppm).

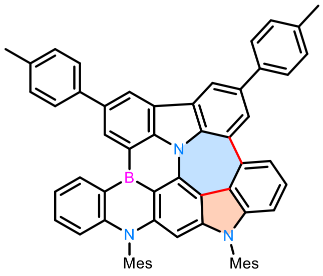

8f

8f (26.5 mg, 63% yield, orange solid) was synthesized from **7f** (43.1 mg, 0.05 mmol, 1.0 equiv.) following **General Procedure A**. Purification by column chromatography on silica gel (hexane/DCM = 10/1 − 3/1).

^{1}H NMR (400 MHz, $CDCl_3$) δ 9.11 (s, 1H), 9.05 (d, J = 7.7 Hz, 1H), 8.39 (s, 1H), 8.04 (d, J = 7.3 Hz, 2H), 7.79 (d, J = 7.6 Hz, 2H), 7.68 (d, J = 7.6 Hz, 2H), 7.53 (d, J = 7.9 Hz, 1H), 7.47 – 7.30 (m, 5H), 7.22 (d, J = 7.5 Hz, 1H), 7.08 (t, J = 8.0 Hz, 1H), 7.00 (s, 2H), 6.94 (s, 2H), 6.62 (d, J = 8.6 Hz, 1H), 6.53 (d, J = 7.9 Hz, 1H), 5.33 (s, 1H), 2.56 – 2.28 (m, 12H), 1.94 – 1.73 (m, 12H).

^{13}C NMR (101 MHz, $CDCl_3$) δ 140.74, 139.73, 137.13, 136.88, 136.84, 136.68, 135.64,

132.39, 132.23, 130.99, 130.12, 129.79, 129.71, 129.56, 129.19, 127.80, 127.73, 126.83, 126.36, 123.58, 123.05, 120.97, 120.11, 118.99, 115.91, 115.28, 109.78, 53.58, 21.33, 21.20, 21.13, 17.48, 17.35.

^{11}B NMR (128 MHz, $CDCl_3/CS_2$) δ 42.2.

HRMS (MALDI-TOF) m/z: $[M]^+$ Calculated for $C_{62}H_{48}BN_3$ 845.3936; Found 845.3903 (-3.9 ppm).

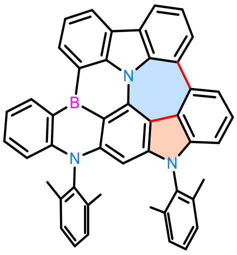

8g

8g (21.8 mg, 66% yield, orange solid) was synthesized from **7g** (33.5 mg, 0.05 mmol, 1.0 equiv.) following **General Procedure A**. Purification by column chromatography on silica gel (hexane/DCM = 10/1 − 2/1).

^{1}H NMR (600 MHz, Acetone-*d6*/CS_2) δ 8.97 (d, J = 7.7 Hz, 1H), 8.90 (d, J = 7.5 Hz, 1H), 8.21 (d, J = 7.5 Hz, 1H), 7.85 (t, J = 7.1 Hz, 2H), 7.60 (t, J = 7.5 Hz, 1H), 7.46 (d, J = 7.8 Hz, 1H), 7.38 (t, J = 7.9 Hz, 1H), 7.32 – 7.14 (m, 8H), 7.07 (t, J = 8.0 Hz, 1H), 6.55 – 6.41 (m, 2H), 5.38 (s, 1H), 1.89 (s, 12H).

^{13}C NMR (151 MHz, Acetone-*d6*/CS_2) δ 146.65, 146.41, 145.09, 142.73, 142.33, 141.89, 141.00, 139.85, 137.81, 137.61, 136.35, 134.04, 133.87, 132.97, 131.82, 130.25, 130.21, 129.72, 129.57, 129.40, 129.37, 129.33, 128.12, 127.45, 124.95, 124.56, 123.62, 123.15, 123.06, 121.81, 121.05, 116.97, 115.58, 110.15, 106.93, 88.19, 17.85, 17.73.

^{11}B NMR (128 MHz, $CDCl_3$) δ 41.7.

HRMS (MALDI-TOF) m/z: $[M]^+$ Calculated for $C_{42}H_{20}BN_3$ 637.2692; Found 637.2779 (13.6 ppm).

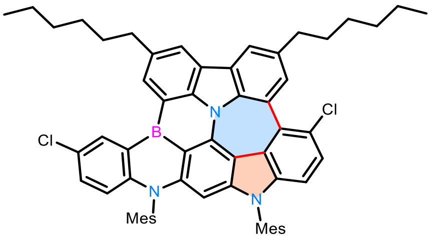

8h

8h (22.9 mg, 51% yield, orange solid) was synthesized from **7h** (46.6 mg, 0.05 mmol, 1.0 equiv.) following **General Procedure A**. Purification by column chromatography on silica gel (hexane/DCM = 10/1 − 4/1).

^{1}H NMR (400 MHz, $CDCl_3$) δ 8.89 (d, J = 2.6 Hz, 1H), 8.57 (s, 1H), 8.17 (d, J = 1.7 Hz, 1H), 8.00 (s, 1H), 7.63 (d, J = 1.7 Hz, 1H), 7.30 (dd, J = 9.1, 2.5 Hz, 1H), 7.14 (d, J = 8.5 Hz, 1H), 6.97 (s, 2H), 6.93 (s, 2H), 6.50 (d, J = 9.1 Hz, 1H), 6.41 (d, J = 8.5 Hz, 1H), 5.22 (s, 1H), 2.99 (t, J = 7.7 Hz, 2H), 2.73 (t, J = 7.7 Hz, 2H), 2.34 (s, 3H), 2.32 (s, 3H), 1.89 – 1.71 (m, 14H), 1.52 – 1.30 (m, 14H), 0.92 (q, J = 8.3 Hz, 6H).

^{13}C NMR (101 MHz, $CDCl_3$) δ 150.18, 146.34, 145.37, 144.59, 144.32, 142.49, 141.03, 140.58, 140.36, 138.98, 138.71, 138.46, 138.39, 138.25, 137.08, 136.72, 136.59, 136.46, 136.01, 134.68, 132.60, 131.88, 131.50, 131.09, 130.60, 130.52, 130.40, 130.22, 129.90, 129.25, 128.96, 127.52, 126.06, 125.66, 125.53, 125.38, 124.66, 123.48, 122.78, 122.56, 122.33, 120.71, 117.50, 116.80, 114.81, 109.71, 106.11, 90.03, 87.21, 37.19, 36.60, 35.67, 32.88, 32.50, 32.05, 32.01, 31.98, 31.44, 29.85, 29.27, 29.14, 22.84, 22.82, 21.18, 21.12, 17.42, 17.27, 17.17, 14.32.

^{11}B NMR (128 MHz, $CDCl_3/CS_2$) δ 39.7.

HRMS (MALDI-TOF) m/z: $[M]^+$ Calculated for $C_{60}H_{58}BCl_2N_3$ 901.4105; Found 901.4130 (2.8 ppm).

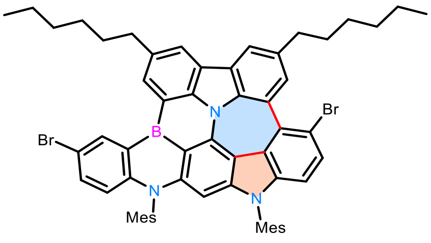

8i

8i (22.4 mg, 45% yield, brown solid) was synthesized from **7i** (50.5 mg, 0.05 mmol, 1.0 equiv.) following **General Procedure A**. Purification by column chromatography on silica gel (hexane/DCM = 10/1 − 6/1).

^{1}H NMR (600 MHz, Acetone-*d6*/CS_2) δ 8.92 (d, J = 2.4 Hz, 1H), 8.51 (s, 1H), 7.96 – 7.91 (m, 1H), 7.57 – 7.52 (m, 1H), 7.51 – 7.47 (m, 1H), 7.37 (dd, J = 8.9, 2.5 Hz, 1H), 7.24 (d, J = 8.7 Hz, 1H), 7.12 (d, J = 8.4 Hz, 1H), 6.97 (s, 2H), 6.84 (s, 2H), 6.45 (d, J = 9.0 Hz, 1H), 5.07 (s, 1H), 3.00 (t, J = 7.8 Hz, 2H), 2.72 (t, J = 7.8 Hz, 2H), 2.08 (s, 6H), 1.89 (p, J = 6.6 Hz, 3H), 1.83 – 1.73 (m, 12H), 1.57 – 1.38 (m, 12H), 0.97 (td, J = 7.1, 2.6 Hz, 6H).

^{13}C NMR (151 MHz, Acetone-*d6*/CS_2) δ 146.61, 145.87, 145.04, 142.36, 140.53, 140.44, 138.99, 138.82, 138.16, 137.82, 137.22, 136.87, 136.81, 136.67, 135.10, 133.37, 132.51, 131.25, 131.20, 130.66, 130.47, 129.67, 128.97, 127.59, 126.72, 125.18, 123.75, 123.23, 121.28, 117.90, 117.54, 114.33, 106.22, 103.85, 88.02, 37.27, 36.50, 33.18, 32.87, 32.82, 32.30, 30.69, 23.86, 23.85, 21.78, 21.65, 17.98, 17.61, 17.52, 15.13, 15.10.

^{11}B NMR (128 MHz, $CDCl_3/CS_2$) δ 39.0.

HRMS (MALDI-TOF) m/z: $[M]^+$ Calculated for $C_{60}H_{58}BBr_2N_3$ 991.3082; Found 991.3137 (5.5 ppm).

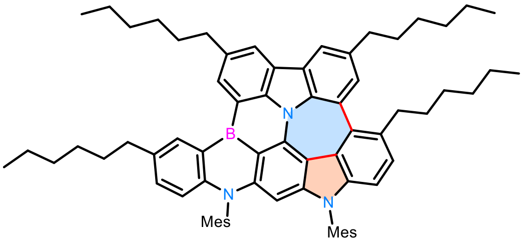

8j

8j (28.9 mg, 58% yield, yellow solid) was synthesized from **7j** (51.0 mg, 0.05 mmol, 1.0 equiv.) following **General Procedure A**. Purification by column chromatography on silica gel (hexane/DCM = 10/1 − 6/1).

^{1}H NMR (600 MHz, Acetone-*d6*/CS_2) δ 9.68 (s, 1H), 9.17 (d, *J* = 2.1 Hz, 1H), 9.02 – 8.97 (m, 1H), 8.86 – 8.82 (m, 1H), 8.42 (d, *J* = 8.3 Hz, 1H), 7.60 (dd, *J* = 8.3, 1.6 Hz, 1H), 7.42 – 7.32 (m, 2H), 7.04 (s, 3H), 6.82 (d, *J* = 8.6 Hz, 1H), 6.77 (d, *J* = 8.6 Hz, 1H), 5.34 (s, 1H), 3.29 (t, *J* = 7.8 Hz, 2H), 3.18 (t, *J* = 7.6 Hz, 2H), 2.96 (t, *J* = 7.8 Hz, 2H), 2.92 (t, *J* = 7.8 Hz, 2H), 2.51 (m, 12H), 2.13 – 2.09 (m, 2H), 1.93 – 1.85 (m, 4H), 1.79 (d, *J* = 4.3 Hz, 6H), 1.74 – 1.34 (m, 28H), 1.05 – 0.98 (m, 6H), 0.96 – 0.90 (m, 6H).

^{13}C NMR (151 MHz, Acetone-*d6*/CS_2) δ 150.54, 150.20, 145.40, 144.88, 144.53, 140.19, 140.11, 138.77, 138.75, 138.45, 137.01, 136.95, 136.61, 135.70, 135.57, 135.29, 135.09, 133.26, 133.14, 132.81, 132.31, 132.15, 130.59, 130.52, 130.49, 126.79, 126.70, 125.87, 122.52, 118.04, 116.56, 116.40, 90.81, 38.17, 36.51, 36.46, 34.24, 33.92, 33.00, 32.92, 32.75, 30.70, 28.10, 23.88, 23.83, 23.79, 21.81, 17.60, 16.63, 14.94, 14.85.

^{11}B NMR (128 MHz, $CDCl_3$/CS_2) δ 40.9.

HRMS (MALDI-TOF) m/z: $[M]^+$ Calculated for $C_{72}H_{84}BN_3$ 1001.6764; Found 1001.6737 (-2.7 ppm).

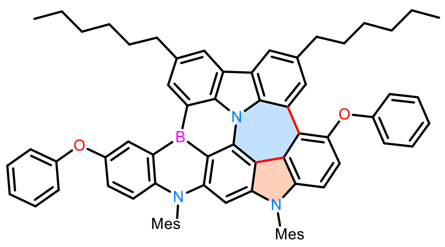

8k

8k (23.1 mg, 45% yield, brown solid) was synthesized from **7k** (52.0 mg, 0.05 mmol, 1.0 equiv.) following **General Procedure A**. Purification by column chromatography on silica gel (hexane/DCM = 12/1 – 3/1).

^{1}H NMR (400 MHz, $CDCl_3$) δ 8.52 (d, *J* = 2.9 Hz, 1H), 8.37 (d, *J* = 1.8 Hz, 1H), 8.32 (s, 1H), 7.87 (d, *J* = 1.6 Hz, 1H), 7.48 (d, *J* = 1.8 Hz, 1H), 7.42 – 7.37 (m, 2H), 7.25 – 7.21 (m, 2H), 7.20 – 7.11 (m, 4H), 7.00 – 6.91 (m, 6H), 6.76 (d, *J* = 8.5 Hz, 1H), 6.57 (d, *J* = 9.2 Hz, 1H), 6.45 (d, *J* = 8.5 Hz, 1H), 5.23 (s, 1H), 2.80 (t, *J* = 7.8 Hz, 2H), 2.51 (t, *J* = 7.7 Hz, 2H), 2.34 (s, 3H), 2.32 (s, 3H), 1.82 – 1.80 (m, 12H), 1.74 – 1.69 (dd, *J* = 16.1, 8.7 Hz, 4H), 1.53 – 1.28 (m, 12H), 0.92 – 0.86 (m, 6H).

^{13}C NMR (151 MHz, $CDCl_3$) δ 158.49, 158.13, 150.50, 146.60, 146.25, 145.29, 142.49, 142.26, 140.29, 140.06, 138.49, 138.41, 138.23, 137.55, 137.21, 136.92, 136.67, 132.54, 130.92, 130.13, 129.90, 129.66, 129.20, 128.67, 126.04, 125.76, 124.97, 123.82, 123.62, 123.30, 123.09, 122.74, 121.90, 121.86, 120.29, 119.06, 116.65, 116.35, 110.15, 106.40, 86.93, 36.60, 35.86, 32.64, 32.01, 31.92, 31.28, 29.29, 29.13, 22.85, 22.74, 21.18, 21.12, 17.51, 17.35, 14.31.

^{11}B NMR (128 MHz, $CDCl_3$/CS_2) δ 38.0.

HRMS (MALDI-TOF) m/z: $[M]^+$ Calculated for $C_{72}H_{68}BN_3O_2$ 1017.5411; Found 1017.5391 (-1.9 ppm).

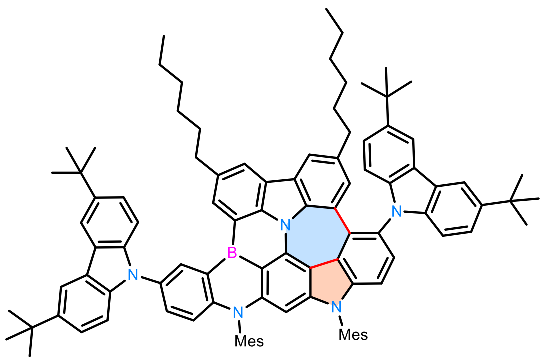

8l

8l (26.7 mg, 38% yield, brown solid) was synthesized from **7l** (71.0 mg, 0.05 mmol, 1.0 equiv.) following **General Procedure A**. Purification by column chromatography on silica gel (hexane/DCM = 12/1 − 5/1).

^{1}H NMR (400 MHz, $CDCl_3$) δ 9.15 (d, *J* = 2.6 Hz, 1H), 8.49 (s, 1H), 8.41 – 8.28 (m, 1H), 8.19 (d, *J* = 1.9 Hz, 2H), 8.11 (d, *J* = 1.3 Hz, 1H), 7.82 (s, 1H), 7.58 – 7.55 (m, 3H), 7.51 – 7.49 (m, 2H), 7.38 – 7.34 (m, 3H), 7.22 (s, 1H), 7.16 – 7.08 (m, 2H), 7.05 (s, 2H), 6.98 (s, 2H), 6.77 (d, *J* = 9.0 Hz, 1H), 6.62 (d, *J* = 8.4 Hz, 1H), 6.41 (d, *J* = 1.8 Hz, 1H), 5.37 (s, 1H), 2.77 (t, *J* = 7.7 Hz, 2H), 2.37 (s, s, 6H), 1.92 (s, 6H), 1.90 (s, 6H), 1.68 – 1.64 (m, 4H), 1.49 (s, 18H), 1.44 (s, 18H), 1.23 – 1.19 (m, 6H), 0.93 – 0.77 (m, 14H).

^{13}C NMR (151 MHz, Acetone-*d6*/CS_2) δ 147.04, 146.04, 145.21, 143.14, 142.92, 142.80, 140.87, 140.75, 140.29, 140.11, 139.51, 139.39, 139.26, 137.56, 137.48, 137.26, 137.17, 133.37, 133.26, 131.29, 131.14, 131.03, 130.62, 130.24, 129.86, 129.65, 129.38, 128.91, 128.69, 128.53, 127.65, 127.32, 126.08, 126.02, 124.44, 124.30, 124.28, 124.20, 124.16, 124.00, 123.38, 123.05, 121.21, 117.26, 117.08, 116.83, 110.81, 110.71, 110.00, 107.44, 88.12, 37.20, 35.93, 35.10, 35.07, 35.05, 33.14, 32.76, 32.60, 32.56, 32.49, 32.32, 31.15, 30.71, 30.67, 23.70, 23.68, 21.70, 18.02, 17.90, 14.95, 14.86.

^{11}B NMR (128 MHz, $CDCl_3$/CS_2) δ 39.6.

HRMS (MALDI-TOF) m/z: $[M]^+$ Calculated for $C_{100}H_{106}BN_5$ 1387.8551; Found 1387.8547 (-0.3 ppm).

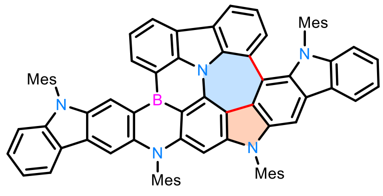

8m

8m (34.4 mg, 63% yield, brown-red solid) was synthesized from **7l** (55.1 mg, 0.05 mmol, 1.0 equiv.) following **General Procedure A**. Purification by column chromatography on silica gel (hexane/DCM = 12/1 − 5/1).

^{1}H NMR (600 MHz, DMSO-*d6*) δ 8.41 (d, *J* = 7.5 Hz, 1H), 8.37 (s, 1H), 8.31 (d, *J* = 7.5 Hz, 1H), 8.13 (d, *J* = 7.8 Hz, 1H), 7.92 (dd, *J* = 10.0, 8.0 Hz, 2H), 7.50 (t, *J* = 7.4 Hz, 1H), 7.48 – 7.43 (m, 2H), 7.31 – 7.23 (m, 4H), 7.19 (d, *J* = 7.6 Hz, 3H), 7.17 – 7.12 (m, 2H), 7.09 (s, 2H), 7.01 (d, *J* = 8.2 Hz, 1H), 6.89 (d, *J* = 8.0 Hz, 1H), 6.78 (d, *J* = 8.3 Hz, 1H), 6.66 (s, 1H), 6.47 (s, 1H), 5.32 (s, 1H), 2.47 – 2.50 (m, 6H), 2.36 – 2.38 (m, 6H), 1.86 (s, 6H), 1.83 (s, 6H), 1.78 (s, 6H), 1.56 (s, 3H),

1.50 (s, 3H).

^{13}C NMR (151 MHz, DMSO-*d6*) δ 146.13, 145.42, 142.83, 141.99, 141.43, 140.63, 140.28, 140.17, 140.06, 138.50, 138.47, 138.22, 138.16, 136.91, 136.64, 136.45, 136.07, 135.44, 134.30, 132.27, 132.03, 131.17, 130.83, 130.54, 130.26, 129.63, 129.29, 127.07, 125.55, 123.40, 123.06, 122.75, 122.33, 121.87, 121.44, 121.23, 120.90, 120.43, 119.58, 119.33, 117.70, 117.19, 114.05, 113.42, 109.76, 109.30, 107.47, 104.91, 104.32, 100.74, 98.26, 87.22, 67.01, 31.15, 29.82, 20.87, 20.66, 18.58, 18.31, 17.24, 17.08, 16.83.

^{11}B NMR (128 MHz, $CDCl_3/CS_2$) δ 39.7.

HRMS (MALDI-TOF) m/z: $[M]^+$ Calculated for $C_{78}H_{62}BN_5$ 1079.5105; Found 1079.5124 (1.8 ppm).

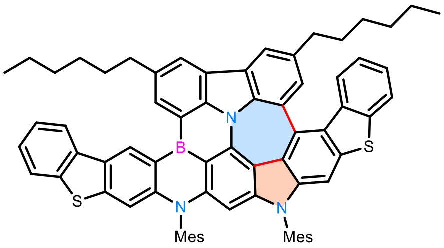

8n

8n (26.1 mg, 49% yield, brown-red solid) was synthesized from **7n** (53.0 mg, 0.05 mmol, 1.0 equiv.) following **General Procedure A**. Purification by column chromatography on silica gel (hexane/DCM = 12/1 – 5/1).

^{1}H NMR (400 MHz, $CDCl_3$) δ 9.73 (s, 1H), 8.81 (s, 1H), 8.32 (dd, *J* = 12.5, 8.0 Hz, 2H), 8.05 (d, *J* = 1.5 Hz, 1H), 7.86 – 7.76 (m, 2H), 7.71 (d, *J* = 7.8 Hz, 1H), 7.65 (d, *J* = 1.7 Hz, 1H), 7.53 (t, *J* = 7.4 Hz, 1H), 7.42 (t, *J* = 7.4 Hz, 1H), 7.28 (d, *J* = 7.4 Hz, 1H), 7.16 (q, *J* = 7.8 Hz, 1H), 7.10 – 6.91 (m, 5H), 6.85 (s, 1H), 5.25 (s, 1H), 3.08 (t, *J* = 7.7 Hz, 2H), 2.56 (t, *J* = 6.5 Hz, 2H), 2.37 (d, *J* = 2.2 Hz, 6H), 1.98 (d, *J* = 16.6 Hz, 5H), 1.84 (s, 3H), 1.79 (s, 3H), 1.64 (s, 6H), 1.53 – 1.27 (m, 11H), 0.95 (t, *J* = 7.0 Hz, 3H), 0.92 – 0.86 (m, 3H).

^{13}C NMR (101 MHz, $CDCl_3$) δ 146.54, 146.13, 145.46, 144.63, 142.75, 141.82, 141.15, 141.05, 139.95, 138.99, 138.72, 138.64, 138.45, 138.17, 137.54, 136.98, 136.90, 136.77, 136.68, 136.21, 136.04, 133.04, 131.87, 131.19, 130.83, 130.59, 130.13, 129.45, 129.37, 129.29, 128.53, 127.19, 126.54, 126.10, 125.53, 125.36, 124.81, 123.48, 122.82, 122.69, 122.23, 120.94, 120.88, 114.24, 107.61, 106.64, 102.32, 87.70, 36.76, 35.57, 32.38, 32.08, 31.97, 31.20, 29.86, 29.26, 22.83, 22.78, 21.23, 21.19, 17.67, 17.47, 17.38, 17.32, 14.38, 14.30.

^{11}B NMR (128 MHz, $CDCl_3/CS_2$) δ 41.7.

HRMS (MALDI-TOF) m/z: $[M]^+$ Calculated for $C_{72}H_{64}BN_3S_2$ 1045.4641; Found 1045.4704 (6.0 ppm).

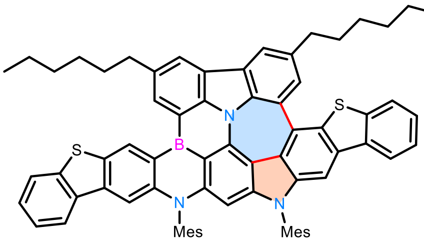

8o

8o (481 mg, 56% yield, brown-red solid) was synthesized from **7o** (870 mg, 0.82 mmol, 1.0 equiv.) following modified **General Procedure A** (20 mL DCM and 500 µl HOTf for 30 min). Purification by column chromatography on silica gel (hexane/DCM = 12/1 − 6/1).

^{1}H NMR (400 MHz, $CDCl_3$) δ 9.44 (s, 1H), 8.75 (s, 1H), 8.32 (d, *J* = 1.6 Hz, 1H), 8.04 (d, *J* = 1.4 Hz, 1H), 7.94 (d, *J* = 7.7 Hz, 1H), 7.90 – 7.80 (m, 3H), 7.65 (d, *J* = 1.7 Hz, 1H), 7.43 (dt, *J* = 13.7, 7.5 Hz, 2H), 7.35 (t, *J* = 7.6 Hz, 2H), 7.31 (s, 1H), 7.29 (d, *J* = 4.0 Hz, 1H), 7.06 (s, 2H), 7.01 (s, 2H), 5.25 (s, 1H), 3.05 (t, *J* = 7.8 Hz, 2H), 2.82 (t, *J* = 7.6 Hz, 2H), 2.41 (d, *J* = 4.6 Hz, 6H), 1.94 – 1.79 (m, 16H), 1.54 – 1.26 (m, 12H), 0.97 – 0.92 (m, 6H).

^{13}C NMR (101 MHz, $CDCl_3$) δ 147.00, 146.26, 143.84, 142.63, 141.54, 140.95, 140.10, 139.58, 139.24, 138.66, 138.56, 138.54, 138.35, 137.40, 136.93, 136.88, 136.59, 135.57, 135.34, 132.87, 131.23, 130.97, 130.31, 129.64, 129.36, 129.13, 128.71, 128.30, 127.78, 127.66, 127.61, 126.83, 126.62, 126.16, 124.25, 124.06, 123.29, 123.17, 122.20, 122.12, 120.92, 120.35, 106.85, 105.59, 101.87, 87.12, 36.59, 35.59, 32.58, 32.02, 31.99, 31.33, 29.33, 29.21, 22.86, 22.84, 21.27, 21.23, 17.56, 17.45, 14.34.

^{11}B NMR (128 MHz, $CDCl_3/CS_2$) δ 39.1.

HRMS (MALDI-TOF) m/z: $[M]^+$ Calculated for $C_{72}H_{64}BN_3S_2$ 1045.4641; Found 1045.4558 (-7.9 ppm).

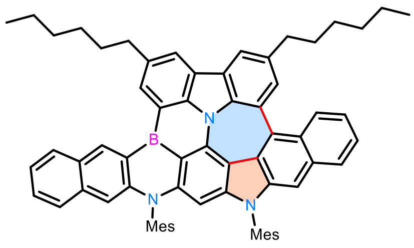

8p

8p (33.3 mg, 71% yield, brown solid) was synthesized from **7p** (48.0 mg, 0.05 mmol, 1.0 equiv.) following **General Procedure A**. Purification by column chromatography on silica gel (hexane/DCM = 12/1 − 6/1). Single crystal was grown by slow diffusion of methanol to the toluene solution of **8p**.

^{1}H NMR (600 MHz, $CDCl_3$) δ 9.52 (s, 1H), 8.80 (s, 1H), 8.60 (d, *J* = 8.9 Hz, 1H), 8.10 (d, *J* = 8.3 Hz, 1H), 8.02 (s, 1H), 7.59 (dd, *J* = 14.8, 7.9 Hz, 3H), 7.46 – 7.33 (m, 2H), 7.31 – 7.22 (m, 3H), 7.14 – 6.92 (m, 4H), 6.91 – 6.75 (m, 2H), 5.17 (s, 1H), 3.04 (t, *J* = 7.9 Hz, 2H), 2.70 (t, *J* = 7.8 Hz, 2H), 2.38 (d, *J* = 11.1 Hz, 6H), 2.00 – 1.67 (m, 14H), 1.53 – 1.28 (m, 12H), 0.93 (dt, *J* = 12.8, 6.7 Hz, 6H).

^{13}C NMR (151 MHz, $CDCl_3$) δ 148.24, 148.01, 144.03, 143.49, 143.03, 140.85, 139.68, 138.56, 138.24, 138.19, 137.39, 137.22, 136.98, 136.93, 136.87, 136.06, 135.02, 133.10, 131.98, 131.02, 130.21, 129.69, 129.29, 129.10, 128.88, 128.74, 128.61, 128.47, 127.94, 127.25, 127.12, 127.00, 125.89, 125.20, 124.70, 123.60, 123.29, 122.82, 122.41, 119.85, 113.69, 109.92, 105.62, 104.86, 87.34, 36.76, 35.70, 32.55, 32.05, 32.01, 31.69, 29.85, 29.32, 29.17, 22.87, 22.84, 22.82, 21.24, 21.18, 17.56, 17.39, 14.33, 14.31.

^{11}B NMR (128 MHz, $CDCl_3$) δ 41.4.

HRMS (MALDI-TOF) m/z: $[M]^+$ Calculated for $C_{68}H_{64}BN_3$ 933.5199; Found 933.5218 (2.0 ppm).

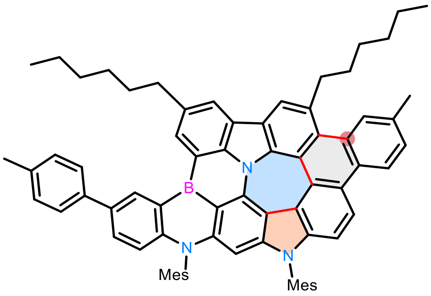

10a

10a (42.0 mg, 83% yield, brown-red solid) was synthesized from **9a** (50.3 mg, 0.05 mmol, 1.0 equiv.) following **General Procedure B**. Purification by column chromatography on silica gel (hexane/DCM = 12/1 − 4/1).

^{1}H NMR (400 MHz, $CDCl_3$) δ 9.30 – 9.18 (m, 1H), 8.80 (s, 1H), 8.31 (d, J = 8.4 Hz, 1H), 8.19 (d, J = 8.4 Hz, 1H), 8.08 (d, J = 16.2 Hz, 3H), 7.66 (dd, J = 16.5, 8.7 Hz, 3H), 7.39 (d, J = 8.3 Hz, 1H), 7.33 (d, J = 7.8 Hz, 2H), 7.02 (s, 2H), 6.97 (s, 2H), 6.86 (d, J = 8.7 Hz, 1H), 6.65 (d, J = 8.9 Hz, 1H), 5.39 (s, 1H), 3.33 (t, J = 8.0 Hz, 2H), 3.02 (t, J = 7.7 Hz, 2H), 2.55 (s, 3H), 2.44 (s, 3H), 2.36 (d, J = 9.5 Hz, 6H), 1.98 – 1.89 (m, 4H), 1.85 (s, 6H), 1.81 (s, 6H), 1.49 – 1.28 (m, 12H), 0.91 (dt, J = 13.6, 6.9 Hz, 6H).

^{13}C NMR (101 MHz, $CDCl_3$) δ 138.82, 138.48, 138.19, 137.19, 136.98, 136.25, 136.14, 133.84, 132.94, 131.47, 131.13, 130.83, 130.15, 129.92, 129.77, 129.35, 129.23, 128.58, 128.11, 126.77, 126.20, 125.84, 124.30, 123.28, 122.69, 122.30, 121.91, 120.79, 115.59, 114.98, 111.01, 37.13, 36.69, 32.57, 32.41, 32.11, 31.97, 29.86, 29.66, 29.29, 22.83, 21.80, 21.27, 21.24, 21.17, 17.48, 17.44, 14.35, 14.27.

^{11}B NMR (128 MHz, $CDCl_3/CS_2$) δ 40.0.

HRMS (MALDI-TOF) m/z: $[M]^+$ Calculated for $C_{74}H_{70}BN_3$ 1011.5669; Found 1011.5767 (9.6 ppm).

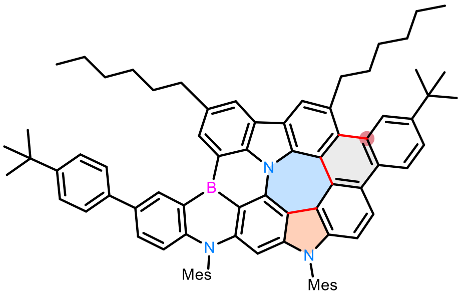

10b

10b (44.4 mg, 81% yield, brown-red solid) was synthesized from **9b** (56.0 mg, 0.05 mmol, 1.0 equiv.) following **General Procedure B**. Purification by column chromatography on silica gel (hexane/DCM = 12/1 − 4/1).

^{1}H NMR (400 MHz, $CDCl_3$) δ 9.27 (s, 1H), 8.82 (s, 1H), 8.44 – 8.02 (m, 5H), 7.87 – 7.48 (m, 6H), 7.13 – 6.91 (m, 4H), 6.86 (d, J = 8.5 Hz, 1H), 6.65 (d, J = 8.8 Hz, 1H), 5.40 (s, 1H), 3.39 (s, 2H), 3.04 (s, 2H), 2.36 (d, J = 9.7 Hz, 6H), 2.02 – 1.71 (m, 16H), 1.56 – 1.23 (m, 30H), 0.99 – 0.82 (m, 6H).

^{13}C NMR (101 MHz, $CDCl_3$) δ 149.48, 146.03, 145.82, 145.66, 143.81, 141.68, 140.37, 139.04, 138.77, 138.47, 138.26, 138.18, 137.19, 136.99, 136.86, 136.71, 136.00, 133.85, 133.02, 132.08, 131.91, 131.13, 130.92, 130.15, 129.22, 129.02, 128.56, 126.60, 126.53, 126.30, 125.99,

125.81, 124.56, 124.32, 123.32, 122.41, 122.16, 121.90, 120.79, 115.57, 110.98, 87.41, 37.56, 36.69, 35.02, 34.67, 32.49, 32.44, 32.11, 32.01, 31.63, 31.58, 29.81, 29.30, 22.84, 22.79, 21.24, 21.17, 17.46, 14.37, 14.27.

^{11}B NMR (128 MHz, $CDCl_3/CS_2$) δ 40.7.

HRMS (MALDI-TOF) m/z: $[M]^+$ Calculated for $C_{80}H_{82}BN_3$ 1095.6609; Found 1095.6589 (-1.8 ppm).

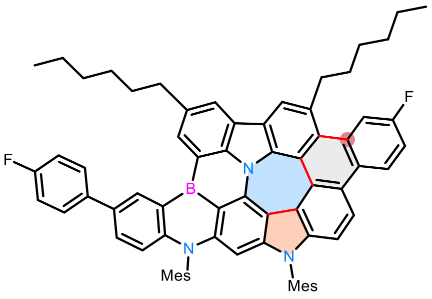

10c

10c (39.0 mg, 76% yield, brown-red solid) was synthesized from **9c** (52.5 mg, 0.05 mmol, 1.0 equiv.) following **General Procedure B**. Purification by column chromatography on silica gel (hexane/DCM = 12/1 – 4/1).

^{1}H NMR (400 MHz, $CDCl_3$) δ 9.19 (s, 1H), 8.77 (s, 1H), 8.38 (dd, J = 9.2, 5.9 Hz, 1H), 8.21 – 8.03 (m, 3H), 7.94 (d, J = 11.8 Hz, 1H), 7.72 (dd, J = 8.4, 5.2 Hz, 2H), 7.60 (d, J = 8.9 Hz, 1H), 7.30 (s, 1H), 7.20 (t, J = 8.5 Hz, 2H), 7.03 (s, 2H), 6.98 (s, 2H), 6.88 (d, J = 8.7 Hz, 1H), 6.67 (d, J = 8.9 Hz, 1H), 5.41 (s, 1H), 3.33 (t, J = 7.7 Hz, 2H), 3.02 (t, J = 7.7 Hz, 2H), 2.38 (s, 3H), 2.36 (s, 3H), 1.83 (d, J = 17.8 Hz, 16H), 1.48 – 1.24 (m, 12H), 0.90 (dt, J = 13.9, 6.9 Hz, 6H).

^{13}C NMR (101 MHz, $CDCl_3$) δ 163.42, 160.99, 160.53, 158.13, 145.88, 145.76, 143.87, 141.62, 140.39, 138.87, 138.60, 138.31, 137.79, 137.78, 137.13, 136.91, 136.80, 136.74, 136.19, 133.94, 133.13, 131.40, 131.00, 130.81, 130.64, 130.63, 130.21, 129.28, 128.34, 128.27, 127.41, 126.35, 126.16, 124.90, 124.75, 123.97, 123.13, 122.47, 122.11, 122.01, 120.83, 115.96, 115.73, 115.06, 114.97, 114.84, 114.75, 111.23, 107.12, 87.53, 36.96, 36.62, 32.35, 32.28, 32.07, 31.82, 29.52, 29.23, 22.80, 22.77, 21.24, 21.17, 17.47, 17.41, 14.32, 14.22.

^{11}B NMR (128 MHz, $CDCl_3/CS_2$) δ 40.2.

^{19}F NMR (377 MHz, $CDCl_3$) δ -116.98, -117.63.

HRMS (MALDI-TOF) m/z: $[M]^+$ Calculated for $C_{72}H_{64}BF_2N_3$ 1019.5167; Found 1019.5207 (3.9 ppm).

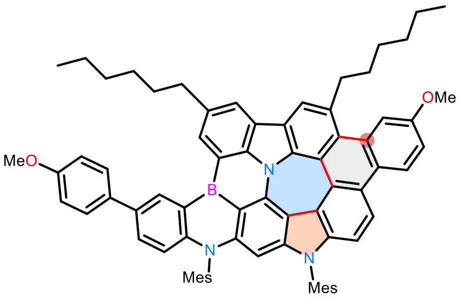

10d

10d (41.8 mg, 80% yield, brown-red solid) was synthesized from **9d** (53.4 mg, 0.05 mmol, 1.0 equiv.) following **General Procedure B**. Purification by column chromatography on silica gel

(hexane/DCM = 12/1 − 1/1). Single crystal was grown by slow diffusion of methanol to the toluene solution of **10d**.

^{1}H NMR (400 MHz, $CDCl_3$) δ 9.27 – 9.13 (m, 1H), 8.80 (s, 1H), 8.34 (d, *J* = 9.0 Hz, 1H), 8.10 (t, *J* = 11.3 Hz, 3H), 7.80 – 7.65 (m, 3H), 7.66 – 7.55 (m, 1H), 7.21 (dd, *J* = 9.0, 2.5 Hz, 1H), 7.07 (s, 1H), 7.05 (d, *J* = 1.9 Hz, 1H), 7.02 (s, 2H), 6.97 (s, 2H), 6.86 (d, *J* = 8.8 Hz, 1H), 6.65 (d, *J* = 8.9 Hz, 1H), 5.40 (s, 1H), 3.98 (s, 3H), 3.90 (s, 3H), 3.39 (t, *J* = 7.9 Hz, 2H), 3.03 (t, *J* = 7.9 Hz, 2H), 2.3 – 2.35 (m, 6H), 1.91 (q, *J* = 7.7 Hz, 4H), 1.85 – 1.81 (m, 12H), 1.54 – 1.26 (m, 12H), 0.94 –0.86 (m, 6H).

^{13}C NMR (101 MHz, $CDCl_3$) δ 158.75, 155.85, 145.78, 141.63, 139.02, 138.48, 138.20, 137.91, 137.19, 136.98, 136.70, 135.98, 134.34, 133.52, 133.09, 131.25, 131.13, 130.70, 130.58, 130.16, 129.23, 127.89, 125.97, 125.57, 125.12, 124.69, 124.40, 123.23, 122.23, 122.02, 121.92, 120.54, 115.85, 115.62, 114.49, 112.45, 111.13, 107.08, 55.61, 55.52, 37.30, 36.69, 32.56, 32.39, 32.09, 32.01, 29.77, 29.27, 22.83, 22.78, 21.24, 21.17, 17.48, 17.43, 14.35, 14.25.

^{11}B NMR (128 MHz, $CDCl_3/CS_2$) δ 42.0.

HRMS (MALDI-TOF) m/z: $[M]^+$ Calculated for $C_{74}H_{70}BN_3O_2$ 1043.5567; Found 1043.5660 (8.9 ppm).

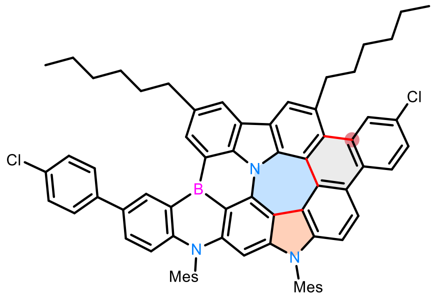

10e

10e (38.0 mg, 72% yield, brown-red solid) was synthesized from **9e** (53.5 mg, 0.05 mmol, 1.0 equiv.) following **General Procedure B**. Purification by column chromatography on silica gel (hexane/DCM = 12/1 − 5/1).

^{1}H NMR (400 MHz, $CDCl_3$) δ 9.20 (s, 1H), 8.76 (s, 1H), 8.33 (d, *J* = 8.9 Hz, 1H), 8.23 (s, 1H), 8.15 (d, *J* = 8.8 Hz, 1H), 8.08 (d, *J* = 17.6 Hz, 2H), 7.69 (d, *J* = 8.0 Hz, 2H), 7.61 (d, *J* = 9.0 Hz, 1H), 7.51 – 7.46 (m, 3H), 7.03 (s, 2H), 6.98 (s, 2H), 6.88 (d, *J* = 8.8 Hz, 1H), 6.67 (d, *J* = 8.9 Hz, 1H), 5.41 (s, 1H), 3.30 (t, *J* = 8.0 Hz, 2H), 3.02 (t, *J* = 7.7 Hz, 2H), 2.38-2.36 (m, 6H), 1.99 – 1.79 (m, 16H), 1.50 – 1.28 (m, 12H), 0.93 – 0.84 (m, 6H).

^{13}C NMR (101 MHz, $CDCl_3$) δ 145.97, 145.89, 143.89, 141.64, 140.38, 140.11, 138.88, 138.65, 138.57, 138.36, 137.11, 136.87, 136.67, 136.18, 134.01, 133.17, 132.61, 131.10, 130.93, 130.74, 130.48, 130.37, 130.22, 129.48, 129.29, 129.22, 129.16, 129.03, 128.06, 126.77, 126.59, 126.40, 124.66, 124.40, 123.72, 123.14, 122.71, 122.19, 122.05, 120.92, 115.79, 111.25, 107.16, 87.62, 36.98, 36.64, 32.45, 32.32, 32.09, 31.89, 29.51, 29.25, 22.82, 22.80, 21.24, 21.17, 17.47, 17.40, 14.34, 14.26.

^{11}B NMR (128 MHz, $CDCl_3/CS_2$) δ 40.1.

HRMS (MALDI-TOF) m/z: $[M]^+$ Calculated for $C_{74}H_{64}BCl_2N_3$ 1051.4576; Found 1051.4664 (8.4ppm).

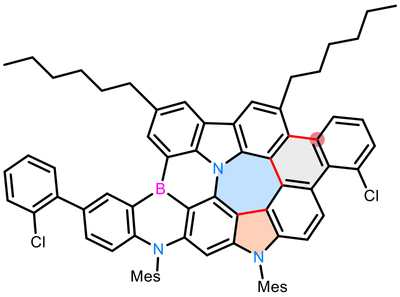

10f

10f (34.3 mg, 65% yield, brown-red solid) was synthesized from **9f** (54.0 mg, 0.05 mmol, 1.0 equiv.) following **General Procedure B**. Purification by column chromatography on silica gel (hexane/DCM = 12/1 − 5/1).

^{1}H NMR (400 MHz, $CDCl_3$) δ 9.20 – 9.05 (m, 2H), 8.73 (s, 1H), 8.17 – 8.00 (m, 3H), 7.61 (d, *J* = 8.0 Hz, 1H), 7.58 – 7.51 (m, 3H), 7.41 – 7.30 (m, 3H), 7.05 – 6.95 (m, 4H), 6.83 (d, *J* = 9.0 Hz, 1H), 6.64 (d, *J* = 9.0 Hz, 1H), 5.40 (s, 1H), 3.29 (t, *J* = 8.0 Hz, 2H), 2.98 (t, *J* = 7.9 Hz, 2H), 2.37 – 2.35 (m, 6H), 1.86 – 1.78 (m, 16H), 1.41 – 1.27 (m, 12H), 0.93 – 0.85 (m, 6H).

^{13}C NMR (101 MHz, $CDCl_3$) δ 145.90, 145.83, 143.97, 141.55, 140.75, 140.44, 138.55, 138.27, 138.15, 137.10, 136.96, 136.71, 136.69, 136.00, 133.36, 132.75, 132.59, 131.81, 131.60, 131.02, 130.53, 130.49, 130.46, 130.41, 130.19, 129.26, 129.12, 128.25, 128.10, 128.04, 127.16, 126.54, 126.24, 124.59, 123.95, 122.96, 122.36, 122.22, 122.04, 121.33, 114.91, 108.61, 107.22, 87.54, 36.95, 36.63, 32.66, 32.41, 32.00, 31.82, 31.58, 29.85, 29.52, 29.20, 22.78, 22.74, 21.23, 21.17, 17.53, 17.49, 14.33, 14.28, 14.21.

^{11}B NMR (128 MHz, $CDCl_3/CS_2$) δ 39.4.

HRMS (MALDI-TOF) m/z: $[M]^+$ Calculated for $C_{74}H_{64}BCl_2N_3$ 1051.4576; Found 1051.4582 (0.6 ppm).

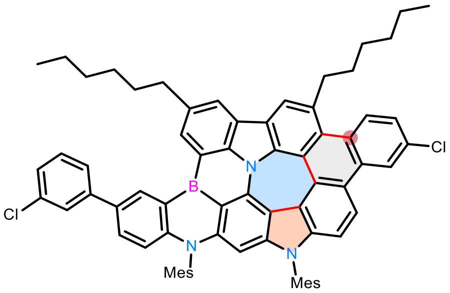

10g

10g (39.9 mg, 76% yield, brown-red solid) was synthesized from **9g** (53.0 mg, 0.05 mmol, 1.0 equiv.) following **General Procedure B**. Purification by column chromatography on silica gel (hexane/DCM = 12/1 − 5/1).

^{1}H NMR (400 MHz, $CDCl_3$) δ 9.23 (s, 1H), 8.78 (s, 1H), 8.35 (s, 1H), 8.19 – 8.04 (m, 3H), 7.77 (s, 1H), 7.64 (t, *J* = 9.7 Hz, 2H), 7.43 (t, *J* = 7.9 Hz, 2H), 7.35 (d, *J* = 8.2 Hz, 2H), 7.04 (s, 2H), 6.99 (s, 2H), 6.89 (d, *J* = 8.9 Hz, 1H), 6.67 (d, *J* = 8.9 Hz, 1H), 5.41 (s, 1H), 3.29 (t, *J* = 7.9 Hz, 2H), 3.01 (t, *J* = 7.9 Hz, 2H), 2.38 – 2.36 (m, 6H), 1.93 – 1.78 (m, 16H), 1.49 – 1.26 (m, 12H), 0.96 – 0.83 (m, 6H).

^{13}C NMR (101 MHz, $CDCl_3$) δ 146.16, 145.92, 143.92, 143.47, 141.67, 140.33, 138.89, 138.77, 138.68, 138.39, 137.08, 136.95, 136.86, 136.65, 136.01, 134.95, 134.22, 133.99, 133.11, 132.57, 132.25, 131.28, 130.90, 130.85, 130.75, 130.23, 129.31, 127.67, 127.04, 127.01, 126.84,

126.57, 126.19, 124.87, 124.11, 123.78, 123.28, 123.21, 122.55, 122.32, 122.17, 122.07, 121.04, 120.00, 115.80, 115.06, 111.18, 107.19, 87.66, 37.07, 36.70, 32.43, 32.40, 32.02, 31.83, 29.56, 29.27, 22.83, 22.77, 21.24, 21.17, 17.45, 17.40, 14.32, 14.23.

^{11}B NMR (128 MHz, $CDCl_3/CS_2$) δ 41.3.

HRMS (MALDI-TOF) m/z: $[M]^+$ Calculated for $C_{74}H_{64}BCl_2N_3$ 1051.4576; Found 1051.4655 (7.5 ppm).

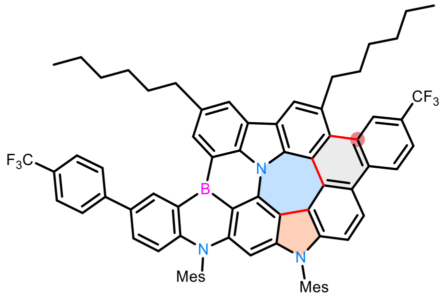

10h

10h (36.3 mg, 64% yield, orange solid) was synthesized from **9h** (56.7 mg, 0.05 mmol, 1.0 equiv.) following **General Procedure B**. Purification by column chromatography on silica gel (hexane/DCM = 12/1 − 5/1).

^{1}H NMR (400 MHz, $CDCl_3$) δ 9.27 (s, 1H), 8.78 (s, 1H), 8.64 – 8.43 (m, 2H), 8.24 (d, J = 8.9 Hz, 1H), 8.12 (d, J = 10.9 Hz, 2H), 7.98 – 7.62 (m, 6H), 7.17 – 6.96 (m, 4H), 6.92 (d, J = 8.7 Hz, 1H), 6.70 (d, J = 8.9 Hz, 1H), 5.43 (s, 1H), 3.29 (t, J = 7.7 Hz, 2H), 3.03 (t, J = 7.5 Hz, 2H), 2.38 – 2.36 (m, 6H), 2.05 – 1.74 (m, 16H), 1.53 – 1.18 (m, 12H), 1.05 – 0.80 (m, 6H).

^{13}C NMR (101 MHz, $CDCl_3$) δ 146.38, 145.96, 145.12, 143.97, 141.68, 140.38, 139.06, 138.89, 138.77, 138.47, 137.06, 137.01, 136.83, 136.58, 136.21, 134.48, 133.21, 133.09, 130.94, 130.83, 130.26, 129.33, 128.85, 127.49, 126.95, 126.58, 126.25, 126.01, 125.97, 125.45, 125.14, 124.62, 123.44, 123.36, 123.14, 123.08, 122.58, 122.34, 122.11, 121.42, 115.93, 111.34, 107.24, 87.79, 37.20, 36.63, 32.53, 32.31, 32.10, 31.92, 29.57, 29.25, 22.81, 22.77, 21.25, 21.18, 17.45, 17.40, 14.27, 14.23.

^{11}B NMR (128 MHz, $CDCl_3/CS_2$) δ 40.3.

^{19}F NMR (377 MHz, $CDCl_3$) δ -61.70, -62.18.

HRMS (MALDI-TOF) m/z: $[M]^+$ Calculated for $C_{74}H_{64}BF_6N_3$ 1119.5104; Found 1119.5209 (9.4 ppm).

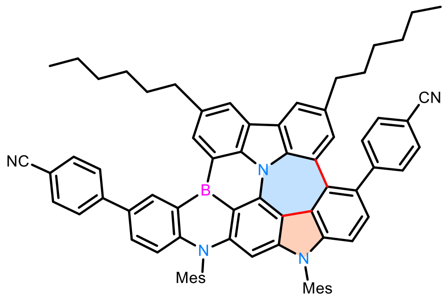

10i

10i (20.2 mg, 39% yield, orange solid) was synthesized from **9i** (53.0 mg, 0.05 mmol, 1.0 equiv.) following **General Procedure B**. Purification by column chromatography on silica gel (hexane/EA = 12/1 − 8/1). Single crystal was grown by slow diffusion of methanol to the toluene

solution of **10i**.

^{1}H NMR (400 MHz, $CDCl_3$) δ 9.24 (d, *J* = 2.5 Hz, 1H), 8.67 (d, *J* = 1.6 Hz, 1H), 7.97 (d, *J* = 1.5 Hz, 1H), 7.85 (d, *J* = 8.4 Hz, 2H), 7.78 (d, *J* = 8.3 Hz, 2H), 7.70 – 7.60 (m, 3H), 7.56 (d, *J* = 8.3 Hz, 2H), 7.44 (d, *J* = 1.7 Hz, 1H), 7.05 – 6.92 (m, 5H), 6.69 (d, *J* = 8.9 Hz, 1H), 6.65 (d, *J* = 1.7 Hz, 1H), 6.58 (d, *J* = 8.2 Hz, 1H), 5.32 (s, 1H), 2.98 (t, *J* = 7.6 Hz, 2H), 2.35 (d, *J* = 5.0 Hz, 5H), 2.19 (t, *J* = 7.5 Hz, 2H), 1.82 (s, 12H), 1.47 (q, *J* = 6.9 Hz, 2H), 1.44 – 1.09 (m, 14H), 0.92 (dt, *J* = 8.6, 6.9 Hz, 6H).

^{13}C NMR (101 MHz, $CDCl_3$) δ 150.29, 146.55, 146.36, 146.07, 145.53, 142.56, 141.36, 140.23, 138.76, 138.56, 137.73, 137.07, 136.91, 136.71, 136.42, 134.63, 134.41, 132.90, 132.66, 131.24, 130.65, 130.26, 129.30, 128.94, 127.18, 126.37, 123.45, 122.42, 120.16, 119.42, 116.06, 109.98, 109.59, 109.21, 106.60, 103.68, 102.51, 99.28, 87.77, 36.60, 35.32, 32.22, 32.05, 31.86, 30.84, 29.18, 29.14, 22.85, 22.79, 21.22, 21.17, 17.52, 17.34, 14.31.

^{11}B NMR (128 MHz, $CDCl_3/CS_2$) δ 38.5.

HRMS (MALDI-TOF) m/z: $[M]^+$ Calculated for $C_{74}H_{66}BN_5$ 1035.5417; Found 1035.5555 (13.3 ppm).

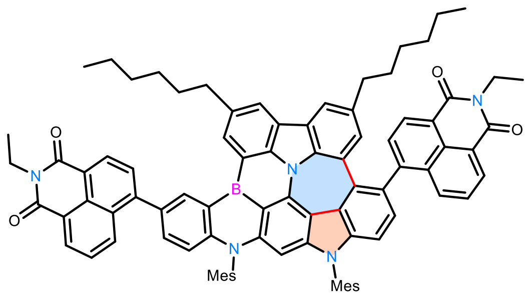

10j

10j (30.6 mg, 48% yield, orange solid) was synthesized from **9j** (65.0 mg, 0.05 mmol, 1.0 equiv.) following **General Procedure B**. Purification by column chromatography on silica gel (hexane/EA = 12/1 − 3/1).

^{1}H NMR (600 MHz, Acetone-*d6*/CS_2) δ 9.14 (s, 1H), 8.70 – 8.59 (m, 3H), 8.59 – 8.45 (m, 4H), 7.89 – 7.83 (m, 4H), 7.69 – 7.62 (m, 2H), 7.27 (s, 1H), 7.12 (s, 2H), 7.08 (d, *J* = 8.2 Hz, 1H), 7.04 (s, 2H), 6.73 (d, *J* = 8.7 Hz, 1H), 6.66 – 6.60 (m, 2H), 5.53 (s, 1H), 4.24 – 4.15 (m, 4H), 2.81 (t, *J* = 7.7 Hz, 2H), 2.42 (d, *J* = 3.1 Hz, 3H), 1.96 – 1.94 (m, 6H), 1.87 (t, *J* = 7.9 Hz, 2H), 1.70 (q, *J* = 7.6 Hz, 2H), 1.43 – 1.26 (m, 14H), 1.15 (q, *J* = 7.3 Hz, 2H), 0.95 – 0.86 (m, 6H), 0.54 (q, *J* = 7.7 Hz, 3H). Due to its poor solubility, we could not get clear ^{13}C NMR spectrum.

^{11}B NMR (128 MHz, $CDCl_3/CS_2$) δ 39.3.

HRMS (MALDI-TOF) m/z: $[M]^+$ Calculated for $C_{88}H_{78}BN_5O_4$ 1279.6155; Found 1279.6165 (0.8 ppm).

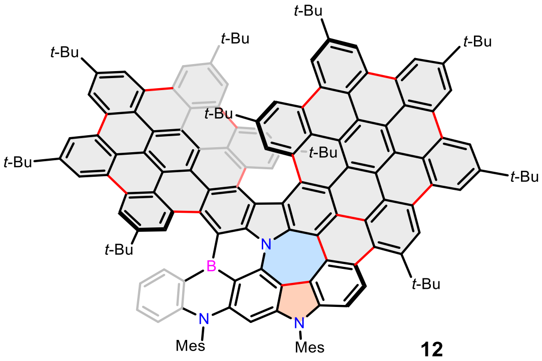

12

12 (18.3 mg, 17% yield, dark-brown solid) was synthesized from **11** (108 mg, 0.05 mmol, 1.0 equiv.) following **General Procedure C** (18.0 equiv. DDQ, reaction time: 12 h). Purification by column chromatography on silica gel (hexane/DCM = 12/1 − 2/3).

^{1}H NMR (600 MHz, Acetone-*d6*/CS_2) δ 9.94 (s, 1H), 9.59 (s, 1H), 9.47 (s, 1H), 9.44 – 9.40 (m, 4H), 9.36 – 9.35 (m, 2H), 9.33 – 9.29 (m, 4H), 9.19 (s, 1H), 9.09 (s, 1H), 9.04 (s, 1H), 8.75 (s, 1H), 8.67 (s, 1H), 8.61 (d, J = 8.3 Hz, 1H), 7.68 (d, *J* = 8.3 Hz, 1H), 7.55 – 7.52 (m, 2H), 7.47 – 7.40 (m, 2H), 7.14 (s, 1H), 7.06 (s, 1H), 6.70 (t, *J* = 7.2 Hz, 1H), 5.78 (d, *J* = 15.0 Hz, 1H), 5.40 (d, *J* = 1.6 Hz, 1H), 4.67 (d, *J* = 15.1 Hz, 1H), 2.90 (s, 3H), 2.88 (s, 3H), 2.72 (s, 3H), 2.46 (s, 3H), 2.00 (s, 9H), 1.96 (s, 9H), 1.93 (m, 18H), 1.80 (m, 18H), 1.57 (s, 3H), 1.36 (s, 9H), 0.15 (s, 9H), 0.11 (s, 9H).

^{13}C NMR (151 MHz, Acetone-*d6*/CS_2) δ 149.36, 149.31, 149.21, 147.89, 147.65, 147.52, 145.01, 144.70, 144.64, 143.89, 143.64, 143.24, 141.59, 139.02, 138.37, 138.19, 138.10, 138.05, 136.81, 136.51, 135.25, 134.27, 133.09, 133.07, 133.06, 131.67, 131.38, 131.35, 131.31, 131.26, 131.25, 131.20, 131.16, 131.13, 131.08, 130.94, 130.89, 130.87, 130.86, 130.73, 130.14, 130.13, 130.10, 129.86, 129.81, 129.65, 129.60, 129.34, 127.14, 127.12, 126.88, 126.37, 126.30, 126.18, 125.62, 125.16, 124.75, 124.72, 124.42, 124.36, 124.24, 124.18, 124.01, 123.92, 123.51, 123.47, 123.42, 123.29, 122.33, 121.91, 121.79, 121.71, 121.58, 121.25, 121.19, 121.18, 121.14, 121.05, 121.01, 120.99, 120.94, 120.89, 120.79, 120.77, 120.66, 120.29, 120.27, 119.84, 119.80, 119.76, 119.73, 119.69, 119.61, 119.53, 119.33, 118.69, 112.09, 111.60, 104.91, 39.52, 36.22, 36.13, 36.08, 35.97, 35.95, 35.82, 35.47, 34.42, 34.40, 32.89, 32.70, 32.58, 32.54, 32.42, 32.03, 31.94, 30.82, 30.80, 30.69, 27.99, 26.45, 23.83, 23.19, 21.69, 21.19, 20.85, 20.23, 18.00, 15.08.

^{11}B NMR (128 MHz, $CDCl_3$/CS_2) δ 41.2

HRMS (MALDI-TOF) m/z: $[M]^+$ Calculated for $C_{160}H_{138}BN_3$ 2113.1024; Found 2113.1744 (34.0 ppm).

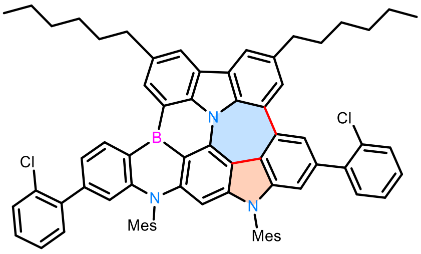

14

14 (34.7 mg, 67% yield, orange solid) was synthesized from **13** (54.0 mg, 0.05 mmol, 1.0 equiv.) following **General Procedure A**. Purification by column chromatography on silica gel (hexane/DCM = 15/1 − 4/1). Single crystals of **13** and **14** were grown by slow diffusion of methanol

to the toluene solution of **13** and **14**, respectively.

^{1}H NMR (600 MHz, $CDCl_3$) δ 9.05 (d, J = 7.8 Hz, 1H), 8.72 (s, 1H), 7.98 (s, 1H), 7.58 (d, J = 8.1 Hz, 2H), 7.46 – 7.40 (m, 3H), 7.35 – 7.27 (m, 4H), 7.26 (s, 3H), 6.94 (s, 2H), 6.90 (s, 2H), 6.73 (s, 1H), 6.54 (s, 1H), 5.24 (s, 1H), 2.99 (t, J = 7.8 Hz, 2H), 2.68 (t, J = 7.8 Hz, 2H), 2.31 (s, 3H), 2.28 (s, 3H), 1.91 – 1.62 (m, 16H), 1.50 (t, J = 7.4 Hz, 2H), 1.46 – 1.32 (m, 10H), 0.94 – 0.89 (m, 6H).

^{13}C NMR (151 MHz, $CDCl_3$) δ 145.20, 142.76, 142.11, 141.35, 140.94, 140.65, 140.39, 138.37, 138.15, 137.37, 137.14, 136.85, 136.68, 136.52, 135.44, 133.30, 132.79, 131.49, 130.95, 130.85, 130.13, 130.04, 129.16, 128.60, 128.47, 126.95, 126.86, 124.39, 124.19, 123.19, 122.20, 121.23, 120.45, 117.43, 116.21, 115.28, 110.65, 105.89, 87.56, 36.72, 35.82, 32.68, 32.06, 31.97, 31.68, 29.31, 29.19, 22.86, 22.83, 21.15, 21.09, 17.53, 17.40, 14.33, 14.29.

^{11}B NMR (128 MHz, $CDCl_3/CS_2$) δ 41.2.

HRMS (MALDI-TOF) m/z: $[M]^+$ Calculated for $C_{72}H_{66}BCl_2N_3$ 1053.4733; Found 1053.4690 (-4.1 ppm).

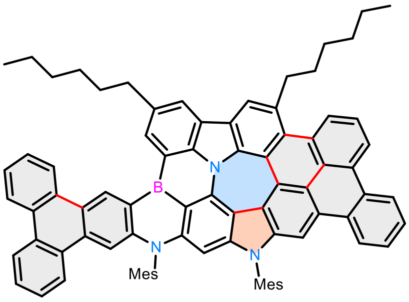

16a

16a (38.7 mg, 68% yield, brown-red solid) was synthesized from **15a** (58.0 mg, 0.05 mmol, 1.0 equiv.) following **General Procedure C** (6.0 equiv. DDQ, reaction time: 30 min). Purification by column chromatography on silica gel (hexane/DCM = 12/1 − 2/1). Single crystal was grown by slow diffusion of methanol to the toluene solution of **16a**.

^{1}H NMR (600 MHz, Acetone-*d6*/CS_2) δ 10.32 (s, 1H), 9.07 (s, 1H), 8.91 (d, J = 8.1 Hz, 1H), 8.75 (d, J = 7.6 Hz, 1H), 8.70 (d, J = 8.0 Hz, 1H), 8.65 (d, J = 8.1 Hz, 2H), 8.47 (d, J = 7.9 Hz, 1H), 8.39 (d, J = 8.0 Hz, 1H), 8.28 (s, 1H), 8.24 (s, 1H), 7.98 (d, J = 8.1 Hz, 1H), 7.94 (d, J = 2.0 Hz, 1H), 7.83 (t, J = 7.7 Hz, 1H), 7.79 – 7.74 (m, 1H), 7.70 (s, 1H), 7.68 – 7.57 (m, 4H), 7.50 (t, J = 7.5 Hz, 1H), 7.24 (s, 2H), 7.15 (s, 2H), 5.49 (s, 1H), 3.50 – 3.42 (m, 2H), 3.24 (t, J = 7.8 Hz, 2H), 2.53 (s, 3H), 2.51 (s, 3H), 2.14 (p, J = 7.7 Hz, 2H), 2.01 (s, 6H), 1.97 (s, 6H), 1.71 (q, J = 7.8 Hz, 2H), 1.62 – 1.58 (m, 2H), 1.54 – 1.49 (m, 2H), 1.43 – 1.37 (m, 4H), 1.02 (t, J = 7.3 Hz, 3H), 0.94 – 0.89 (m, 6H).

^{13}C NMR (151 MHz, Acetone-*d6*/CS_2) δ 147.60, 145.86, 145.77, 142.94, 140.79, 139.87, 139.64, 139.30, 139.26, 137.79, 137.68, 137.53, 137.46, 137.33, 134.02, 133.94, 132.41, 132.24, 131.56, 131.52, 131.25, 131.08, 130.61, 130.44, 130.26, 129.64, 129.23, 128.76, 128.69, 128.46, 128.45, 128.25, 128.04, 128.02, 127.83, 127.20, 127.04, 126.10, 125.96, 125.50, 124.52, 124.41, 124.34, 124.30, 124.22, 124.21, 123.86, 123.80, 123.72, 123.57, 123.38, 123.18, 121.66, 120.51, 115.49, 108.07, 107.75, 104.72, 88.34, 54.89, 42.19, 37.84, 37.55, 37.00, 35.57, 35.49, 34.67, 33.28, 33.23, 33.06, 32.91, 32.76, 32.56, 30.71, 30.54, 28.59, 27.89, 26.29, 23.87, 23.79, 23.70, 23.21, 21.85, 21.80, 21.48, 21.35, 20.07, 19.39, 18.04, 17.94, 15.02, 14.90, 12.17.

^{11}B NMR (128 MHz, $CDCl_3/CS_2$) δ 40.4.

HRMS (MALDI-TOF) m/z: $[M]^+$ Calculated for $C_{84}H_{70}BN_3$ 1131.5670; Found 1131.5750 (7.1 ppm).

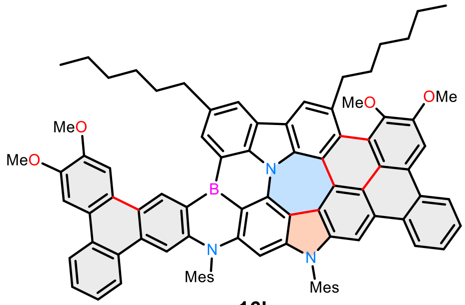

16b

16b (33.6 mg, 54% yield, brown-red solid) was synthesized from **15b** (64.0 mg, 0.05 mmol, 1.0 equiv.) following **General Procedure C** (6.0 equiv. DDQ, reaction time: 30 min). Purification by column chromatography on silica gel (hexane/DCM = 12/1 − 1/2).

^{1}**H NMR** (600 MHz, Acetone-*d6*/CS_2) δ 10.19 (d, *J* = 2.6 Hz, 1H), 9.08 (d, *J* = 2.3 Hz, 1H), 8.63 – 8.60 (m, 1H), 8.54 – 8.51 (m, 1H), 8.36 – 8.28 (m, 3H), 8.25 (dd, *J* = 3.3, 1.6 Hz, 1H), 8.02 (d, *J* = 3.3 Hz, 2H), 7.95 (d, *J* = 8.2 Hz, 1H), 7.80 (d, *J* = 3.3 Hz, 1H), 7.68 (d, *J* = 2.7 Hz, 1H), 7.64 – 7.54 (m, 3H), 7.42 (ddd, *J* = 8.2, 6.9, 1.3 Hz, 1H), 7.23 (s, 2H), 7.17 (s, 1H), 7.10 (s, 1H), 5.49 (s, 1H), 4.30 (s, 3H), 4.21 (s, 3H), 4.09 (s, 3H), 3.50 (s, 3H), 3.28 (ddd, *J* = 15.0, 9.9, 5.2 Hz, 2H), 3.18 (t, *J* = 7.9 Hz, 2H), 3.10 – 2.99 (m, 2H), 2.53 (s, 3H), 2.50 (s, 3H), 2.07 (s, 3H), 2.00 (s, 6H), 1.84 (s, 3H), 1.67 – 1.59 (m, 4H), 1.52 – 1.46 (m, 4H), 1.01 (t, *J* = 7.0 Hz, 3H), 0.94 – 0.87 (m, 6H), 0.80 – 0.75 (m, 3H).

^{13}**C NMR** (151 MHz, Acetone-*d6*/CS_2) δ 151.51, 150.94, 150.07, 147.67, 147.18, 145.91, 145.07, 143.02, 140.73, 140.67, 139.53, 139.18, 138.89, 137.83, 137.72, 137.54, 137.51, 137.43, 134.16, 133.40, 131.67, 131.61, 131.51, 131.31, 131.11, 130.99, 130.43, 130.37, 129.53, 128.43, 128.06, 127.83, 127.66, 127.50, 127.32, 126.68, 125.52, 125.48, 125.23, 124.48, 124.47, 124.42, 124.19, 123.97, 123.83, 123.71, 123.34, 123.29, 122.96, 122.19, 120.76, 120.71, 108.01, 107.47, 107.12, 106.40, 105.32, 104.58, 88.11, 61.20, 56.96, 56.23, 55.88, 54.88, 42.19, 38.09, 37.00, 36.88, 35.57, 35.50, 33.91, 33.69, 33.01, 32.73, 32.56, 30.71, 28.59, 27.90, 26.29, 23.89, 23.70, 23.65, 23.21, 21.86, 21.79, 21.48, 21.35, 20.07, 19.39, 18.16, 17.95, 17.93, 17.91, 15.06, 14.98, 14.84, 12.17.

^{11}**B NMR** (128 MHz, $CDCl_3/CS_2$) δ 38.3.

HRMS (MALDI-TOF) m/z: $[M]^+$ Calculated for $C_{88}H_{78}BN_3O_4$ 1251.6093; Found 1251.6043 (-4.0 ppm).

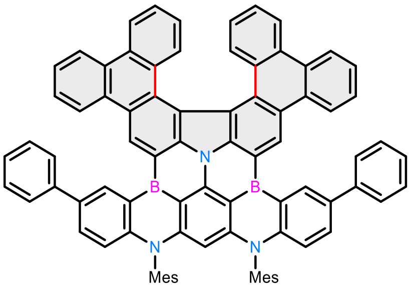

18

18 was isolated from the BDA reaction of **17** towards **19** (7.5 equiv. DDQ, quenched the

reaction after 3 h). The yield of **18** was not determined.

^{1}H NMR (600 MHz, Acetone-*d6*/CS_2) δ 10.66 (s, 2H), 9.78 (s, 2H), 9.09 (d, *J* = 8.1 Hz, 2H), 8.75 (d, *J* = 8.1 Hz, 2H), 8.53 (d, *J* = 8.1 Hz, 2H), 8.07 (d, *J* = 7.9 Hz, 2H), 7.94 (d, *J* = 7.6 Hz, 4H), 7.89 – 7.82 (m, 4H), 7.77 (t, *J* = 7.5 Hz, 2H), 7.61 (q, *J* = 7.1 Hz, 4H), 7.43 (t, *J* = 7.4 Hz, 2H), 7.36 (t, *J* = 7.5 Hz, 2H), 7.08 (d, *J* = 10.5 Hz, 4H), 7.02 (d, *J* = 8.7 Hz, 2H), 6.47 (t, *J* = 7.4 Hz, 2H), 5.53 (s, 1H), 2.54 (s, 6H), 1.91 (s, 6H), 1.87 (s, 6H).

^{13}C NMR (151 MHz, Acetone-*d6*/CS_2) δ 150.29, 145.95, 144.23, 142.36, 141.82, 138.89, 136.99, 136.91, 136.64, 134.72, 134.05, 132.01, 131.44, 131.14, 130.69, 130.55, 130.55, 130.46, 130.23, 129.81, 129.56, 129.52, 128.94, 128.47, 127.65, 127.60, 127.55, 127.45, 125.43, 124.75, 124.24, 122.71, 121.28, 117.17, 91.95, 21.95, 17.72, 17.69.

HRMS (MALDI-TOF) m/z: $[M]^+$ Calculated for $C_{84}H_{57}B_2N_3$ 1129.4757; Found 1129.4800 (3.8 ppm).

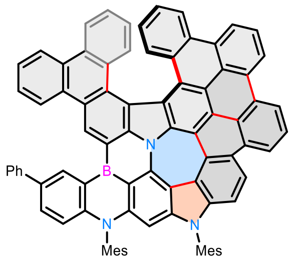

19

19 (13.8 mg, 25% yield, dark-brown solid) was synthesized from **17** (57.3 mg, 0.05 mmol, 1.0 equiv.) following **General Procedure C** (7.5 equiv. DDQ, reaction time: 12 h). Purification by column chromatography on silica gel (hexane/DCM = 12/1 − 1/1).

^{1}H NMR (600 MHz, Acetone-*d6*/CS_2) δ 10.02 (s, 1H), 9.53 (s, 1H), 9.04 – 8.99 (m, 2H), 8.91 (br, 1H), 8.86 – 8.52 (m, 4H), 8.42 (br, 1H), 8.09 (s, 2H), 7.96 (br, 1H), 7.81 (br, 2H), 7.72 – 7.65 (m, 2H), 7.53 – 7.29 (m, 3H), 7.25 – 6.94 (m, 7H), 6.72 (d, *J* = 7.4 Hz, 2H), 6.54 (d, *J* = 9.0 Hz, 1H), 5.88 (s, 1H), 5.45 (s, 2H), 2.49 (d, *J* = 10.3 Hz, 6H).

^{13}C NMR (151 MHz, Acetone-*d6*/CS_2) δ 145.99, 145.79, 144.40, 142.84, 142.72, 141.25, 140.57, 139.60, 139.54, 139.19, 139.08, 137.84, 137.63, 137.25, 137.22, 136.72, 136.51, 136.10, 132.37, 132.11, 131.79, 131.65, 131.54, 131.33, 131.12, 130.89, 130.78, 130.71, 130.60, 130.53, 130.46, 130.43, 130.27, 130.20, 129.95, 129.69, 129.40, 128.83, 128.75, 128.60, 128.48, 128.04, 127.68, 127.50, 127.40, 127.19, 127.13, 126.73, 126.60, 126.34, 126.18, 126.14, 125.98, 125.59, 125.49, 124.90, 124.85, 124.43, 124.01, 123.84, 123.62, 123.43, 123.17, 122.89, 122.63, 122.46, 122.19, 122.13, 122.08, 121.85, 120.17, 115.88, 112.33, 108.08, 88.50, 21.87, 18.13, 18.05, 17.91.

^{11}B NMR (128 MHz, $CDCl_3$/CS_2) δ 40.7.

HRMS (MALDI-TOF) m/z: $[M]^+$ Calculated for $C_{84}H_{52}BN_3$ 1113.4262; Found 1113.4312 (4.5 ppm).

4. Synthetic derivatization and product elaboration

4.1 Sequential nitrogen-deletion, BDA reaction and amination reaction

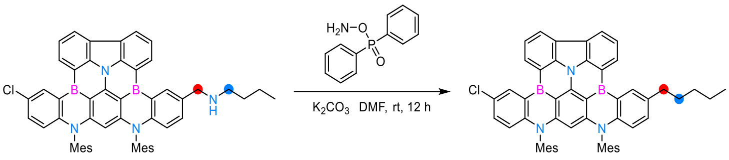

20 **21**

Compound **20** (60.0 mg, 0.075 mmol, 1.0 equiv.) and K_2CO_3 (30 mg, 0.225 mmol, 3.0 equiv.) were added to a 10 mL vial. DMF (1.0 mL) and *o*-diphenylphosphinylhydroxylamine (38 mg, 0.165 mmol, 2.2 equiv.) were finally added under N_2. The reaction mixture stirred at rt for 12 hours. The reaction was quenched by water and was extracted with DCM. After the removal of solvent, the residue was further purified by preparative thin-layer chromatography using Hex/DCM (v/v = 5:1) as the eluent to give compound **21** (15.0 mg, 26% yield) as a yellow solid.

^{1}H NMR (400 MHz, $CDCl_3$) δ 9.15 (d, *J* = 7.6 Hz, 1H), 9.10 (d, *J* = 2.6 Hz, 1H), 9.07 (d, *J* = 7.6 Hz, 1H), 9.00 (s, 1H), 8.53 (dd, *J* = 7.4, 2.4 Hz, 2H), 7.84 (td, *J* = 7.6, 2.3 Hz, 2H), 7.40 (dd, *J* = 9.2, 2.5 Hz, 1H), 7.35 – 7.30 (m, 1H), 6.94 (s, 4H), 6.77 (dd, *J* = 8.9, 2.9 Hz, 2H), 5.32 (s, 1H), 2.85 (t, *J* = 7.9 Hz, 2H), 2.43 (s, 6H), 1.82 – 1.77 (m, 2H), 1.72 (s, 12H), 1.50 – 1.40 (m, 4H), 0.95 (t, *J* = 6.7 Hz, 3H).

^{13}C NMR (101 MHz, $CDCl_3$) δ 150.21, 149.84, 144.44, 144.19, 143.02, 141.81, 141.68, 138.40, 138.15, 136.71, 136.63, 136.37, 136.05, 134.89, 134.63, 133.17, 132.67, 132.46, 131.53, 129.90, 129.76, 126.21, 125.95, 125.45, 125.36, 124.13, 123.95, 123.35, 117.43, 115.92, 111.19, 90.24, 35.78, 31.93, 31.71, 29.86, 22.82, 21.41, 17.25, 17.19, 14.29, 6.96, 6.55.

HRMS (MALDI-TOF) m/z: $[M]^+$ Calculated for $C_{53}H_{46}B_2ClN_3$ 781.3578; Found 781.3635 (7.3 ppm).

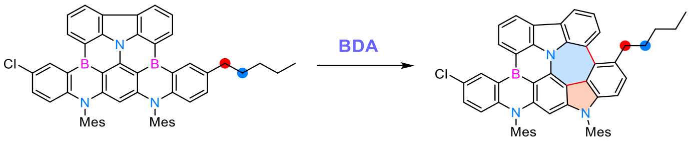

21 **22**

Compound **22** (26.7 mg, 67% yield, orange solid) was synthesized from **21** (40 mg, 0.05 mmol, 1.0 equiv.) followed **General Procedure A** for BDA reaction.

^{1}H NMR (400 MHz, $CDCl_3$) δ 8.91 (d, *J* = 2.5 Hz, 1H), 8.83 (d, *J* = 7.5 Hz, 1H), 8.21 (d, *J* = 7.6 Hz, 1H), 7.85 (d, *J* = 7.5 Hz, 1H), 7.64 (t, *J* = 7.5 Hz, 1H), 7.43 (d, *J* = 7.8 Hz, 1H), 7.31 (dd, *J* = 9.3, 2.5 Hz, 1H), 7.25 – 7.22 (m, 1H), 7.10 (d, *J* = 8.3 Hz, 1H), 6.98 (s, 2H), 6.93 (s, 2H), 6.52 (dd, *J* = 8.7, 5.6 Hz, 2H), 5.30 (s, 1H), 2.95 (t, *J* = 8.2 Hz, 2H), 2.33 (d, *J* = 9.4 Hz, 6H), 1.77 (d, *J* = 7.3 Hz, 14H), 1.40 – 1.37 (m, 4H), 0.94 – 0.90 (m, 3H).

^{13}C NMR (101 MHz, $CDCl_3$) δ 145.68, 145.34, 144.63, 142.91, 142.21, 141.73, 138.68, 138.43, 137.22, 136.78, 136.52, 134.59, 132.79, 132.52, 132.27, 131.87, 131.07, 130.61, 130.37, 130.22, 130.07, 129.16, 128.79, 128.50, 125.88, 125.27, 123.56, 123.32, 122.45, 122.20, 120.18, 116.81, 109.28, 106.76, 87.72, 35.06, 32.24, 32.08, 22.64, 21.20, 21.13, 17.51, 17.27, 14.28.

^{11}B NMR (128 MHz, $CDCl_3/CS_2$) δ 39.5.

HRMS (MALDI-TOF) m/z: $[M]^+$ Calculated for $C_{53}H_{45}BClN_3$ 769.3398; Found 769.3356 (-5.4 ppm).

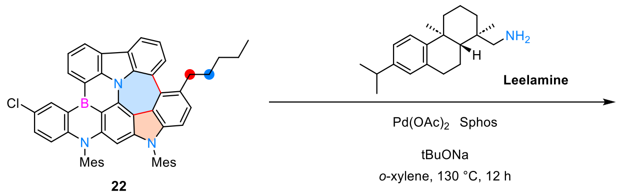

Compound **22** (20.0 mg, 0.025 mmol, 1.0 equiv.), $Pd(OAc)_2$/Sphos (5.0 mg/6.0 mg), leelamine (11.0 mg, 0.04 mmol, 1.5 equiv.), *t*-BuONa (20.0 mg, 0.21 mmol, 8.0 equiv.) and *o*-xylene (5.0 mL) were mixed and degassed by bubbling N_2 for 5 min. The reaction mixture stirred at 130 °C for 12 h. After cooling to rt, the solvent was removed in vacuo. The residue was further purified by preparative thin-layer chromatography using Hex/EA (v/v = 5:1) as the eluent to give compound **23** (22.8 mg, 86% yield) as orange-red solid.

^{1}H NMR (600 MHz, Acetone-*d6*/CS_2) δ 8.84 (dd, *J* = 7.4, 1.6 Hz, 1H), 8.24 – 8.16 (m, 2H), 7.89 (dt, *J* = 7.5, 1.5 Hz, 1H), 7.55 (t, *J* = 7.4 Hz, 1H), 7.40 (dt, *J* = 7.7, 1.4 Hz, 1H), 7.27 – 7.22 (m, 1H), 7.15 (d, *J* = 8.1 Hz, 1H), 7.10 (dd, *J* = 8.1, 1.2 Hz, 1H), 7.03 (s, 2H), 7.00 (s, 2H), 6.93 (dd, *J* = 8.1, 2.1 Hz, 1H), 6.85 (dt, *J* = 9.2, 2.4 Hz, 1H), 6.81 (d, *J* = 2.0 Hz, 1H), 6.41 (dd, *J* = 8.2, 1.0 Hz, 1H), 6.35 – 6.32 (m, 1H), 5.40 (s, 1H), 4.50 (s, 1H), 3.34 – 3.29 (m, 1H), 3.05 (dd, *J* = 12.9, 5.8 Hz, 1H), 3.00 – 2.96 (m, 2H), 2.87 (dt, *J* = 9.8, 4.8 Hz, 2H), 2.80 (p, *J* = 6.9 Hz, 1H), 2.38 (s, 3H), 2.37 (s, 3H), 1.84 (s, 6H), 1.81 (s, 6H), 1.78 – 1.69 (m, 3H), 1.63 – 1.59 (m, 1H), 1.50 – 1.40 (m, 6H), 1.35 – 1.31 (m, 3H), 1.28 (s, 3H), 1.23 (s, 3H), 1.22 (s, 3H), 1.14 (s, 3H), 0.98 (t, *J* = 6.8 Hz, 3H), 0.93 – 0.88 (m, 3H).

^{13}C NMR (151 MHz, Acetone-*d6*/CS_2) δ 148.06, 146.24, 145.85, 145.81, 144.18, 143.40, 142.76, 142.37, 139.28, 139.16, 139.10, 138.68, 137.75, 137.70, 137.48, 135.45, 133.42, 133.04, 132.73, 131.85, 130.98, 130.95, 130.85, 130.55, 130.08, 129.42, 129.13, 127.55, 126.70, 125.00, 124.53, 124.21, 123.58, 122.98, 122.46, 120.87, 120.59, 117.21, 116.62, 109.64, 106.70, 87.43, 56.72, 54.92, 45.56, 42.19, 39.42, 38.50, 38.15, 37.24, 37.01, 35.76, 35.55, 35.48, 34.66, 34.48, 33.11, 32.94, 32.55, 30.98, 30.67, 30.58, 28.58, 27.86, 26.25, 25.94, 24.67, 24.64, 23.66, 23.58, 23.36, 23.17, 21.59, 21.44, 21.30, 20.09, 20.03, 19.94, 19.86, 19.35, 17.90, 17.75, 17.74, 14.99, 14.88, 14.79, 12.11.

^{11}B NMR (128 MHz, $CDCl_3$/CS_2) δ 41.0.

HRMS (MALDI-TOF) m/z: $[M]^+$ Calculated for $C_{73}H_{75}BN_4$ 1018.6091; Found 1018.6051 (-3.9 ppm).

4.2 Synthesis of nanographenes by BDA-triggered cyclodehydrogenation

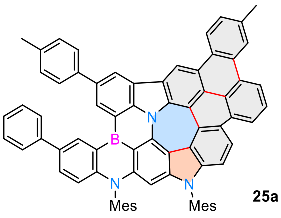

25a

25a (45.5 mg, 92% yield, orange-red solid) was synthesized from **24a** (50.5 mg, 0.05 mmol, 1.0 equiv.) following **General Procedure C** (5.0 equiv. DDQ). Purification by column chromatography on silica gel (hexane/DCM = 12/1 − 1/1). Single crystal was grown by slow diffusion of methanol to the toluene solution of **25a**.

^{1}**H NMR** (600 MHz, Acetone-*d6*/CS_2) δ 9.65 (s, 1H), 9.37 (d, *J* = 2.3 Hz, 1H), 9.34 (d, *J* = 1.8 Hz, 1H), 8.94 (d, *J* = 1.7 Hz, 1H), 8.91 (d, *J* = 8.4 Hz, 1H), 8.86 (d, *J* = 7.5 Hz, 1H), 8.81 – 8.78 (m, 1H), 8.54 (dd, *J* = 10.7, 2.2 Hz, 2H), 7.98 (t, *J* = 7.8 Hz, 1H), 7.95 – 7.92 (m, 2H), 7.81 – 7.78 (m, 2H), 7.70 (dd, *J* = 8.7, 2.3 Hz, 1H), 7.57 – 7.54 (m, 1H), 7.52 – 7.49 (m, 2H), 7.43 – 7.40 (m, 2H), 7.37 – 7.34 (m, 1H), 7.14 (s, 2H), 7.09 (s, 2H), 6.98 (d, *J* = 8.8 Hz, 1H), 6.65 (d, *J* = 8.7 Hz, 1H), 5.67 (s, 1H), 2.67 (s, 3H), 2.54 (s, 3H), 2.46 (s, 3H), 2.44 (s, 3H), 1.97 (s, 6H), 1.96 (s, 6H).

^{13}**C NMR** (151 MHz, Acetone-*d6*/CS_2) δ 146.39, 146.31, 144.33, 142.33, 142.16, 141.81, 140.64, 139.82, 139.47, 139.15, 139.12, 137.57, 137.30, 137.09, 136.33, 134.64, 133.19, 132.76, 131.67, 131.52, 131.09, 130.55, 130.53, 130.25, 130.24, 130.19, 129.84, 129.72, 128.79, 128.12, 127.53, 127.48, 127.42, 127.35, 127.09, 126.95, 125.32, 124.98, 124.83, 124.64, 124.35, 124.31, 123.22, 122.59, 122.38, 121.66, 121.29, 116.39, 115.86, 112.35, 108.41, 88.63, 22.37, 21.75, 21.74, 17.99, 17.85.

^{11}**B NMR** (128 MHz, $CDCl_3$/CS_2) δ 40.2.

HRMS (MALDI-TOF) m/z: $[M]^+$ Calculated for $C_{74}H_{52}BN_3$ 993.4260; Found 993.4425 (16.6 ppm).

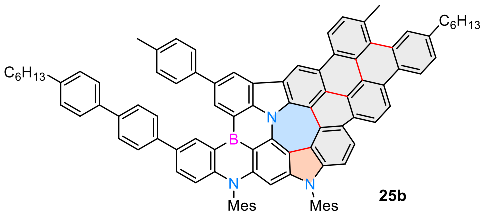

25b

25b (53.1 mg, 81% yield, orange-red solid) was synthesized from **24b** (66.7 mg, 0.05 mmol, 1.0 equiv.) following **General Procedure C** (6.0 equiv. DDQ). Purification by column chromatography on silica gel (hexane/DCM = 12/1 − 3/1).

^{1}**H NMR** (600 MHz, Acetone-*d6*/CS_2) δ 9.76 (s, 1H), 9.40 (d, *J* = 2.3 Hz, 1H), 9.34 (d, *J* = 1.7 Hz, 1H), 9.12 (t, *J* = 8.2 Hz, 3H), 8.95 (d, *J* = 1.7 Hz, 1H), 8.79 (d, *J* = 8.3 Hz, 1H), 8.68 (d, *J* = 8.9 Hz, 1H), 8.52 (d, *J* = 1.7 Hz, 1H), 7.99 (d, *J* = 8.1 Hz, 1H), 7.94 (d, *J* = 7.6 Hz, 2H), 7.85 (d, *J* = 7.9 Hz, 2H), 7.71 (d, *J* = 8.0 Hz, 3H), 7.58 (t, *J* = 7.4 Hz, 3H), 7.43 (d, *J* = 7.6 Hz, 2H), 7.28 (d, *J* = 7.7 Hz, 2H), 7.14 (s, 2H), 7.10 (s, 2H), 7.03 (d, *J* = 8.8 Hz, 1H), 6.65 (d, *J* = 8.7 Hz, 1H), 5.69 (s, 1H), 3.32 (s, 3H), 2.95 (t, *J* = 7.8 Hz, 2H), 2.71 (t, *J* = 7.7 Hz, 2H), 2.54 (s, 3H), 2.46 (d, *J* = 11.6 Hz, 6H), 1.97 (d, *J* = 4.6 Hz, 6H), 1.88 (p, *J* = 7.6 Hz, 2H), 1.72 (td, *J* = 13.4, 5.9 Hz, 2H), 1.58 – 1.35 (m, 10H), 1.00 – 0.87 (m, 6H).

^{13}**C NMR** (151 MHz, Acetone-*d6*/CS_2) δ 146.32, 146.28, 144.27, 142.34, 142.27, 142.13, 140.73, 140.63, 140.37, 139.98, 139.82, 139.41, 139.05, 138.98, 138.74, 137.56, 137.29, 137.07, 136.39, 134.38, 133.91, 132.77, 132.67, 132.34, 131.59, 131.51, 131.30, 131.07, 130.55, 130.29, 130.23, 130.15, 129.63, 129.17, 129.06, 128.47, 128.27, 128.13, 128.11, 127.80, 127.64, 127.54, 127.52, 126.48, 125.56, 125.48, 124.93, 124.79, 124.63, 123.88, 123.19, 122.56, 122.53, 122.48, 121.88, 121.70, 121.62, 116.42, 112.50, 108.42, 88.65, 37.23, 36.56, 32.84, 32.80, 32.56, 32.49, 30.71, 27.94, 27.44, 23.83, 23.80, 21.81, 18.03, 17.88, 14.99.

^{11}**B NMR** (128 MHz, $CDCl_3$/CS_2) δ 40.5.

HRMS (MALDI-TOF) m/z: $[M]^+$ Calculated for $C_{98}H_{82}BN_3$ 1311.6611; Found 1311.6586 (-1.9 ppm).

4.3 Product elaboration

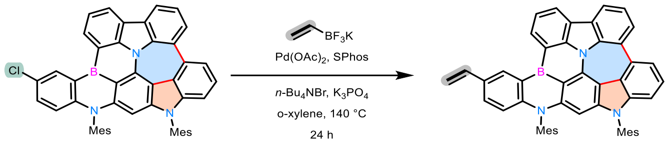

5c **26**

Compound **5c** (40.0 mg, 0.057 mmol, 1.0 equiv.) was added to a 50 mL Schlenk bottle. Potassium ethenyltrifluoroborate (25.0 mg, 0.18 mmol, 3.0 equiv.), Pd(OAc)$_2$/Sphos/*n*-Bu$_4$NBr (5 mg/6 mg/12 mg) and K_3PO_4 (120 mg, 0.57 mmol, 10.0 equiv.) were added. The mixture was dried under vacuum. After back-filled with N_2, *o*-xylene (5.0 mL) was added. The reaction mixture stirred at 140 °C for 24 hours. After cooling to rt, the mixture was passed through a pad of celite, rinsed with DCM. After removal of the solvent, the residue was purified by column chromatography on silica gel (hexane/DCM = 12/1 − 5/1) to give compound **26** (16.6 mg, 42% yield) as orange solid.

^{1}H NMR (600 MHz, C_6D_6) δ 9.29 (d, *J* = 2.2 Hz, 1H), 9.14 (d, *J* = 7.3 Hz, 1H), 8.00 – 7.92 (m, 1H), 7.72 (d, *J* = 7.7 Hz, 1H), 7.60 (dd, *J* = 7.5, 1.1 Hz, 1H), 7.49 (t, *J* = 7.5 Hz, 1H), 7.45 (dd, *J* = 8.9, 2.1 Hz, 1H), 7.36 (d, *J* = 7.9 Hz, 1H), 6.98 (t, *J* = 7.8 Hz, 1H), 6.96 – 6.85 (m, 2H), 6.72 – 6.61 (m, 5H), 6.42 (d, *J* = 7.9 Hz, 1H), 5.75 (d, *J* = 17.5 Hz, 1H), 5.68 (s, 1H), 5.16 (d, *J* = 10.9 Hz, 1H), 2.06 (s, 3H), 2.04 (s, 3H), 1.83 (s, 6H), 1.78 (s, 6H).

^{13}C NMR (151 MHz, C_6D_6) δ 142.92, 142.47, 141.96, 140.99, 138.33, 138.14, 137.55, 137.38, 137.26, 137.01, 135.19, 133.51, 131.82, 131.62, 130.58, 130.10, 129.75, 129.68, 129.40, 126.73, 125.08, 124.21, 124.00, 123.47, 122.56, 122.48, 121.16, 116.46, 115.76, 111.45, 109.55, 107.07, 87.81, 21.01, 20.97, 17.38, 17.31.

^{11}B NMR (128 MHz, $CDCl_3/CS_2$) δ 40.3.

HRMS (MALDI-TOF) m/z: [M]$^+$ Calculated for $C_{50}H_{38}BN_3$ 691.3162; Found 691.3179 (2.5 ppm).

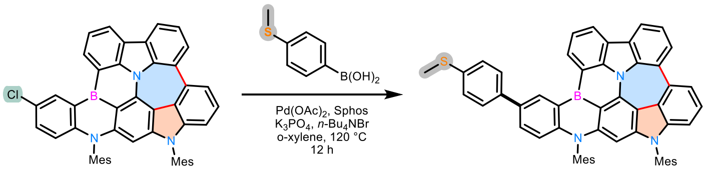

5c **27**

Compound **5c** (40.0 mg, 0.057 mmol, 1.0 equiv.) was added to a 50 mL Schlenk bottle. (4-(Methylthio)phenyl)boronic acid (30.0 mg, 0.18 mmol, 3.0 equiv.), Pd(OAc)$_2$/Sphos/*n*-Bu$_4$NBr (5 mg/6 mg/12 mg) and K_3PO_4 (120 mg, 0.57 mmol, 10.0 equiv.) were added. The mixture was dried under vacuum. After back-filled with N_2, *o*-xylene (5.0 mL) was added. The reaction mixture stirred at 120 °C for 12 hours. After cooling to rt, the mixture was passed through a pad of celite, rinsed with DCM. After removal of the solvent, the residue was purified by column chromatography on silica gel (hexane/DCM = 12/1 − 2/1) to give compound **27** (30.6 mg, 68% yield) as yellow solid.

^{1}H NMR (400 MHz, $CDCl_3$) δ 9.19 (d, *J* = 2.3 Hz, 1H), 8.93 (d, *J* = 7.5 Hz, 1H), 8.17 (d, *J* = 7.4 Hz, 1H), 7.81 (t, *J* = 7.2 Hz, 2H), 7.71 – 7.66 (m, 2H), 7.63 – 7.57 (m, 2H), 7.42 (t, *J* = 8.5 Hz, 3H), 7.15 (t, *J* = 7.7 Hz, 1H), 7.05 (t, *J* = 7.9 Hz, 1H), 7.01 (s, 2H), 6.94 (s, 2H), 6.66 (d, *J* = 8.9 Hz, 1H), 6.50 (d, *J* = 7.9 Hz, 1H), 5.32 (s, 1H), 2.56 (s, 3H), 2.35 – 2.34 (m, 6H), 1.82 (s, 6H), 1.80 (s, 6H).

^{13}C NMR (101 MHz, $CDCl_3$) δ 146.12, 145.59, 144.80, 142.13, 142.00, 141.47, 140.67, 138.62, 138.45, 138.32, 137.11, 136.84, 136.74, 136.61, 133.78, 133.03, 131.91, 131.20, 130.97, 130.82, 130.15, 129.19, 128.89, 127.64, 127.44, 126.38, 124.50, 124.35, 123.98, 123.71, 123.04, 122.25, 122.21, 120.93, 115.86, 115.75, 109.64, 106.27, 87.93, 51.06, 21.19, 21.13, 17.45, 17.36, 16.30.

^{11}B NMR (128 MHz, $CDCl_3/CS_2$) δ 41.5.

HRMS (MALDI-TOF) m/z: $[M]^+$ Calculated for $C_{55}H_{42}BN_3S$ 787.3196; Found 787.3277 (10.3 ppm).

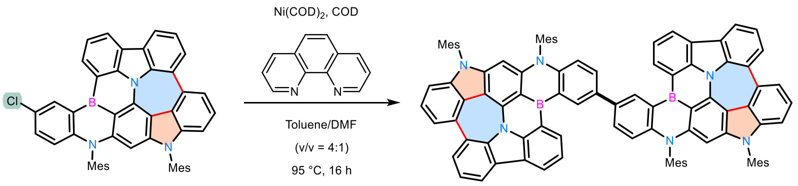

5c **28**

In a round-bottom flask, $Ni(COD)_2$ (47.0 mg, 0.17 mmol, 3.0 equiv.), COD (20 μL), 1,10-phenanthroline (30.0 mg, 0.17 mmol, 3.0 equiv.) and toluene/DMF (4.0 mL/2.0 mL) were added in the glovebox. The reaction mixture was removed from the glovebox and stirred at 80 °C for 30 min. Compound **5c** (40.0 mg, 0.057 mmol, 1.0 equiv.) was dissolved in anhydrous toluene (4.0 mL) and added to the activated Ni(COD)L solution via syringe. The reaction further stirred at 95 °C for 16 h. After cooling to rt, the mixture was passed through a pad of celite. After removal of solvent in vacuo, the residue was further purified by column chromatography on silica gel (hexane/DCM = 12/1 − 1/2) to give compound **28** (16.7 mg, 44% yield) as orange solid.

^{1}H NMR (600 MHz, Acetone-*d6* /CS_2) δ 9.33 (s, 2H), 9.09 (d, J = 7.4 Hz, 2H), 8.21 (d, J = 7.4 Hz, 2H), 7.84 (dd, J = 12.7, 7.8 Hz, 4H), 7.78 (d, J = 8.9 Hz, 2H), 7.63 (t, J = 7.4 Hz, 2H), 7.43 (d, J = 7.9 Hz, 2H), 7.18 (d, J = 7.6 Hz, 2H), 7.10 (s, 4H), 7.05 (d, J = 7.8 Hz, 2H), 7.01 (s, 4H), 6.65 (d, J = 8.8 Hz, 2H), 6.43 (d, J = 7.9 Hz, 2H), 5.49 (s, 2H), 2.42 (s, 6H), 2.41 (s, 6H), 1.92 (s, 12H), 1.87 (s, 12H). Owing to its poor solubility, we could not get clear ^{13}C NMR spectrum.

^{11}B NMR (128 MHz, $CDCl_3/CS_2$) δ 38.8.

HRMS (MALDI-TOF) m/z: $[M]^+$ Calculated for $C_{96}H_{70}B_2N_6$ 1328.5869; Found 1328.5851 (-1.4 ppm).

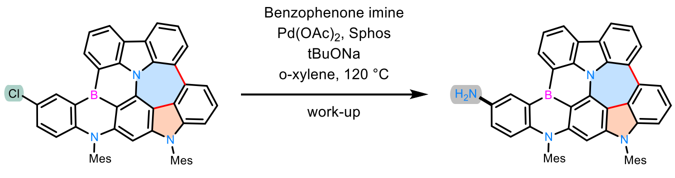

5c **29a**

Compound **5c** (40.0 mg, 0.057 mmol, 1.0 equiv.), $Pd(OAc)_2$/Sphos (5 mg/6 mg) and *t*-BuONa (33 mg, 0.34 mmol, 6.0 equiv.) were added to a 50 mL flask. After vacuumed and backfilled with nitrogen, *o*-xylene (10 mL) was added. The mixture stirred at rt for 10 min. Benzophenone imine (25 μL, ca. 3.0 equiv.) was finally added. The reaction mixture stirred at 120 °C overnight. After cooling to rt, the mixture was passed through a pad of celite, rinsed with *o*-xylene. Then 2.0 mL HCl (conc.) was added. The solution was directly adopted to the rotary evaporator with 60 °C water bath

and was dried in vacuo. The solid residue was sonicated with DCM for 30 min and filtered. The solid was further washed with DCM (10 ml×3), Et_2O (10 mL) and MeCN (10 mL). The residue was finally washed with saturated NaOH (aq.) for several times. The orange-yellow solid was finally washed with MeOH and dried in vacuo to give compound **29a** (12.5 mg, 32% yield).

^{1}H NMR (400 MHz, DMSO-*d6*) δ 8.93 (d, *J* = 7.5 Hz, 1H), 8.38 (d, *J* = 7.4 Hz, 1H), 8.21 (d, *J* = 2.6 Hz, 1H), 8.04 – 8.00 (m, 1H), 7.95 (d, *J* = 8.0 Hz, 1H), 7.63 (t, *J* = 7.4 Hz, 1H), 7.56 (d, *J* = 8.1 Hz, 1H), 7.24 (d, *J* = 7.7 Hz, 1H), 7.12 – 7.03 (m, 5H), 6.84 (dd, *J* = 9.0, 2.6 Hz, 1H), 6.41 (d, *J* = 8.0 Hz, 1H), 6.25 (d, *J* = 9.0 Hz, 1H), 5.24 (s, 1H), 5.00 (s, 2H), 2.31 (s, 3H), 2.29 (s, 3H), 1.73 (s, 6H), 1.69 (s, 6H). Owing to its poor solubility, we could not get clear ^{13}C NMR spectrum.

^{11}B NMR (128 MHz, $CDCl_3/CS_2$) δ 40.3.

HRMS (MALDI-TOF) m/z: $[M]^+$ Calculated for $C_{48}H_{37}BN_4$ 680.3114; Found 680.3226 (1.6 ppm).

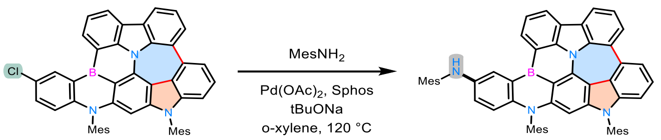

5c **29b**

Compound **5c** (40.0 mg, 0.057 mmol, 1.0 equiv.), Pd(OAc)$_2$/Sphos (5 mg/6 mg) and *t*-BuONa (33 mg, 0.34 mmol, 6.0 equiv.) were added to a 50 mL flask. After vacuumed and backfilled with nitrogen, *o*-xylene (10 mL) was added. The mixture stirred at rt for 10 min. 2,4,6-Trimethylaniline (25 μL, ca. 3.0 equiv.) was finally added. The reaction mixture stirred at 120 °C overnight. After cooling to rt, the mixture was passed through a pad of celite, rinsed with DCM. After removal of the solvent, the residue was further purified by column chromatography on silica gel (hexane/DCM = 12/1 − 1/1) to give compound **29b** (40.5 mg, 89% yield) as orange-red solid.

^{1}H NMR (400 MHz, Acetone-*d6*/DMSO-*d6*/CS_2) δ 8.53 (d, *J* = 7.5 Hz, 1H), 8.14 (d, *J* = 7.5 Hz, 1H), 8.00 (s, 1H), 7.81 (t, *J* = 8.3 Hz, 2H), 7.40 (d, *J* = 8.2 Hz, 2H), 7.14 (t, *J* = 7.6 Hz, 1H), 7.08 – 6.91 (m, 6H), 6.73 (s, 1H), 6.67 (d, *J* = 9.0 Hz, 1H), 6.36 (d, *J* = 8.0 Hz, 1H), 6.29 (d, *J* = 8.9 Hz, 1H), 5.54 (s, 1H), 5.34 (s, 1H), 2.48 (s, 6H), 2.37 – 2.35 (m, 6H), 2.23 (s, 6H), 1.83 – 1.81 (m, 9H).

^{13}C NMR (151 MHz, Acetone-*d6*/DMSO-*d6*/CS_2) δ 146.25, 144.95, 142.32, 141.96, 141.56, 141.33, 140.88, 139.08, 138.84, 138.37, 137.53, 137.30, 137.15, 136.99, 135.81, 134.42, 133.34, 131.27, 131.23, 130.57, 129.83, 129.65, 129.12, 127.87, 126.92, 125.12, 124.72, 124.56, 124.13, 122.94, 122.59, 122.49, 121.48, 120.25, 117.69, 116.49, 116.22, 109.74, 105.83, 87.23, 55.02, 49.29, 21.39, 21.26, 18.86, 17.58, 17.49.

^{11}B NMR (128 MHz, $CDCl_3/CS_2$) δ 40.7.

HRMS (MALDI-TOF) m/z: $[M]^+$ Calculated for $C_{57}H_{47}BN_4$ 798.3898; Found 798.3959 (7.6 ppm).

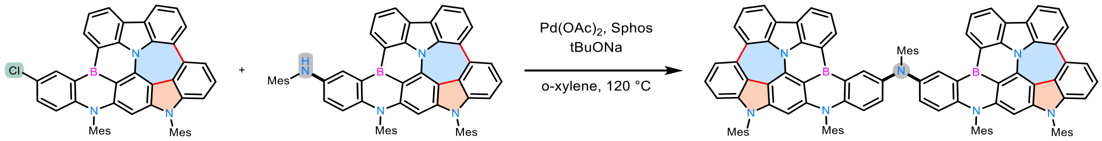

5c **29b** **30**

Compound **5c** (18.0 mg, 0.025 mmol, 1.0 equiv.), **29b** (20.0 mg, 0.025 mmol, 1.0 equiv.) $Pd(OAc)_2$/Sphos (5 mg/6 mg) and *t*-BuONa (24 mg, 10.0 equiv.) were added to a 50 mL flask. After vacuumed and backfilled with nitrogen, *o*-xylene (10 mL) was finally added. The mixture stirred at 120 °C overnight. After cooling to rt, the mixture was passed through a pad of celite, rinsed with DCM. After removal of the solvent, the residue was further purified by column chromatography on silica gel (hexane/DCM = 12/1 − 2/1) to give compound **30** (21.9 mg, 60% yield) as orange-red solid.

^{1}H NMR (400 MHz, Acetone-*d6*/CS_2) δ 8.55 (d, *J* = 2.8 Hz, 2H), 8.24 (dd, *J* = 7.6, 1.2 Hz, 2H), 8.04 (dd, *J* = 7.5, 1.0 Hz, 2H), 7.82 – 7.75 (m, 4H), 7.40 (d, *J* = 7.9 Hz, 2H), 7.30 (dd, *J* = 9.2, 2.8 Hz, 2H), 7.20 (t, *J* = 7.5 Hz, 2H), 7.15 – 7.10 (m, 4H), 7.07 – 6.99 (m, 10H), 6.47 (d, *J* = 9.3 Hz, 2H), 6.39 (d, *J* = 7.9 Hz, 2H), 5.43 (s, 2H), 2.39 – 2.36 (m, 15H), 2.20 (s, 6H), 1.91 (s, 12H), 1.86 (s, 12H).

^{13}C NMR (151 MHz, Acetone-*d6*/CS_2) δ 150.36, 146.47, 145.28, 142.69, 142.32, 141.83, 141.19, 141.16, 139.83, 139.13, 138.84, 138.33, 137.49, 137.29, 135.92, 133.42, 131.66, 131.58, 130.92, 130.54, 130.52, 130.10, 129.47, 128.16, 127.21, 125.02, 124.76, 124.64, 124.36, 123.27, 122.82, 122.65, 121.62, 116.78, 116.61, 110.07, 106.60, 87.89, 54.86, 36.07, 32.91, 28.13, 26.34, 23.78, 21.83, 21.67, 19.41, 19.19, 17.87, 17.59, 14.89.

^{11}B NMR (128 MHz, $CDCl_3/CS_2$) δ 40.3.

HRMS (MALDI-TOF) m/z: $[M]^+$ Calculated for $C_{105}H_{81}B_2N_7$ 1461.6762; Found 1461.6774 (0.8 ppm).

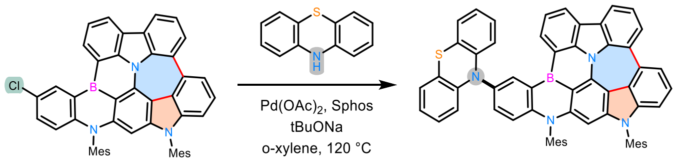

5c **31**

Compound **5c** (120.0 mg, 0.17 mmol, 1.0 equiv.), 10*H*-phenothiazine (42.0 mg, 0.21 mmol, 1.2 equiv.), $Pd(OAc)_2$/Sphos (15 mg/18 mg) and *t*-BuONa (50 mg, 0.51 mmol, 3.0 equiv.) were added to a 50 mL flask. After vacuumed and backfilled with nitrogen, *o*-xylene (20 mL) was added. The mixture stirred at 120 °C overnight. After cooling to rt, the mixture was passed through a pad of celite, rinsed with DCM. After removal of the solvent, the residue was further purified by column chromatography on silica gel (hexane/DCM = 12/1 − 3/1) to give compound **31** (133.8 mg, 91% yield) as bright orange solid.

^{1}H NMR (400 MHz, $CDCl_3$) δ 8.94 (s, 1H), 8.74 (d, *J* = 7.5 Hz, 1H), 8.14 (d, *J* = 7.5 Hz, 1H), 7.80 (dd, *J* = 12.3, 7.6 Hz, 2H), 7.52 (t, *J* = 7.5 Hz, 1H), 7.44 (d, *J* = 7.8 Hz, 1H), 7.35 (d, *J* = 8.8 Hz, 1H), 7.15 (t, *J* = 7.7 Hz, 1H), 7.09 – 6.98 (m, 5H), 6.95 (s, 2H), 6.82 (dq, *J* = 12.7, 6.6 Hz, 5H), 6.51 (d, *J* = 8.0 Hz, 1H), 6.34 (dd, *J* = 8.2, 1.4 Hz, 2H), 5.35 (s, 1H), 2.36 (s, 3H), 2.34 (s, 3H), 1.88 (s, 6H), 1.81 (s, 6H).

^{13}C NMR (151 MHz, Acetone-*d6*/CS_2) δ 146.68, 146.09, 145.29, 142.78, 142.32, 141.75, 141.20, 139.24, 138.39, 137.42, 137.14, 137.11, 135.24, 133.61, 133.14, 131.81, 131.39, 131.10, 130.11, 129.54, 128.10, 127.61, 127.53, 127.22, 125.02, 124.84, 124.59, 123.64, 123.33, 123.23, 123.06, 121.81, 120.50, 118.20, 116.98, 116.71, 110.28, 107.26, 88.62, 21.69, 17.85, 17.83.

^{11}B NMR (128 MHz, $CDCl_3/CS_2$) δ 38.1.

HRMS (MALDI-TOF) m/z: $[M]^+$ Calculated for $C_{60}H_{43}BN_4S$ 862.3306; Found 862.3329 (2.7

ppm).

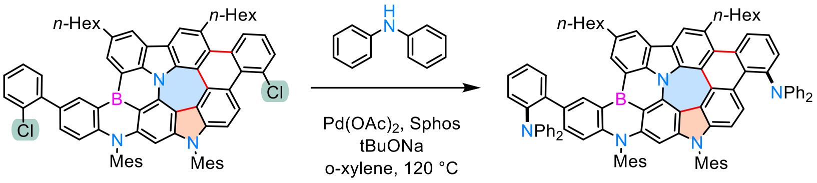

10f **32**

Compound **10f** (20.0 mg, 0.02 mmol, 1.0 equiv.), diphenylamine (16.0 mg, 0.09 mmol, 4.0 equiv.), Pd(OAc)$_2$/Sphos (10 mg/12 mg) and *t*-BuONa (20 mg, ca. 8.0 equiv.) were added to a 50 mL flask. After vacuumed and backfilled with nitrogen, *o*-xylene (10 mL) was added. The mixture stirred at 120 °C overnight. After cooling to rt, the mixture was passed through a pad of celite, rinsed with DCM. After removal of the solvent, the residue was further purified by column chromatography on silica gel (hexane/DCM = 12/1 − 1/1) to give compound **32** (22.0 mg, 88% yield) as orange-red solid.

^{1}H NMR (600 MHz, Acetone-*d6*/CS$_2$) δ 9.13 (s, 1H), 8.70 (s, 1H), 8.17 (dd, *J* = 9.0, 2.1 Hz, 1H), 8.14 (dt, *J* = 7.5, 2.0 Hz, 2H), 8.05 (d, *J* = 2.3 Hz, 1H), 7.88 (d, *J* = 8.7 Hz, 1H), 7.54 – 7.38 (m, 5H), 7.38 – 7.23 (m, 8H), 7.23 – 7.12 (m, 8H), 7.12 – 6.94 (m, 9H), 6.73 (dd, *J* = 8.7, 1.8 Hz, 1H), 6.54 (dd, *J* = 9.0, 1.8 Hz, 1H), 5.53 (d, *J* = 1.4 Hz, 1H), 3.39 (t, *J* = 8.1 Hz, 2H), 3.04 (t, *J* = 7.7 Hz, 2H), 2.39 (m, 6H), 2.01 – 1.93 (m, 4H), 1.88 (s, 6H), 1.85 (s, 6H), 1.55 (q, *J* = 7.7 Hz, 2H), 1.50 – 1.37 (m, 10H), 0.97 – 0.91 (m, 6H).

^{13}C NMR (151 MHz, Acetone-*d6*/CS$_2$) δ 158.27, 151.70, 149.29, 148.64, 148.44, 147.24, 146.54, 146.50, 144.49, 143.39, 142.38, 140.93, 139.59, 139.55, 139.26, 139.25, 137.58, 137.34, 136.63, 134.54, 133.80, 132.98, 132.77, 132.43, 131.70, 131.61, 131.57, 131.10, 130.80, 130.30, 130.21, 130.18, 128.68, 128.18, 127.48, 127.44, 126.34, 126.20, 125.39, 125.26, 124.99, 124.95, 124.88, 124.26, 124.09, 124.03, 123.69, 123.53, 123.29, 123.23, 123.02, 122.60, 122.23, 122.11, 120.76, 117.50, 117.44, 116.79, 116.37, 111.72, 107.98, 104.02, 88.25, 55.43, 37.91, 37.38, 33.19, 33.10, 32.92, 32.74, 31.28, 30.64, 30.45, 23.71, 23.67, 21.48, 17.79, 17.69, 14.81, 14.76.

^{11}B NMR (128 MHz, CDCl$_3$/CS$_2$) δ 38.8.

HRMS (MALDI-TOF) m/z: $[M]^+$ Calculated for $C_{96}H_{84}BN_5$ 1317.6829; Found 1317.6887 (4.4 ppm).

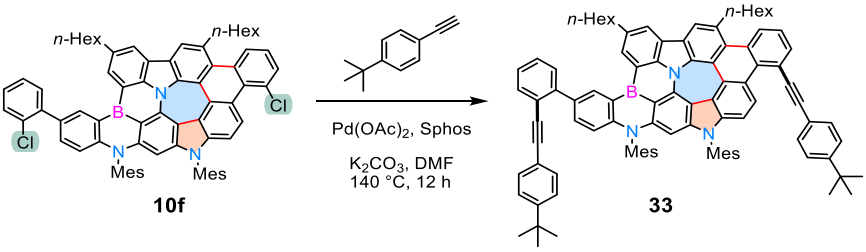

10f **33**

Compound **10f** (40.0 mg, 0.038 mmol, 1.0 equiv.), Pd(OAc)$_2$/Sphos (10 mg/12 mg) and K_2CO_3 (105 mg, ca. 20.0 equiv.) were added to a 50 mL flask. After vacuumed and backfilled with nitrogen, DMF (5.0 mL) was added. 1-(*tert*-Butyl)-4-ethynylbenzene (30 μL) was finally injected via syringe. The mixture stirred at 140 °C for 12 h. After cooling to rt, the mixture was poured into brine. The precipitate was collected by filtration and was further purified by column chromatography on silica

gel (hexane/DCM = 12/1 − 4/1) to give compound **33** (41.0 mg, 84% yield) as orange-red solid.

^{1}H NMR (600 MHz, Acetone-*d6*/CS_2) δ 9.84 (dd, *J* = 9.0, 1.9 Hz, 1H), 9.41 (t, *J* = 2.5 Hz, 1H), 8.87 – 8.81 (m, 1H), 8.25 – 8.09 (m, 3H), 7.86 (dt, *J* = 7.2, 1.3 Hz, 1H), 7.75 – 7.69 (m, 2H), 7.61 (dd, *J* = 7.5, 1.6 Hz, 1H), 7.55 – 7.38 (m, 7H), 7.22 – 7.06 (m, 8H), 6.91 – 6.86 (m, 1H), 6.68 – 6.64 (m, 1H), 5.53 (d, *J* = 2.2 Hz, 1H), 3.41 – 3.34 (m, 2H), 2.83 (t, *J* = 7.8 Hz, 2H), 2.43 (s, 3H), 2.42 (s, 3H), 1.96 (s, 6H), 1.92 (s, 6H), 1.78 – 1.73 (m, 2H), 1.45 (d, *J* = 7.6 Hz, 2H), 1.38 – 1.31 (m, 17H), 1.23 (s, 9H), 0.96 – 0.86 (m, 10H).

^{13}C NMR (151 MHz, Acetone-*d6*/CS_2) δ 152.14, 151.69, 146.65, 146.47, 144.62, 144.54, 142.33, 140.96, 139.57, 139.48, 139.26, 138.84, 137.71, 137.62, 137.43, 137.40, 137.28, 136.93, 135.66, 134.27, 134.06, 133.96, 132.78, 132.58, 132.15, 132.03, 131.81, 131.78, 131.64, 131.48, 131.34, 131.11, 130.40, 130.30, 130.26, 129.49, 128.23, 127.49, 127.21, 126.37, 126.31, 126.26, 126.05, 125.81, 125.04, 124.91, 124.71, 123.83, 123.81, 123.43, 123.02, 122.49, 122.24, 121.66, 121.19, 119.17, 115.60, 109.94, 108.13, 95.69, 93.41, 93.12, 90.62, 88.32, 54.95, 42.19, 37.71, 37.13, 37.00, 35.51, 35.46, 35.25, 35.06, 34.64, 33.37, 32.87, 32.81, 32.70, 32.53, 31.60, 31.51, 28.56, 27.82, 26.19, 23.67, 23.62, 23.31, 23.12, 21.61, 21.58, 21.40, 21.23, 19.98, 19.30, 17.89, 17.79, 14.92, 14.87, 14.77, 14.72, 12.04.

^{11}B NMR (128 MHz, $CDCl_3$/CS_2) δ 40.1.

HRMS (MALDI-TOF) m/z: $[M]^+$ Calculated for $C_{96}H_{90}BN_3$ 1295.7237; Found 1295.7192 (-3.5 ppm).

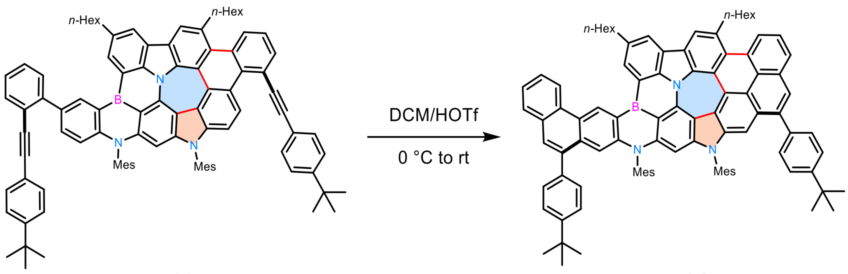

33 **34**

Compound **33** (10.0 mg, 0.038 mmol, 1.0 equiv.) was dissolved in anhydrous DCM (5.0 mL) under N_2 atmosphere. After cooled to 0 °C, HOTf (10 μl) was injected via syringe. The mixture was naturally warmed to rt and stirred for 20 min. The mixture was quenched by $NaHCO_3$ (aq.) and extracted with DCM. The residue was further purified by preparative thin-layer chromatography using Hex/DCM (v/v = 3:1) as the eluent to give compound **34** (5.2 mg, 52% yield) as orange-red solid.

^{1}H NMR (600 MHz, Acetone-*d6*/CS_2) δ 9.23 (d, *J* = 2.3 Hz, 1H), 8.80 (s, 1H), 8.43 (d, *J* = 8.2 Hz, 1H), 8.39 (d, *J* = 1.7 Hz, 1H), 8.29 (dt, *J* = 8.9, 1.6 Hz, 1H), 8.23 – 8.15 (m, 2H), 7.84 – 7.54 (m, 7H), 7.52 – 7.37 (m, 7H), 7.12 (s, 2H), 7.06 (s, 2H), 6.87 (d, *J* = 8.8 Hz, 1H), 6.62 (d, *J* = 8.8 Hz, 1H), 5.57 (s, 1H), 3.43 – 3.37 (m, 2H), 3.11 (t, *J* = 7.7 Hz, 2H), 2.43 – 2.42 (m, 6H), 2.12 – 2.07 (m, 2H), 2.00 (t, *J* = 7.7 Hz, 2H), 1.92 (s, 6H), 1.89 (s, 6H), 1.66 – 1.32 (m, 30H), 1.00 (t, *J* = 7.2 Hz, 3H), 0.92 (t, *J* = 7.2 Hz, 3H).

^{13}C NMR (151 MHz, Acetone-*d6*/CS_2) δ 151.79, 146.50, 146.44, 144.38, 142.27, 141.63, 140.89, 139.45, 139.38, 139.32, 139.13, 137.50, 137.25, 137.20, 136.91, 134.65, 133.97, 133.54, 132.87, 132.25, 132.09, 132.04, 131.50, 131.47, 131.11, 131.07, 130.21, 129.97, 129.60, 127.47, 127.24, 127.03, 126.13, 126.12, 125.01, 124.76, 123.89, 123.75, 123.70, 123.13, 122.60, 122.41,

121.33, 121.29, 119.32, 116.42, 115.81, 111.86, 108.01, 91.34, 90.97, 90.75, 90.19, 88.30, 37.76, 37.39, 35.14, 35.13, 33.39, 33.16, 33.01, 32.87, 31.66, 23.85, 21.72, 17.91, 17.80, 15.04, 14.95.

^{11}B NMR (128 MHz, $CDCl_3/CS_2$) δ 41.7.

HRMS (MALDI-TOF) m/z: $[M]^+$ Calculated for $C_{96}H_{90}BN_3$ 1295.7237; Found 1295.7173 (-4.9 ppm).

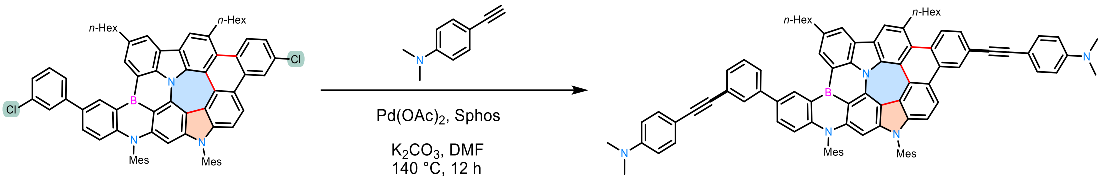

10g **35**

Compound **10f** (40.0 mg, 0.038 mmol, 1.0 equiv.), Pd(OAc)$_2$/Sphos (10 mg/12 mg) and K_2CO_3 (105 mg, ca. 20.0 equiv.) were added to a 50 mL flask. After vacuumed and backfilled with nitrogen, DMF (5.0 mL) was added. 4-Ethynyl-*N*,*N*-dimethylaniline (17.0 mg, ca. 3.0 equiv.) was finally added under N_2 flow. The mixture stirred at 140 °C for 12 h. After cooling to rt, the mixture was poured into brine. The precipitate was collected by filtration and was further purified by column chromatography on silica gel (hexane/EA = 12/1 − 8/1) to give compound **35** (31.3 mg, 65% yield) as orange-red solid.

^{1}H NMR (600 MHz, Acetone-*d6*/CS_2) δ 9.25 (d, *J* = 2.5 Hz, 1H), 8.83 (d, *J* = 2.2 Hz, 1H), 8.53 (q, *J* = 1.8 Hz, 1H), 8.36 – 8.31 (m, 1H), 8.24 (dt, *J* = 8.5, 1.6 Hz, 1H), 8.20 (q, *J* = 3.2 Hz, 2H), 7.86 (p, *J* = 1.7 Hz, 1H), 7.71 (ddd, *J* = 8.4, 4.3, 1.8 Hz, 2H), 7.52 – 7.46 (m, 2H), 7.43 (d, *J* = 7.5 Hz, 1H), 7.42 – 7.31 (m, 4H), 7.13 (s, 2H), 7.07 (s, 2H), 6.90 (dt, *J* = 8.7, 1.3 Hz, 1H), 6.76 – 6.67 (m, 4H), 6.66 – 6.61 (m, 1H), 5.58 (s, 1H), 3.42 (t, *J* = 8.0 Hz, 2H), 3.11 (t, *J* = 7.7 Hz, 2H), 3.05 (d, *J* = 1.9 Hz, 12H), 2.43 (s, 3H), 2.42 (s, 3H), 1.98 (q, *J* = 7.7 Hz, 4H), 1.93 (s, 6H), 1.90 (s, 6H), 1.60 – 1.55 (m, 2H), 1.50 – 1.36 (m, 10H), 0.96 – 0.90 (m, 6H).

^{13}C NMR (151 MHz, Acetone-*d6*/CS_2) δ 150.88, 150.86, 146.54, 144.49, 142.32, 142.14, 140.98, 139.54, 139.45, 139.36, 139.22, 137.60, 137.58, 137.33, 137.31, 137.01, 134.68, 134.08, 133.46, 133.42, 132.50, 132.01, 131.64, 131.56, 131.46, 131.11, 130.50, 130.25, 130.15, 130.01, 129.80, 128.94, 127.41, 126.99, 126.88, 126.53, 126.18, 125.87, 124.88, 124.66, 124.04, 123.53, 123.38, 123.13, 122.58, 122.28, 116.45, 112.68, 112.64, 111.90, 110.85, 108.02, 92.84, 92.16, 89.19, 88.65, 88.32, 54.93, 42.19, 40.40, 37.85, 37.38, 37.00, 35.54, 35.53, 35.48, 34.65, 33.19, 33.14, 32.90, 32.74, 32.54, 30.69, 30.47, 28.57, 27.86, 27.84, 26.24, 26.22, 23.75, 23.65, 23.35, 23.17, 23.15, 21.63, 21.44, 21.29, 21.27, 20.01, 19.34, 17.89, 17.78, 14.97, 14.89, 14.84, 14.77, 12.10, 12.08.

^{11}B NMR (128 MHz, $CDCl_3/CS_2$) δ 39.4.

HRMS (MALDI-TOF) m/z: $[M]^+$ Calculated for $C_{92}H_{84}BN_5$ 1269.6828; Found 1269.6772 (-4.4 ppm).

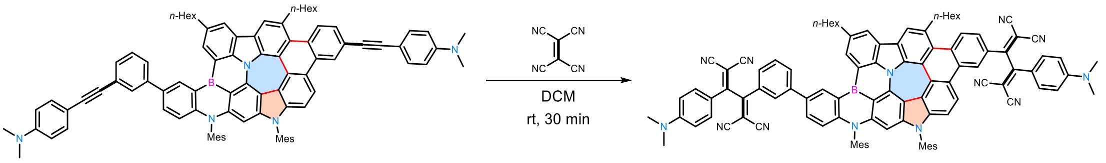

35 **36**

Compound **35** (15.0 mg, 0.011 mmol, 1.0 equiv.) was dissolved in anhydrous DCM (5.0 mL) under N_2 atmosphere. Tetracyanoethylene (5.0 mg, ca. 3.0 equiv.) was added. The mixture was stirred at rt for 30 min. The mixture was quenched by $NaHCO_3$ (aq.) and extracted with DCM. The residue was further purified by preparative thin-layer chromatography using Hex/DCM (v/v = 1:1) as the eluent to give compound **36** (15.6 mg, 87% yield) as dark-brown solid.

^{1}H NMR (400 MHz, $CDCl_3$) δ 9.20 (s, 1H), 9.04 (s, 1H), 8.76 (s, 1H), 8.45 – 8.22 (m, 2H), 8.18 – 8.03 (m, 4H), 7.95 – 7.78 (m, 4H), 7.72 – 7.53 (m, 4H), 7.11 – 6.90 (m, 5H), 6.82 – 6.59 (m, 5H), 5.46 (s, 1H), 3.32 (s, 2H), 3.16 – 3.05 (d, J = 8.7 Hz, 14H), 2.38 – 2.36 (m, 6H), 2.01 – 1.73 (m, 14H), 1.46 – 1.17 (m, 14H), 0.88 (s, 6H).

^{13}C NMR (151 MHz, Acetone-*d6*/CS_2) δ 169.40, 168.52, 163.45, 155.35, 146.78, 144.74, 143.78, 142.31, 141.33, 139.92, 139.65, 137.54, 137.24, 137.17, 134.88, 134.21, 133.63, 133.39, 133.03, 132.30, 131.78, 131.67, 131.20, 131.04, 130.33, 128.43, 128.29, 128.25, 128.01, 126.31, 126.03, 124.48, 124.36, 124.13, 123.77, 122.52, 117.89, 116.78, 114.94, 114.65, 113.60, 113.28, 113.04, 112.48, 88.67, 75.38, 60.52, 54.96, 42.17, 40.30, 37.62, 36.99, 35.48, 35.45, 34.62, 33.29, 33.11, 32.89, 32.67, 32.50, 28.53, 27.77, 26.12, 23.61, 23.09, 23.07, 21.49, 21.35, 21.17, 19.92, 19.24, 17.77, 17.65, 14.84, 14.67, 11.97.

^{11}B NMR (128 MHz, $CDCl_3/CS_2$) δ 40.0.

HRMS (MALDI-TOF) m/z: $[M]^+$ Calculated for $C_{104}H_{84}BN_{13}$ 1526.7100; Found 1526.6942 (-10.3 ppm).

Scale-up reaction

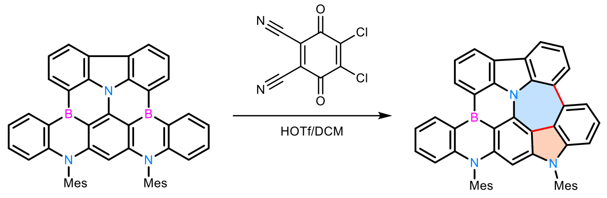

1 **2**

For the scale-up BDA reaction of **1** to **2**: compound **1**(1.0 g, 1.5 mmol, 1.0 equiv.) and DDQ (1.01 g, 4.5 mmol, 3.0 equiv.) was used with variable concentration and ratio of HOTf/DCM. Entry b: HOTf/DCM = 4 mL/200 mL; Entry c: HOTf/DCM = 4 mL/100 mL; Entry d: HOTf/DCM = 1 mL/50 mL. The concentration and ratio of HOTf/DCM had great effect on the conversion of scale-up DBA reaction of **1**. The entry d showed a full conversion of **1**, and 726 mg of **2** was isolated (74% yield).

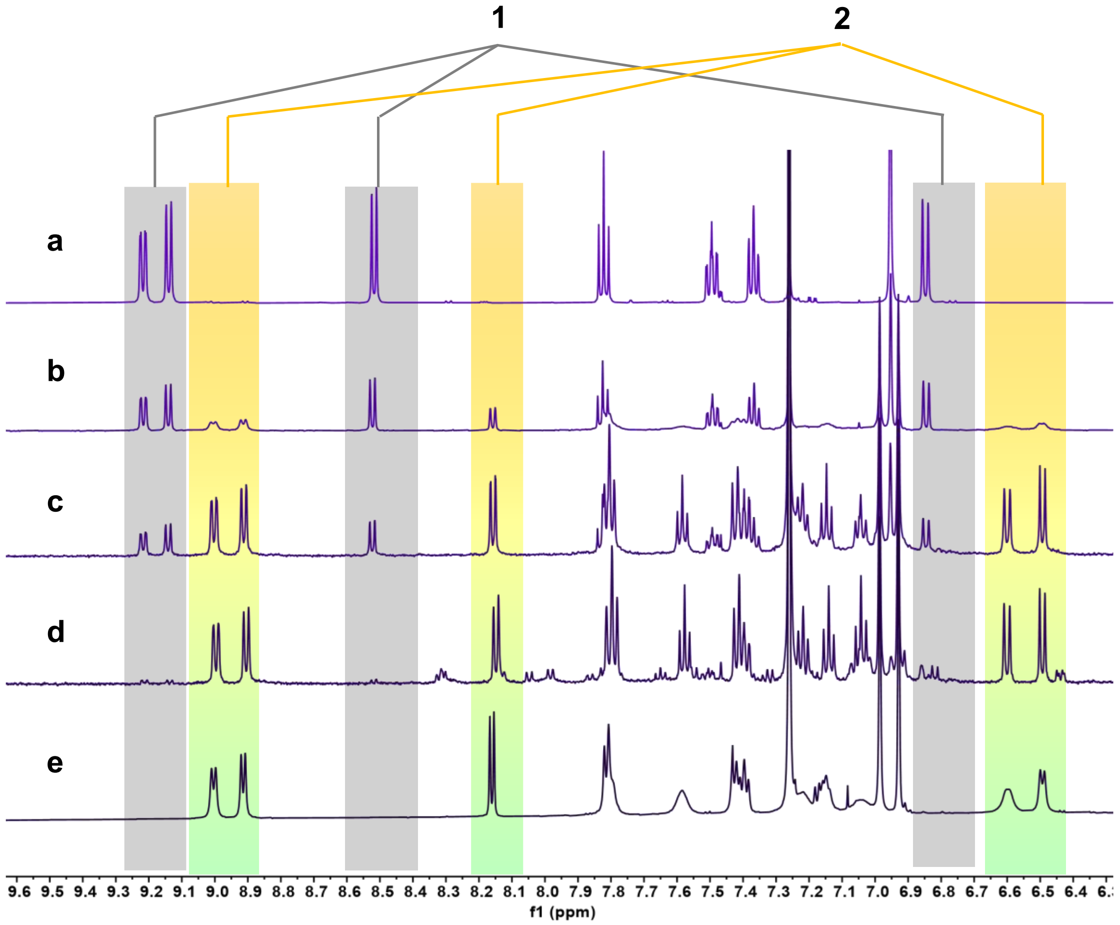

Fig. S1. ^{1}H NMR spectra of **1** (a), mixture under different reaction conditions (b-d), and the purified **2** (e) from entry d.

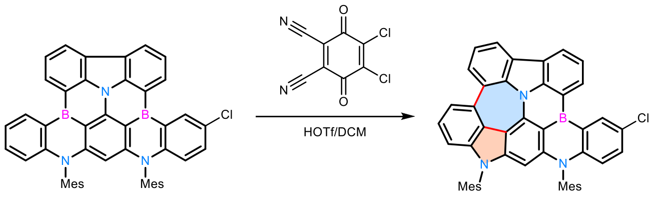

For the scale-up BDA reaction of **3c** to **5c**: compound **3c** (800 mg, 1.12 mmol, 1.0 equiv.) and DDQ (765 mg, 3.37 mmol, 3.0 equiv.) were added to a 50 mL vial and was dried in vacuo overnight. Anhydrous DCM (10.0 mL) was added under N_2. HOTf (1.0 mL) was finally added. The reaction mixture stirred at 30 °C for 24 h. The reaction mixture was poured into saturated Na_2CO_3 (aq.) and extracted with DCM. The residue was finally purified by column chromatography on silica gel (hexane/DCM = 12/1 − 4/1) to give compound **5c** (487 mg, 62% yield) as orange solid.

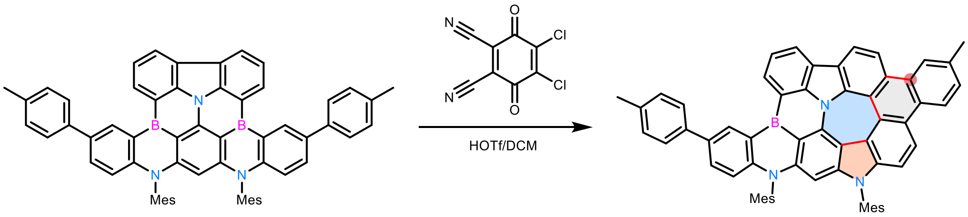

10k (263 mg, 76% yield, brown-red solid) was synthesized from **9k** (350 mg, 0.4 mmol, 1.0 equiv.) following modified **General Procedure B** with DCM (40 mL)/HOTf (1.0 mL), and reacted at rt for 30 min. Purification by column chromatography on silica gel (hexane/DCM = 12/1 – 4/1).

^{1}H NMR (600 MHz, Acetone-*d6*/CS_2) δ 9.14 (d, *J* = 2.3 Hz, 1H), 8.97 (d, *J* = 7.4 Hz, 1H), 8.59 (d, *J* = 8.7 Hz, 1H), 8.49 (s, 1H), 8.40 (d, *J* = 8.3 Hz, 1H), 8.32 (t, *J* = 8.5 Hz, 2H), 8.18 (d, *J* = 8.6 Hz, 1H), 7.68 (t, *J* = 7.4 Hz, 1H), 7.61 – 7.57 (m, 3H), 7.44 (d, *J* = 8.3 Hz, 1H), 7.27 (d, *J* = 7.6 Hz, 2H), 7.09 (s, 2H), 7.04 (s, 2H), 6.86 (d, *J* = 8.7 Hz, 1H), 6.57 (d, *J* = 8.7 Hz, 1H), 5.59 (s, 1H), 2.66 (s, 3H), 2.49 – 2.41 (m, 9H), 1.91 (s, 6H), 1.88 (s, 6H).

^{13}C NMR (151 MHz, Acetone-*d6*/CS_2) δ 146.09, 145.77, 144.18, 142.17, 141.80, 141.41, 139.13, 138.96, 138.77, 138.42, 137.83, 137.46, 137.26, 137.21, 136.39, 135.98, 134.79, 134.20, 133.51, 133.18, 131.51, 131.45, 130.91, 130.45, 130.41, 130.24, 130.16, 130.07, 129.91, 129.53, 129.23, 128.79, 128.07, 127.35, 127.04, 126.68, 126.52, 126.17, 125.14, 125.00, 123.84, 123.68, 123.54, 123.34, 122.55, 122.39, 122.25, 121.12, 120.84, 116.09, 111.56, 107.96, 88.41, 22.50, 21.98, 21.83, 21.82, 21.63, 17.96, 17.82.

^{11}B NMR (128 MHz, $CDCl_3$) δ 39.3.

HRMS (MALDI-TOF) m/z: $[M]^+$ Calculated for $C_{62}H_{46}BN_3$ 843.3789; Found 843.3893.

Attempts for the boron-deleting annulation of model BDA product 2

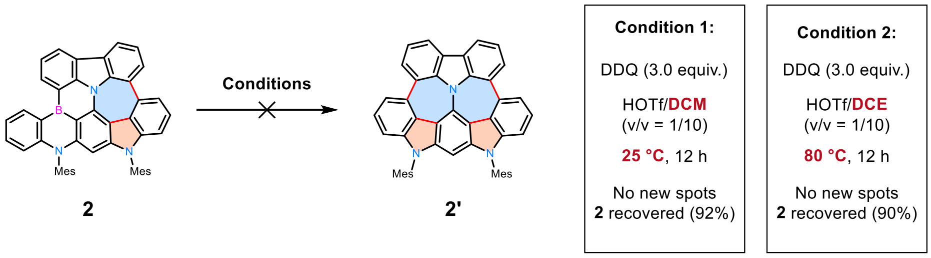

Fig. S2. Attempts for the boron-deleting annulation of model BDA product **2**.

5. SC-XRD data

Table S6. Crystallographic data and details of the structural refinements of **2**.

Identification code	2431227
Empirical formula	$C_{48}H_{36}BN_3$
Formula weight	665.61
Temperature/K	223.00
Crystal system	monoclinic
Space group	$P2_1/c$
a/Å	18.9116(4)
b/Å	10.1412(3)
c/Å	17.8053(4)
α/°	90
β/°	93.989(2)
γ/°	90
Volume/Å^3	3406.54(15)
Z	4
ρ_{calc}g/cm^3	1.298
μ/mm^{-1}	0.575
F(000)	1400.0
Crystal size/mm^3	0.13 × 0.12 × 0.11
Radiation	CuKα (λ = 1.54178)
2Θ range for data collection/°	9.376 to 136.798
Index ranges	$-22 \leq h \leq 22, -12 \leq k \leq 12, -19 \leq l \leq 21$
Reflections collected	31622
Independent reflections	6243 [R_{int} = 0.0714, R_{sigma} = 0.0479]
Data/restraints/parameters	6243/0/476
Goodness-of-fit on F^2	1.044
Final R indexes [I>=2σ (I)]	R_1 = 0.0457, wR_2 = 0.1130
Final R indexes [all data]	R_1 = 0.0689, wR_2 = 0.1264
Largest diff. peak/hole / e Å^{-3}	0.24/-0.17

Table S7. Crystallographic data and details of the structural refinements of **5b**.

Identification code	2431233
Empirical formula	$C_{54}H_{40}BN_3O$
Formula weight	757.70
Temperature/K	223.00
Crystal system	monoclinic
Space group	$P2_1/n$
a/Å	23.0109(13)
b/Å	7.5121(4)
c/Å	25.7255(15)
α/°	90
β/°	116.234(4)
γ/°	90
Volume/Å^3	3988.9(4)
Z	4
ρ_{calc}g/cm^3	1.262
μ/mm^{-1}	0.576
F(000)	1592.0
Crystal size/mm^3	0.14 × 0.12 × 0.1
Radiation	CuKα (λ = 1.54178)
2Θ range for data collection/°	4.3 to 136.802
Index ranges	$-22 \leq h \leq 27$, $-9 \leq k \leq 8$, $-30 \leq l \leq 28$
Reflections collected	29038
Independent reflections	7273 [R_{int} = 0.0589, R_{sigma} = 0.0411]
Data/restraints/parameters	7273/0/539
Goodness-of-fit on F^2	1.009
Final R indexes [I>=2σ (I)]	R_1 = 0.0463, wR_2 = 0.1191
Final R indexes [all data]	R_1 = 0.0715, wR_2 = 0.1343
Largest diff. peak/hole / e Å^{-3}	0.19/-0.18

Table S8. Crystallographic data and details of the structural refinements of **5c**.

Identification code	2431234
Empirical formula	$C_{48}H_{35}BClN_3$
Formula weight	700.05
Temperature/K	223.00
Crystal system	monoclinic
Space group	C2/c
a/Å	45.066(3)
b/Å	7.4791(4)
c/Å	26.8047(15)
α/°	90
β/°	120.440(3)
γ/°	90
Volume/Å^3	7789.3(8)
Z	8
ρ_{calc}g/cm^3	1.194
μ/mm^{-1}	1.144
F(000)	2928.0
Crystal size/mm^3	0.15 × 0.13 × 0.12
Radiation	CuKα (λ = 1.54178)
2Θ range for data collection/°	6.63 to 145.104
Index ranges	$-55 \leq h \leq 51, -7 \leq k \leq 9, -30 \leq l \leq 33$
Reflections collected	41181
Independent reflections	7665 [R_{int} = 0.0453, R_{sigma} = 0.0298]
Data/restraints/parameters	7665/0/484
Goodness-of-fit on F^2	1.054
Final R indexes [I>=2σ (I)]	R_1 = 0.0481, wR_2 = 0.1376
Final R indexes [all data]	R_1 = 0.0590, wR_2 = 0.1470
Largest diff. peak/hole / e Å^{-3}	0.93/-0.39

Table S9. Crystallographic data and details of the structural refinements of **8a**.

Identification code	2431235
Empirical formula	$C_{48}H_{34}BBr_2N_3$
Formula weight	823.41
Temperature/K	223.00
Crystal system	monoclinic
Space group	$P2_1/n$
a/Å	12.8304(5)
b/Å	14.7588(8)
c/Å	20.3632(9)
α/°	90
β/°	106.698(3)
γ/°	90
Volume/Å^3	3693.4(3)
Z	4
ρ_{calc}g/cm^3	1.481
μ/mm^{-1}	3.086
F(000)	1672.0
Crystal size/mm^3	0.19 × 0.12 × 0.11
Radiation	CuKα (λ = 1.54178)
2Θ range for data collection/°	7.318 to 137.048
Index ranges	$-15 \leq h \leq 15, -17 \leq k \leq 14, -24 \leq l \leq 22$
Reflections collected	28585
Independent reflections	6732 [R_{int} = 0.0650, R_{sigma} = 0.0455]
Data/restraints/parameters	6732/37/481
Goodness-of-fit on F^2	1.050
Final R indexes [I>=2σ (I)]	R_1 = 0.0478, wR_2 = 0.1250
Final R indexes [all data]	R_1 = 0.0697, wR_2 = 0.1383
Largest diff. peak/hole / e Å^{-3}	0.89/-0.69

Table S10. Crystallographic data and details of the structural refinements of **8b**.

Identification code	2431236
Empirical formula	$C_{52}H_{44}BN_3$
Formula weight	721.71
Temperature/K	223.00
Crystal system	triclinic
Space group	P-1
a/Å	13.2867(4)
b/Å	14.8700(4)
c/Å	21.0717(6)
α/°	92.005(2)
β/°	100.231(2)
γ/°	101.568(2)
Volume/Å^3	4003.0(2)
Z	4
ρ_{calc}g/cm^3	1.198
μ/mm^{-1}	0.526
F(000)	1528.0
Crystal size/mm^3	0.13 × 0.12 × 0.11
Radiation	CuKα (λ = 1.54178)
2Θ range for data collection/°	4.272 to 137.064
Index ranges	-15 ≤ h ≤ 16, -17 ≤ k ≤ 17, -24 ≤ l ≤ 25
Reflections collected	50361
Independent reflections	14656 [R_{int} = 0.0594, R_{sigma} = 0.0474]
Data/restraints/parameters	14656/3289/1542
Goodness-of-fit on F^2	1.050
Final R indexes [I>=2σ (I)]	R_1 = 0.0754, wR_2 = 0.2217
Final R indexes [all data]	R_1 = 0.1043, wR_2 = 0.2465
Largest diff. peak/hole / e Å^{-3}	0.54/-0.39

Table S11. Crystallographic data and details of the structural refinements of **8p**.

Identification code	2431237
Empirical formula	$C_{68}H_{64}BN_3$
Formula weight	934.03
Temperature/K	213.00
Crystal system	triclinic
Space group	P-1
a/Å	12.6032(4)
b/Å	14.8701(6)
c/Å	15.4276(5)
α/°	85.259(2)
β/°	67.305(2)
γ/°	86.856(2)
Volume/Å^3	2657.48(17)
Z	2
ρ_{calc}g/cm^3	1.167
μ/mm^{-1}	0.505
F(000)	996.0
Crystal size/mm^3	0.13 × 0.12 × 0.1
Radiation	CuKα (λ = 1.54178)
2Θ range for data collection/°	5.966 to 136.482
Index ranges	$-15 \leq h \leq 15$, $-17 \leq k \leq 17$, $-18 \leq l \leq 14$
Reflections collected	33192
Independent reflections	9692 [R_{int} = 0.0684, R_{sigma} = 0.0751]
Data/restraints/parameters	9692/247/731
Goodness-of-fit on F^2	1.036
Final R indexes [I>=2σ (I)]	R_1 = 0.0707, wR_2 = 0.2014
Final R indexes [all data]	R_1 = 0.1000, wR_2 = 0.2372
Largest diff. peak/hole / e Å^{-3}	0.33/-0.27

Table S12. Crystallographic data and details of the structural refinements of **10d**.

Identification code	2431222
Empirical formula	$C_{74}H_{70}BN_3O_2$
Formula weight	1044.14
Temperature/K	223.00
Crystal system	triclinic
Space group	P-1
a/Å	10.1036(3)
b/Å	16.9530(5)
c/Å	18.9091(6)
α/°	64.878(2)
β/°	87.924(2)
γ/°	73.239(2)
Volume/Å^3	2793.99(16)
Z	2
ρ_{calc}g/cm^3	1.241
μ/mm^{-1}	0.563
F(000)	1112.0
Crystal size/mm^3	0.15 × 0.14 × 0.11
Radiation	CuKα (λ = 1.54178)
2Θ range for data collection/°	6.04 to 136.852
Index ranges	$-12 \leq h \leq 12$, $-20 \leq k \leq 20$, $-22 \leq l \leq 22$
Reflections collected	33005
Independent reflections	10246 [R_{int} = 0.0570, R_{sigma} = 0.0507]
Data/restraints/parameters	10246/6/732
Goodness-of-fit on F^2	1.049
Final R indexes [I>=2σ (I)]	R_1 = 0.0471, wR_2 = 0.1212
Final R indexes [all data]	R_1 = 0.0681, wR_2 = 0.1355
Largest diff. peak/hole / e Å^{-3}	0.22/-0.20

Table S13. Crystallographic data and details of the structural refinements of **10i**.

Identification code	2431223
Empirical formula	$C_{74}H_{66}BN_5$
Formula weight	1036.12
Temperature/K	223.00
Crystal system	monoclinic
Space group	$P2_1/n$
a/Å	20.8239(11)
b/Å	10.8612(5)
c/Å	25.4252(12)
α/°	90
β/°	96.975(4)
γ/°	90
Volume/Å^3	5707.9(5)
Z	4
ρ_{calc}g/cm^3	1.206
μ/mm^{-1}	0.533
F(000)	2200.0
Crystal size/mm^3	0.13 × 0.12 × 0.11
Radiation	CuKα (λ = 1.54178)
2Θ range for data collection/°	5.186 to 137.28
Index ranges	$-25 \leq h \leq 22, -13 \leq k \leq 11, -29 \leq l \leq 30$
Reflections collected	40432
Independent reflections	10452 [R_{int} = 0.0800, R_{sigma} = 0.0663]
Data/restraints/parameters	10452/273/804
Goodness-of-fit on F^2	1.028
Final R indexes [I>=2σ (I)]	R_1 = 0.0569, wR_2 = 0.1433
Final R indexes [all data]	R_1 = 0.0969, wR_2 = 0.1714
Largest diff. peak/hole / e Å^{-3}	0.25/-0.30

Table S14. Crystallographic data and details of the structural refinements of **13**.

Identification code	2431224
Formula weight	1066.80
Temperature/K	193.00
Crystal system	monoclinic
Space group	C2/c
a/Å	43.7364(17)
b/Å	20.6725(8)
c/Å	16.0686(6)
α/°	90
β/°	107.644(2)
γ/°	90
Volume/Å^3	13844.8(9)
Z	8
ρ_{calc}g/cm^3	1.024
μ/mm^{-1}	1.132
F(000)	4512.0
Crystal size/mm^3	0.13 × 0.12 × 0.11
Radiation	CuKα (λ = 1.54178)
2Θ range for data collection/°	8.358 to 136.784
Index ranges	-52 ≤ h ≤ 52, -24 ≤ k ≤ 24, -14 ≤ l ≤ 19
Reflections collected	54820
Independent reflections	12431 [R_{int} = 0.0795, R_{sigma} = 0.0516]
Data/restraints/parameters	12431/309/782
Goodness-of-fit on F^2	0.916
Final R indexes [I>=2σ (I)]	R_1 = 0.0990, wR_2 = 0.1823
Final R indexes [all data]	R_1 = 0.1363, wR_2 = 0.2016
Largest diff. peak/hole / e Å^{-3}	0.94/-0.90

Table S15. Crystallographic data and details of the structural refinements of **14**.

Identification code	2431225
Empirical formula	$C_{72}H_{66}BCl_2N_3$
Formula weight	1054.98
Temperature/K	223.00
Crystal system	triclinic
Space group	P-1
a/Å	12.2714(3)
b/Å	14.9809(4)
c/Å	16.1184(4)
α/°	80.9560(10)
β/°	89.534(2)
γ/°	81.756(2)
Volume/Å^3	2895.76(13)
Z	2
ρ_{calc}g/cm^3	1.210
μ/mm^{-1}	1.351
F(000)	1116.0
Crystal size/mm^3	0.15 × 0.13 × 0.12
Radiation	CuKα (λ = 1.54178)
2Θ range for data collection/°	7.28 to 145.088
Index ranges	-15 ≤ h ≤ 15, -18 ≤ k ≤ 18, -19 ≤ l ≤ 19
Reflections collected	33807
Independent reflections	11331 [R_{int} = 0.0367, R_{sigma} = 0.0378]
Data/restraints/parameters	11331/0/711
Goodness-of-fit on F^2	1.058
Final R indexes [I>=2σ (I)]	R_1 = 0.0665, wR_2 = 0.1958
Final R indexes [all data]	R_1 = 0.0820, wR_2 = 0.2107
Largest diff. peak/hole / e Å^{-3}	0.59/-1.02

Table S16. Crystallographic data and details of the structural refinements of **16a**.

Identification code	2431226
Empirical formula	$C_{175}H_{148}B_2N_6$
Formula weight	2356.61
Temperature/K	213.00
Crystal system	triclinic
Space group	P-1
a/Å	13.6288(3)
b/Å	15.3318(3)
c/Å	16.7967(3)
α/°	99.0280(10)
β/°	104.6690(10)
γ/°	105.6160(10)
Volume/Å^3	3173.15(11)
Z	1
ρ_{calc}g/cm^3	1.233
μ/mm^{-1}	0.534
F(000)	1250.0
Crystal size/mm^3	0.13 × 0.12 × 0.1
Radiation	CuKα (λ = 1.54178)
2Θ range for data collection/°	7.084 to 159.334
Index ranges	$-16 \le h \le 17$, $-19 \le k \le 19$, $-20 \le l \le 18$
Reflections collected	35373
Independent reflections	12952 [R_{int} = 0.0396, R_{sigma} = 0.0369]
Data/restraints/parameters	12952/173/910
Goodness-of-fit on F^2	1.063
Final R indexes [I>=2σ (I)]	R_1 = 0.0575, wR_2 = 0.1702
Final R indexes [all data]	R_1 = 0.0784, wR_2 = 0.1891
Largest diff. peak/hole / e Å^{-3}	0.51/-0.48

Table S17. Crystallographic data and details of the structural refinements of **25a**.

Identification code	2431232
Empirical formula	$C_{74}H_{52}BN_3$
Formula weight	993.99
Temperature/K	223.00
Crystal system	triclinic
Space group	P-1
a/Å	8.1836(3)
b/Å	19.6890(6)
c/Å	21.0349(6)
α/°	69.639(2)
β/°	79.963(2)
γ/°	87.613(2)
Volume/Å^3	3128.23(18)
Z	2
ρ_{calc}g/cm^3	1.055
μ/mm^{-1}	0.463
F(000)	1044.0
Crystal size/mm^3	0.13 × 0.11 × 0.1
Radiation	CuKα (λ = 1.54178)
2Θ range for data collection/°	7.664 to 136.914
Index ranges	$-9 \leq h \leq 9$, $-23 \leq k \leq 23$, $-25 \leq l \leq 25$
Reflections collected	42363
Independent reflections	11414 [R_{int} = 0.0635, R_{sigma} = 0.0569]
Data/restraints/parameters	11414/0/711
Goodness-of-fit on F^2	1.104
Final R indexes [I>=2σ (I)]	R_1 = 0.0601, wR_2 = 0.1788
Final R indexes [all data]	R_1 = 0.0911, wR_2 = 0.1958
Largest diff. peak/hole / e Å^{-3}	0.27/-0.22

6. Mechanistic study

6.1 Control experiments

a. In the absence of DDQ or HOTf, no desired product **2** was observed.

b. When HOTf was reduced to 3.0 equiv., only the trace amount of **2** was observed by TLC.

c. When the reaction was protected from ambient light by aluminum foil, no obvious decrease in yield of **2** was observed (79% vs 82%).

d. *In situ* NMR experiments:

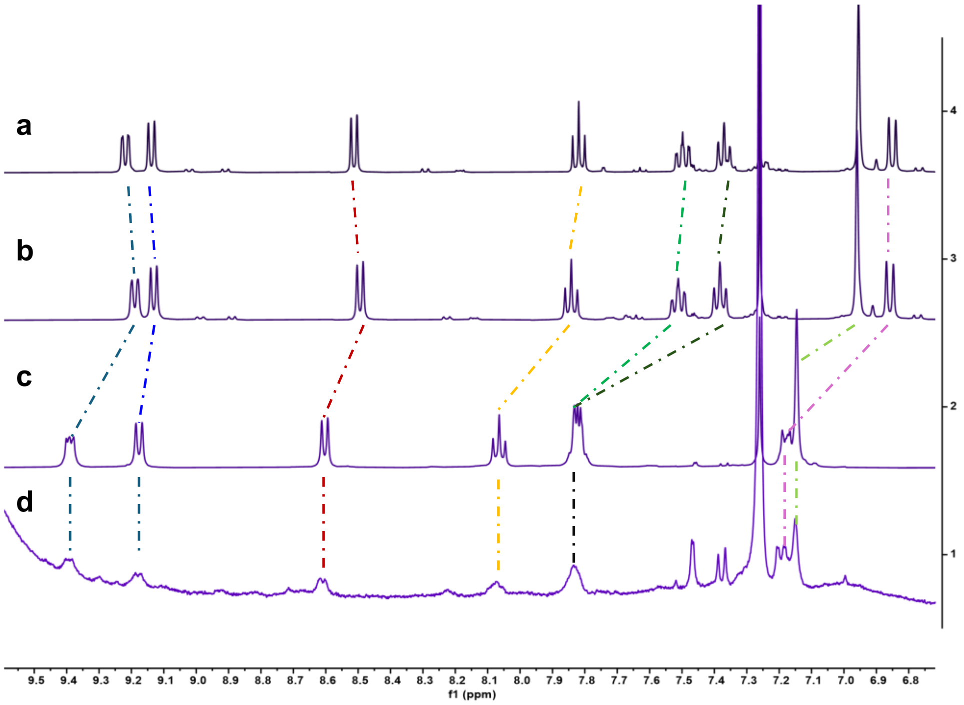

Fig. S3. ^{1}H NMR spectra (400 MHz, $CDCl_3$, 298 K) of **1** (a), **1**/DDQ (b), **1**/HOTf (c) and **1**/DDQ/HOTf (d).

The mixture of **1** and DDQ showed slight chemical shifts compared with **1**. While the addition of HOTf induced an obvious down-field shift of aromatic peaks for **1**. The combination of **1**/DDQ/HOTf showed sluggish and broadened peaks.

6.2 Electronic effects

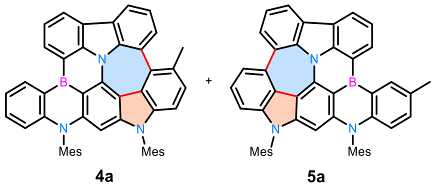

4a and **5a** were synthesized from **3a** following **General Procedure A**. Purification by column chromatography on silica gel (hexane/DCM = 10/1 – 4/1) afforded a mixture of **4a** and **5a** (ca. 2:3).

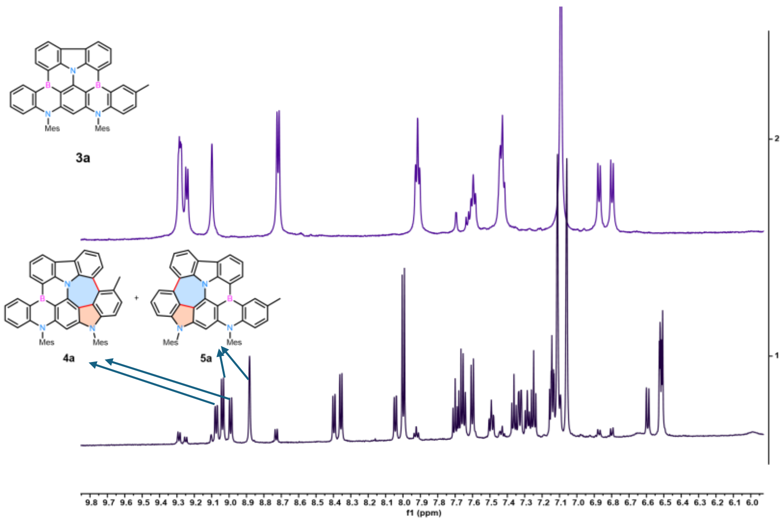

HRMS (MALDI-TOF) m/z: [M]$^+$ Calculated for $C_{49}H_{38}BN_3$ 679.3162; Found 679.3151 (1.6 ppm).

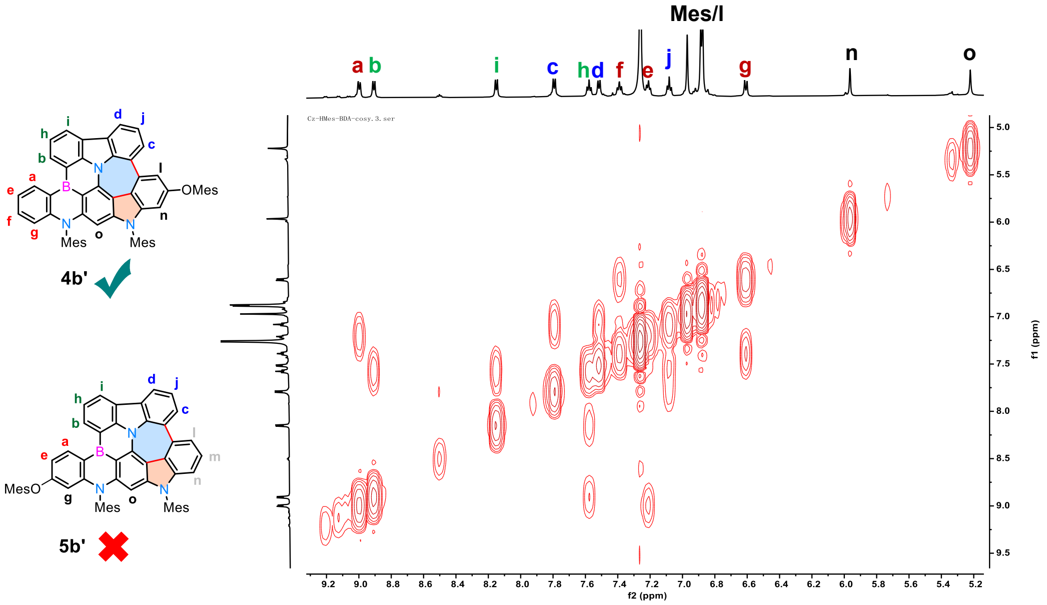

Fig. S4. The 2D ^{1}H-^{1}H COSY spectrum of **4b'** in $CDCl_3$ at 298K (600 MHz).

4b' was synthesized from **3b'** following **General Procedure A**. Purification by column chromatography on silica gel (hexane/DCM = 15/1 – 4/1) afforded **4b'**.

^{1}H NMR (600 MHz, $CDCl_3$) δ 9.00 (d, J = 7.8 Hz, 1H), 8.91 (d, J = 7.4 Hz, 1H), 8.15 (d, J = 7.4 Hz, 1H), 7.79 (d, J = 7.6 Hz, 1H), 7.58 (t, J = 7.5 Hz, 1H), 7.52 (d, J = 7.8 Hz, 1H), 7.39 (t, J = 7.8 Hz, 1H), 7.21 (t, J = 7.3 Hz, 1H), 7.08 (t, J = 7.6 Hz, 1H), 6.97 (s, 2H), 6.95 – 6.83 (m, 5H), 6.61 (d, J = 8.6 Hz, 1H), 5.96 (s, 1H), 5.22 (s, 1H), 2.31 (9H), 2.12 (s, 6H), 1.78 (12H).

^{13}C NMR (101 MHz, $CDCl_3$) δ 149.04, 146.17, 145.16, 142.13, 138.32, 138.16, 136.95, 136.90, 136.83, 135.63, 134.35, 133.09, 132.12, 131.84, 131.05, 130.89, 130.05, 129.74, 129.18, 128.68, 127.03, 123.63, 122.78, 122.01, 121.22, 119.87, 118.85, 115.22, 107.45, 103.65, 96.09, 88.09, 21.15, 21.10, 20.97, 17.47, 17.31, 16.55.

^{11}B NMR (128 MHz, $CDCl_3$) δ 37.4.

HRMS (MALDI-TOF) m/z: [M]$^+$ Calculated for $C_{57}H_{46}BN_3O$ 799.3738; Found 799.3779 (5.1 ppm).

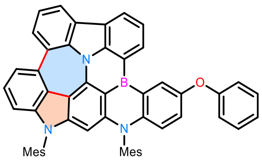

5b

5b was synthesized from **3b** following **General Procedure A**. Purification by column chromatography on silica gel (hexane/DCM = 15/1 – 4/1) afforded **5b**. Single crystal was grown by slow diffusion of methanol to the toluene solution of **5b**.

^{1}**H NMR** (600 MHz, $CDCl_3$) δ 8.70 (d, J = 7.4 Hz, 1H), 8.64 (d, J = 2.9 Hz, 1H), 8.12 (dd, J = 7.5, 1.1 Hz, 1H), 7.80 (d, J = 7.9 Hz, 2H), 7.56 – 7.38 (m, 3H), 7.38 – 7.32 (m, 2H), 7.17 – 7.06 (m, 5H), 6.99 (s, 2H), 6.93 (s, 2H), 6.64 – 6.55 (m, 1H), 6.53 – 6.46 (m, 1H), 5.30 (br, 1H), 2.35 (s, 3H), 2.32 (s, 3H), 1.81 (s, 6H), 1.80 (s, 6H).

^{13}**C NMR** (151 MHz, Acetone-*d6*/CS_2) δ 159.08, 150.30, 146.63, 145.24, 143.08, 142.66, 142.30, 141.73, 141.12, 139.08, 138.93, 137.41, 137.29, 137.16, 133.32, 131.68, 131.43, 130.96, 130.34, 130.04, 129.44, 128.10, 127.31, 125.46, 125.10, 124.90, 124.86, 124.43, 123.47, 123.11, 123.08, 122.95, 121.66, 118.42, 117.26, 116.82, 110.11, 106.84, 88.05, 21.72, 17.86, 17.75.

^{11}**B NMR** (128 MHz, $CDCl_3$) δ 38.0.

HRMS (MALDI-TOF) m/z: $[M]^+$ Calculated for $C_{54}H_{40}BN_3O$ 757.3268; Found 757.3276 (1.1 ppm).

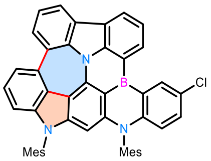

5c

5c was synthesized from **3c** ollowing **General Procedure A**. Purification by column chromatography on silica gel (hexane/DCM = 12/1 – 4/1) afforded **5c**. Single crystal was grown by slow diffusion of methanol to the toluene solution of **5c**.

^{1}**H NMR** (400 MHz, $CDCl_3$) δ 8.90 (d, J = 2.5 Hz, 1H), 8.83 (dd, J = 7.5, 1.1 Hz, 1H), 8.17 (dd, J = 7.5, 1.1 Hz, 1H), 7.80 (ddd, J = 7.4, 6.3, 1.3 Hz, 2H), 7.61 (t, J = 7.5 Hz, 1H), 7.42 (d, J = 7.9 Hz, 1H), 7.31 (dd, J = 9.1, 2.6 Hz, 1H), 7.15 (t, J = 7.7 Hz, 1H), 7.05 (t, J = 7.9 Hz, 1H), 6.99 (s, 2H), 6.93 (s, 2H), 6.51 (dd, J = 14.2, 8.5 Hz, 2H), 5.28 (s, 1H), 2.34 (s, 3H), 2.32 (s, 3H), 1.78 (s, 12H).

^{13}**C NMR** (101 MHz, $CDCl_3$) δ 146.05, 144.86, 144.61, 141.99, 141.36, 140.69, 138.48, 137.07, 136.74, 136.52, 134.62, 132.75, 131.94, 131.23, 130.89, 130.23, 129.20, 128.92, 127.66, 126.49, 125.36, 124.44, 124.10, 123.78, 123.09, 122.44, 122.31, 121.00, 116.87, 115.93, 109.71, 106.34, 101.62, 87.84, 21.19, 21.12, 17.43, 17.27.

^{11}**B NMR** (128 MHz, $CDCl_3$) δ 40.2.

HRMS (MALDI-TOF) m/z: $[M]^+$ Calculated for $C_{48}H_{35}BClN_3$ 699.2615; Found 699.2658 (6.1 ppm).

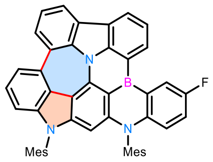

5d

5d was synthesized from **3d** following **General Procedure A**. Purification by column chromatography on silica gel (hexane/DCM = 12/1 − 4/1) afforded **5d**.

^{1}**H NMR** (600 MHz, Acetone-*d6*/CS_2) δ 8.79 (dd, *J* = 7.8, 2.6 Hz, 1H), 8.53 (dt, *J* = 9.5, 2.3 Hz, 1H), 8.17 (td, *J* = 5.8, 3.1 Hz, 1H), 7.79 (dtd, *J* = 11.0, 6.9, 2.3 Hz, 2H), 7.62 – 7.57 (m, 1H), 7.38 (dd, *J* = 7.6, 4.0 Hz, 1H), 7.17 – 7.12 (m, 1H), 7.10 – 6.95 (m, 6H), 6.47 (ddd, *J* = 9.3, 4.7, 2.6 Hz, 1H), 6.42 – 6.37 (m, 1H), 5.41 – 5.37 (m, 1H), 2.38 (s, 6H), 1.83 (s, 6H), 1.83 (s, 6H).

^{13}**C NMR** (151 MHz, Acetone-*d6*/CS_2) δ 158.34, 156.75, 146.59, 145.23, 143.00, 142.63, 142.30, 141.68, 141.09, 139.05, 138.95, 137.37, 137.22, 137.12, 136.24, 133.20, 131.67, 131.37, 130.97, 130.41, 130.02, 129.43, 128.09, 127.32, 124.89, 124.86, 124.44, 123.52, 123.14, 123.05, 121.67, 120.30, 120.20, 120.15, 120.07, 117.35, 117.30, 116.83, 110.11, 106.87, 88.00, 21.74, 17.85, 17.69.

^{19}**F NMR** (565 MHz, Acetone-*d6*/CS_2) δ -122.84.

^{11}**B NMR** (128 MHz, $CDCl_3$) δ 39.7.

HRMS (MALDI-TOF) m/z: $[M]^+$ Calculated for $C_{48}H_{35}BFN_3$ 683.2911; Found 683.2956 (6.6 ppm).

6.3 Interrupted BDA reaction

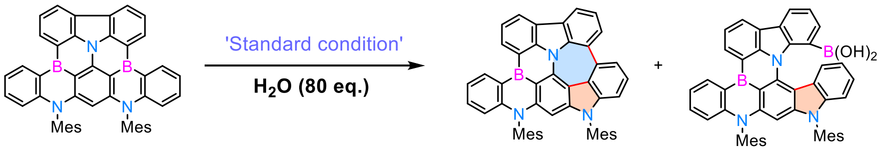

1 **2** (Trace) **6-Mes** (38%)

Compound **1** (34.0 mg, 0.05 mmol, 1.0 equiv.) and DDQ (34.0 mg, 0.15 mmol, 3.0 equiv.) were added to a 20 mL vial. The vial was then sealed and purged with nitrogen flow. Anhydrous DCM (1.5 mL) was injected via a syringe followed by 4 µl H_2O (80 equiv.). HOTf (150 uL) was finally injected via a micro-syringe with stainless needle. The vial was further sealed with electrical tape and stirred at room temperature for 12 h. The reaction mixture was poured into saturated Na_2CO_3 solution and extracted with DCM. The organic phase was dried over Na_2SO_4. After removal of solvent, the residue was purified by column chromatography using hexane/DCM = 4:1 to hexane/EA = 8:1 to give **6-Mes** (13.7 mg, 38% yield) as dark-green solid.

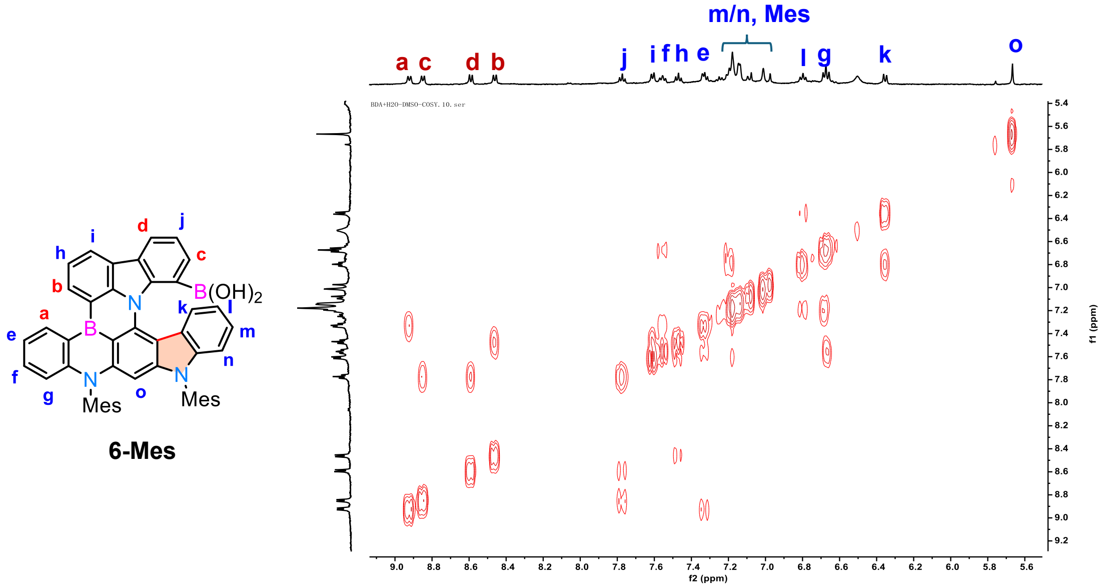

Fig. S5. The 2D ^{1}H-^{1}H COSY spectrum of **6-Mes** in DMSO-*d6* at 298K (500 MHz).

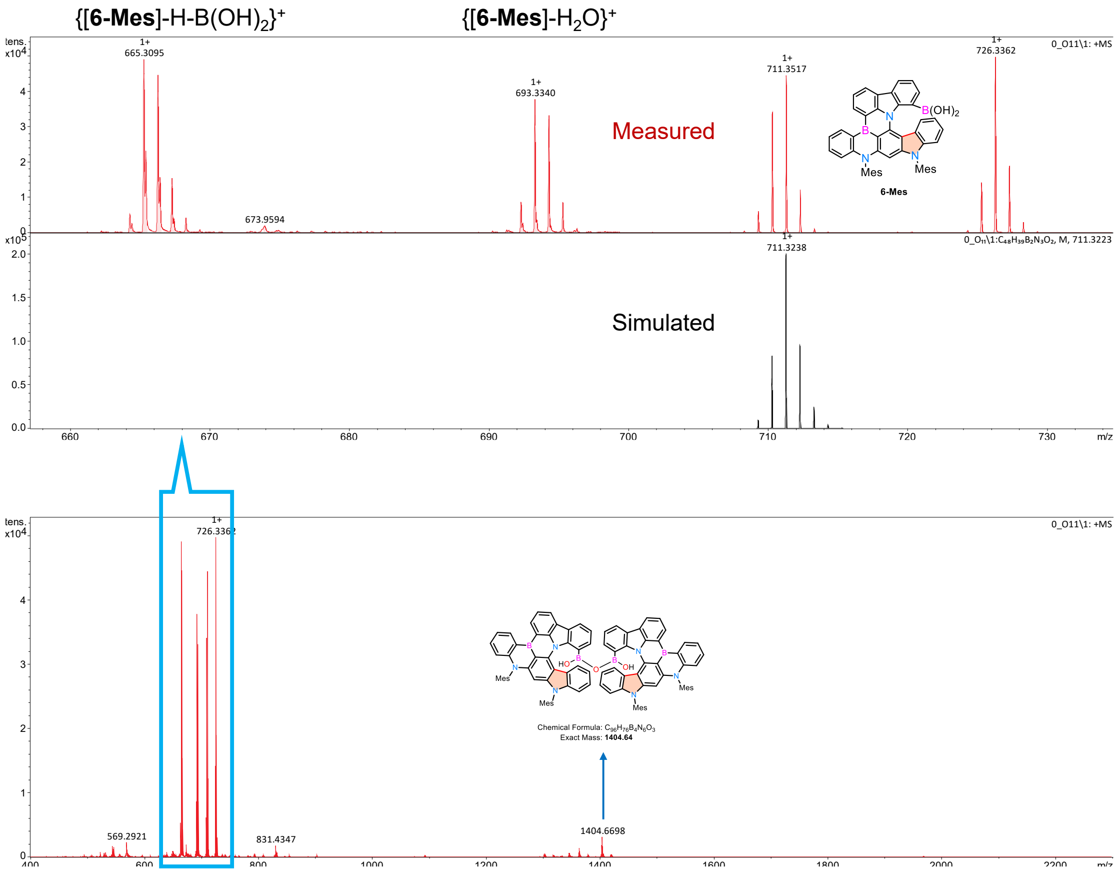

Fig. S6. Mass spectrum (MALDI-TOF) of **6-Mes**.

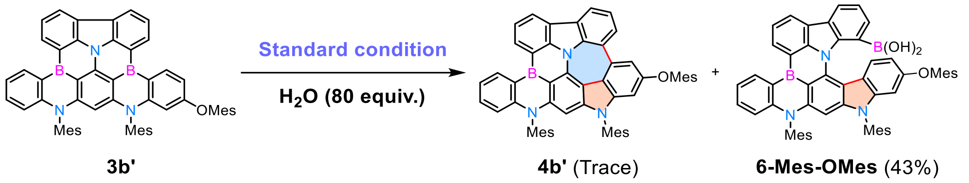

Compound **3b’** (41.0 mg, 0.05 mmol, 1.0 equiv.) and DDQ (34.0 mg, 0.15 mmol, 3.0 equiv.) were added to a 20 mL vial. The vial was then sealed and purged with nitrogen flow. Anhydrous DCM (1.5 mL) was injected via a syringe followed by 4 μl H_2O (80 equiv.). HOTf (150 uL) was finally injected via a micro-syringe with stainless needle. The vial was further sealed with electrical tape and stirred at room temperature for 12 h. The reaction mixture was poured into saturated Na_2CO_3 solution and extracted with DCM. The organic phase was dried over Na_2SO_4. After removal of solvent, the residue was purified by column chromatography using hexane/EA = 8:1-4:1 to give **6-Mes-OMes** (18.0 mg, 43% yield) as dark-green solid.

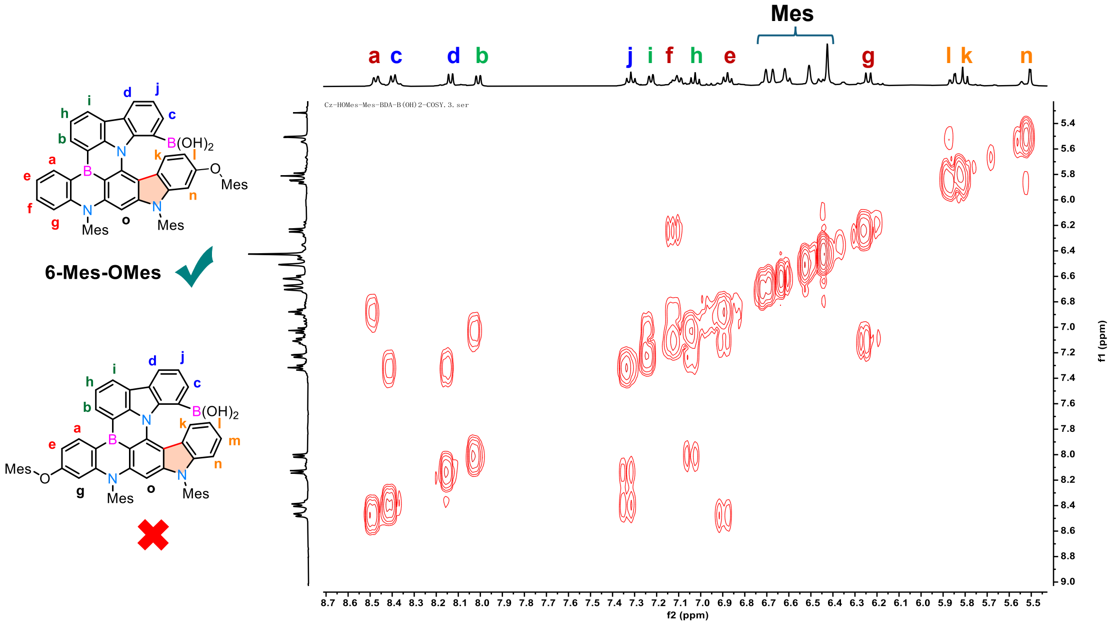

Fig. S7. The 2D ^1H-^1H COSY spectrum of **6-Mes-OMes** in Acetone-*d6* at 298K (400 MHz).

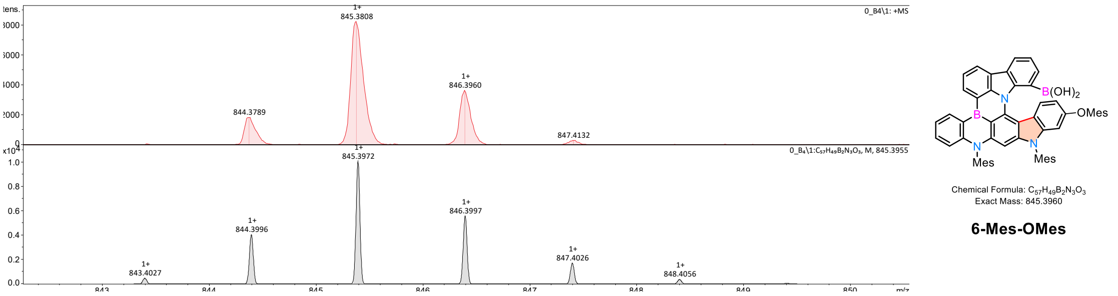

Fig. S8. Mass spectrum (MALDI-TOF) of **6-Mes-OMes**.

7. DFT calculation

7.1 Computational details and Cartesian coordinates

All structures considering the reaction mechanistic studies were fully optimized using Gaussian 16[S4] Optimizations were performed with the M06-2X functional developed by Truhlar and Zhao[S5]. Solvent effects were incorporated using the SMD solvation model [S6] with dichloromethane (ε = 8.93) as solvent to match experimental conditions. The def2-SVP basis set (BS1) [S7,8] was employed for all atoms during geometry optimizations and frequency calculations.

Transition states were located using the Berny algorithm, and their connectivity to minima was verified through Intrinsic Reaction Coordinate (IRC) calculations[S9]. To improve energy accuracy, single-point calculations were performed on all optimized structures using the M06-2X functional with the larger def2-TZVP basis set (BS2)[S7,8]. Tight convergence criteria and an ultrafine integration grid were employed to enhance computational precision.

The overall solvation free energy values reported in this work were obtained by adding the free energy corrections from the frequency analysis to the single-point solvation free energies calculated using the SMD solvation model. Moreover, the overall free energy values were corrected by adding $\Delta G^{1atm\rightarrow 1M}$ = 1.89 kcal/mol to account for the free energy change associated with compressing 1 mol of an ideal gas from 1 atm to the 1 M solution phase standard state.

Spin Natural Orbital (SNO) analysis was conducted using a Python-based SNO package[S10]. Natural Atomic Orbitals (NAOs) were obtained using the NBO 7.0 program[S11].

The time-dependent density functional theory (TD-DFT) calculation[S12] was conducted after the geometry optimization (B3LYP/6-31g(d) with Grimme's D3BJ dispersion correction[S13,14]) and employed the PBE0[S15,16] hybrid functional considered the solvent used during experiments (toluene) through the self-consistent reaction field (SCRF) method using the SMD variation of the polarizable continuum model (IEFPCM)[S17,18] with the 6-311G(d) basic set.

The visualizations were completed by using Multiwfn software[S19], VMD[S20], CYLview[S21].

Cartesian coordinates:

A

6 -4.475107 -2.192851 -1.444034
6 -3.290753 -2.874595 -1.154869
6 -3.113493 -4.272754 -1.091787
6 -4.192965 -5.072753 -1.430606
6 -5.404355 -4.445610 -1.771597
6 -5.550650 -3.059359 -1.762101
1 -4.112878 -6.160449 -1.423463
1 -6.263972 -5.064702 -2.032545
1 -6.535616 -2.654947 -1.991256
5 -4.406434 -0.648971 -1.206657
7 -2.097328 -2.257394 -0.803555
6 -1.191795 -3.232320 -0.377558
6 0.003334 -2.966918 0.287214
6 -1.761739 -4.501508 -0.570670
6 0.704476 -4.111091 0.718797
6 -1.024281 -5.606401 -0.160765
6 0.210071 -5.394073 0.472103
1 1.654081 -4.013748 1.246860
1 -1.402874 -6.618571 -0.307928
1 0.792780 -6.255109 0.801575
5 0.333623 -1.461718 0.462076
6 -1.919223 -0.931538 -0.715668
6 -3.038781 -0.096224 -0.766948
6 -0.554477 -0.440744 -0.447652
6 -2.877903 1.262943 -0.335904
6 -0.495765 0.998590 -0.131957
6 -1.604142 1.781258 0.058385
1 -1.478832 2.832419 0.302614
6 0.694742 -0.624397 -1.320105
6 1.499917 0.514545 -1.071851

6 1.157446 -1.654338 -2.148651
6 2.786180 0.617138 -1.587745
6 2.429954 -1.527539 -2.702075
1 0.522142 -2.511577 -2.383636
6 3.233565 -0.421340 -2.406263
1 3.411841 1.483792 -1.374407
1 2.798911 -2.307209 -3.368491
1 4.236021 -0.359002 -2.832904
6 -5.521519 0.424186 -1.279024
6 -6.815171 0.177837 -1.782778
6 -5.244463 1.730672 -0.805141
6 -7.803660 1.148847 -1.803782
1 -7.043202 -0.808369 -2.183737
6 -6.252711 2.715232 -0.805400
6 -7.514551 2.420210 -1.298814
1 -8.792096 0.924418 -2.206275
1 -6.056562 3.715865 -0.426605
1 -8.280124 3.198004 -1.294344
7 0.791779 1.438755 -0.300942
6 1.194645 2.784606 -0.049307
6 1.527668 3.624340 -1.113706
6 1.208342 3.251178 1.266465
6 1.890515 4.944498 -0.850590
1 1.495392 3.245128 -2.136618
6 1.557384 4.576612 1.516717
1 0.946829 2.572779 2.080872
6 1.902632 5.421658 0.460615
1 2.154385 5.605088 -1.677726
1 1.567493 4.947634 2.542538
1 2.181614 6.457307 0.660634
7 -3.958306 2.085027 -0.321876
6 -3.782330 3.423806 0.190371
6 -3.963651 3.659689 1.550774
6 -3.422016 4.450087 -0.681150
6 -3.777093 4.949648 2.047685
1 -4.247129 2.835507 2.207824
6 -3.236341 5.736046 -0.175973
1 -3.286766 4.233787 -1.742649
6 -3.413109 5.985373 1.186378
1 -3.916480 5.144175 3.112100
1 -2.951624 6.545556 -0.849589
1 -3.266938 6.993129 1.578275
16 2.605700 -1.476347 1.848264
8 3.315325 -1.911940 0.673223
8 2.479944 -2.301635 3.019156
8 1.181680 -0.886319 1.428153
6 3.317802 0.139585 2.394868
9 3.364206 0.973559 1.378333
9 4.532496 -0.091981 2.841565
9 2.572958 0.643620 3.354382
16 -1.269445 -4.607434 -4.102702
8 -0.182848 -4.791599 -5.064284
8 -1.692685 -5.811361 -3.384024
8 -1.176733 -3.378424 -3.295226
6 -2.720680 -4.233341 -5.177083
9 -2.475284 -3.183192 -5.957997
9 -3.799402 -3.954569 -4.448484
9 -3.014442 -5.266556 -5.963382

E (BS1) = -3748.97793897 au
H (BS1) = -3748.301097 au
G (BS1) = -3748.438493 au
E (BS2) = -3752.68920329 au
#............................

B

6 -4.550779 -1.806798 -1.038140
6 -3.355900 -2.483644 -1.293723
6 -3.341174 -3.745019 -1.909218
6 -4.526211 -4.337209 -2.339852
6 -5.728615 -3.658186 -2.134020
6 -5.734536 -2.420913 -1.486511
1 -4.510057 -5.313723 -2.827509
1 -6.666768 -4.099152 -2.473633
1 -6.686196 -1.906641 -1.339753
5 -4.368833 -0.434284 -0.377435
7 -2.048272 -2.060401 -0.977965
6 -1.201840 -3.179541 -1.256520
6 0.106703 -3.505734 -0.866714
6 -1.969902 -4.186835 -1.895778
6 0.629765 -4.746679 -1.281264
6 -1.429653 -5.405138 -2.307449
6 -0.099582 -5.674362 -2.026666
1 1.654314 -5.004932 -1.001742
1 -2.063981 -6.134912 -2.813795
1 0.360956 -6.613955 -2.333256
5 1.151526 -2.700667 -0.066750
6 -1.795216 -0.671452 -0.761721

6 -2.953475 0.132785 -0.482728
6 -0.534444 -0.005305 -0.899197
6 -2.879409 1.562616 -0.426947
6 -0.604451 1.415907 -1.053940
6 -1.718090 2.210624 -0.832802
1 -1.637711 3.290601 -0.912741
6 0.913127 -0.290038 -0.926502
6 1.572557 0.927980 -1.233374
6 1.733772 -1.361128 -0.526609
6 2.960926 1.053763 -1.330737
6 3.127316 -1.223701 -0.554515
6 3.739586 -0.046760 -0.999488
1 3.410765 2.004356 -1.620341
1 3.748099 -2.059838 -0.226394
1 4.827266 0.021416 -1.048751
6 -5.458876 0.444794 0.262943
6 -6.688758 -0.049880 0.742417
6 -5.190392 1.823843 0.406795
6 -7.638371 0.779223 1.320215
1 -6.884643 -1.121796 0.672733
6 -6.161923 2.675697 0.977272
6 -7.365528 2.149815 1.422045
1 -8.580521 0.374133 1.691499
1 -5.973039 3.742707 1.082538
1 -8.102370 2.821419 1.867156
7 0.638211 1.934027 -1.345294
6 0.919733 3.320515 -1.505306
6 0.444272 3.995335 -2.631982
6 1.645263 4.000270 -0.523585
6 0.690609 5.360483 -2.770565
1 -0.119175 3.445485 -3.388013
6 1.902257 5.361632 -0.678322
1 1.998992 3.458624 0.355778
6 1.422231 6.043147 -1.797704
1 0.315237 5.890598 -3.647246
1 2.470924 5.893892 0.085827
1 1.618554 7.110452 -1.911837
7 -3.969302 2.338847 -0.028987
6 -3.799931 3.765221 -0.022359
6 -3.225753 4.390377 1.084991
6 -4.200515 4.509885 -1.131155
6 -3.046775 5.773075 1.078012
1 -2.919486 3.786288 1.941308
6 -4.019961 5.893130 -1.132815
1 -4.646820 3.998244 -1.986024
6 -3.441725 6.524252 -0.030590
1 -2.596618 6.265214 1.941609
1 -4.330338 6.478671 -1.999695
1 -3.298628 7.606066 -0.034675
16 1.355400 -4.571753 1.875384
8 2.124381 -5.720599 1.477124
8 -0.074193 -4.612619 2.033095
8 1.810744 -3.290635 1.029955
6 2.066586 -3.968125 3.469592
9 3.368305 -3.825315 3.348926
9 1.798102 -4.866890 4.394071
9 1.515411 -2.817650 3.790430

E (BS1) = -2787.71395294 au
H (BS1) = -2787.087868 au
G (BS1) = -2787.204744 au
E (BS2) = -2790.54035731 au
#............................

B2

6 -4.385371 -1.915864 -1.132067
6 -3.162488 -2.550201 -1.328597
6 -3.004591 -3.739300 -2.056711
6 -4.119262 -4.337035 -2.623427
6 -5.374327 -3.733910 -2.435760
6 -5.505628 -2.555580 -1.707564
1 -4.024650 -5.254640 -3.206101
1 -6.258541 -4.194104 -2.878961
1 -6.492922 -2.102806 -1.605360
5 -4.279236 -0.533632 -0.464989
7 -1.897946 -2.093393 -0.874845
6 -0.969561 -3.150521 -1.132664
6 0.268093 -3.415004 -0.555489
6 -1.595516 -4.098402 -1.977634
6 0.984749 -4.490931 -1.115476
6 -0.893388 -5.184919 -2.467863
6 0.440079 -5.343694 -2.065422
1 2.006506 -4.668449 -0.772738
1 -1.372937 -5.903067 -3.134398
1 1.032339 -6.173037 -2.454983
5 0.898503 -2.616592 0.672606
6 -1.705068 -0.776334 -0.631605
6 -2.866545 0.037764 -0.455941

6 -0.391113 -0.101322 -0.705936
6 -2.764025 1.447315 -0.423336
6 -0.453369 1.317101 -1.033774
6 -1.538979 2.096193 -0.856261
1 -1.479821 3.172308 -1.013548
6 0.966856 -0.386553 -0.604419
6 1.694208 0.792954 -1.075240
6 1.728660 -1.465158 -0.025355
6 3.089153 0.791708 -1.225828
6 3.089037 -1.428548 -0.154358
6 3.754434 -0.333240 -0.790476
1 3.609875 1.651524 -1.644992
1 3.692437 -2.225302 0.284047
1 4.842295 -0.366137 -0.879503
6 -5.390408 0.391490 0.060757
6 -6.679082 -0.051115 0.417614
6 -5.080877 1.755459 0.263606
6 -7.624417 0.815055 0.943898
1 -6.925030 -1.107221 0.296194
6 -6.038468 2.642348 0.797137
6 -7.295397 2.165527 1.129180
1 -8.614683 0.450381 1.219646
1 -5.801229 3.691557 0.962243
1 -8.030590 2.854445 1.547827
7 0.826030 1.782847 -1.316861
6 1.126660 3.095608 -1.796148
6 0.663052 3.477726 -3.055883
6 1.860850 3.965177 -0.988715
6 0.940079 4.767025 -3.507503
1 0.122663 2.748501 -3.661584
6 2.140107 5.246821 -1.459409
1 2.199939 3.636921 -0.004475
6 1.677056 5.647747 -2.713803
1 0.586524 5.080580 -4.490800
1 2.714634 5.936306 -0.839346
1 1.893920 6.653947 -3.076010
7 -3.804693 2.240932 -0.086508
6 -3.602194 3.673827 -0.040782
6 -2.983964 4.232954 1.075099
6 -4.028372 4.455303 -1.111961
6 -2.782532 5.612390 1.112566
1 -2.666379 3.588624 1.897288
6 -3.820885 5.832812 -1.063417
1 -4.509478 3.983338 -1.970416
6 -3.199528 6.409621 0.045954
1 -2.297514 6.063380 1.979276
1 -4.143514 6.456405 -1.898244
1 -3.038221 7.488152 0.078330
16 1.419035 -4.718833 2.331868
8 0.090372 -5.174696 1.997441
8 1.840140 -4.587011 3.704644
8 1.814571 -3.428400 1.545115
6 2.617432 -5.948557 1.589804
9 1.997765 -6.706073 0.706684
9 3.081725 -6.708016 2.561508
9 3.626166 -5.328465 1.003328
16 -0.217327 -0.888006 2.517371
8 -0.238535 -2.092114 1.531466
8 0.966669 -0.067683 2.383180
8 -1.530130 -0.285494 2.531593
6 -0.032293 -1.775328 4.138216
9 -0.617760 -2.954538 4.084461
9 -0.600141 -1.043715 5.078618
9 1.246439 -1.922974 4.408180
16 -0.090227 -0.872936 -3.863981
8 0.811049 -1.911708 -3.363878
8 -1.490575 -0.972009 -3.428202
8 0.458094 0.492429 -3.845770
6 -0.204780 -1.248133 -5.663799
9 0.992361 -1.185747 -6.238822
9 -1.003075 -0.381322 -6.280717
9 -0.693141 -2.469219 -5.858278

E (BS1) = -4709.03393033 au
H (BS1) = -4708.329777 au
G (BS1) = -4708.484821 au
E (BS2) = -4713.61635939 au
#............................

B(OTf)$_3$

5 0.669636 -3.180257 0.512678
16 1.868554 -4.713242 2.396998
8 0.698901 -4.574225 3.217892
8 3.174439 -4.289967 2.803775
8 1.597704 -4.108163 0.910935
6 1.968591 -6.475957 1.836445
9 0.851338 -6.792213 1.222674
9 2.125770 -7.218779 2.907894

9 2.990892 -6.617466 1.027923
16 -0.453795 -1.110522 1.832740
8 -0.238513 -2.656701 1.403379
8 0.375833 -0.279907 1.002597
8 -1.846475 -0.897657 2.065715
6 0.406657 -1.264963 3.468701
9 -0.308486 -2.002850 4.277916
9 0.564415 -0.059444 3.961736
9 1.586381 -1.822889 3.258741
16 -0.373058 -2.101459 -1.746534
8 0.718882 -2.871418 -0.823779
8 -0.707016 -2.977148 -2.828141
8 -1.364423 -1.479717 -0.910890
6 0.777149 -0.797364 -2.415772
9 1.551989 -0.338430 -1.465748
9 0.022735 0.163473 -2.897359
9 1.498151 -1.335650 -3.370242

E (BS1) = -2906.91668621 au
H (BS1) = -2906.795011 au
G (BS1) = -2906.878615 au
E (BS2) = -2909.56108325 au
#............................

DCDHP

6 0.971366 -1.409596 1.817393
6 0.627162 -0.053786 1.976624
6 -0.675506 0.326710 2.286499
6 -1.693385 -0.631649 2.450535
6 -1.357190 -1.980219 2.292630
6 -0.036783 -2.365812 1.978886
8 -2.954544 -0.323062 2.746360
17 -1.089018 1.989489 2.480279
17 1.868205 1.126203 1.776391
6 -2.386810 -2.965969 2.455479
6 0.299382 -3.751346 1.817353
7 -3.212173 -3.761931 2.585383
7 0.564457 -4.866985 1.687991
8 2.201257 -1.827468 1.522622
1 2.815413 -1.081627 1.419634
1 -3.074935 0.636928 2.835479

E (BS1) = -1485.44007176 au
H (BS1) = -1485.338272 au
G (BS1) = -1485.391664 au
E (BS2) = -1486.39751381 au
#............................

DDQ

6 0.998866 -1.389290 1.738406
6 0.613182 0.048240 1.887643
6 -0.646555 0.415367 2.194042
6 -1.730315 -0.593067 2.403346
6 -1.348930 -2.040790 2.250422
6 -0.089085 -2.408223 1.942557
8 -2.855898 -0.284622 2.680474
17 -1.121376 2.043634 2.371975
17 1.872774 1.171536 1.643642
6 -2.404440 -2.992798 2.450595
6 0.300324 -3.781723 1.789630
7 -3.255577 -3.753462 2.611691
7 0.618839 -4.882508 1.663902
8 2.115850 -1.733938 1.469274

E (BS1) = -1484.17935495 au
H (BS1) = -1484.101224 au
G (BS1) = -1484.154308 au
E (BS2) = -1485.13639853 au
#............................

HOtf

16 9.448265 -1.109689 4.236947
8 10.721268 -1.475229 4.787936
8 8.787324 -1.854924 3.197819
8 8.460817 -0.866518 5.457193
6 9.600080 0.627091 3.628064
9 10.160033 1.375622 4.553456
9 8.397132 1.091398 3.347246
9 10.342223 0.628755 2.540800
1 7.523477 -0.910700 5.173017

E (BS1) = -961.246961621 au
H (BS1) = -961.198512 au
G (BS1) = -961.239896 au
E (BS2) = -962.130795096 au
#............................

P

6 -4.537145 -1.931814 -0.723000
6 -3.302842 -2.533200 -1.020497
6 -3.156521 -3.904293 -1.288083
6 -4.272795 -4.736474 -1.292925
6 -5.522982 -4.167390 -1.043806
6 -5.644817 -2.801672 -0.770490
1 -4.173341 -5.802888 -1.504199
1 -6.416776 -4.792390 -1.066545
1 -6.647193 -2.406422 -0.610995
5 -4.481380 -0.408208 -0.421586
7 -2.067187 -1.899931 -1.118607
6 -1.082781 -2.878882 -1.450063
6 0.319480 -2.819544 -1.679716
6 -1.752369 -4.127088 -1.547583
6 0.936042 -4.041063 -1.995769
6 -1.094976 -5.318190 -1.856258
6 0.268543 -5.264708 -2.082358
1 2.003744 -4.051529 -2.196728
1 -1.653832 -6.253197 -1.918148
1 0.830340 -6.165269 -2.332630
6 -1.961327 -0.512740 -0.987056
6 -3.121917 0.248476 -0.666542
6 -0.732766 0.130095 -1.159862
6 -3.006285 1.673880 -0.547995
6 -0.676660 1.536267 -1.028806
6 -1.782870 2.326891 -0.734810
1 -1.676268 3.402590 -0.635766
6 0.616329 -0.323107 -1.414494
6 1.429931 0.834876 -1.418486
6 1.170827 -1.593521 -1.633064
6 2.806497 0.777578 -1.640130
6 2.559034 -1.640668 -1.825149
6 3.351886 -0.485107 -1.833478
1 3.422144 1.676824 -1.656669
1 3.070358 -2.587542 -1.978290
1 4.425558 -0.585687 -1.999457
6 -5.608849 0.517416 0.093835
6 -6.860671 0.061055 0.562542
6 -5.376948 1.916501 0.106017
6 -7.866451 0.921561 0.969847
1 -7.037652 -1.011577 0.627540
6 -6.414753 2.799380 0.489178
6 -7.636985 2.302028 0.907990
1 -8.819490 0.532791 1.330503
1 -6.260270 3.876305 0.471111
1 -8.419592 3.002697 1.205628
7 0.630318 1.950662 -1.196263
6 1.084724 3.293757 -1.110093
6 0.533086 4.269838 -1.945654
6 2.074978 3.635057 -0.184259
6 0.967270 5.590357 -1.843319
1 -0.233483 3.988312 -2.669926
6 2.518321 4.954176 -0.104122
1 2.487395 2.865892 0.471112
6 1.963085 5.934102 -0.928021
1 0.531132 6.351949 -2.491767
1 3.293982 5.218472 0.616371
1 2.306586 6.967231 -0.856296
7 -4.132301 2.440334 -0.246180
6 -3.971707 3.868219 -0.237418
6 -3.616126 4.519903 0.943753
6 -4.154466 4.585789 -1.418874
6 -3.441103 5.903249 0.939535
1 -3.475806 3.936510 1.855900
6 -3.976376 5.969359 -1.418106
1 -4.431221 4.052744 -2.330522
6 -3.619181 6.627806 -0.240517
1 -3.162345 6.416740 1.861110
1 -4.118016 6.533979 -2.341065
1 -3.479174 7.710032 -0.241788

E (BS1) = -1802.20725642 au
H (BS1) = -1801.624511 au
G (BS1) = -1801.717513 au
E (BS2) = -1804.13439775 au
#............................

Pi

6 -4.807874 -1.927561 -1.480327
6 -3.634142 -2.683717 -1.391510
6 -3.601186 -4.085637 -1.487895
6 -4.750771 -4.773160 -1.856836
6 -5.912452 -4.033691 -2.105862
6 -5.943652 -2.654099 -1.898391
1 -4.744615 -5.859609 -1.959282
1 -6.816705 -4.546920 -2.435714
1 -6.883844 -2.132625 -2.075144
5 -4.677794 -0.482079 -0.934693

7 -2.348780 -2.195913 -1.120952
6 -1.499922 -3.307389 -0.936272
6 -0.144145 -3.386340 -0.581954
6 -2.260411 -4.488491 -1.117379
6 0.377075 -4.677526 -0.325501
6 -1.707052 -5.743471 -0.897916
6 -0.380544 -5.833977 -0.473950
1 1.429153 -4.780904 -0.047016
1 -2.310962 -6.640565 -1.044475
1 0.071132 -6.806879 -0.278763
5 0.945702 -2.356958 -0.860731
6 -2.126339 -0.839665 -0.957025
6 -3.242326 0.000589 -0.699984
6 -0.833340 -0.252166 -1.044215
6 -3.023981 1.357878 -0.291998
6 -0.679343 1.100663 -0.596665
6 -1.724386 1.886449 -0.159491
1 -1.541100 2.903254 0.172135
6 0.403184 -0.592262 -1.663564
6 1.310354 0.500313 -1.439461
6 0.876812 -1.726804 -2.420234
6 2.606873 0.489239 -1.917535
6 2.210678 -1.668262 -2.954804
1 0.136506 -2.364371 -2.919883
6 3.052446 -0.616932 -2.676100
1 3.271102 1.334973 -1.732336
1 2.546991 -2.505393 -3.567657
1 4.070068 -0.612817 -3.066696
6 -5.802146 0.494503 -0.517113
6 -7.173320 0.159088 -0.493125
6 -5.442661 1.796902 -0.093927
6 -8.155110 1.061021 -0.117828
1 -7.471760 -0.852857 -0.761037
6 -6.441930 2.726001 0.272840
6 -7.776656 2.356735 0.253614
1 -9.205170 0.766565 -0.109545
1 -6.176546 3.734864 0.581853
1 -8.532928 3.087976 0.544991
7 0.628791 1.501387 -0.786084
6 1.125423 2.822020 -0.559970
6 0.544841 3.897422 -1.238314
6 2.187712 3.024746 0.321861
6 1.023865 5.186227 -1.014114
1 -0.276108 3.719238 -1.935230
6 2.672468 4.316625 0.524616
1 2.630222 2.176269 0.843137
6 2.087987 5.397178 -0.135499
1 0.567923 6.028149 -1.537193
1 3.505159 4.476922 1.210911
1 2.464173 6.407437 0.032594
7 -4.096284 2.184785 -0.027229
6 -3.808202 3.548781 0.333511
6 -3.632747 3.887539 1.674101
6 -3.689242 4.508927 -0.670538
6 -3.329452 5.206240 2.012268
1 -3.731341 3.116110 2.440316
6 -3.383781 5.825240 -0.325742
1 -3.832017 4.215538 -1.712443
6 -3.202618 6.173480 1.013907
1 -3.190050 5.477739 3.059800
1 -3.287598 6.580941 -1.107027
1 -2.963052 7.203942 1.281239
16 2.364642 -2.422919 1.432988
8 3.609456 -3.120268 1.573759
8 1.157771 -2.821996 2.102119
8 2.123974 -2.187425 -0.140680
6 2.642236 -0.645254 1.854104
9 3.640857 -0.165462 1.144562
9 2.915983 -0.561370 3.135521
9 1.538042 0.023794 1.583142
16 -2.162707 -2.294347 -4.471851
8 -1.414317 -3.404359 -3.853543
8 -3.527318 -2.608562 -4.883078
8 -1.961943 -0.990430 -3.826320
6 -1.261922 -2.078770 -6.065984
9 0.023869 -1.805336 -5.841064
9 -1.774079 -1.078937 -6.776995
9 -1.321197 -3.181919 -6.805654

E (BS1) = -3748.96874149 au
H (BS1) = -3748.292258 au
G (BS1) = -3748.427736 au
E (BS2) = -3752.67889682 au
#............................

S

6 1.764982 2.991906 -1.345757
6 2.289979 1.712836 -1.574413

6 3.633718 1.405766 -1.895839
6 4.537781 2.457716 -1.993469
6 4.066642 3.760292 -1.775023
6 2.728377 4.019375 -1.465484
1 5.586478 2.280432 -2.238800
1 4.762701 4.596786 -1.853231
1 2.442336 5.060635 -1.323631
5 0.224913 3.018538 -1.028533
7 1.562063 0.537110 -1.537426
6 2.388183 -0.533406 -1.829940
6 1.978210 -1.871688 -1.896631
6 3.696321 -0.047599 -2.064931
6 3.019006 -2.757670 -2.256953
6 4.683158 -0.968181 -2.400211
6 4.325003 -2.320650 -2.496485
1 2.819650 -3.822663 -2.371728
1 5.709399 -0.650634 -2.593567
1 5.085762 -3.052890 -2.771042
5 0.456080 -2.103145 -1.580102
6 0.215521 0.450961 -1.297060
6 -0.475386 1.659059 -1.048990
6 -0.362631 -0.838703 -1.318312
6 -1.873237 1.534275 -0.818563
6 -1.763301 -0.892061 -1.079174
6 -2.502094 0.278436 -0.841248
1 -3.568530 0.210552 -0.666243
6 -0.355273 -3.420648 -1.486212
6 -1.756882 -3.347051 -1.258907
6 0.215045 -4.706832 -1.603920
6 -2.523194 -4.534011 -1.198092
6 -0.532849 -5.870232 -1.533080
1 1.291527 -4.789415 -1.745241
6 -1.915677 -5.770576 -1.336741
1 -3.598334 -4.488425 -1.035827
1 -0.054243 -6.845665 -1.627844
1 -2.529830 -6.671599 -1.283805
6 -0.707704 4.213075 -0.699466
6 -0.266211 5.549264 -0.582047
6 -2.093259 3.965020 -0.496997
6 -1.119888 6.601609 -0.296486
1 0.792547 5.764356 -0.715604
6 -2.966876 5.040130 -0.212192
6 -2.482273 6.333577 -0.116452
1 -0.738567 7.620019 -0.213257
1 -4.029866 4.861722 -0.062355
1 -3.177846 7.145147 0.105666
7 -2.407967 -2.118622 -1.084852
6 -3.829968 -2.114430 -0.869915
6 -4.332151 -2.179249 0.429697
6 -4.689335 -1.998438 -1.961724
6 -5.709925 -2.122004 0.636814
1 -3.638245 -2.264136 1.268256
6 -6.066553 -1.942409 -1.748764
1 -4.271374 -1.944241 -2.968730
6 -6.576760 -2.001354 -0.450882
1 -6.107001 -2.167979 1.652165
1 -6.742714 -1.848777 -2.600067
1 -7.654451 -1.952811 -0.285941
7 -2.625435 2.670911 -0.569333
6 -4.036634 2.491082 -0.357860
6 -4.904210 2.513523 -1.449270
6 -4.515596 2.249548 0.929501
6 -6.265570 2.286207 -1.249010
1 -4.503897 2.698777 -2.447864
6 -5.877870 2.023541 1.123964
1 -3.815418 2.231892 1.766967
6 -6.752149 2.038916 0.035708
1 -6.947476 2.298571 -2.100832
1 -6.256545 1.830833 2.129062
1 -7.817151 1.856906 0.189584

E (BS1) = -1827.66562250 au
H (BS1) = -1827.066092 au
G (BS1) = -1827.161931 au
E (BS2) = -1829.61912942 au
#............................

TS1

6 1.990883 2.389827 -0.651286
6 2.410267 1.074500 -0.885215
6 3.744277 0.642415 -1.052716
6 4.753174 1.591341 -0.962485
6 4.391740 2.924012 -0.709373
6 3.058849 3.312379 -0.559680
1 5.800503 1.317097 -1.098488
1 5.172464 3.683330 -0.645077
1 2.863661 4.368570 -0.374441
5 0.434614 2.579311 -0.630461

7 1.563875 -0.014204 -1.058835
6 2.324890 -1.148671 -1.367110
6 1.803078 -2.404790 -1.675081
6 3.687112 -0.788728 -1.371887
6 2.781535 -3.352685 -2.029925
6 4.619479 -1.766463 -1.704843
6 4.147261 -3.043671 -2.035098
1 2.481042 -4.354990 -2.338784
1 5.687309 -1.543756 -1.729934
1 4.863470 -3.816091 -2.319654
5 0.221408 -2.502063 -1.639273
6 0.216021 0.028587 -0.998837
6 -0.382648 1.303120 -0.795609
6 -0.485038 -1.188711 -1.188055
6 -1.788457 1.321401 -0.792539
6 -1.873321 -1.145831 -1.045005
6 -2.529716 0.102114 -0.847382
1 -3.609469 0.127747 -0.752075
6 -0.662197 -3.757125 -1.334882
6 -2.058953 -3.579431 -1.155322
6 -0.159599 -5.068319 -1.257356
6 -2.900410 -4.699609 -0.979162
6 -0.980624 -6.169722 -1.061515
1 0.915046 -5.223171 -1.356964
6 -2.361286 -5.975132 -0.936924
1 -3.974812 -4.571909 -0.859280
1 -0.558170 -7.174293 -1.013845
1 -3.026140 -6.828527 -0.792352
6 -0.426411 3.861637 -0.556672
6 0.113078 5.164994 -0.464337
6 -1.844724 3.753048 -0.653579
6 -0.675992 6.300507 -0.474903
1 1.193069 5.282770 -0.390964
6 -2.650248 4.918030 -0.690579
6 -2.068859 6.166219 -0.599085
1 -0.221677 7.290303 -0.412461
1 -3.729833 4.839857 -0.801422
1 -2.703610 7.052998 -0.634181
7 -2.631922 -2.293291 -1.127910
6 -4.066804 -2.171698 -1.087235
6 -4.721840 -2.084616 0.141641
6 -4.778895 -2.115027 -2.284535
6 -6.106577 -1.924193 0.169128
1 -4.140837 -2.134189 1.064809
6 -6.164955 -1.960978 -2.248880
1 -4.243790 -2.188895 -3.232081
6 -6.827621 -1.861656 -1.024877
1 -6.623380 -1.848029 1.127005
1 -6.727173 -1.914908 -3.182799
1 -7.911258 -1.735358 -1.000492
7 -2.476048 2.506283 -0.729440
6 -3.920323 2.451091 -0.744279
6 -4.581707 2.197549 -1.944926
6 -4.619494 2.592918 0.452720
6 -5.971703 2.086534 -1.943818
1 -4.002839 2.061792 -2.860021
6 -6.009472 2.485175 0.442546
1 -4.073630 2.781097 1.379087
6 -6.684578 2.231743 -0.752779
1 -6.496798 1.878554 -2.877259
1 -6.565348 2.592604 1.375021
1 -7.771982 2.141833 -0.755028
6 -0.138648 3.386426 -3.910624
6 -1.035799 4.515031 -3.920806
6 -0.576856 5.792293 -3.758752
6 0.848633 6.083729 -3.528911
6 1.733358 4.921907 -3.570268
6 1.257322 3.631900 -3.778378
8 1.266413 7.216377 -3.324061
17 -1.619977 7.142799 -3.810180
17 -2.706483 4.188325 -4.155764
6 3.128102 5.173680 -3.381311
6 2.180073 2.535807 -3.873340
7 4.252724 5.377064 -3.212467
7 2.936907 1.670552 -3.978142
8 -0.665204 2.208602 -4.029918
16 -0.058465 -1.276110 -4.549538
8 0.498313 -1.560134 -5.855628
8 -0.164594 -2.439631 -3.634475
8 0.446376 -0.057320 -3.873542
6 -1.825147 -0.850356 -4.881610
9 -1.891910 0.086869 -5.813709
9 -2.405446 -0.386445 -3.778964
9 -2.490752 -1.917185 -5.292782
1 -0.067623 1.385170 -3.981170

E (BS1) = -4273.12886473 au
H (BS1) = -4272.402804 au

G (BS1) = -4272.547181 au
E (BS2) = -4276.90462497 au
#............................

TS2

6 -5.009043 -2.336246 -0.561389
6 -3.812066 -2.997225 -0.875619
6 -3.659115 -4.381947 -1.120013
6 -4.799840 -5.170563 -1.098735
6 -6.036223 -4.549897 -0.845135
6 -6.140902 -3.183407 -0.581446
1 -4.747916 -6.245344 -1.279953
1 -6.941719 -5.158486 -0.843253
1 -7.135338 -2.778382 -0.394334
5 -4.837612 -0.822244 -0.199557
7 -2.570048 -2.392060 -0.953159
6 -1.590850 -3.362608 -1.226624
6 -0.224819 -3.112690 -1.323967
6 -2.222338 -4.617461 -1.323788
6 0.548711 -4.270347 -1.516961
6 -1.417096 -5.734966 -1.530484
6 -0.032520 -5.542301 -1.621048
1 1.636776 -4.188265 -1.569582
1 -1.845904 -6.735184 -1.608200
1 0.612835 -6.409365 -1.771328
5 0.245368 -1.589274 -1.108283
6 -2.343632 -1.070089 -0.775789
6 -3.434946 -0.263192 -0.382927
6 -1.010186 -0.613825 -0.943918
6 -3.132392 1.099752 -0.160152
6 -0.796938 0.778765 -0.878987
6 -1.835663 1.620745 -0.463803
1 -1.653081 2.686020 -0.371225
6 1.262029 -0.857254 -2.096066
6 1.371326 0.550194 -1.990749
6 2.121751 -1.518503 -2.982396
6 2.384661 1.232334 -2.685889
6 3.106441 -0.843979 -3.701052
1 2.025094 -2.600417 -3.097414
6 3.244086 0.533892 -3.528758
1 2.495256 2.311367 -2.585488
1 3.770446 -1.386929 -4.375350
1 4.020307 1.081277 -4.066908
6 -5.831887 0.191215 0.418883
6 -7.142244 -0.140402 0.823415
6 -5.387420 1.520416 0.676947
6 -7.984590 0.771848 1.439730
1 -7.507115 -1.156084 0.667107
6 -6.242744 2.447640 1.321884
6 -7.520829 2.072717 1.691046
1 -8.987390 0.467647 1.739706
1 -5.897017 3.454944 1.546941
1 -8.163602 2.793371 2.199107
7 0.452219 1.286268 -1.200893
6 0.729583 2.651714 -0.859107
6 0.369073 3.690598 -1.720379
6 1.315999 2.925189 0.377102
6 0.594923 5.011840 -1.336246
1 -0.094390 3.455564 -2.680948
6 1.542113 4.248969 0.755078
1 1.579540 2.093464 1.034040
6 1.180213 5.291095 -0.099434
1 0.310330 5.826358 -2.004269
1 1.999758 4.465435 1.721710
1 1.353988 6.326276 0.199157
7 -4.092538 1.941803 0.331318
6 -3.756410 3.341594 0.476944
6 -2.963380 3.754125 1.545427
6 -4.192666 4.242176 -0.492874
6 -2.602521 5.097427 1.643342
1 -2.617320 3.024741 2.278658
6 -3.830630 5.584009 -0.383558
1 -4.806075 3.887905 -1.323433
6 -3.036180 6.011102 0.681828
1 -1.977215 5.427104 2.474264
1 -4.167120 6.296298 -1.138169
1 -2.751272 7.061357 0.761424
16 0.525494 -1.702535 1.713356
8 -0.927838 -1.504421 1.774292
8 1.355433 -1.045391 2.691664
8 1.067150 -1.518036 0.308121
6 0.711505 -3.529233 2.018496
9 -0.352988 -4.175357 1.581621
9 0.830557 -3.719985 3.318625
9 1.786809 -3.983405 1.407604
6 -4.010362 -0.678134 3.151378
6 -4.451318 -2.018762 2.890537
6 -5.761167 -2.373486 3.106080

6 -6.730824 -1.427914 3.630740
6 -6.251701 -0.115058 3.989104
6 -4.940426 0.244057 3.737401
8 -7.925407 -1.809857 3.763478
17 -6.324722 -3.939959 2.759694
17 -3.307001 -3.151320 2.317263
6 -7.138571 0.810707 4.630277
6 -4.467019 1.553767 4.083229
7 -7.823201 1.569838 5.164771
7 -4.100399 2.607885 4.375011
8 -2.823787 -0.235099 2.910974
1 -2.137023 -0.851097 2.492016
16 -11.033668 -0.859647 3.329804
8 -12.120497 0.093218 3.389220
8 -10.576553 -1.338900 2.033071
8 -9.880200 -0.472520 4.235455
6 -11.663161 -2.365993 4.179929
9 -12.075868 -2.070693 5.401369
9 -10.696404 -3.269867 4.262877
9 -12.673706 -2.880335 3.498224
1 -8.795229 -1.116397 4.024942

E (BS1) = -5234.39559043 au
H (BS1) = -5233.623596 au
G (BS1) = -5233.787662 au
E (BS2) = -5239.04760029 au
#............................

TS3

6 -4.827817 -1.961473 -1.461471
6 -3.644628 -2.705635 -1.398071
6 -3.577871 -4.107485 -1.503500
6 -4.724986 -4.811634 -1.849794
6 -5.905114 -4.089386 -2.063434
6 -5.959171 -2.710167 -1.852456
1 -4.705553 -5.897094 -1.960615
1 -6.808273 -4.617898 -2.371551
1 -6.915120 -2.210652 -2.006081
5 -4.697613 -0.502781 -0.934080
7 -2.368516 -2.196112 -1.149360
6 -1.492261 -3.272870 -0.982454
6 -0.135860 -3.279707 -0.630296
6 -2.214815 -4.477948 -1.166334
6 0.450568 -4.548890 -0.404209
6 -1.597011 -5.704745 -0.969452
6 -0.256391 -5.733603 -0.568715
1 1.508666 -4.607618 -0.135223
1 -2.154849 -6.631490 -1.114313
1 0.242935 -6.687979 -0.399746
5 0.841511 -2.117980 -0.783982
6 -2.147799 -0.845356 -0.977186
6 -3.262522 -0.008872 -0.707868
6 -0.857533 -0.276875 -1.062509
6 -3.034413 1.339466 -0.290919
6 -0.688626 1.058037 -0.596515
6 -1.727810 1.851110 -0.150948
1 -1.534347 2.861103 0.196198
6 0.404257 -0.642705 -1.680536
6 1.308051 0.462413 -1.448120
6 0.827640 -1.680509 -2.583033
6 2.611354 0.462030 -1.931877
6 2.152042 -1.652576 -3.057147
1 0.101186 -2.387860 -2.990114
6 3.024364 -0.619730 -2.714946
1 3.282658 1.296586 -1.728497
1 2.480422 -2.454750 -3.717916
1 4.044559 -0.632872 -3.100141
6 -5.814781 0.485629 -0.526525
6 -7.189614 0.162206 -0.519541
6 -5.449744 1.788431 -0.103462
6 -8.169203 1.071556 -0.158889
1 -7.492117 -0.847316 -0.791926
6 -6.448394 2.724511 0.249963
6 -7.784995 2.365204 0.215669
1 -9.221605 0.785562 -0.164654
1 -6.179355 3.732352 0.559337
1 -8.539224 3.102812 0.496198
7 0.632104 1.446597 -0.786992
6 1.133107 2.767293 -0.550149
6 0.539617 3.844239 -1.214451
6 2.206928 2.966289 0.317339
6 1.016622 5.133072 -0.987383
1 -0.288379 3.668131 -1.903348
6 2.687734 4.259082 0.523880
1 2.663475 2.117179 0.823822
6 2.090106 5.342055 -0.119852
1 0.551104 5.976388 -1.499561
1 3.529100 4.417288 1.199899

1 2.463941 6.352604 0.051495
7 -4.104213 2.172131 -0.023934
6 -3.808765 3.533030 0.339792
6 -3.642308 3.870435 1.681945
6 -3.671219 4.492583 -0.662758
6 -3.330280 5.186372 2.023252
1 -3.755136 3.099926 2.447103
6 -3.356956 5.806018 -0.315031
1 -3.807213 4.200740 -1.706040
6 -3.185392 6.152552 1.026378
1 -3.198414 5.456537 3.072112
1 -3.246853 6.560949 -1.095243
1 -2.939295 7.180839 1.296168
16 2.344120 -2.359979 1.438916
8 3.548463 -3.137315 1.478965
8 1.141919 -2.733590 2.131259
8 2.045640 -1.992613 -0.094523
6 2.747002 -0.631538 1.950838
9 3.741632 -0.171807 1.222315
9 3.078104 -0.634542 3.220963
9 1.675189 0.115314 1.762923
16 -2.218009 -2.299009 -4.502935
8 -1.473091 -3.422935 -3.906688
8 -3.592696 -2.593436 -4.894475
8 -1.991794 -1.003064 -3.848790
6 -1.340410 -2.077855 -6.109204
9 -0.048982 -1.817668 -5.901610
9 -1.854292 -1.067406 -6.803698
9 -1.420890 -3.174035 -6.857100

E (BS1) = -3748.96802509 au
H (BS1) = -3748.292485 au
G (BS1) = -3748.427412 au
E (BS2) = -3752.67826309 au
#............................

TS4

6 -4.674212 -1.913915 -1.644041
6 -3.519694 -2.692691 -1.512524
6 -3.534598 -4.093625 -1.628734
6 -4.678135 -4.740101 -2.084986
6 -5.798896 -3.964923 -2.396706
6 -5.801992 -2.590428 -2.152473
1 -4.695003 -5.824885 -2.203963
1 -6.694054 -4.443839 -2.795756
1 -6.713503 -2.030043 -2.361949
5 -4.549980 -0.513023 -1.004745
7 -2.238451 -2.247756 -1.153508
6 -1.453134 -3.403647 -0.917705
6 -0.132134 -3.556829 -0.467844
6 -2.242095 -4.549534 -1.173397
6 0.302664 -4.867623 -0.179919
6 -1.764271 -5.837353 -0.933288
6 -0.490930 -5.994487 -0.401192
1 1.324457 -5.019177 0.176527
1 -2.397777 -6.700424 -1.145175
1 -0.099610 -6.987499 -0.178784
5 1.058246 -2.641965 -0.747736
6 -1.984745 -0.881729 -0.957408
6 -3.117454 -0.045140 -0.723774
6 -0.692634 -0.280866 -1.028706
6 -2.936576 1.322568 -0.331352
6 -0.601442 1.106770 -0.684187
6 -1.658186 1.886854 -0.255877
1 -1.490398 2.921944 0.023021
6 0.603946 -0.617107 -1.570461
6 1.409017 0.560355 -1.497537
6 1.208634 -1.810701 -2.086472
6 2.695309 0.616319 -2.010973
6 2.536071 -1.713640 -2.609921
1 0.431609 -2.585601 -2.887552
6 3.245444 -0.530416 -2.610912
1 3.269908 1.542594 -1.953934
1 3.001778 -2.626378 -2.988828
1 4.248907 -0.483472 -3.034316
6 -5.695046 0.420112 -0.548937
6 -7.054779 0.042524 -0.507697
6 -5.362036 1.716960 -0.092159
6 -8.053406 0.904917 -0.085201
1 -7.328188 -0.972023 -0.795800
6 -6.378577 2.606348 0.323131
6 -7.702707 2.199211 0.318139
1 -9.094378 0.580435 -0.062174
1 -6.133880 3.611730 0.660033
1 -8.472944 2.898871 0.648264
7 0.673338 1.571146 -0.921804
6 1.127831 2.905652 -0.712470
6 0.533166 3.964086 -1.404220

6 2.172549 3.136749 0.184796
6 0.980857 5.264622 -1.179683
1 -0.275607 3.763122 -2.109200
6 2.627003 4.439167 0.387421
1 2.619952 2.295229 0.716378
6 2.028134 5.503060 -0.288010
1 0.513785 6.094523 -1.712173
1 3.445604 4.622445 1.084981
1 2.379664 6.522291 -0.120372
7 -4.026879 2.131571 -0.049074
6 -3.759984 3.499679 0.306543
6 -3.537176 3.837186 1.640746
6 -3.707827 4.469142 -0.694539
6 -3.254603 5.161494 1.974838
1 -3.583261 3.059249 2.405383
6 -3.422758 5.791318 -0.354594
1 -3.885297 4.177923 -1.731731
6 -3.194688 6.137188 0.978480
1 -3.078838 5.430863 3.017468
1 -3.378488 6.553457 -1.134340
1 -2.970811 7.172124 1.242199
16 2.294344 -2.605327 1.669339
8 3.534665 -3.234272 2.022947
8 1.040326 -2.952271 2.280959
8 2.204079 -2.585840 0.074998
6 2.502738 -0.778180 1.884635
9 3.538083 -0.357608 1.190361
9 2.685006 -0.528316 3.162406
9 1.409710 -0.171544 1.460343
16 -1.331775 -2.566927 -4.415578
8 -0.248544 -3.306340 -3.638235
8 -2.454049 -3.423369 -4.732539
8 -1.568228 -1.227676 -3.902596
6 -0.448611 -2.294413 -6.008309
9 0.687297 -1.648919 -5.782053
9 -1.197983 -1.569036 -6.822677
9 -0.174166 -3.448757 -6.593163

E (BS1) = -3748.94971648 au
H (BS1) = -3748.279070 au
G (BS1) = -3748.415090 au
E (BS2) = -3752.65394172 au
#............................

TS5

6 -4.690305 -1.726236 -0.597910
6 -3.519399 -2.414758 -0.908357
6 -3.516093 -3.659470 -1.557550
6 -4.718399 -4.247508 -1.925917
6 -5.914140 -3.578773 -1.628153
6 -5.898745 -2.347062 -0.978870
1 -4.730793 -5.210042 -2.439949
1 -6.866280 -4.027039 -1.915141
1 -6.845292 -1.839866 -0.785670
5 -4.458031 -0.326555 -0.014914
7 -2.192532 -1.980833 -0.670541
6 -1.345130 -3.097352 -0.999410
6 -0.027197 -3.382260 -0.642748
6 -2.129772 -4.074215 -1.661095
6 0.536427 -4.525744 -1.238647
6 -1.563317 -5.225889 -2.189205
6 -0.191781 -5.417803 -2.019864
1 1.584996 -4.747208 -1.038146
1 -2.182886 -5.960098 -2.706320
1 0.297567 -6.296187 -2.442960
5 0.863039 -2.537385 0.373586
6 -1.922442 -0.633963 -0.513446
6 -3.042939 0.210291 -0.204153
6 -0.639501 0.003429 -0.786254
6 -2.927988 1.627926 -0.245059
6 -0.708196 1.408309 -1.088093
6 -1.772238 2.229066 -0.807876
1 -1.702836 3.298336 -0.991277
6 0.755322 -0.326060 -0.922927
6 1.411548 0.827864 -1.458085
6 1.571706 -1.392727 -0.456055
6 2.779038 0.844563 -1.767762
6 2.924046 -1.358494 -0.730888
6 3.515424 -0.273210 -1.422037
1 3.239424 1.717396 -2.230247
1 3.561667 -2.165128 -0.363562
1 4.585956 -0.293189 -1.634451
6 -5.495469 0.632378 0.598238
6 -6.722595 0.210606 1.146254
6 -5.174235 2.009727 0.663342
6 -7.602014 1.104544 1.737661
1 -6.963184 -0.854918 1.141928
6 -6.087830 2.932139 1.225846

6 -7.281165 2.473099 1.756341
1 -8.532790 0.766923 2.195049
1 -5.851438 3.993511 1.273455
1 -7.975372 3.173235 2.224637
7 0.511100 1.850513 -1.562876
6 0.797967 3.192485 -1.949592
6 0.115769 3.758575 -3.028554
6 1.742377 3.929270 -1.231149
6 0.379324 5.080127 -3.384980
1 -0.613330 3.161613 -3.579499
6 2.010514 5.244209 -1.606789
1 2.253929 3.470356 -0.383180
6 1.327754 5.821197 -2.678707
1 -0.152906 5.528592 -4.225091
1 2.749573 5.823230 -1.051117
1 1.535763 6.853375 -2.964678
7 -3.942964 2.455582 0.171965
6 -3.715407 3.881307 0.132480
6 -2.948760 4.475103 1.132758
6 -4.257912 4.631340 -0.908953
6 -2.721862 5.850208 1.087069
1 -2.536448 3.856485 1.931945
6 -4.027320 6.005868 -0.944970
1 -4.852726 4.136611 -1.679010
6 -3.261015 6.614076 0.050977
1 -2.121404 6.324658 1.864603
1 -4.446689 6.602591 -1.756209
1 -3.081887 7.689838 0.018402
16 1.769589 -4.611714 1.975201
8 0.657850 -5.441907 1.578926
8 1.990032 -4.310800 3.368788
8 1.921285 -3.331889 1.097580
6 3.320707 -5.452699 1.433091
9 3.301253 -5.700700 0.135286
9 3.422945 -6.592343 2.090700
9 4.360068 -4.689153 1.711049
6 -4.226404 1.057646 3.571849
6 -5.046912 2.067918 4.178314
6 -6.231726 1.753465 4.797338
6 -6.719782 0.389535 4.812444
6 -5.866956 -0.623450 4.231930
6 -4.656270 -0.305199 3.640555
8 -7.814106 0.027599 5.317574
17 -7.163316 2.943104 5.582190
17 -4.479482 3.679101 4.128625
6 -6.353968 -1.970785 4.238985
6 -3.868972 -1.331706 3.016571
7 -6.745257 -3.056124 4.231590
7 -3.249550 -2.149843 2.487885
8 -3.131444 1.448998 3.000903
16 0.135125 -0.741657 2.369582
8 -0.079065 -1.964414 1.456750
8 1.289378 0.063182 2.057473
8 -1.150841 -0.089891 2.569119
6 0.476389 -1.529460 4.012302
9 -0.211531 -2.642155 4.136679
9 0.103954 -0.674919 4.949192
9 1.763620 -1.758916 4.121690
1 -2.470876 0.739531 2.757436
16 -10.523843 1.880803 4.889545
8 -11.477320 2.857143 5.363654
8 -9.666983 2.201654 3.757854
8 -9.745546 1.242465 6.029170
6 -11.524451 0.449588 4.305528
9 -12.295531 -0.007627 5.276210
9 -10.711218 -0.520968 3.908127
9 -12.284123 0.815678 3.286987
1 -8.703156 0.722082 5.683045

E (BS1) = -6194.48858591 au
H (BS1) = -6193.689312 au
G (BS1) = -6193.870505 au
E (BS2) = -6200.01083834 au
#............................

TS6

6 -4.315382 -1.977494 -1.010991
6 -3.072185 -2.581787 -1.182638
6 -2.868453 -3.781935 -1.878055
6 -3.958440 -4.436102 -2.433594
6 -5.230305 -3.865241 -2.274733
6 -5.404921 -2.668304 -1.582648
1 -3.829724 -5.366487 -2.988630
1 -6.096342 -4.362470 -2.713673
1 -6.407479 -2.244536 -1.508485
5 -4.265921 -0.577263 -0.364225
7 -1.829960 -2.076559 -0.725587
6 -0.863847 -3.060918 -0.980457

6 0.409319 -3.266831 -0.428331
6 -1.446509 -4.074190 -1.791814
6 1.196617 -4.298062 -1.008395
6 -0.689102 -5.120284 -2.273297
6 0.671833 -5.200405 -1.905979
1 2.239403 -4.392617 -0.697740
1 -1.136977 -5.872068 -2.924903
1 1.293685 -6.006234 -2.297204
5 0.944798 -2.526522 0.880245
6 -1.693934 -0.735171 -0.485169
6 -2.871745 0.038425 -0.330215
6 -0.412829 -0.049730 -0.554079
6 -2.794380 1.459349 -0.290221
6 -0.466915 1.380841 -0.828355
6 -1.587925 2.133992 -0.678161
1 -1.554511 3.209772 -0.834800
6 0.939535 -0.379168 -0.545342
6 1.670607 0.783913 -1.016102
6 1.694880 -1.496942 -0.045716
6 3.023274 0.726769 -1.263751
6 3.057456 -1.562521 -0.350705
6 3.690671 -0.476101 -0.947802
1 3.563414 1.582778 -1.667179
1 3.648962 -2.405948 0.008254
1 4.771064 -0.518343 -1.100785
6 -5.414658 0.315339 0.135338
6 -6.697313 -0.165195 0.465702
6 -5.146524 1.687622 0.343863
6 -7.681666 0.670734 0.969002
1 -6.907955 -1.228889 0.342240
6 -6.144722 2.542644 0.853357
6 -7.395695 2.029575 1.157080
1 -8.666182 0.276372 1.223490
1 -5.943962 3.598856 1.022115
1 -8.159616 2.698655 1.556517
7 0.795519 1.824294 -1.146376
6 1.116325 3.134509 -1.616325
6 1.422831 3.312533 -2.966285
6 1.114367 4.206190 -0.722158
6 1.741881 4.591189 -3.421496
1 1.388466 2.445711 -3.629027
6 1.418212 5.482601 -1.193910
1 0.875925 4.033588 0.329161
6 1.736043 5.673462 -2.539601
1 1.986158 4.743558 -4.473926
1 1.414633 6.328620 -0.504972
1 1.979797 6.672947 -2.903529
7 -3.876977 2.214723 0.030362
6 -3.712493 3.648675 0.099844
6 -3.137749 4.208444 1.239208
6 -4.119312 4.437539 -0.973526
6 -2.961154 5.590314 1.298787
1 -2.830474 3.559837 2.062048
6 -3.939797 5.818515 -0.903269
1 -4.564353 3.967683 -1.852396
6 -3.360866 6.393401 0.229427
1 -2.510003 6.039513 2.184686
1 -4.249582 6.446062 -1.740080
1 -3.219691 7.474187 0.278605
16 1.506315 -4.617831 2.481437
8 0.198224 -5.086117 2.089505
8 1.885512 -4.500377 3.865622
8 1.888214 -3.293328 1.727450
6 2.754758 -5.793001 1.732907
9 2.169942 -6.535997 0.815641
9 3.216234 -6.565924 2.693174
9 3.755881 -5.128232 1.186230
16 -0.087115 -0.750066 2.644588
8 -0.179521 -1.988306 1.685497
8 1.134350 -0.002648 2.447557
8 -1.371933 -0.095857 2.665303
6 0.093755 -1.615785 4.281560
9 -0.517094 -2.782343 4.247684
9 -0.451120 -0.856821 5.211364
9 1.370933 -1.786968 4.540054
16 -0.028057 -0.972124 -3.735745
8 0.956499 -1.887333 -3.150363
8 -1.426267 -1.216046 -3.357879
8 0.363792 0.443716 -3.755024
6 -0.008014 -1.437697 -5.517710
9 1.204700 -1.274047 -6.038448
9 -0.859585 -0.692561 -6.215767
9 -0.353402 -2.712892 -5.671749

E (BS1) = -4709.03012051 au
H (BS1) = -4708.327753 au
G (BS1) = -4708.481967 au
E (BS2) = -4713.60956613 au

#............................

V1

6 1.764714 3.053142 -1.294129
6 2.301336 1.776169 -1.503485
6 3.629240 1.479172 -1.889554
6 4.504607 2.543393 -2.076582
6 4.020067 3.845489 -1.883316
6 2.696058 4.093784 -1.510451
1 5.540984 2.376004 -2.375124
1 4.693840 4.689858 -2.036185
1 2.390976 5.134118 -1.401806
5 0.242909 3.065859 -0.907875
7 1.597398 0.590245 -1.382634
6 2.424937 -0.476507 -1.691403
6 2.029896 -1.819912 -1.711058
6 3.706343 0.021837 -2.022060
6 3.051150 -2.700712 -2.136509
6 4.676622 -0.892804 -2.415127
6 4.329462 -2.250520 -2.474389
1 2.852557 -3.769463 -2.220152
1 5.682856 -0.566697 -2.684221
1 5.080738 -2.978216 -2.783295
5 0.540292 -2.065570 -1.295002
6 0.260723 0.495136 -1.107750
6 -0.445080 1.698688 -0.897310
6 -0.296270 -0.806611 -1.047861
6 -1.841219 1.566312 -0.668219
6 -1.696362 -0.871153 -0.804102
6 -2.453277 0.298021 -0.645790
1 -3.521181 0.226898 -0.484770
6 -0.221311 -3.388664 -1.036248
6 -1.612821 -3.330634 -0.746142
6 0.397308 -4.656967 -1.017258
6 -2.318963 -4.516923 -0.441811
6 -0.297544 -5.819516 -0.728207
1 1.469639 -4.719242 -1.199055
6 -1.665175 -5.737626 -0.434733
1 -3.379014 -4.475355 -0.194651
1 0.214902 -6.782032 -0.711330
1 -2.228051 -6.638932 -0.185187
6 -0.691175 4.247892 -0.548045
6 -0.247473 5.577527 -0.381202
6 -2.077098 3.993008 -0.359229
6 -1.104155 6.620304 -0.069258
1 0.814842 5.791905 -0.490315
6 -2.953581 5.058105 -0.052558
6 -2.468548 6.348209 0.086094
1 -0.723308 7.634840 0.053876
1 -4.017590 4.875594 0.084742
1 -3.165523 7.153162 0.326789
7 -2.318397 -2.114948 -0.724255
6 -3.751843 -2.168453 -0.889572
6 -4.623312 -1.846497 0.150458
6 -4.253521 -2.530227 -2.142038
6 -6.001418 -1.890776 -0.065184
1 -4.234631 -1.529715 1.118333
6 -5.630308 -2.580955 -2.349208
1 -3.557043 -2.773114 -2.946928
6 -6.506708 -2.261449 -1.310853
1 -6.679605 -1.630569 0.748968
1 -6.018650 -2.865919 -3.328233
1 -7.584864 -2.297708 -1.474741
7 -2.606135 2.696185 -0.456803
6 -4.028015 2.518171 -0.314275
6 -4.839266 2.583413 -1.446297
6 -4.572553 2.242276 0.939878
6 -6.211984 2.370478 -1.320570
1 -4.387475 2.794025 -2.417488
6 -5.945817 2.029528 1.058708
1 -3.916944 2.174997 1.809348
6 -6.765360 2.093408 -0.069591
1 -6.849956 2.417513 -2.204481
1 -6.374911 1.808381 2.037417
1 -7.839064 1.923147 0.025872
6 0.852103 -1.247066 1.883025
6 0.494621 0.189509 2.034412
6 -0.768290 0.573379 2.319192
6 -1.871853 -0.421135 2.496386
6 -1.493456 -1.872795 2.395700
6 -0.224357 -2.256805 2.135598
8 -3.002210 -0.098502 2.746409
17 -1.201437 2.207394 2.529602
17 1.777074 1.292091 1.828478
6 -2.526834 -2.836448 2.636596
6 0.181824 -3.632015 2.160386
7 -3.353063 -3.623359 2.807284
7 0.539438 -4.727173 2.227660

8 1.977703 -1.592535 1.602177
16 4.685727 -3.584648 0.677086
8 5.248185 -4.480295 -0.297355
8 4.970195 -2.172440 0.713398
8 3.134300 -3.852510 0.762197
6 5.174473 -4.241883 2.330611
9 4.925608 -5.533492 2.395896
9 4.488744 -3.611967 3.267963
9 6.463502 -4.030144 2.511191
1 2.653315 -3.043006 1.086911

E (BS1) = -4273.14194931 au
H (BS1) = -4272.412268 au
G (BS1) = -4272.558474 au
E (BS2) = -4276.91712618 au
#............................

V2

6 1.683022 2.367413 -0.872282
6 2.093813 1.042003 -1.051785
6 3.424480 0.573792 -1.081107
6 4.442709 1.499053 -0.889445
6 4.087863 2.843259 -0.695473
6 2.757727 3.266784 -0.689512
1 5.485846 1.183258 -0.868008
1 4.877098 3.579391 -0.535977
1 2.564503 4.324001 -0.510891
5 0.131045 2.570873 -0.851998
7 1.235626 -0.045568 -1.224228
6 1.989164 -1.208973 -1.397942
6 1.456446 -2.483735 -1.617257
6 3.357470 -0.873394 -1.314186
6 2.443648 -3.488177 -1.743710
6 4.283978 -1.896701 -1.469646
6 3.807488 -3.200456 -1.674278
1 2.154921 -4.529438 -1.879185
1 5.354892 -1.699388 -1.426422
1 4.527924 -4.014314 -1.766582
5 -0.110201 -2.543869 -1.624458
6 -0.112060 0.013867 -1.194968
6 -0.699195 1.291388 -0.977873
6 -0.818885 -1.212308 -1.357674
6 -2.098943 1.312978 -0.848017
6 -2.218453 -1.138548 -1.231797
6 -2.854553 0.107411 -0.961608
1 -3.933529 0.145548 -0.864498
6 -1.076892 -3.730463 -1.822205
6 -2.476030 -3.526128 -1.640067
6 -0.654168 -5.039749 -2.143637
6 -3.376135 -4.614107 -1.741219
6 -1.535844 -6.098132 -2.258112
1 0.405336 -5.222743 -2.312836
6 -2.905850 -5.876002 -2.044416
1 -4.442174 -4.467065 -1.581516
1 -1.172468 -7.096083 -2.505908
1 -3.613148 -6.703629 -2.118678
6 -0.720003 3.850793 -0.698873
6 -0.179561 5.155524 -0.679508
6 -2.128771 3.727760 -0.526600
6 -0.957724 6.278760 -0.466005
1 0.888676 5.283235 -0.847194
6 -2.919844 4.874509 -0.277277
6 -2.335865 6.126131 -0.248290
1 -0.506197 7.271488 -0.458844
1 -3.991363 4.783422 -0.112491
1 -2.960891 7.000352 -0.058992
7 -2.994090 -2.262528 -1.342331
6 -4.414356 -2.126477 -1.112009
6 -4.895771 -2.196558 0.193693
6 -5.263458 -1.884839 -2.189900
6 -6.260002 -2.016072 0.420701
1 -4.199143 -2.375861 1.014533
6 -6.625523 -1.705380 -1.951834
1 -4.853187 -1.832411 -3.200117
6 -7.122444 -1.769521 -0.648756
1 -6.647579 -2.063673 1.439373
1 -7.299238 -1.511121 -2.787659
1 -8.188408 -1.624656 -0.465657
7 -2.763048 2.480961 -0.589340
6 -4.184055 2.407393 -0.337433
6 -5.082422 2.638379 -1.377076
6 -4.619245 2.061247 0.940230
6 -6.449275 2.522459 -1.127269
1 -4.707415 2.901063 -2.368021
6 -5.988699 1.947781 1.178903
1 -3.882497 1.856106 1.719434
6 -6.901230 2.178410 0.148182
1 -7.162647 2.697764 -1.933830

1 -6.341685 1.670738 2.173336
1 -7.971650 2.085163 0.338623
6 1.870817 -0.556211 1.985476
6 1.191891 0.710035 2.204106
6 -0.169424 0.812567 2.155693
6 -1.030782 -0.346430 1.882543
6 -0.332159 -1.621787 1.764255
6 1.051973 -1.721527 1.810314
8 -2.250610 -0.253817 1.744383
17 -0.971907 2.304492 2.409125
17 2.188245 2.069788 2.516205
6 -1.160251 -2.775054 1.584956
6 1.674234 -3.004108 1.652510
7 -1.849413 -3.691647 1.439786
7 2.136159 -4.053726 1.520379
8 3.140509 -0.559911 1.978360
16 6.045090 -1.991878 1.252542
8 6.702847 -3.025149 0.475217
8 6.120796 -0.612923 0.792206
8 4.654271 -2.394678 1.687226
6 6.924750 -1.983327 2.869745
9 6.941491 -3.196258 3.398582
9 6.317279 -1.159285 3.711577
9 8.173375 -1.575146 2.699768
1 3.801072 -1.473096 1.822455

E (BS1) = -4273.14108718 au
H (BS1) = -4272.415673 au
G (BS1) = -4272.563950 au
E (BS2) = -4276.92027595 au
#............................

V3

6 1.981137 2.370832 -0.681646
6 2.395325 1.069423 -1.000225
6 3.731505 0.626482 -1.139738
6 4.744943 1.545602 -0.918275
6 4.388084 2.858882 -0.562916
6 3.056608 3.260197 -0.448471
1 5.794660 1.266962 -1.023237
1 5.176742 3.591784 -0.386673
1 2.866282 4.296799 -0.170831
5 0.429072 2.588062 -0.718432
7 1.545440 0.017754 -1.298238
6 2.302869 -1.109740 -1.656832
6 1.771746 -2.324658 -2.080648
6 3.667836 -0.774770 -1.576617
6 2.746052 -3.256987 -2.478108
6 4.598318 -1.740108 -1.952312
6 4.117995 -2.975357 -2.405787
1 2.434380 -4.227017 -2.872124
1 5.669799 -1.537776 -1.915977
1 4.832599 -3.736627 -2.723214
5 0.165473 -2.429893 -2.135375
6 0.194722 0.081040 -1.259526
6 -0.394935 1.336043 -0.979054
6 -0.520283 -1.112100 -1.544180
6 -1.806249 1.354142 -0.979608
6 -1.910648 -1.085295 -1.331900
6 -2.550816 0.138844 -1.085922
1 -3.630287 0.165381 -0.986244
6 -0.623704 -3.673208 -1.517550
6 -2.013878 -3.530093 -1.289862
6 -0.046980 -4.926346 -1.270138
6 -2.787142 -4.645199 -0.918887
6 -0.801650 -6.028035 -0.874678
1 1.029910 -5.043018 -1.408048
6 -2.180790 -5.881062 -0.719731
1 -3.861577 -4.548752 -0.768346
1 -0.324204 -6.993916 -0.702820
1 -2.796426 -6.733338 -0.425780
6 -0.419972 3.879601 -0.624475
6 0.129223 5.175824 -0.515627
6 -1.836837 3.782467 -0.733631
6 -0.652277 6.318551 -0.507858
1 1.211343 5.285743 -0.452405
6 -2.633870 4.951226 -0.746622
6 -2.044250 6.195590 -0.630095
1 -0.190031 7.303760 -0.433047
1 -3.713828 4.882439 -0.861301
1 -2.673909 7.086532 -0.648934
7 -2.639496 -2.263763 -1.411040
6 -4.070018 -2.173509 -1.331395
6 -4.697613 -2.034792 -0.090527
6 -4.820850 -2.181667 -2.506479
6 -6.082387 -1.888139 -0.030335
1 -4.092385 -2.032267 0.818638
6 -6.207736 -2.039832 -2.440452

1 -4.311397 -2.295045 -3.464104
6 -6.837717 -1.888077 -1.205207
1 -6.573367 -1.772287 0.937277
1 -6.796161 -2.044877 -3.359348
1 -7.921630 -1.771034 -1.156126
7 -2.479213 2.538827 -0.850868
6 -3.923748 2.494929 -0.822379
6 -4.628028 2.277212 -2.005350
6 -4.581777 2.608879 0.400708
6 -6.017470 2.171561 -1.959800
1 -4.081487 2.158285 -2.942290
6 -5.972020 2.509289 0.435321
1 -4.003843 2.768453 1.312842
6 -6.688991 2.289600 -0.741985
1 -6.574717 1.989227 -2.879810
1 -6.495087 2.595486 1.388766
1 -7.776283 2.204418 -0.709428
6 -0.159706 3.355651 -4.011628
6 -1.036400 4.495748 -3.999434
6 -0.552995 5.760533 -3.803400
6 0.875383 6.017149 -3.549292
6 1.737175 4.837485 -3.595062
6 1.236347 3.564548 -3.843838
8 1.314270 7.136571 -3.319974
17 -1.566336 7.131927 -3.845388
17 -2.710450 4.207910 -4.254906
6 3.132171 5.052877 -3.368318
6 2.128778 2.444579 -3.943689
7 4.256739 5.226302 -3.168831
7 2.851645 1.550783 -4.049236
8 -0.704410 2.185591 -4.185844
16 -0.086901 -1.432983 -4.768529
8 0.499144 -1.937204 -5.984965
8 -0.266330 -2.513933 -3.712756
8 0.430492 -0.183152 -4.202785
6 -1.844687 -1.049960 -5.184856
9 -1.867161 -0.228105 -6.218692
9 -2.433213 -0.461093 -4.155570
9 -2.496523 -2.156030 -5.485797
1 -0.101656 1.393518 -4.167408

E (BS1) = -4273.13258178 au
H (BS1) = -4272.405317 au
G (BS1) = -4272.549170 au
E (BS2) = -4276.90522883 au
#............................

V4

6 -5.154035 -2.227191 -0.284936
6 -3.996853 -2.931980 -0.651815
6 -3.903269 -4.323302 -0.893553
6 -5.068296 -5.071243 -0.812777
6 -6.267210 -4.407694 -0.494183
6 -6.312031 -3.038479 -0.229180
1 -5.063072 -6.147438 -0.993102
1 -7.190579 -4.985758 -0.434716
1 -7.279066 -2.609198 0.034280
5 -4.918373 -0.709864 0.030280
7 -2.742569 -2.369182 -0.806334
6 -1.811408 -3.373958 -1.122558
6 -0.445180 -3.169028 -1.291053
6 -2.487685 -4.608063 -1.172549
6 0.280939 -4.352293 -1.506644
6 -1.729975 -5.752619 -1.410925
6 -0.345879 -5.605414 -1.571132
1 1.367348 -4.304246 -1.614079
1 -2.193750 -6.739044 -1.457615
1 0.262901 -6.494458 -1.744324
5 0.086197 -1.660052 -1.128054
6 -2.460945 -1.055213 -0.642799
6 -3.500484 -0.207307 -0.203049
6 -1.124446 -0.645406 -0.886739
6 -3.135484 1.145491 -0.002172
6 -0.860506 0.743302 -0.846569
6 -1.835024 1.618198 -0.364415
1 -1.606275 2.674424 -0.268689
6 1.026940 -0.970205 -2.220019
6 1.201236 0.431257 -2.126325
6 1.774718 -1.663828 -3.179030
6 2.174306 1.074145 -2.908835
6 2.716641 -1.026565 -3.985134
1 1.625893 -2.741419 -3.281496
6 2.924943 0.343474 -3.826500
1 2.339911 2.146859 -2.817517
1 3.294338 -1.593580 -4.716707
1 3.671469 0.860760 -4.432214
6 -5.868210 0.364335 0.614041
6 -7.206265 0.110037 0.981698

6 -5.360556 1.670245 0.862954
6 -8.011076 1.071497 1.571112
1 -7.630298 -0.877315 0.809612
6 -6.173161 2.642988 1.491651
6 -7.477896 2.341244 1.837544
1 -9.045659 0.848870 1.835963
1 -5.777377 3.630799 1.720359
1 -8.088915 3.099770 2.329362
7 0.386528 1.196584 -1.250883
6 0.718122 2.563674 -0.970584
6 0.321667 3.584812 -1.837590
6 1.388407 2.860456 0.216221
6 0.597174 4.911450 -1.509179
1 -0.208725 3.332200 -2.758311
6 1.662604 4.189840 0.539299
1 1.676955 2.043365 0.880420
6 1.265811 5.214152 -0.320913
1 0.285100 5.712053 -2.181780
1 2.184959 4.424402 1.468162
1 1.477241 6.253777 -0.065035
7 -4.043513 2.024976 0.517539
6 -3.630057 3.400371 0.685991
6 -2.829049 3.747877 1.771342
6 -3.997632 4.339834 -0.275576
6 -2.388517 5.065271 1.894509
1 -2.540759 2.985747 2.496799
6 -3.555692 5.655111 -0.141469
1 -4.618920 4.035366 -1.119864
6 -2.751511 6.017134 0.940782
1 -1.756626 5.344726 2.738704
1 -3.836557 6.397529 -0.889814
1 -2.403151 7.046462 1.039378
16 0.725003 -1.377165 1.658034
8 -0.615298 -0.831984 1.866304
8 1.833126 -0.761011 2.346257
8 1.042464 -1.612614 0.191675
6 0.636798 -3.103868 2.305915
9 -0.316882 -3.778275 1.683440
9 0.362584 -3.062473 3.597143
9 1.790626 -3.710539 2.116950
6 -3.893721 -0.430713 3.326594
6 -4.176129 -1.807562 3.057999
6 -5.424808 -2.329693 3.289128
6 -6.517051 -1.502789 3.798628
6 -6.180656 -0.127832 4.137373
6 -4.915892 0.381170 3.908682
8 -7.653140 -1.973920 3.948438
17 -5.778498 -3.964126 2.986003
17 -2.903940 -2.781258 2.448174
6 -7.217545 0.670303 4.719571
6 -4.597682 1.740623 4.244720
7 -8.070356 1.290897 5.188634
7 -4.351069 2.832559 4.523788
8 -2.751413 0.137178 3.081205
1 -2.046613 -0.422079 2.657281
16 -10.542040 -1.520890 2.432225
8 -11.594132 -0.653516 1.973023
8 -9.512635 -2.007730 1.540425
8 -9.923735 -0.912798 3.730741
6 -11.343689 -3.028029 3.130018
9 -12.331578 -2.685181 3.930392
9 -10.444626 -3.718571 3.807175
9 -11.809973 -3.762766 2.139788
1 -8.987030 -1.293272 3.913888

E (BS1) = -5234.40530434 au
H (BS1) = -5233.628074 au
G (BS1) = -5233.796433 au
E (BS2) = -5239.05366285 au
#............................

V5

6 -4.837129 -1.412847 -0.691496
6 -3.667991 -2.163825 -0.856983
6 -3.697868 -3.482284 -1.344827
6 -4.898705 -4.058294 -1.746663
6 -6.073362 -3.307643 -1.643117
6 -6.037898 -2.016631 -1.117340
1 -4.918838 -5.077730 -2.136606
1 -7.022469 -3.736564 -1.967316
1 -6.969464 -1.452377 -1.055556
5 -4.609955 -0.009351 -0.115026
7 -2.349885 -1.771040 -0.566474
6 -1.544122 -2.952020 -0.706352
6 -0.252951 -3.269415 -0.257336
6 -2.346761 -3.980702 -1.259471
6 0.223844 -4.557617 -0.544134
6 -1.848424 -5.254113 -1.534634

6 -0.532354 -5.532898 -1.199917
1 1.227431 -4.821573 -0.206057
1 -2.499634 -6.011561 -1.974597
1 -0.103723 -6.516690 -1.396024
5 0.738268 -2.317123 0.515794
6 -2.046020 -0.389290 -0.461778
6 -3.163454 0.474908 -0.186645
6 -0.777570 0.220938 -0.712467
6 -3.013831 1.898040 -0.200300
6 -0.796792 1.626122 -0.969334
6 -1.863900 2.476948 -0.717347
1 -1.764234 3.545358 -0.881664
6 0.642133 -0.141902 -0.840648
6 1.321016 0.994977 -1.342211
6 1.424626 -1.200349 -0.351571
6 2.698091 1.022312 -1.577262
6 2.804404 -1.172478 -0.558866
6 3.430797 -0.097911 -1.207230
1 3.176915 1.909343 -1.993511
1 3.414781 -1.988373 -0.167483
1 4.510622 -0.112254 -1.365501
6 -5.677180 0.970787 0.427377
6 -6.981590 0.588640 0.804720
6 -5.312389 2.331617 0.587837
6 -7.901181 1.496053 1.307382
1 -7.265211 -0.463369 0.730052
6 -6.259484 3.267186 1.086930
6 -7.529802 2.849446 1.425914
1 -8.898743 1.170129 1.606648
1 -5.986001 4.312475 1.218108
1 -8.243837 3.578526 1.813108
7 0.430037 2.048440 -1.427110
6 0.782308 3.394560 -1.727941
6 1.159838 3.735020 -3.028294
6 0.759598 4.361740 -0.718725
6 1.517161 5.051631 -3.317993
1 1.168567 2.965869 -3.802736
6 1.097740 5.679378 -1.021298
1 0.474569 4.073691 0.295258
6 1.479817 6.024830 -2.318871
1 1.815702 5.318816 -4.332897
1 1.071664 6.436961 -0.236244
1 1.750683 7.055979 -2.551304
7 -4.039681 2.748255 0.244514
6 -3.721436 4.148780 0.332648
6 -2.946004 4.601726 1.400189
6 -4.151564 5.026066 -0.661581
6 -2.600661 5.950300 1.473826
1 -2.612982 3.886572 2.154993
6 -3.808138 6.375731 -0.578617
1 -4.748621 4.646326 -1.492990
6 -3.032569 6.837469 0.485808
1 -1.992882 6.309007 2.306159
1 -4.143524 7.067142 -1.353112
1 -2.762079 7.892967 0.545040
16 1.626284 -3.695050 2.683374
8 0.551900 -4.655054 2.742393
8 1.756922 -2.684765 3.716215
8 1.793723 -3.044062 1.283481
6 3.211812 -4.633716 2.667812
9 3.190563 -5.523000 1.694222
9 3.336243 -5.250002 3.826682
9 4.223851 -3.809452 2.492437
6 -4.348781 0.893206 3.250415
6 -5.329129 1.836911 3.834862
6 -6.578349 1.439186 4.163455
6 -7.035700 0.026287 3.982836
6 -6.017979 -0.937246 3.441820
6 -4.768142 -0.536612 3.121717
8 -8.135486 -0.346210 4.288110
17 -7.727739 2.494705 4.844529
17 -4.775600 3.432695 4.087350
6 -6.451978 -2.295444 3.288666
6 -3.765727 -1.455953 2.667535
7 -6.813936 -3.382426 3.157460
7 -2.952267 -2.206662 2.340543
8 -3.235119 1.252577 2.921568
16 0.132904 -0.444923 2.581500
8 -0.191845 -1.602609 1.686914
8 1.478066 0.030096 2.650857
8 -0.805113 0.745692 2.268910
6 -0.497272 -1.008587 4.281484
9 -0.799806 -2.273540 4.263273
9 -1.578648 -0.296267 4.546590
9 0.435530 -0.740784 5.151678
1 -1.804535 0.666642 2.405078

E (BS1) = -5233.20351940 au

H (BS1) = -5232.448047 au
G (BS1) = -5232.607686 au
E (BS2) = -5237.83590500 au
#............................

V6

6 -4.679782 -1.513122 -0.627170
6 -3.490757 -2.200385 -0.866087
6 -3.454667 -3.452000 -1.500915
6 -4.633843 -4.050633 -1.922429
6 -5.845419 -3.384074 -1.694490
6 -5.864829 -2.143898 -1.062420
1 -4.615032 -5.019563 -2.424151
1 -6.780586 -3.838891 -2.023786
1 -6.820816 -1.636259 -0.927161
5 -4.489045 -0.097233 -0.069732
7 -2.177038 -1.761533 -0.566534
6 -1.312559 -2.883447 -0.845743
6 -0.012884 -3.173012 -0.427128
6 -2.066164 -3.866523 -1.532795
6 0.569470 -4.329874 -0.976423
6 -1.483275 -5.028454 -2.020158
6 -0.124469 -5.228987 -1.781128
1 1.605317 -4.553862 -0.720943
1 -2.083892 -5.764246 -2.557098
1 0.378622 -6.117013 -2.166465
5 0.835320 -2.333351 0.628332
6 -1.919786 -0.410972 -0.404704
6 -3.063232 0.435126 -0.182769
6 -0.620049 0.228251 -0.587732
6 -2.953350 1.852835 -0.258815
6 -0.668578 1.632483 -0.898873
6 -1.761651 2.446468 -0.747325
1 -1.693578 3.506405 -0.976173
6 0.783131 -0.101578 -0.646721
6 1.469289 1.053405 -1.140510
6 1.574328 -1.169380 -0.144712
6 2.855451 1.090498 -1.342619
6 2.942734 -1.124174 -0.330729
6 3.575503 -0.024880 -0.957252
1 3.338049 1.977531 -1.752551
1 3.557545 -1.934907 0.064625
1 4.659534 -0.031988 -1.085391
6 -5.578588 0.881446 0.416388
6 -6.851556 0.482580 0.873605
6 -5.268100 2.259345 0.444317
6 -7.790574 1.403504 1.313810
1 -7.091720 -0.582188 0.904610
6 -6.230138 3.204203 0.866847
6 -7.474595 2.771209 1.290426
1 -8.762760 1.070562 1.682013
1 -5.997756 4.267052 0.885273
1 -8.205707 3.506986 1.629904
7 0.576706 2.075886 -1.293982
6 0.894358 3.411335 -1.684505
6 1.299104 3.663726 -2.996061
6 0.797887 4.446265 -0.750997
6 1.611441 4.968708 -3.374595
1 1.363340 2.838891 -3.708007
6 1.093607 5.750122 -1.144764
1 0.492826 4.223254 0.273360
6 1.502904 6.011291 -2.453361
1 1.931238 5.171412 -4.397751
1 1.012811 6.563167 -0.421621
1 1.740072 7.032366 -2.756087
7 -3.997731 2.690060 0.041218
6 -3.754149 4.112807 0.000896
6 -3.091119 4.713900 1.069043
6 -4.167253 4.850990 -1.105877
6 -2.832917 6.083566 1.022878
1 -2.783515 4.103953 1.920420
6 -3.906191 6.220317 -1.142131
1 -4.682051 4.350536 -1.928002
6 -3.239417 6.835171 -0.080830
1 -2.312874 6.563442 1.853331
1 -4.221687 6.807217 -2.005900
1 -3.035182 7.906458 -0.114764
16 1.651379 -4.349790 2.317155
8 0.474685 -5.117226 1.986130
8 1.917940 -3.969976 3.684222
8 1.879590 -3.143211 1.360161
6 3.124598 -5.348198 1.825096
9 3.006518 -5.798149 0.588618
9 3.215226 -6.374055 2.650711
9 4.216520 -4.612910 1.916838
6 -4.471908 1.140762 3.431823
6 -5.421904 2.098304 3.920758
6 -6.650627 1.707621 4.380880

6 -7.049060 0.292843 4.436881
6 -6.035344 -0.654021 3.981150
6 -4.800482 -0.243825 3.493418
8 -8.152186 -0.068114 4.829758
17 -7.819377 2.838193 4.894514
17 -4.963344 3.753759 3.891410
6 -6.394413 -2.038211 4.013054
6 -3.889878 -1.230044 2.980592
7 -6.692891 -3.153612 4.034973
7 -3.184845 -2.040735 2.557690
8 -3.361147 1.616476 2.936324
16 -0.005535 -0.601328 2.637024
8 -0.151679 -1.802690 1.682870
8 1.234301 0.123511 2.503726
8 -1.261746 0.129254 2.660541
6 0.031655 -1.437227 4.301758
9 -0.534569 -2.621361 4.237357
9 -0.642620 -0.675823 5.145977
9 1.275352 -1.537724 4.709764
1 -2.672387 0.949449 2.692119

E (BS1) = -5233.22526683 au
H (BS1) = -5232.471331 au
G (BS1) = -5232.627903 au
E (BS2) = -5237.86695666 au
#............................

V7

6 -4.740508 -1.618593 -0.616347
6 -3.585362 -2.329170 -0.939892
6 -3.620732 -3.586472 -1.562460
6 -4.839187 -4.157232 -1.903947
6 -6.017265 -3.459651 -1.603634
6 -5.966439 -2.220695 -0.970774
1 -4.876951 -5.130019 -2.397069
1 -6.982190 -3.892714 -1.870525
1 -6.899149 -1.692380 -0.767199
5 -4.480771 -0.222399 -0.039477
7 -2.245447 -1.919595 -0.730124
6 -1.427072 -3.069212 -1.042724
6 -0.115289 -3.394912 -0.685051
6 -2.248889 -4.043662 -1.658995
6 0.384260 -4.611933 -1.181796
6 -1.736698 -5.248372 -2.124757
6 -0.384645 -5.510737 -1.918548
1 1.411445 -4.883935 -0.932882
1 -2.392046 -5.975136 -2.607241
1 0.056199 -6.443453 -2.273280
5 0.822970 -2.543629 0.277899
6 -1.956577 -0.574168 -0.574286
6 -3.060065 0.287314 -0.249656
6 -0.670622 0.052775 -0.852207
6 -2.927762 1.705383 -0.293065
6 -0.726235 1.455752 -1.163918
6 -1.778248 2.290508 -0.884476
1 -1.700630 3.357356 -1.078511
6 0.722764 -0.284497 -0.970280
6 1.391841 0.861839 -1.509493
6 1.519424 -1.368760 -0.520719
6 2.761122 0.873062 -1.804420
6 2.877233 -1.336779 -0.782262
6 3.486062 -0.250836 -1.453476
1 3.230100 1.745008 -2.260189
1 3.504526 -2.154911 -0.424945
1 4.558899 -0.278604 -1.652879
6 -5.492256 0.750947 0.596734
6 -6.712936 0.345978 1.171756
6 -5.144686 2.119211 0.671709
6 -7.567408 1.251576 1.781940
1 -6.974168 -0.714480 1.163597
6 -6.025119 3.052141 1.266239
6 -7.219875 2.611987 1.811254
1 -8.495266 0.921621 2.249902
1 -5.764891 4.107366 1.323962
1 -7.890608 3.326777 2.291534
7 0.497564 1.886463 -1.636217
6 0.794117 3.225190 -2.027953
6 0.176278 3.761815 -3.159126
6 1.680840 3.987393 -1.264324
6 0.446157 5.079974 -3.523672
1 -0.508361 3.144346 -3.743416
6 1.955946 5.299138 -1.646440
1 2.143808 3.549985 -0.377897
6 1.337085 5.846637 -2.771401
1 -0.035985 5.505820 -4.404808
1 2.649880 5.898665 -1.055477
1 1.550292 6.876224 -3.062946
7 -3.918055 2.547750 0.146187

6 -3.663019 3.968751 0.116447
6 -2.848510 4.530944 1.097715
6 -4.225564 4.745621 -0.893794
6 -2.591931 5.900944 1.062713
1 -2.421344 3.890735 1.871928
6 -3.964968 6.115207 -0.918967
1 -4.858332 4.275712 -1.648952
6 -3.149719 6.691541 0.056707
1 -1.953435 6.350540 1.824528
1 -4.398842 6.732873 -1.706569
1 -2.946898 7.763307 0.031968
16 1.748542 -4.279801 2.139933
8 0.608473 -5.161244 2.047592
8 1.994091 -3.525099 3.349299
8 1.920685 -3.378720 0.888988
6 3.248893 -5.312885 1.862812
9 3.112190 -6.015700 0.752909
9 3.383203 -6.133730 2.887102
9 4.320115 -4.549117 1.767667
6 -4.004994 0.962462 3.518253
6 -4.838662 1.952648 4.138308
6 -6.031004 1.609778 4.718985
6 -6.513340 0.231038 4.719924
6 -5.631060 -0.758751 4.125388
6 -4.424559 -0.400544 3.544856
8 -7.608847 -0.105365 5.192640
17 -7.036197 2.774836 5.450742
17 -4.290656 3.574476 4.122476
6 -6.094752 -2.112681 4.104160
6 -3.615248 -1.376329 2.874266
7 -6.472363 -3.203086 4.079005
7 -2.962254 -2.140110 2.305920
8 -2.916199 1.375897 2.946843
16 0.214285 -0.902818 2.418689
8 -0.080878 -2.046129 1.428753
8 1.607017 -0.538397 2.480281
8 -0.768395 0.168180 2.264361
6 -0.253315 -1.692623 4.063502
9 -0.620438 -2.941738 3.921828
9 -1.263207 -0.995505 4.569458
9 0.770518 -1.596492 4.877853
1 -2.260530 0.689889 2.635336
16 -10.557488 1.606788 4.927641
8 -11.654712 2.334542 5.506249
8 -9.747821 2.164110 3.867361
8 -9.675822 1.033816 6.077031
6 -11.256367 0.042627 4.243304
9 -11.988733 -0.564295 5.153946
9 -10.262480 -0.749883 3.876483
9 -12.001296 0.325191 3.191888
1 -8.782626 0.656907 5.732499

E (BS1) = -6194.50251829 au
H (BS1) = -6193.698047 au
G (BS1) = -6193.880509 au
E (BS2) = -6200.01929741 au
#............................

7.2 Calculated mechanism

The experimental results demonstrate that the oxidation of azaborine **1** by 2,3-dichloro-5,6-dicyano-1,4-benzoquinone (DDQ) in the presence of trifluoromethanesulfonic acid (HOTf) under anhydrous dichloromethane (DCM) conditions affords the π-extended product **P**. To gain insight into the reaction mechanism, we carried out density functional theory (DFT) calculations at the SMD/M06-2X/def2-TZVP//SMD/M06-2X/def2-SVP level of theory. For computational simplicity, we used a model azaborine **S**, in which the mesityl (Mes) substituents in **S** were replaced by phenyl (Ph) (Figure 3B).

Stoichiometrically, the transformation from azaborine **S** to the π-extended product **P** requires two equivalents of the oxidant DDQ. Therefore, mechanistically, it is reasonable to assume that the transformation occurs in two major stages. Initially, the azaborine molecule reacts with the first molecule of DDQ to form an intermediate. Subsequently, this intermediate reacts with the second molecule of DDQ to yield the final π-extended product **P**. Indeed, our DFT calculations support this

mechanism, as detailed below.

In the first stage, the reaction begins with the formation of a van der Waals complex **V1** among azaborine **S**, DDQ and HOTf (Figure 3B). From this van der Waals complex, our calculations indicate that a single-electron transfer (SET) occurs from the π-system of **S** to DDQ, leading to the formation of the radical ion pair **V2**. The observation of the single-electron transfer here is consistent that the commonly observed one-electron reduction of quinones to form the semiquinone anion radicals. These partially reduced species are often stabilized via hydrogen bonding with or by direct protonation from a Brønsted acid[S22,23].

Within the DDQ–HOTf moiety of the ion pair in **V2**, the proton is primarily associated with the partially reduced DDQ, as indicated by a H···DDQ bond length of 1.137 Å and an OTf···H bond length of 1.263 Å. The step **S → V2** is nearly thermodynamically neutral (ΔG = +0.9 kcal/mol) and involves a very minimal structural reorganization, consistent with a facile electron transfer. Spin density analysis (Fig. S9) shows that the unpaired electron in the oxidized azaborine is predominantly localized on the central ring, which is reasonable given that the electron-deficient radical center is stabilized by the lone pairs on adjacent nitrogen atoms.

From the ion pair **V2**, the in situ generated triflate anion (OTf^-) then coordinates to the Lewis acidic boron center in the oxidized azaborine via transition state **TS1**, forming the boron–triflate adduct **V3**. Subsequently, another molecule of HOTf enters, forming a hydrogen bond with the remaining carbonyl group of the partially reduced DDQ to yield **V4** (Figure 3B). This hydrogen-bonding interaction increases the electrophilicity of the DDQ moiety, facilitating a second electron transfer through transition state **TS2**. This second electron transfer completes the reduction of DDQ to give 4,5-dichloro-3,6-dihydroxyphthalonitrile (DCDHP) while simultaneously triggering a rearrangement that results in C–C bond formation, generating the arenium intermediate **A**. The formation of **A** is highly exergonic (ΔG = –19.2 kcal/mol), rendering it essentially irreversible and establishing **A** as the resting state of the process. This is consistent with experimental observations showing that, in the presence of water, **A** undergoes hydrolysis to give a boronic acid derivative.

From **A**, a cationic boryl migration occurs via **TS3**, from which the electrons from the cleaved B–C σ-bond are redistributed to the π-system, resulting in re-aromatization of the central ring. This process leads to B–C bond cleavage and formation of a π-complex **Pi**, where the cationic boryl is coordinated by the adjacent phenyl π-system. Deprotonation of the C-H bond associated with the boryl-coordinated unit in **Pi** by the free triflate anion occurs via **TS4**, with an activation barrier of 19.1 kcal/mol relative to **A**, yielding intermediate **B** (ΔG = –20.5 kcal/mol). The transformation from **A** to **B** via **TS4** is rate-determining.

As previously mentioned, the second stage involves the reaction of intermediate **B** with a second molecule of DDQ. Again, the first step here is the formation of a van der Waals complex (**V5**) among **B**, DDQ and HOTf. From this complex, a single-electron transfer generates the radical zwitterion **V7** and a partially reduced DDQ. Spin density analysis on **V7** reveals that, like observed in **V2**, the unpaired electron within the azaborine moiety in **V7** is predominantly delocalized over the central aromatic ring (Fig. S10). Following formation of the radical zwitterion **V7**, a second single-electron transfer proceeds through transition state **TS5**, wherein the unpaired electron delocalized over the central aromatic ring is transferred to the partially reduced DDQ molecule, generating a new zwitterion **B2** and competing the reduction of DDQ to give DCDHP. In **B2**, the +2 charge is delocalized across a triannular π-system, as illustrated in Extended Data Fig. 1c. This charge assignment is further supported by the LUMO plot (Fig. S11) of **B2**, which corresponds to

the HOMO of the neutral triannular π-system. **B2** then undergoes C–C bond formation, followed by the elimination of $B(OTf)_3$via a low-energy transition state **TS6**, with a $\Delta G^{\ddagger}$ = 3.7 kcal/mol. This facile step leads to the final π-extended product **P**, with an overall free energy change of –102.0 kcal/mol, indicating a highly exergonic process.

The transformation of **B2** to **P** formally involves redistribution of the four electrons from the two C–$B(OTf)_2$ σ bonds. Two electrons are transferred to the electron-deficient triannular π-system, while the other two electrons facilitate C–C bond formation, resulting in the formation of **P**.

It is evident that the second stage is more facile compared to the first stage. Notably, the intermediate **B**, which contains an 8-membered ring, is more easily being oxidized by DDQ than azaborine **S**.

7.3 Spin natural orbitals and frontier molecular orbitals for selected species

Cartesian coordinates, total potential energy (E), enthalpy (H), and Gibbs free energy (G) for all optimized structures at the SMD/M06-2X/BS1 level of theory are provided in xyz format. Total energies calculated at the SMD/M06-2X/BS2//SMD/M06-2X/BS1 level are also included.

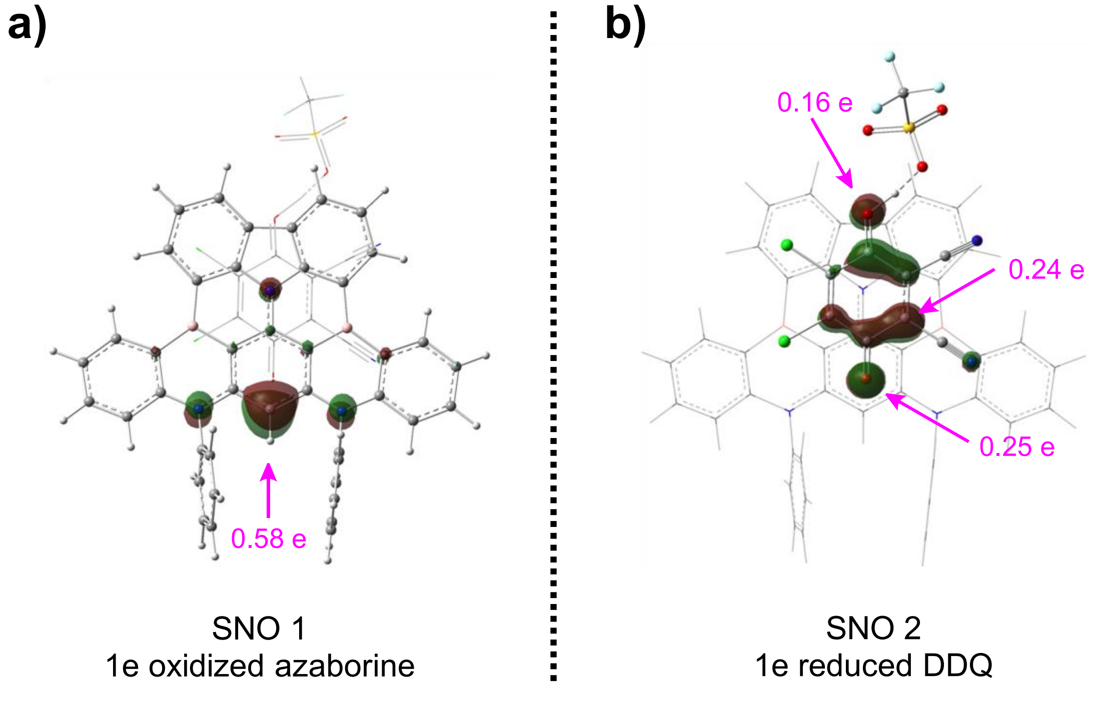

Fig. S9. Spin Natural Orbital (SNO) plots depicting the distribution of two unpaired electrons in singlet biradical **V2**. (a) The SNO for α unpaired electron localized on the oxidized azaborine moiety. (b) The SNO for β unpaired electron localized on the partially reduced DDQ moiety.

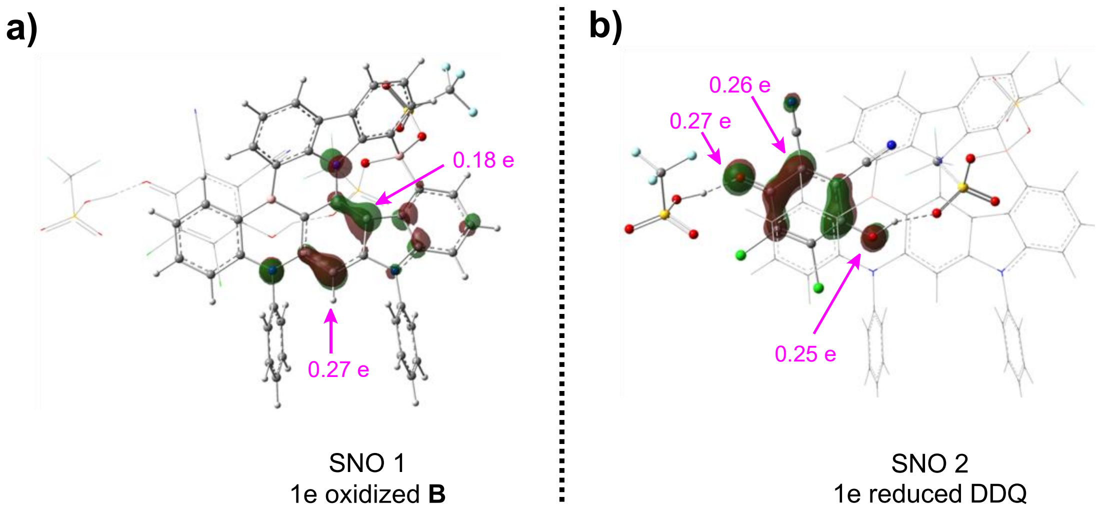

Fig. S10. Spin Natural Orbital (SNO) plots depicting the distribution of two unpaired electrons in singlet biradical **V7**. (a) The SNO for α unpaired electron localized on the oxidized azaborine moiety. (b) The SNO for β unpaired electron localized on the partially reduced DDQ moiety.

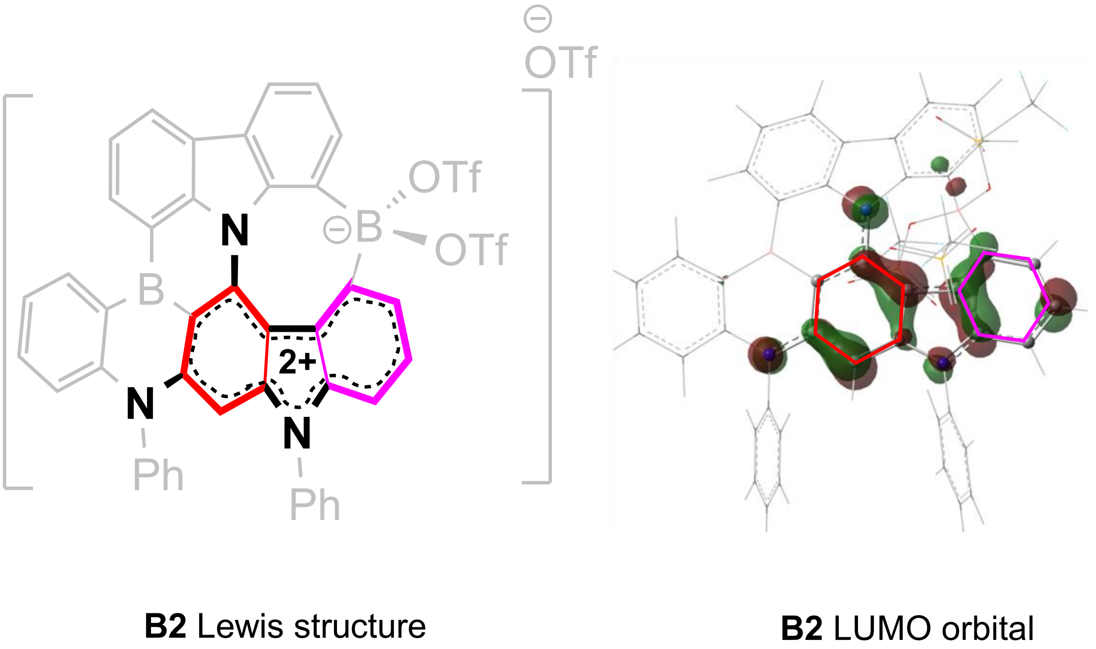

Fig. S11. LUMO orbital representation of the **B2** intermediate. For visual reference, key aromatic rings are highlighted in colors that correspond to those used in the Lewis structure.

Cartesian coordinate for optimized structures of BDA products at the B3LYP/6-31g(d)-D3 level of theory are also provided in xyz format.

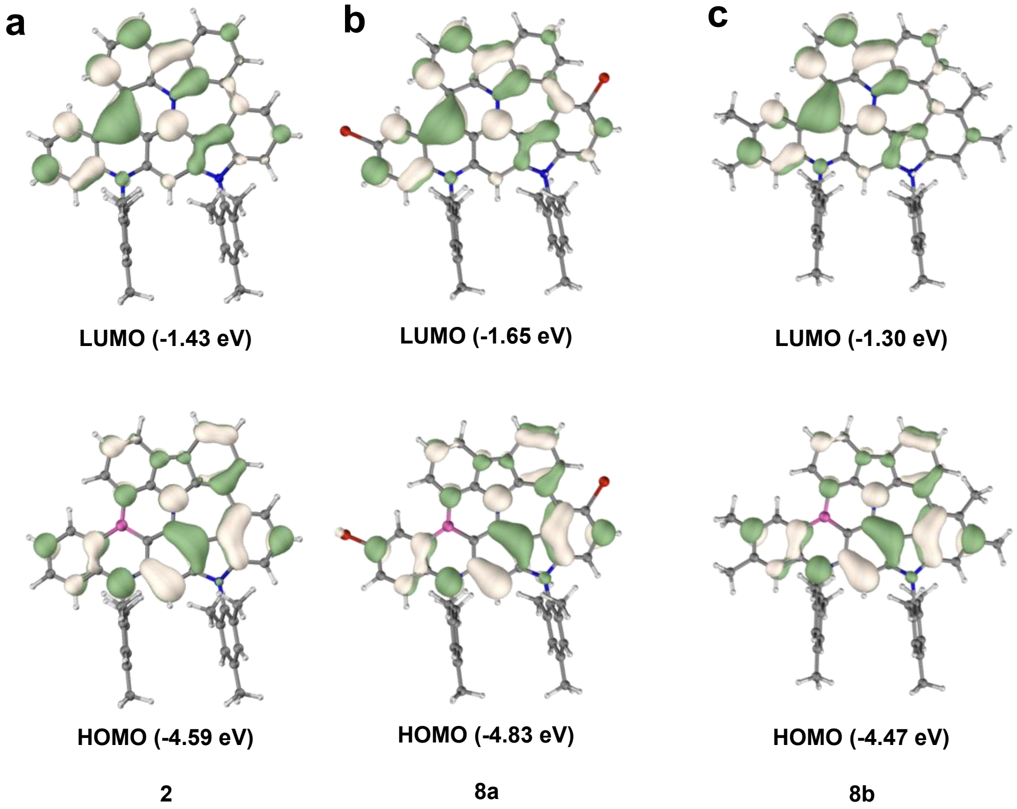

Fig. S12. Frontier molecular orbital profiles and energies **2** (a), **8a** (b), and **8b** (c).

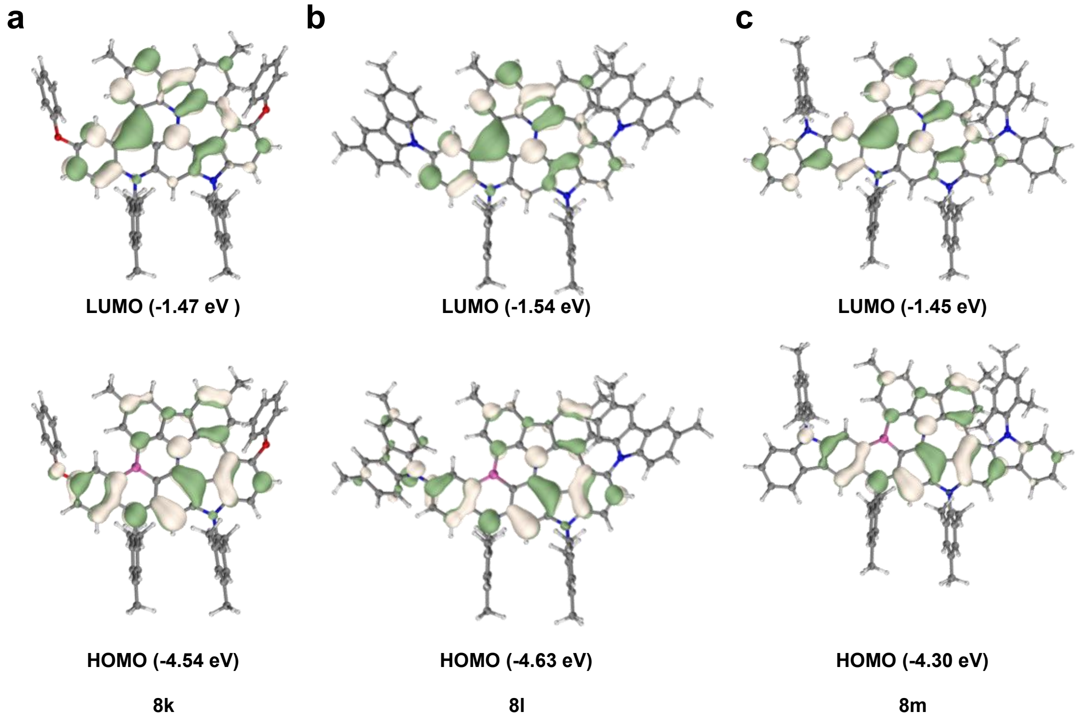

Fig. S13. Frontier molecular orbital profiles and energies **8k** (a), **8l** (b), and **8m** (c). *n*-Hexyl and *tert*-butyl groups were replaced by methyl groups for calculation simplicity.

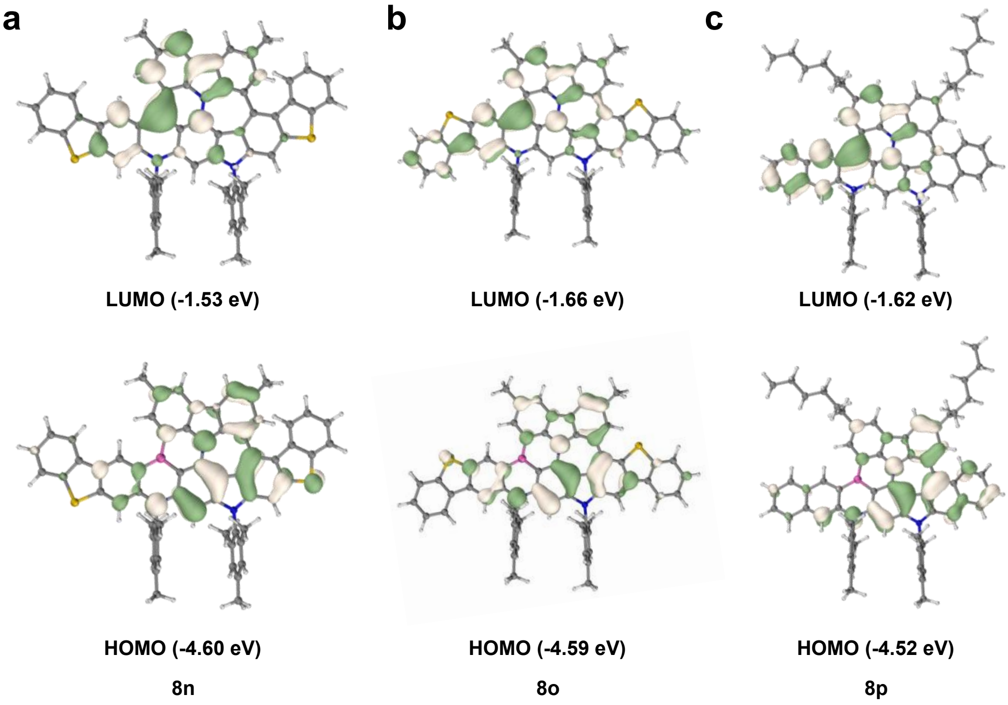

Fig. S14. Frontier molecular orbital profiles and energies **8n** (a), **8o** (b), and **8p** (c). *n*-Hexyl groups of **8n** and **8o** were replaced by methyl groups for calculation simplicity.

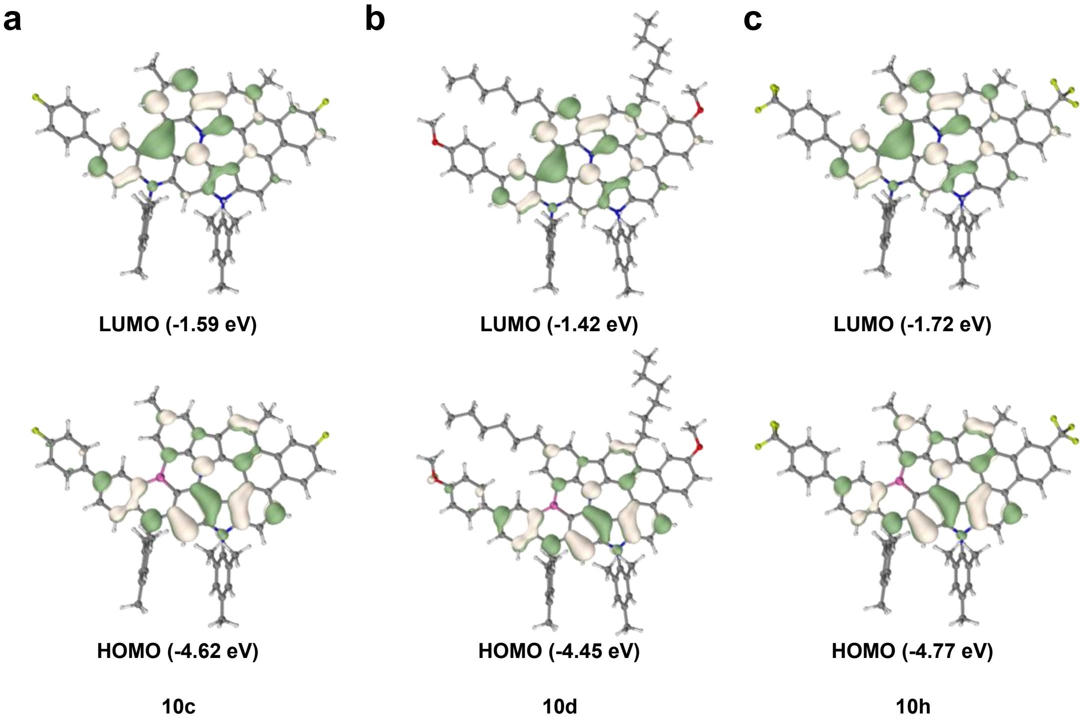

Fig. S15. Frontier molecular orbital profiles and energies **10c** (a), **10d** (b), and **10h** (c). *n*-Hexyl groups of **10c** and **10h** were replaced by methyl groups for calculation simplicity.

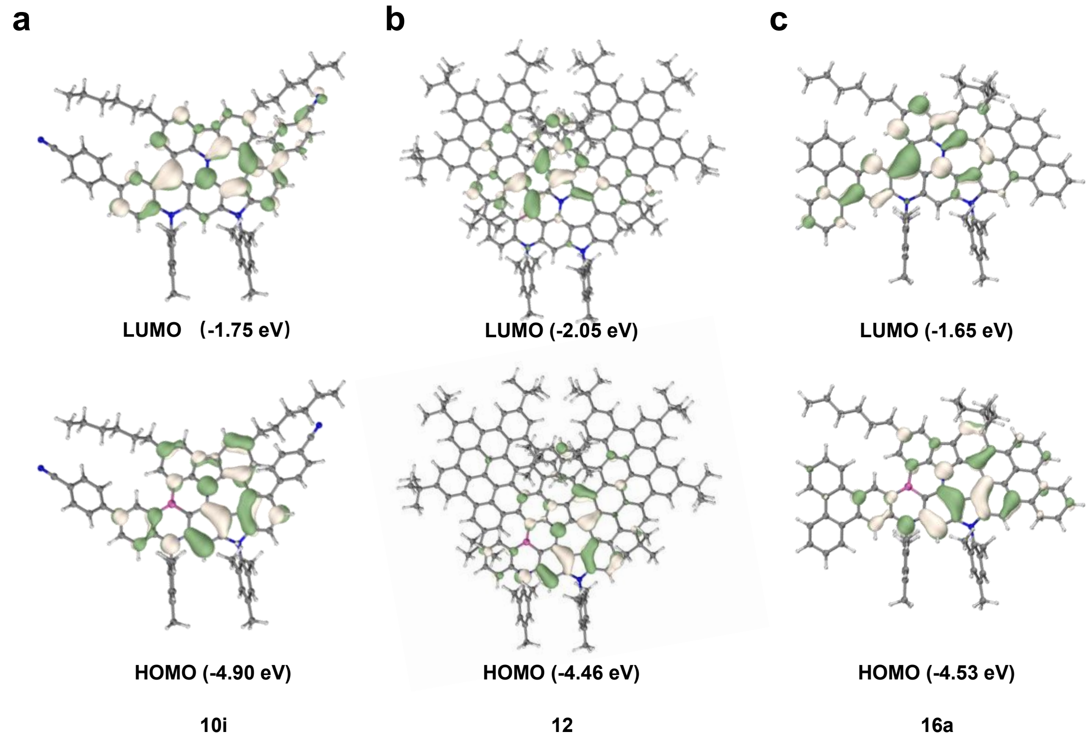

Fig. S16. Frontier molecular orbital profiles and energies **10i** (a), **12** (b), and **16a** (c).

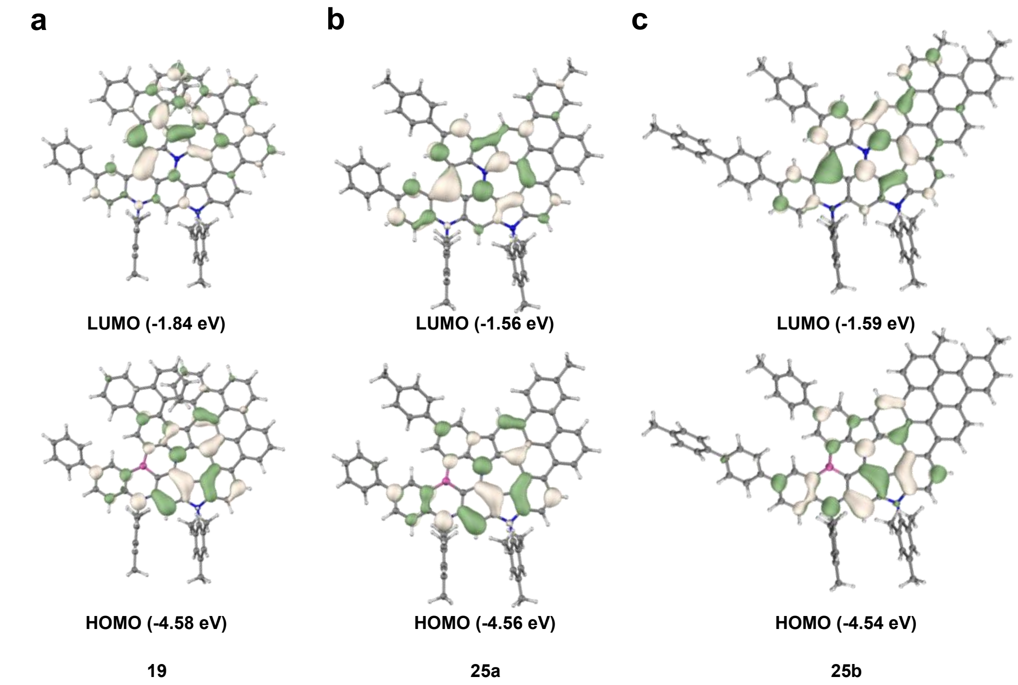

Fig. S17. Frontier molecular orbital profiles and energies **19** (a), **25a** (b), and **25b** (c).

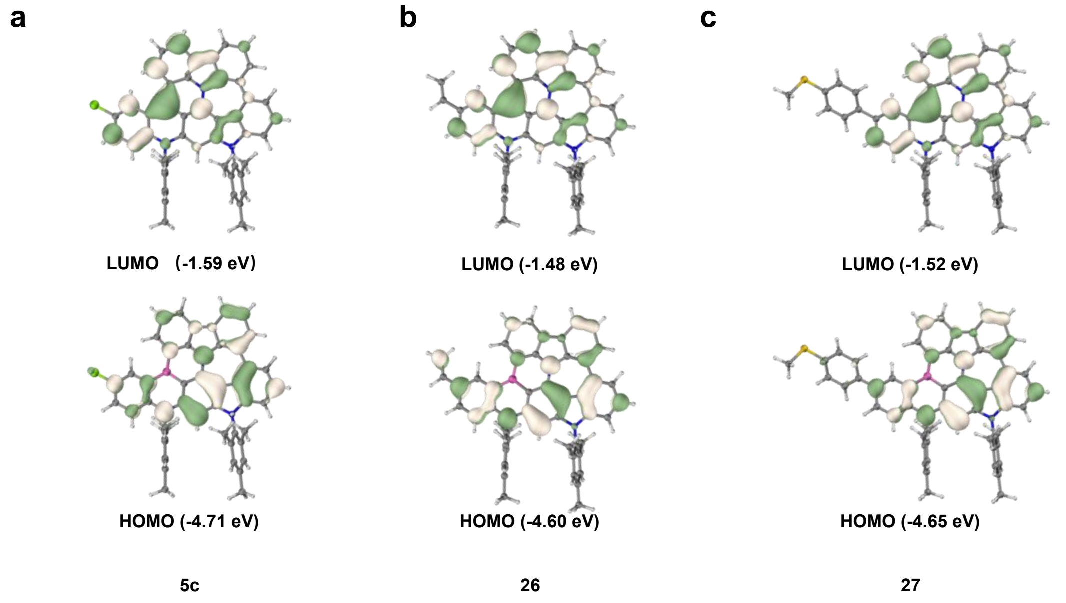

Fig. S18. Frontier molecular orbital profiles and energies **5c** (a), **26** (b), and **27** (c).

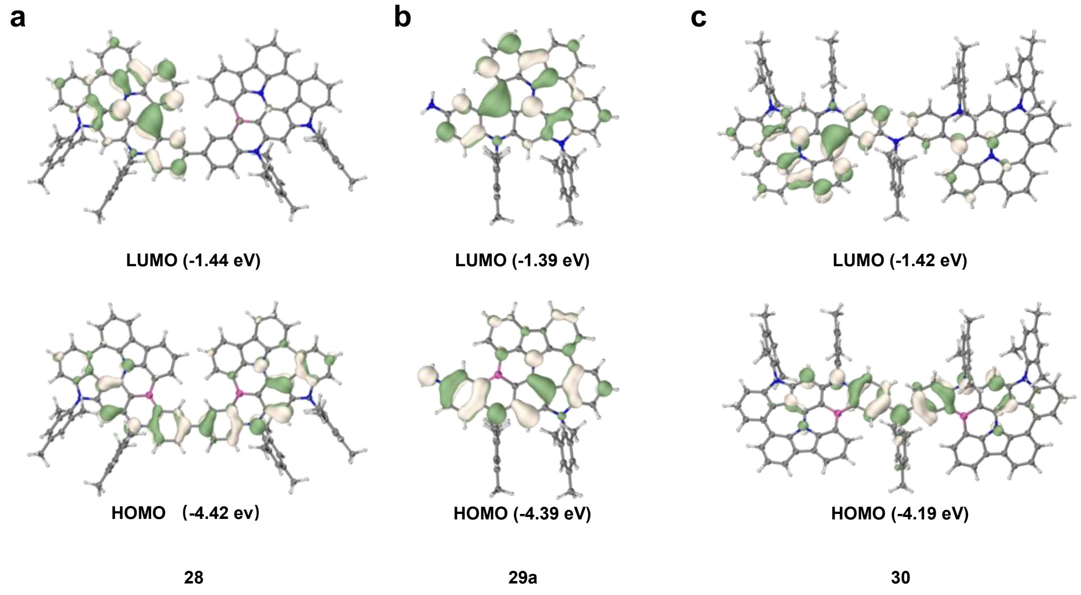

Fig. S19. Frontier molecular orbital profiles and energies **28** (a), **29a** (b), and **30** (c).

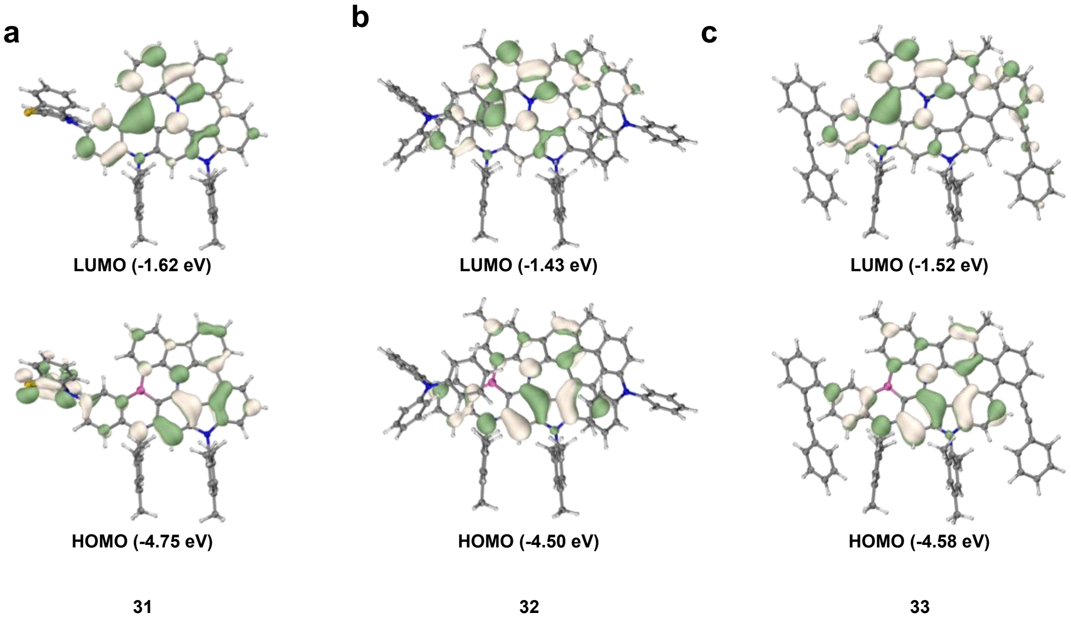

Fig. S20. Frontier molecular orbital profiles and energies **31** (a), **32** (b), and **33** (c). *n*-Hexyl groups of **32** and **33** were replaced by methyl groups for calculation simplicity. *tert*-Butyl groups of **33** were replaced by hydrogen atoms for calculation simplicity.

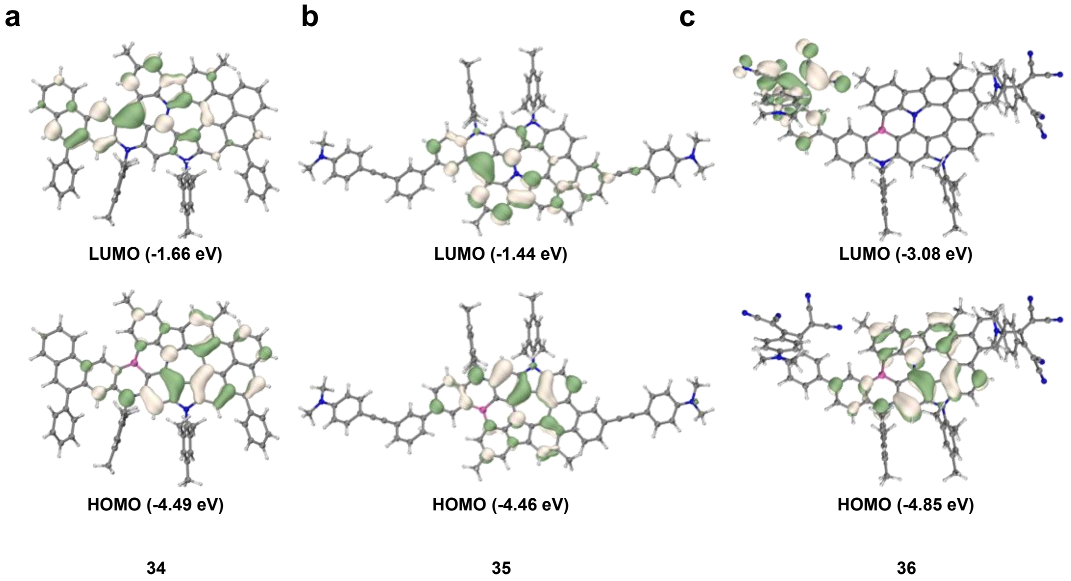

Fig. S21. Frontier molecular orbital profiles and energies **34** (a), **35** (b), and **36** (c). *n*-Hexyl groups were replaced by methyl groups for calculation simplicity. *tert*-Butyl groups of **34** were replaced by hydrogen atoms for calculation simplicity.

Table S18. Calculated photophysical properties data of **2**, **10k** and **25a** at the PBE0/6-311G(d) level.

compd	Excited States	Wavelength [nm]	Oscillator Strength	Transitions
**2**	S_1	480	0.0764	HOMO->LUMO (89%)
	S_2	428	0.2308	HOMO->L+1 (89%)
	S_3	372	0.0418	H-1->LUMO (91%)
	S_4	337	0.1363	H-1->L+1 (76%)
	S_5	333	0.0233	H-2->LUMO (59%), HOMO->L+2 (24%)
	S_6	321	0.0791	H-3->LUMO (59%), HOMO->L+2 (27%)
	S_7	313	0.4297	H-3->LUMO (31%), H-2->LUMO (21%), HOMO->L+2 (34%)
	S_8	308	0.0569	HOMO->L+3 (42%), HOMO->L+4 (12%), HOMO->L+5 (25%)
	S_9	305	0.0073	HOMO->L+4 (74%), HOMO->L+5 (13%)
	S_{10}	302	0.0347	H-2->L+1 (47%), HOMO->L+3 (23%)
**10k**	S_1	495	0.0743	HOMO->LUMO (93%)
	S_2	430	0.3285	HOMO->L+1 (91%)
	S_3	382	0.0021	H-1->LUMO (83%)
	S_4	369	0.0510	H-2->LUMO (28%), H-1->L+1 (17%), HOMO->L+2 (44%)
	S_5	354	0.1456	H-2->LUMO (27%), H-1->L+1 (43%), HOMO->L+3 (12%)
	S_6	350	0.0032	H-3->LUMO (22%), H-2->LUMO (28%), HOMO->L+2 (32%)
	S_7	340	0.0638	H-3->LUMO (65%), HOMO->L+2 (13%)
	S_8	333	0.2761	H-2->L+1 (11%), HOMO->L+3 (57%)
	S_9	329	0.1915	H-2->L+1 (19%), HOMO->L+4 (57%)
	S_{10}	324	0.1349	H-2->L+1 (30%), H-1->L+1 (12%), HOMO->L+3 (11%),

				HOMO->L+4 (28%)
**25a**	S_1	513	0.0894	HOMO->LUMO (95%)
	S_2	453	0.2330	HOMO->L+1 (94%)
	S_3	397	0.0381	H-1->LUMO (86%)
	S_4	386	0.0167	H-1->L+1 (38%), HOMO->L+2 (24%), HOMO->L+3 (10%)
	S_5	368	0.2467	H-2->LUMO (17%), HOMO->L+2 (50%), HOMO->L+3 (21%)
	S_6	359	0.3099	H-1->L+1 (40%), HOMO->L+2 (15%), HOMO->L+3 (29%)
	S_7	354	0.0849	H-3->LUMO (15%), H-2->LUMO (49%), HOMO->L+3 (22%)
	S_8	346	0.0267	H-2->L+1 (55%)
	S_9	343	0.2645	H-3->LUMO (67%), HOMO->L+3 (11%)
	S_{10}	340	0.0640	HOMO->L+4 (73%)

8. Photophysical and electrochemical properties of skeletal-edited products

8.1 UV-Vis absorption and emission spectra

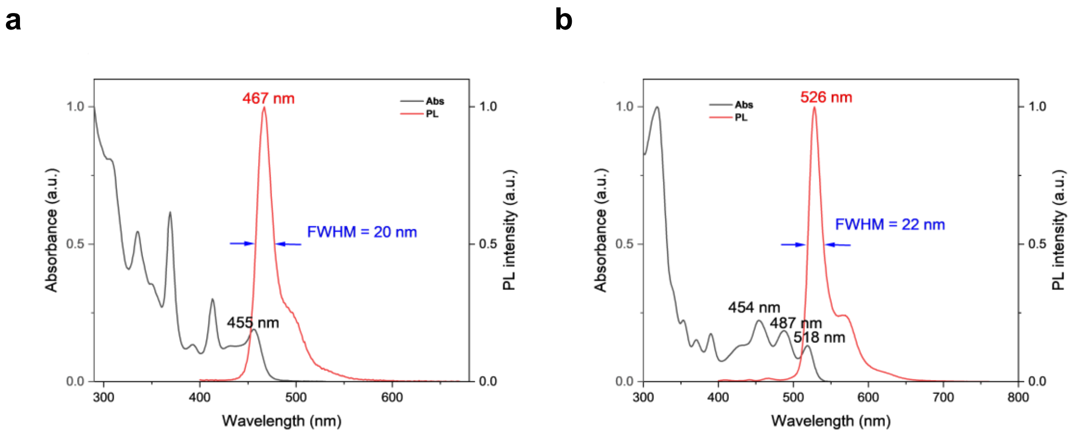

Fig. S22. UV-Vis absorption and emission spectra of **1** (a) and **2** (b) in toluene.

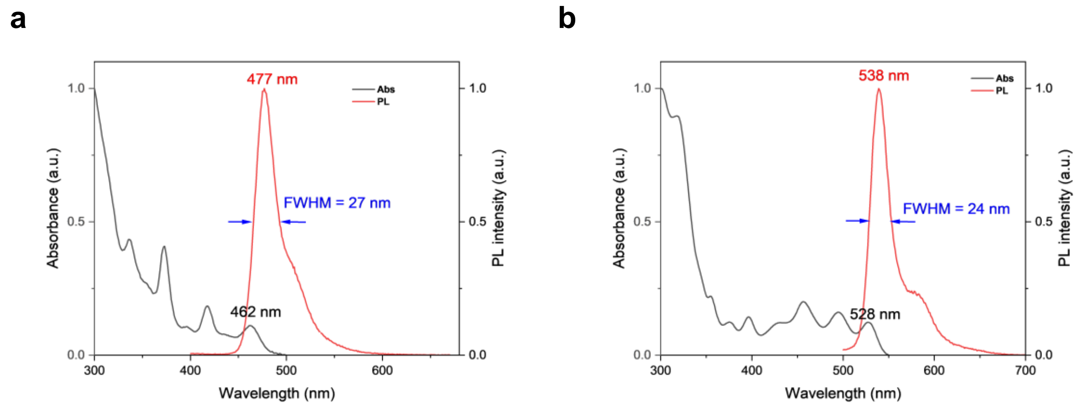

Fig. S23. UV-Vis absorption and emission spectra of **3b** (a) and **5b** (b) in toluene.

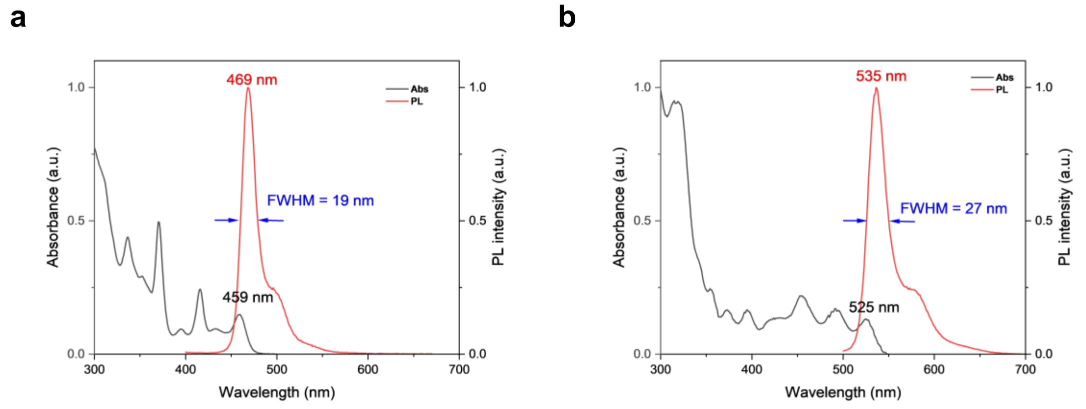

Fig. S24. UV-Vis absorption and emission spectra of **3c** (a) and **5c** (b) in toluene.

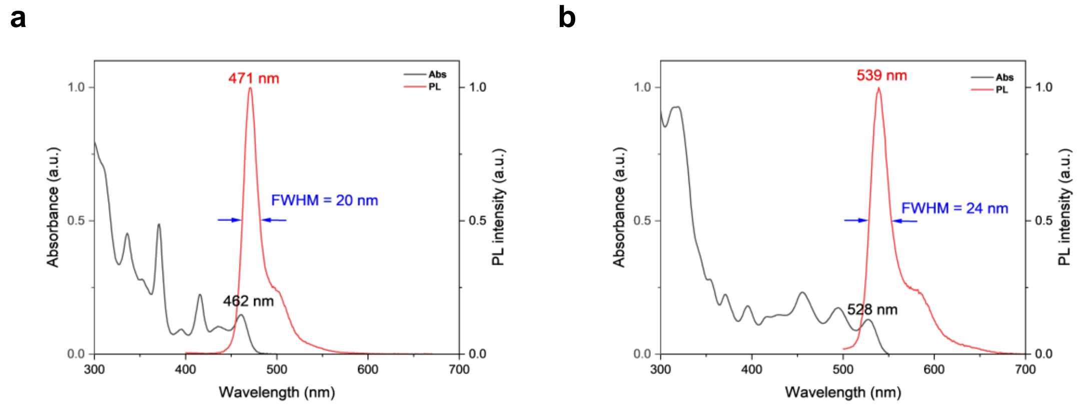

Fig. S25. UV-Vis absorption and emission spectra of **3d** (a) and **5d** (b) in toluene.

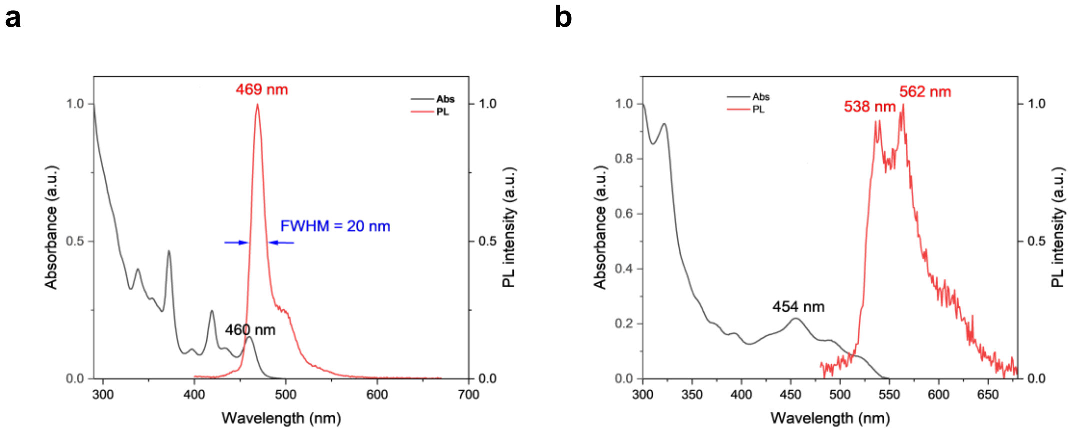

Fig. S26. UV-Vis absorption and emission spectra of **7a** (a) and **8a** (b) in toluene.

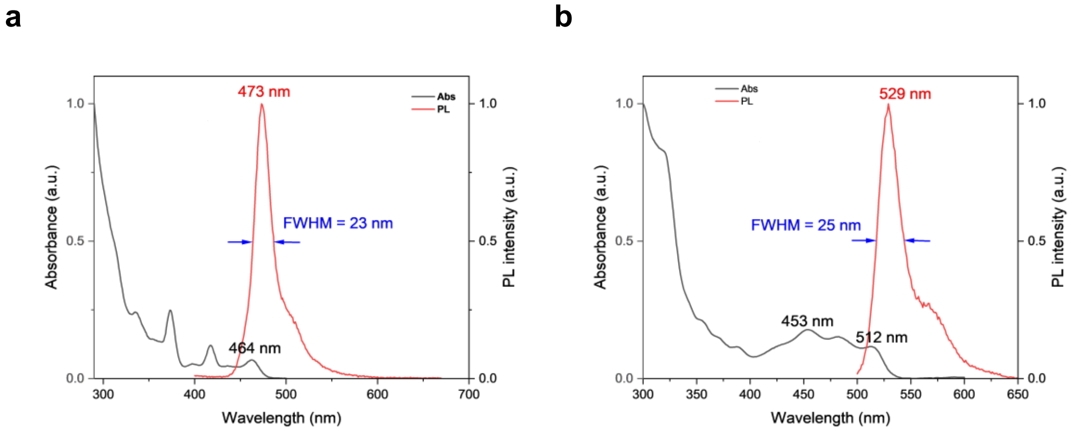

Fig. S27. UV-Vis absorption and emission spectra of **7b** (a) and **8b** (b) in toluene.

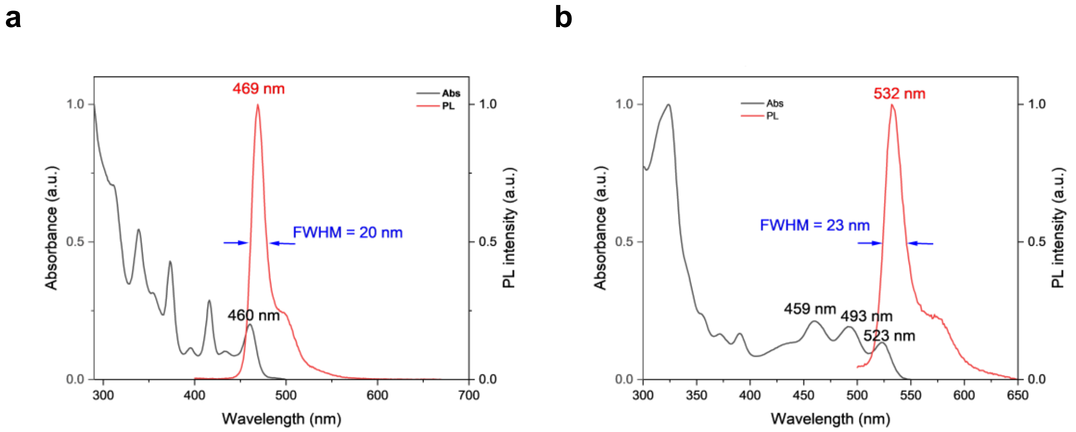

Fig. S28. UV-Vis absorption and emission spectra of **7c** (a) and **8c** (b) in toluene.

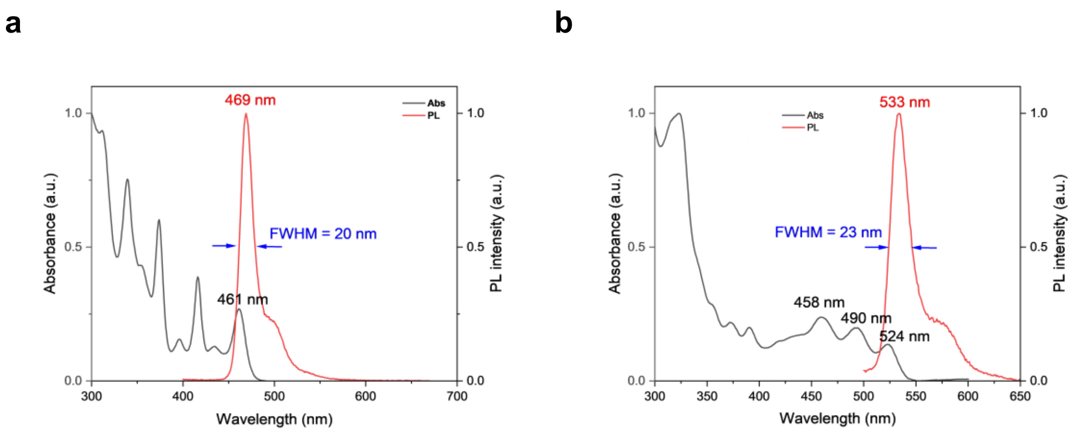

Fig. S29. UV-Vis absorption and emission spectra of **7d** (a) and **8d** (b) in toluene.

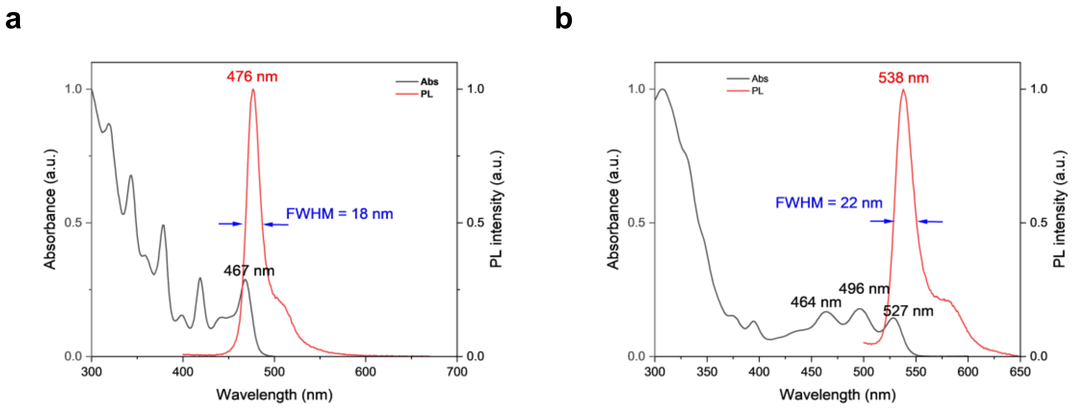

Fig. S30. UV-Vis absorption and emission spectra of **7e** (a) and **8e** (b) in toluene.

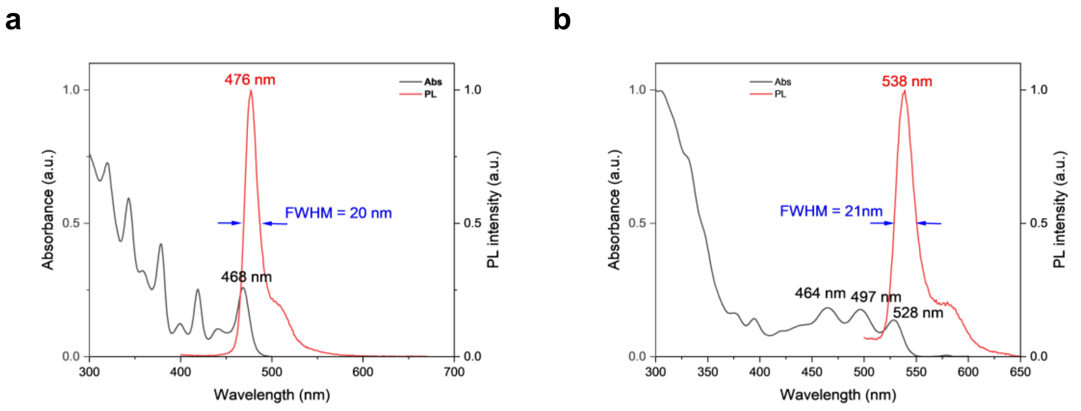

Fig. S31. UV-Vis absorption and emission spectra of **7f** (a) and **8f** (b) in toluene.

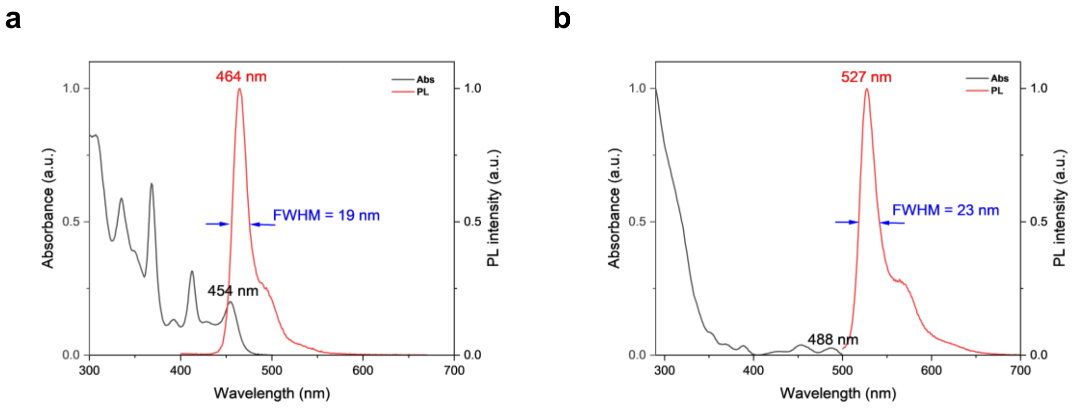

Fig. S32. UV-Vis absorption and emission spectra of **7g** (a) and **8g** (b) in toluene.

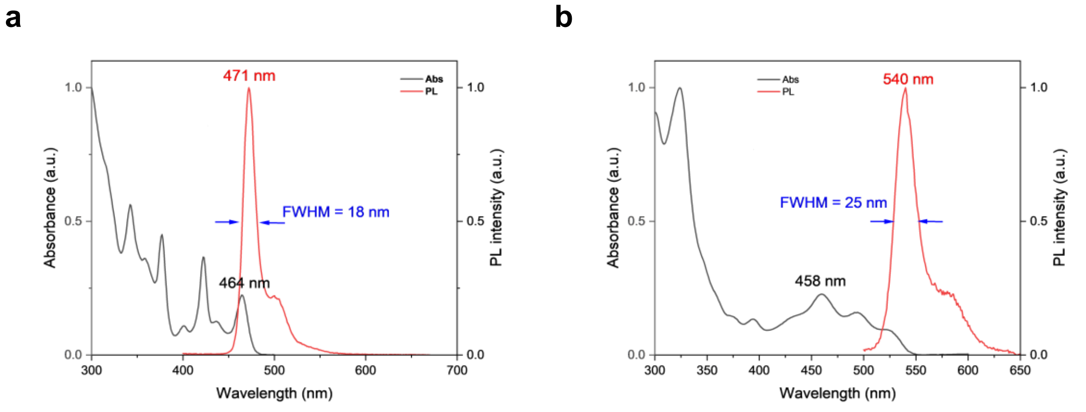

Fig. S33. UV-Vis absorption and emission spectra of **7h** (a) and **8h** (b) in toluene.

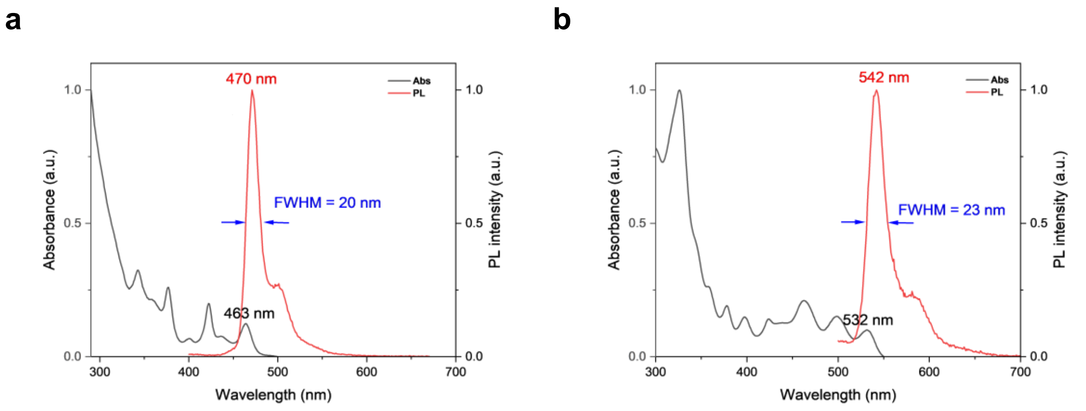

Fig. S34. UV-Vis absorption and emission spectra of **7i** (a) and **8i** (b) in toluene.

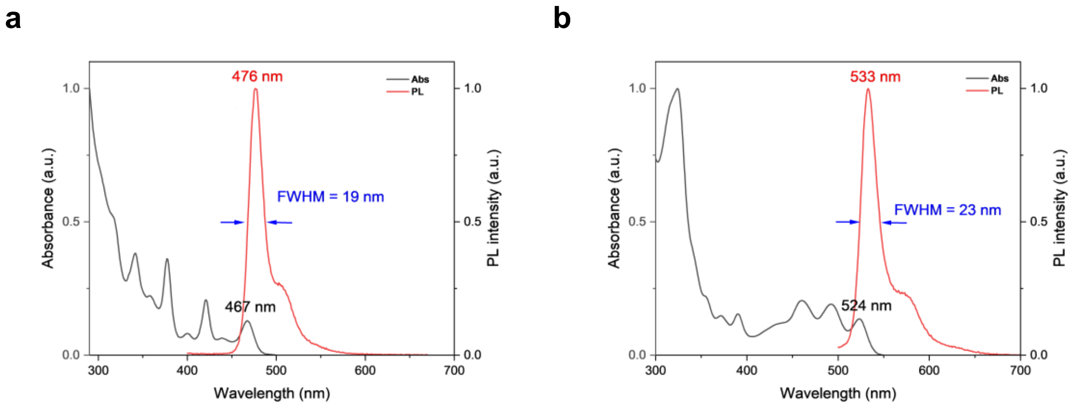

Fig. S35. UV-Vis absorption and emission spectra of **7j** (a) and **8j** (b) in toluene.

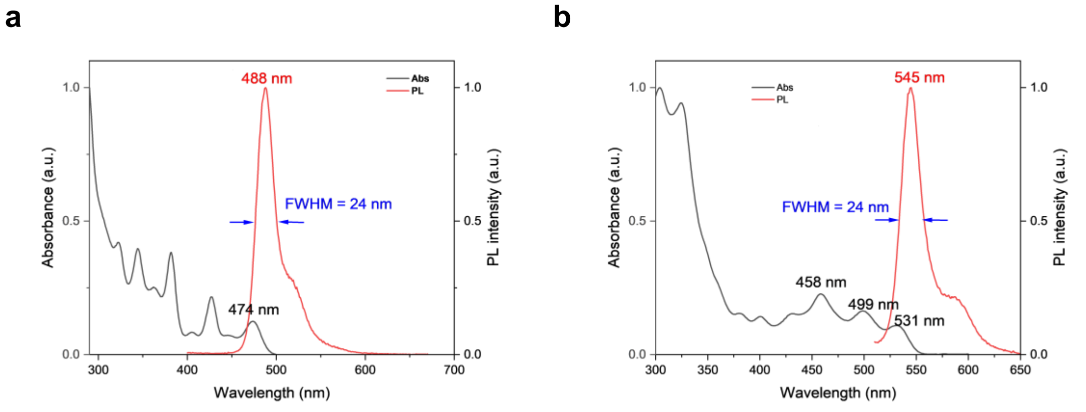

Fig. S36. UV-Vis absorption and emission spectra of **7k** (a) and **8k** (b) in toluene.

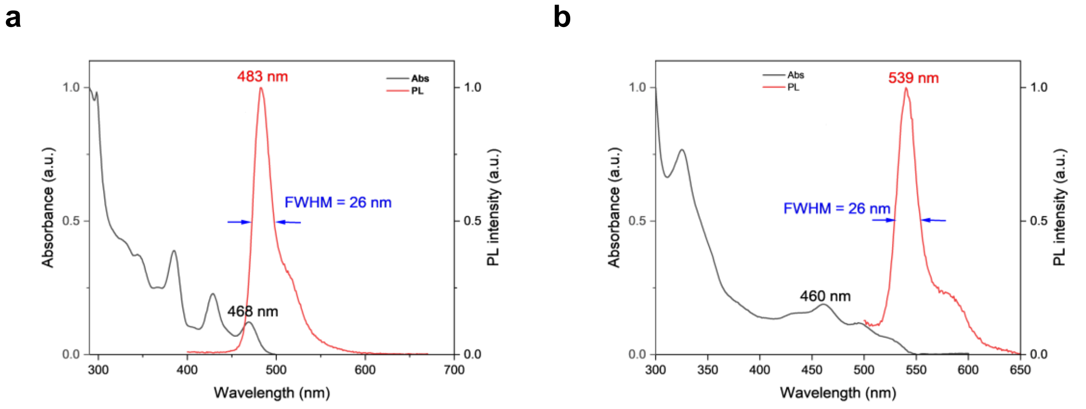

Fig. S37. UV-Vis absorption and emission spectra of **7l** (a) and **8l** (b) in toluene.

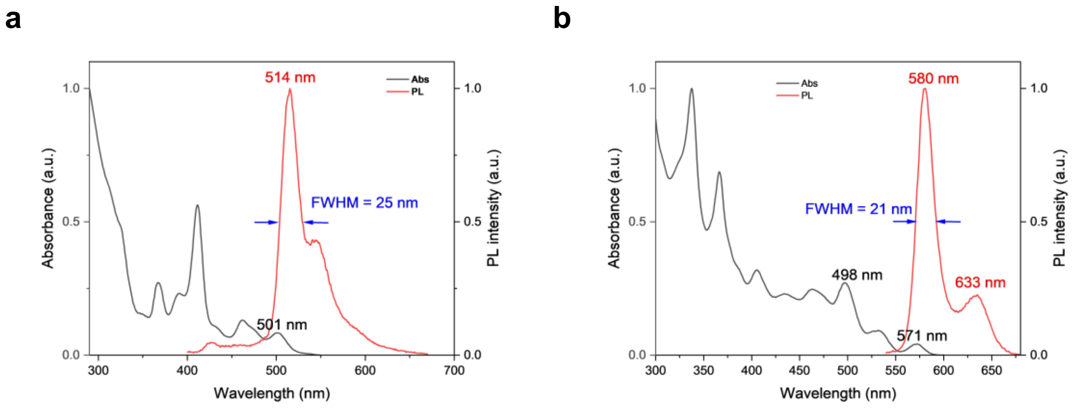

Fig. S38. UV-Vis absorption and emission spectra of **7m** (a) and **8m** (b) in toluene.

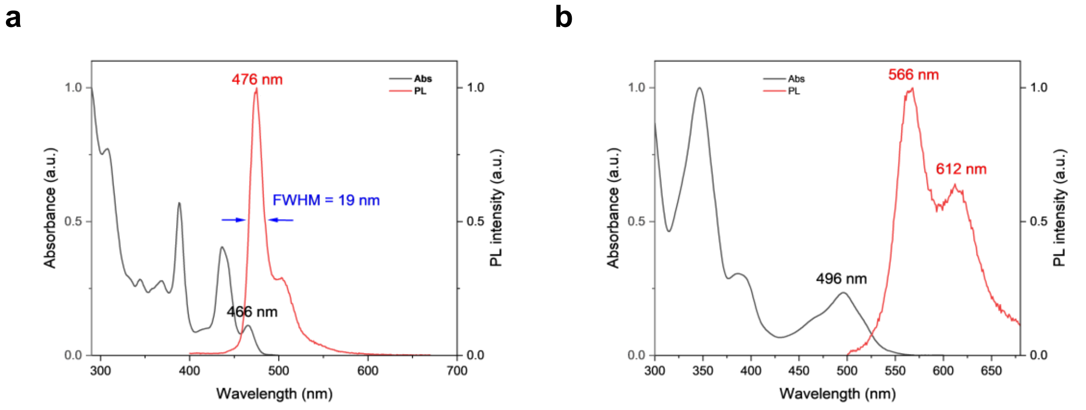

Fig. S39. UV-Vis absorption and emission spectra of **7n** (a) and **8n** (b) in toluene.

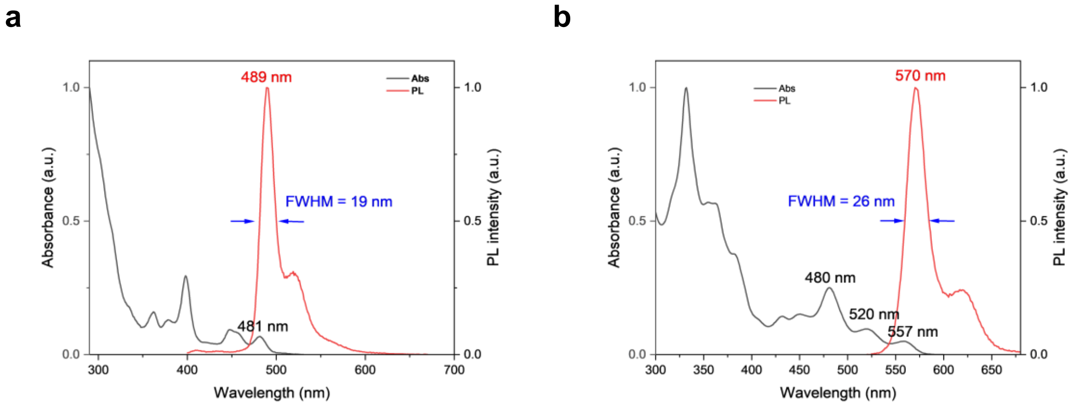

Fig. S40. UV-Vis absorption and emission spectra of **7o** (a) and **8o** (b) in toluene.

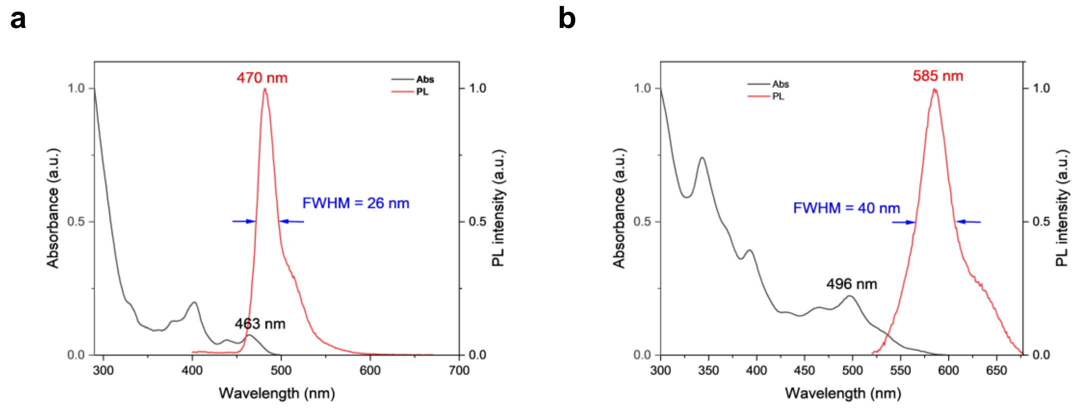

Fig. S41. UV-Vis absorption and emission spectra of **7p** (a) and **8p** (b) in toluene.

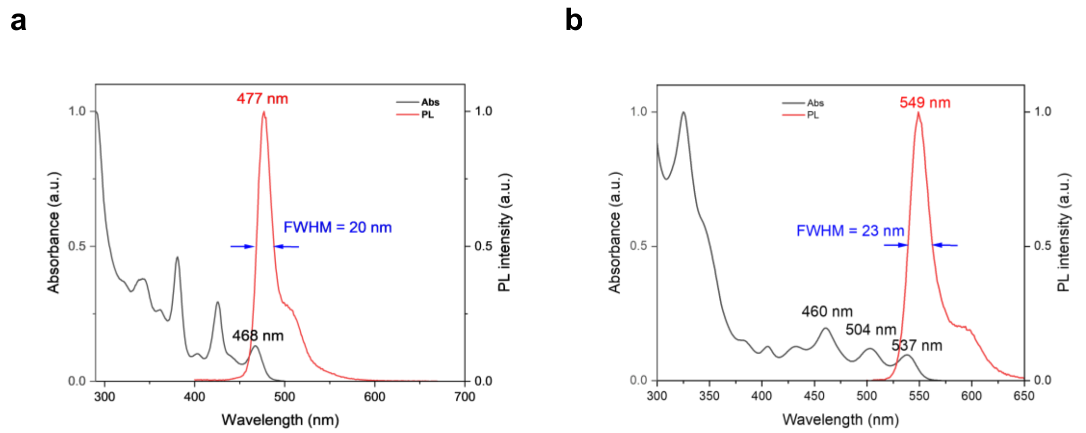

Fig. S42. UV-Vis absorption and emission spectra of **9a** (a) and **10a** (b) in toluene.

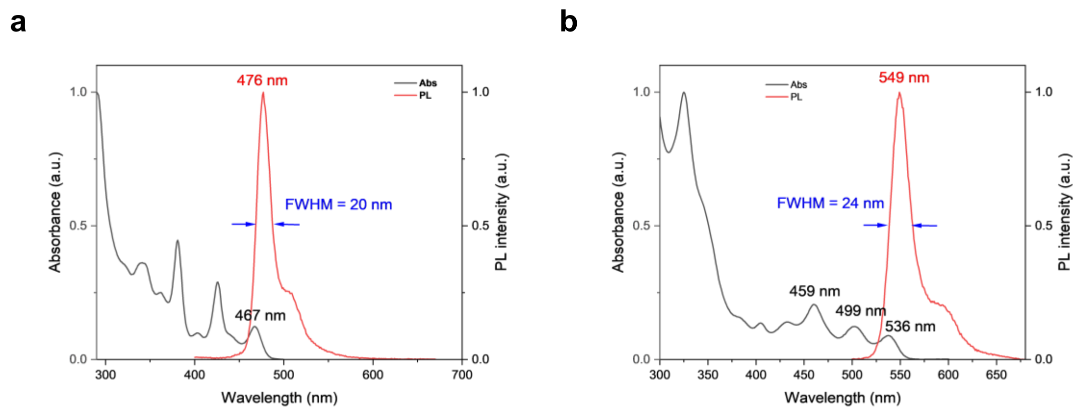

Fig. S43. UV-Vis absorption and emission spectra of **9b** (a) and **10b** (b) in toluene.

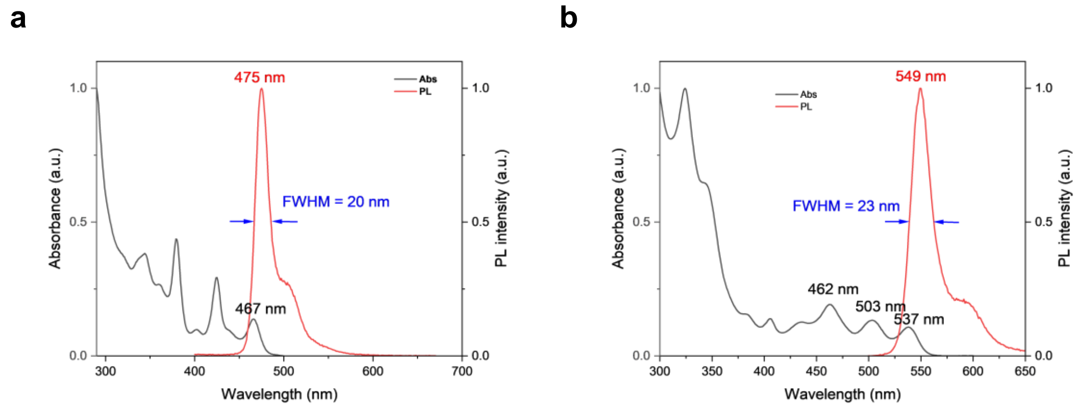

Fig. S44. UV-Vis absorption and emission spectra of **9c** (a) and **10c** (b) in toluene.

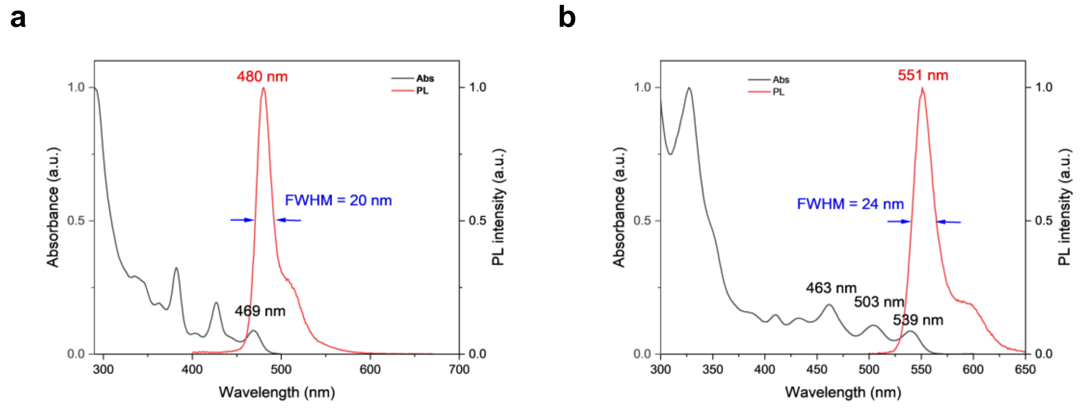

Fig. S45. UV-Vis absorption and emission spectra of **9d** (a) and **10d** (b) in toluene.

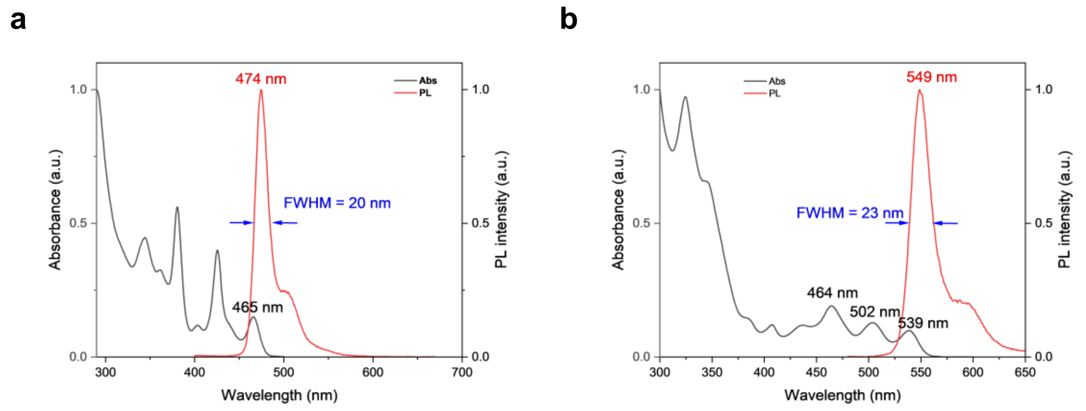

Fig. S46. UV-Vis absorption and emission spectra of **9e** (a) and **10e** (b) in toluene.

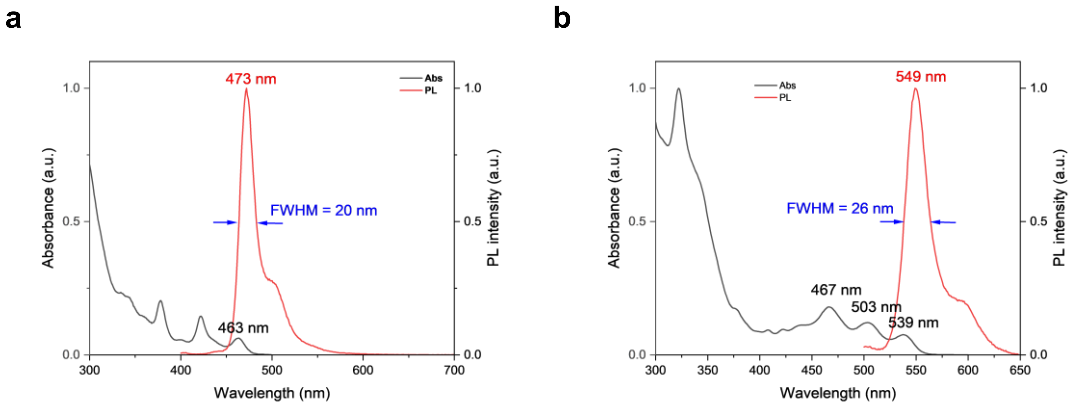

Fig. S47. UV-Vis absorption and emission spectra of **9f** (a) and **10f** (b) in toluene.

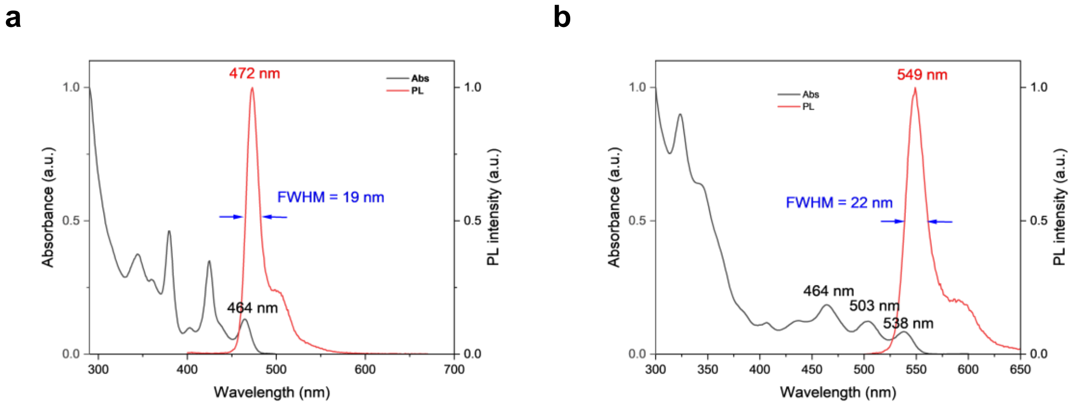

Fig. S48. UV-Vis absorption and emission spectra of **9g** (a) and **10g** (b) in toluene.

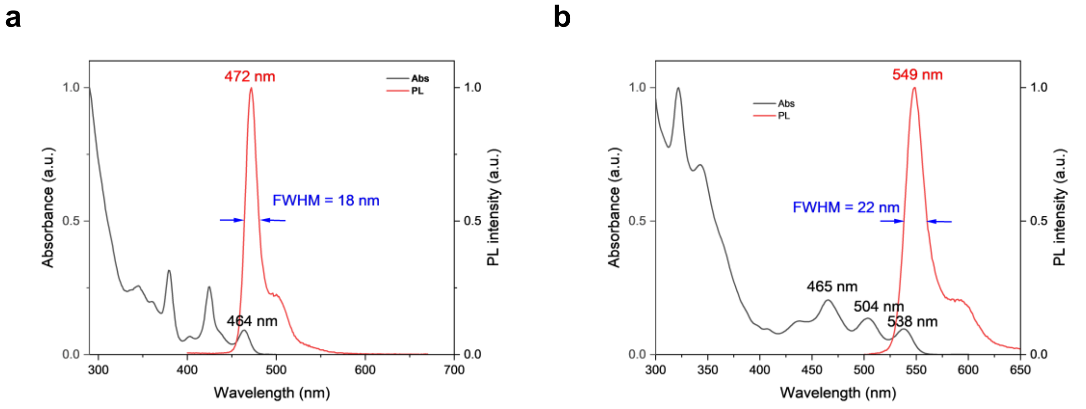

Fig. S49. UV-Vis absorption and emission spectra of **9h** (a) and **10h** (b) in toluene.

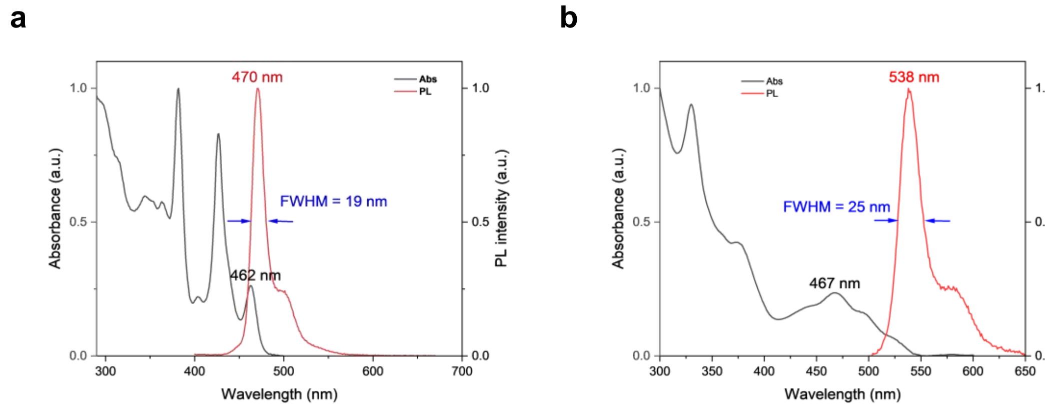

Fig. S50. UV-Vis absorption and emission spectra of **9i** (a) and **10i** (b) in toluene.

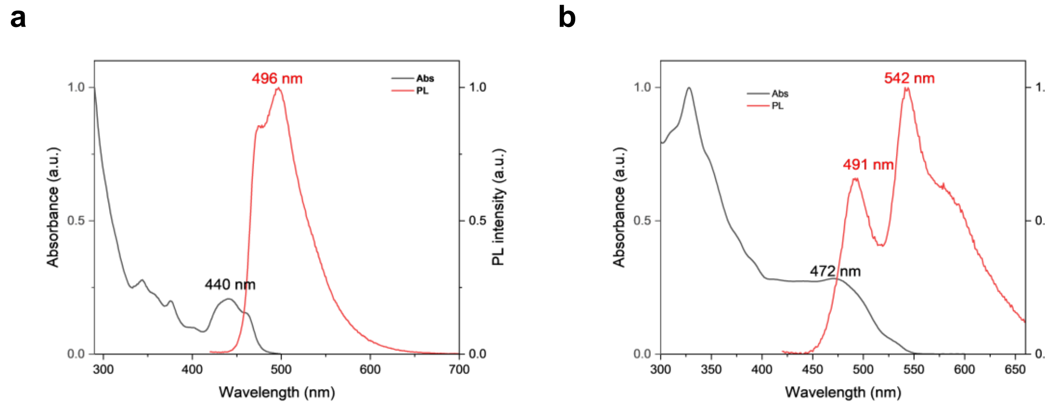

Fig. S51. UV-Vis absorption and emission spectra of **9j** (a) and **10j** (b) in toluene.

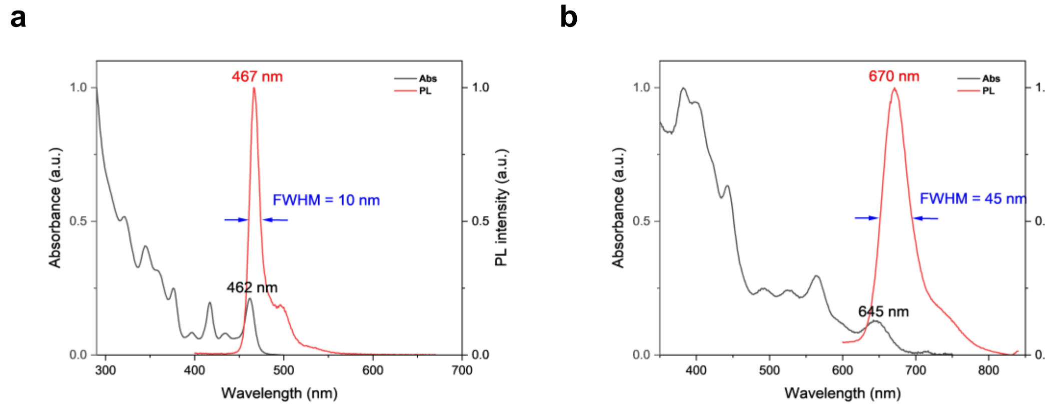

Fig. S52. UV-Vis absorption and emission spectra of **11** (a) and **12** (b) in toluene.

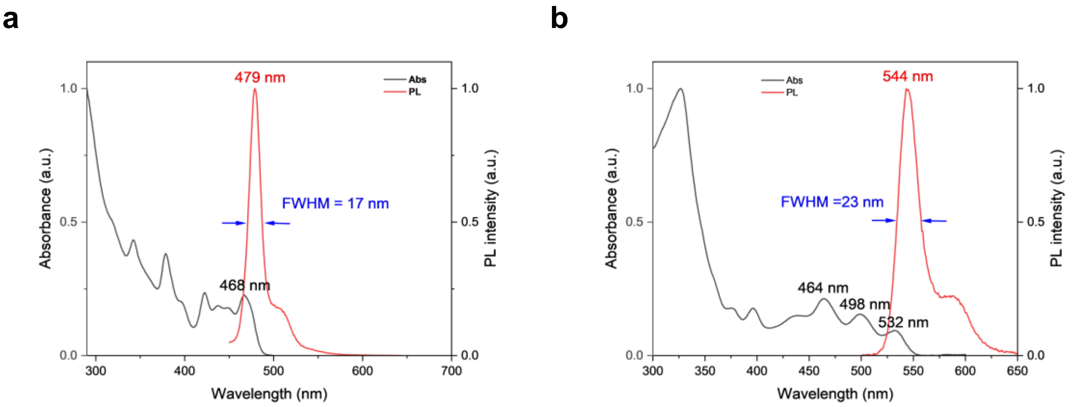

Fig. S53. UV-Vis absorption and emission spectra of **13** (a) and **14** (b) in toluene.

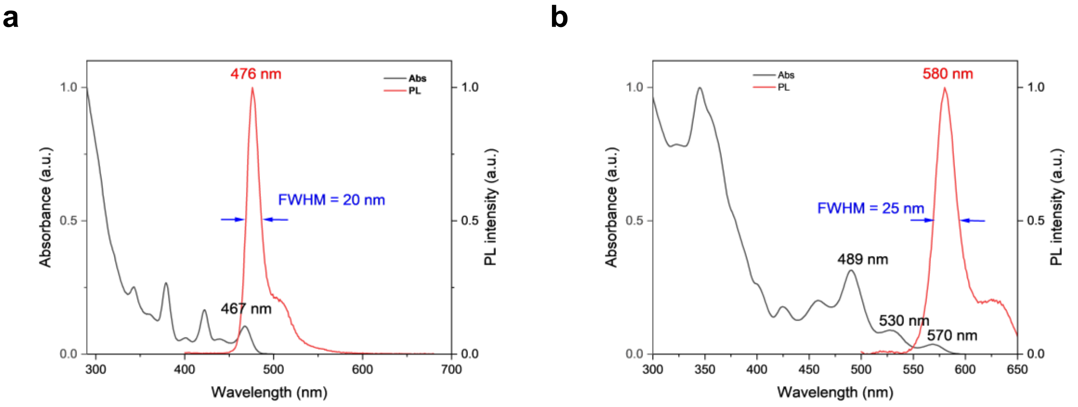

Fig. S54. UV-Vis absorption and emission spectra of **15a** (a) and **16a** (b) in toluene.

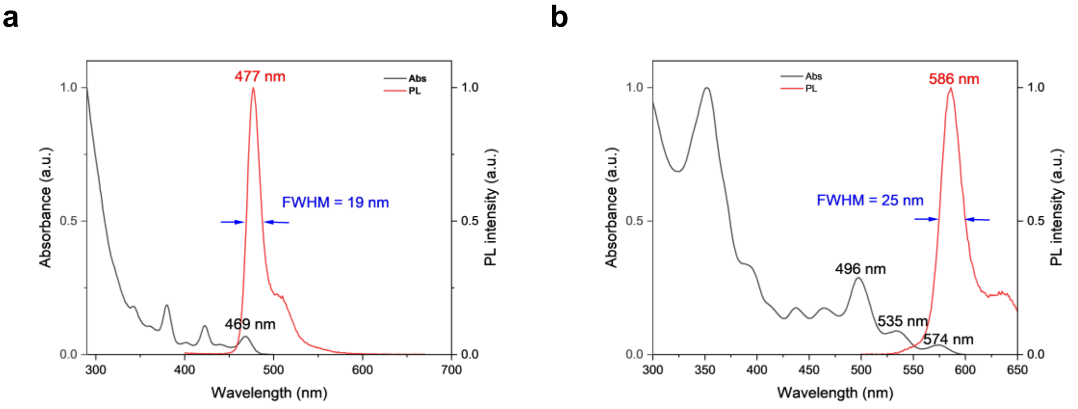

Fig. S55. UV-Vis absorption and emission spectra of **15b** (a) and **16b** (b) in toluene.

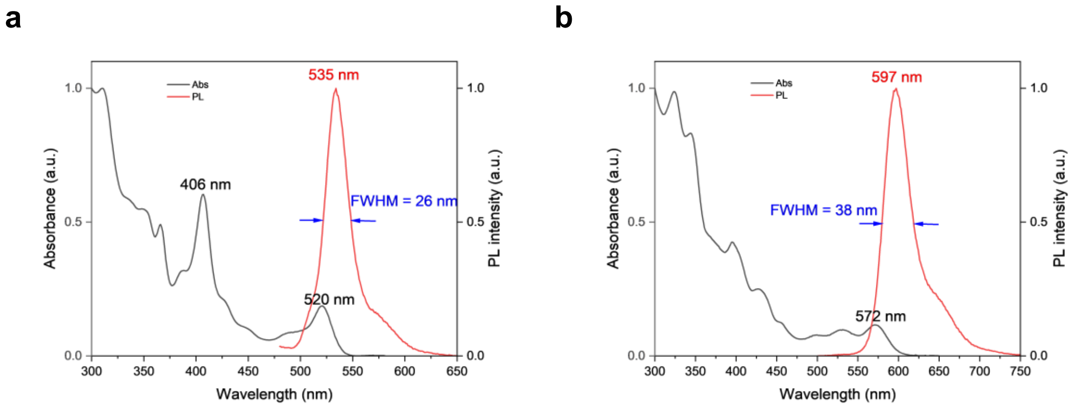

Fig. S56. UV-Vis absorption and emission spectra of **18** (a) and **19** (b) in toluene.

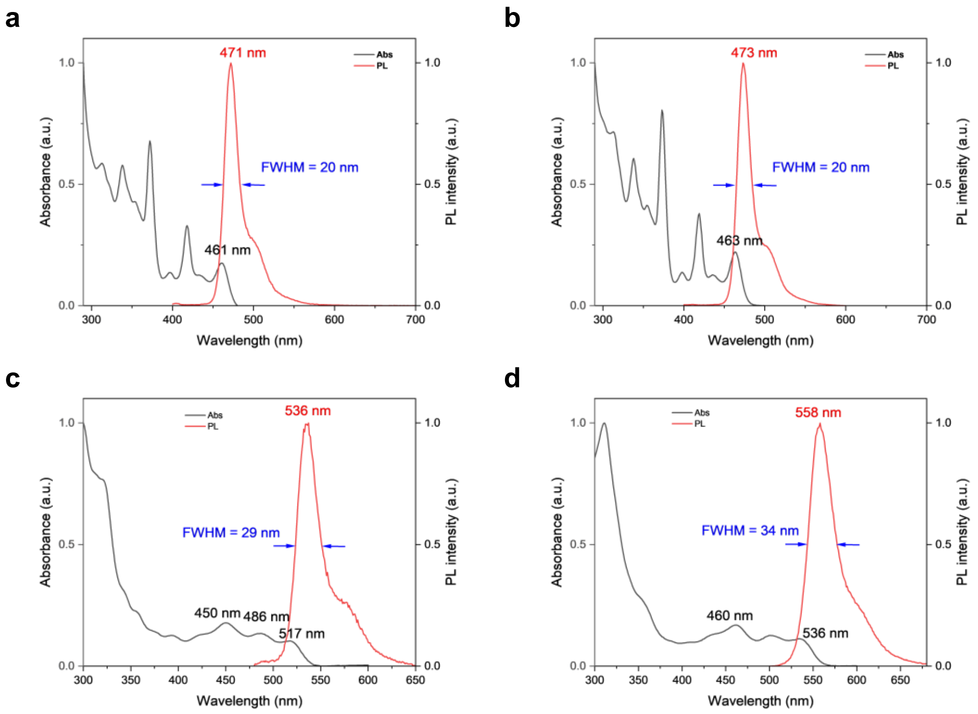

Fig. S57. UV-Vis absorption and emission spectra of **20** (a), **21** (b), **22** (c), and **23** (d) in toluene.

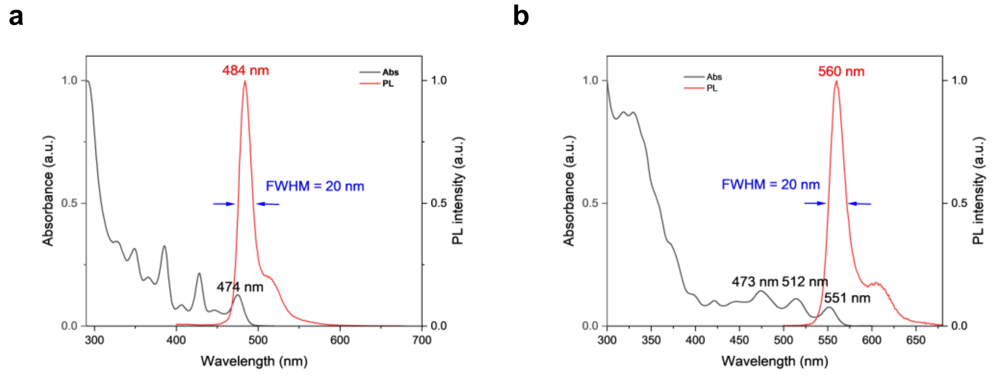

Fig. S58. UV-Vis absorption and emission spectra of **24a** (a) and **25a** (b) in toluene.

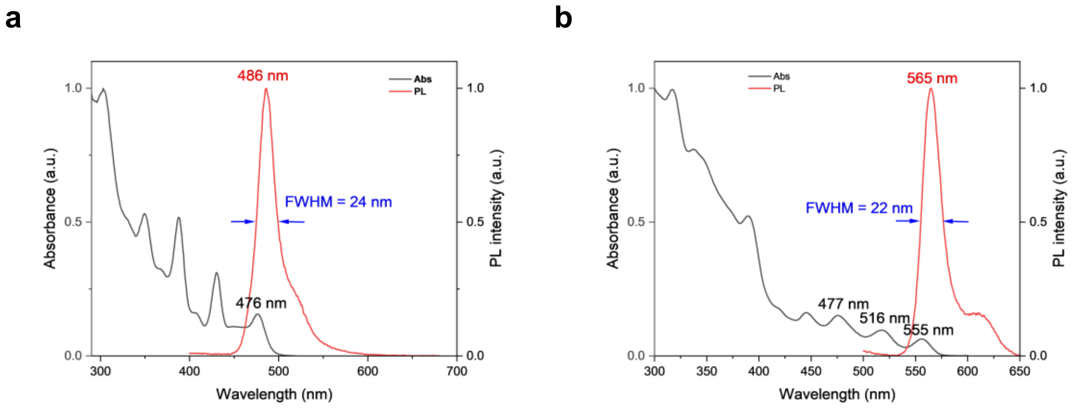

Fig. S59. UV-Vis absorption and emission spectra of **24b** (a) and **25b** (b) in toluene.

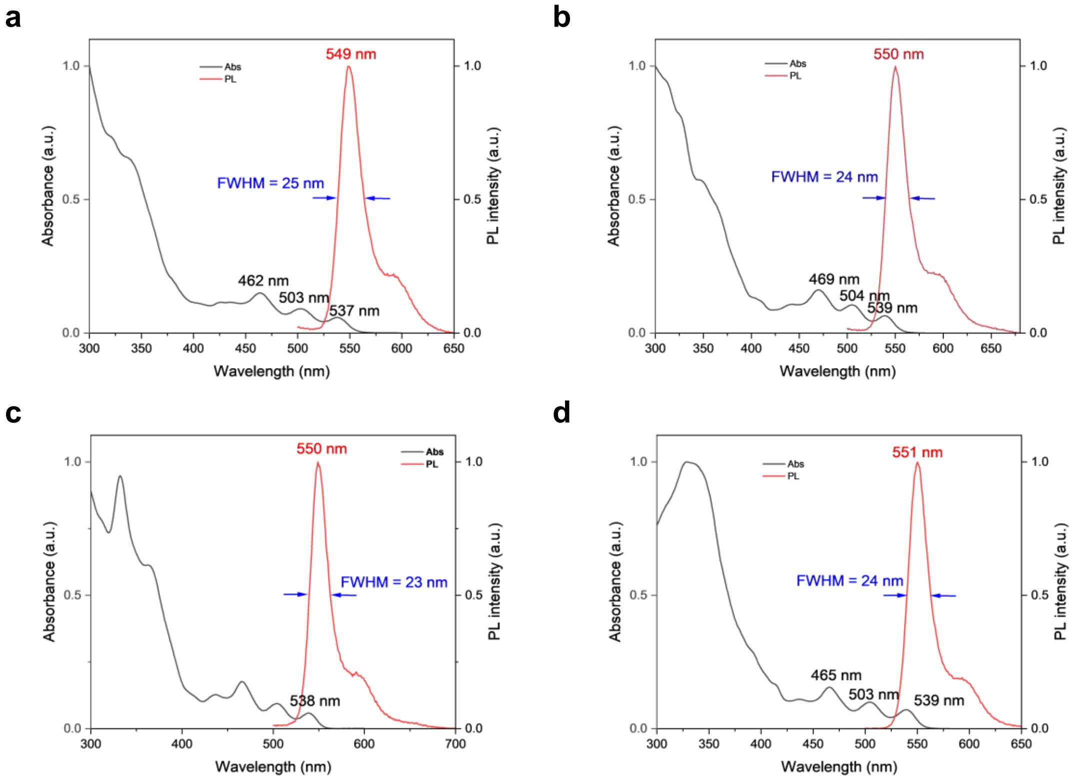

Fig. S60. UV-Vis absorption and emission spectra of **32** (a), **33** (b), **34** (c), and **35** (d) in toluene.

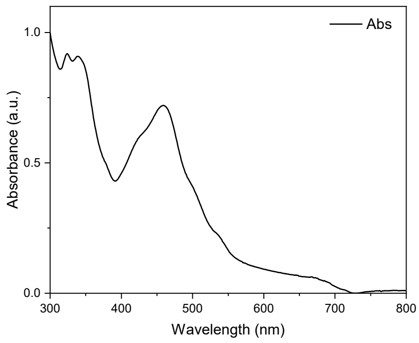

Fig. S61. UV-Vis absorption spectrum of **36** in toluene.

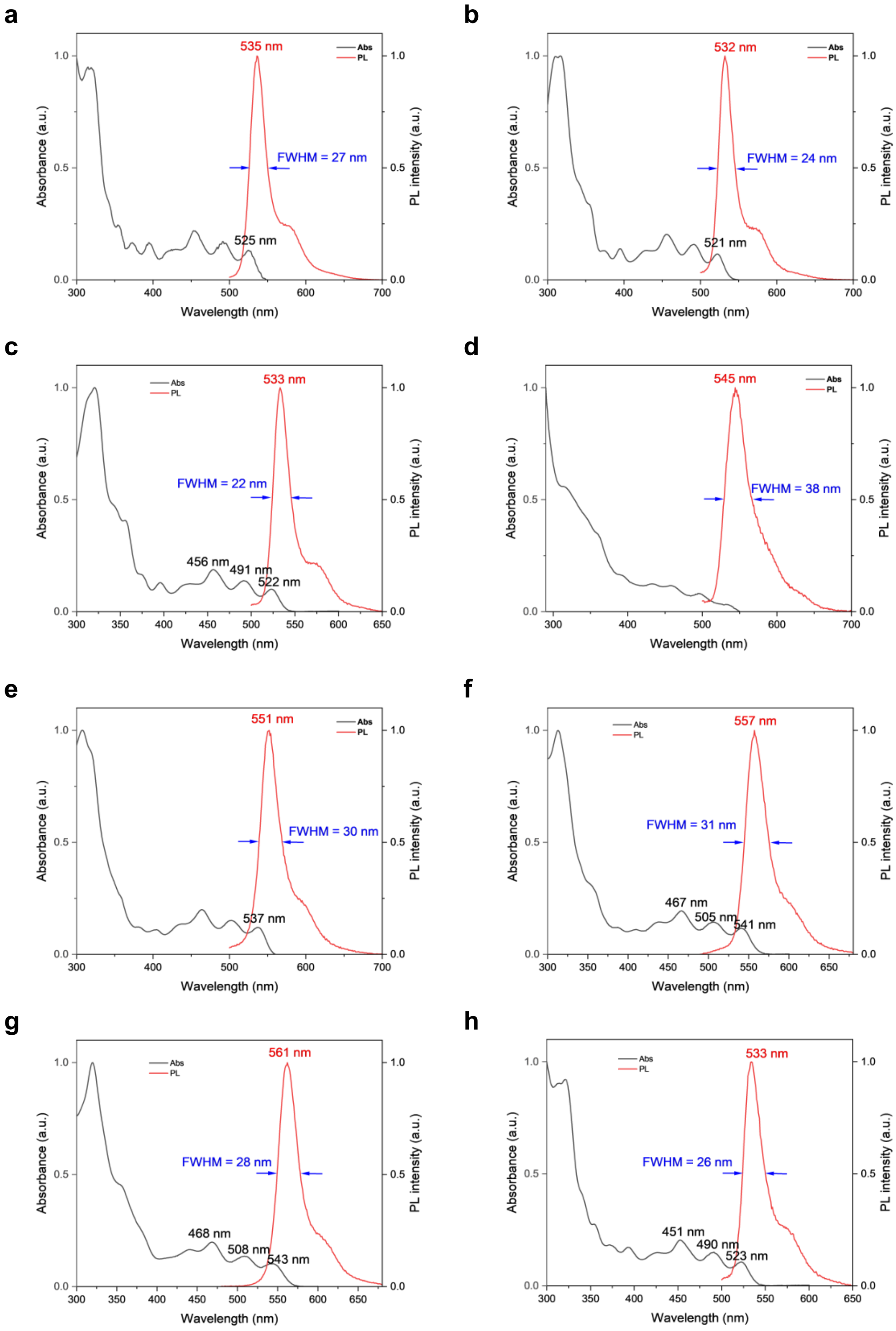

Fig. S62. UV-Vis absorption and emission spectra of **5c** (a), **26** (b), **27** (c), **28** (d), **29a** (e), **29b** (f), **30** (g), and **31** (h) in toluene.

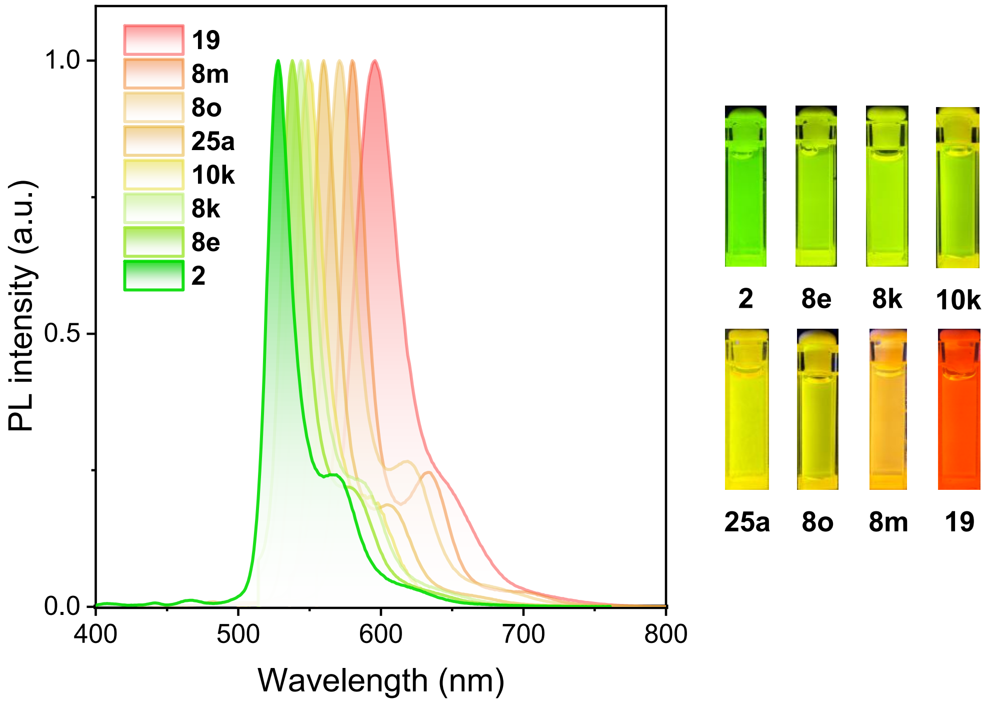

Fig. S63. Fluorescence spectra of representative products with images of toluene solutions under 365 nm UV light irradiation.

8.2 Quantum yields and lifetime

Table S19. Quantum yields of skeletal-edited products

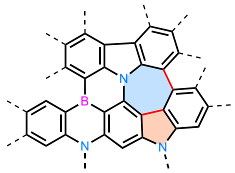

Compound name
Emission (nm) / FWHM (nm)
PLQY (%) (Excitation) (nm)
PLQY (%) (Excitation) (nm)

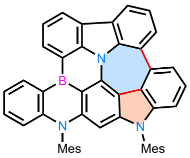

2
526 nm / 22 nm
85% (350 nm)
67% (390 nm)

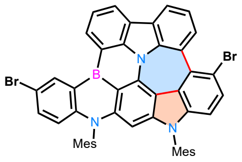

8a
538, 562 nm
5% (360 nm)
7% (390 nm)

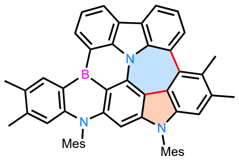

8b
529 nm / 25 nm
61% (350 nm)
51% (390 nm)

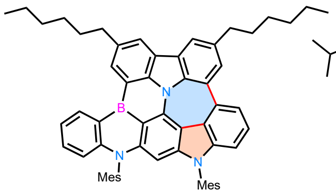

8c
532 nm / 23 nm
61% (350 nm)
53% (390 nm)

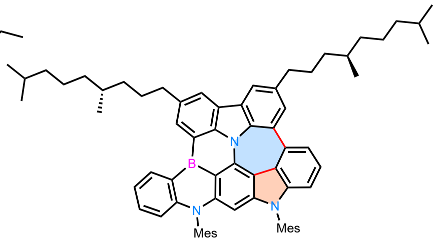

8d
533 nm / 23 nm
66% (350 nm)
59% (390 nm)

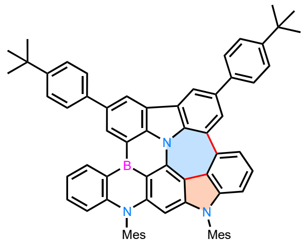

8e
538 nm / 22 nm
85% (350 nm)
67% (390 nm)

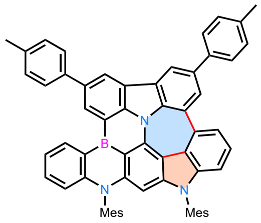

8f
538 nm / 21 nm
77% (360 nm)
66% (460 nm)

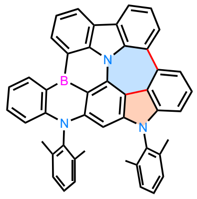

8g
527 nm / 23 nm
61% (350 nm)
56% (390 nm)

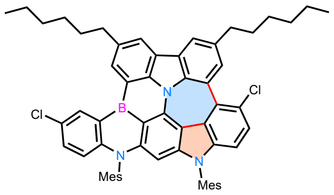

8h
540 nm / 25 nm

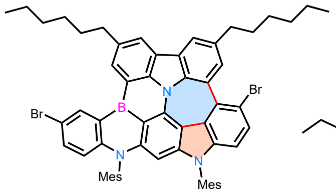

8i
542 nm / 23 nm
16% (350 nm)
12% (390 nm)

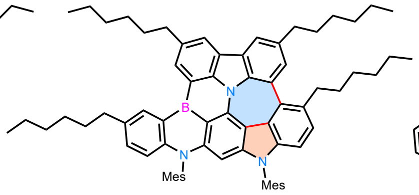

8j
533 nm / 23 nm
58% (360 nm)
58% (380 nm)

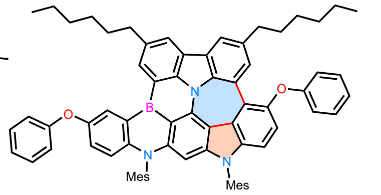

8k
545 nm / 24 nm
66% (350 nm)
47% (410 nm)

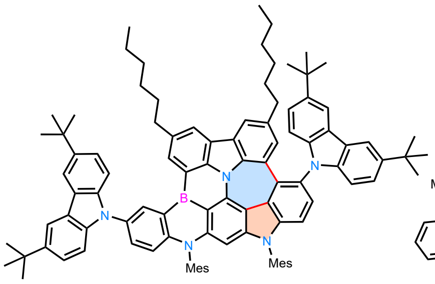

8l
539 nm / 26 nm
65% (360 nm)
60% (380 nm)

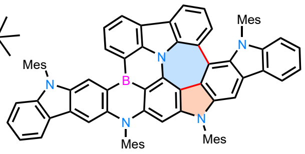

8m
580 nm / 21 nm
55% (360 nm)
48% (410 nm)

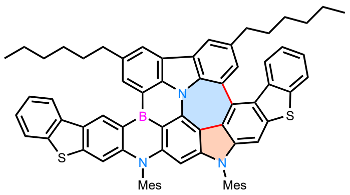

8n
566, 612 nm
17% (370 nm)
12% (430 nm)

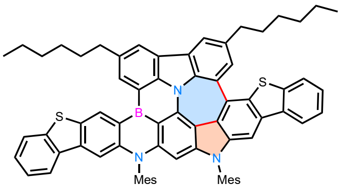

8o

570 nm / **26 nm**
41% (370 nm)
33% (410 nm)

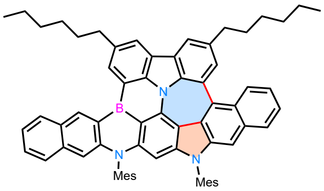

8p

585 nm / **40 nm**
16% (350 nm)
12% (450 nm)

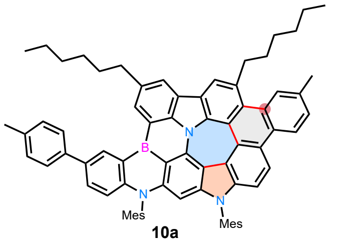

10a

549 nm / **23 nm**
>99% (350 nm)
82% (390 nm)

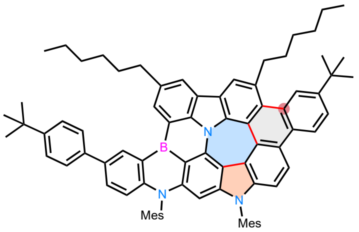

10b

549 nm / **24 nm**
>99% (350 nm)
81% (400 nm)

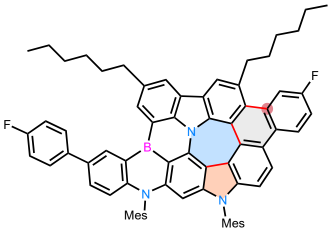

10c

549 nm / **23 nm**
95% (350 nm)
72% (410 nm)

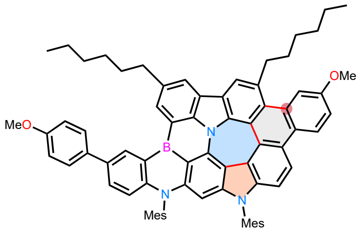

10d

551 nm / **24 nm**
98% (350 nm)
80% (390 nm)

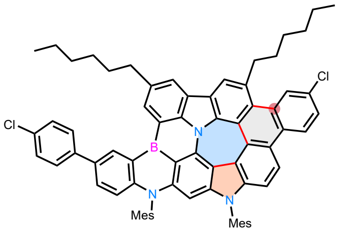

10e

549 nm / **23 nm**
91% (350 nm)
73% (390 nm)

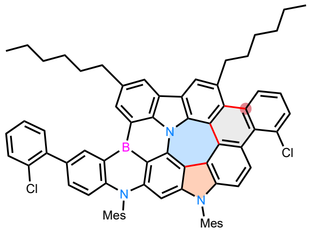

10f

549 nm / **26 nm**
42% (350 nm)
34% (420 nm)

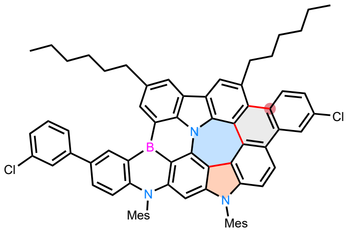

10g

549 nm / **22 nm**
84% (350 nm)
66% (390 nm)

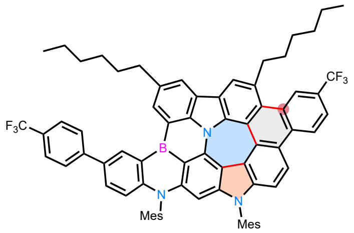

10h

549 nm / **22 nm**
96% (350 nm)
77% (390 nm)

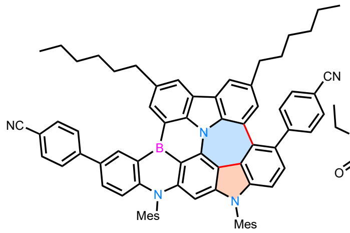

10i

538 nm / **25 nm**
47% (350 nm)
42% (380 nm)

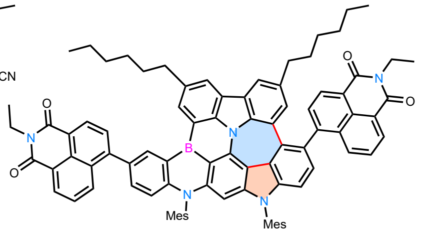

10j

491, 542 nm
29% (360 nm)
23% (420 nm)

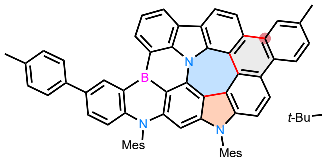

10k

549 nm / **22 nm**
>99% (350 nm)

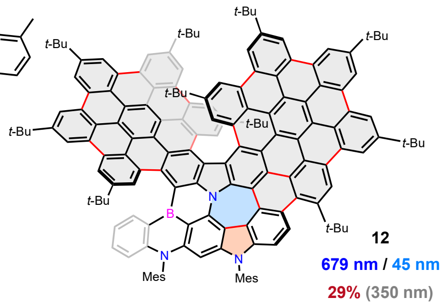

12

679 nm / **45 nm**
29% (350 nm)
24% (410 nm)

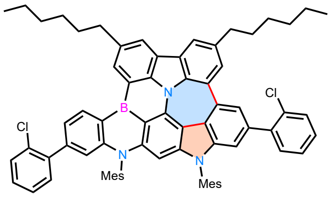

14

544 nm / **23 nm**
79% (360 nm)
65% (440 nm)

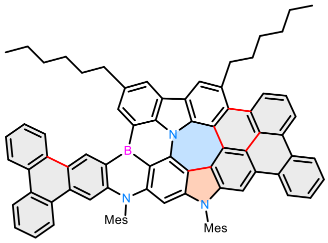

16a

580 nm / 25 nm
53% (420 nm)
53% (480 nm)

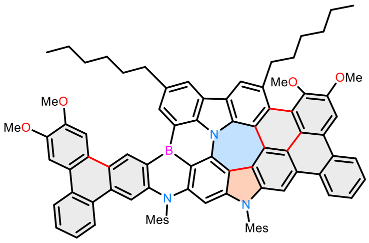

16b

586 nm / 25 nm
57% (360 nm)
49% (400 nm)

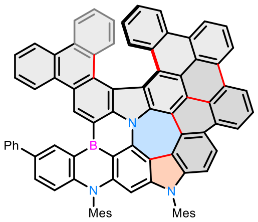

19

597 nm / 38 nm
56% (350 nm)
47% (400 nm)

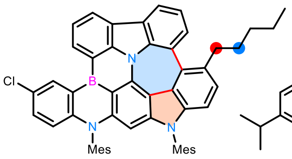

22

536 nm / 29 nm
64% (360 nm)
56% (400 nm)

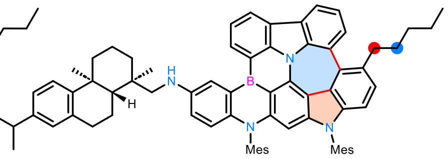

23

558 nm / 34 nm
85% (350 nm)
62% (390 nm)

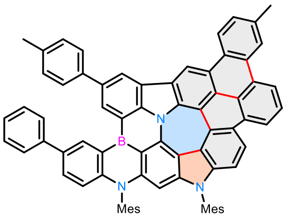

25a

560 nm / 20 nm
90 % (360 nm)
68 % (460 nm)

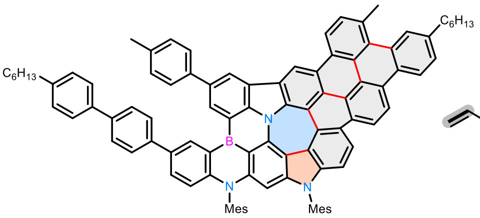

25b

565 nm / 22 nm
56% (360 nm)
50% (390 nm)

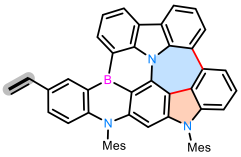

26

532 nm / 24 nm

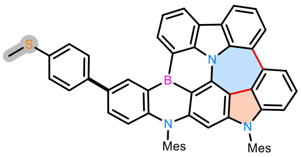

27

533 nm / 22 nm
85% (350 nm)
66% (400 nm)

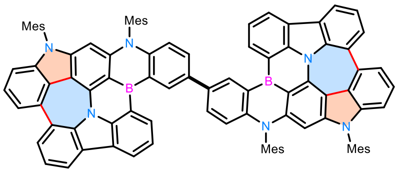

28

545 nm / 38 nm
64% (360 nm)
51% (380 nm)

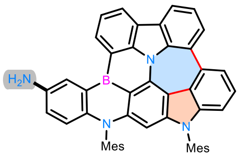

29a

551 nm / 30 nm
72% (350 nm)
52% (400 nm)

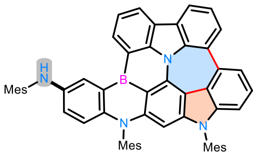

29b

557 nm / 31 nm
90% (350 nm)
66% (410 nm)

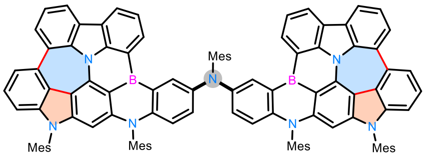

30

561 nm / 28 nm
80% (350 nm)
58% (400 nm)

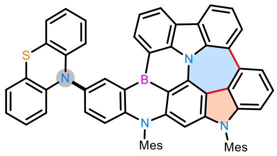

31

533 nm / 26 nm
70% (360 nm)
62% (390 nm)

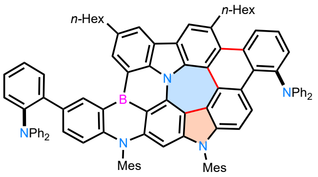

32

549 nm / 25 nm

89% (360 nm)

74% (410 nm)

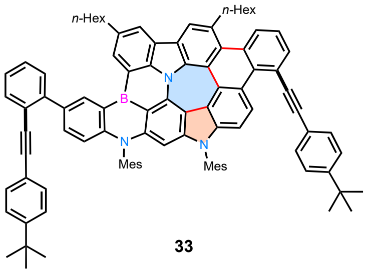

33

550 nm / 24 nm

54% (360 nm)

49% (390 nm)

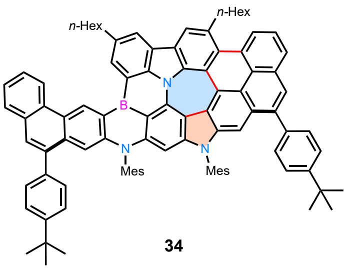

34

550 nm / 23 nm

88% (350 nm)

76% (390 nm)

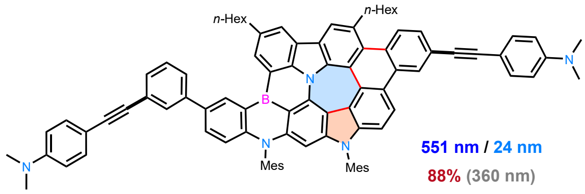

35

551 nm / 24 nm

88% (360 nm)

76% (400 nm)

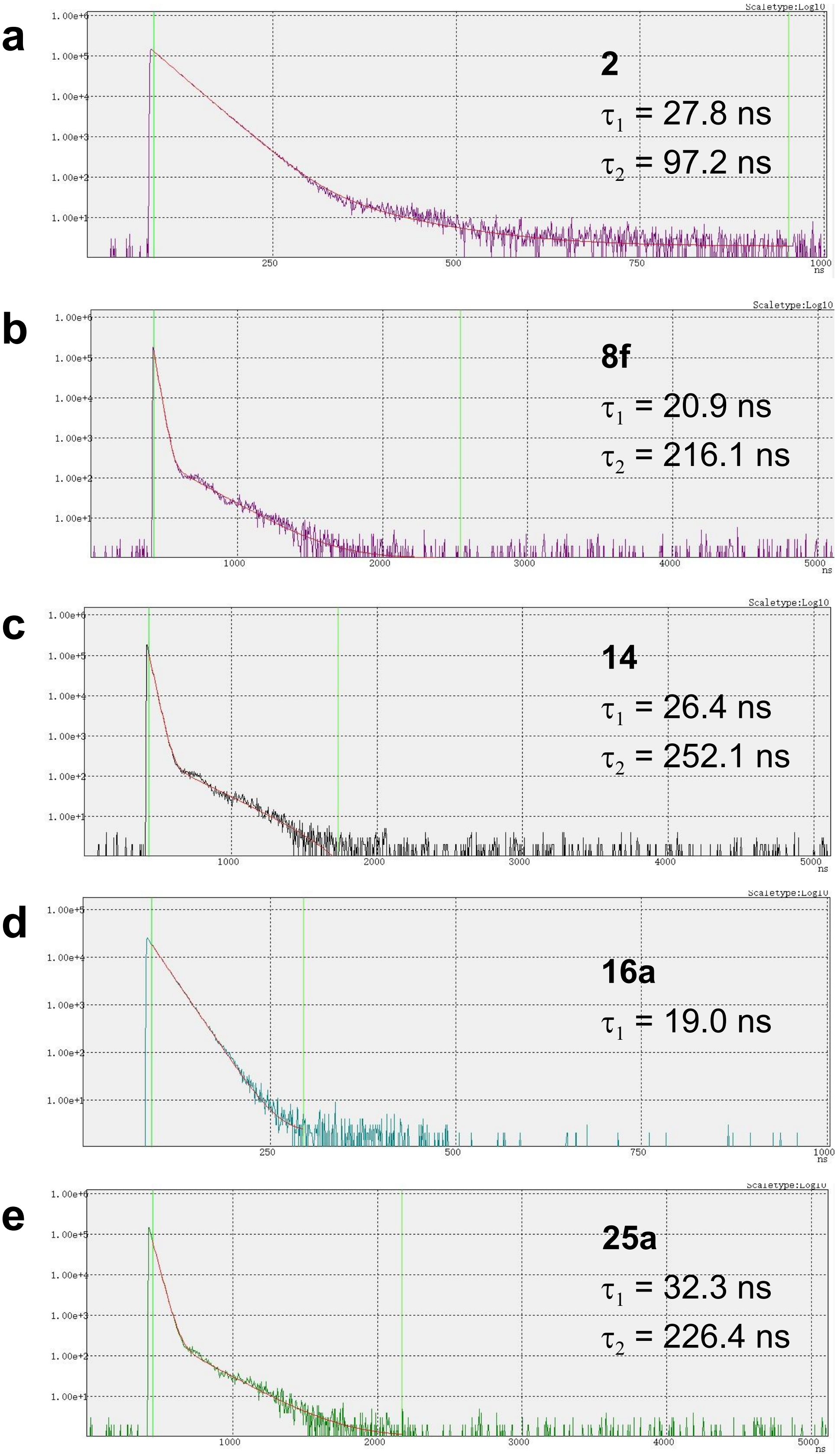

Fig. S64. Lifetime of prompt fluorescence and delayed fluorescence of **2** (a), **8f** (b), **14** (c) and **25a** (e). Lifetime of prompt fluorescence of **16a** (d). Measured in degassed toluene solution (~10^{-5} M).

8.3 Electrochemical properties

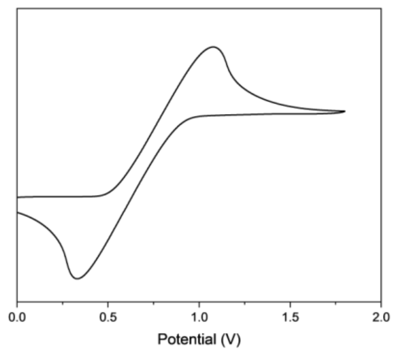

Fig. S65. Cyclic voltammogram (CV) of ferrocene in 0.1 M n-Bu_4NPF_6/dichloromethane, scan rate: 100 mV/s for CV, room temperature).

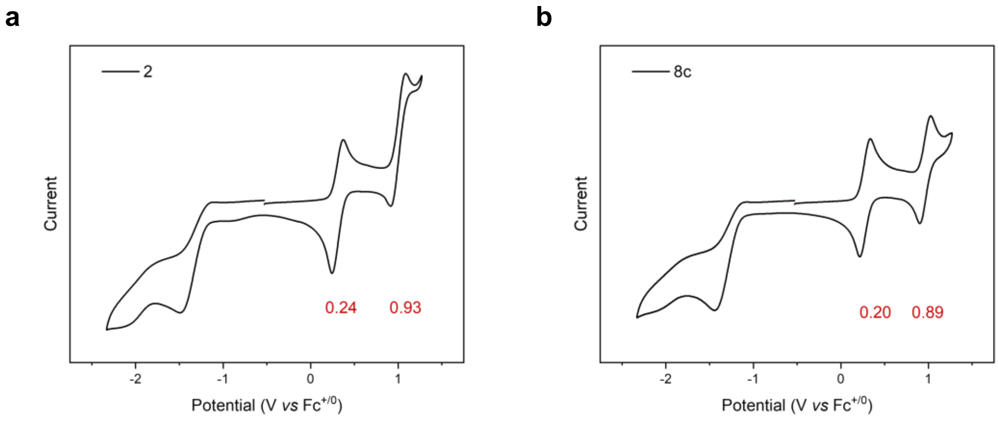

Fig. S66. Cyclic voltammograms (CVs) of **2** (a) and **8c** (b) (V *vs* $Fc^{+/0}$) in 0.1 M n-Bu_4NPF_6/dichloromethane, scan rate: 100 mV/s for CV, room temperature).

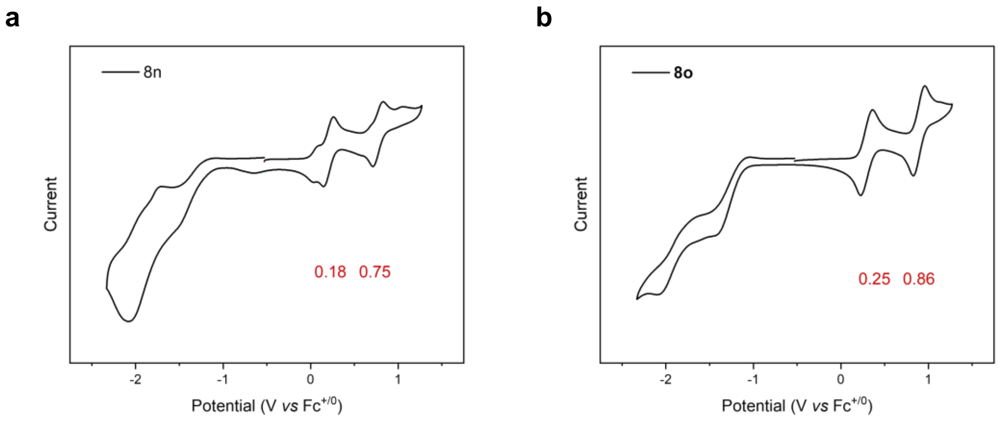

Fig. S67. Cyclic voltammograms (CVs) of **8n** (a) and **8o** (b) (V *vs* $Fc^{+/0}$) in 0.1 M n-Bu_4NPF_6/dichloromethane, scan rate: 100 mV/s for CV, room temperature).

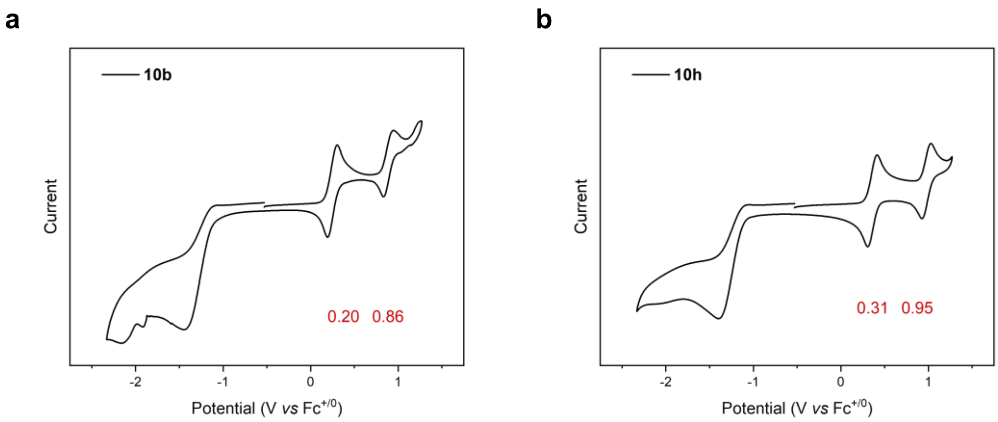

Fig. S68. Cyclic voltammograms (CVs) of **10b** (a) and **10h** (b) (V *vs* $Fc^{+/0}$) in 0.1 M *n*-Bu_4NPF_6/dichloromethane, scan rate: 100 mV/s for CV, room temperature).

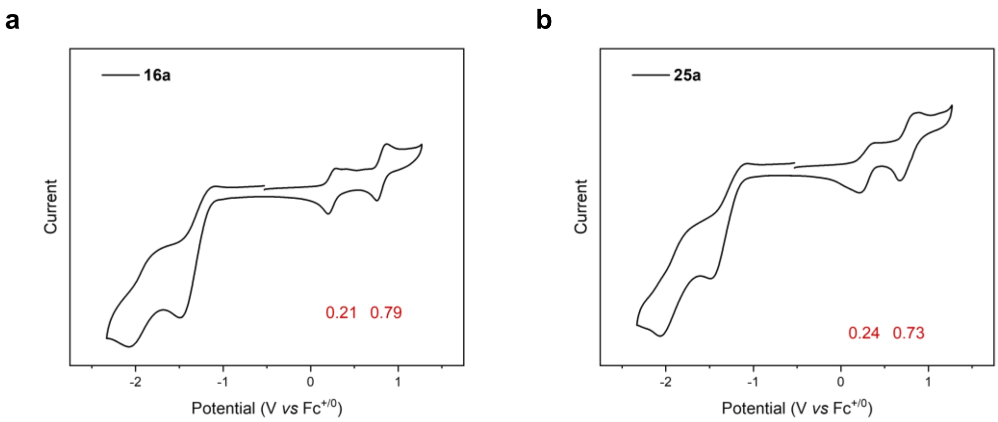

Fig. S69. Cyclic voltammograms (CVs) of **16a** (a) and **25a** (b) (V *vs* $Fc^{+/0}$) in 0.1 M *n*-Bu_4NPF_6/dichloromethane, scan rate: 100 mV/s for CV, room temperature).

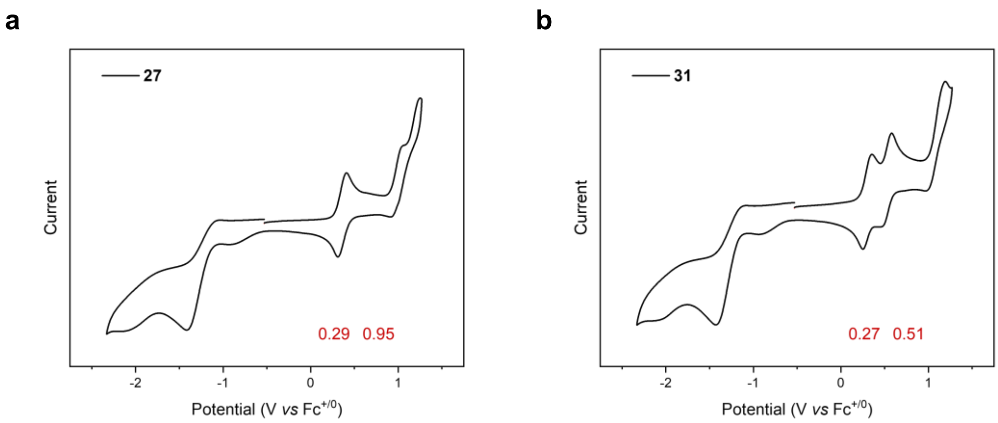

Fig. S70. Cyclic voltammograms (CVs) of **27** (a) and **31** (b) (V *vs* $Fc^{+/0}$) in 0.1 M *n*-Bu_4NPF_6/dichloromethane, scan rate: 100 mV/s for CV, room temperature).

9. Device performances

Device fabrication and measurement of EL characteristics:

All compounds were purified by temperature-gradient sublimation under high vacuum prior to device fabrication. Organic light-emitting diodes (OLEDs) were fabricated on pre-patterned indium-tin-oxide (ITO)-coated glass substrates. The device structure consisted of multiple organic layers sandwiched between the bottom transparent ITO anode and the top reflective metal cathode.

Before thin-film deposition, the ITO substrates underwent a rigorous cleaning procedure. All organic layers were subsequently deposited via thermal evaporation in a high-vacuum chamber with a base pressure maintained below 1×10^{-6} Torr. The deposition system allowed for the sequential fabrication of the complete device stack in a single pump-down cycle without breaking the vacuum. The deposition rates for the organic materials were controlled at 0.1–0.2 nm/s. For the emissive layer, dopant molecules were co-evaporated from independent sources, with their evaporation rates precisely calibrated to achieve the desired doping concentration.

The current density–voltage–luminance (J–V–L) characteristics of the OLEDs were measured using a Keithley 2400 source meter. Simultaneously, the electroluminescence (EL) spectra and the external quantum efficiency (EQE) were characterized with an absolute EQE measurement system (Hamamatsu C9920-12) incorporating an integrating sphere and a photonic multichannel analyzer (PMA-12, C10027-02), which has a detection wavelength range extending to 1100 nm.

Table S20. Summary of EL data of devices based on emitters **10k** with different doping concentrations.

x wt%	λ_{EL} (nm) ^{a}	FWHM (nm)	V_{on} (V) ^{b}	EQE (%) ^{c}	PE_{max} (lm W-1)	CIE (x,y) $^{a)}$
1	548	29	2.6	23/15.8/13.5	87.7	(0.39, 0.59)
2	549	29	2.6	20.7/12.9/11.5	80.2	(0.41, 0.58)
3	551	29	3.0	19.5/11.4/9.7	61.2	(0.42, 0.57)

^{a}Values recorded at a luminance around 1000 cd m^{-2}.

^{b}Turn-on voltage at the luminance of 1 cd m^{-2}.

^{c}Maximum EQE, along with the values at 1000 and 5000 cd m^{-2}.

10. Preparation of substrates

10.1 Synthesis of substrates

General one-shot borylation reaction for the synthesis of substrates:

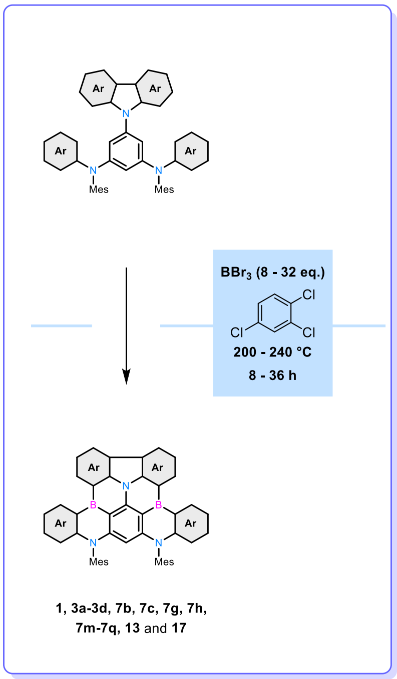

The starting material (1.0 equiv.) was added to a 120 mL thick-wall vial (*synthware*, P260006F), dried under vacuum while heated by a heating gun. After cooling to room temperature, TCB (25% wt) was added under nitrogen flow. BBr_3 (8 – 32 equiv.) was finally injected within ca. 2 min. The vial was sealed and stirred for 30 min at rt. Then it was moved to a sand bath, and heated to 200 – 240 °C for 8 – 36 hours. After cooling to rt, the reaction mixture was carefully poured into MeOH with rigorous stirring. The solid residue was further dissolved in minimum amount of DCM, transferred to the suspension [*If no precipitation, the solvent was removed, and the residue was further purified by column chromatography using hexane and DCM as the eluents*]. The mixture was filtered. The filter cake was further washed with DCM/MeOH (v/v = 1:20), EtOH, MeOH. The solid was dried under vacuum to give analytical pure sample [*If necessary, column chromatography using hexane and DCM as the eluents might be further applied to obtain pure sample*]. Compounds **1**, **3a-3d**, **7b**, **7c**, **7g**, **7h**, **7m-7q**, **13**, and **17**were synthesized by this method.

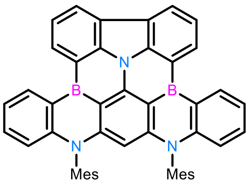

1

1 (7.23 g, 88%) was synthesized from compound **S2** (8.0 g).

^{1}H NMR (500 MHz, $CDCl_3$) δ 9.22 (dd, *J* = 7.7, 1.7 Hz, 2H), 9.14 (d, *J* = 7.5 Hz, 2H), 8.52 (dd, *J* = 7.5, 0.9 Hz, 2H), 7.82 (t, *J* = 7.5 Hz, 2H), 7.49 (ddd, *J* = 8.5, 6.9, 1.6 Hz, 2H), 7.39 – 7.34 (m, 2H), 6.95 (s, 4H), 6.85 (dd, *J* = 8.6, 1.0 Hz, 2H), 5.36 (s, 1H), 2.44 (s, 6H), 1.73 (s, 12H).

^{13}C NMR (101 MHz, $CDCl_3$) δ 150.15, 146.04, 142.98, 141.81, 138.16, 136.73, 136.31,

135.64, 133.07, 131.82, 129.79, 129.77, 125.37, 123.90, 123.23, 120.60, 115.89, 90.42, 21.40, 17.23.

HRMS (MALDI-TOF) m/z: $[M]^+$ Calculated for $C_{48}H_{37}B_2N_3$ 677.3183; Found 677.3183.

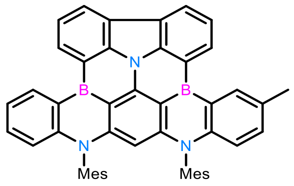

3a

3a (690 mg, 90%) was synthesized from compound **S5** (750 mg).

^{1}H NMR (400 MHz, $CDCl_3$) δ 9.31 – 9.08 (m, 3H), 9.01 (s, 1H), 8.51 (d, *J* = 7.4 Hz, 2H), 7.82 (s, 2H), 7.49 (s, 1H), 7.40 – 7.29 (m, 2H), 6.95 (s, 4H), 6.84 (d, *J* = 8.5 Hz, 1H), 6.76 (d, *J* = 8.5 Hz, 1H), 5.33 (s, 1H), 2.59 (s, 3H), 2.44 (s, 6H), 1.73 (s, 12H).

^{13}C NMR (101 MHz, $CDCl_3$) δ 150.04, 146.05, 144.07, 143.02, 141.76, 138.12, 138.08, 136.73, 136.40, 136.33, 135.64, 135.57, 133.47, 133.11, 133.02, 131.77, 131.20, 130.39, 129.78, 129.75, 129.56, 128.10, 126.23, 125.31, 123.82, 123.17, 123.13, 120.53, 115.83, 111.13, 90.19, 21.40, 21.27, 17.23, 17.21.

HRMS (MALDI-TOF) m/z: $[M]^+$ Calculated for $C_{49}H_{39}B_2N_3$ 691.3340; Found 691.3383 (6.2 ppm).

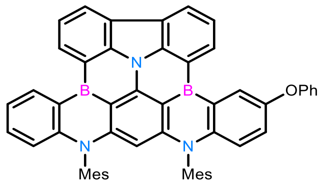

3b

3b (140 mg, 34%) was synthesized from compound **S6** (400 mg).

^{1}H NMR (400 MHz, $CDCl_3$) δ 9.22 (d, *J* = 7.5 Hz, 1H), 9.13 (d, *J* = 7.6 Hz, 1H), 8.92 (d, *J* = 7.6 Hz, 1H), 8.84 (d, *J* = 2.8 Hz, 1H), 8.49 (t, *J* = 7.2 Hz, 2H), 7.81 (t, *J* = 7.5 Hz, 1H), 7.73 (t, *J* = 7.5 Hz, 1H), 7.50 (t, *J* = 8.0 Hz, 1H), 7.40 – 7.35 (m, 2H), 7.21 (dd, *J* = 9.1, 2.8 Hz, 1H), 7.17 – 7.06 (m, 3H), 6.96 (s, 4H), 6.86 (d, *J* = 8.8 Hz, 2H), 5.35 (s, 1H), 2.44 (s, 6H), 1.75 – 1.74 (m, 12H).

^{13}C NMR (101 MHz, $CDCl_3$) δ 158.65, 150.21, 150.05, 150.01, 146.05, 143.05, 142.56, 141.81, 141.75, 138.28, 138.19, 136.74, 136.71, 136.34, 136.32, 135.65, 133.13, 132.76, 131.86, 129.88, 129.85, 129.81, 125.42, 125.38, 125.03, 124.02, 123.97, 123.33, 122.70, 120.65, 118.06, 117.45, 115.91, 90.22, 21.41, 17.27, 17.24.

HRMS (MALDI-TOF) m/z: $[M]^+$ Calculated for $C_{54}H_{41}B_2N_3O$ 769.3447; Found 769.3517 (9.1 ppm).

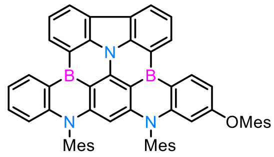

3b'

3b’ (260 mg, 26%) was synthesized from compound **S22** (1.0 g) at 190 °C.

^{1}H NMR (400 MHz, $CDCl_3$) δ 9.21 (dd, J = 7.7, 1.7 Hz, 1H), 9.12 (d, J = 7.5 Hz, 1H), 9.02 (dd, J = 15.1, 8.0 Hz, 2H), 8.49 (t, J = 6.9 Hz, 2H), 7.79 (dt, J = 13.3, 7.5 Hz, 2H), 7.49 (ddd, J = 8.5, 6.9, 1.6 Hz, 1H), 7.36 (t, J = 7.2 Hz, 1H), 6.93 (s, 2H), 6.85 (m, 5H), 6.74 (dd, J = 8.4, 2.4 Hz, 1H), 6.19 (d, J = 2.3 Hz, 1H), 5.34 (s, 1H), 2.41 (s, 3H), 2.39 (s, 3H), 2.29 (s, 3H), 2.04 (s, 6H), 1.72 (s, 6H), 1.70 (s, 6H).

^{13}C NMR (101 MHz, $CDCl_3$) δ 161.48, 150.18, 149.94, 148.81, 148.17, 146.05, 142.94, 141.88, 141.85, 138.17, 138.02, 137.25, 136.73, 136.36, 136.31, 136.04, 135.64, 134.56, 133.08, 132.96, 131.81, 131.01, 129.77, 129.60, 129.45, 125.35, 123.91, 123.77, 123.18, 123.14, 120.56, 115.89, 108.40, 101.58, 90.59, 21.36, 21.30, 20.90, 17.23, 17.10, 16.26.

HRMS (MALDI-TOF) m/z: $[M]^+$ Calculated for $C_{57}H_{47}B_2N_3O$ 811.3917; Found 811.3926 (1.1 ppm).

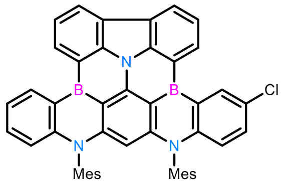

3c

3c (1.97 g, 77%) was synthesized from compound **S7** (2.50 g).

^{1}H NMR (400 MHz, $CDCl_3$) δ 9.22 (dd, J = 7.7, 1.7 Hz, 1H), 9.14 (d, J = 7.5 Hz, 1H), 9.11 (d, J = 2.6 Hz, 1H), 9.07 (d, J = 7.5 Hz, 1H), 8.52 (dd, J = 7.4, 3.2 Hz, 2H), 7.83 (td, J = 7.5, 4.5 Hz, 2H), 7.51 (ddd, J = 8.6, 6.9, 1.7 Hz, 1H), 7.44 – 7.36 (m, 2H), 6.96 (s, 4H), 6.86 (d, J = 8.6 Hz, 1H), 6.78 (d, J = 9.1 Hz, 1H), 5.35 (s, 1H), 2.44 (s, 6H), 1.72 (s, 12H).

^{13}C NMR (101 MHz, $CDCl_3$) δ 150.31, 149.95, 145.97, 144.40, 142.91, 141.72, 141.62, 138.44, 138.24, 136.68, 136.60, 136.25, 136.02, 135.67, 134.64, 133.10, 132.63, 131.91, 131.56, 129.92, 129.82, 126.02, 125.39, 125.32, 124.12, 123.97, 123.36, 120.75, 117.47, 115.95, 90.41, 21.41, 17.22, 17.19.

HRMS (MALDI-TOF) m/z: $[M]^+$ Calculated for $C_{48}H_{36}B_2ClN_3$ 711.2778; Found 711.2835 (8.0 ppm).

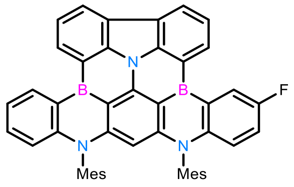

3d

3d (651 mg, 80%) was synthesized from compound **S8** (800 mg).

^{1}H NMR (500 MHz, $CDCl_3$) δ 9.21 (dd, *J* = 7.6, 1.6 Hz, 1H), 9.13 (d, *J* = 7.6 Hz, 1H), 9.05 (d, *J* = 7.5 Hz, 1H), 8.81 (dd, *J* = 9.2, 3.0 Hz, 1H), 8.50 (d, *J* = 7.4 Hz, 2H), 7.81 (t, *J* = 7.5 Hz, 2H), 7.50 (ddd, *J* = 8.6, 6.8, 1.7 Hz, 1H), 7.37 (dd, *J* = 8.6, 6.0 Hz, 1H), 7.23 – 7.19 (m, 1H), 6.96 (s, 4H), 6.88 – 6.84 (m, 1H), 6.82 (dd, *J* = 9.3, 4.5 Hz, 1H), 5.34 (s, 1H), 2.44 (s, 6H), 1.73 (s, 12H).

^{13}C NMR (101 MHz, $CDCl_3$) δ 158.55, 153.16, 150.28, 150.01, 146.02, 143.00, 142.45, 141.78, 138.36, 138.21, 136.70, 136.28, 135.66, 133.15, 132.52, 131.89, 129.90, 129.81, 125.38, 124.11, 123.99, 123.37, 120.70, 120.21, 119.94, 119.75, 119.45, 119.21, 117.43, 115.92, 111.09, 90.17, 53.58, 29.85, 21.41, 17.23, 17.20.

^{19}F NMR (471 MHz, $CDCl_3$) δ -123.82.

HRMS (MALDI-TOF) m/z: $[M]^+$ Calculated for $C_{48}H_{36}B_2FN_3$ 695.3089; Found 695.3153 (9.2 ppm).

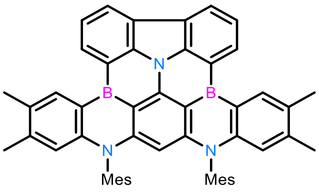

7b

7b (572 mg, 70%) was synthesized from compound **S9** (800 mg).

^{1}H NMR (600 MHz, $CDCl_3$) δ 9.13 (d, *J* = 7.4 Hz, 2H), 8.93 (s, 2H), 8.49 (dd, *J* = 7.4, 0.9 Hz, 2H), 7.81 (d, *J* = 7.4 Hz, 2H), 6.94 (s, 4H), 6.60 (s, 2H), 5.30 (s, 1H), 2.51 (s, 6H), 2.45 (s, 6H), 2.27 (s, 6H), 1.72 (s, 12H).

^{13}C NMR (101 MHz, $CDCl_3$) δ 149.84, 144.81, 144.60, 141.87, 141.25, 137.90, 136.75, 136.50, 136.07, 133.03, 132.95, 129.73, 128.94, 125.29, 123.68, 123.00, 116.39, 113.17, 90.18, 21.46, 21.11, 19.65, 17.28.

HRMS (MALDI-TOF) m/z: $[M]^+$ Calculated for $C_{52}H_{45}B_2N_3$ 733.3810; Found 733.3891 (11.0 ppm).

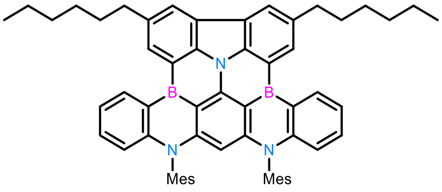

7c

7c (3.21 g, 87%) was synthesized from compound **S12** (3.6 g).

^{1}H NMR (400 MHz, $CDCl_3$) δ 9.21 (dd, *J* = 7.7, 1.7 Hz, 2H), 8.91 (d, *J* = 1.4 Hz, 2H), 8.33 (d, *J* = 1.3 Hz, 2H), 7.49 (ddd, *J* = 8.8, 7.0, 1.8 Hz, 2H), 7.41 – 7.35 (m, 2H), 6.95 (s, 4H), 6.84 (dd, *J* = 8.6, 1.1 Hz, 2H), 5.31 (s, 1H), 3.11 (t, *J* = 7.8 Hz, 4H), 2.44 (s, 6H), 1.93 (p, *J* = 7.6 Hz, 4H), 1.73 (s, 12H), 1.54 – 1.31 (m, 12H), 0.99 – 0.88 (m, 6H).

^{13}C NMR (101 MHz, $CDCl_3$) δ 150.21, 146.02, 142.52, 140.70, 138.09, 137.77, 136.79, 136.77, 136.38, 135.65, 133.00, 131.65, 129.76, 125.53, 124.25, 122.68, 120.50, 115.84, 90.03,

37.29, 32.96, 32.07, 29.32, 29.23, 22.87, 21.41, 17.27, 17.24, 14.34.

HRMS (MALDI-TOF) m/z: $[M]^+$ Calculated for $C_{60}H_{61}B_2N_3$ 845.5065; Found 845.5078 (1.5 ppm).

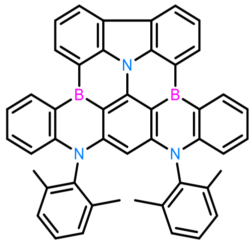

7g

7g (892 mg, 73%) was synthesized from compound **S14** (1.20 g).

^{1}H NMR (400 MHz, $CDCl_3$) δ 9.23 (dd, *J* = 7.6, 1.7 Hz, 2H), 9.17 – 9.10 (m, 2H), 8.51 (dd, *J* = 7.5, 0.9 Hz, 2H), 7.82 (t, *J* = 7.5 Hz, 2H), 7.50 (ddd, *J* = 8.6, 6.9, 1.7 Hz, 2H), 7.40 – 7.36 (m, 2H), 7.25 – 7.21 (m, 2H), 7.12 (d, *J* = 7.6 Hz, 4H), 6.82 (dd, *J* = 8.6, 1.1 Hz, 2H), 5.24 (s, 1H), 1.78 (s, 12H).

^{13}C NMR (101 MHz, $CDCl_3$) δ 150.06, 145.81, 141.84, 138.85, 137.07, 135.70, 133.12, 131.91, 131.25, 130.43, 129.32, 128.64, 128.14, 125.42, 123.98, 123.31, 120.73, 115.79, 90.14, 17.30.

HRMS (MALDI-TOF) m/z: $[M]^+$ Calculated for $C_{46}H_{33}B_2N_3$ 649.2870; Found 649.2948 (12.0 ppm).

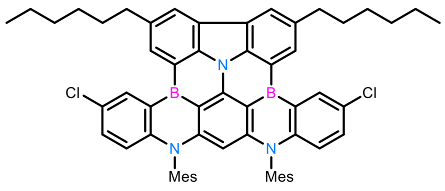

7h

7h (682 mg, 84%) was synthesized from compound **S15** (800 mg).

^{1}H NMR (400 MHz, $CDCl_3$) δ 9.10 (d, *J* = 2.6 Hz, 2H), 8.82 (d, *J* = 1.5 Hz, 2H), 8.36 (d, *J* = 1.3 Hz, 2H), 7.41 (dd, *J* = 9.1, 2.5 Hz, 2H), 6.95 (s, 4H), 6.78 (d, *J* = 9.1 Hz, 2H), 5.28 (s, 1H), 3.13 (t, *J* = 7.8 Hz, 4H), 2.43 (s, 6H), 1.94 (p, *J* = 7.7 Hz, 4H), 1.70 (s, 12H), 1.55 – 1.51 (m, , 4H), 1.49 – 1.34 (m, 8H), 0.94 (t, *J* = 7.1 Hz, 6H).

^{13}C NMR (101 MHz, $CDCl_3$) δ 150.19, 144.32, 142.38, 140.52, 138.45, 138.19, 136.59, 136.02, 134.67, 132.53, 131.50, 129.90, 127.78, 126.06, 125.63, 124.60, 122.24, 117.50, 111.22, 90.03, 37.17, 32.86, 32.05, 29.28, 22.87, 21.40, 17.18, 14.34.

HRMS (MALDI-TOF) m/z: $[M]^+$ Calculated for $C_{60}H_{59}B_2Cl_2N_3$ 913.4286; Found 913.4345 (6.4 ppm).

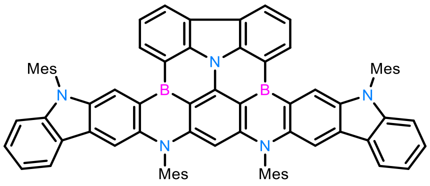

7m

7m (1.82 g, 89%) was synthesized from compound **S16** (2.00 g).

^{1}H NMR (400 MHz, $CDCl_3$) δ 8.76 (d, *J* = 7.5 Hz, 2H), 8.69 (s, 2H), 8.45 (d, *J* = 7.4 Hz, 2H), 8.03 (d, *J* = 7.7 Hz, 2H), 7.69 (t, *J* = 7.5 Hz, 2H), 7.56 (s, 2H), 7.40 (ddd, *J* = 8.2, 7.1, 1.2 Hz, 2H), 7.24 – 7.15 (m, 6H), 7.04 (s, 4H), 6.98 (d, *J* = 8.0 Hz, 2H), 5.37 (s, 1H), 2.54 (d, *J* = 3.9 Hz, 12H), 1.97 (s, 12H), 1.81 (s, 12H).

^{13}C NMR (101 MHz, $CDCl_3$) δ 150.19, 143.30, 142.43, 141.89, 140.53, 138.50, 138.00, 137.96, 137.06, 137.00, 136.17, 132.59, 132.19, 129.97, 129.73, 127.18, 126.98, 125.28, 123.54, 123.10, 122.71, 121.23, 118.77, 114.41, 110.11, 109.72, 106.30, 89.89, 21.58, 21.48, 17.93, 17.48.

HRMS (MALDI-TOF) m/z: $[M]^+$ Calculated for $C_{78}H_{63}B_2N_5$ 1091.5287; Found 1091.5355 (6.2 ppm).

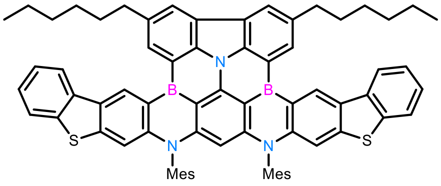

7n

7n (930 mg, 76%) was synthesized from compound **S17** (1.20 g).

^{1}H NMR (400 MHz, $CDCl_3$) δ 9.97 (s, 2H), 9.09 (s, 2H), 8.41 (d, *J* = 5.9 Hz, 4H), 7.83 (d, *J* = 7.9 Hz, 2H), 7.58 (t, *J* = 7.4 Hz, 2H), 7.46 (t, *J* = 7.4 Hz, 2H), 7.26 (s, 2H), 7.01 (s, 4H), 5.37 (s, 1H), 3.22 (t, *J* = 7.8 Hz, 4H), 2.50 (s, 6H), 2.13 – 1.98 (m, 4H), 1.78 (s, 12H), 1.64 – 1.60 (m, 4H), 1.54 – 1.39 (m, 8H), 0.96 (t, *J* = 7.0 Hz, 6H).

^{13}C NMR (101 MHz, $CDCl_3$) δ 150.45, 145.24, 144.05, 140.81, 138.83, 138.42, 137.83, 136.78, 136.34, 135.96, 132.74, 130.07, 129.61, 128.36, 126.28, 125.70, 124.83, 124.48, 122.86, 121.17, 108.48, 99.64, 90.74, 83.65, 37.20, 32.54, 32.07, 29.23, 22.83, 21.51, 17.33, 14.38.

HRMS (MALDI-TOF) m/z: $[M]^+$ Calculated for $C_{72}H_{65}B_2N_3S_2$ 1057.4822; Found 1057.4865 (4.0 ppm).

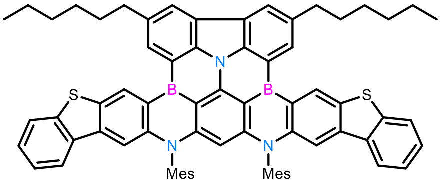

7o

7o (880 mg, 72%) was synthesized from compound **S18** (1.20 g).

¹H NMR (400 MHz, $CDCl_3$) δ 9.65 (s, 2H), 8.98 (s, 2H), 8.38 (s, 2H), 7.89 (dd, *J* = 7.8, 3.2 Hz, 4H), 7.56 (s, 2H), 7.47 (t, *J* = 7.5 Hz, 2H), 7.38 (t, *J* = 7.6 Hz, 2H), 7.04 (s, 4H), 5.38 (s, 1H), 3.19 (t, *J* = 9.1 Hz, 4H), 2.54 (s, 6H), 1.98 (p, *J* = 7.7 Hz, 4H), 1.79 (s, 12H), 1.59 – 1.55 (m, 4H), 1.53 – 1.35 (m, 8H), 0.96 (t, *J* = 7.0 Hz, 6H).

¹³C NMR (101 MHz, $CDCl_3$) δ 150.65, 143.69, 142.61, 141.38, 140.67, 138.81, 138.29, 138.06, 136.81, 136.43, 135.59, 133.12, 132.71, 131.98, 131.76, 131.21, 130.40, 130.04, 129.71, 129.43, 129.04, 128.11, 127.58, 126.99, 126.15, 125.91, 125.66, 124.37, 124.11, 123.17, 122.58, 122.18, 110.76, 107.68, 90.18, 37.27, 33.03, 32.09, 29.38, 22.90, 21.58, 17.37, 14.37.

HRMS (MALDI-TOF) m/z: $[M]^+$ Calculated for $C_{72}H_{65}B_2N_3S_2$ 1057.4822; Found 1057.4938 (11.0 ppm).

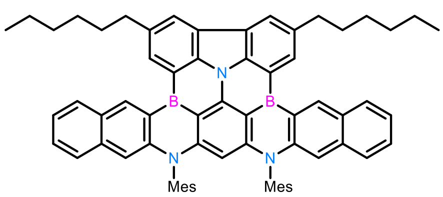

7p

7p (261 mg, 43%) was synthesized from compound **S19** (600 mg).

¹H NMR (400 MHz, $CDCl_3$) δ 9.76 (s, 2H), 9.08 (d, *J* = 1.4 Hz, 2H), 8.39 (d, *J* = 1.4 Hz, 2H), 8.24 – 8.18 (m, 2H), 7.69 – 7.65 (m, 2H), 7.49 – 7.42 (m, 4H), 7.15 (s, 2H), 7.00 (s, 4H), 5.33 (s, 1H), 3.19 (t, *J* = 7.7 Hz, 4H), 2.50 (s, 6H), 2.02 – 1.97 (m, 4H), 1.78 (s, 12H), 1.51 – 1.37 (m, 12H), 0.94 (t, *J* = 6.9 Hz, 6H).

¹³C NMR (101 MHz, $CDCl_3$) δ 151.62, 143.42, 142.74, 140.86, 138.13, 138.04, 136.86, 136.81, 136.71, 136.46, 135.56, 132.94, 129.95, 129.72, 128.99, 128.56, 127.26, 127.11, 125.72, 124.58, 123.66, 111.01, 90.65, 37.31, 32.89, 32.08, 29.34, 22.89, 21.53, 17.32, 14.36.、

HRMS (MALDI-TOF) m/z: $[M]^+$ Calculated for $C_{68}H_{65}B_2N_3$ 945.5379; Found 945.5316 (-6.7 ppm).

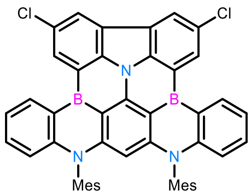

7q

7q (1.46 g, 95%) was synthesized from compound **S13** (1.50 g).

¹H NMR (400 MHz, $CDCl_3$) δ 9.05 (dd, *J* = 7.7, 1.7 Hz, 2H), 9.00 (d, *J* = 1.8 Hz, 2H), 8.30 (d, *J* = 1.8 Hz, 2H), 7.52 (ddd, *J* = 8.5, 6.9, 1.7 Hz, 2H), 7.39 (td, *J* = 8.2, 2.2 Hz, 2H), 6.96 (s, 4H), 6.86 (d, *J* = 8.5 Hz, 2H), 5.38 (s, 1H), 2.44 (s, 6H), 1.73 (s, 12H).

¹³C NMR (101 MHz, $CDCl_3/CS_2$) δ 149.89, 145.82, 142.60, 140.08, 138.11, 136.53, 136.44, 136.00, 135.26, 133.57, 133.17, 133.03, 132.17, 131.24, 131.08, 130.32, 129.77, 129.73, 129.48, 127.95, 125.66, 125.47, 124.13, 124.04, 120.92, 115.90, 111.09, 90.69, 21.39, 17.14.

HRMS (MALDI-TOF) m/z: $[M]^+$ Calculated for $C_{48}H_{35}B_2Cl_2N_3$ 745.2405; Found 745.2552

(19.7 ppm).

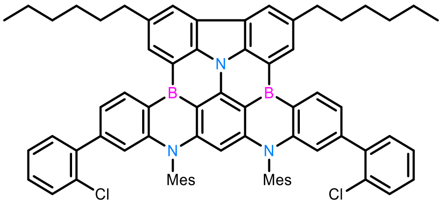

13

13 (1.12 g, 46%) was synthesized from compound **S20** (2.40 g).

^{1}H NMR (400 MHz, $CDCl_3$) δ 9.27 (d, *J* = 7.9 Hz, 2H), 8.93 (d, *J* = 1.4 Hz, 2H), 8.35 (d, *J* = 1.3 Hz, 2H), 7.45 – 7.42 (m, 4H), 7.37 (dd, *J* = 7.3, 2.1 Hz, 2H), 7.34 – 7.26 (m, 4H), 6.96 (d, *J* = 1.5 Hz, 2H), 6.90 (s, 4H), 5.30 (s, 1H), 3.13 (t, *J* = 7.7 Hz, 4H), 2.39 (s, 6H), 1.94 (p, *J* = 7.7 Hz, 5H), 1.76 (s, 12H), 1.54 – 1.34 (m, 12H), 0.93 (t, *J* = 7.0 Hz, 7H).

^{13}C NMR (101 MHz, $CDCl_3$) δ 150.43, 145.76, 142.36, 140.92, 140.73, 138.10, 137.84, 136.68, 136.13, 135.41, 133.01, 132.83, 131.58, 131.24, 130.43, 130.15, 128.65, 128.13, 126.91, 125.57, 124.33, 121.79, 116.90, 90.33, 37.30, 32.97, 32.08, 29.31, 22.87, 21.43, 17.29, 14.34.

HRMS (MALDI-TOF) m/z: $[M]^+$ Calculated for $C_{72}H_{67}B_2Cl_2N_3$ 1065.4915; Found 1065.4914.

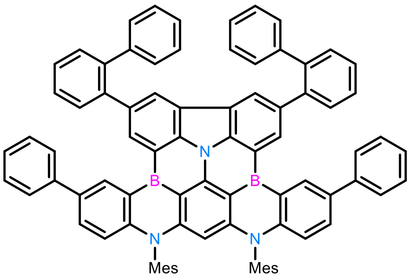

17

17 (345 mg, 68%) was synthesized from compound **S21** (500 mg).

^{1}H NMR (400 MHz, $CDCl_3$) δ 8.83 (d, *J* = 2.3 Hz, 2H), 8.79 (d, *J* = 1.4 Hz, 2H), 8.44 (d, *J* = 1.4 Hz, 2H), 7.79 (dd, *J* = 7.7, 1.7 Hz, 2H), 7.74 – 7.69 (m, 4H), 7.64 (dd, *J* = 8.8, 2.2 Hz, 2H), 7.62 – 7.52 (m, 10H), 7.46 – 7.41 (m, 2H), 7.32 – 7.28 (m, 4H), 6.96 (d, *J* = 5.8 Hz, 6H), 6.92 – 6.83 (m, 6H), 5.32 (s, 1H), 2.44 (s, 6H), 1.73 (s, 12H).

^{13}C NMR (101 MHz, $CDCl_3$) δ 149.95, 145.27, 142.78, 141.92, 141.76, 141.72, 141.35, 140.91, 138.24, 136.73, 136.68, 136.25, 135.75, 133.98, 133.38, 131.54, 131.23, 131.00, 130.85, 130.42, 130.24, 129.81, 128.85, 128.14, 127.91, 127.68, 127.58, 126.86, 126.70, 126.36, 125.54, 125.37, 122.19, 116.20, 111.29, 90.56, 21.43, 17.28.

HRMS (MALDI-TOF) m/z: $[M]^+$ Calculated for $C_{84}H_{61}B_2N_3$ 1133.5070; Found 1133.5177 (9.4 ppm).

General Suzuki-Miyaura coupling for the synthesis of precursors:

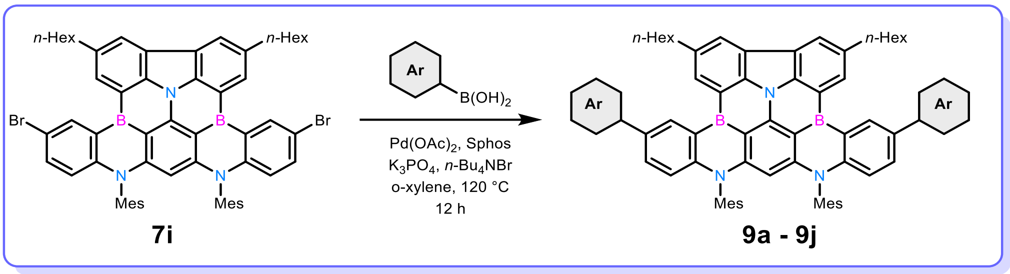

7i **9a - 9j**

Compound **7i** (1.0 equiv.) was added to a 120 mL Schlenk bottle. Boronic acids (4.0 equiv.), $Pd(OAc)_2$ (10 mol%), Sphos (15 mol%), *n*-Bu_4NBr (20 %) and K_3PO_4 (10.0 equiv.) were added. The mixture was dried under vacuum. After back-filled with nitrogen, *o*-xylene (20 mL) was added. The reaction mixture stirred at 120 °C for 12 hours. After cooling to rt, the mixture was passed through a pad of celite, rinsed with DCM. After removal of the solvent, DCM (5 mL) was added to dissolve the residue. MeOH was added to precipitate yellow solids, which were collected by filtration. Conpunds **9a-9j** were obtained.

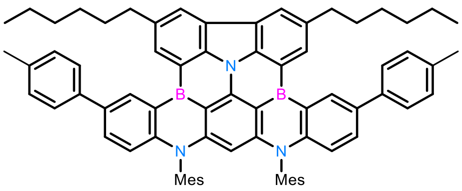

9a

9a (178 mg, 87%) was synthesized from compound **7i** (200 mg) with *p*-tolylboronic acid.

^{1}H NMR (400 MHz, $CDCl_3$) δ 9.43 (d, *J* = 2.3 Hz, 2H), 8.96 (d, *J* = 1.4 Hz, 2H), 8.35 (d, *J* = 1.3 Hz, 2H), 7.76 – 7.68 (m, 6H), 7.36 (d, *J* = 8.0 Hz, 4H), 6.97 (s, 4H), 6.90 (d, *J* = 8.8 Hz, 2H), 5.34 (s, 1H), 3.11 (t, *J* = 7.8 Hz, 4H), 2.46 (d, *J* = 4.8 Hz, 13H), 1.99 – 1.91 (m, 4H), 1.77 (s, 12H), 1.53 – 1.35 (m, 12H), 0.93 (t, *J* = 7.0 Hz, 6H).

^{13}C NMR (101 MHz, $CDCl_3$) δ 150.12, 145.27, 142.57, 140.76, 138.87, 138.19, 137.76, 136.79, 136.48, 136.35, 133.80, 133.04, 132.93, 130.50, 129.83, 129.80, 127.05, 126.69, 125.60, 124.37, 116.29, 90.19, 37.16, 32.58, 32.07, 29.87, 29.22, 22.83, 21.43, 21.30, 17.30, 14.34.

HRMS (MALDI-TOF) m/z: $[M]^+$ Calculated for $C_{74}H_{73}B_2N_3$ 1025.6007; Found 1025.6076 (6.7 ppm).

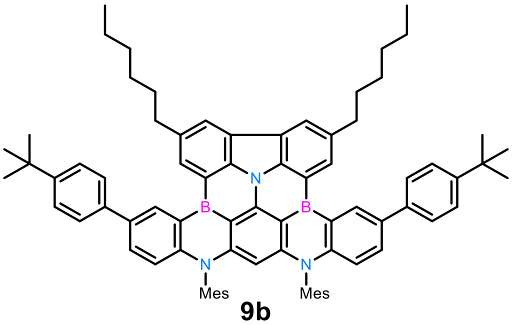

9b

9b (196 mg, 89%) was synthesized from compound **7i** (200 mg) with (4-(*tert*-butyl)phenyl)boronic acid.

^{1}H NMR (400 MHz, $CDCl_3$) δ 9.45 (d, *J* = 2.3 Hz, 2H), 8.98 (d, *J* = 1.4 Hz, 2H), 8.36 (d, *J* =

1.3 Hz, 2H), 7.80 – 7.70 (m, 6H), 7.61 – 7.56 (m, 4H), 6.97 (s, 4H), 6.90 (d, *J* = 8.8 Hz, 2H), 5.34 (s, 1H), 3.13 (t, *J* = 7.7 Hz, 4H), 2.45 (s, 6H), 1.98 (q, *J* = 7.6 Hz, 4H), 1.77 (s, 12H), 1.60 – 1.51 (m, 8H), 1.41 – 1.37 (m, 4H), 0.94 (t, *J* = 7.0 Hz, 6H).

^{13}C NMR (101 MHz, $CDCl_3$) δ 150.10, 149.70, 145.29, 140.76, 138.82, 138.18, 137.77, 136.80, 136.38, 133.82, 132.95, 130.57, 129.79, 126.81, 126.06, 125.60, 124.36, 116.27, 90.19, 37.13, 34.71, 32.60, 32.07, 31.60, 29.87, 29.22, 22.82, 21.43, 17.31, 14.36.

HRMS (MALDI-TOF) m/z: $[M]^+$ Calculated for $C_{80}H_{85}B_2N_3$ 1109.6947; Found 1109.6939 (-0.7 ppm).

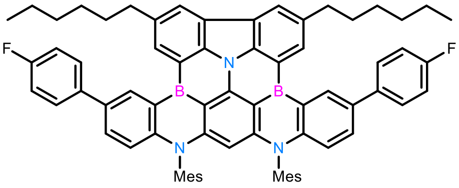

9c

9c (174 mg, 84%) was synthesized from compound **7i** (200 mg) with (4-fluorophenyl)boronic acid.

^{1}H NMR (400 MHz, $CDCl_3$) δ 9.38 (d, *J* = 2.3 Hz, 2H), 8.92 (s, 2H), 8.44 – 8.24 (m, 2H), 7.84 – 7.63 (m, 6H), 7.26 – 7.20 (m, 4H), 6.99 (s, 4H), 6.92 (d, *J* = 8.8 Hz, 2H), 5.36 (s, 1H), 3.10 (t, *J* = 7.7 Hz, 4H), 2.46 (s, 6H), 1.94 (p, *J* = 7.5 Hz, 4H), 1.78 (s, 12H), 1.55 – 1.33 (m, 12H), 0.93 (t, *J* = 6.9 Hz, 6H).

^{13}C NMR (101 MHz, $CDCl_3$) δ 163.54, 161.10, 150.15, 145.38, 142.52, 140.73, 138.28, 137.85, 137.80, 136.73, 136.25, 133.89, 132.80, 132.17, 130.43, 129.84, 129.71, 129.04, 128.63, 128.55, 126.71, 126.16, 125.92, 125.63, 124.46, 122.60, 116.40, 116.03, 115.82, 111.30, 90.26, 37.11, 32.48, 32.04, 29.16, 22.80, 21.44, 17.28, 14.32.

^{19}F NMR (377 MHz, $CDCl_3$) δ -116.71.

HRMS (MALDI-TOF) m/z: $[M]^+$ Calculated for $C_{72}H_{67}B_2F_2N_3$ 1033.5505; Found 1033.5574 (6.7 ppm).

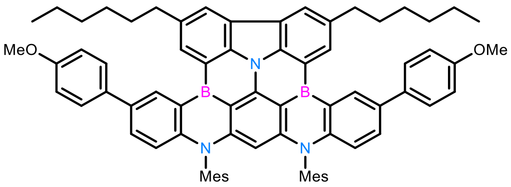

9d

9d (166 mg, 79%) was synthesized from compound **7i** (200 mg) with (4-methoxyphenyl)boronic acid.

^{1}H NMR (400 MHz, $CDCl_3$) δ 9.39 (d, *J* = 2.3 Hz, 2H), 8.95 (d, *J* = 1.4 Hz, 2H), 8.35 (d, *J* = 1.3 Hz, 2H), 7.78 – 7.73 (m, 4H), 7.68 (dd, *J* = 8.8, 2.2 Hz, 2H), 7.11 – 7.07 (m, 4H), 6.97 (s, 4H), 6.89 (d, *J* = 8.8 Hz, 2H), 5.33 (s, 1H), 3.91 (s, 6H), 3.12 (t, *J* = 7.7 Hz, 4H), 2.45 (s, 6H), 1.94 (q, *J* = 7.7 Hz, 4H), 1.77 (s, 12H), 1.53 – 1.33 (m, 12H), 0.93 (t, *J* = 7.0 Hz, 6H).

^{13}C NMR (101 MHz, $CDCl_3$) δ 158.89, 150.09, 145.06, 140.77, 138.18, 137.74, 136.79,

136.36, 134.35, 133.50, 132.93, 132.78, 130.33, 129.80, 128.16, 125.60, 124.36, 116.30, 114.56, 90.14, 55.55, 37.16, 32.56, 32.06, 29.20, 22.82, 21.43, 17.30, 14.35.

HRMS (MALDI-TOF) m/z: $[M]^+$ Calculated for $C_{74}H_{73}B_2N_3O_2$ 1057.5905; Found 1057.5918 (1.2 ppm).

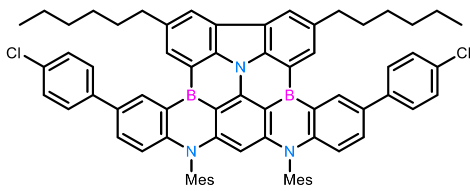

9e

9e (186 mg, 87%) was synthesized from compound **7i** (200 mg) with (4-chlorophenyl)boronic acid (reaction temperature: 90 °C).

^{1}H NMR (400 MHz, $CDCl_3$) δ 9.39 (d, *J* = 2.3 Hz, 2H), 8.92 (s, 2H), 8.35 (s, 2H), 7.77 – 7.72 (m, 4H), 7.68 (dd, *J* = 8.8, 2.3 Hz, 2H), 7.51 (d, *J* = 8.2 Hz, 4H), 6.98 (s, 4H), 6.92 (d, *J* = 8.9 Hz, 2H), 5.35 (s, 1H), 3.11 (t, *J* = 7.7 Hz, 4H), 2.46 (s, 6H), 1.94 (p, *J* = 7.6 Hz, 4H), 1.77 (s, 12H), 1.54 – 1.33 (m, 12H), 0.93 (t, *J* = 6.9 Hz, 6H).

^{13}C NMR (101 MHz, $CDCl_3$) δ 150.16, 145.59, 142.49, 140.74, 140.16, 138.33, 137.86, 136.71, 136.21, 133.95, 132.84, 132.80, 131.87, 130.34, 129.85, 129.22, 129.13, 128.38, 128.34, 127.66, 127.56, 125.66, 124.52, 122.56, 116.47, 90.36, 58.65, 37.13, 32.50, 32.08, 32.06, 29.18, 22.82, 21.44, 18.59, 17.30, 17.27, 14.33.

HRMS (MALDI-TOF) m/z: $[M]^+$ Calculated for $C_{72}H_{67}B_2Cl_2N_3$ 1065.4915; Found 1065.5002 (8.1 ppm).

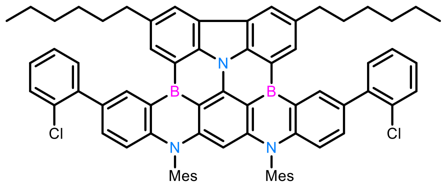

9f

9f (143 mg, 67%) was synthesized from compound **7i** (200 mg) with (2-chlorophenyl)boronic acid (reaction temperature: 90 °C).

^{1}H NMR (400 MHz, $CDCl_3$) δ 9.37 (s, 2H), 8.90 (s, 2H), 8.33 (s, 2H), 7.60 (t, *J* = 6.7 Hz, 6H), 7.42 (t, *J* = 7.5 Hz, 2H), 7.34 (t, *J* = 7.7 Hz, 2H), 6.97 (s, 4H), 6.90 (d, *J* = 8.7 Hz, 2H), 5.35 (s, 1H), 3.08 (t, *J* = 7.7 Hz, 4H), 2.45 (s, 6H), 1.91 – 1.88 (m, 4H), 1.78 (s, 12H), 1.54 – 1.31 (m, 12H), 0.90 (t, *J* = 6.9 Hz, 6H).

^{13}C NMR (101 MHz, $CDCl_3$) δ 150.21, 145.44, 142.49, 140.77, 140.72, 138.23, 137.86, 136.80, 136.55, 136.31, 132.93, 132.88, 131.94, 131.11, 130.45, 129.82, 128.18, 127.21, 125.92, 125.57, 124.40, 122.60, 115.55, 111.30, 90.30, 37.10, 32.60, 32.01, 29.15, 22.79, 21.43, 17.36, 14.33.

HRMS (MALDI-TOF) m/z: $[M]^+$ Calculated for $C_{72}H_{67}B_2Cl_2N_3$ 1065.4915; Found 1065.4910 (-0.5 ppm).

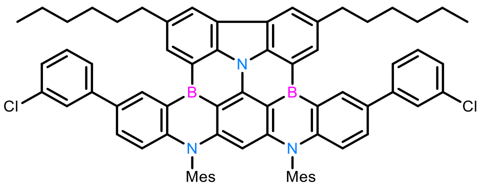

9g

9g (172 mg, 81%) was synthesized from compound **7i** (200 mg) with (3-chlorophenyl)boronic acid (reaction temperature: 90 °C).

^{1}H NMR (400 MHz, $CDCl_3$) δ 9.41 (d, *J* = 2.3 Hz, 2H), 8.94 (d, *J* = 1.4 Hz, 2H), 8.36 (d, *J* = 1.4 Hz, 2H), 7.81 (t, *J* = 1.9 Hz, 2H), 7.76 – 7.65 (m, 4H), 7.47 (t, *J* = 7.8 Hz, 2H), 7.37 (ddd, *J* = 8.0, 2.1, 1.1 Hz, 2H), 6.98 (s, 4H), 6.92 (d, *J* = 9.0 Hz, 2H), 5.36 (s, 1H), 3.13 (t, *J* = 7.7 Hz, 4H), 2.46 (s, 6H), 1.96 (p, *J* = 7.5 Hz, 4H), 1.77 (s, 12H), 1.50 – 1.33 (m, 8H), 0.91 (t, *J* = 7.1 Hz, 6H).

^{13}C NMR (101 MHz, $CDCl_3$) δ 150.19, 145.78, 143.52, 142.47, 140.73, 138.35, 137.93, 136.70, 136.18, 135.00, 134.14, 132.80, 131.62, 130.35, 130.29, 129.86, 129.72, 129.04, 127.54, 127.27, 126.75, 126.16, 125.92, 125.66, 125.15, 124.57, 116.48, 90.42, 37.15, 32.53, 32.01, 29.21, 22.83, 21.49, 21.44, 19.89, 17.27, 14.31, 14.29.

HRMS (MALDI-TOF) m/z: $[M]^{+}$ Calculated for $C_{72}H_{67}B_2Cl_2N_3$ 1065.4915; Found 1065.4944 (2.7 ppm).

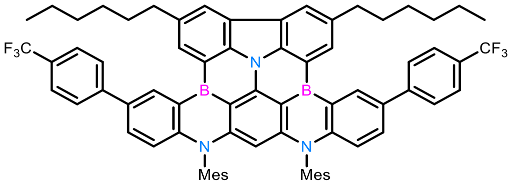

9h

9h (201 mg, 89%) was synthesized from compound **7i** (200 mg) with ((4-(trifluoromethyl)phenyl)boronic acid.

^{1}H NMR (500 MHz, CDCl3) δ 9.45 (d, J = 2.3 Hz, 2H), 8.93 (d, J = 1.4 Hz, 2H), 8.37 (d, J = 1.4 Hz, 2H), 7.92 (d, J = 8.0 Hz, 4H), 7.80 (d, J = 8.1 Hz, 4H), 7.73 (dd, J = 8.8, 2.2 Hz, 2H), 6.99 (s, 4H), 6.95 (d, J = 8.7 Hz, 2H), 5.38 (s, 1H), 3.13 (t, J = 7.7 Hz, 4H), 2.46 (s, 6H), 1.96 (p, J = 7.7 Hz, 4H), 1.77 (s, 12H), 1.55 (s, 13H), 1.48 – 1.35 (m, 9H), 0.92 (t, J = 7.1 Hz, 6H).

^{13}C NMR (101 MHz, $CDCl_3$) δ 150.23, 145.99, 145.19, 142.47, 140.75, 138.43, 137.97, 136.68, 136.14, 134.39, 132.77, 131.59, 130.52, 129.89, 127.26, 126.03, 125.71, 124.63, 123.29, 116.61, 90.52, 37.12, 32.49, 32.07, 29.18, 22.81, 21.45, 17.28, 14.29.

^{19}F NMR (377 MHz, $CDCl_3$) δ -62.19.

HRMS (MALDI-TOF) m/z: $[M]^{+}$ Calculated for $C_{74}H_{67}B_2F_6N_3$ 1133.5442; Found 1133.5504 (5.5 ppm).

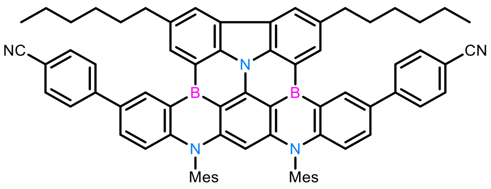

9i

9i (95 mg, 46%) was synthesized from compound **7i** (200 mg) with (4-cyanophenyl)boronic acid.

^{1}H NMR (400 MHz, $CDCl_3$) δ 9.44 (d, *J* = 2.3 Hz, 2H), 8.89 (s, 2H), 8.35 (s, 2H), 7.94 – 7.80 (m, 8H), 7.72 (dd, *J* = 8.9, 2.3 Hz, 2H), 7.02 – 6.95 (m, 6H), 5.40 (s, 1H), 3.10 (t, *J* = 7.6 Hz, 4H), 2.47 (s, 6H), 1.94 (p, *J* = 7.6 Hz, 4H), 1.78 (s, 12H), 1.45 – 1.36 (m, 12H), 0.93 (t, *J* = 6.9 Hz, 6H).

^{13}C NMR (101 MHz, $CDCl_3$) δ 150.22, 146.25, 146.10, 140.70, 138.52, 138.00, 136.60, 136.01, 134.49, 132.94, 132.67, 130.96, 130.37, 129.92, 127.46, 125.72, 124.71, 119.37, 116.74, 110.20, 90.68, 37.11, 32.44, 32.03, 29.14, 22.80, 21.44, 17.25, 14.32.

HRMS (MALDI-TOF) m/z: $[M]^+$ Calculated for $C_{74}H_{67}B_2N_5$ 1047.5599; Found 1047.5621 (2.1 ppm).

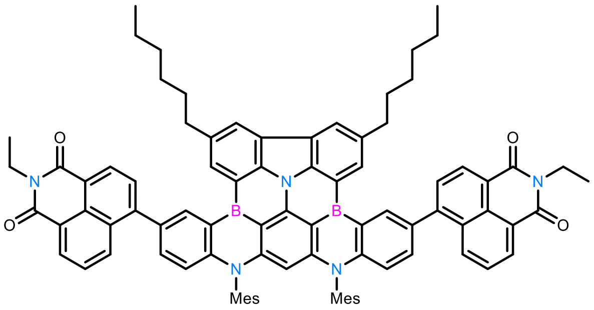

9j

9j (148 g, 57%) was synthesized from compound **7i** (200 mg) with 2-ethyl-6-(4,4,5,5-tetramethyl-1,3,2-dioxaborolan-2-yl)-1*H*-benzo[*de*]isoquinoline-1,3(2*H*)-dione.

^{1}H NMR (400 MHz, $CDCl_3$) δ 9.38 (d, *J* = 2.3 Hz, 2H), 8.79 – 8.64 (m, 8H), 8.32 (d, *J* = 1.3 Hz, 2H), 7.94 (d, *J* = 7.6 Hz, 2H), 7.77 (dd, *J* = 8.5, 7.2 Hz, 2H), 7.68 (dd, *J* = 8.8, 2.2 Hz, 2H), 7.08 – 7.00 (m, 6H), 5.46 (s, 1H), 4.33 (q, *J* = 7.0 Hz, 4H), 2.95 (t, *J* = 7.7 Hz, 4H), 2.48 (s, 6H), 1.84 (s, 12H), 1.84 – 1.74 (m, 6H), 1.40 (t, *J* = 7.0 Hz, 11H), 1.35 – 1.22 (m, 9H), 0.89 – 0.81 (m, 6H).

^{13}C NMR (101 MHz, $CDCl_3$) δ 164.42, 164.26, 150.33, 147.50, 146.08, 142.42, 140.68, 138.58, 138.17, 137.44, 136.66, 136.08, 133.19, 132.65, 131.32, 131.20, 130.73, 130.46, 129.98, 129.24, 128.20, 126.85, 126.43, 125.69, 124.73, 123.25, 122.42, 121.48, 116.37, 111.56, 90.72, 37.05, 35.67, 32.64, 31.97, 29.12, 22.76, 21.46, 17.36, 14.26, 13.57.

HRMS (MALDI-TOF) m/z: $[M]^+$ Calculated for $C_{88}H_{79}B_2N_5O_4$ 1291.6338; Found 1291.6307 (-2.4 ppm).

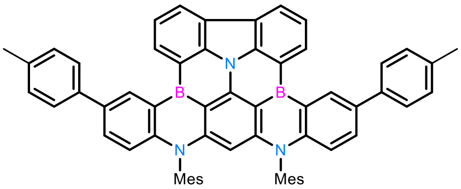

9k

9k (480 mg, 93%) was synthesized from compound **7a** (500 mg) with *p*-tolylboronic acid following similar procedure of **9a**.

^{1}H NMR (400 MHz, $CDCl_3$) δ 9.41 (d, *J* = 2.3 Hz, 2H), 9.18 (d, *J* = 7.4 Hz, 2H), 8.53 (d, *J* = 7.4 Hz, 2H), 7.84 (t, *J* = 7.5 Hz, 2H), 7.74 – 7.69 (m, 6H), 7.36 (d, *J* = 7.9 Hz, 4H), 6.98 (s, 4H), 6.92 (d, *J* = 8.8 Hz, 2H), 5.39 (s, 1H), 2.46 (s, 12H), 1.78 (s, 12H).

^{13}C NMR (101 MHz, $CDCl_3$) δ 150.06, 145.28, 141.90, 138.76, 138.27, 136.75, 136.57, 136.29, 133.83, 133.28, 133.15, 130.69, 129.84, 129.79, 127.14, 125.48, 124.03, 123.39, 116.36, 90.60, 29.86, 21.44, 21.32, 17.30.

HRMS (MALDI-TOF) m/z: $[M]^+$ Calculated for $C_{62}H_{49}B_2N_3$ 857.4126; Found 857.4109 (-2.0 ppm).

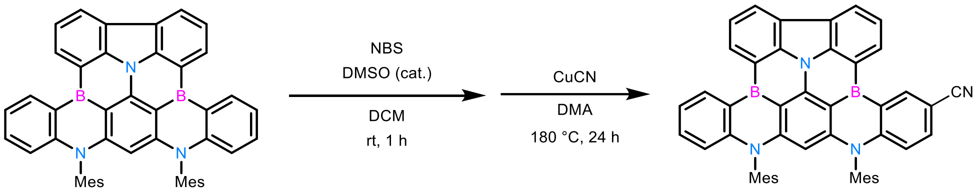

1 **3e**

Compound **1** (100 mg, 0.15 mmol, 1.0 equiv.) was dissolved in DCM (10 mL). DMSO (2 drops) was added. NBS (26 mg, 0.16 mmol, 1.1 equiv.) was added slowly at rt. The reaction mixture stirred at rt for 1 hour. The reaction was quenched by Na_2SO_3 (aq.). The organic phase was separated and dried over Na_2SO_4. After removal of solvent, the residue was sonicated with MeOH and collected by filtration, which was used for the next step.

The intermediate and CuCN (120 mg) were added to a Schlenk bottle. After the addition of DMA (2.0 mL), the bottle was degassed by vacuum for one min. The reaction mixture stirred at 180 °C for 24 hours. After cooling to rt, the mixture was diluted with DCM and $FeCl_3$ (aq.). After work-up, the residue was further purified by column chromatography using hexane/EA (v/v = 20:1 to 4:1) as the eluents to give **3e** (60 mg, 60% yield for two steps) as a yellow solid.

^{1}H NMR (400 MHz, $CDCl_3$) δ 9.43 (d, *J* = 2.1 Hz, 1H), 9.23 (dd, *J* = 7.7, 1.7 Hz, 1H), 9.13 (d, *J* = 7.6 Hz, 1H), 9.00 (d, *J* = 7.6 Hz, 1H), 8.48 (dd, *J* = 7.6, 4.8 Hz, 2H), 7.87 – 7.77 (m, 2H), 7.67 (dd, *J* = 8.9, 2.0 Hz, 1H), 7.53 (ddd, *J* = 8.6, 7.0, 1.7 Hz, 1H), 7.45 – 7.38 (m, 1H), 6.97 (d, *J* = 4.5 Hz, 4H), 6.88 (dd, *J* = 10.6, 8.7 Hz, 2H), 5.42 (s, 1H), 2.45 (s, 6H), 1.73 (d, *J* = 5.7 Hz, 12H).

^{13}C NMR (101 MHz, $CDCl_3$) δ 150.58, 149.85, 148.34, 145.86, 142.76, 141.80, 141.59, 140.85, 138.86, 138.43, 136.61, 136.37, 136.11, 135.70, 135.48, 134.00, 133.26, 132.60, 132.12, 130.08, 129.88, 125.60, 125.40, 124.52, 124.23, 123.65, 123.63, 121.07, 120.50, 116.43, 116.15, 111.44, 103.40, 91.26, 21.42, 17.20, 17.18.

HRMS (MALDI-TOF) m/z: $[M]^+$ Calculated for $C_{49}H_{36}B_2N_4$ 702.3136; Found 702.3220 (11.9 ppm).

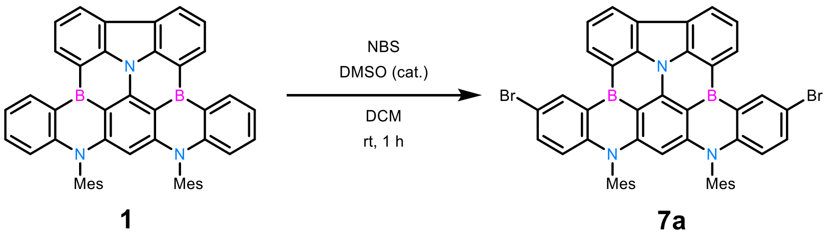

Compound **1** (1.0 g, 1.48 mmol, 1.0 equiv.) was dissolved in DCM (120 mL). DMSO (10 drops) was added. NBS (680 mg, 3.84 mmol, 2.6 equiv.) was added portion-wise at rt. The reaction mixture stirred at rt for 1 hour. The reaction was quenched by Na_2SO_3 (aq.). The organic phase was separated and dried over Na_2SO_4. After removal of solvent, the residue was purified by column chromatography using hexane/DCM (v/v = 4:1) as the eluents to give **7a** (1.05 g, 85%) as a brown solid.

^{1}H NMR (400 MHz, $CDCl_3$) δ 9.26 (d, J = 2.4 Hz, 2H), 9.10 – 9.01 (m, 2H), 8.52 (dd, J = 7.4, 0.9 Hz, 2H), 7.84 (t, J = 7.5 Hz, 2H), 7.55 (dd, J = 9.1, 2.4 Hz, 2H), 6.96 (s, 4H), 6.73 (d, J = 9.1 Hz, 2H), 5.34 (s, 1H), 1.71 (s, 12H).

^{13}C NMR (101 MHz, $CDCl_3$) δ 150.11, 144.70, 142.84, 141.62, 138.55, 137.74, 136.54, 135.88, 134.42, 132.73, 129.95, 125.42, 124.28, 123.56, 117.96, 114.14, 90.53, 21.41, 17.17.

HRMS (MALDI-TOF) m/z: $[M]^+$ Calculated for $C_{48}H_{35}B_2Br_2N_3$ 835.1378; Found 835.1393 (1.8 ppm).

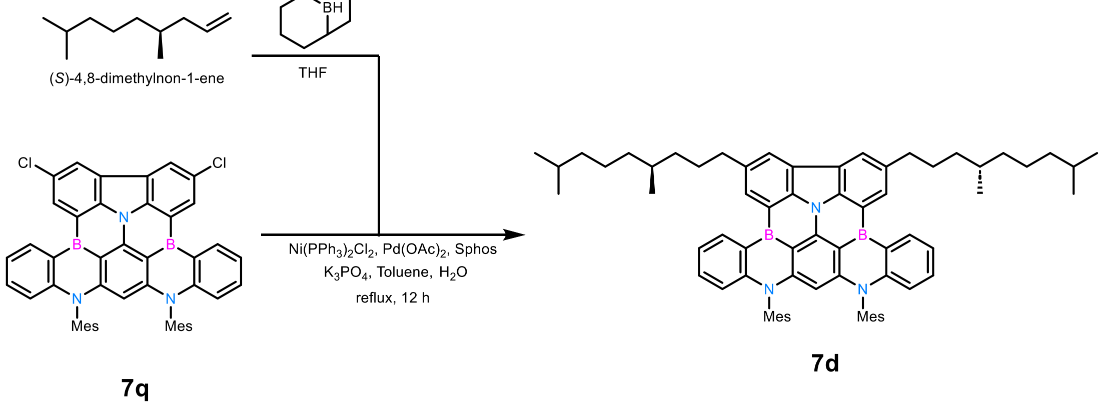

(S)-4,8-dimethylnon-1-ene (250 mg, 1.61 mmol, 4.0 equiv.) was added to a THF solution of 9-borabicyclo[3.3.1]nonane (3.2 mL, 0.5 M) at rt and stirred for 4 h to generate (*S*)-9-(3,7-dimethyloctyl)-9-borabicyclo[3.3.1]nonane (alkyl-BBN) solution. Another Schlenk bottle was charged with **7q** (300 mg, 0.4 mmol, 1.0 equiv.), $Pd(OAc)_2$ (9 mg, 10 mol%), Sphos (24 mg, 15 mol%), $Ni(PPh_3)_2Cl_2$ (26 mg, 10 mol%) and K_3PO_4 (850 mg, 4.0 mmol, 10.0 equiv.). Toluene (25 mL) and water (2 mL) were added, followed by the addition of alkyl-BBN solution. The reaction mixture was heated to reflux for 12 h. After work-up, the residue was purified by column chromatography using hexane/DCM (v/v = 8:1) as the eluents to give **7d** (153 mg, 38%) as a yellow solid.

^{1}H NMR (400 MHz, $CDCl_3$) δ 9.23 (d, J = 7.6 Hz, 2H), 8.91 (s, 2H), 8.33 (s, 2H), 7.53 – 7.46 (m, 2H), 7.39 (t, J = 7.1 Hz, 2H), 6.95 (s, 4H), 6.85 (d, J = 8.4 Hz, 2H), 5.32 (s, 1H), 3.09 (td, J = 8.2, 2.8 Hz, 4H), 2.44 (s, 6H), 1.94 – 1.90 (m, 4H), 1.74 (s, 12H), 1.59 – 1.50 (m, 6H), 1.39 – 1.28 (m, 8H), 1.19 – 1.14 (m, 6H), 0.97 – 0.94 (m, 6H), 0.88 – 0.85 (m, 12H).

^{13}C NMR (101 MHz, $CDCl_3$) δ 150.22, 146.02, 142.53, 140.72, 138.09, 137.80, 136.78, 136.39, 135.66, 133.01, 131.65, 131.24, 129.77, 126.44, 125.55, 124.25, 122.71, 120.50, 115.84, 111.11, 90.04, 39.51, 37.63, 37.46, 37.08, 32.99, 30.55, 28.12, 25.01, 22.88, 22.78, 21.41, 19.95, 17.24.

HRMS (MALDI-TOF) m/z: $[M]^+$ Calculated for $C_{70}H_{81}B_2N_3$ 985.6632; Found 985.6696 (6.5 ppm).

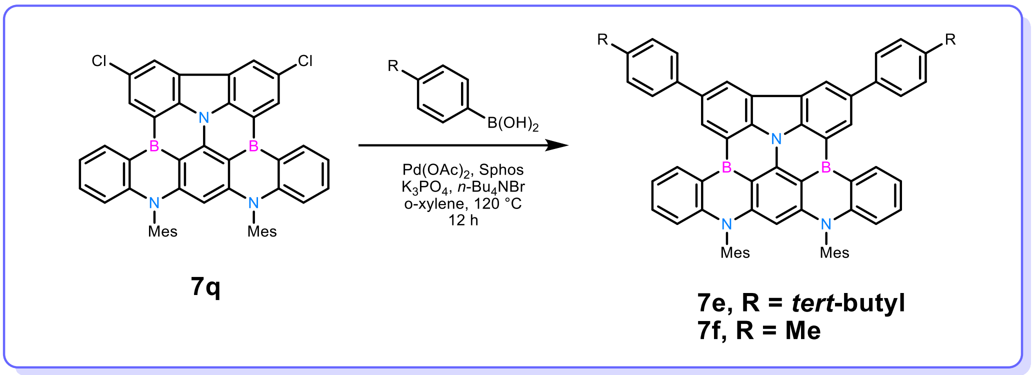

Compound **7q** (1.0 equiv.) was added to a 120 mL Schlenk bottle. Boronic acids (4.0 equiv.), $Pd(OAc)_2$ (10 mol%), Sphos (15 mol%), *n*-Bu_4NBr (20 mol%) and K_3PO_4 (10.0 equiv.) were added. The mixture was dried under vacuum. After back-filled with nitrogen, *o*-xylene (20 mL) was added. The reaction mixture stirred at 120 °C for 12 hours. After cooling to rt, the mixture was passed through a pad of celite, rinsed with DCM. After removal of the solvent, DCM (5 mL) was added to dissolve the residue. MeOH was added to precipitate yellow solids, which were collected by filtration. Conpunds **7e** or **7f** were obtained as yellow powders, respectively.

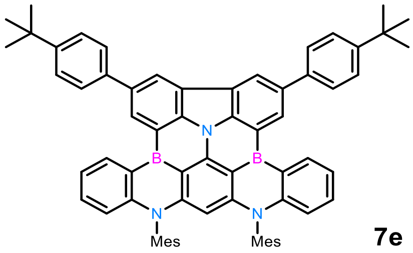

7e

7e (146 mg, 58% yield) was synthesized from **7q** (200 mg, 0.27 mmol).

^{1}H NMR (400 MHz, $CDCl_3$) δ 9.33 (d, J = 1.5 Hz, 2H), 9.25 (dd, J = 7.7, 1.7 Hz, 2H), 8.75 (d, J = 1.5 Hz, 2H), 7.97 – 7.87 (m, 4H), 7.70 – 7.62 (m, 4H), 7.50 (ddd, J = 8.6, 6.9, 1.7 Hz, 2H), 7.40 – 7.34 (m, 2H), 6.96 (s, 4H), 6.89 – 6.83 (m, 2H), 5.37 (s, 1H), 2.45 (s, 6H), 1.75 (s, 12H), 1.47 (s, 18H).

^{13}C NMR (101 MHz, $CDCl_3$) δ 150.20, 150.08, 146.09, 142.78, 141.67, 140.19, 138.19, 136.90, 136.75, 136.31, 135.68, 132.54, 131.91, 129.81, 127.89, 126.09, 125.94, 123.33, 120.71, 115.96, 90.50, 34.78, 31.66, 29.85, 21.42, 17.26.

HRMS (MALDI-TOF) m/z: $[M]^+$ Calculated for $C_{68}H_{61}B_2N_3$ 941.5066; Found 941.5151 (9.0 ppm).

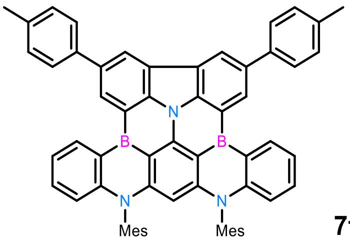

7f

7f (734 mg, 80% yield) was synthesized from **7q** (800 mg, 1.07 mmol).

^{1}H NMR (400 MHz, $CDCl_3$) δ 9.31 (d, *J* = 1.6 Hz, 2H), 9.25 (dd, *J* = 7.7, 1.7 Hz, 2H), 8.75 (d, *J* = 1.4 Hz, 2H), 7.92 – 7.84 (m, 4H), 7.54 – 7.35 (m, 9H), 6.96 (s, 4H), 6.86 (dd, *J* = 8.6, 1.1 Hz, 2H), 5.37 (s, 1H), 2.52 (s, 6H), 2.45 (s, 6H), 1.75 (s, 12H).

^{13}C NMR (101 MHz, $CDCl_3$) δ 150.18, 146.08, 142.76, 141.64, 140.20, 138.20, 137.00, 136.82, 136.74, 136.29, 135.65, 132.48, 131.91, 129.84, 129.81, 128.09, 125.93, 123.30, 120.70, 115.96, 90.49, 21.43, 21.35, 17.26.

HRMS (MALDI-TOF) m/z: $[M]^+$ Calculated for $C_{62}H_{49}B_2N_3$ 857.4126; Found 857.4205 (9.2 ppm).

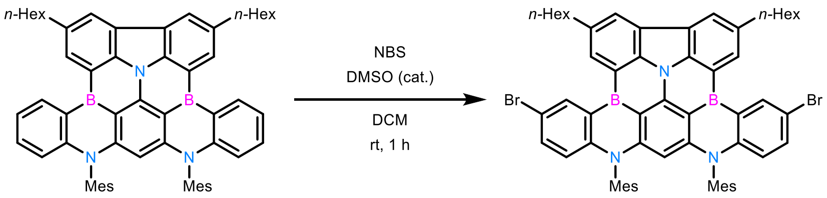

7c **7i**

Compound **7c** (1.80 g, 2.13 mmol, 1.0 equiv.) was dissolved in DCM (300 mL). DMSO (10 drops) was added. NBS (980 mg, 5.53 mmol, 2.6 equiv.) was added portion-wise at rt. The reaction mixture stirred at rt for 1 hour. The reaction was quenched by Na_2SO_3 (aq.). The organic phase was separated and dried over Na_2SO_4. After removal of solvent, the residue was purified by column chromatography using hexane/DCM (v/v = 6:1) as the eluents to give **7i** (1.64 g, 77%) as a brown solid.

^{1}H NMR (400 MHz, $CDCl_3$) δ 9.25 (d, *J* = 2.5 Hz, 1H), 8.80 (d, *J* = 1.4 Hz, 1H), 8.34 (d, *J* = 1.3 Hz, 1H), 7.54 (dd, *J* = 9.1, 2.4 Hz, 1H), 6.95 (s, 2H), 6.72 (d, *J* = 9.1 Hz, 1H), 3.11 (t, *J* = 7.7 Hz, 2H), 2.43 (s, 3H), 1.93 (dq, *J* = 12.9, 7.5 Hz, 2H), 1.71 (s, 6H), 1.55 – 1.35 (m, 6H), 0.94 (t, *J* = 7.0 Hz, 3H).

^{13}C NMR (101 MHz, $CDCl_3$) δ 150.18, 144.68, 142.37, 140.57, 138.48, 138.25, 137.75, 136.58, 135.93, 134.25, 132.58, 129.91, 125.67, 124.69, 117.91, 114.01, 90.15, 37.17, 32.82, 32.04, 29.27, 22.86, 21.41, 17.17, 14.35.

HRMS (MALDI-TOF) m/z: $[M]^+$ Calculated for $C_{60}H_{59}B_2Br_2N_3$ 1003.3261; Found 1003.3224 (-3.7 ppm).

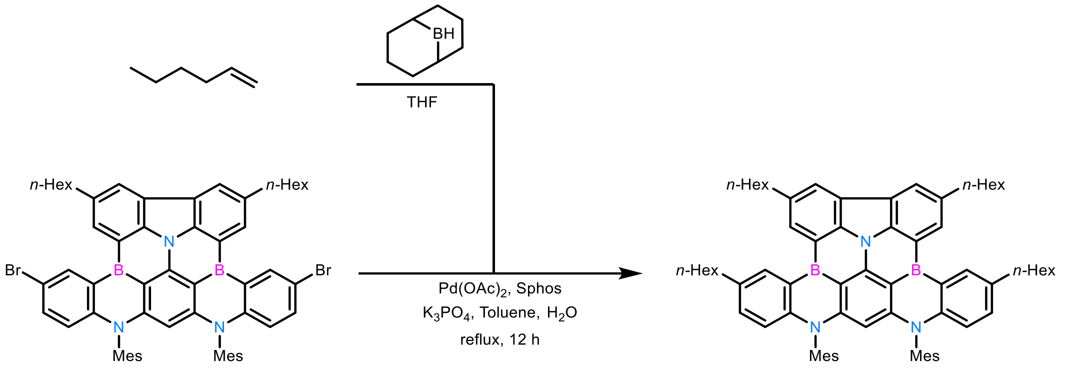

7i **7j**

Hex-1-ene (130 mg, 1.59 mmol, 4.0 equiv.) was added to a THF solution of 9-borabicyclo[3.3.1]nonane (3.2 mL, 0.5 M) at rt and stirred for 4 h to generate C_6H_{13}-BBN solution. Another Schlenk bottle was charged with **7i** (400 mg, 0.4 mmol, 1.0 equiv.), $Pd(OAc)_2$ (9 mg, 10 mol%), Sphos (24 mg, 15 mol%) and K_3PO_4 (850 mg, 4.0 mmol, 10.0 equiv.). Toluene (25 mL) and water (2 mL) were added, followed by the addition of C_6H_{13}-BBN solution. The reaction mixture was heated to reflux for 12 h. After work-up, the residue was purified by column chromatography using hexane/DCM (v/v = 20:1 to 15:1, 6:1) as the eluents to give **7j** (125 mg, 31%) as a yellow solid.

^{1}H NMR (400 MHz, $CDCl_3$) δ 8.98 (d, J = 2.1 Hz, 2H), 8.91 (d, J = 1.4 Hz, 2H), 8.33 (s, 2H), 7.29 (dd, J = 8.7, 2.1 Hz, 2H), 6.93 (s, 4H), 6.75 (d, J = 8.7 Hz, 2H), 5.24 (s, 1H), 3.13 (t, J = 7.8 Hz, 4H), 2.86 (t, J = 7.7 Hz, 4H), 2.43 (s, 6H), 2.01 – 1.93 (m, 4H), 1.91 – 1.85 (m, 4H), 1.72 (s, 12H), 1.53 – 1.33 (m, 24H), 0.95 – 0.90 (m, 12H).

^{13}C NMR (101 MHz, $CDCl_3$) δ 150.00, 144.26, 142.65, 140.75, 139.86, 137.95, 137.53, 136.84, 136.56, 134.78, 134.40, 132.97, 132.14, 131.57, 129.69, 126.33, 125.50, 124.13, 122.86, 115.73, 111.01, 89.63, 37.22, 35.68, 32.69, 32.07, 32.02, 31.68, 29.32, 29.24, 22.86, 22.83, 21.41, 17.28, 14.34, 14.31.

HRMS (MALDI-TOF) m/z: $[M]^+$ Calculated for $C_{72}H_{85}B_2N_3$ 1013.6945; Found 1013.6995 (4.9 ppm).

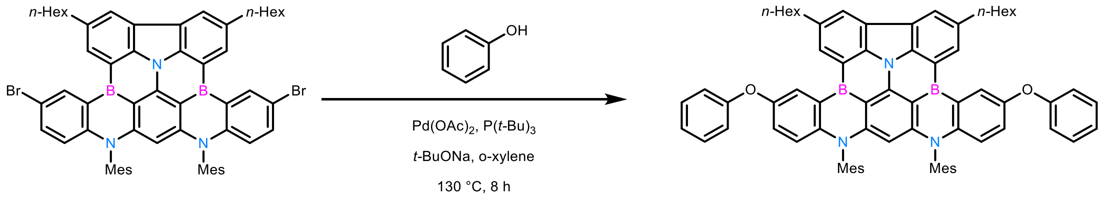

7i **7k**

Compound **7i** (200 mg, 0.2 mmol, 1.0 equiv.) was added to a 120 mL Schlenk bottle. $Pd(OAc)_2$ (13.4mg, 30 mol%), tri-*tert*-butylphosphine (240 mg, 10 wt%, 60 mol%), phenol (56 mg, 0.6 mmol, 3.0 equiv.) and *o*-xylene (20 mL) were added. The mixture was vacuumed and stirred at rt for 5 min. After back-filled with nitrogen, *t*-BuONa (120 mg, 1.2 mmol, 6.0 equiv.) was added. The reaction mixture stirred at 130 °C for 8 hours. After cooling to rt, the mixture was passed through a pad of celite, rinsed with DCM. After removal of the solvent, the residue was purified by column chromatography using hexane/DCM (v/v = 8:1) as the eluents to give **7k** (65 mg, 32%) as a yellow solid.

^{1}H NMR (400 MHz, $CDCl_3$) δ 8.75 (d, J = 2.9 Hz, 2H), 8.59 (s, 2H), 8.29 (s, 2H), 7.43 (t, J =

7.9 Hz, 4H), 7.25 – 7.16 (m, 8H), 6.95 (s, 4H), 6.84 (d, *J* = 9.2 Hz, 2H), 5.27 (s, 1H), 2.99 (t, *J* = 7.8 Hz, 4H), 2.43 (s, 6H), 1.83 (q, *J* = 7.7 Hz, 4H), 1.75 (s, 12H), 1.51 – 1.36 (m, 12H), 0.93 (t, *J* = 6.8 Hz, 6H).

^{13}C NMR (101 MHz, $CDCl_3$) δ 158.08, 150.78, 150.05, 142.60, 142.23, 140.58, 138.21, 137.97, 136.75, 136.39, 132.61, 129.94, 129.81, 127.31, 125.50, 124.35, 123.72, 123.27, 123.18, 122.50, 119.08, 118.71, 117.36, 110.91, 89.52, 37.22, 32.99, 32.04, 29.33, 22.88, 21.39, 17.27, 14.34.

HRMS (MALDI-TOF) m/z: $[M]^+$ Calculated for $C_{72}H_{69}B_2N_3O_2$ 1029.5570; Found 1029.5659 (8.6 ppm).

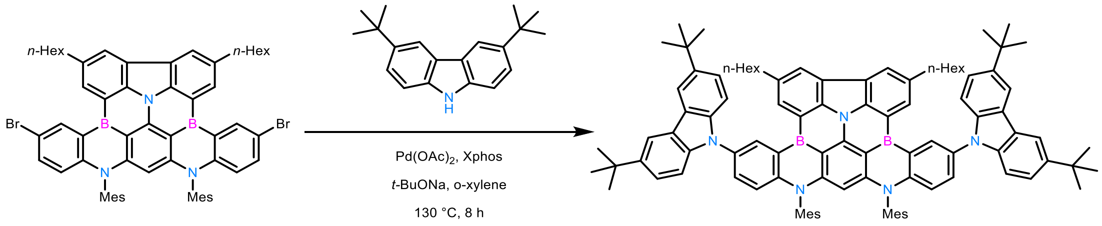

7i **7l**

Compound **7i** (200 mg, 0.2 mmol, 1.0 equiv.) was added to a 120 mL Schlenk bottle. $Pd(OAc)_2$ (9.0 mg, 20 mol%), Xphos (38 mg, 40 mol%), 3,6-di-*tert*-butyl-9H-carbazole (167 mg, 0.6 mmol, 3.0 equiv.) and *o*-xylene (20 mL) were added. The mixture was vacuumed and stirred at rt for 5 min. After back-filled with nitrogen, *t*-BuONa (120 mg, 1.2 mmol, 6.0 equiv.) was added. The reaction mixture stirred at 130 °C for 8 hours. After cooling to rt, the mixture was passed through a pad of celite, rinsed with DCM. After removal of the solvent, the residue was purified by column chromatography using hexane/DCM (v/v = 5:1) as the eluents to give **7l** (235 mg, 84%) as a yellow solid.

^{1}H NMR (400 MHz, $CDCl_3$) δ 9.38 (d, *J* = 2.5 Hz, 2H), 8.71 (s, 2H), 8.31 (s, 2H), 8.22 (d, *J* = 1.8 Hz, 4H), 7.64 (dd, *J* = 9.1, 2.4 Hz, 2H), 7.57 (d, *J* = 8.6 Hz, 4H), 7.51 (dd, *J* = 8.6, 1.9 Hz, 4H), 7.03 (d, *J* = 11.4 Hz, 6H), 5.42 (s, 1H), 2.94 (t, *J* = 7.8 Hz, 4H), 2.48 (s, 6H), 1.85 (s, 12H), 1.80 – 1.73 (m, 4H), 1.50 (s, 36H), 1.44 – 1.28 (m, 13H), 0.88 – 0.81 (m, 6H).

^{13}C NMR (101 MHz, $CDCl_3$) δ 150.35, 144.69, 142.81, 142.56, 140.67, 139.99, 138.45, 138.29, 136.76, 136.28, 133.19, 132.67, 130.81, 129.97, 127.26, 125.66, 124.67, 123.74, 123.39, 122.49, 117.20, 116.36, 111.29, 109.56, 90.27, 37.10, 34.91, 32.88, 32.22, 31.98, 29.88, 29.21, 22.79, 21.45, 17.41, 14.24.

HRMS (MALDI-TOF) m/z: $[M]^+$ Calculated for $C_{100}H_{107}B_2N_5$ 1399.8707; Found 1399.8720 (0.9 ppm).

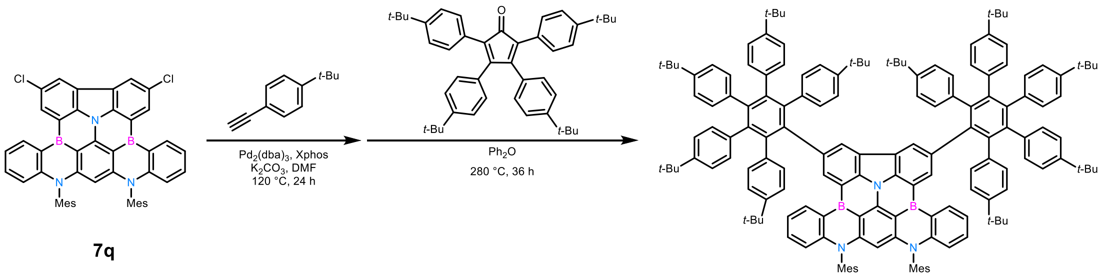

7q **11**

Compound **7q** (200 mg, 0.27 mmol, 1.0 equiv.) was added to a 50 mL Schlenk bottle. $Pd_2(dba)_3$

(24 mg, 10 mol%), Xphos (26 mg, 20 mol%), and K_3CO_3 (370 mg, 2.68 mmol, 10.0 equiv.) were added. The mixture was dried under vacuum. After backfilled with nitrogen, DMF (5.0 mL) was added. 1-(*Tert*-butyl)-4-ethynylbenzene (260 mg, 1.61 mmol, 6.0 equiv.) was finally injected via syringe. The reaction mixture stirred at 120 °C for 24 hours. After cooling to rt, the mixture was passed through a pad of celite, rinsed with DCM. After removal of the solvent, the residue was sonicated in MeOH, which was collected by filtration. The intermediate was used directly for the next step.

The intermediate (ca. 236 mg) was transferred to a 120 mL thick-wall vial, and 2,3,4,5-tetrakis(4-(*tert*-butyl)phenyl)cyclopenta-2,4-dien-1-one (850 mg, 1.39 mmol, 6.0 equiv.) was added. After the addition of Ph_2O (3.0 mL), the vial was connected to the Schlenk line and vacuumed for 1 min. Stirred in a 280 °C sand bath for 36 hours. After cooling to rt, the mixture was poured into brine, extracted with DCM, dried over Na_2SO_4. After removal of solvent, the residue was purified by column chromatography using hexane/DCM (v/v = 4:1) as the eluents to give **11** (243 mg, 42% yield for two steps).

^{1}H NMR (400 MHz, $CDCl_3$) δ 8.56 (dd, *J* = 7.9, 1.7 Hz, 2H), 8.43 – 8.34 (m, 2H), 7.67 (d, *J* = 1.3 Hz, 2H), 7.36 (ddd, *J* = 8.6, 6.9, 1.6 Hz, 2H), 7.18 (t, *J* = 7.2 Hz, 2H), 6.97 – 6.63 (m, 48H), 5.14 (s, 1H), 2.40 (s, 6H), 1.61 (s, 12H), 1.15 – 1.14 (m, 60H), 0.84 (s, 30H).

^{13}C NMR (101 MHz, $CDCl_3$) δ 149.96, 147.56, 147.47, 145.70, 142.15, 141.18, 141.05, 140.94, 139.53, 138.24, 138.19, 138.12, 137.91, 137.17, 136.70, 136.51, 136.39, 135.43, 131.86, 131.44, 131.37, 131.28, 131.17, 129.66, 127.32, 124.05, 123.94, 123.43, 123.33, 123.23, 120.61, 119.98, 115.16, 110.56, 89.62, 34.23, 34.03, 31.37, 31.03, 21.39, 16.98.

HRMS (MALDI-TOF) m/z: $[M]^+$ Calculated for $C_{160}H_{165}B_2N_3$ 2151.3241; Found 2151.3349 (5.0 ppm).

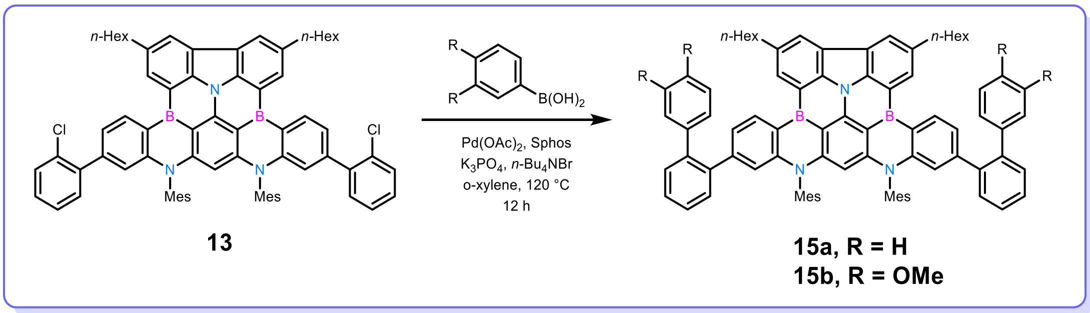

Compound **13** (1.0 equiv.) was added to a 120 mL Schlenk bottle. Boronic acids (4.0 equiv.), $Pd(OAc)_2$ (10 mol%), Sphos (15 mol%), *n*-Bu_4NBr (20 %) and K_3PO_4 (10.0 equiv.) were added. The mixture was dried under vacuum. After back-filled with nitrogen, *o*-xylene (20 mL) was added. The reaction mixture stirred at 120 °C for 12 hours. After cooling to rt, the mixture was passed through a pad of celite, rinsed with DCM. After removal of the solvent, DCM (5 mL) was added to dissolve the residue. MeOH was added to precipitate yellow solids, which were collected by filtration. Conpunds **15a** or **15b** were obtained.

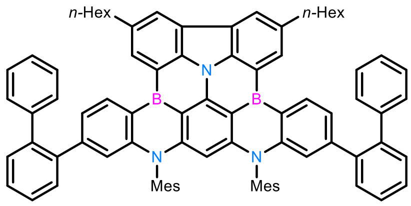

15a

15a (482 mg, 89% yield) was synthesized from **13** (500 mg) with phenylboronic acid.

^{1}H NMR (400 MHz, $CDCl_3$) δ 9.12 (d, *J* = 7.8 Hz, 2H), 8.87 (s, 2H), 8.32 (s, 2H), 7.52 – 7.29 (m, 10H), 7.24 – 6.97 (m, 10H), 6.72 (s, 4H), 6.52 (s, 2H), 5.09 (s, 1H), 3.10 (t, *J* = 7.8 Hz, 4H), 2.36 (s, 6H), 1.93 – 1.89 (m, 4H), 1.46 – 1.29 (m, 24H), 0.93 (t, *J* = 6.8 Hz, 6H).

^{13}C NMR (101 MHz, $CDCl_3$) δ 150.13, 145.58, 144.87, 142.37, 141.54, 141.16, 140.86, 140.68, 137.73, 137.58, 136.32, 135.94, 135.38, 132.98, 131.06, 130.93, 129.71, 129.64, 128.11, 127.56, 127.40, 126.58, 125.48, 124.74, 124.19, 122.73, 122.27, 117.49, 111.12, 90.12, 37.30, 33.02, 32.08, 29.86, 29.34, 22.88, 21.35, 17.17, 14.35.

HRMS (MALDI-TOF) m/z: $[M]^+$ Calculated for $C_{84}H_{77}B_2N_3$ 1149.6322; Found 1149.6291 (2.3 ppm).

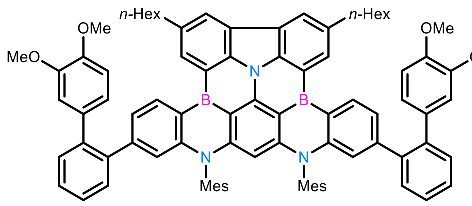

15b

15b (298 mg, 83% yield) was synthesized from **13** (300 mg) with (3,4-dimethoxyphenyl)boronic acid.

^{1}H NMR (400 MHz, $CDCl_3$) δ 9.15 (d, *J* = 7.9 Hz, 2H), 8.88 (s, 2H), 8.33 (s, 2H), 7.44 – 7.34 (m, 10H), 6.73 (s, 4H), 6.67 – 6.48 (m, 8H), 5.06 (s, 1H), 3.79 (s, 6H), 3.57 (s, 6H), 3.12 (t, *J* = 7.8 Hz, 4H), 2.35 (s, 6H), 1.98 – 1.88 (m, 4H), 1.46 – 1.24 (m, 36H), 0.93 (t, *J* = 6.9 Hz, 6H).

^{13}C NMR (101 MHz, $CDCl_3$) δ 150.13, 148.34, 147.66, 145.60, 145.06, 141.08, 140.69, 140.58, 137.78, 137.55, 136.36, 135.93, 135.50, 134.06, 133.00, 131.11, 130.66, 129.54, 127.61, 127.25, 125.51, 124.23, 122.25, 122.03, 117.32, 112.96, 110.78, 90.07, 55.80, 55.58, 37.32, 33.02, 32.08, 31.66, 30.27, 29.86, 29.38, 22.89, 21.40, 16.95, 14.35.

HRMS (MALDI-TOF) m/z: $[M]^+$ Calculated for $C_{88}H_{85}B_2N_3O_4$ 1269.6746; Found 1269.6672 (-5.8 ppm).

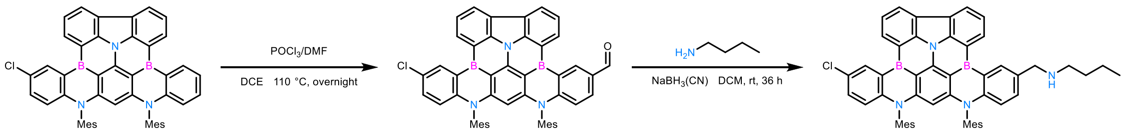

3c **7r** **20**

Compound **3c** (1.0 g, 1.4 mmol, 1.0 equiv.) was dissolved in DMF/DCE (4 mL/16 mL) at rt. $POCl_3$ (1.6 mL) was slowly added. The reaction mixture was heated to reflux overnight. After cooling to rt, the residue was dissolved in DCM and carefully poured into K_2CO_3 (aq.) with vigorous stirring. Extracted with DCM and dried over Na_2SO_4. After removal of solvent, the residue was purified by column chromatography using hexane/EA (v/v = 20:1 to 6:1) as the eluents to give **7r** (624 mg, 60%) as a yellow solid.

Compound **7r** (140 mg, 0.19 mmol, 1.0 equiv.) was dissolved in DCM (10 mL). Butan-1-amine (30 mg, 0.38 mmol, 2.0 eq) was injected and stirred at rt for 3 hours. $NaBH_3(CN)$ (100 mg, 1.5 mmol, 8.0 equiv.) was finally added. The reaction mixture stirred at rt for 36 hours. After removal of DCM, the residue was directly purified by column chromatography using hexane/EA (v/v = 9:1 to 1:1) as the eluents to give **20** (83 mg, 55%) as a brown solid.

^{1}H NMR (400 MHz, $CDCl_3$) δ 9.16 (d, *J* = 7.6 Hz, 1H), 9.12 – 9.04 (m, 3H), 8.52 (dd, *J* = 7.3,

3.1 Hz, 2H), 7.84 (t, J = 7.5 Hz, 2H), 7.48 (dd, J = 8.8, 2.0 Hz, 1H), 7.40 (dd, J = 9.1, 2.5 Hz, 1H), 6.94 (d, J = 4.4 Hz, 4H), 6.82 (d, J = 8.7 Hz, 1H), 6.77 (d, J = 9.1 Hz, 1H), 5.32 (s, 1H), 4.03 (s, 2H), 3.24 (td, J = 7.2, 5.7 Hz, 2H), 2.79 (t, J = 7.3 Hz, 2H), 2.43 (s, 6H), 1.71 (d, J = 4.3 Hz, 12H), 1.52 – 1.44 (m, 2H), 0.95 – 0.92 (m, 3H).

^{13}C NMR (151 MHz, DMSO-*d6*) δ 149.63, 146.61, 144.56, 143.74, 142.06, 141.08, 140.69, 137.44, 137.30, 136.13, 135.84, 135.79, 133.40, 133.14, 133.11, 131.53, 130.54, 130.17, 129.57, 129.49, 127.39, 124.06, 123.85, 122.86, 122.46, 122.38, 121.49, 119.90, 114.67, 114.19, 90.00, 63.20, 41.05, 34.53, 20.78, 20.75, 19.51, 17.09, 16.61, 13.79.

HRMS (MALDI-TOF) m/z: [M]$^+$ Calculated for $C_{53}H_{47}B_2ClN_4$ 796.3687; Found 796.3902 (27.0 ppm).

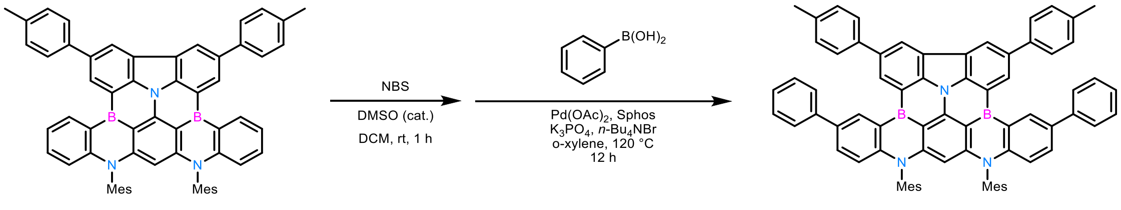

7f **24a**

Compound **7f** (300 mg, 0.35 mmol, 1.0 equiv.) was dissolved in DCM (50 mL). DMSO (5 drops) was added. NBS (161 mg, 0.91 mmol, 2.6 equiv.) was added portion-wise at rt. The reaction mixture stirred at rt for 1 hour. The reaction was quenched by Na_2SO_3 (aq.). The organic phase was separated and dried over Na_2SO_4. After removal of solvent, the residue was purified by column chromatography using hexane/DCM (v/v = 3:1) as the eluents to give the intermediate (325 mg, 91%) as a brown solid, which was used for the next step. **^{1}H NMR** (400 MHz, $CDCl_3$) δ 9.30 (s, 2H), 9.23 (s, 2H), 8.77 (s, 2H), 7.88 (d, J = 7.7 Hz, 4H), 7.60 – 7.53 (m, 2H), 7.46 (d, J = 7.6 Hz, 4H), 6.96 (s, 4H), 6.75 (d, J = 9.1 Hz, 2H), 5.35 (s, 1H), 2.52 (s, 6H), 2.44 (s, 6H), 1.73 (s, 12H). **^{13}C NMR** (101 MHz, $CDCl_3$) δ 150.15, 144.73, 142.64, 141.49, 139.90, 138.58, 137.75, 137.39, 137.04, 136.54, 135.84, 134.52, 131.99, 129.97, 129.95, 128.09, 126.06, 123.78, 118.02, 114.19, 90.60, 21.42, 21.36, 17.19.

The intermediate (230 mg, 0.22 mmol, 1.0 equiv.) was added to a 120 mL Schlenk bottle. phenylboronic acid (90 mg, 0.68 mmol, 3.0 equiv.), $Pd(OAc)_2$ (10 mg, 20 mol%), Sphos (28 mg, 30 mol%), *n*-Bu_4NBr (36 mg, 50 mol%) and K_3PO_4 (480 mg, 2.26 mmol, 10.0 equiv.) were added. The mixture was dried under vacuum. After back-filled with nitrogen, *o*-xylene (20 mL) was added. The reaction mixture stirred at 120 °C for 12 hours. After cooling to rt, the mixture was passed through a pad of celite, rinsed with DCM. After removal of the solvent, the residue was purified by column chromatography using hexane/DCM (v/v = 4:1) as the eluents to give **24a** (190 mg, 83%) as a yellow solid.

^{1}H NMR (400 MHz, $CDCl_3$) δ 9.54 (d, J = 2.1 Hz, 2H), 9.43 (d, J = 1.6 Hz, 2H), 8.79 (d, J = 1.4 Hz, 2H), 7.90 (d, J = 7.8 Hz, 4H), 7.86 – 7.80 (m, 4H), 7.77 (dt, J = 8.9, 1.7 Hz, 2H), 7.54 (t, J = 7.6 Hz, 4H), 7.46 – 7.37 (m, 6H), 7.00 (s, 4H), 6.96 (dd, J = 8.9, 1.3 Hz, 2H), 5.42 (s, 1H), 2.52 (s, 6H), 2.47 (s, 6H), 1.81 (s, 12H).

^{13}C NMR (101 MHz, $CDCl_3$) δ 150.13, 145.51, 142.79, 141.70, 141.45, 139.98, 138.33, 136.89, 136.84, 136.73, 136.24, 133.99, 133.19, 132.30, 130.77, 129.94, 129.86, 129.13, 127.85, 127.07, 126.84, 126.05, 123.29, 116.48, 90.72, 21.44, 21.33, 17.31.

HRMS (MALDI-TOF) m/z: [M]$^+$ Calculated for $C_{74}H_{57}B_2N_3$ 1009.4755; Found 1009.4791

(3.6 ppm).

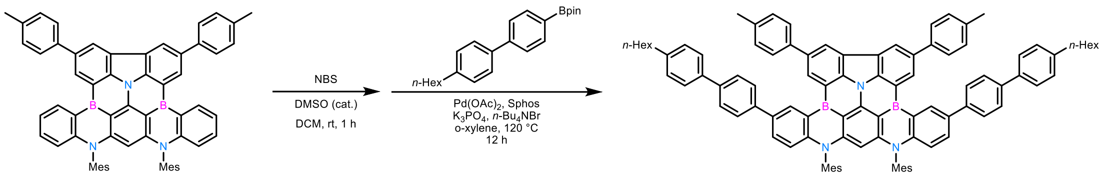

Similar to the synthesis of **28a**, the brominated intermediate (230 mg, 0.22 mmol, 1.0 equiv.) was added to a 120 mL Schlenk bottle. 2-(4'-Hexyl-[1,1'-biphenyl]-4-yl)-4,4,5,5-tetramethyl-1,3,2-dioxaborolane (250 mg, 0.68 mmol, 3.0 equiv.), $Pd(OAc)_2$ (10 mg, 20 mol%), Sphos (28 mg, 30 mol%), *n*-Bu_4NBr (36 mg, 50 mol%) and K_3PO_4 (480 mg, 2.26 mmol, 10.0 equiv.) were added. The mixture was dried under vacuum. After back-filled with nitrogen, *o*-xylene (20 mL) was added. The reaction mixture stirred at 120 °C for 12 hours. After cooling to rt, the mixture was passed through a pad of celite, rinsed with DCM. After removal of the solvent, the residue was purified by column chromatography using hexane/DCM (v/v = 3:1) as the eluents to give **24b** (172 mg, 57%) as a yellow solid.

^{1}H NMR (400 MHz, $CDCl_3$) δ 9.56 (d, *J* = 2.4 Hz, 2H), 9.44 (d, *J* = 2.4 Hz, 2H), 8.80 (d, *J* = 1.4 Hz, 2H), 7.90 (dd, *J* = 8.1, 6.1 Hz, 8H), 7.80 (dd, *J* = 8.8, 2.2 Hz, 2H), 7.75 (d, *J* = 8.2 Hz, 4H), 7.63 (d, *J* = 7.9 Hz, 4H), 7.44 (d, *J* = 7.9 Hz, 4H), 7.31 (d, *J* = 7.8 Hz, 4H), 7.00 (s, 4H), 6.96 (d, *J* = 8.9 Hz, 2H), 6.59 (d, *J* = 8.4 Hz, 2H), 5.42 (s, 1H), 2.68 (t, *J* = 7.8 Hz, 4H), 2.51 (s, 6H), 2.47 (s, 6H), 1.81 (s, 12H), 1.69 (d, *J* = 8.2 Hz, 4H), 1.42 – 1.32 (m, 12H), 0.92 (t, *J* = 4.8 Hz, 6H).

^{13}C NMR (101 MHz, $CDCl_3$) δ 158.13, 150.11, 145.53, 142.30, 141.70, 140.07, 140.00, 139.65, 138.38, 136.96, 136.87, 136.75, 136.25, 133.81, 132.76, 132.30, 130.70, 129.98, 129.88, 129.04, 127.86, 127.72, 127.34, 127.07, 126.06, 123.35, 117.33, 116.54, 111.50, 103.65, 90.75, 55.75, 35.83, 31.93, 31.68, 29.25, 27.02, 26.89, 22.80, 21.46, 21.35, 17.34, 14.29, 0.15.

HRMS (MALDI-TOF) m/z: $[M]^+$ Calculated for $C_{98}H_{89}B_2N_3$ 1329.7264; Found 1329.7247 (-1.3 ppm).

10.2 Synthesis of precursors for the borylated substrates

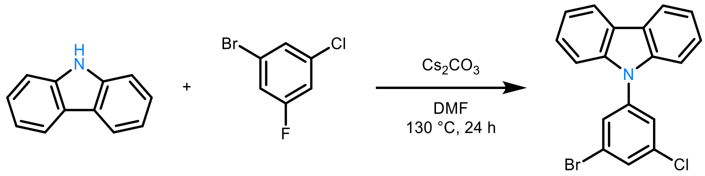

S1

Carbazole (15.0 g, 89.7 mmol, 1.0 equiv.) was dissolved in DMF (60 mL). Cs_2CO_3 (35.0 g, 107 mmol, 1.2 equiv.) and 1-bromo-3-chloro-5-fluorobenzene (28.2 g, 134 mmol, 1.5 equiv.) were added at rt. The reaction mixture stirred at 135 °C for 24 hour. After cooling to rt, the reaction mixture poured into NH_4Cl (aq.) with rigorous stirring. The precipitate was collected by filtration and redissolved in DCM (30 mL). MeOH (400 mL) was finally added to form a suspension, which was further filtered and dried under vacuum to obtain **S1** (27.4 g, 85%) as off-white fine powder.

^{1}H NMR (400 MHz, $CDCl_3$) δ 8.17 – 8.11 (m, 2H), 7.67 (t, *J* = 1.8 Hz, 1H), 7.63 (t, *J* = 1.8 Hz, 1H), 7.56 (t, *J* = 1.8 Hz, 1H), 7.48 – 7.40 (m, 4H), 7.33 (ddd, *J* = 8.0, 6.1, 2.0 Hz, 2H).

^{13}**C NMR** (101 MHz, $CDCl_3$) δ 140.32, 140.05, 136.37, 130.41, 128.46, 126.46, 126.09, 123.84, 123.66, 120.88, 120.63, 109.66.

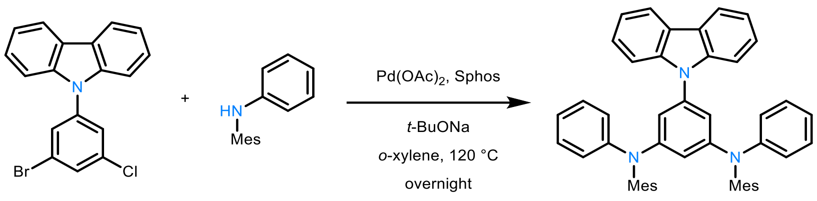

S1 **S2**

Compound **S1** (12.0 g, 22.6 mmol, 1.0 equiv.), $Pd(OAc)_2$ (350 mg, 5 mol%), Sphos (1.0 g, 8 mol%) and 2,4,6-trimethyl-*N*-phenylaniline (14.9 g, 70.6 mmol, 2.1 equiv.) were added to a 1 L flask. After vacuumed and backfilled with nitrogen, *o*-xylene (600 mL) was added. The mixture stirred at rt for 20 min. Under a nitrogen flow, *t*-BuONa (9.7 g, 100 mmol, 3.0 equiv.) was finally added. The reaction mixture stirred at 120 °C overnight. After cooling to rt, the mixture was passed through a pad of celite, rinsed with DCM. After removal of the solvent, the residue was dissolved in DCM (20 mL). MeOH (200 mL) was added to form a suspension, which was collected by filtration and dried under vacuum. Compound **S2** (19.1 g, 86%) was obtained as light gray powder.

^{1}**H NMR** (400 MHz, $CDCl_3$) δ 8.07 (dt, *J* = 7.8, 1.0 Hz, 2H), 7.39 – 7.29 (m, 4H), 7.25 – 7.16 (m, 6H), 7.09 – 7.03 (m, 4H), 6.99 (t, *J* = 2.1 Hz, 1H), 6.92 – 6.84 (m, 6H), 6.48 (d, *J* = 2.1 Hz, 2H), 2.28 (s, 6H), 2.06 (s, 12H).

^{13}**C NMR** (101 MHz, $CDCl_3$) δ 148.04, 145.58, 140.48, 139.83, 139.15, 137.37, 137.02, 130.08, 129.15, 125.81, 123.28, 121.14, 120.23, 119.75, 119.71, 110.17, 110.14, 109.25, 21.12, 18.54.

HRMS (MALDI-TOF) m/z: $[M]^+$ Calculated for $C_{48}H_{43}N_3$ 661.3451; Found 661.3515 (9.7 ppm).

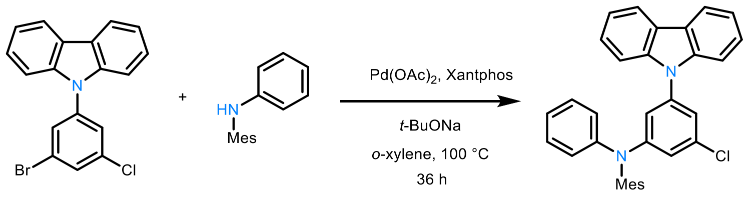

S1 **S3**

Compound **S1** (20.0 g, 56.1 mmol, 1.0 equiv.), $Pd(OAc)_2$ (380 mg, 3 mol%), Xantphos (1.3 g, 4 mol%) and 2,4,6-trimethyl-*N*-phenylaniline (12.5 g, 58.9 mmol, 1.05 equiv.) were added to a 1 L flask. After vacuumed and backfilled with nitrogen, *o*-xylene (600 mL) was added. The mixture stirred at rt for 20 min. Under a nitrogen flow, *t*-BuONa (10.8 g, 112 mmol, 2.0 equiv.) was finally added. The reaction mixture stirred at 100 °C for 36 hours. After cooling to rt, the mixture was passed through a pad of celite, rinsed with DCM. After removal of the solvent, the residue was dissolved in DCM (25 mL). MeOH (350 mL) was added to form a suspension, which was collected by filtration and dried under vacuum. Compound **S3** (22.6 g, 83%) was obtained as white powder.

^{1}**H NMR** (400 MHz, $CDCl_3$) δ 8.12 (d, *J* = 7.7 Hz, 2H), 7.47 – 7.39 (m, 4H), 7.34 – 7.26 (m, 4H), 7.14 (dd, *J* = 7.6, 1.3 Hz, 2H), 7.07 (t, *J* = 1.8 Hz, 1H), 7.04 (t, *J* = 2.0 Hz, 1H), 6.98 (d, *J* = 4.4 Hz, 4H), 2.33 (s, 3H), 2.11 (s, 6H).

^{13}**C NMR** (101 MHz, $CDCl_3$) δ 149.12, 144.83, 140.46, 139.49, 139.37, 137.66, 137.35,

135.97, 130.39, 129.49, 126.12, 123.56, 122.61, 121.18, 120.43, 120.26, 118.27, 116.90, 115.12, 109.94, 21.17, 18.70.

HRMS (MALDI-TOF) m/z: $[M]^+$ Calculated for $C_{33}H_{27}ClN_2$ 486.1857; Found 486.1906 (10.1 ppm).

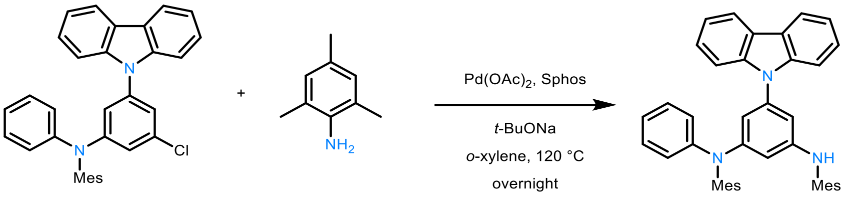

S3 **S4**

Compound **S3** (10.0 g, 20.5 mmol, 1.0 equiv.), $Pd(OAc)_2$ (230 mg, 5 mol%), Sphos (500 mg, 6 mol%) and 2,4,6-trimethylaniline (5.6 g, 41.0 mmol, 2.0 equiv.) were added to a 1 L flask. After vacuumed and backfilled with nitrogen, *o*-xylene (500 mL) was added. The mixture stirred at rt for 20 min. Under a nitrogen flow, *t*-BuONa (4.9 g, 51 mmol, 2.5 equiv.) was finally added. The reaction mixture stirred at 120 °C overnight. After cooling to rt, the mixture was passed through a pad of celite, rinsed with DCM. After removal of the solvent, the residue was dissolved in DCM (20 mL). MeOH (200 mL) was added to form a suspension, which was collected by filtration and dried under vacuum. compound **S4** (8.9 g, 74%) was obtained as white powder.

^{1}H NMR (400 MHz, $CDCl_3$) δ 8.06 (dt, J = 7.8, 1.0 Hz, 2H), 7.38 – 7.33 (m, 4H), 7.24 – 7.16 (m, 4H), 7.10 – 7.06 (m, 2H), 6.92 (s, 2H), 6.89 – 6.82 (m, 3H), 6.46 (t, J = 1.9 Hz, 1H), 6.32 (t, J = 2.1 Hz, 1H), 6.03 (t, J = 1.9 Hz, 1H), 5.09 (s, 1H), 2.31 (s, 3H), 2.22 (d, J = 4.5 Hz, 9H), 2.08 (s, 6H).

^{13}C NMR (101 MHz, $CDCl_3$) δ 149.15, 148.55, 145.73, 140.76, 140.23, 139.23, 137.60, 136.92, 136.12, 135.87, 135.20, 130.07, 129.34, 129.14, 125.74, 123.19, 121.23, 120.51, 120.20, 119.53, 110.27, 108.03, 103.72, 103.36, 21.16, 20.97, 18.71, 18.36.

HRMS (MALDI-TOF) m/z: $[M]^+$ Calculated for $C_{42}H_{39}N_3$ 585.3138; Found 585.3230 (15.7 ppm).

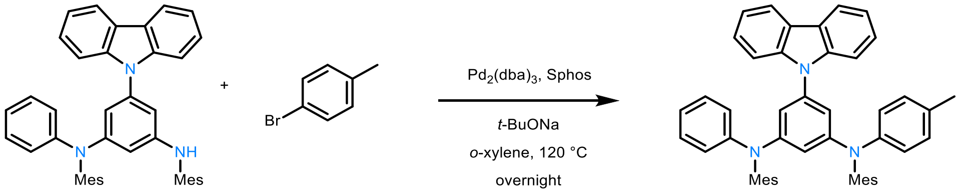

S4 **S5**

Compound **S4** (800 mg, 1.37 mmol, 1.0 equiv.), $Pd_2(dba)_3$ (62 mg, 5 mol%), Sphos (56 mg, 10 mol%) and 1-bromo-4-methylbenzene (470 mg, 2.73 mmol, 2.0 equiv.) were added to a 500 mL flask. After vacuumed and backfilled with nitrogen, *o*-xylene (100 mL) was added. The mixture stirred at rt for 20 min. Under a nitrogen flow, *t*-BuONa (400 mg, 4.1 mmol, 3.0 equiv.) was finally added. The reaction mixture stirred at 120 °C overnight. After cooling to rt, the mixture was passed through a pad of celite, rinsed with DCM. After removal of the solvent, the residue was purified by column chromatography using hexane/DCM (v/v = 4:1) as the eluents to give compound **S5** (852

mg, 92%) as white solid.

^{1}H NMR (600 MHz, $CDCl_3$) δ 8.05 (d, J = 7.7 Hz, 2H), 7.35 – 7.28 (m, 4H), 7.23 – 7.15 (m, 4H), 7.02 (dt, J = 7.0, 1.1 Hz, 2H), 6.98 (d, J = 8.4 Hz, 2H), 6.96 – 6.92 (m, 2H), 6.90 (t, J = 2.1 Hz, 1H), 6.88 – 6.82 (m, 5H), 6.44 (p, J = 1.9 Hz, 2H), 2.27 – 2.25 (m. 9H), 2.04 (m, 12H).

^{13}C NMR (151 MHz, $CDCl_3$) δ 148.37, 147.99, 145.67, 142.95, 140.53, 139.95, 139.90, 139.10, 137.40, 137.37, 136.96, 136.87, 130.68, 130.05, 130.03, 129.70, 129.13, 125.79, 123.27, 121.02, 120.21, 120.07, 119.66, 110.18, 109.80, 109.72, 108.84, 21.11, 20.78, 18.54, 18.53.

HRMS (MALDI-TOF) m/z: $[M]^+$ Calculated for $C_{49}H_{45}N_3$ 675.3608; Found 675.3683 (11.1 ppm).

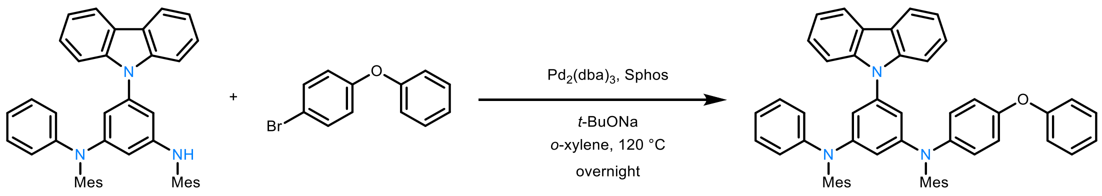

S4 **S6**

Compound **S4** (800 mg, 1.37 mmol, 1.0 equiv.), $Pd_2(dba)_3$ (62 mg, 5 mol%), Sphos (56 mg, 10 mol%) and 1-bromo-4-phenoxybenzene (680 mg, 2.73 mmol, 2.0 equiv.) were added to a 500 mL flask. After vacuumed and backfilled with nitrogen, *o*-xylene (100 mL) was added. The mixture stirred at rt for 20 min. Under a nitrogen flow, *t*-BuONa (400 mg, 4.1 mmol, 3.0 equiv.) was finally added. The reaction mixture stirred at 120 °C overnight. After cooling to rt, the mixture was passed through a pad of celite, rinsed with DCM. After removal of the solvent, the residue was purified by column chromatography using hexane/DCM (v/v = 3:1) as the eluents to give compound **S6** (740 mg, 72%) as white solid.

^{1}H NMR (400 MHz, $CDCl_3$) δ 8.06 (d, J = 7.7 Hz, 2H), 7.38 – 7.28 (m, 6H), 7.25 – 7.15 (m, 4H), 7.12 – 6.99 (m, 6H), 6.99 – 6.92 (m, 3H), 6.88 (q, J = 3.0 Hz, 7H), 6.45 (t, J = 1.9 Hz, 1H), 6.42 (t, J = 1.9 Hz, 1H), 2.26 (s, 6H), 2.08 (s, 6H), 2.06 (s, 6H).

^{13}C NMR (101 MHz, $CDCl_3$) δ 158.29, 150.83, 148.49, 148.13, 145.49, 141.61, 140.52, 139.82, 139.78, 139.26, 137.39, 137.27, 137.07, 137.04, 130.13, 130.08, 129.75, 129.16, 125.82, 123.28, 122.69, 121.79, 121.19, 120.38, 120.25, 119.88, 119.71, 117.95, 110.14, 109.71, 109.47, 108.31, 27.06, 21.11, 18.63, 18.54.

HRMS (MALDI-TOF) m/z: $[M]^+$ Calculated for $C_{54}H_{47}N_3O$ 753.3714; Found 753.3763 (6.1 ppm).

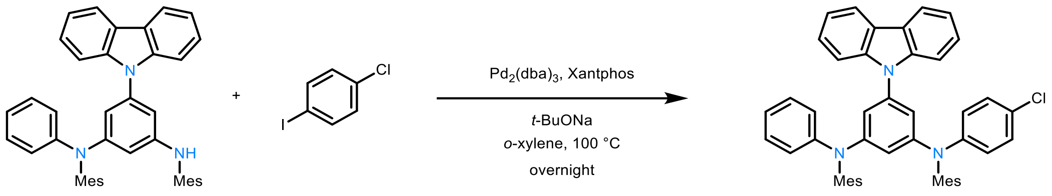

S4 **S7**

Compound **S4** (3.0 g, 5.12 mmol, 1.0 equiv.), $Pd_2(dba)_3$ (234 mg, 5 mol%), Xantphos (296 mg, 10 mol%) and 1-chloro-4-iodobenzene (2.44 g, 20.24 mmol, 2.0 equiv.) were added to a 500 mL

flask. After vacuumed and backfilled with nitrogen, *o*-xylene (150 mL) was added. The mixture stirred at rt for 20 min. Under a nitrogen flow, *t*-BuONa (1.0 g, 10.2 mmol, 2.0 equiv.) was finally added. The reaction mixture stirred at 100 °C overnight. After cooling to rt, the mixture was passed through a pad of celite, rinsed with DCM. After removal of the solvent, the residue was purified by column chromatography using hexane/DCM (v/v = 5:1) as the eluents to give compound **S7** (2.86 g, 80%) as white solid.

^{1}H NMR (600 MHz, $CDCl_3$) δ 8.06 (d, *J* = 7.6 Hz, 2H), 7.33 (ddd, *J* = 8.2, 7.0, 1.2 Hz, 2H), 7.28 (d, *J* = 8.2 Hz, 2H), 7.24 – 7.17 (m, 4H), 7.13 – 7.09 (m, 2H), 7.06 – 7.01 (m, 2H), 6.96 – 6.92 (m, 2H), 6.91 – 6.86 (m, 6H), 6.52 (d, *J* = 1.9 Hz, 1H), 6.45 (d, *J* = 2.0 Hz, 1H), 2.28 (s, 3H), 2.27 (s, 3H), 2.04 (s, 6H), 2.03 (s, 6H).

^{13}C NMR (151 MHz, $CDCl_3$) δ 148.22, 147.68, 145.47, 144.28, 140.46, 139.79, 139.45, 139.31, 137.34, 137.22, 137.14, 130.19, 130.12, 129.21, 129.19, 129.14, 125.85, 125.68, 123.34, 121.39, 120.71, 120.28, 119.89, 119.80, 110.47, 110.20, 110.06, 109.21, 21.11, 18.53, 18.48.

HRMS (MALDI-TOF) m/z: $[M]^+$ Calculated for $C_{48}H_{42}ClN_3$ 695.3062; Found 695.3137 (10.7 ppm).

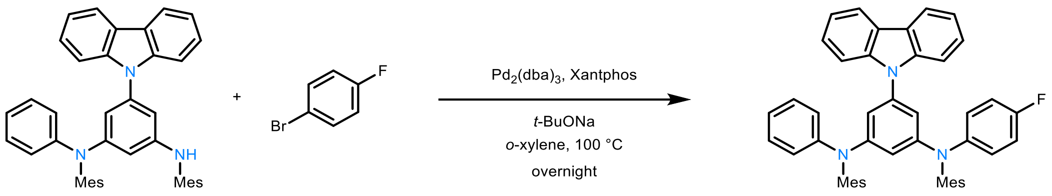

S4 **S8**

Compound **S4** (1.0 g, 1.71 mmol, 1.0 equiv.), $Pd_2(dba)_3$ (78 mg, 5 mol%), Xantphos (98 mg, 10 mol%) and 1-bromo-4-fluorobenzene (814 mg, 3.41 mmol, 2.0 equiv.) were added to a 500 mL flask. After vacuumed and backfilled with nitrogen, *o*-xylene (150 mL) was added. The mixture stirred at rt for 20 min. Under a nitrogen flow, *t*-BuONa (320 mg, 3.41 mmol, 2.0 equiv.) was finally added. The reaction mixture stirred at 100 °C overnight. After cooling to rt, the mixture was passed through a pad of celite, rinsed with DCM. After removal of the solvent, the residue was purified by column chromatography using hexane/DCM (v/v = 5:1) as the eluents to give compound **S8** (960 mg, 81%) as white solid.

^{1}H NMR (600 MHz, $CDCl_3$) δ 8.06 (dd, *J* = 7.6, 2.2 Hz, 2H), 7.37 – 7.33 (m, 2H), 7.30 (dd, *J* = 8.3, 2.5 Hz, 2H), 7.25 – 7.18 (m, 4H), 7.06 – 6.98 (m, 4H), 6.92 – 6.83 (m, 8H), 6.49 (q, *J* = 1.9 Hz, 1H), 6.43 (q, *J* = 1.9 Hz, 1H), 2.28 (m, 6H), 2.06 (s, 12H).

^{13}C NMR (151 MHz, $CDCl_3$) δ 158.50, 156.91, 148.37, 148.20, 145.52, 141.73, 141.72, 140.51, 139.83, 139.78, 139.28, 137.37, 137.24, 137.14, 137.08, 130.17, 130.09, 129.19, 129.16, 128.38, 125.82, 123.31, 121.59, 121.54, 121.26, 120.26, 119.85, 119.75, 115.84, 115.69, 110.10, 109.91, 109.49, 108.39, 21.10, 18.55, 18.53.

^{19}F NMR (565 MHz, $CDCl_3$) δ -122.23.

HRMS (MALDI-TOF) m/z: $[M]^+$ Calculated for $C_{48}H_{42}FN_3$ 679.3357; Found 679.3319 (-5.6 ppm).

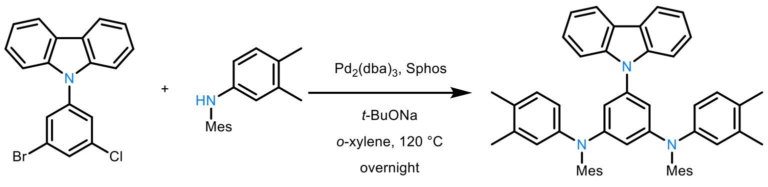

S1 **S9**

Compound **S1** (650 mg, 1.82 mmol, 1.0 equiv.), $Pd_2(dba)_3$ (83 mg, 5 mol%), Sphos (105 mg, 10 mol%) and *N*-(3,4-dimethylphenyl)-2,4,6-trimethylaniline (9.16 g, 3.83 mmol, 2.1 equiv.) were added to a 500 mL flask. After vacuumed and backfilled with nitrogen, *o*-xylene (100 mL) was added. The mixture stirred at rt for 20 min. Under a nitrogen flow, *t*-BuONa (530 mg, 5.5 mmol, 3.0 equiv.) was finally added. The reaction mixture stirred at 120 °C overnight. After cooling to rt, the mixture was passed through a pad of celite, rinsed with DCM. After removal of the solvent, the residue was purified by column chromatography using hexane/DCM (v/v = 6:1) as the eluents to give compound **S9** (1.12 g, 85%) as white solid.

^{1}H NMR (600 MHz, Acetone-*d6*) δ 8.12 (dt, *J* = 7.7, 1.0 Hz, 2H), 7.42 (dt, *J* = 8.3, 1.0 Hz, 2H), 7.38 (ddd, *J* = 8.2, 6.9, 1.2 Hz, 2H), 7.22 (ddd, *J* = 7.9, 6.9, 1.1 Hz, 2H), 6.94 (s, 4H), 6.69 (d, *J* = 1.5 Hz, 4H), 6.64 (t, *J* = 2.1 Hz, 1H), 6.54 (d, *J* = 2.1 Hz, 4H), 2.25 (s, 6H), 2.18 (s, 12H), 2.07 (s, 12H).

^{13}C NMR (151 MHz, Acetone-*d6*) δ 149.40, 146.20, 141.10, 140.93, 139.61, 139.36, 138.00, 137.73, 130.77, 126.77, 124.26, 124.18, 121.15, 120.84, 118.81, 118.32, 110.74, 109.84, 109.19, 21.56, 21.03, 18.72.

HRMS (MALDI-TOF) m/z: $[M]^+$ Calculated for $C_{52}H_{51}N_3$ 717.4078; Found 717.4128 (6.9 ppm).

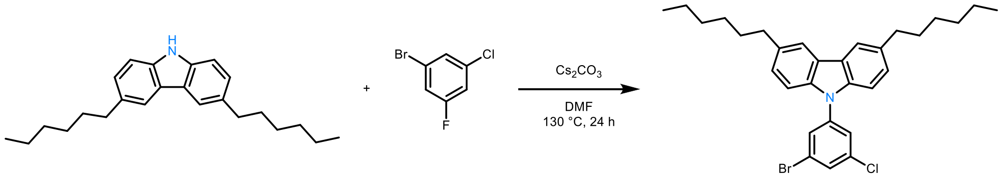

S10

3,6-Dihexyl-9*H*-carbazole (4.5 g, 13.4 mmol, 1.0 equiv.) was dissolved in DMF (20 mL). Cs_2CO_3 (8.7 g, 13.4 mmol, 2.0 equiv.) and 1-bromo-3-chloro-5-fluorobenzene (3.6 g, 17.4 mmol, 1.3 equiv.) were added at rt. The reaction mixture stirred at 135 °C for 24 hours. After cooling to rt, the reaction mixture poured into NH_4Cl (aq.) with rigorous stirring. Extracted with DCM. After work-up, the residue was purified by column chromatography using hexane/DCM (v/v = 1:0 to 10:1) as the eluents to give compound **S10** (6.08 g, 86%) as colorless oil.

^{1}H NMR (400 MHz, $CDCl_3$) δ 7.93 (s, 2H), 7.67 (t, *J* = 1.6 Hz, 1H), 7.58 (t, *J* = 1.5 Hz, 1H), 7.56 (q, *J* = 1.5 Hz, 1H), 7.36 (d, *J* = 8.3 Hz, 2H), 7.28 – 7.24 (m, 2H), 2.82 (t, *J* = 7.7 Hz, 4H), 1.82 – 1.67 (m, 4H), 1.43 (t, *J* = 7.3 Hz, 4H), 1.00 – 0.93 (m, 10H), 0.60 – 0.51 (m, 5H).

^{13}C NMR (101 MHz, $CDCl_3$) δ 140.56, 138.88, 136.24, 135.49, 129.82, 128.03, 126.97, 125.65, 124.00, 123.55, 119.89, 109.31, 36.13, 32.39, 31.98, 29.19, 22.82, 14.30, 6.97, 6.57.

HRMS (MALDI-TOF) m/z: $[M]^+$ Calculated for $C_{30}H_{35}BrClN$ 523.1617; Found 523.1488 (-24.6 ppm).

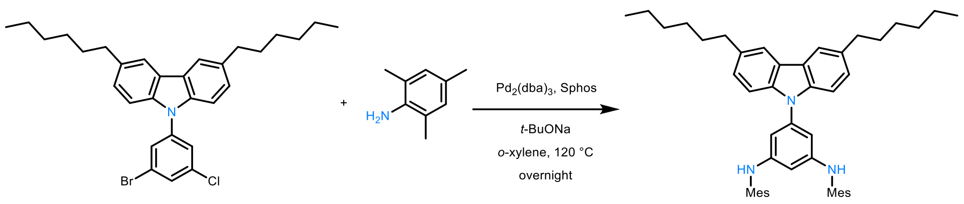

S10 **S11**

Compound **S10** (6.0 g, 11.4 mmol, 1.0 equiv.), $Pd_2(dba)_3$ (523 mg, 5 mol%), Sphos (660 mg, 10 mol%) and 2,4,6-trimethylaniline (4.6 g, 34.3 mmol, 3.0 equiv.) were added to a 500 mL flask. After vacuumed and backfilled with nitrogen, *o*-xylene (200 mL) was added. The mixture stirred at rt for 20 min. Under a nitrogen flow, *t*-BuONa (4.4 g, 45.7 mmol, 4.0 equiv.) was finally added. The reaction mixture stirred at 120 °C overnight. After cooling to rt, the mixture was passed through a pad of celite, rinsed with DCM. After removal of the solvent, the residue was purified by column chromatography using hexane/DCM (v/v = 6:1 to 4:1) as the eluents to give compound **S11** (6.45 g, 83%) as sticky oil, which solidified gradually.

^{1}H NMR (400 MHz, $CDCl_3$) δ 8.03 (d, *J* = 1.7 Hz, 2H), 7.52 (d, *J* = 8.4 Hz, 2H), 7.36 (dd, *J* = 8.4, 1.7 Hz, 2H), 7.07 (s, 4H), 6.15 (d, *J* = 1.9 Hz, 2H), 5.94 (s, 1H), 5.19 (br, 2H), 2.95 (t, *J* = 7.7 Hz, 4H), 1.45 – 1.43 (m, 18H), 1.89 (qd, *J* = 8.2, 6.1 Hz, 4H), 1.64 – 1.47 (m, 12H), 1.13 – 1.06 (m, 6H).

^{13}C NMR (101 MHz, $CDCl_3$) δ 149.11, 139.99, 139.60, 136.21, 135.66, 135.51, 133.83, 129.31, 126.28, 123.17, 119.37, 110.03, 102.03, 97.25, 36.15, 32.46, 31.99, 29.22, 22.82, 20.99, 18.44, 14.30.

HRMS (MALDI-TOF) m/z: $[M]^+$ Calculated for $C_{48}H_{59}N_3$ 677.4704; Found 677.4780 (11.2 ppm).

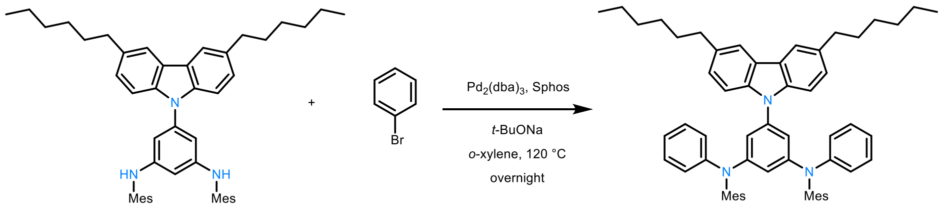

S11 **S12**

Compound **S11** (5.0 g, 7.37 mmol, 1.0 equiv.), $Pd_2(dba)_3$ (337 mg, 5 mol%), Sphos (426mg, 10 mol%) and bromobenzene (4.6 g, 29.5 mmol, 4.0 equiv.) were added to a 500 mL flask. After vacuumed and backfilled with nitrogen, *o*-xylene (200 mL) was added. The mixture stirred at rt for 20 min. Under a nitrogen flow, *t*-BuONa (2.8 g, 29.5 mmol, 4.0 equiv.) was finally added. The reaction mixture stirred at 120 °C overnight. After cooling to rt, the mixture was passed through a pad of celite, rinsed with DCM. After removal of the solvent, the residue was dissolved in DCM (10 mL). EtOH (150 mL) was added to form a suspension, which was collected by filtration and dried under vacuum. Compound **S12** (4.35 g, 71%) was obtained as white powder.

^{1}H NMR (400 MHz, $CDCl_3$) δ 7.82 (s, 2H), 7.22 – 7.11 (m, 8H), 7.06 – 7.00 (m, 4H), 6.93 (d, *J* = 2.1 Hz, 1H), 6.86 (d, *J* = 12.0 Hz, 6H), 6.48 (d, *J* = 2.0 Hz, 2H), 2.75 (t, *J* = 7.7 Hz, 4H), 2.28 (s, 6H), 2.04 (s, 12H), 1.69 (p, *J* = 7.5 Hz, 4H), 1.40 – 1.27 (m, 12H), 1.00 – 0.84 (m, 6H).

^{13}C NMR (101 MHz, $CDCl_3$) δ 147.89, 145.68, 139.88, 139.59, 139.07, 137.39, 136.94, 134.21, 130.06, 129.13, 126.38, 123.34, 121.00, 119.65, 119.45, 110.16, 109.76, 109.15, 36.11, 32.43, 31.97, 29.21, 22.80, 21.12, 18.53, 14.30.

HRMS (MALDI-TOF) m/z: $[M]^+$ Calculated for $C_{60}H_{67}N_3$ 829.5330; Found 829.5443 (13.6 ppm).

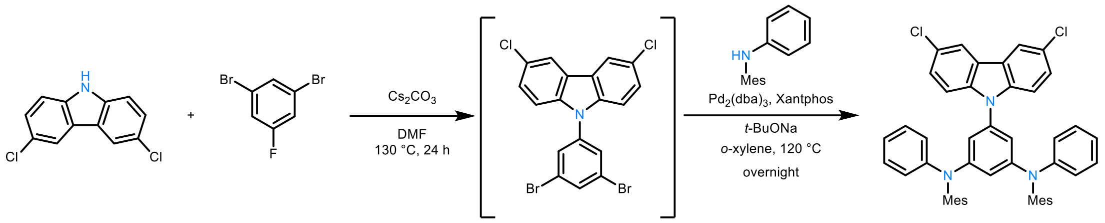

S13

3,6-Dichloro-9*H*-carbazole (4.0 g, 16.9 mmol, 1.0 equiv.) was dissolved in DMF (30 mL). Cs_2CO_3 (8.3 g, 25.4 mmol, 1.5 equiv.) and 1,3-dibromo-5-fluorobenzene (6.45 g, 25.4 mmol, 1.5 equiv.) were added at rt. The reaction mixture stirred at 135 °C for 24 hour. After cooling to rt, the reaction mixture poured into NH_4Cl (aq.) with rigorous stirring. The precipitation was collected by filtration and suspended in DCM (10 mL). MeOH (200 mL) was finally added to form a suspension, which was further filtered and dried under vacuum to obtain the intermediate as off-white fine powder (6.43 g, 81%).

The intermediate (1.5 g, 3.2 mmol, 1.0 equiv.), $Pd_2(dba)_3$/Xantphos (80 mg/100 mg) and 2,4,6-trimethyl-*N*-phenylaniline (1.5 g, 7.0 mmol, 2.2 equiv.) were added to a 500 mL flask. After vacuumed and backfilled with nitrogen, *o*-xylene (250 mL) was added. The mixture stirred at rt for 20 min. Under a nitrogen flow, *t*-BuONa (1.23 g, 12.8 mmol, 4.0 equiv.) was finally added. The reaction mixture stirred at 100 °C overnight. After cooling to rt, the mixture was passed through a pad of celite, rinsed with DCM. After removal of the solvent, the residue was purified by column chromatography using hexane/DCM (v/v = 4:1) as the eluents to give compound **S13** (1.82 g, 78%) as white solid.

^{1}H NMR (600 MHz, $CDCl_3$) δ 7.90 (d, J = 2.1 Hz, 2H), 7.30 – 7.26 (m, 2H), 7.21 – 7.17 (m, 6H), 7.06 – 7.02 (m, 4H), 6.97 (q, J = 2.2 Hz, 1H), 6.91 – 6.86 (m, 6H), 6.37 (t, J = 1.7 Hz, 2H), 2.28 (s, 6H), 2.05 (s, 12H).

^{13}C NMR (151 MHz, $CDCl_3$) δ 148.34, 145.37, 139.73, 139.28, 138.48, 137.34, 137.21, 130.13, 129.20, 129.18, 128.37, 126.59, 125.51, 123.42, 121.46, 120.03, 119.96, 111.40, 109.43, 109.14, 21.13, 18.52.

HRMS (MALDI-TOF) m/z: $[M]^+$ Calculated for $C_{48}H_{41}Cl_2N_3$ 729.2672; Found 729.2758 (11.8 ppm).

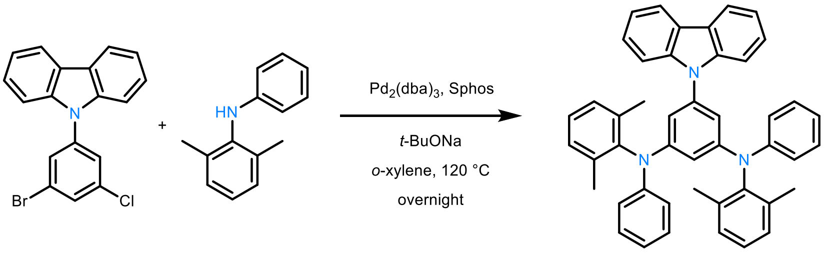

S1 **S14**

Compound **S1** (1.2 g, 3.36 mmol, 1.0 equiv.), $Pd_2(dba)_3$ (154 mg, 5 mol%), Sphos (138 mg, 10 mol%) and 2,6-dimethyl-*N*-phenylaniline (1.45 g, 7.07 mmol, 2.1 equiv.) were added to a 500 mL

flask. After vacuumed and backfilled with nitrogen, *o*-xylene (120 mL) was added. The mixture stirred at rt for 20 min. Under a nitrogen flow, *t*-BuONa (970 mg, 10.1 mmol, 3.0 equiv.) was finally added. The reaction mixture stirred at 120 °C overnight. After cooling to rt, the mixture was passed through a pad of celite, rinsed with DCM. After removal of the solvent, the residue was purified by column chromatography using hexane/DCM (v/v = 3:1) as the eluents to give compound **S14** (1.62 g, 74%) as white solid.

^{1}H NMR (400 MHz, $CDCl_3$) δ 8.06 (d, *J* = 7.7 Hz, 2H), 7.37 – 7.27 (m, 4H), 7.21 (dt, *J* = 10.8, 7.2 Hz, 6H), 7.13 – 7.01 (m, 10H), 6.96 (d, *J* = 2.1 Hz, 1H), 6.88 (t, *J* = 7.3 Hz, 2H), 6.50 (d, *J* = 2.0 Hz, 2H), 2.10 (s, 12H).

^{13}C NMR (101 MHz, $CDCl_3$) δ 147.99, 145.41, 142.38, 140.43, 139.27, 137.77, 129.42, 129.21, 127.47, 125.84, 123.32, 121.34, 120.26, 119.84, 119.78, 110.30, 110.05, 109.36, 18.68.

HRMS (MALDI-TOF) m/z: $[M]^+$ Calculated for $C_{46}H_{39}N_3$ 633.3138; Found 633.3187 (7.7 ppm).

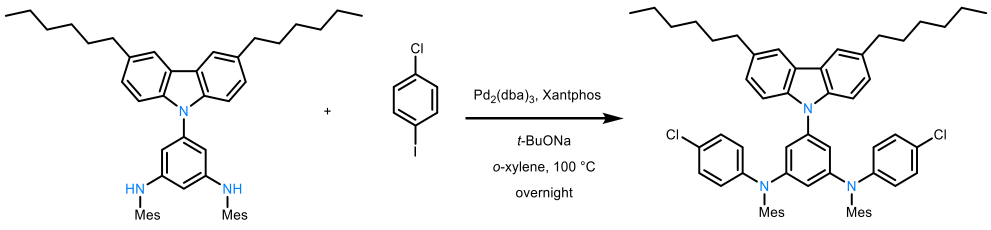

S11 **S15**

Compound **S11** (1.0 g, 1.47 mmol, 1.0 equiv.), $Pd_2(dba)_3$ (67 mg, 5 mol%), Xantphos (85mg, 10 mol%) and 1-chloro-4-iodobenzene (1.06 g, 4.42 mmol, 3.0 equiv.) were added to a 500 mL flask. After vacuumed and backfilled with nitrogen, *o*-xylene (100 mL) was added. The mixture stirred at rt for 20 min. Under a nitrogen flow, *t*-BuONa (560 mg, 5.9 mmol, 4.0 equiv.) was finally added. The reaction mixture stirred at 100 °C overnight. After cooling to rt, the mixture was passed through a pad of celite, rinsed with DCM. After removal of the solvent, the residue was purified by column chromatography using hexane/DCM (v/v = 5:1) as the eluents to give compound **S15** (1.15 g, 87%) as white solid.

^{1}H NMR (600 MHz, $CDCl_3$) δ 7.82 (s, 2H), 7.53 – 7.34 (m, 2H), 7.16 – 7.08 (m, 7H), 6.93 – 6.90 (m, 3H), 6.88 (s, 4H), 6.73 (t, *J* = 2.1 Hz, 1H), 6.51 (d, *J* = 2.1 Hz, 2H), 2.74 (t, *J* = 7.8 Hz, 4H), 2.28 (s, 6H), 2.01 (s, 12H), 1.68 (p, *J* = 7.5 Hz, 4H), 1.43 – 1.27 (m, 12H), 0.96 – 0.84 (m, 6H).

^{13}C NMR (151 MHz, $CDCl_3$) δ 147.70, 144.24, 139.84, 139.47, 139.00, 137.37, 137.19, 134.43, 130.21, 130.17, 130.13, 129.20, 129.18, 129.11, 128.37, 126.46, 125.76, 123.45, 121.12, 120.72, 119.56, 110.43, 109.59, 109.07, 36.10, 32.41, 31.96, 29.21, 22.79, 21.10, 18.47, 14.28.

HRMS (MALDI-TOF) m/z: $[M]^+$ Calculated for $C_{60}H_{65}Cl_2N_3$ 897.4550; Found 897.4585 (3.9 ppm).

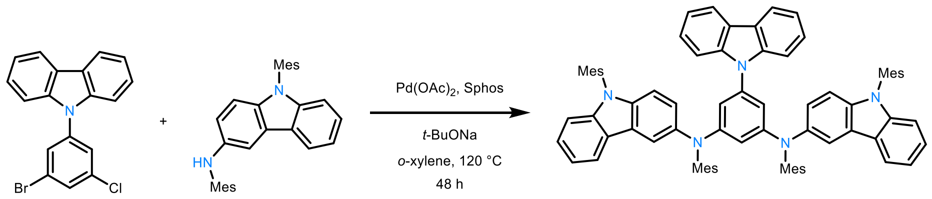

S1 **S16**

Compound **S1** (1.0 g, 2.80 mmol, 1.0 equiv.), Pd(OAc)$_2$ (31 mg, 5 mol%), Sphos (92 mg, 8 mol%) and *N*,9-dimesityl-9*H*-carbazol-3-amine (2.58 g, 6.17 mmol, 2.2 equiv.) were added to a 500 mL flask. After vacuumed and backfilled with nitrogen, *o*-xylene (100 mL) was added. The mixture stirred at rt for 20 min. Under a nitrogen flow, *t*-BuONa (1.08 mg, 11.2 mmol, 4.0 equiv.) was finally added. The reaction mixture stirred at 120 °C for 48 hours in the dark. After cooling to rt, the mixture was passed through a pad of celite, rinsed with DCM. After removal of the solvent, the residue was dissolved in DCM (15 mL). MeOH (150 mL) was added to form a pink suspension, which was collected by filtration and dried under vacuum. Compound **S16** (2.85 g, 94%) was obtained as light pink powder.

^{1}H NMR (400 MHz, DMSO-*d6*) δ 8.20 (d, *J* = 7.8 Hz, 2H), 8.14 (d, *J* = 7.8 Hz, 2H), 7.99 (d, *J* = 2.2 Hz, 2H), 7.39 – 7.28 (m, 6H), 7.24 – 7.17 (m, 4H), 7.07 (d, *J* = 2.9 Hz, 6H), 6.91 (s, 4H), 6.75 (dd, *J* = 12.7, 8.4 Hz, 4H), 6.12 (d, *J* = 2.0 Hz, 2H), 5.76 (s, 1H), 2.38 (s, 6H), 2.13 (s, 6H), 2.09 (s, 12H), 1.54 (s, 12H).

^{13}C NMR (151 MHz, Acetone-*d6*) δ 150.49, 141.80, 141.61, 141.15, 140.05, 139.29, 139.03, 138.08, 138.05, 137.47, 137.19, 132.54, 130.81, 130.36, 130.31, 127.06, 126.72, 124.19, 124.08, 123.37, 122.05, 121.49, 121.04, 120.65, 120.09, 114.09, 110.86, 110.52, 110.06, 107.54, 105.55, 21.22, 20.96, 18.98, 17.45.

HRMS (MALDI-TOF) m/z: [M]$^+$ Calculated for $C_{78}H_{69}N_5$ 1075.5547; Found 1075.5556 (0.8 ppm).

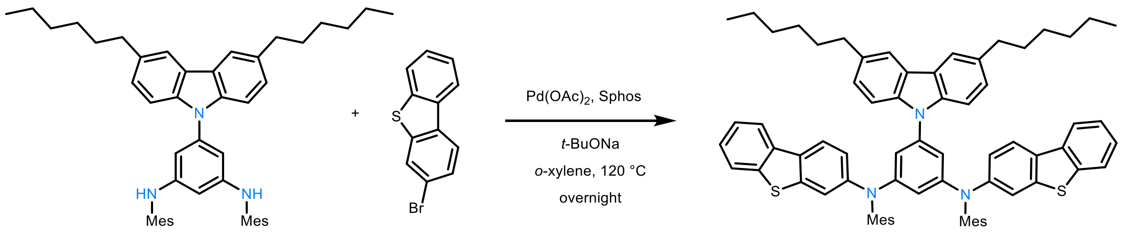

S11 **S17**

Compound **S11** (1.2 g, 2.06 mmol, 1.0 equiv.), Pd(OAc)$_2$/Sphos (50 mg/140 mg) and 3-bromodibenzo[*b*,*d*]thiophene (980 mg, 3.72 mmol, 2.1 equiv.) were added to a 500 mL flask. After vacuumed and backfilled with nitrogen, *o*-xylene (120 mL) was added. The mixture stirred at rt for 20 min. Under a nitrogen flow, *t*-BuONa (680 mg, 7.1 mmol, 4.0 equiv.) was finally added. The reaction mixture stirred at 120 °C overnight. After cooling to rt, the mixture was passed through a pad of celite, rinsed with DCM. After removal of the solvent, the residue was purified by column chromatography using hexane/DCM (v/v = 3:1) as the eluents to give compound **S17** (1.41 g, 76%) as white solid.

^{1}H NMR (400 MHz, $CDCl_3$) δ 7.96 (dd, *J* = 12.6, 7.8 Hz, 4H), 7.83 (d, *J* = 1.7 Hz, 2H), 7.77 (dd, *J* = 7.3, 1.4 Hz, 2H), 7.43 (d, *J* = 2.1 Hz, 2H), 7.37 (dtd, *J* = 16.6, 7.3, 1.3 Hz, 4H), 7.26 – 7.14 (m, 6H), 7.02 (d, *J* = 2.1 Hz, 1H), 6.92 (s, 4H), 6.62 (d, *J* = 2.0 Hz, 2H), 2.75 (t, *J* = 7.7 Hz, 4H), 2.30 (s, 6H), 2.09 (s, 12H), 1.68 (p, *J* = 7.7 Hz, 4H), 1.41 – 1.29 (m, 12H), 0.92 – 0.87 (m, 6H).

^{13}C NMR (101 MHz, $CDCl_3$) δ 147.95, 145.02, 140.99, 139.88, 139.78, 138.97, 138.88, 137.30, 135.68, 134.40, 130.29, 129.45, 126.50, 125.60, 124.47, 123.47, 122.70, 122.11, 120.78, 119.52, 117.41, 112.55, 110.84, 109.72, 36.10, 32.41, 31.96, 29.20, 22.79, 21.17, 18.63, 14.29.

HRMS (MALDI-TOF) m/z: [M]$^+$ Calculated for $C_{72}H_{71}N_3S_2$ 1041.5084; Found 1041.5057 (-2.6 ppm).

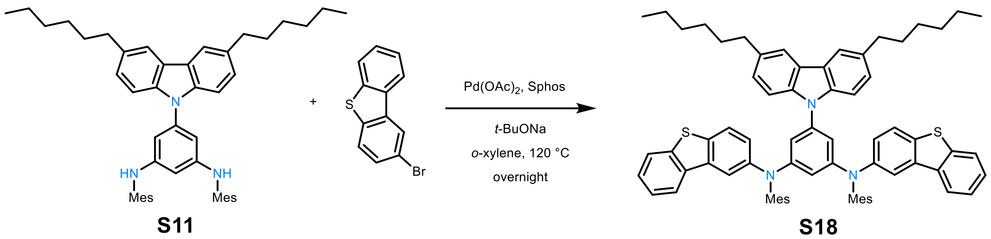

Compound **S11** (1.5 g, 2.21 mmol, 1.0 equiv.), Pd(OAc)$_2$/Sphos (100 mg/300 mg) and 2-bromodibenzo[*b*,*d*]thiophene (1.28 g, 4.87 mmol, 2.2 equiv.) were added to a 500 mL flask. After vacuumed and backfilled with nitrogen, *o*-xylene (150 mL) was added. The mixture stirred at rt for 20 min. Under a nitrogen flow, *t*-BuONa (850 mg, 8.8 mmol, 4.0 equiv.) was finally added. The reaction mixture stirred at 120 °C overnight. After cooling to rt, the mixture was passed through a pad of celite, rinsed with DCM. After removal of the solvent, the residue was dissolved in DCM (15 mL). MeOH (150 mL) was added to form a suspension, which was collected by filtration and dried under vacuum. Compound **S18** (2.14 g, 93%) was obtained as white powder.

^{1}H NMR (400 MHz, CDCl$_3$) δ 8.08 – 7.99 (m, 2H), 7.86 – 7.76 (m, 6H), 7.54 (d, *J* = 8.7 Hz, 2H), 7.42 (tt, *J* = 7.2, 5.5 Hz, 4H), 7.30 (d, *J* = 8.4 Hz, 2H), 7.19 (dd, *J* = 8.7, 2.3 Hz, 2H), 7.12 – 7.08 (m, 2H), 6.88 (s, 4H), 6.85 (t, *J* = 2.1 Hz, 1H), 6.65 (d, *J* = 2.1 Hz, 2H), 2.73 (t, *J* = 7.7 Hz, 4H), 2.25 (s, 6H), 2.10 (s, 12H), 1.67 (dq, *J* = 15.1, 7.0 Hz, 4H), 1.41 – 1.26 (m, 12H), 0.95 – 0.86 (m, 6H).

^{13}C NMR (101 MHz, CDCl$_3$) δ 148.52, 143.24, 140.47, 140.00, 139.85, 139.03, 137.27, 137.08, 136.62, 135.40, 134.29, 131.92, 130.22, 126.78, 126.45, 124.16, 123.45, 123.26, 123.04, 121.82, 120.49, 119.51, 112.49, 109.82, 109.75, 108.36, 36.07, 32.33, 31.95, 29.17, 22.78, 21.09, 18.73, 14.29.

HRMS (MALDI-TOF) m/z: [M]$^+$ Calculated for $C_{72}H_{71}N_3S_2$ 1041.5084; Found 1041.4935 (-14.3 ppm).

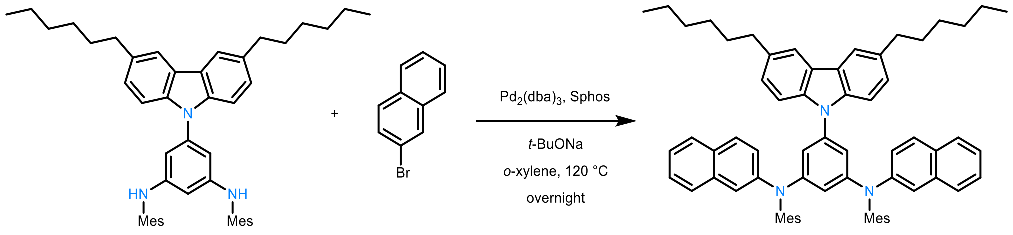

Compound **S11** (800 mg, 1.18 mmol, 1.0 equiv.), Pd$_2$(dba)$_3$/Sphos (54 mg/50 mg) and 2-bromonaphthalene (640 g, 3.07 mmol, 2.6 equiv.) were added to a 500 mL flask. After vacuumed and backfilled with nitrogen, *o*-xylene (80 mL) was added. The mixture stirred at rt for 20 min. Under a nitrogen flow, *t*-BuONa (450 mg, 4.7 mmol, 4.0 equiv.) was finally added. The reaction mixture stirred at 120 °C overnight. After cooling to rt, the mixture was passed through a pad of celite, rinsed with DCM. After removal of the solvent, the residue was purified by column chromatography using hexane/DCM (v/v = 4:1) as the eluents to give compound **S19** (860 mg, 78%) as white solid.

^{1}H NMR (600 MHz, CDCl$_3$) δ 7.81 (d, *J* = 1.7 Hz, 2H), 7.65 (dd, *J* = 17.3, 8.5 Hz, 4H), 7.55 (d, *J* = 8.2 Hz, 2H), 7.35 (ddt, *J* = 9.4, 6.8, 1.8 Hz, 4H), 7.23 (dd, *J* = 5.5, 3.2 Hz, 4H), 7.13 (dd, *J* =

8.4, 1.7 Hz, 2H), 6.97 (t, J = 2.1 Hz, 1H), 6.90 (s, 4H), 6.59 (d, J = 2.1 Hz, 2H), 2.74 (t, J = 7.8 Hz, 4H), 2.29 (s, 6H), 2.06 (s, 12H), 1.70 – 1.65 (m, 4H), 1.40 – 1.30 (m, 12H), 0.92 – 0.86 (m, 6H).

^{13}C NMR (151 MHz, $CDCl_3$) δ 147.99, 143.34, 139.96, 139.75, 139.03, 137.37, 137.11, 134.63, 134.32, 130.19, 129.29, 128.86, 127.63, 126.83, 126.42, 126.28, 123.78, 123.44, 120.96, 119.49, 115.08, 110.65, 109.79, 109.50, 36.11, 32.40, 31.96, 29.20, 22.79, 21.15, 18.63, 14.28.

HRMS (MALDI-TOF) m/z: $[M]^+$ Calculated for $C_{68}H_{71}N_3$ 929.5643; Found 929.5669 (2.8 ppm).

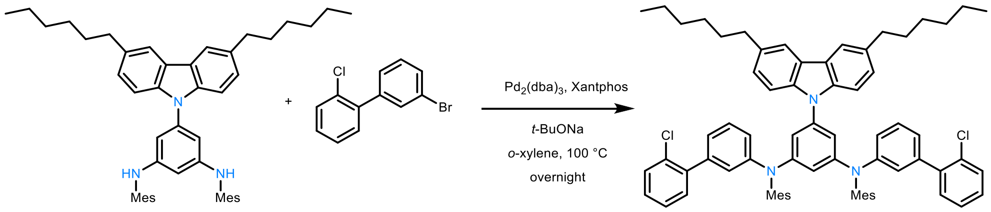

S11 **S20**

Compound **S11** (3.0 g, 4.42 mmol, 1.0 equiv.), $Pd_2(dba)_3$ (202 mg, 5 mol%), Xantphos (256 mg, 10 mol%) and 3'-bromo-2-chloro-1,1'-biphenyl (3.55 g, 13.3 mmol, 3.0 equiv.) were added to a 500 mL flask. After vacuumed and backfilled with nitrogen, *o*-xylene (300 mL) was added. The mixture stirred at rt for 20 min. Under a nitrogen flow, *t*-BuONa (1.70 g, 17.7 mmol, 4.0 equiv.) was finally added. The reaction mixture stirred at 100 °C overnight. After cooling to rt, the mixture was passed through a pad of celite, rinsed with DCM. After removal of the solvent, the residue was purified by column chromatography using hexane/DCM (v/v = 6:1) as the eluents to give compound **S20** (2.63 g, 56%) as white solid.

^{1}H NMR (600 MHz, $CDCl_3$) δ 7.80 (d, J = 1.6 Hz, 2H), 7.43 (dd, J = 7.4, 1.3 Hz, 2H), 7.32 – 7.25 (m, 6H), 7.16 – 6.94 (m, 10H), 6.90 – 6.86 (m, 2H), 6.84 (s, 4H), 6.53 (d, J = 2.0 Hz, 1H), 2.74 (t, J = 7.7 Hz, 4H), 2.26 (s, 6H), 2.04 (s, 12H), 1.68 (p, J = 7.4 Hz, 4H), 1.43 – 1.27 (m, 12H), 0.94 – 0.84 (m, 6H).

^{13}C NMR (151 MHz, $CDCl_3$) δ 147.76, 145.44, 140.76, 140.27, 139.83, 139.67, 139.07, 137.32, 136.94, 134.19, 132.77, 131.42, 130.10, 130.05, 128.85, 128.52, 126.83, 126.36, 123.37, 122.11, 120.73, 119.41, 118.75, 110.40, 109.78, 109.01, 36.11, 32.40, 31.98, 29.20, 22.80, 21.13, 18.57, 18.55, 14.29.

HRMS (MALDI-TOF) m/z: $[M]^+$ Calculated for $C_{72}H_{73}Cl_2N_3$ 1049.5176; Found 1049.5139 (-3.5 ppm).

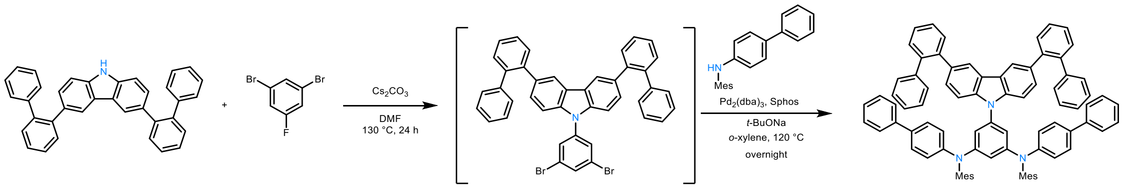

S21

3,6-Di([1,1'-biphenyl]-2-yl)-9*H*-carbazole (800 mg, 1.70 mmol, 1.0 equiv.) was dissolved in DMF (10 mL). Cs_2CO_3 (1.1 g, 3.39 mmol, 2.0 equiv.) and 1,3-dibromo-5-fluorobenzene (600 mg, 2.37 mmol, 1.4 equiv.) were added at rt. The reaction mixture stirred at 135 °C for 24 hour. After cooling to rt, the reaction mixture poured into NH_4Cl (aq.) with rigorous stirring. The precipitation was collected by filtration and suspended in DCM (10 mL). MeOH (100 mL) was finally added to

form a suspension, which was further filtered and dried under vacuum to obtain the intermediate as off-white powder (1.03 g, 86%).

The intermediate (500 mg, 0.71 mmol, 1.0 equiv.), $Pd_2(dba)_3$/Sphos (50 mg/50 mg) and *N*-mesityl-[1,1'-biphenyl]-4-amine (450 mg, 1.56 mmol, 2.2 equiv.) were added to a 500 mL flask. After vacuumed and backfilled with nitrogen, *o*-xylene (300 mL) was added. The mixture stirred at rt for 20 min. Under a nitrogen flow, *t*-BuONa (540 mg, 5.67 mmol, 8.0 equiv.) was finally added. The reaction mixture stirred at 120 °C overnight. After cooling to rt, the mixture was passed through a pad of celite, rinsed with DCM. After removal of the solvent, the residue was purified by column chromatography using hexane/DCM (v/v = 3:1) as the eluents to give compound **S21** (750 mg, 94%) as white solid.

^{1}H NMR (400 MHz, $CDCl_3$) δ 8.07 (d, *J* = 7.7 Hz, 2H), 7.37 – 7.27 (m, 4H), 7.26 – 7.18 (m, 4H), 7.15 – 7.10 (m, 2H), 7.07 – 7.03 (m, 2H), 6.97 – 6.87 (m, 8H), 6.53 (t, *J* = 2.0 Hz, 1H), 6.45 (t, *J* = 2.0 Hz, 1H), 2.29 (d, *J* = 3.9 Hz, 6H), 2.05 (dd, *J* = 6.4, 2.0 Hz, 12H).

^{13}C NMR (101 MHz, $CDCl_3$) δ 147.84, 146.25, 144.86, 141.91, 141.16, 140.87, 140.73, 139.58, 139.51, 139.23, 137.42, 137.17, 136.14, 133.89, 133.47, 131.30, 130.92, 130.85, 130.09, 130.05, 129.72, 129.39, 128.82, 128.75, 128.28, 128.05, 127.80, 127.62, 127.20, 126.74, 126.68, 126.43, 126.42, 126.20, 126.16, 125.93, 123.32, 121.23, 120.01, 113.63, 110.08, 109.46, 21.19, 18.58.

HRMS (MALDI-TOF) m/z: $[M]^+$ Calculated for $C_{84}H_{67}N_3$ 1117.5330; Found 1117.5307 (-2.1 ppm).

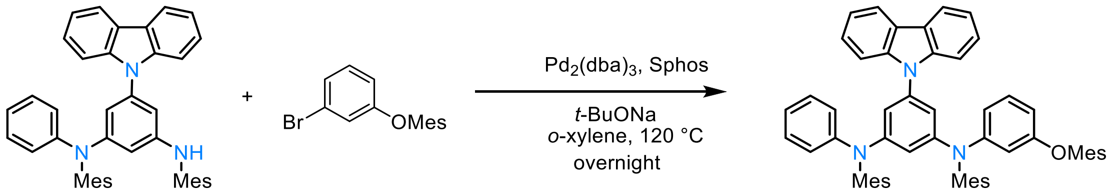

S4 **S22**

Compound **S4** (1.0 g, 1.71 mmol, 1.0 equiv.), $Pd_2(dba)_3$ (60 mg), Sphos (60 mg) and 2-(3-bromophenoxy)-1,3,5-trimethylbenzene (745 mg, 2.6 mmol, 1.5 equiv.) were added to a 500 mL flask. After vacuumed and backfilled with nitrogen, *o*-xylene (100 mL) was added. The mixture stirred at rt for 20 min. Under a nitrogen flow, *t*-BuONa (490 mg, 5.1 mmol, 3.0 equiv.) was finally added. The reaction mixture stirred at 120 °C overnight. After cooling to rt, the mixture was passed through a pad of celite, rinsed with DCM. After removal of the solvent, the residue was purified by column chromatography using hexane/DCM (v/v = 3:1) as the eluents to give compound **S22** (1.2 g, 88%) as white solid.

^{1}H NMR (400 MHz, $CDCl_3$) δ 8.08 (d, *J* = 7.7 Hz, 2H), 7.34 (dt, *J* = 14.5, 8.0 Hz, 4H), 7.26 – 7.20 (m, 4H), 7.11 – 6.93 (m, 5H), 6.92 – 6.76 (m, 8H), 6.60 – 6.47 (m, 3H), 6.04 (dd, *J* = 8.2, 2.4 Hz, 1H), 2.32 – 2.25 (m, 9H), 2.11 – 1.99 (18H).

^{13}C NMR (101 MHz, $CDCl_3$) δ 159.11, 149.10, 148.05, 147.69, 147.10, 145.60, 140.47, 139.89, 139.83, 139.09, 137.40, 137.25, 137.01, 136.99, 134.30, 131.11, 130.07, 130.03, 129.83, 129.52, 129.24, 125.84, 123.31, 121.15, 120.23, 119.81, 119.73, 112.54, 110.42, 110.35, 110.17, 109.64, 106.82, 106.38, 21.12, 20.89, 18.55, 18.44, 16.37.

HRMS (MALDI-TOF) m/z: $[M]^+$ Calculated for $C_{57}H_{53}N_3O$ 795.4183; Found 795.4142 (-5.1 ppm).

11. NMR spectrum

^{1}H NMR spectrum (500 MHz, $CDCl_3$, 298 K) of compound **1**

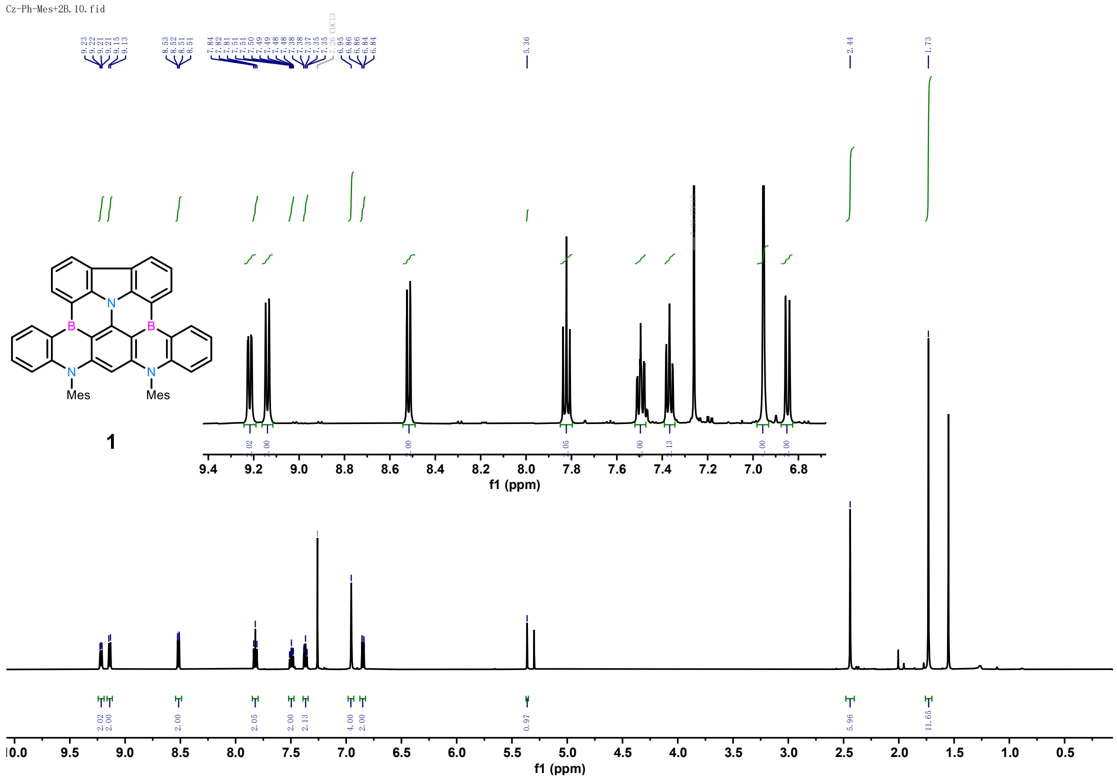

^{13}C NMR spectrum (101 MHz, $CDCl_3$, 298 K) of compound **1**

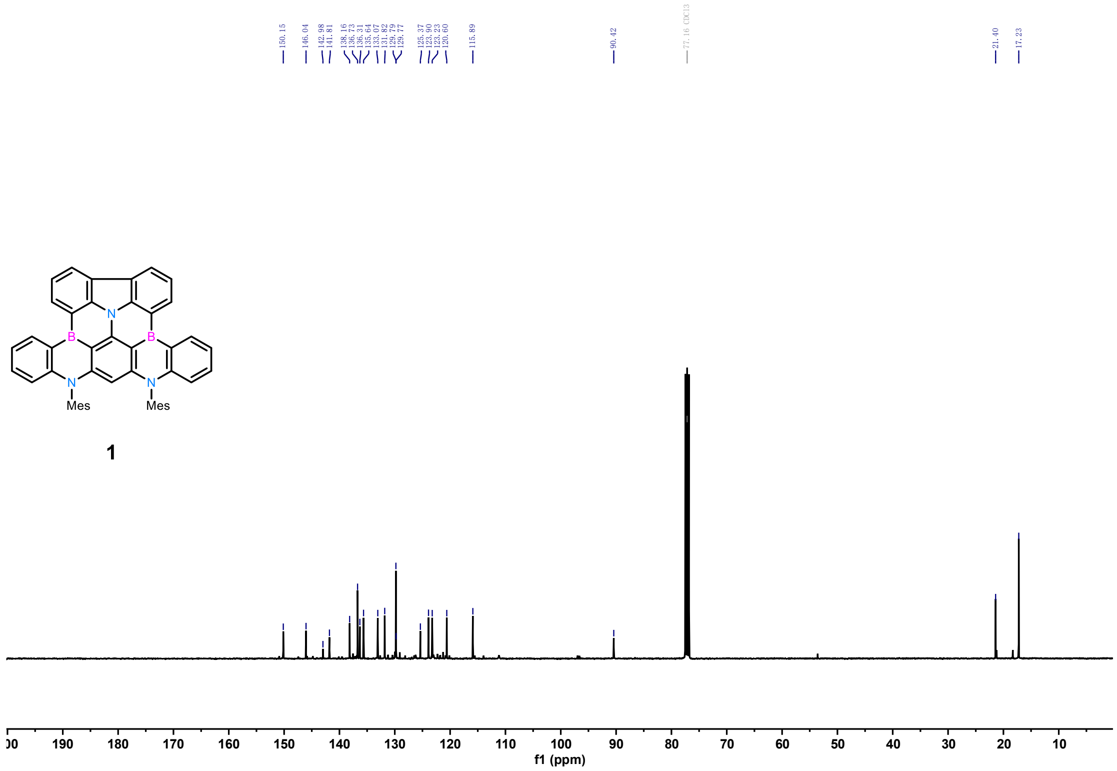

^{1}H NMR spectrum (600 MHz, $CDCl_3$, 298 K) of compound **2**

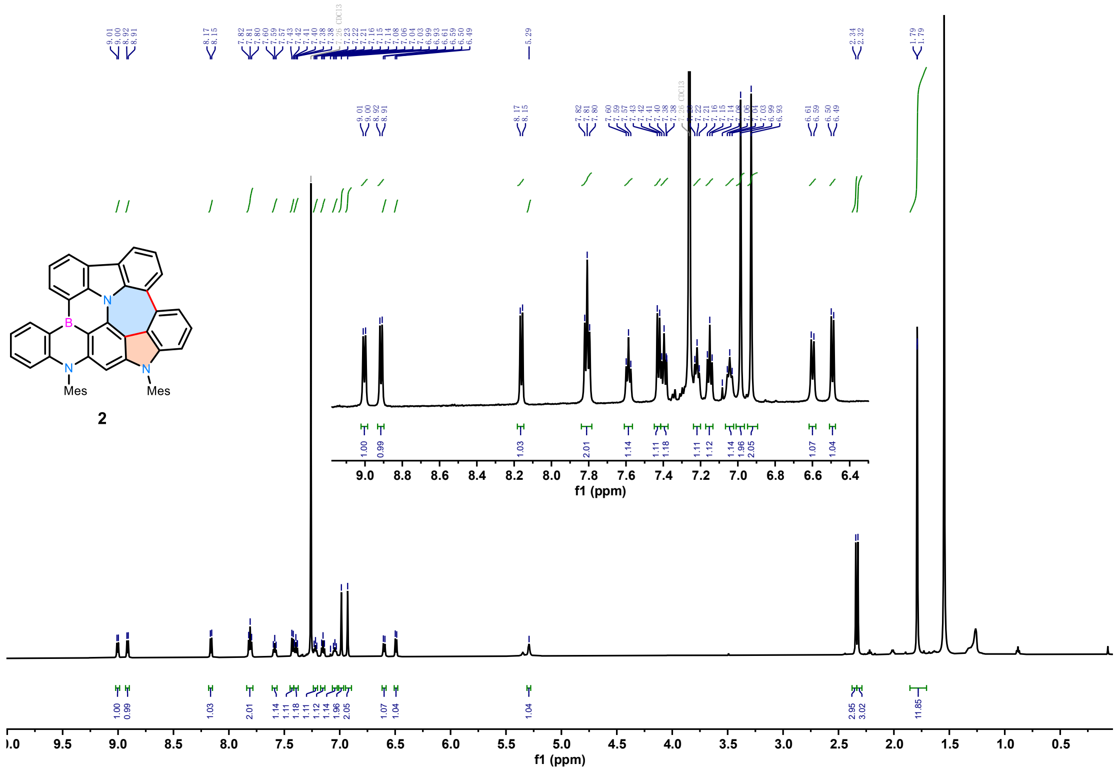

^{13}C NMR spectrum (151 MHz, Acetone-*d6*/CS_2, 298 K) of compound **2**

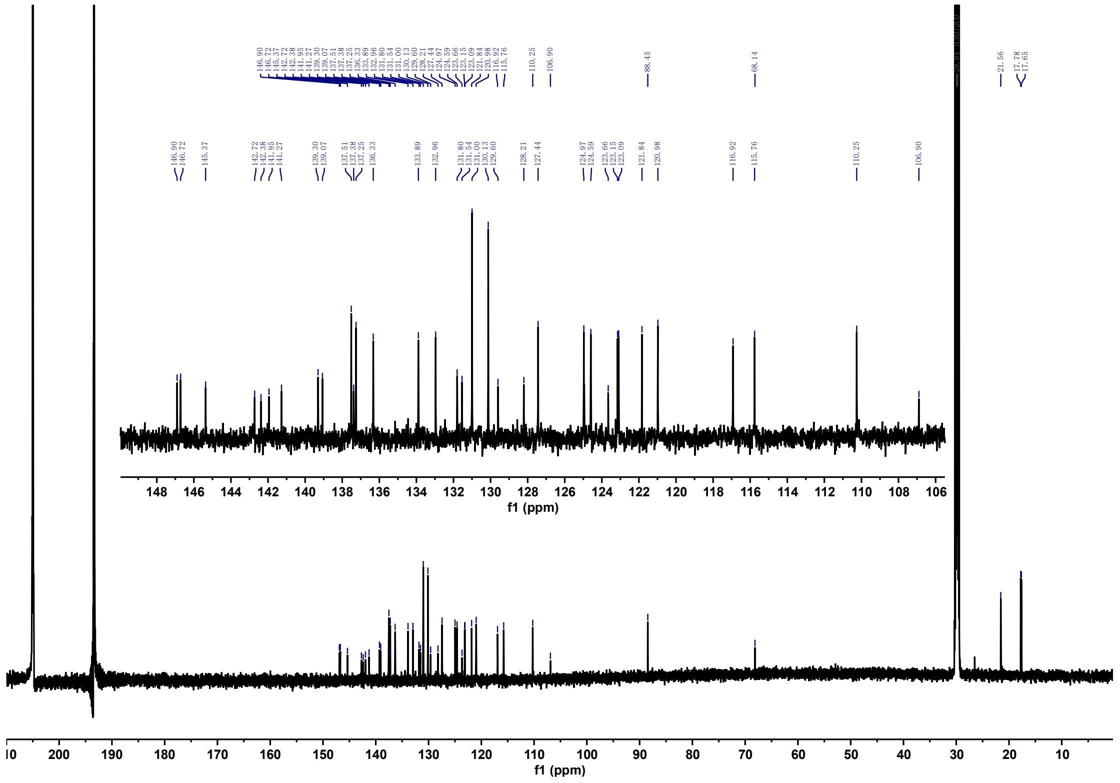

^{1}H NMR spectrum (400 MHz, $CDCl_3$, 298 K) of compound **3a**

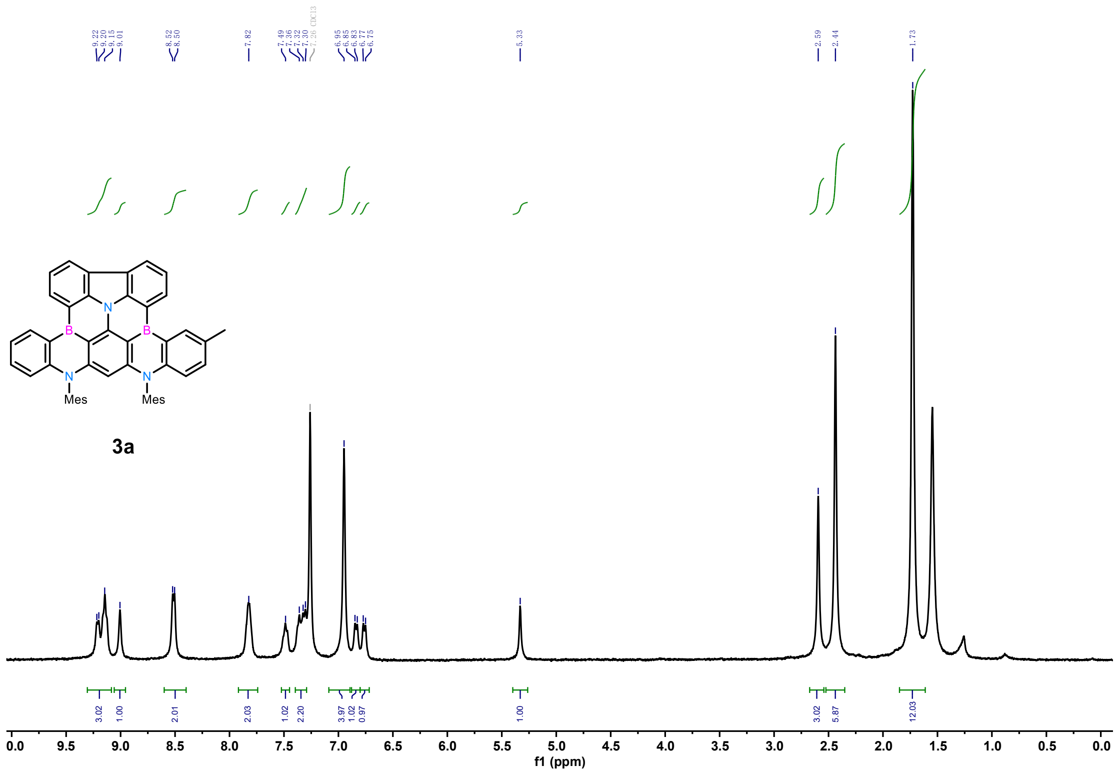

^{13}C NMR spectrum (101 MHz, $CDCl_3$, 298 K) of compound **3a**

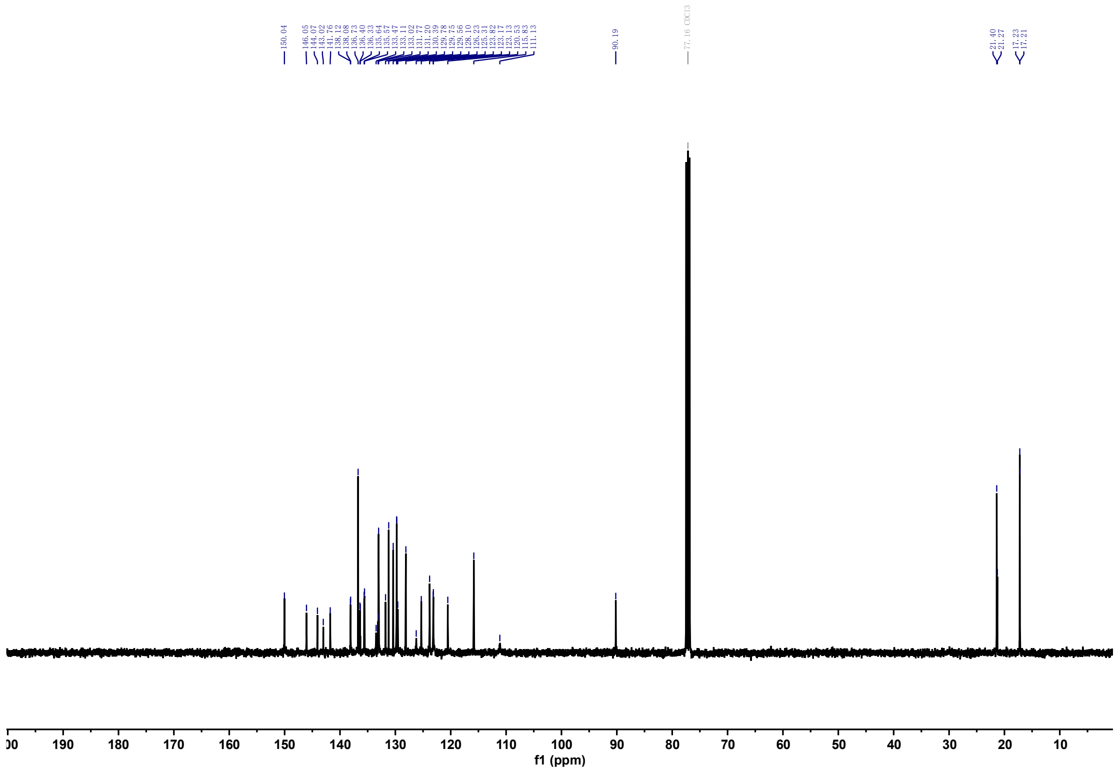

^{1}H NMR spectrum (400 MHz, $CDCl_3$, 298 K) of compound **3b**

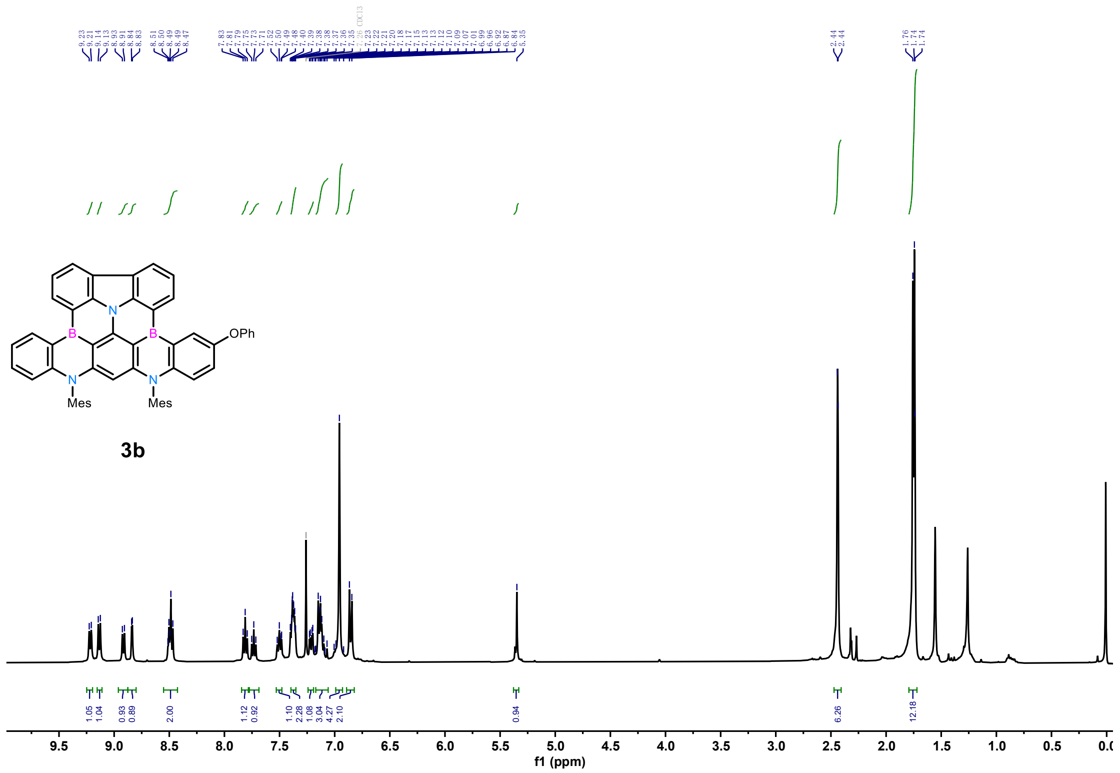

^{13}C NMR spectrum (101 MHz, $CDCl_3$, 298 K) of compound **3b**

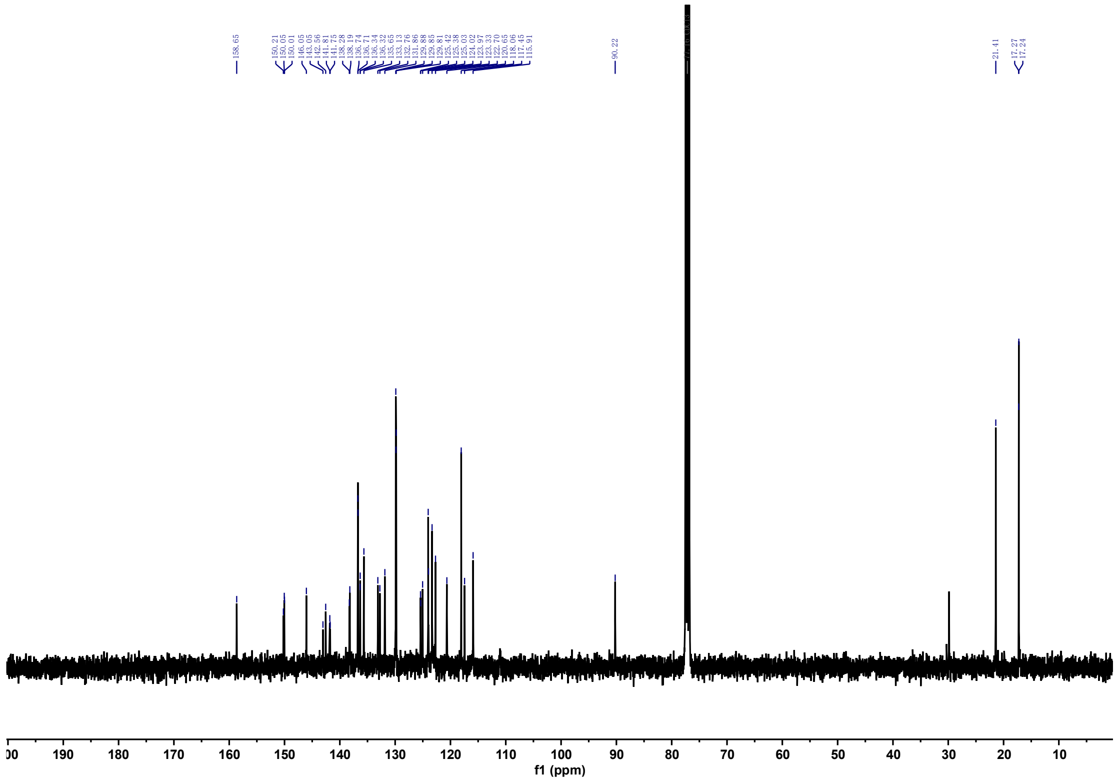

^{1}H NMR spectrum (400 MHz, $CDCl_3$, 298 K) of compound **3b’**

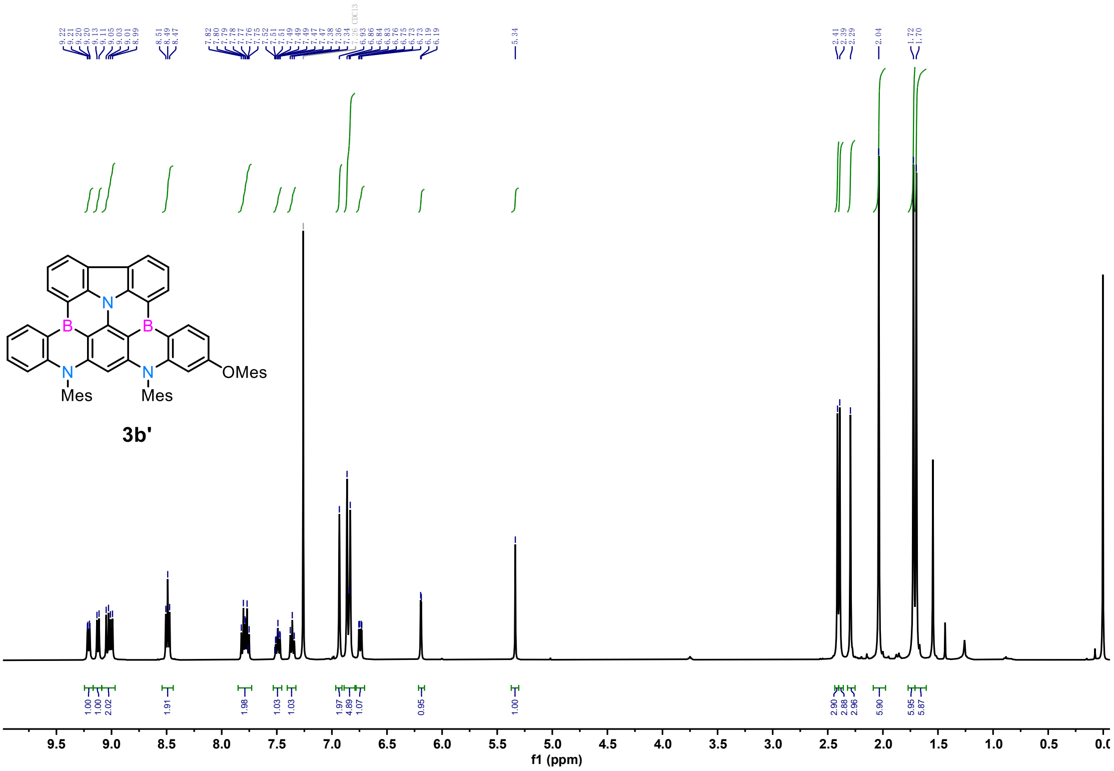

^{13}C NMR spectrum (101 MHz, $CDCl_3$, 298 K) of compound **3b’**

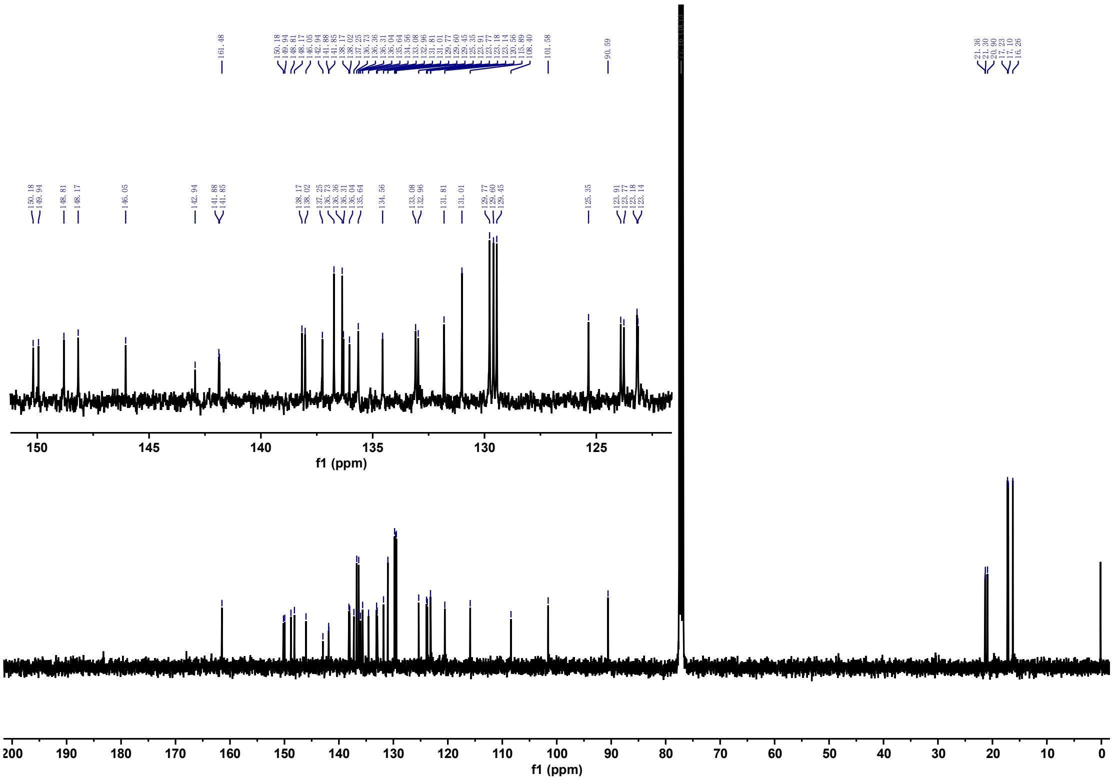

^{1}H NMR spectrum (400 MHz, $CDCl_3$, 298 K) of compound **3c**

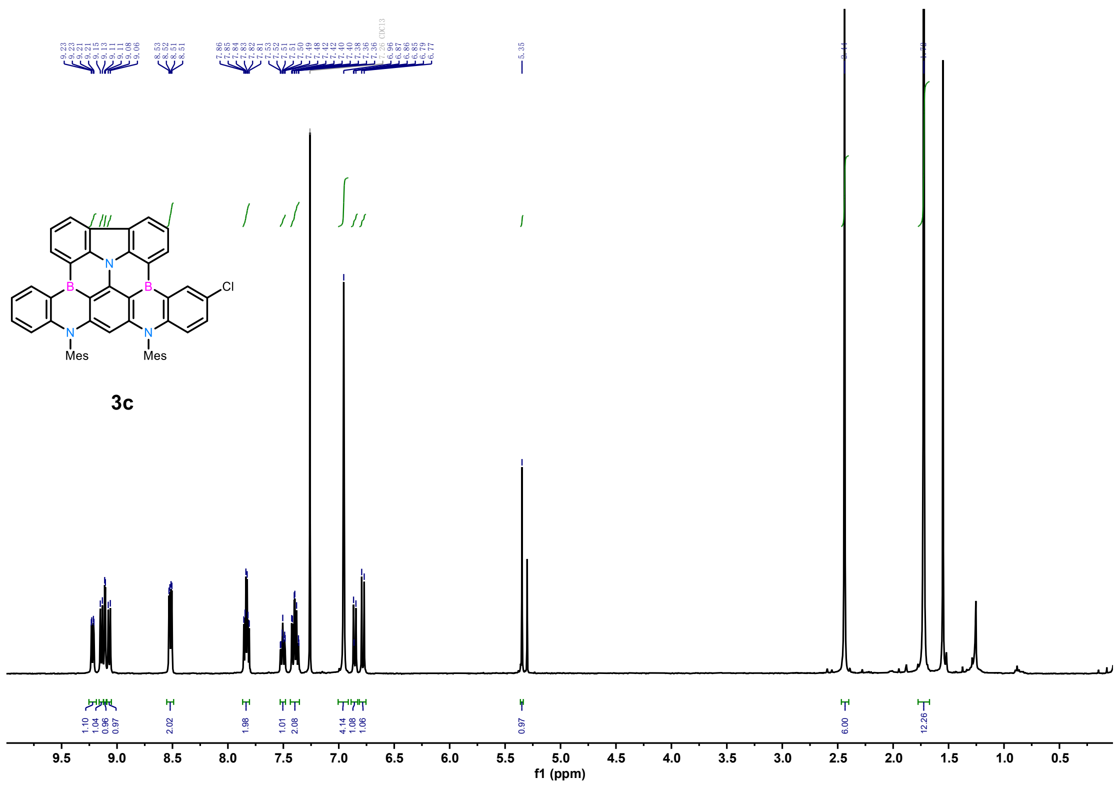

^{13}C NMR spectrum (101 MHz, $CDCl_3$, 298 K) of compound **3c**

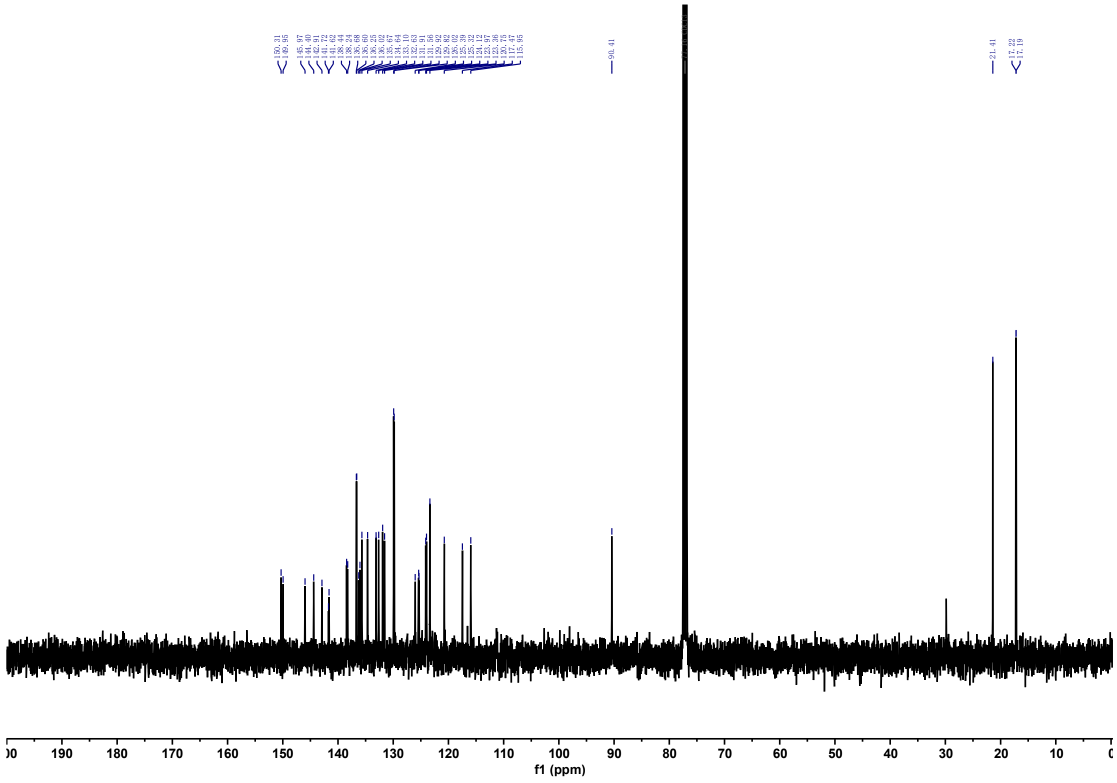

^1H NMR spectrum (500 MHz, $CDCl_3$, 298 K) of compound **3d**

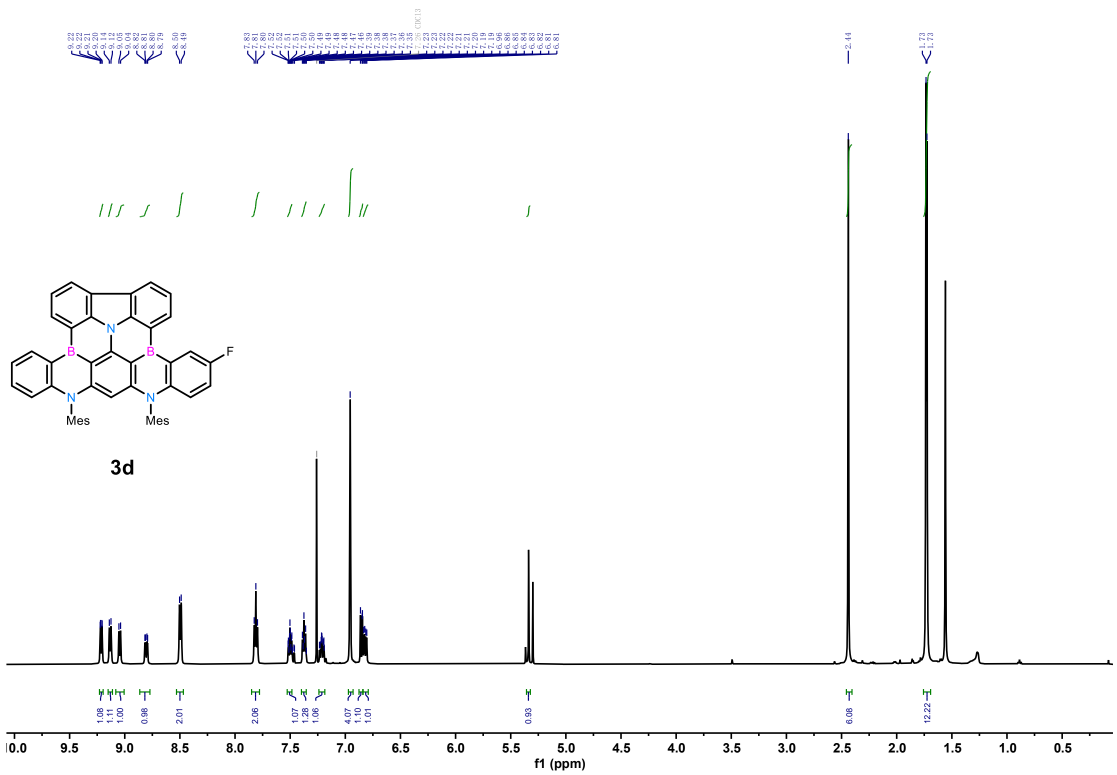

^{13}C NMR spectrum (101 MHz, $CDCl_3$, 298 K) of compound **3d**

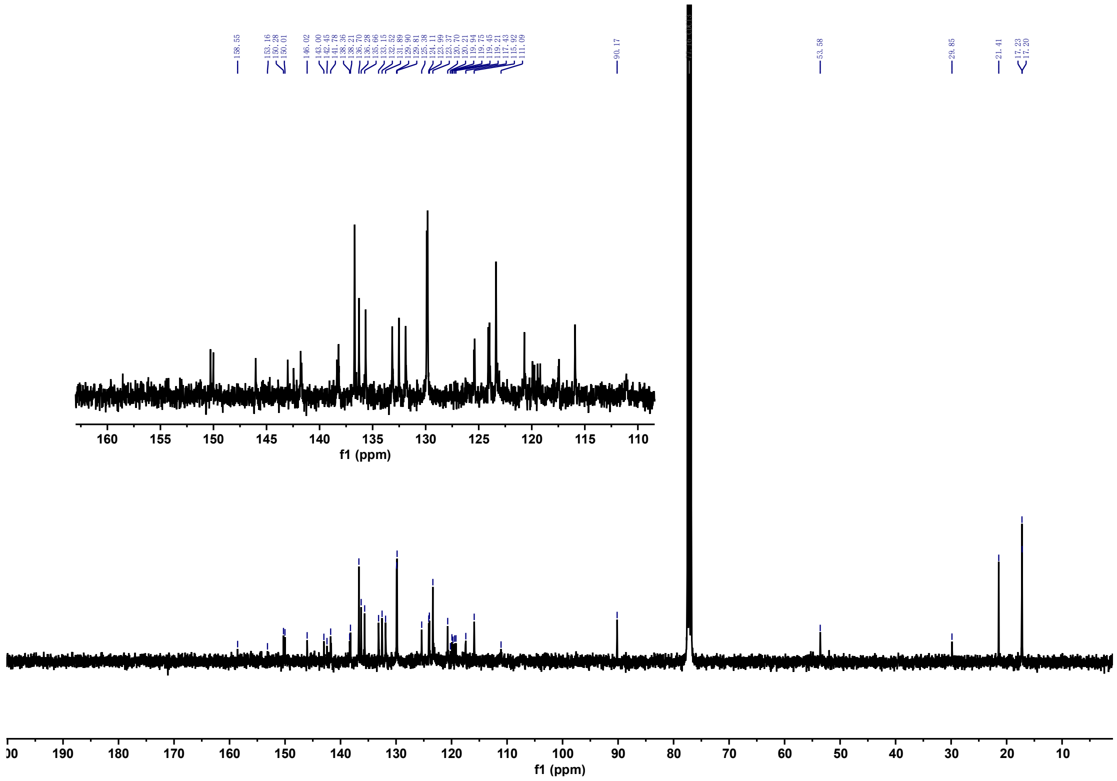

^{1}H NMR spectrum (400 MHz, $CDCl_3$, 298 K) of compound **3e**

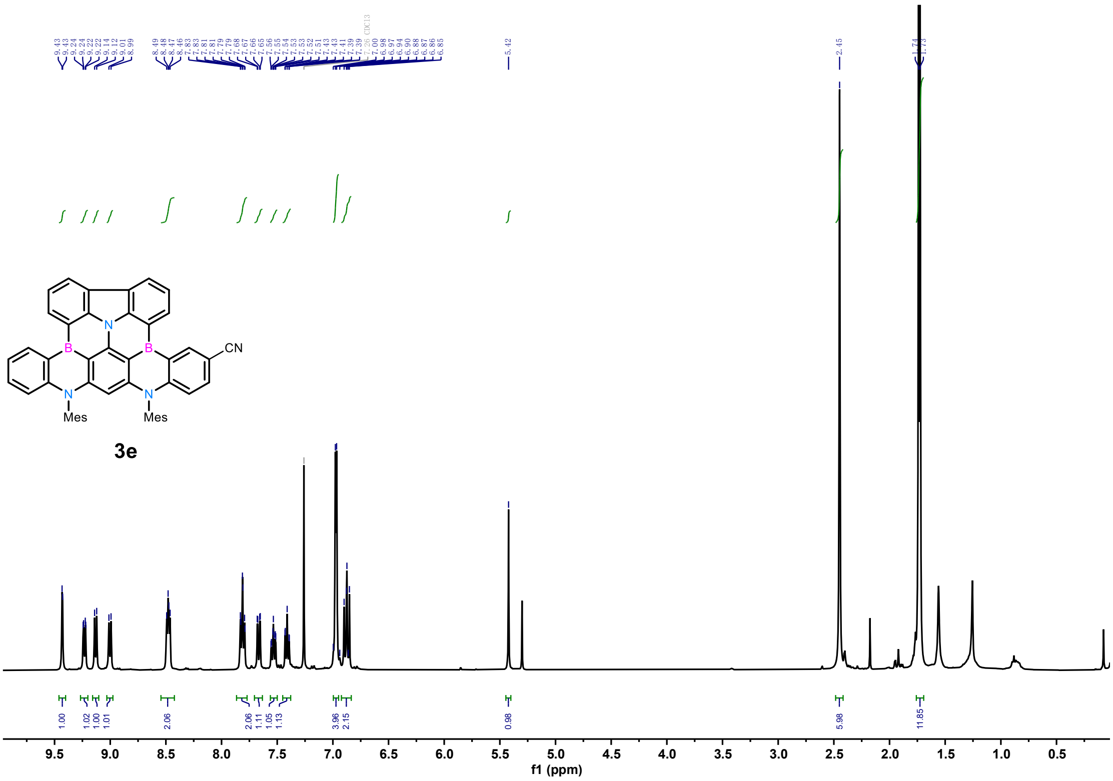

^{13}C NMR spectrum (101 MHz, $CDCl_3$, 298 K) of compound **3e**

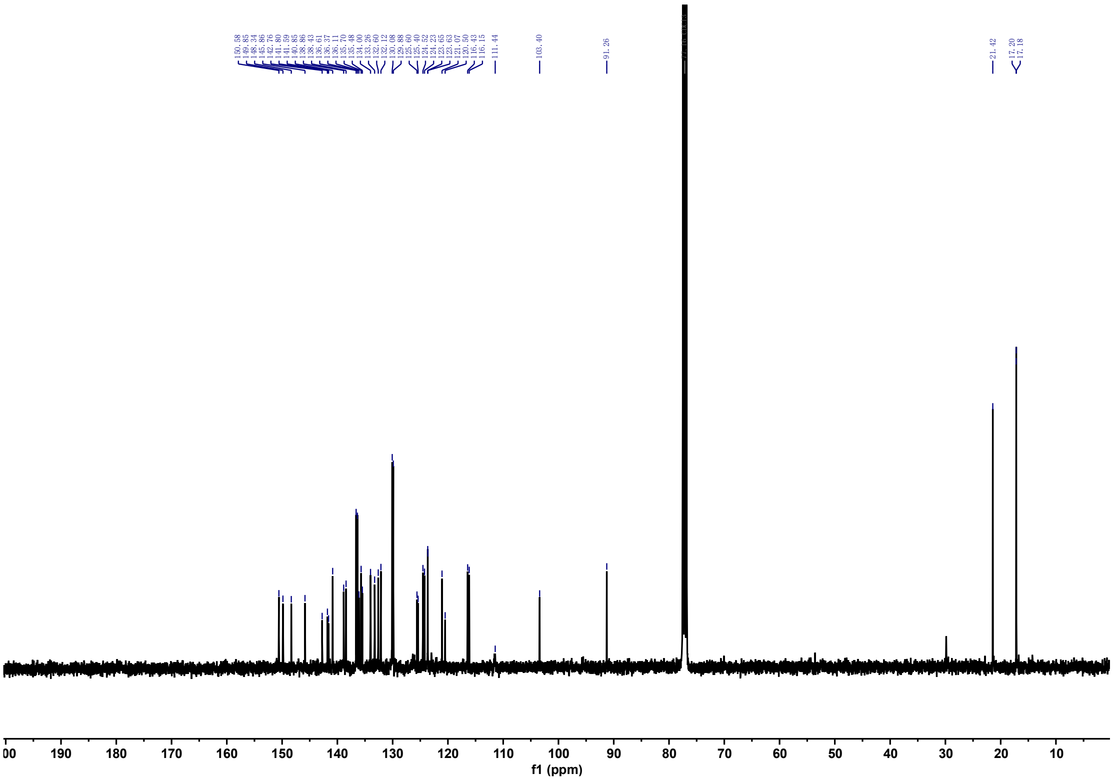

^{1}H NMR spectrum (600 MHz, $CDCl_3$, 298 K) of compound **4b'**

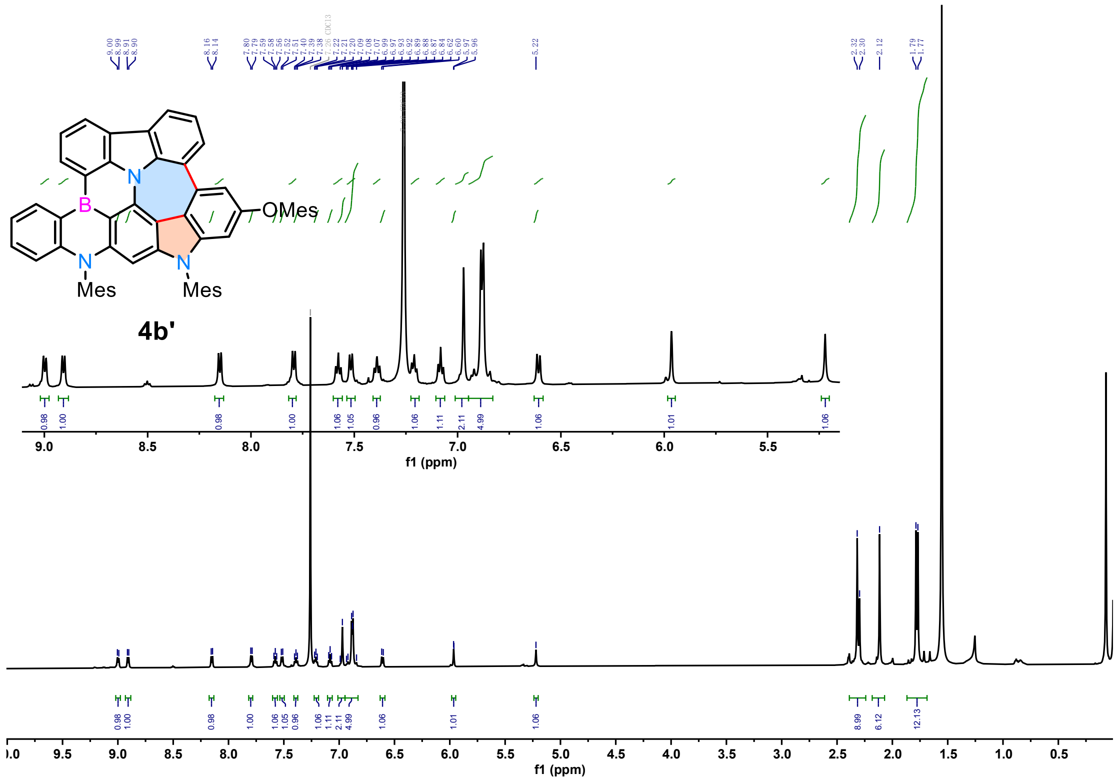

^{13}C NMR spectrum (101 MHz, $CDCl_3$, 298 K) of compound **4b'**

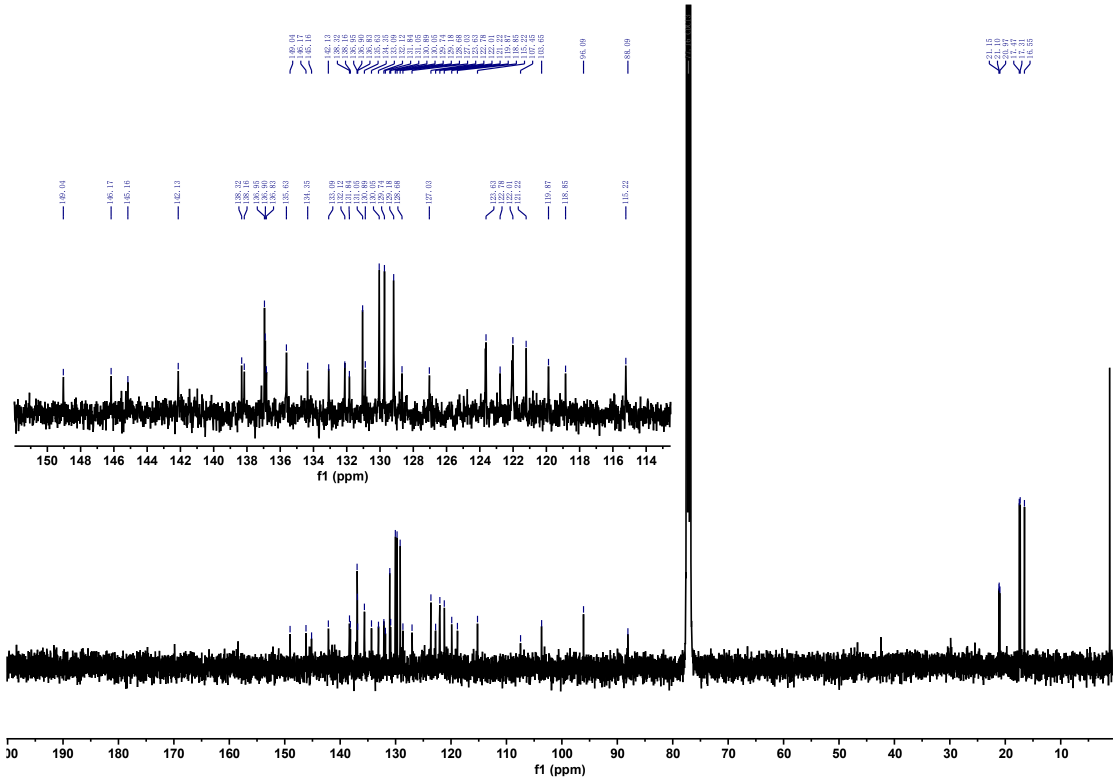

^{1}H NMR spectrum (600 MHz, $CDCl_3$, 298 K) of compound **5b**

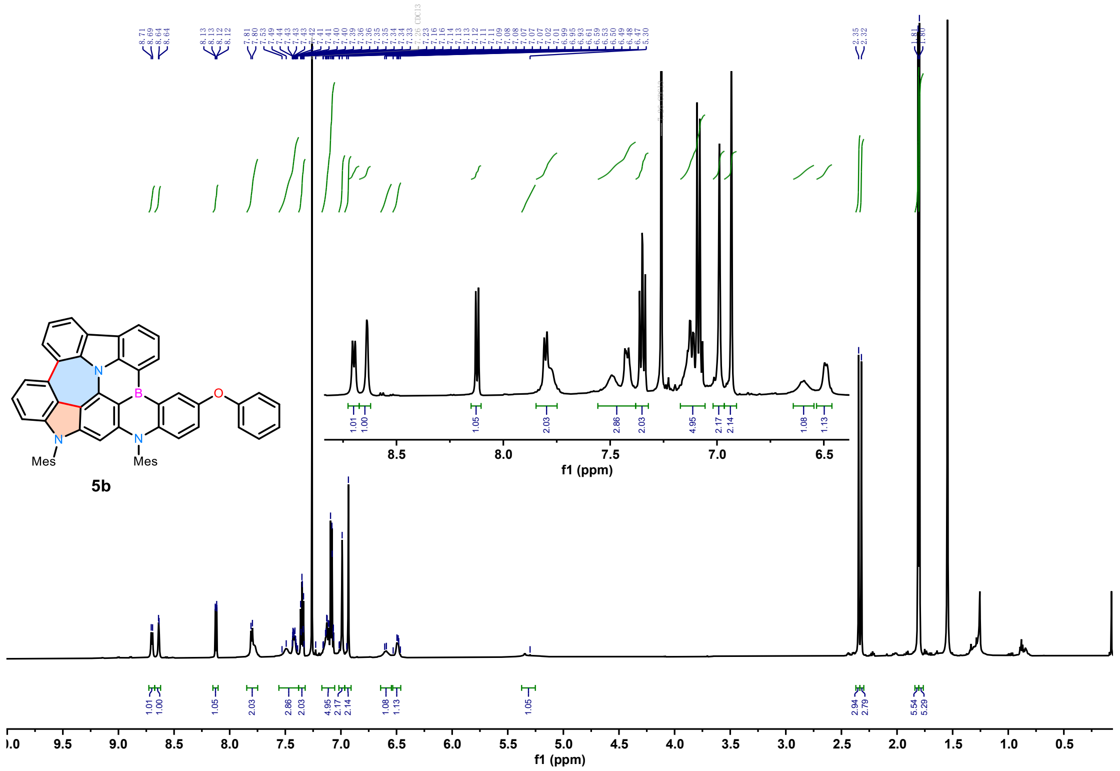

^{13}C NMR spectrum (151 MHz, Acetone-*d6*/CS_2, 298 K) of compound **5b**

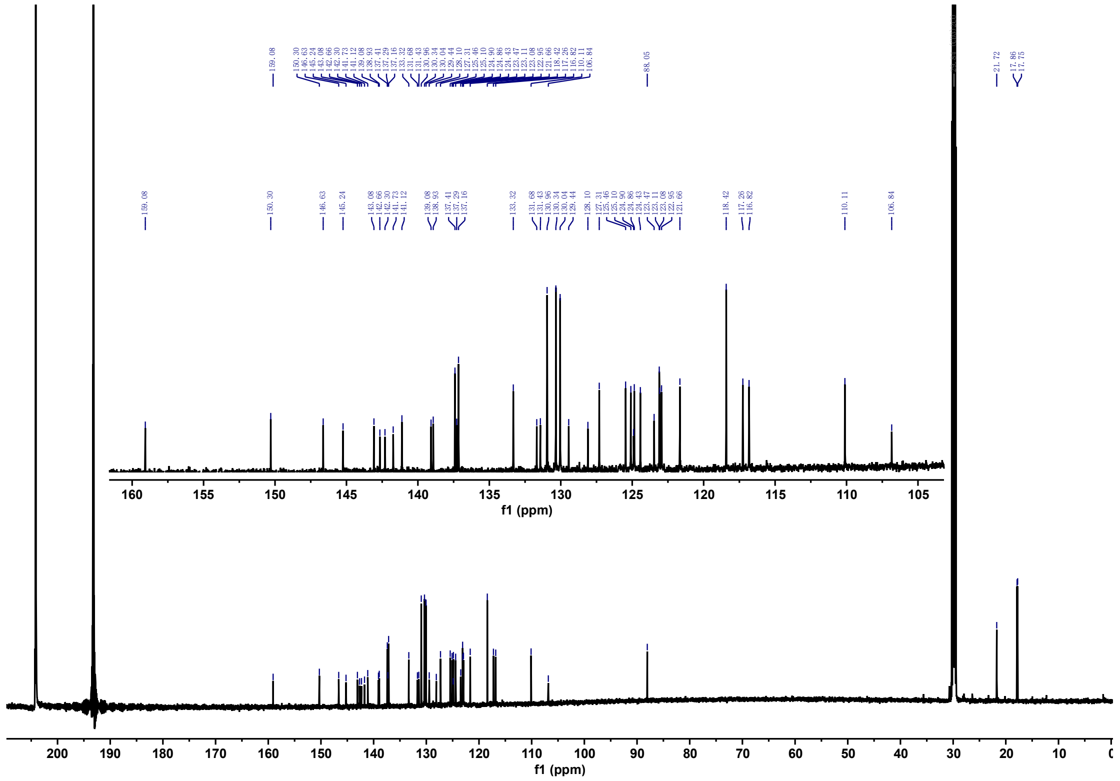

^{1}H NMR spectrum (400 MHz, $CDCl_3$, 298 K) of compound **5c**

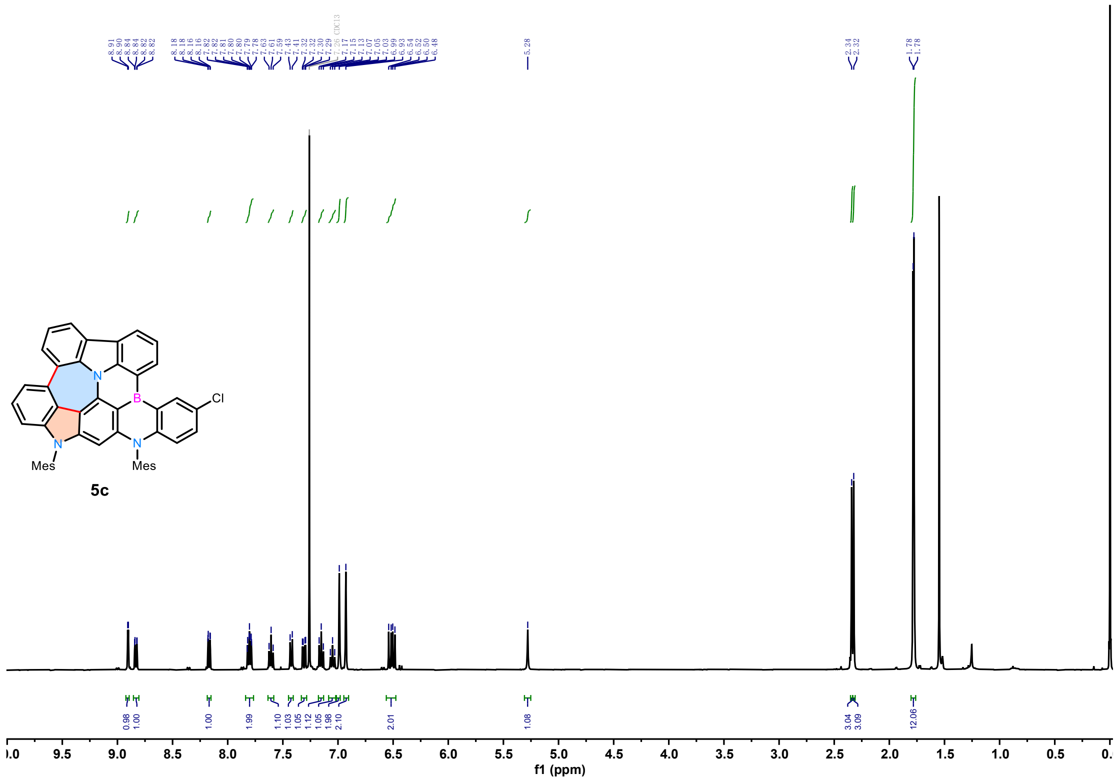

^{13}C NMR spectrum (101 MHz, $CDCl_3$, 298 K) of compound **5c**

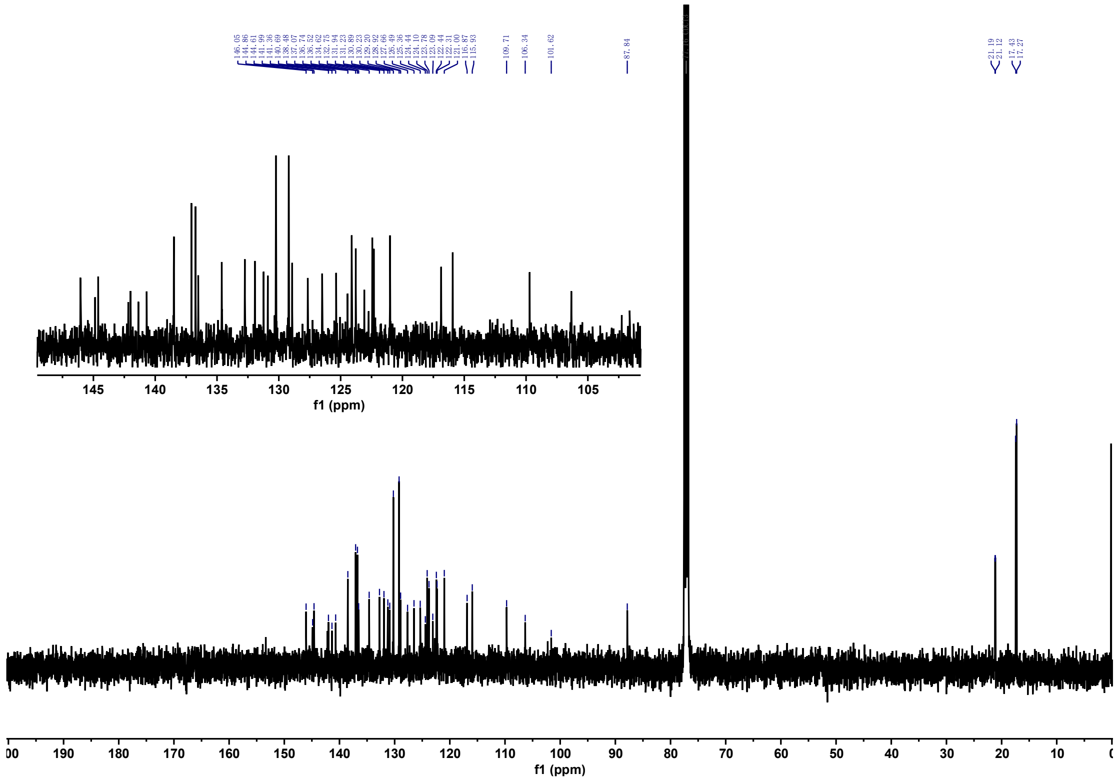

^{1}H NMR spectrum (600 MHz, Acetone-*d6*/CS_2, 298 K) of compound **5d**

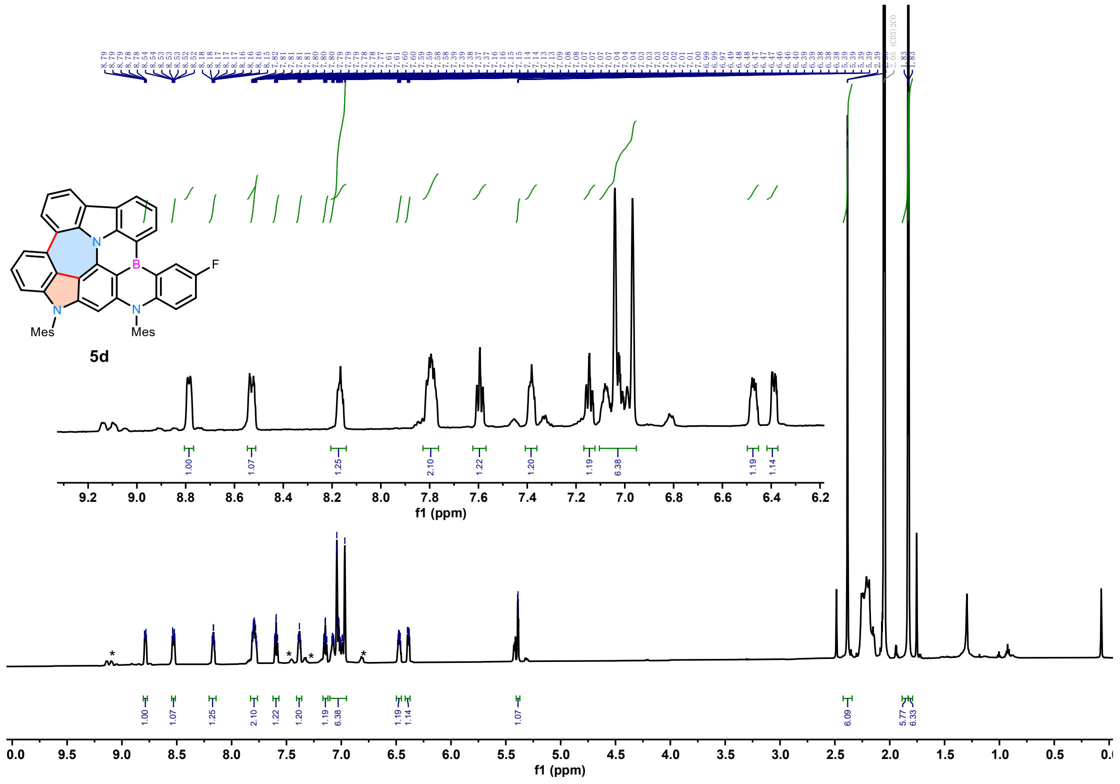

^{13}C NMR spectrum (151 MHz, Acetone-*d6*/CS_2, 298 K) of compound **5d**

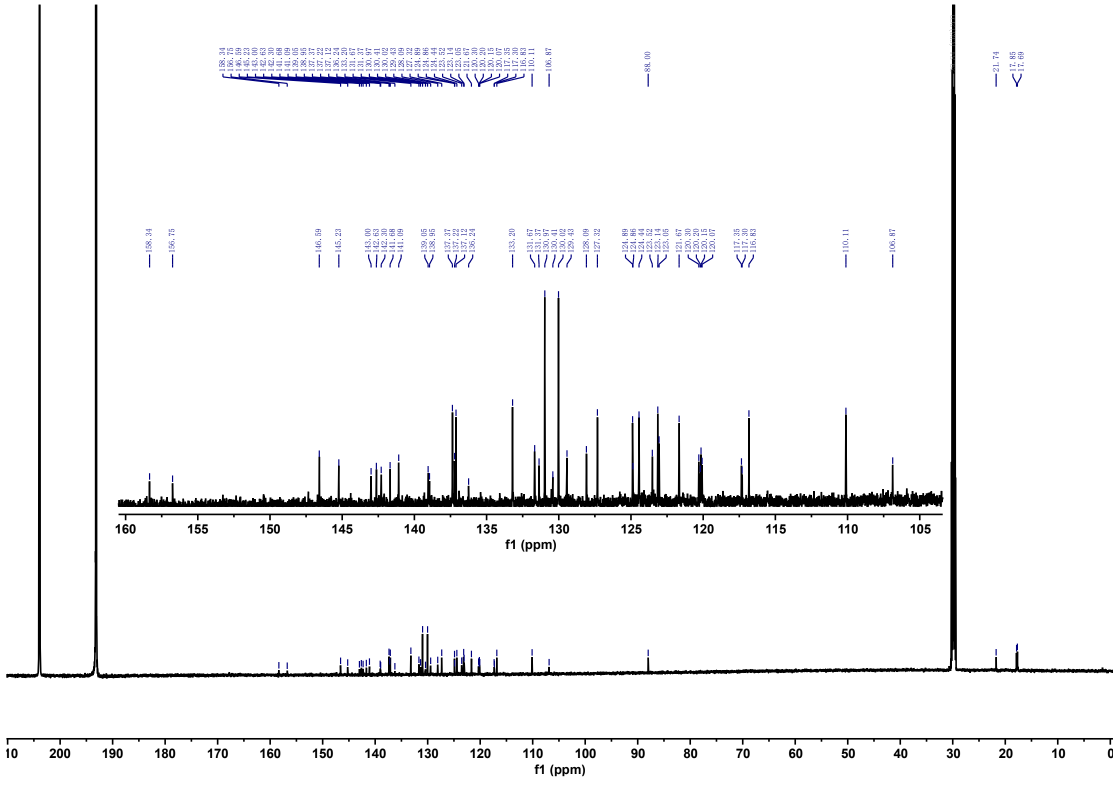

^{1}H NMR spectrum (400 MHz, $CDCl_3$, 298 K) of compound **7a**

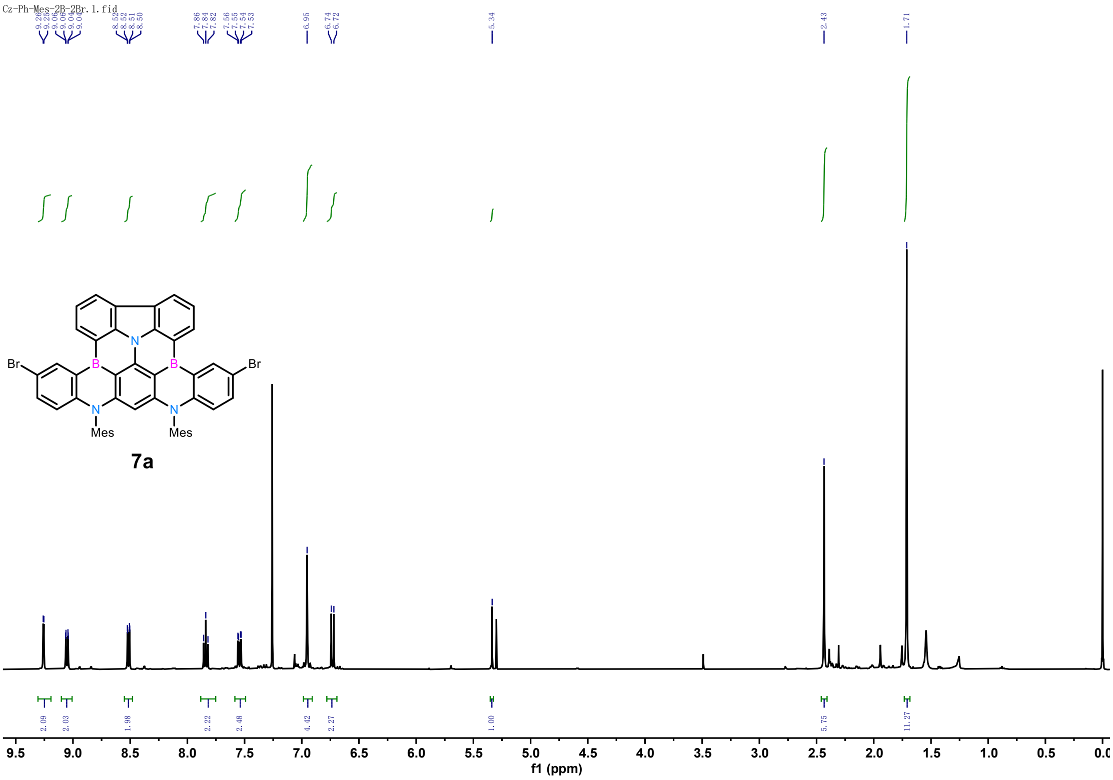

^{13}C NMR spectrum (101 MHz, $CDCl_3$, 298 K) of compound **7a**

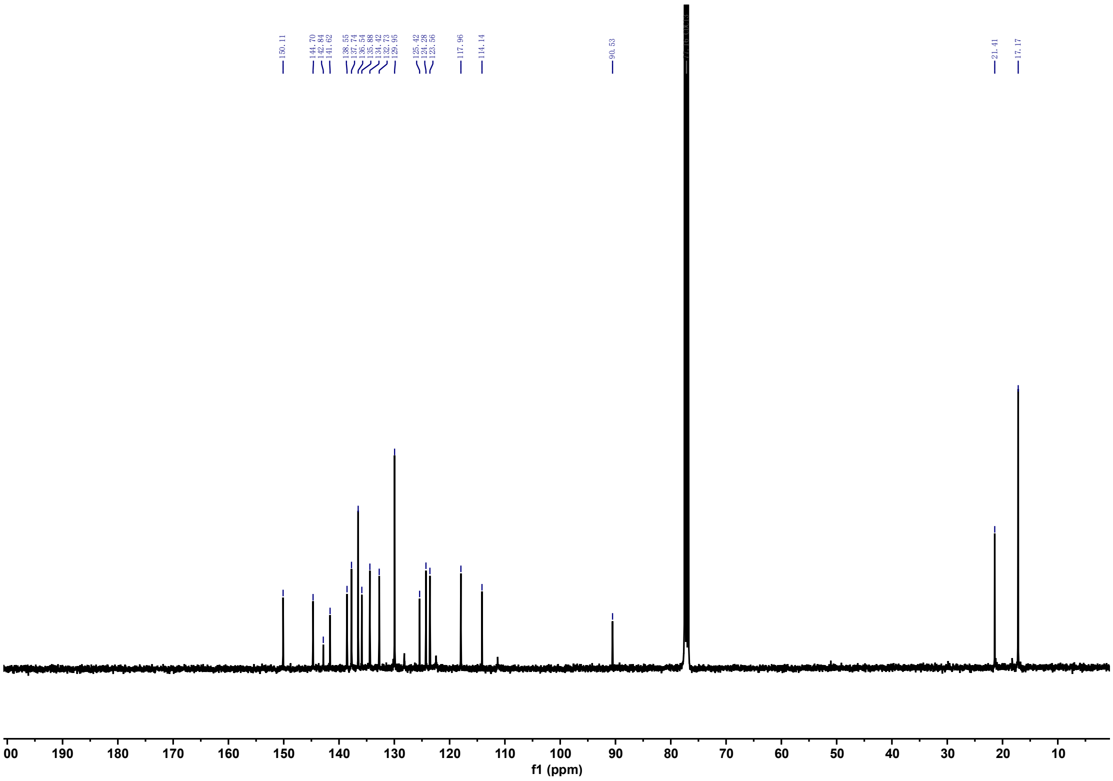

^{1}H NMR spectrum (600 MHz, $CDCl_3$, 298 K) of compound **7b**

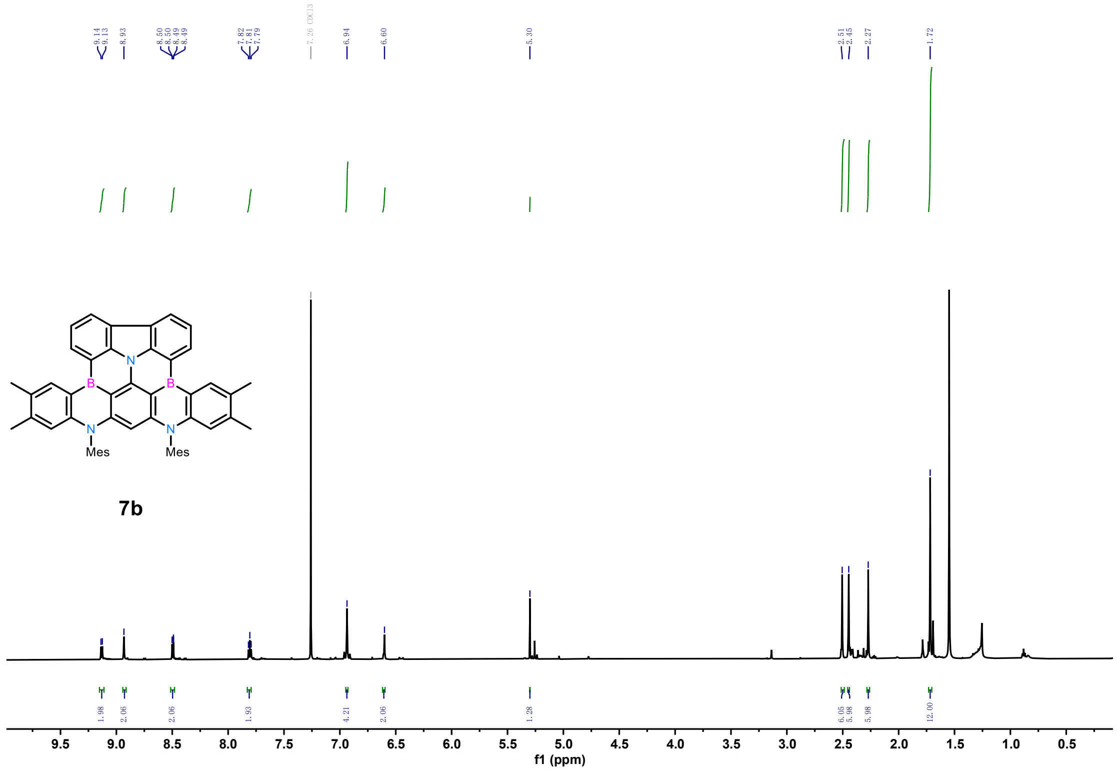

^{13}C NMR spectrum (101 MHz, $CDCl_3$, 298 K) of compound **7b**

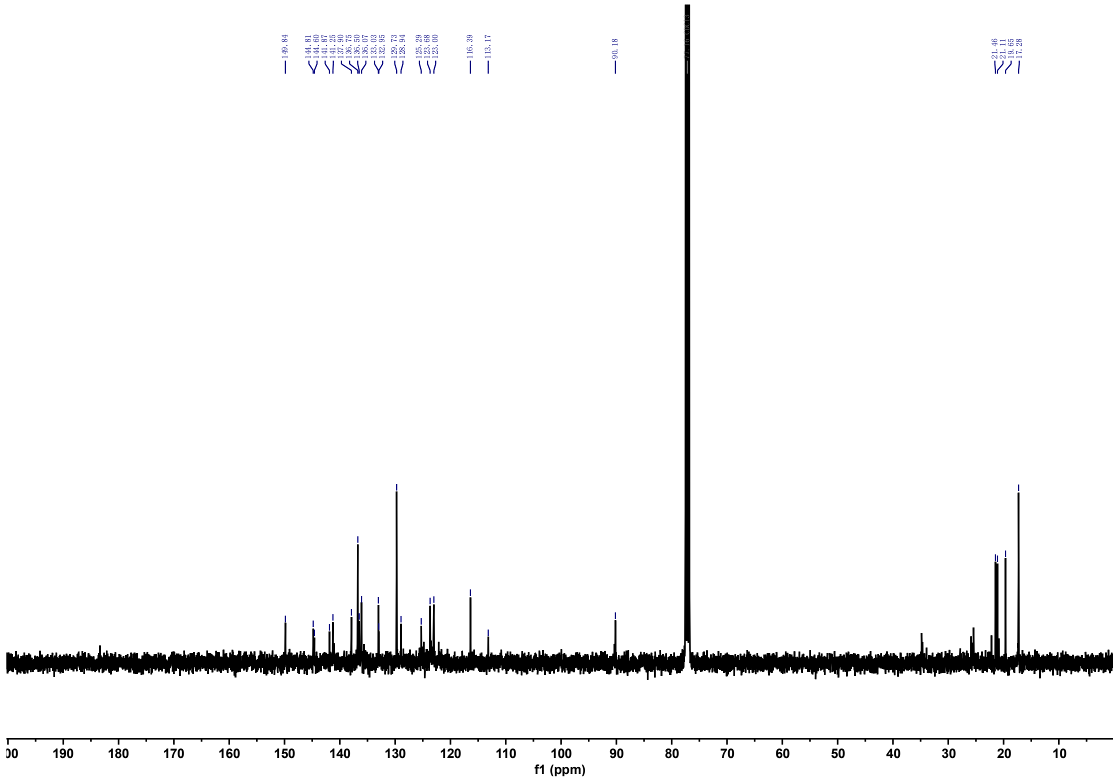

^{1}H NMR spectrum (400 MHz, $CDCl_3$, 298 K) of compound **7c**

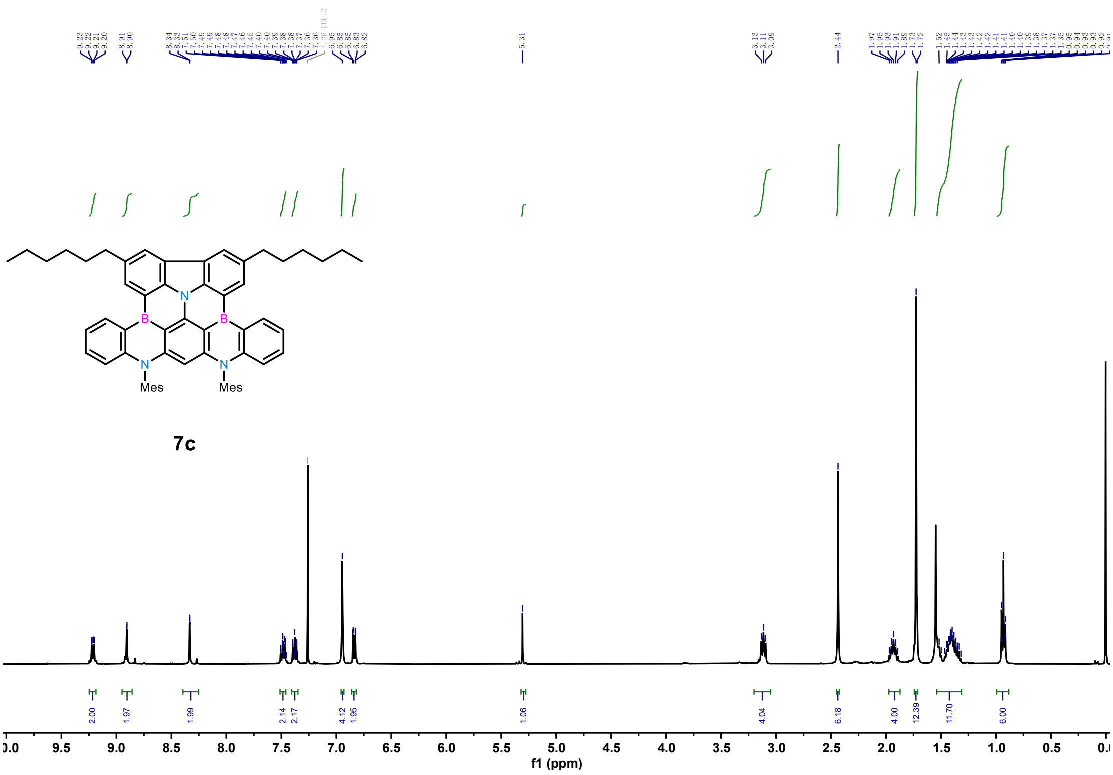

^{13}C NMR spectrum (101 MHz, $CDCl_3$, 298 K) of compound **7c**

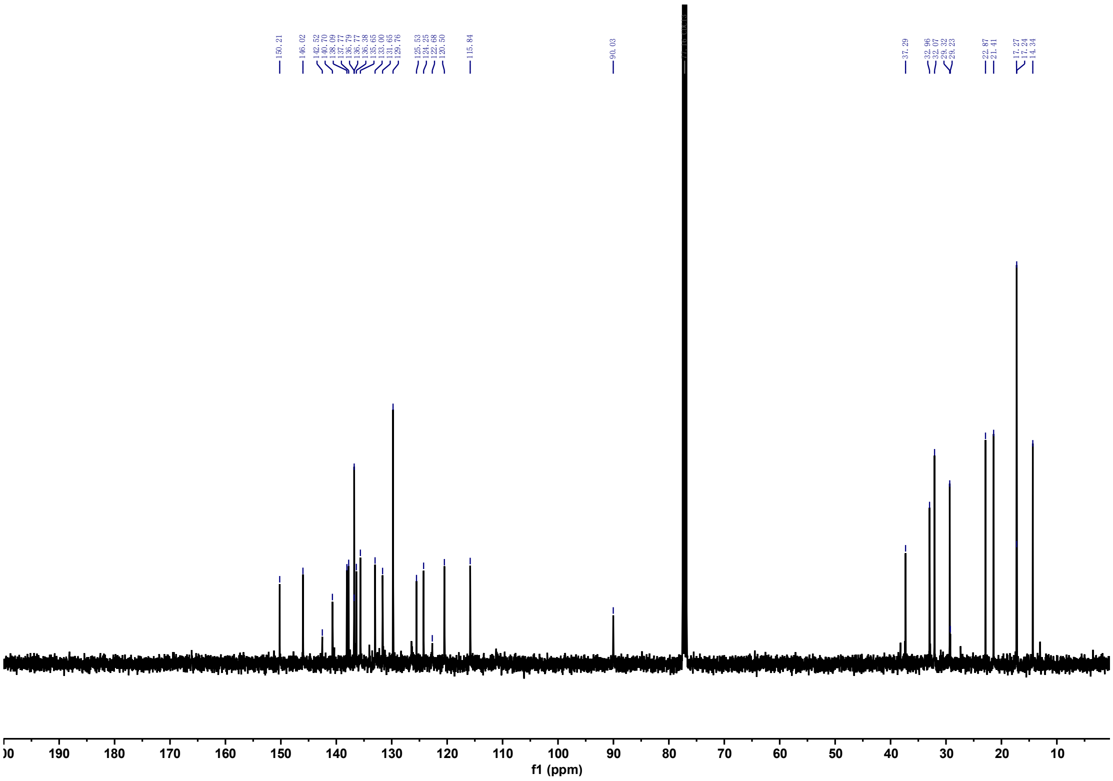

^{1}H NMR spectrum (400 MHz, $CDCl_3$, 298 K) of compound **7d**

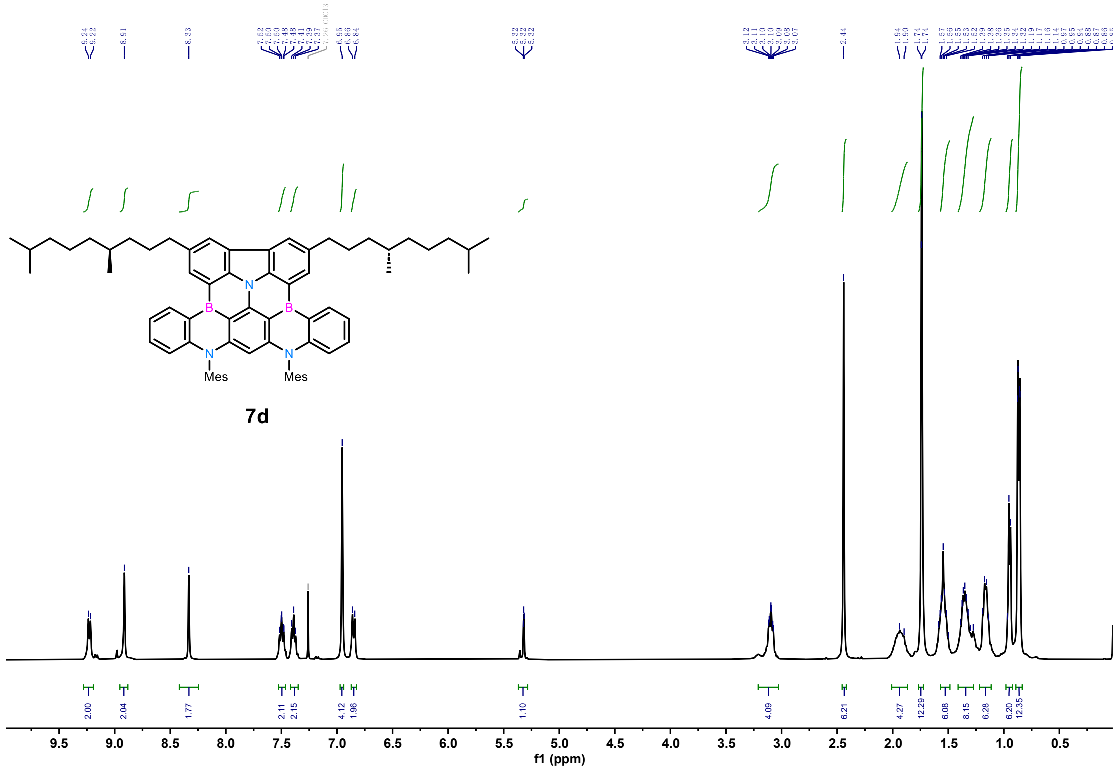

^{13}C NMR spectrum (101 MHz, $CDCl_3$, 298 K) of compound **7d**

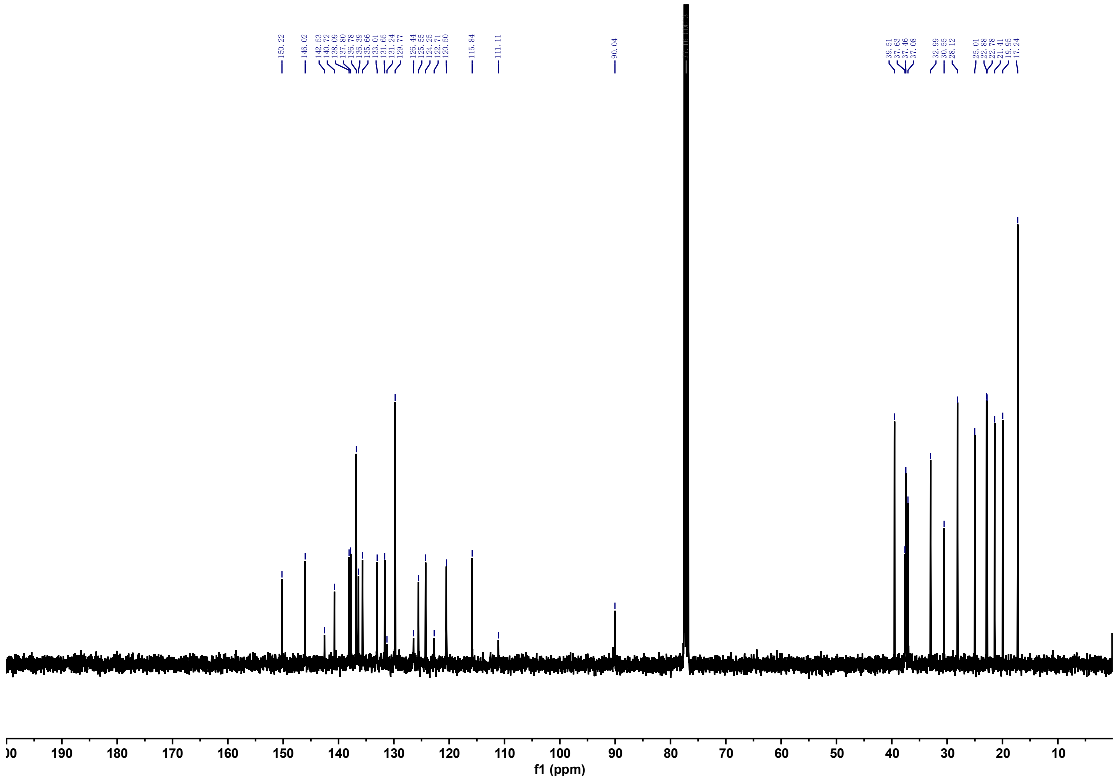

^{1}H NMR spectrum (400 MHz, $CDCl_3$, 298 K) of compound **7e**

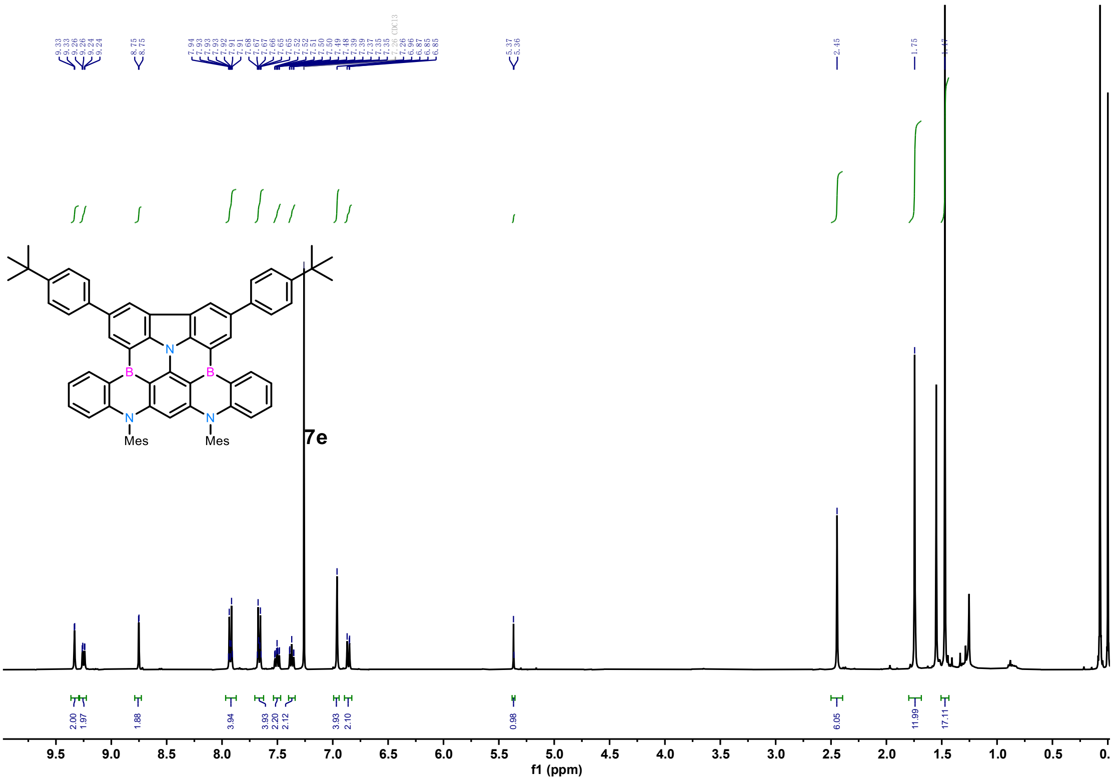

^{13}C NMR spectrum (101 MHz, $CDCl_3$, 298 K) of compound **7e**

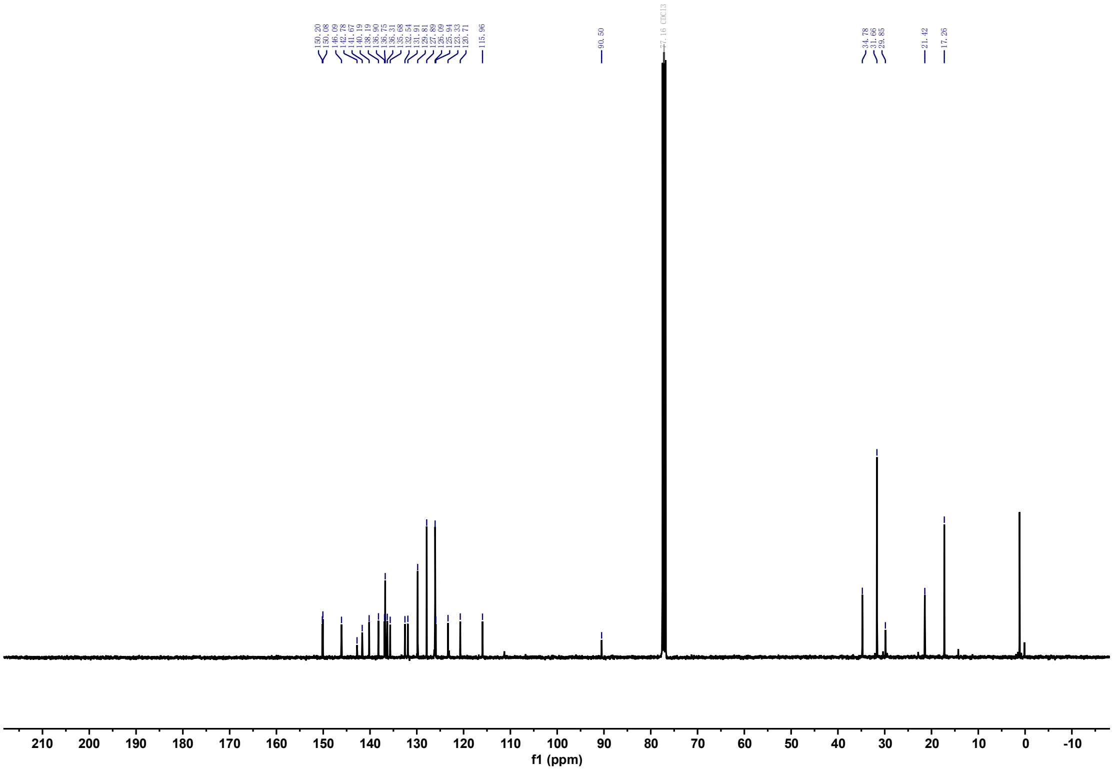

^{1}H NMR spectrum (400 MHz, $CDCl_3$, 298 K) of compound **7f**

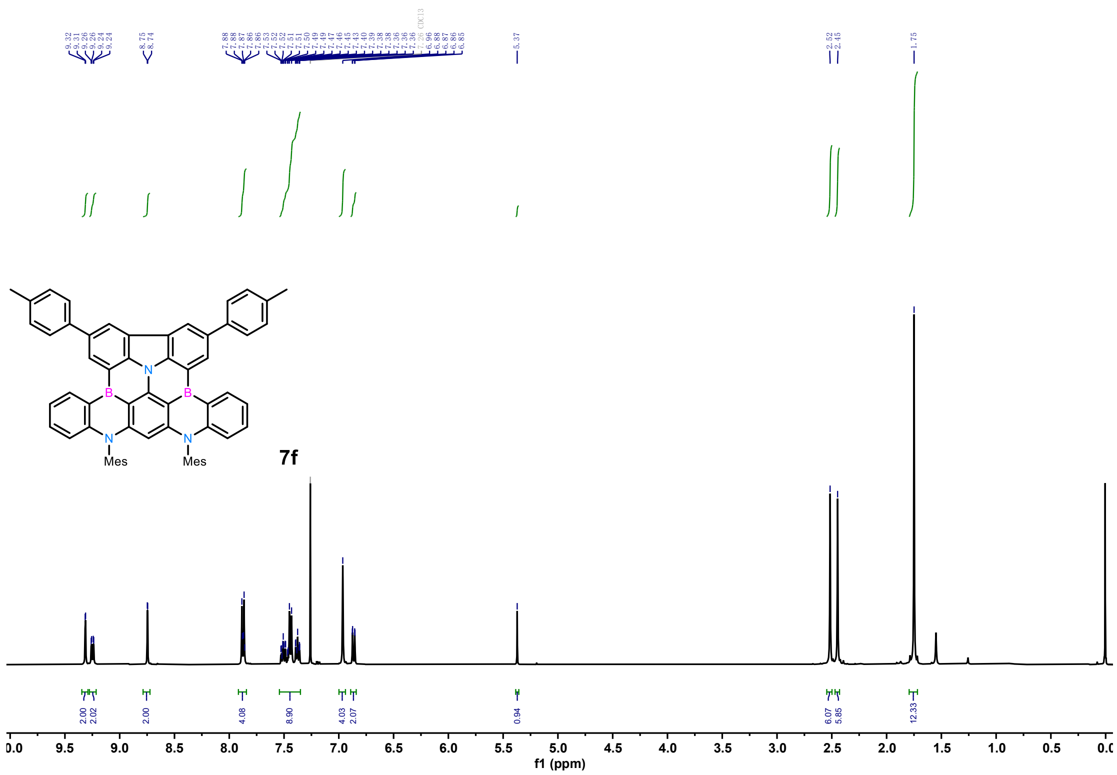

^{13}C NMR spectrum (101 MHz, $CDCl_3$, 298 K) of compound **7f**

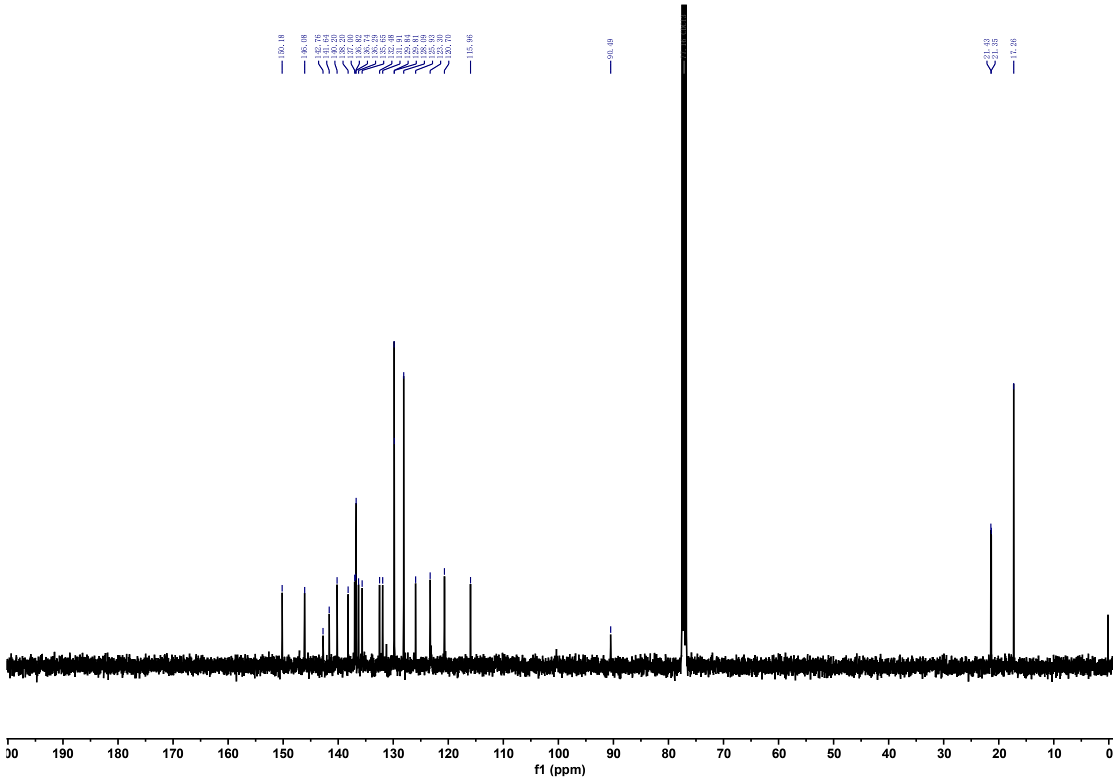

^{1}H NMR spectrum (400 MHz, $CDCl_3$, 298 K) of compound **7g**

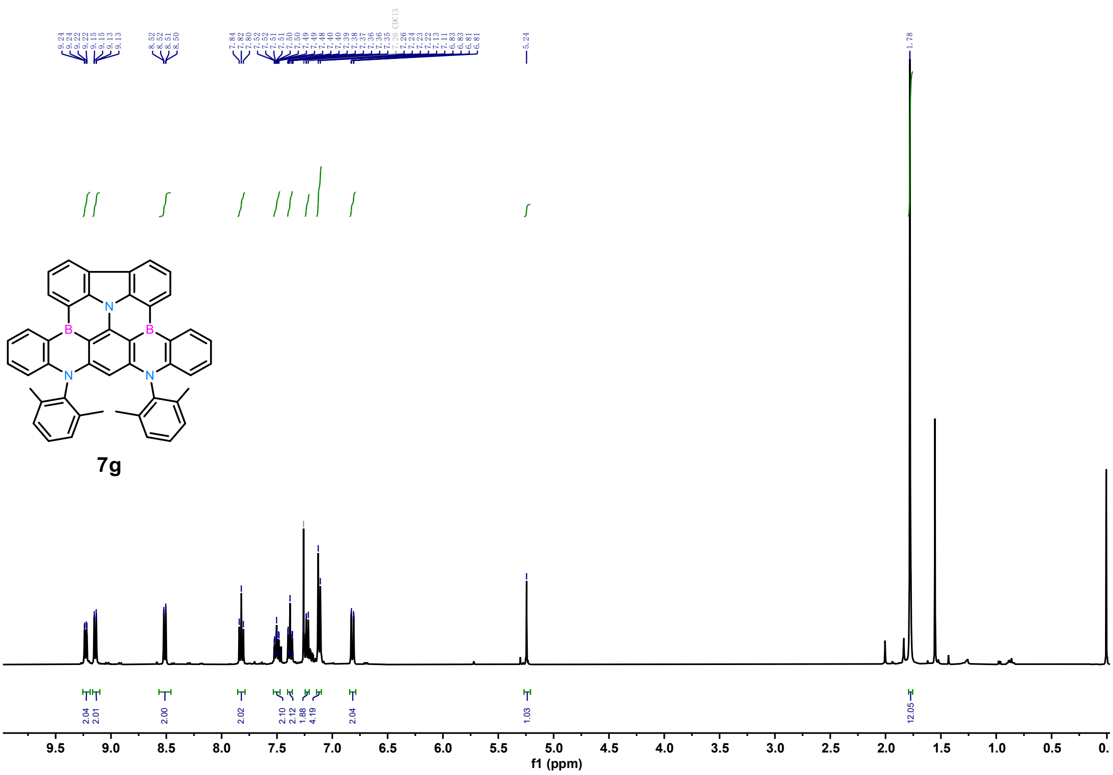

^{13}C NMR spectrum (101 MHz, $CDCl_3$, 298 K) of compound **7g**

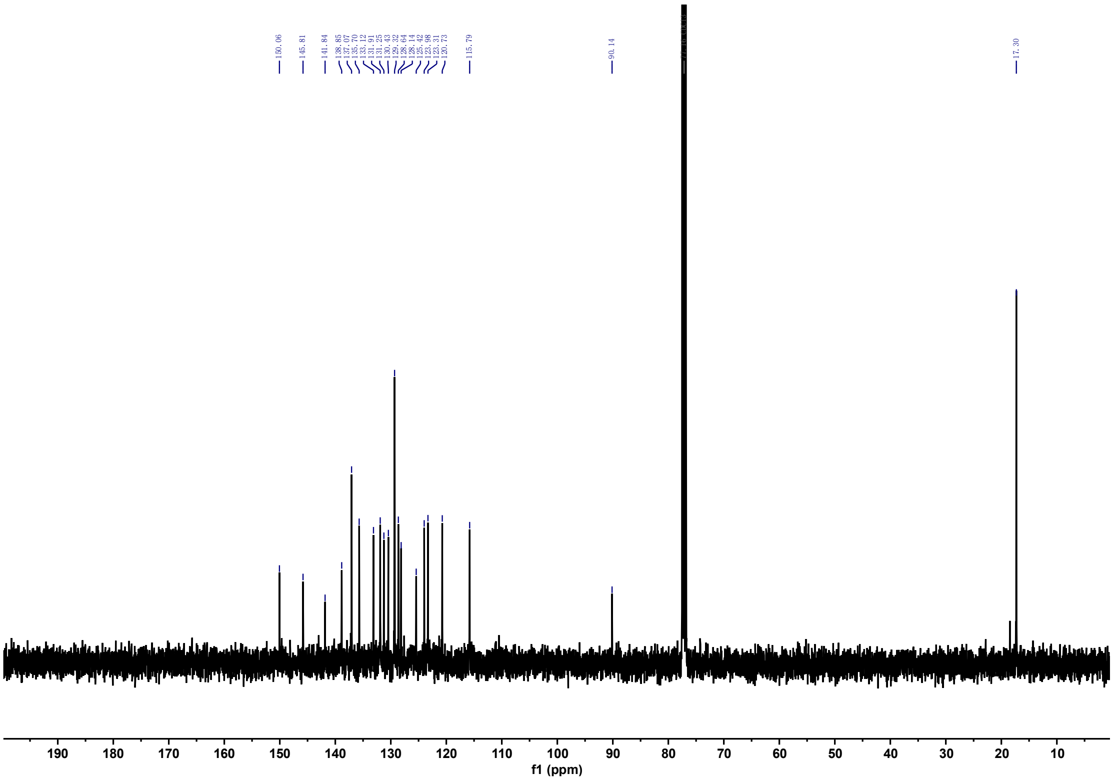

^{1}H NMR spectrum (400 MHz, CDCl$_3$, 298 K) of compound **7h**

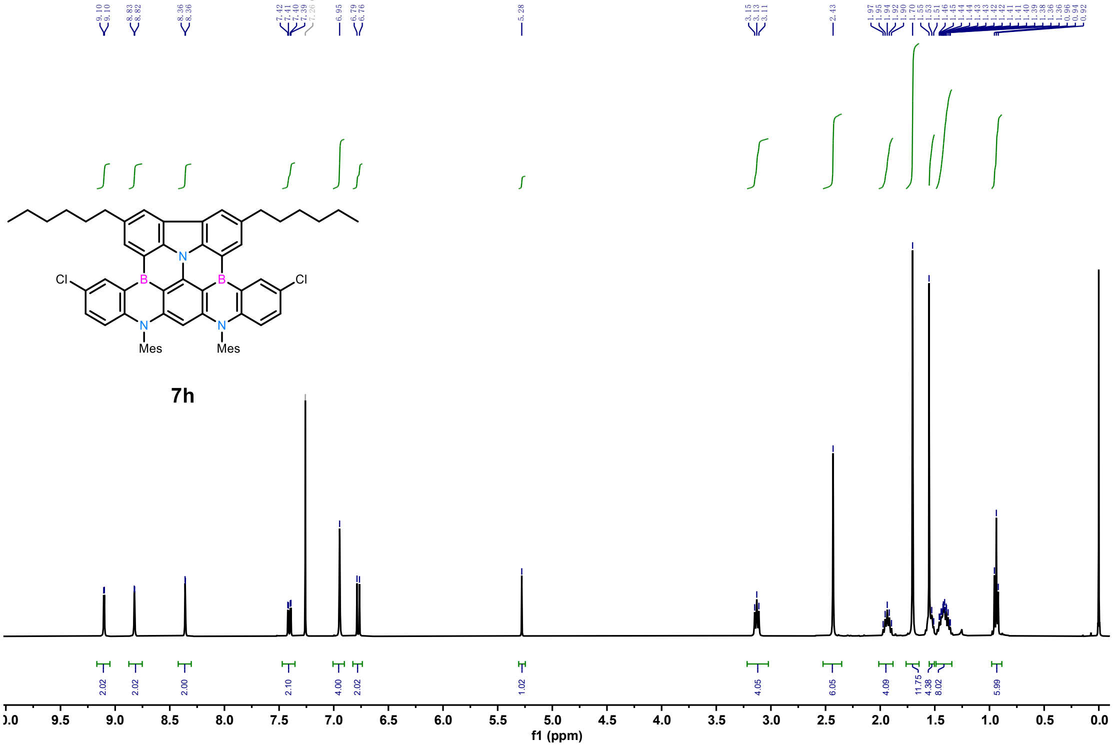

^{13}C NMR spectrum (101 MHz, CDCl$_3$, 298 K) of compound **7h**

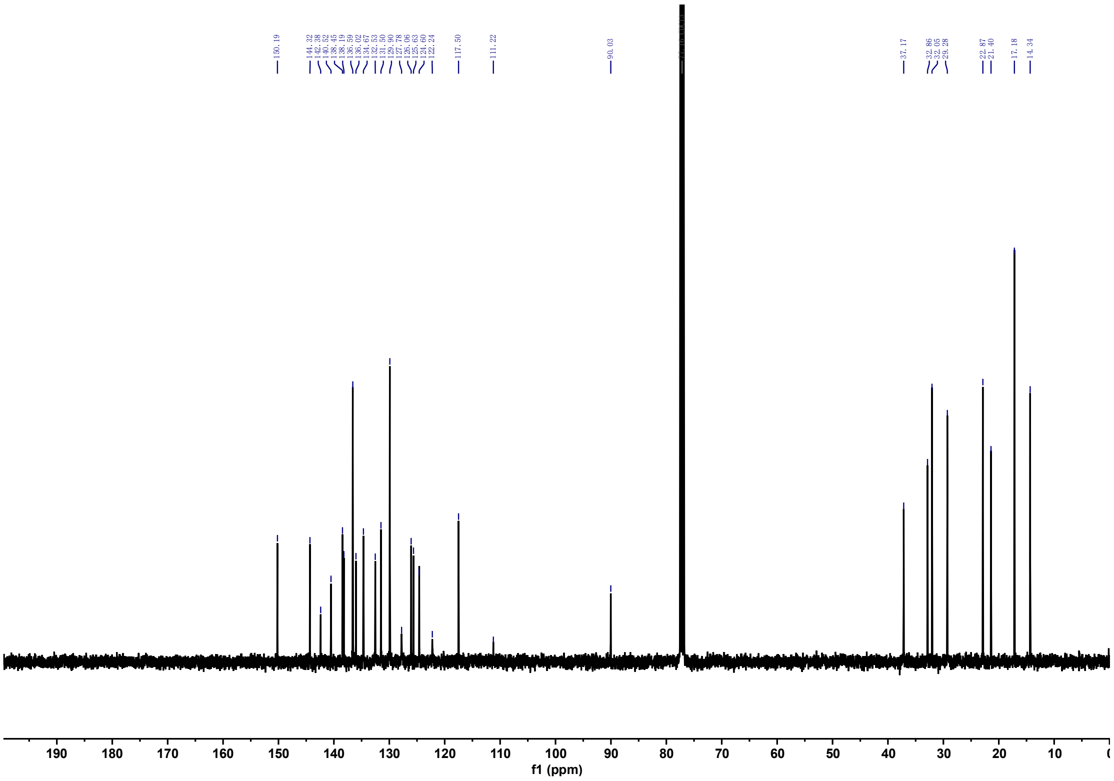

^{1}H NMR spectrum (400 MHz, $CDCl_3$, 298 K) of compound **7i**

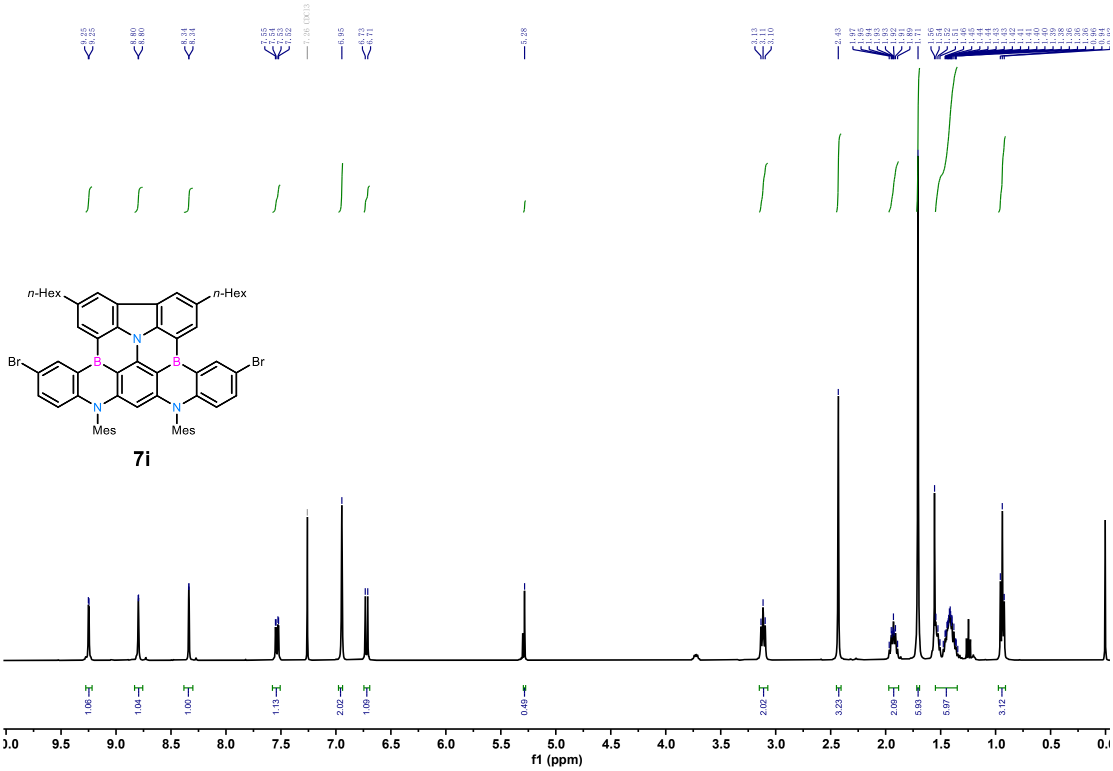

^{13}C NMR spectrum (101 MHz, $CDCl_3$, 298 K) of compound **7i**

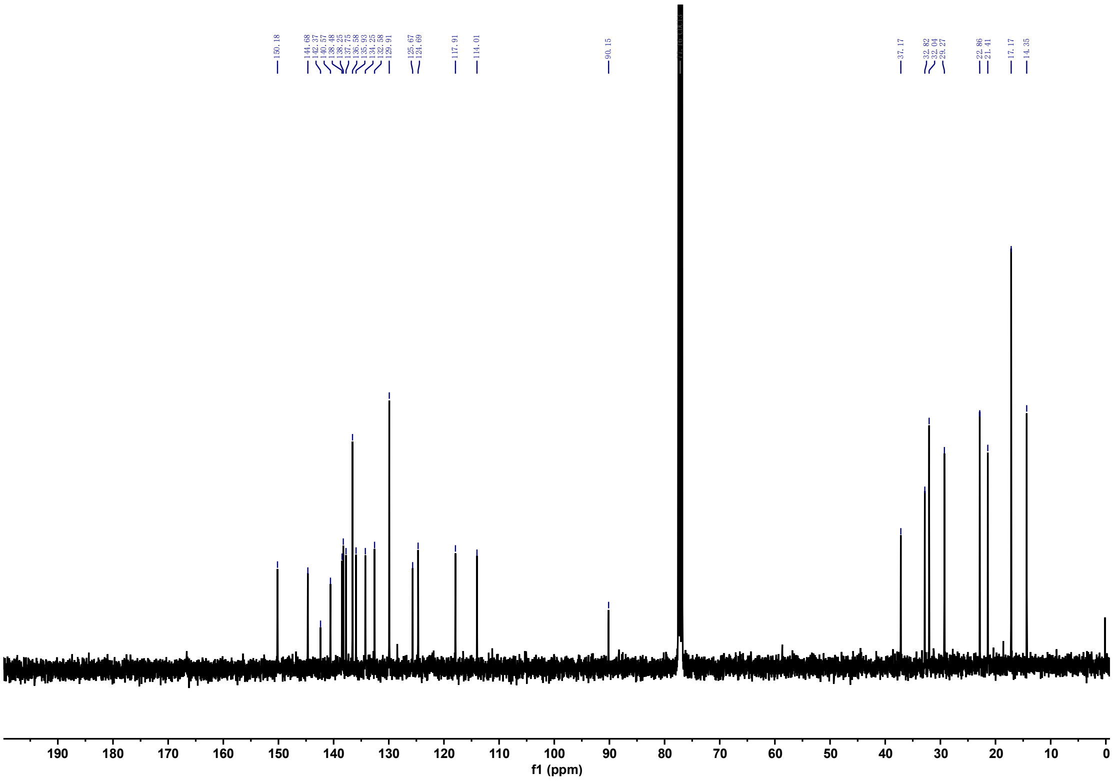

^{1}H NMR spectrum (400 MHz, $CDCl_3$, 298 K) of compound **7j**

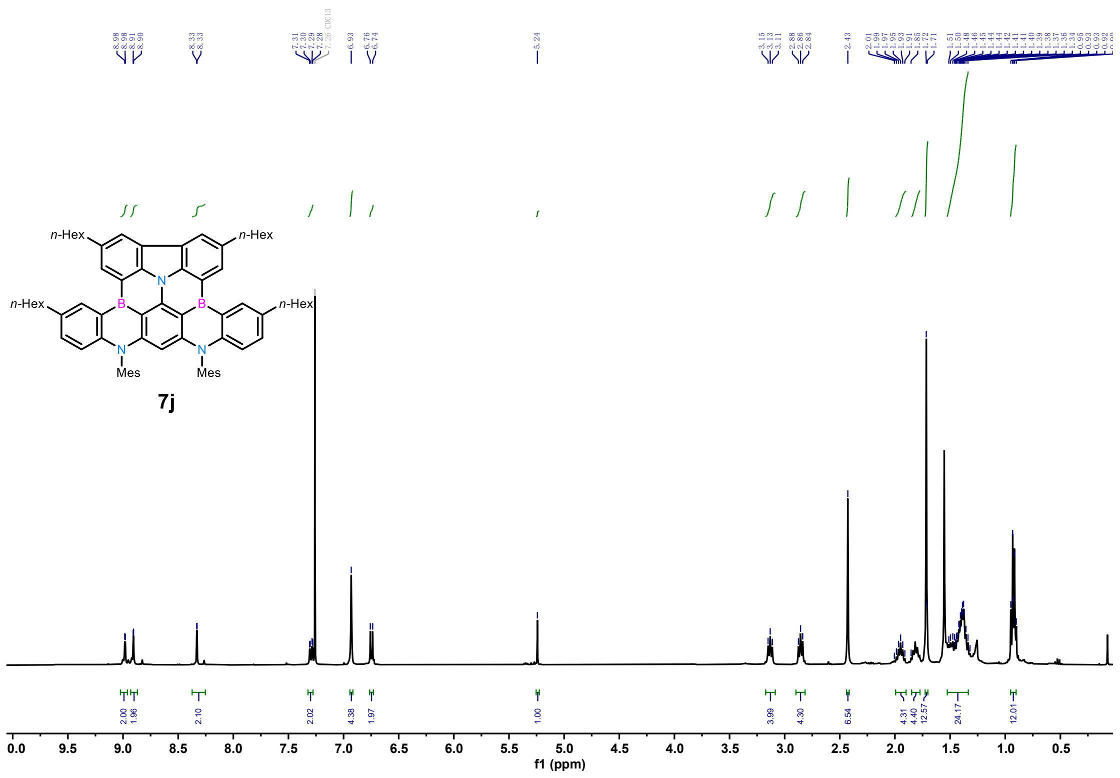

^{13}C NMR spectrum (101 MHz, $CDCl_3$, 298 K) of compound **7j**

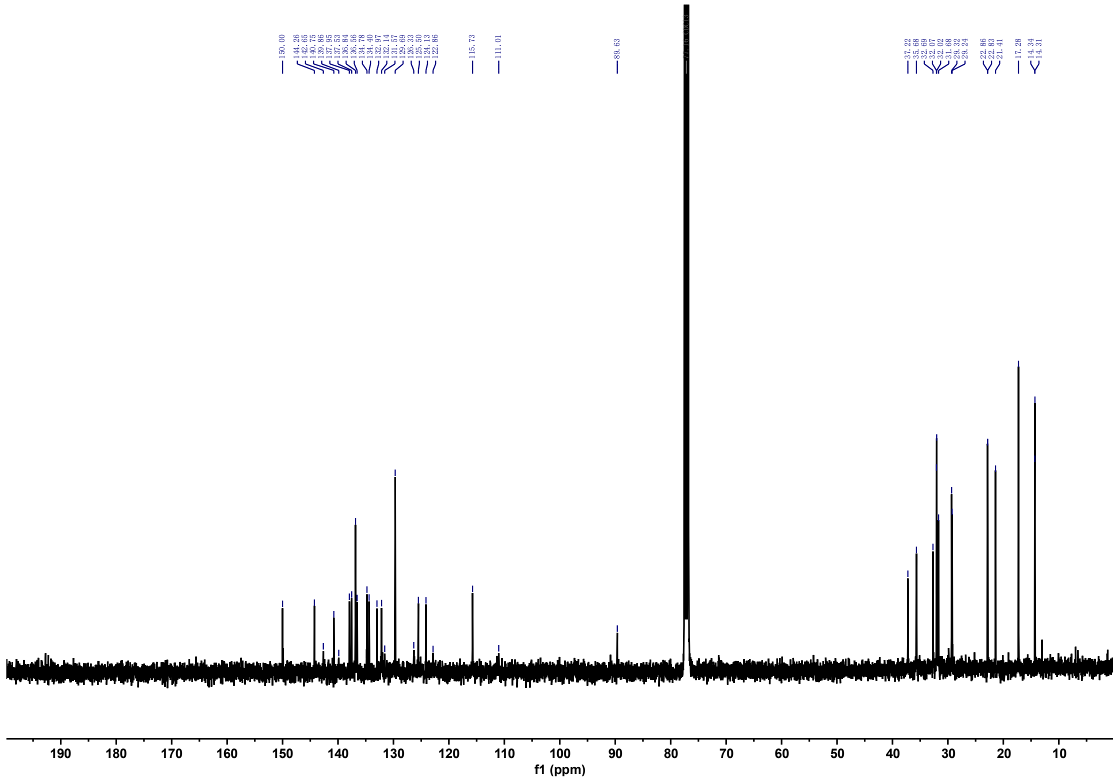

^{1}H NMR spectrum (400 MHz, $CDCl_3$, 298 K) of compound **7k**

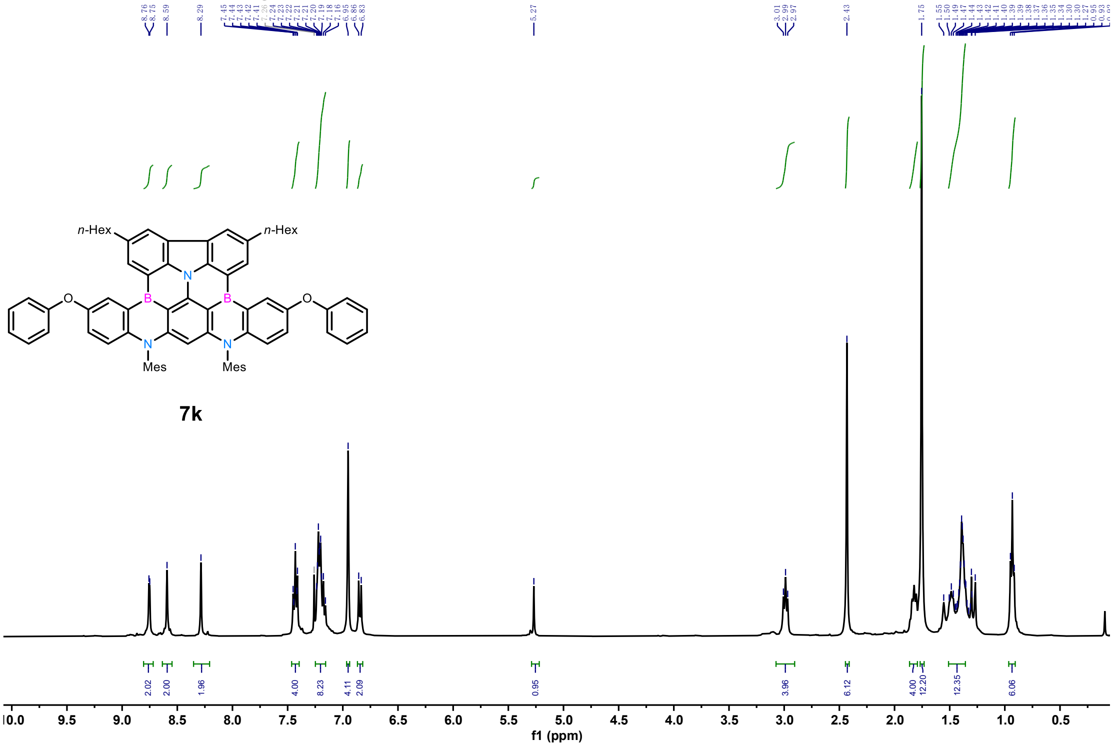

^{13}C NMR spectrum (101 MHz, $CDCl_3$, 298 K) of compound **7k**

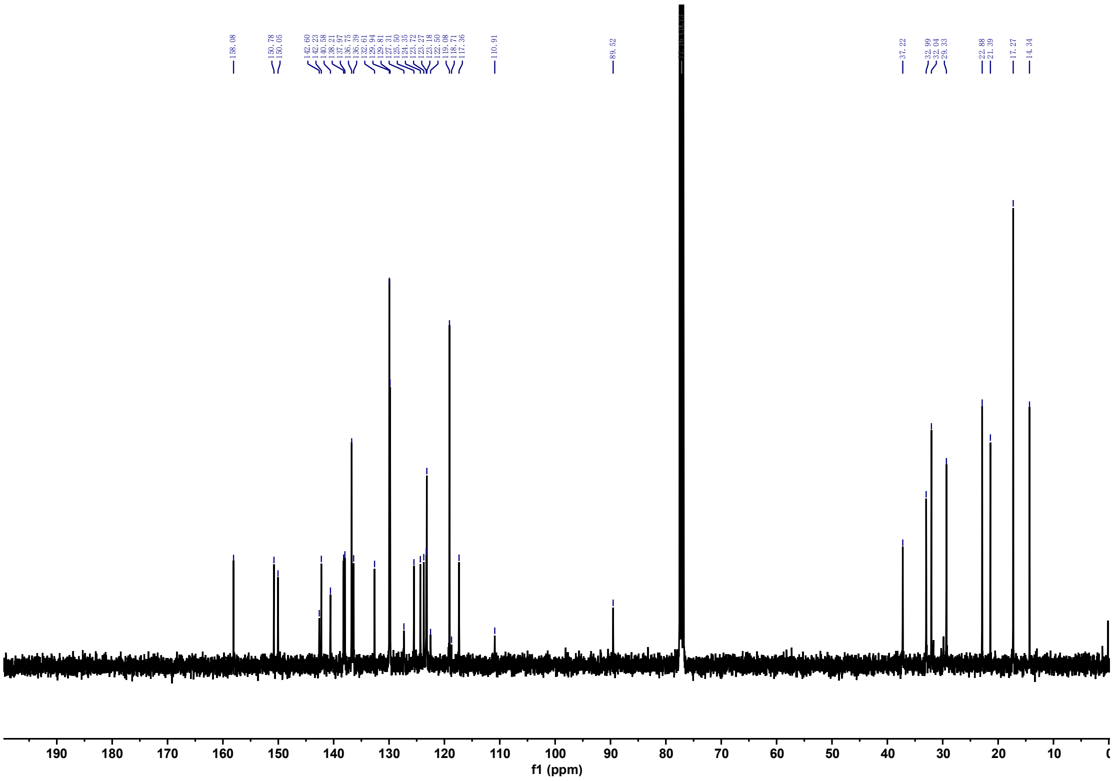

^{1}H NMR spectrum (400 MHz, $CDCl_3$, 298 K) of compound **7l**

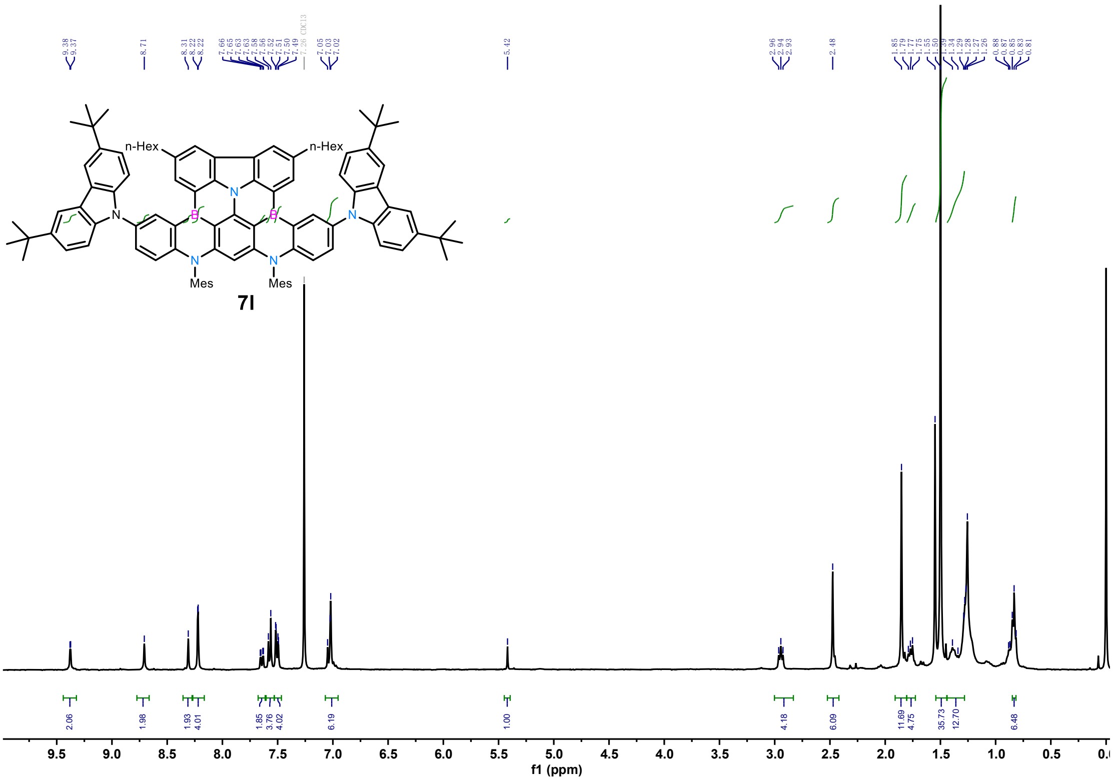

^{13}C NMR spectrum (101 MHz, $CDCl_3$, 298 K) of compound **7l**

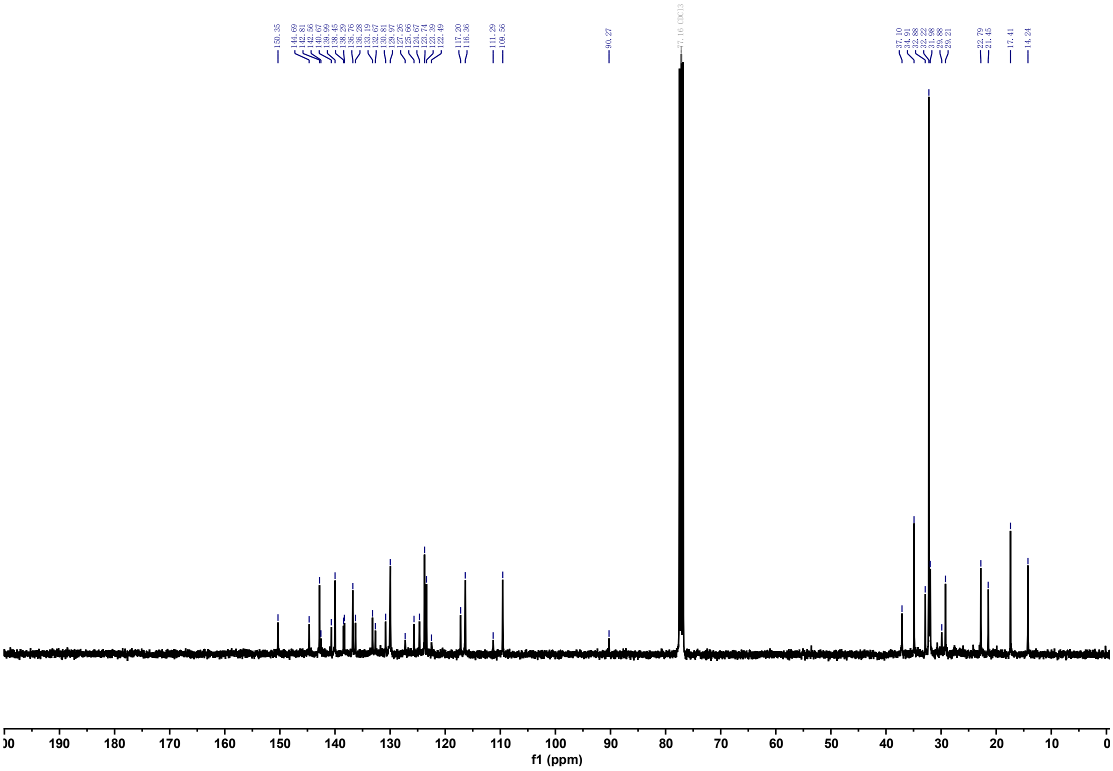

^{1}H NMR spectrum (400 MHz, $CDCl_3$, 298 K) of compound **7m**

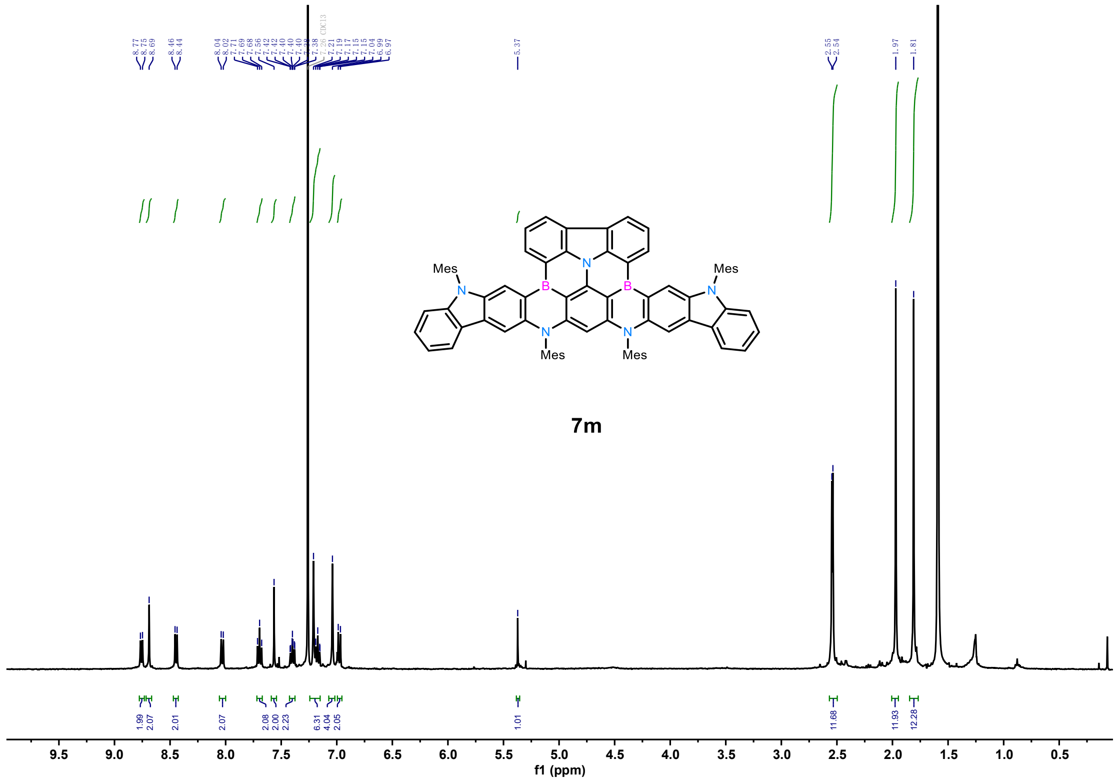

^{13}C NMR spectrum (101 MHz, $CDCl_3$, 298 K) of compound **7m**

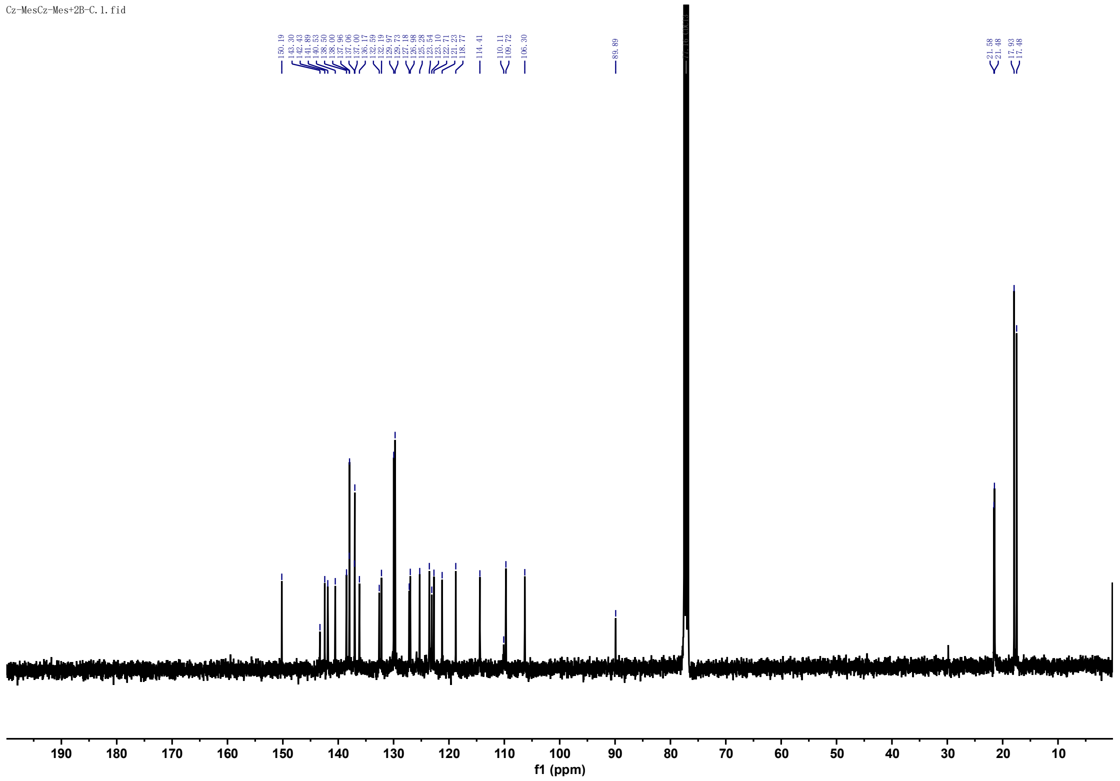

^1H NMR spectrum (400 MHz, $CDCl_3$, 298 K) of compound **7n**

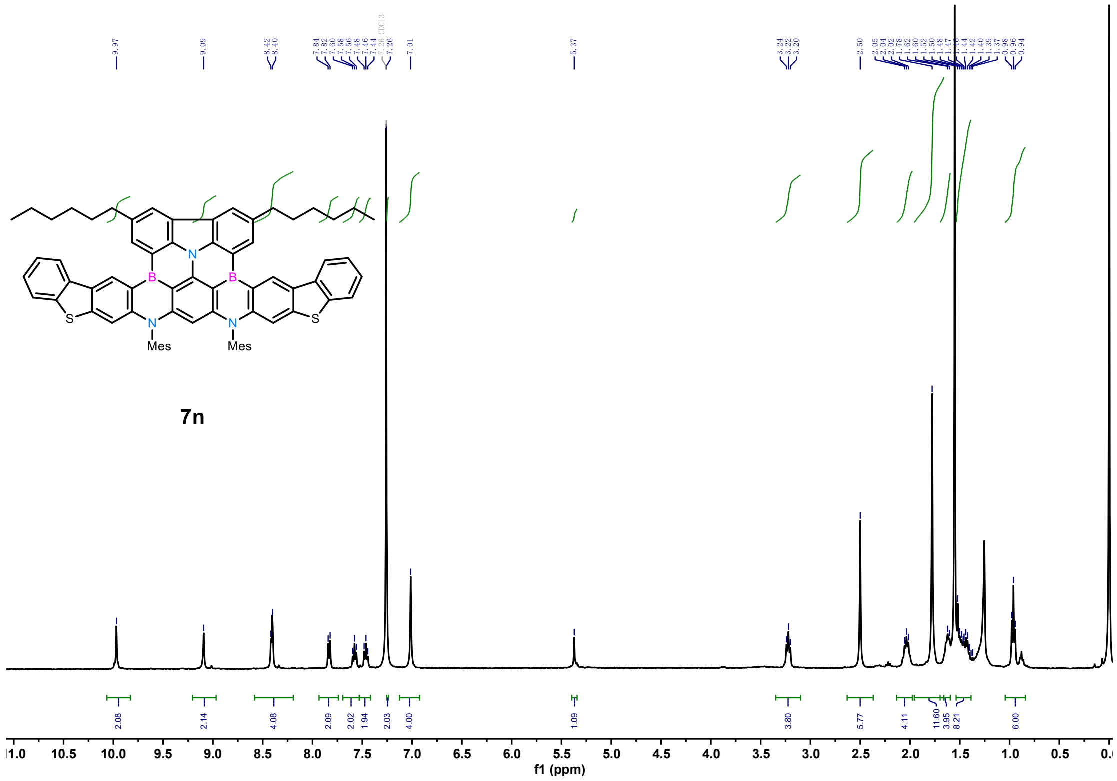

^{13}C NMR spectrum (101 MHz, $CDCl_3$, 298 K) of compound **7n**

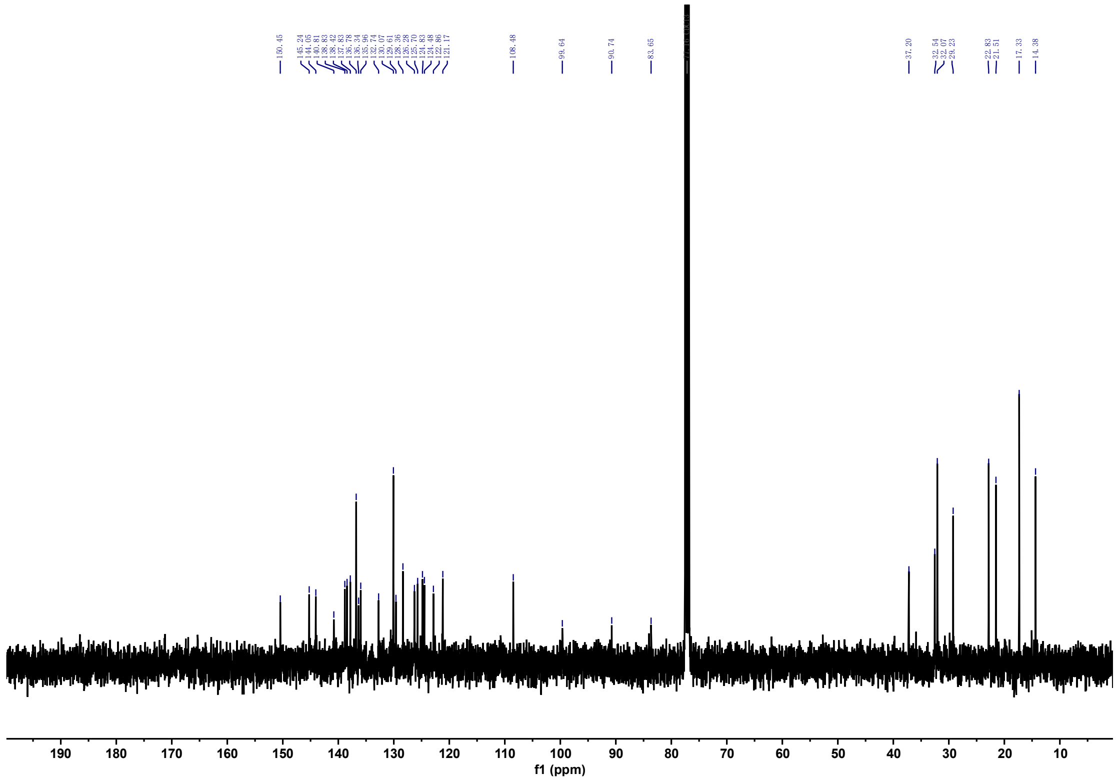

^{1}H NMR spectrum (400 MHz, $CDCl_3$, 298 K) of compound **7o**

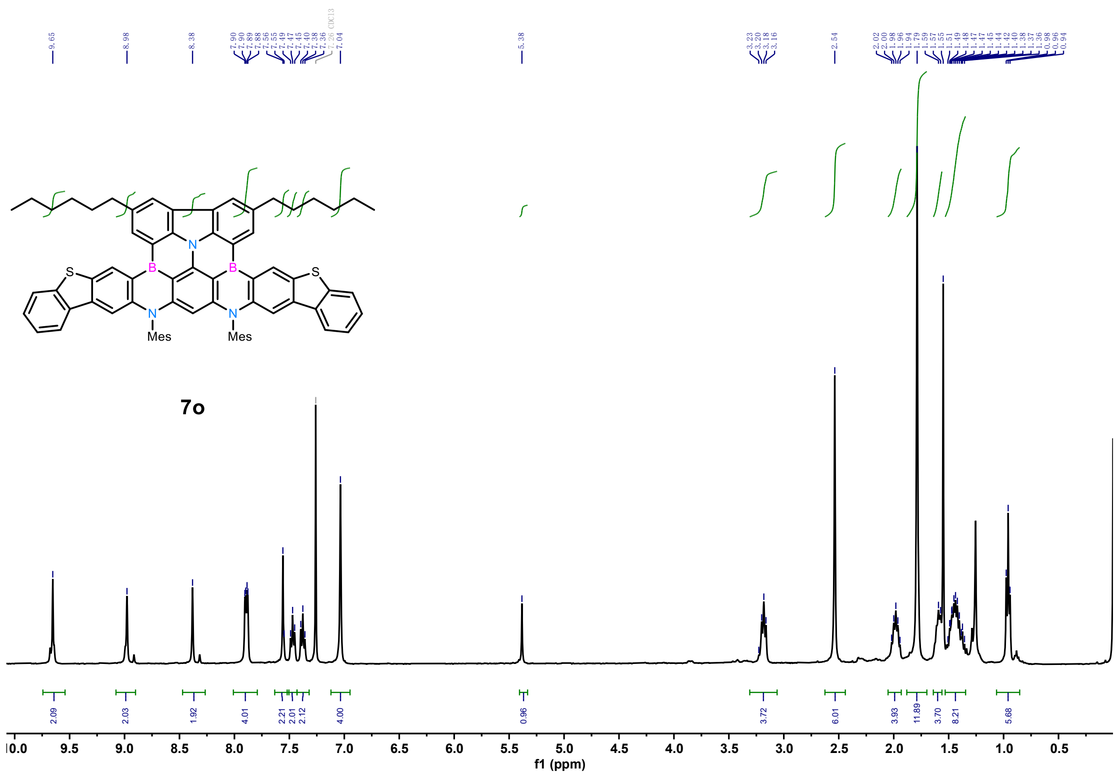

^{13}C NMR spectrum (101 MHz, $CDCl_3$, 298 K) of compound **7o**

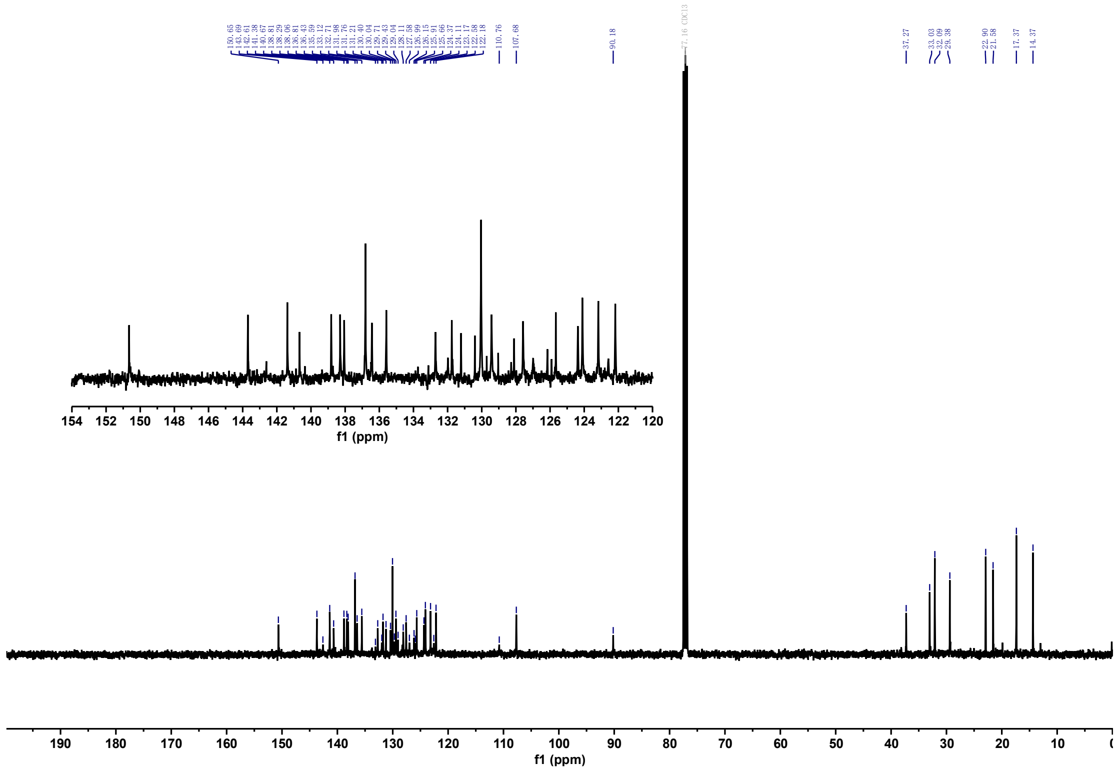

^{1}H NMR spectrum (400 MHz, $CDCl_3$, 298 K) of compound **7p**

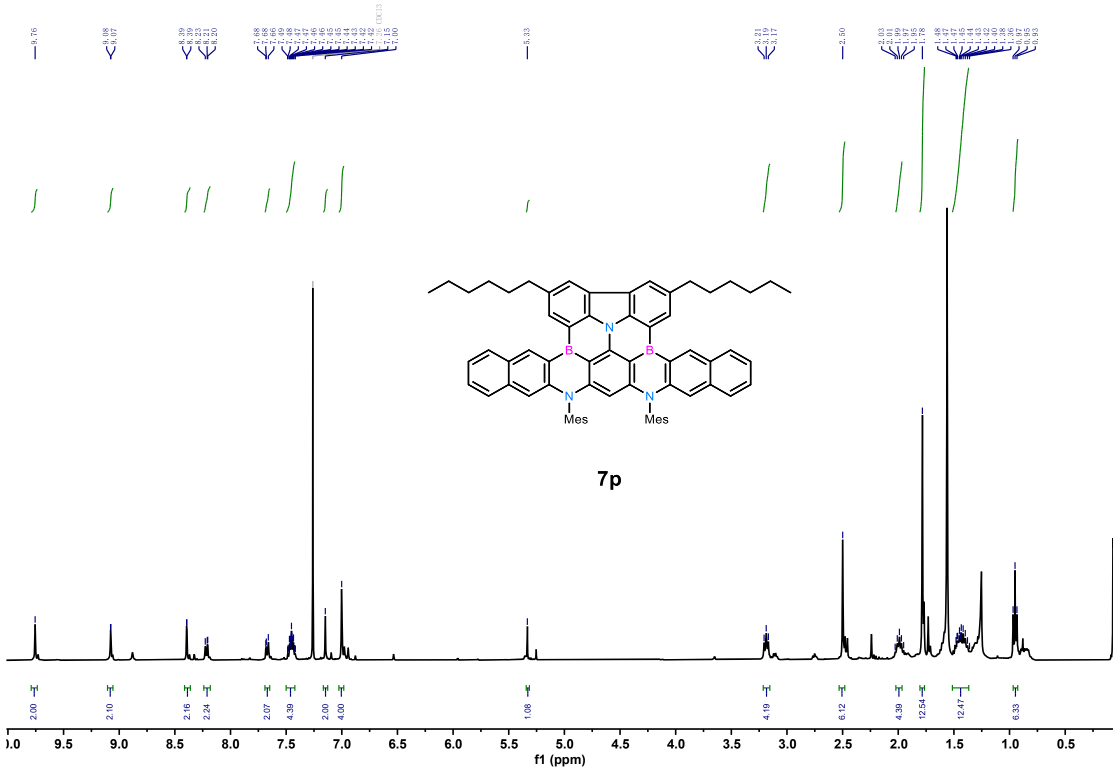

^{13}C NMR spectrum (101 MHz, $CDCl_3$, 298 K) of compound **7p**

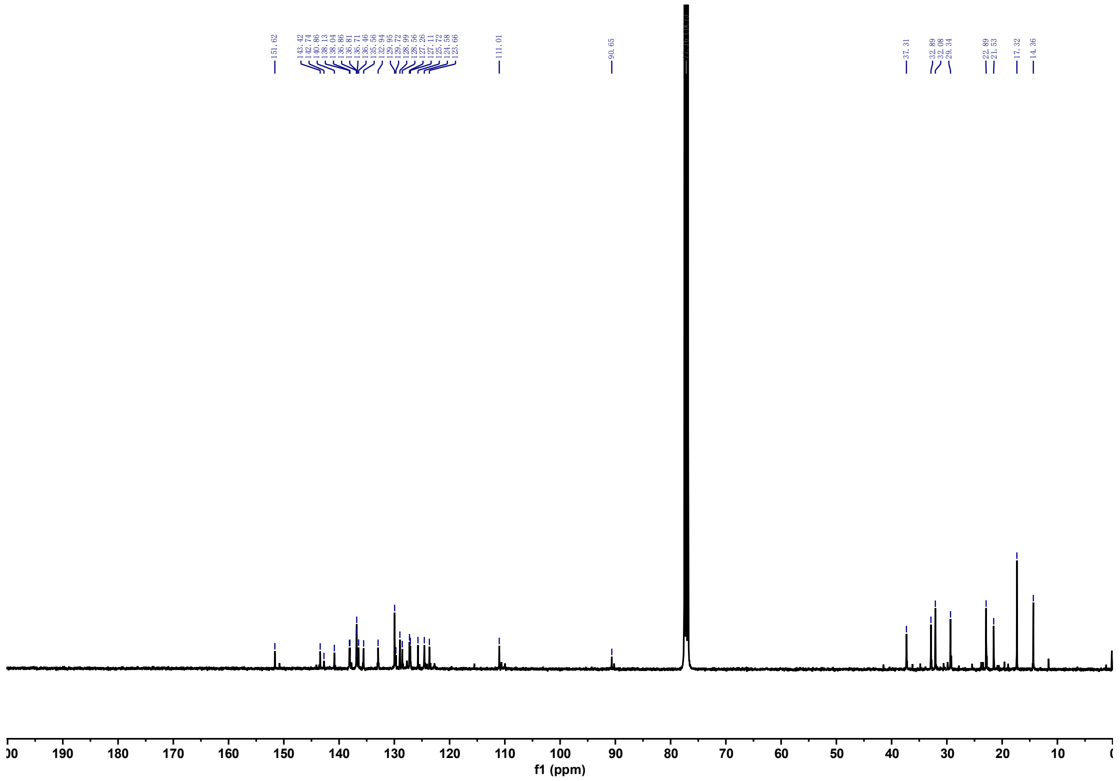

^{1}H NMR spectrum (400 MHz, CDCl$_3$, 298 K) of compound **7q**

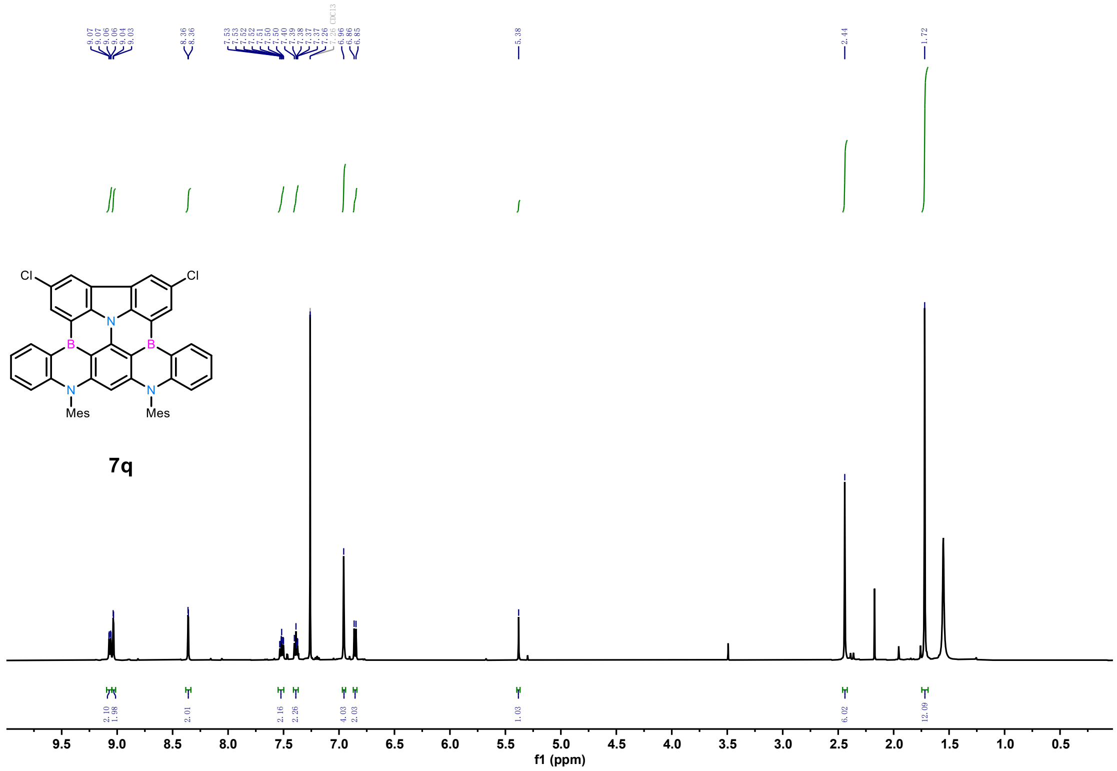

^{13}C NMR spectrum (101 MHz, CDCl$_3$/CS$_2$, 298 K) of compound **7q**

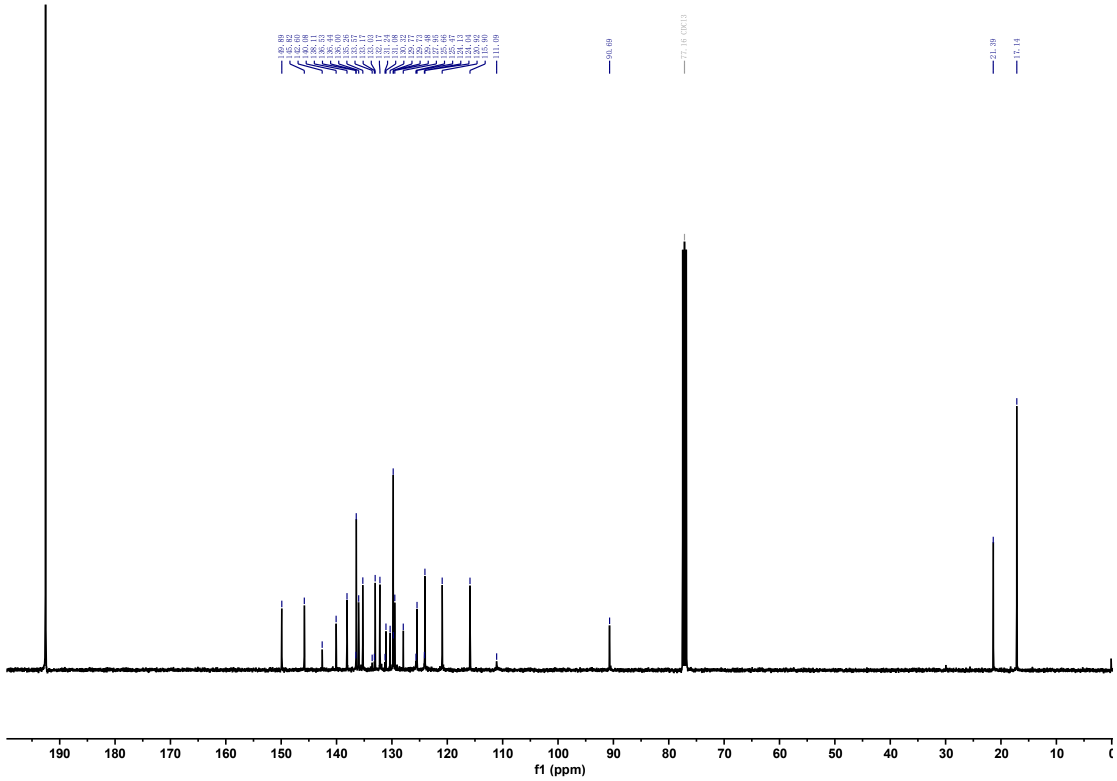

^{1}H NMR spectrum (400 MHz, $CDCl_3$, 298 K) of compound **8a**

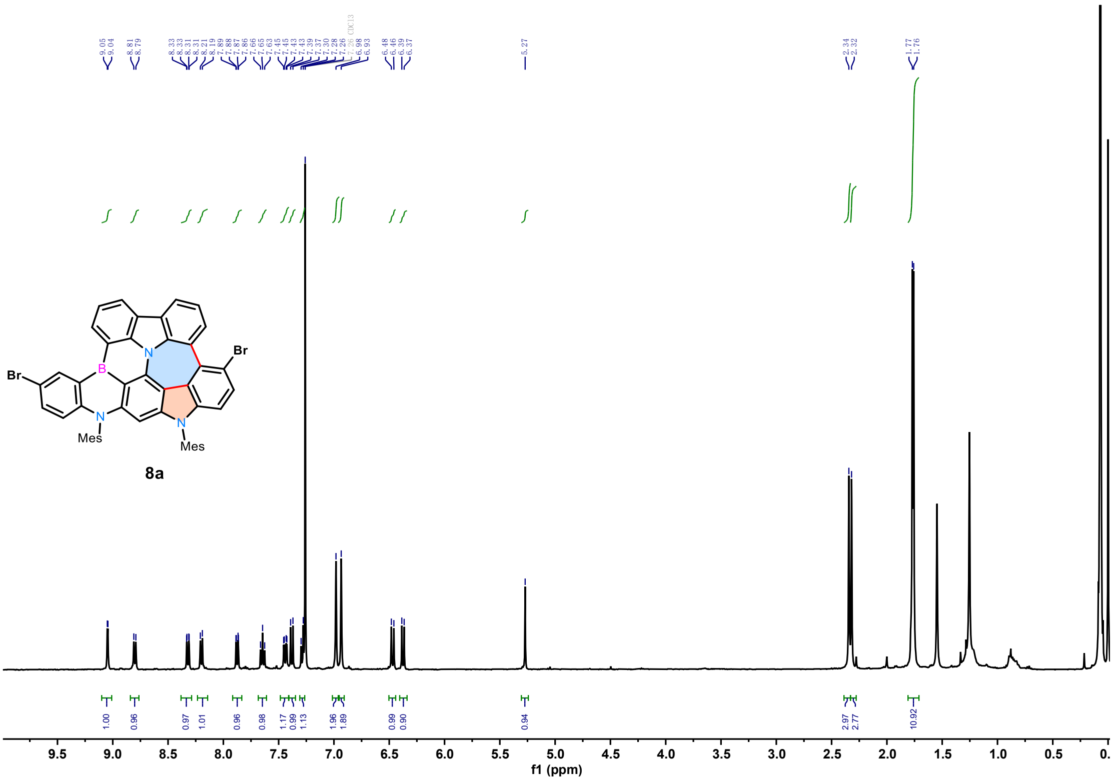

^{13}C NMR spectrum (101 MHz, $CDCl_3$, 298 K) of compound **8a**

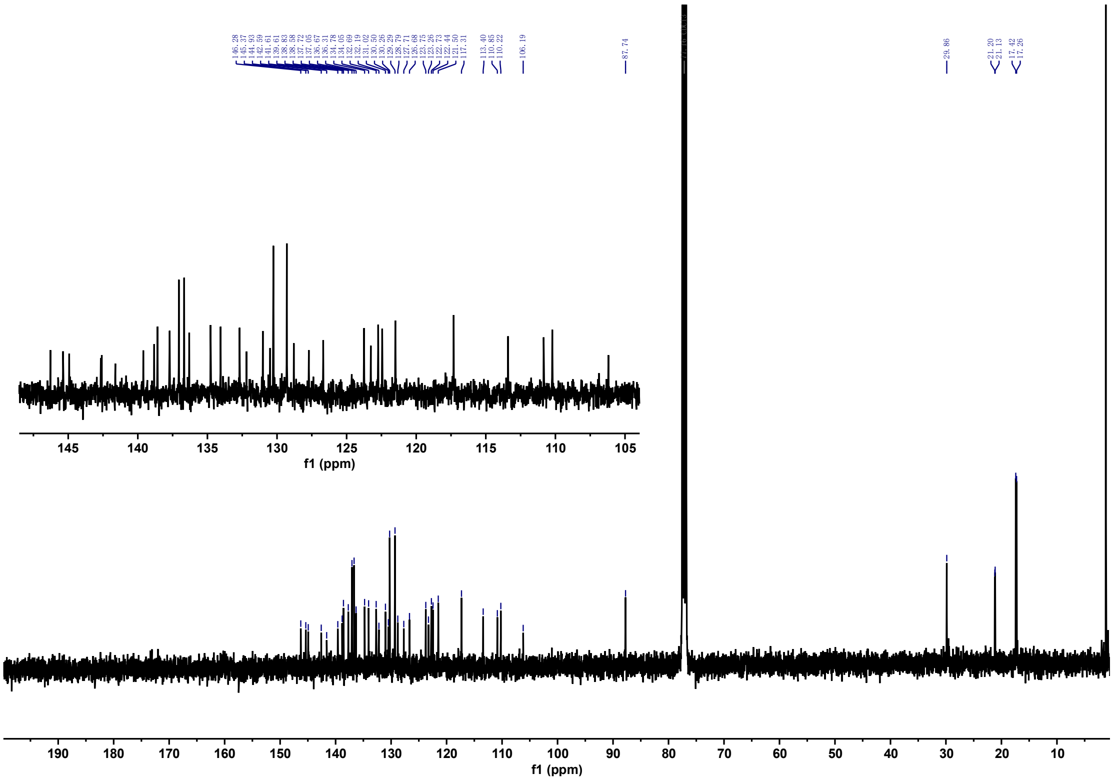

^{1}H NMR spectrum (600 MHz, C_6D_6, 298 K) of compound **8b**

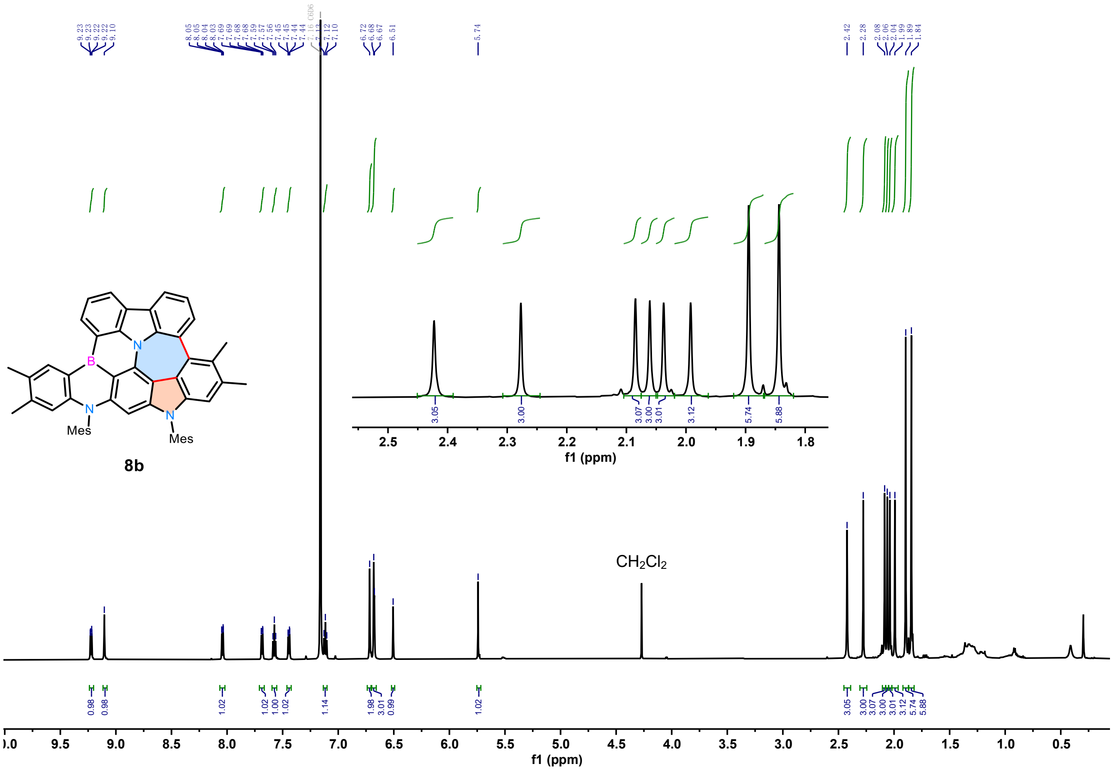

^{13}C NMR spectrum (151 MHz, C_6D_6, 298 K) of compound **8b**

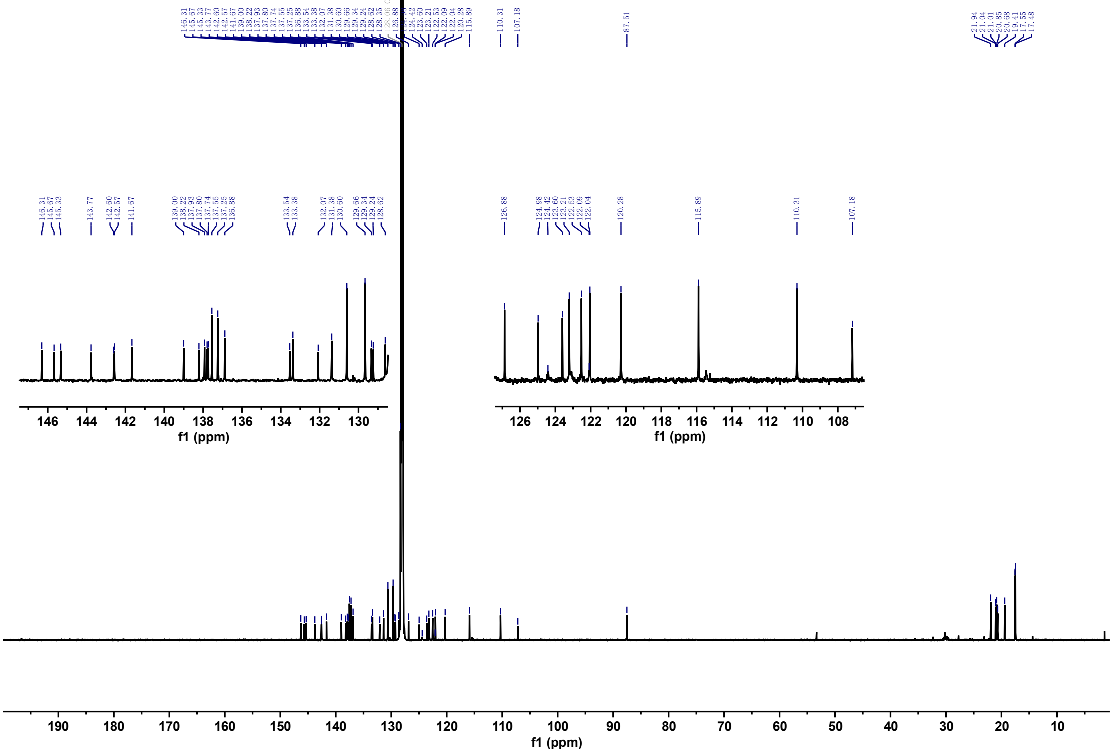

^{1}H NMR spectrum (400 MHz, $CDCl_3$, 298 K) of compound **8c**

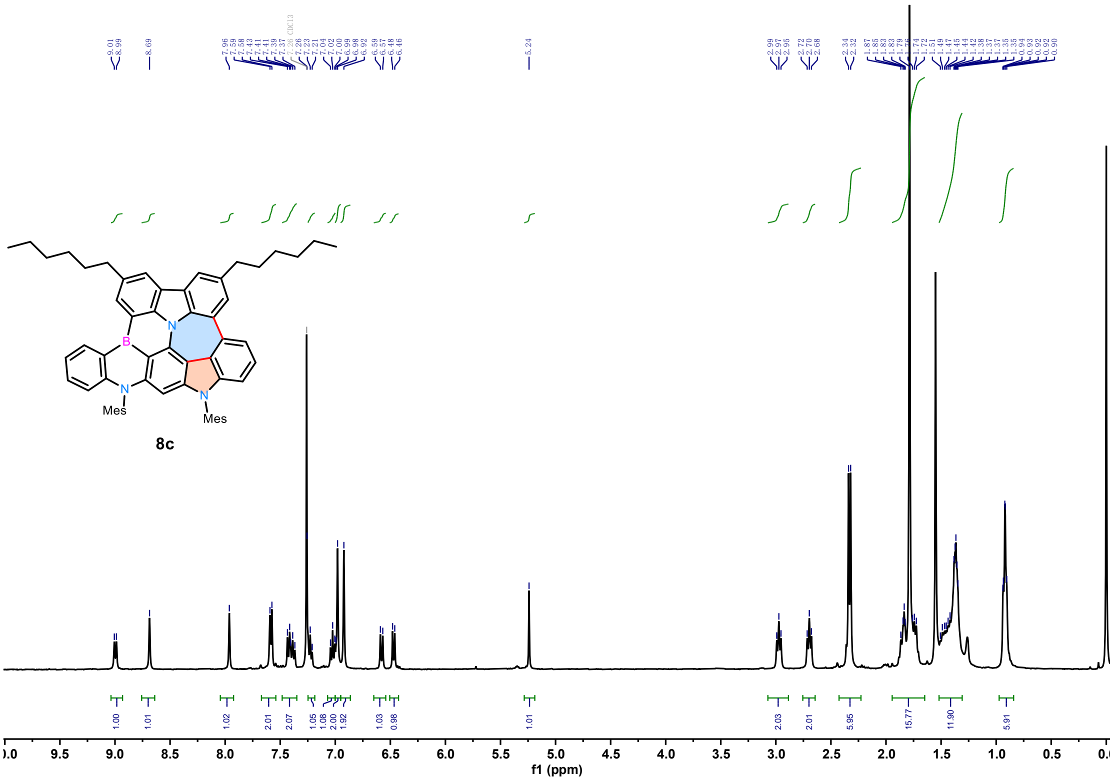

^{13}C NMR spectrum (101 MHz, $CDCl_3$, 298 K) of compound **8c**

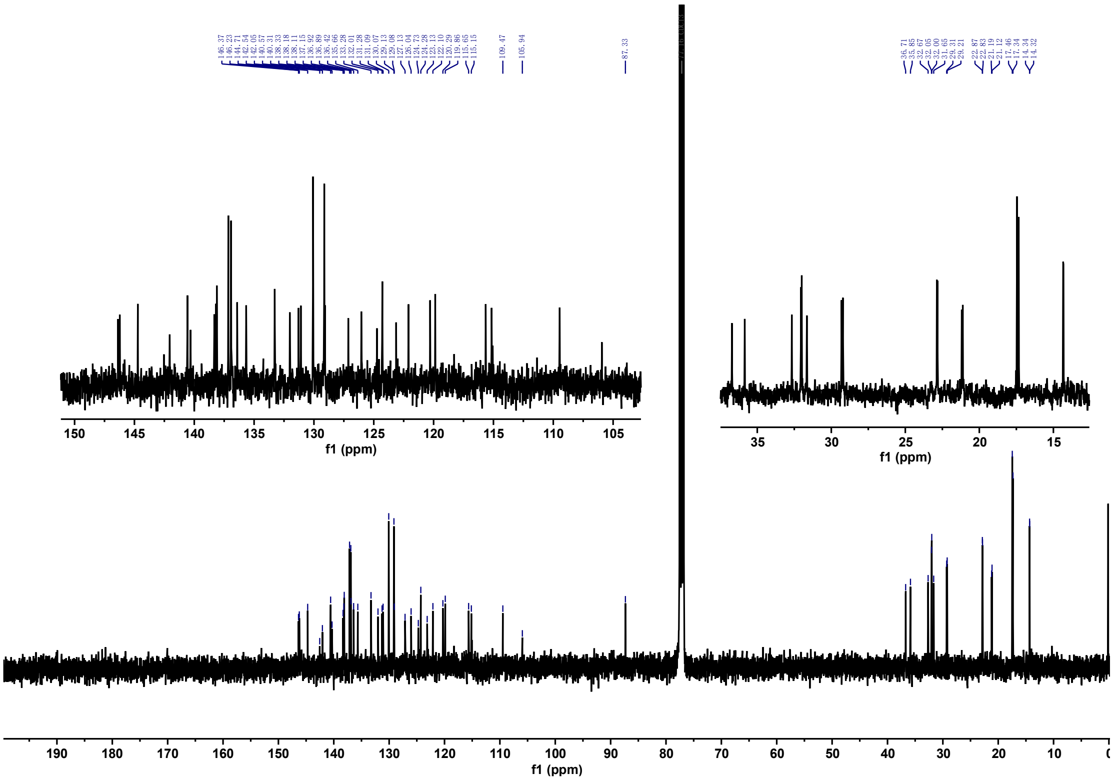

^{1}H NMR spectrum (400 MHz, $CDCl_3$, 298 K) of compound **8d**

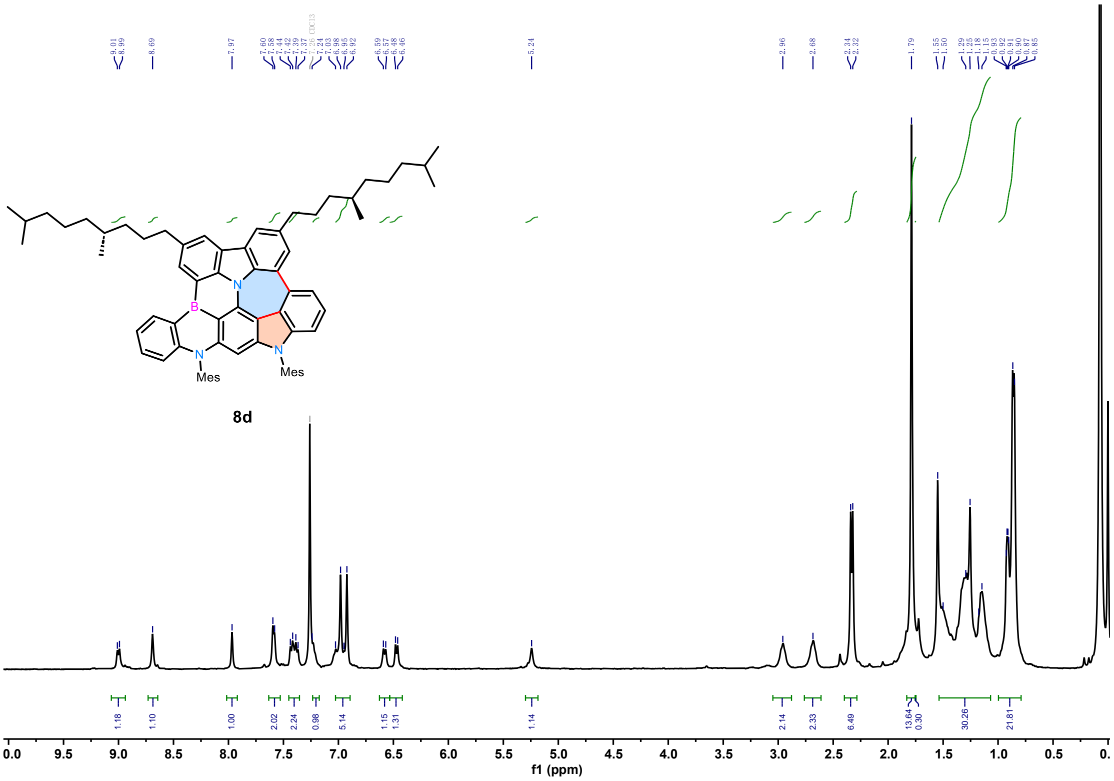

^{13}C NMR spectrum (101 MHz, $CDCl_3$, 298 K) of compound **8d**

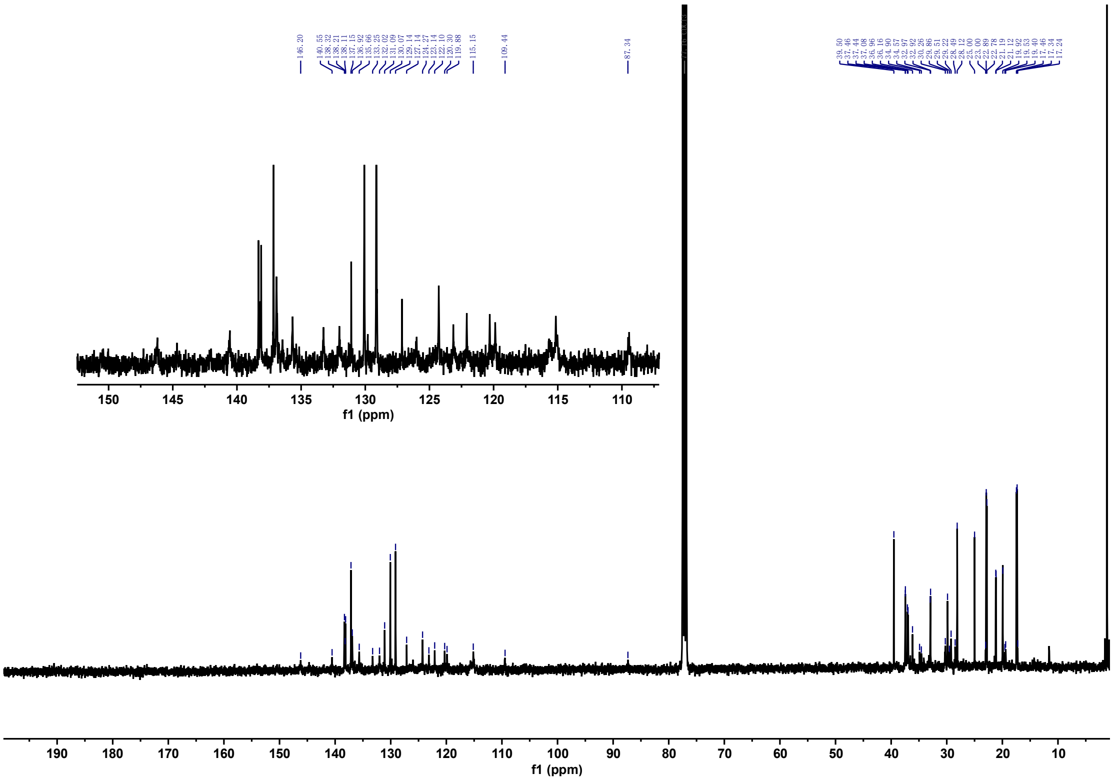

^{1}H NMR spectrum (400 MHz, $CDCl_3$, 298 K) of compound **8e**

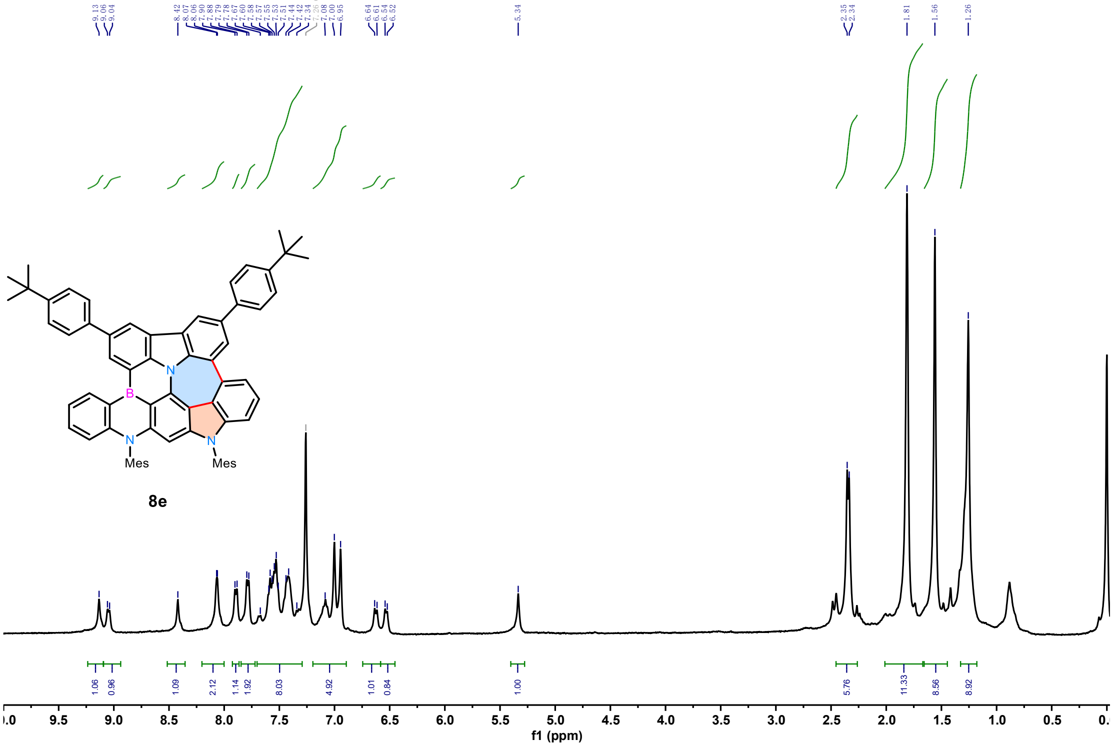

8e

^{13}C NMR spectrum (101 MHz, $CDCl_3$, 298 K) of compound **8e**

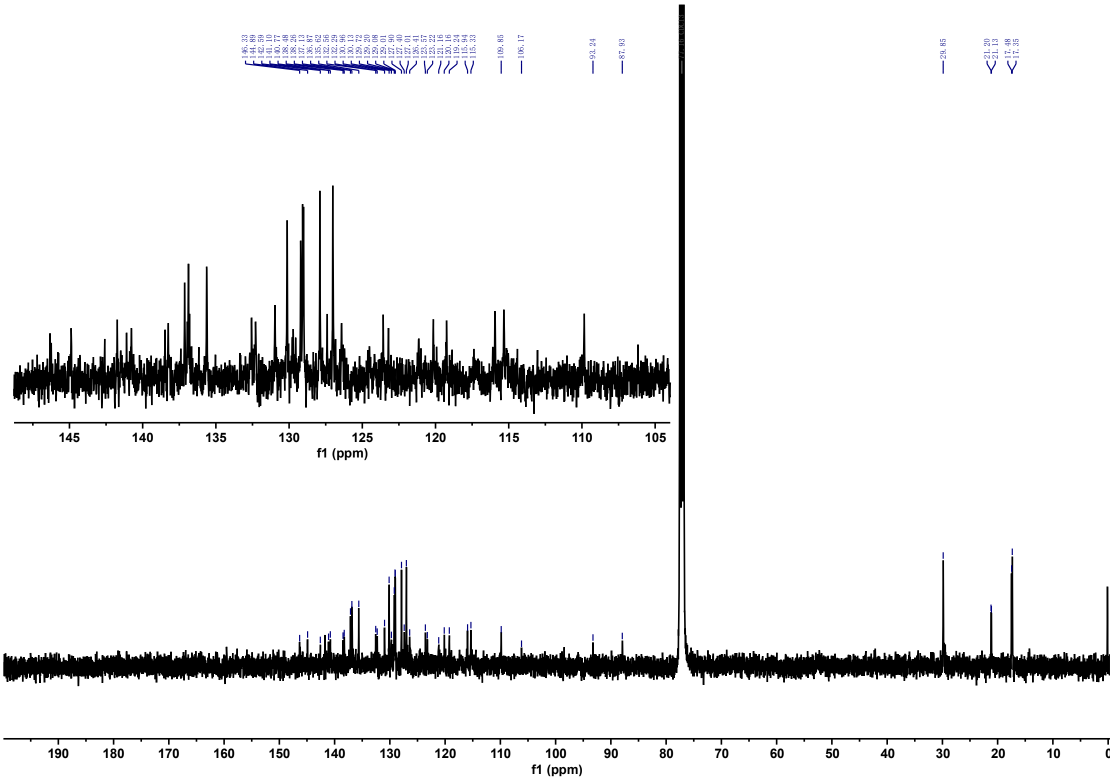

¹H NMR spectrum (400 MHz, $CDCl_3$, 298 K) of compound **8f**

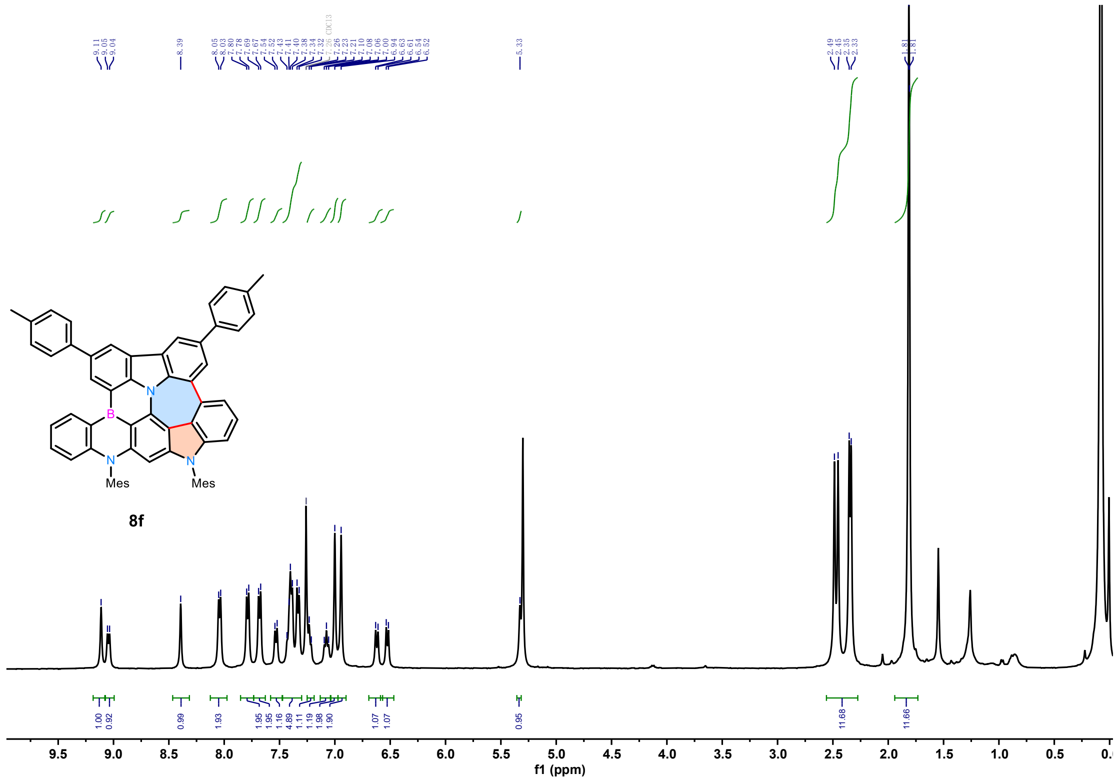

¹³C NMR spectrum (101 MHz, $CDCl_3$, 298 K) of compound **8f**

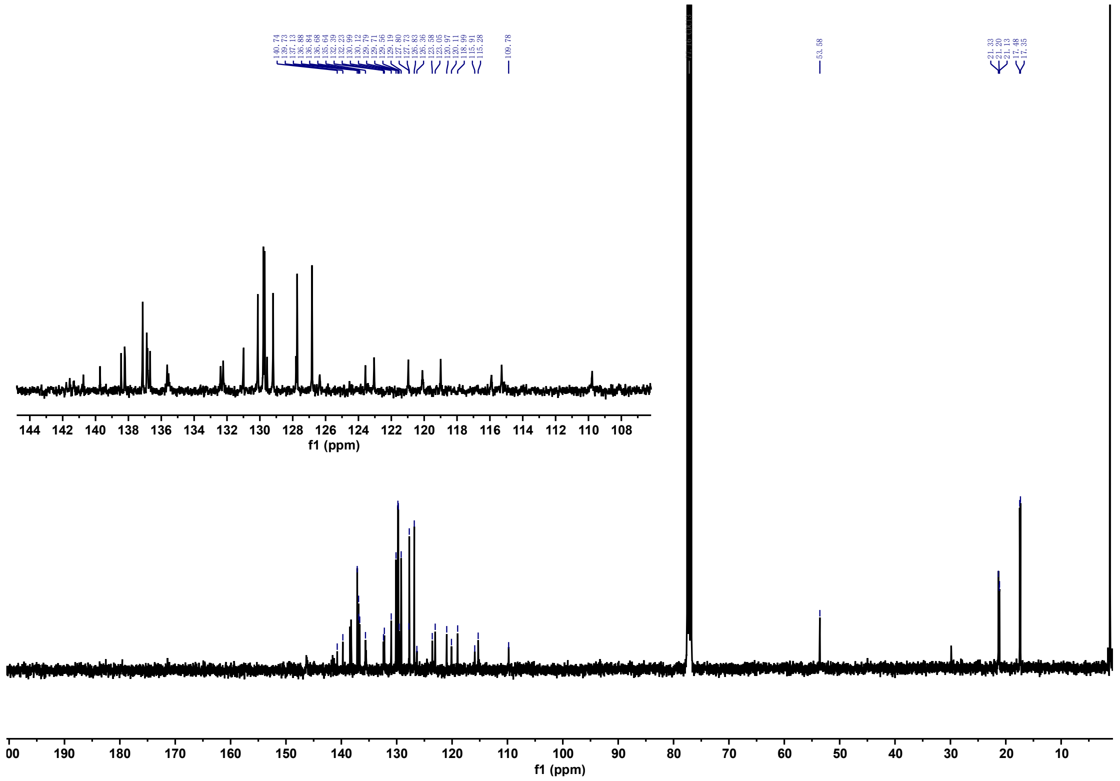

^{1}H NMR spectrum (600 MHz, Acetone-*d6*/CS_2, 298 K) of compound **8g**

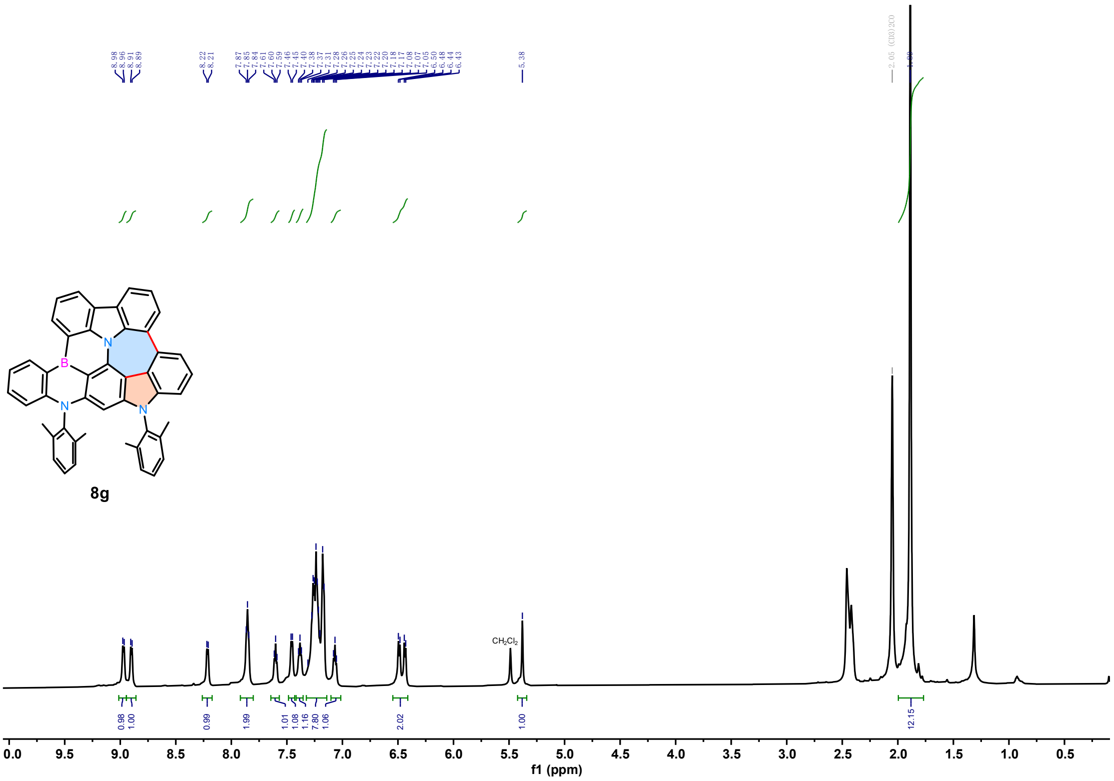

^{13}C NMR spectrum (151 MHz, Acetone-*d6*/CS_2, 298 K) of compound **8g**

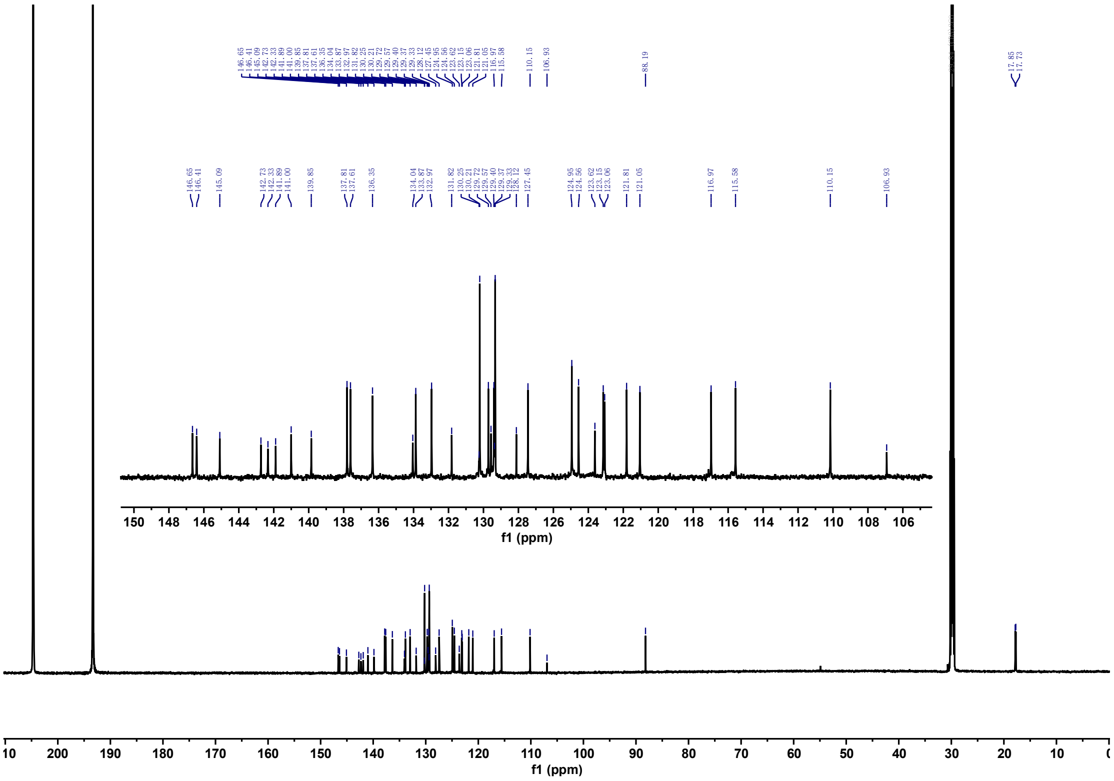

^{1}H NMR spectrum (400 MHz, $CDCl_3$, 298 K) of compound **8h**

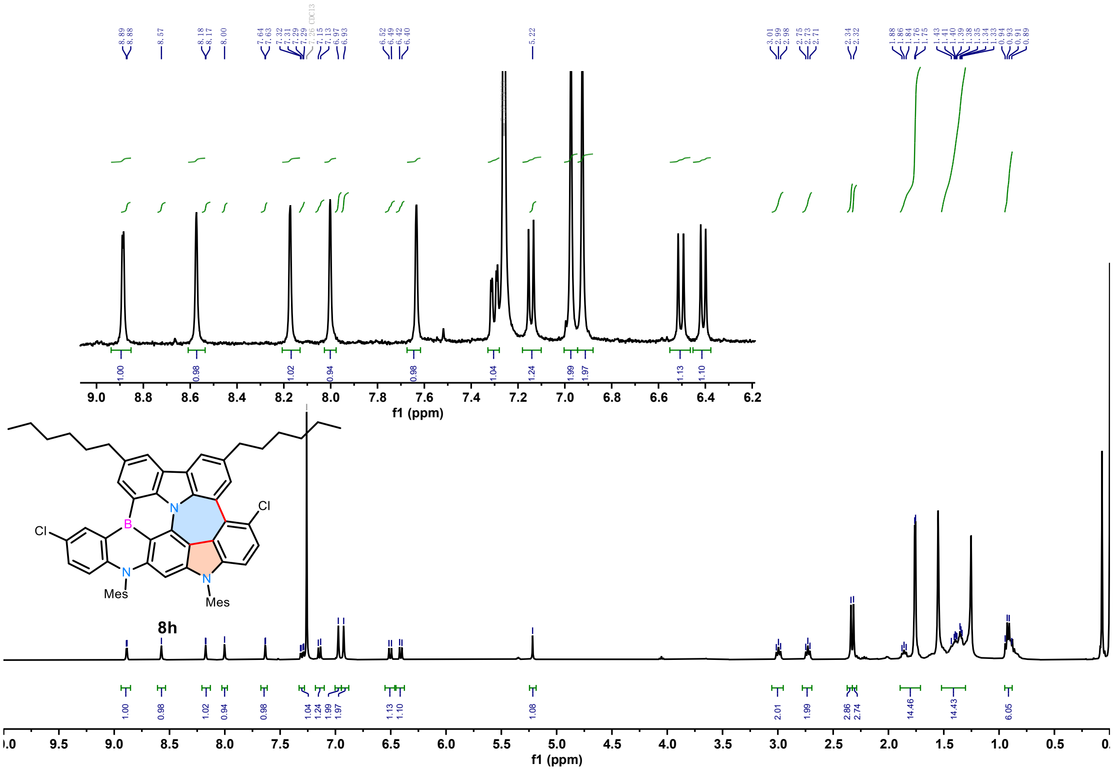

^{13}C NMR spectrum (101 MHz, $CDCl_3$, 298 K) of compound **8h**

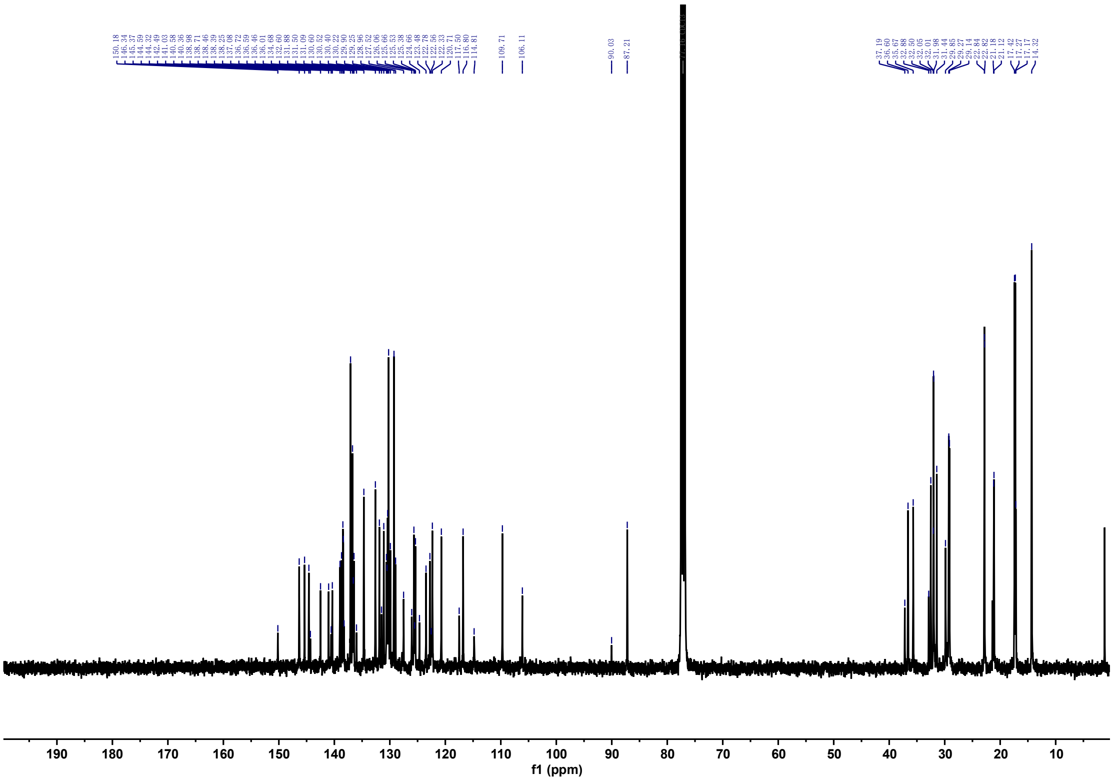

^{1}H NMR spectrum (600 MHz, Acetone-*d6*/CS_2, 298 K) of compound **8i**

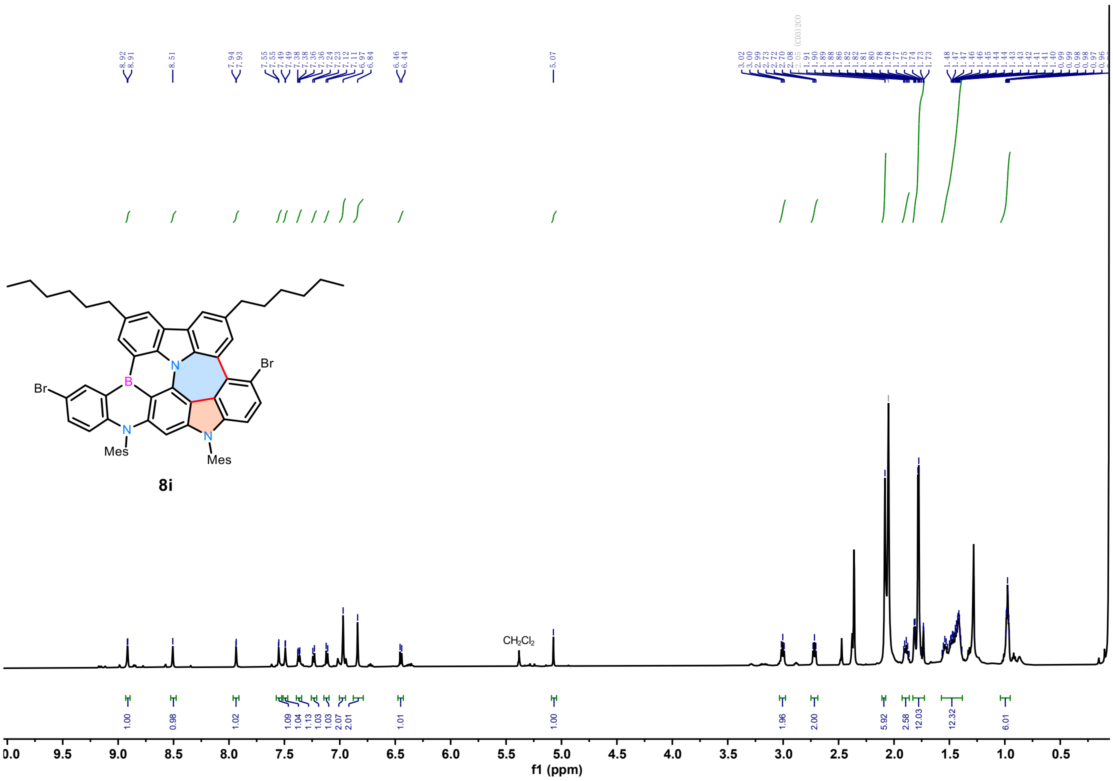

^{13}C NMR spectrum (151 MHz, Acetone-*d6*/CS_2, 298 K) of compound **8i**

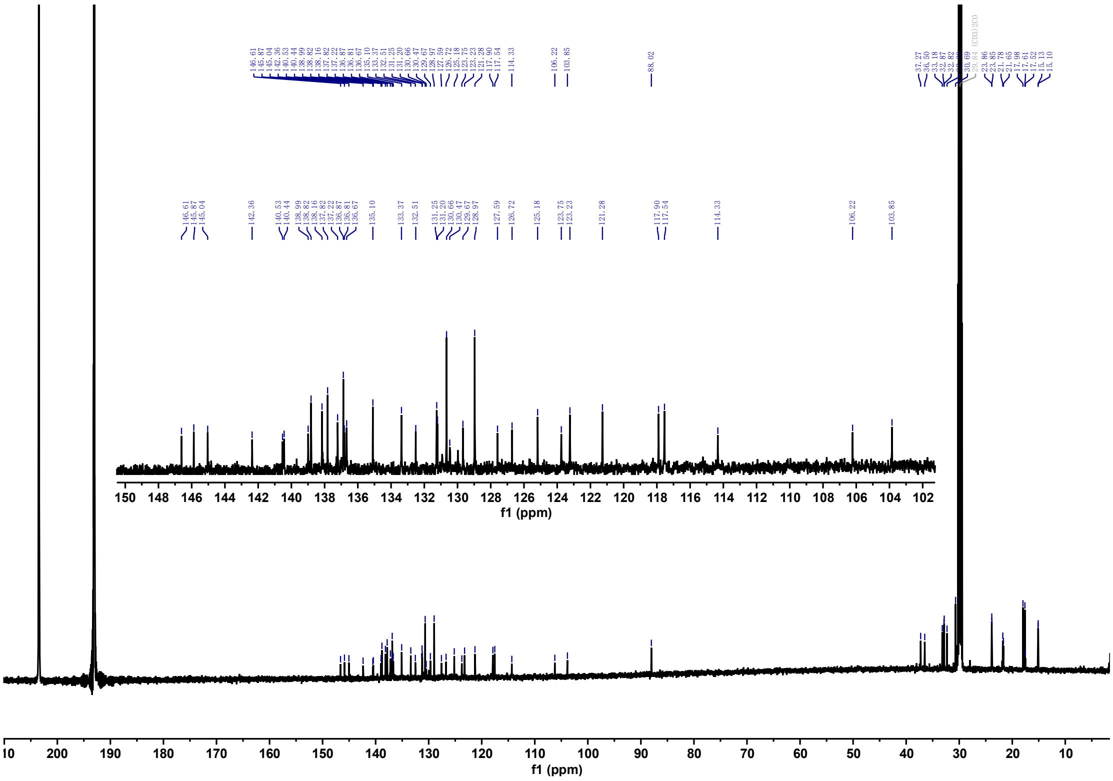

^{1}H NMR spectrum (600 MHz, Acetone-*d6*/CS_2, 298 K) of compound **8j**

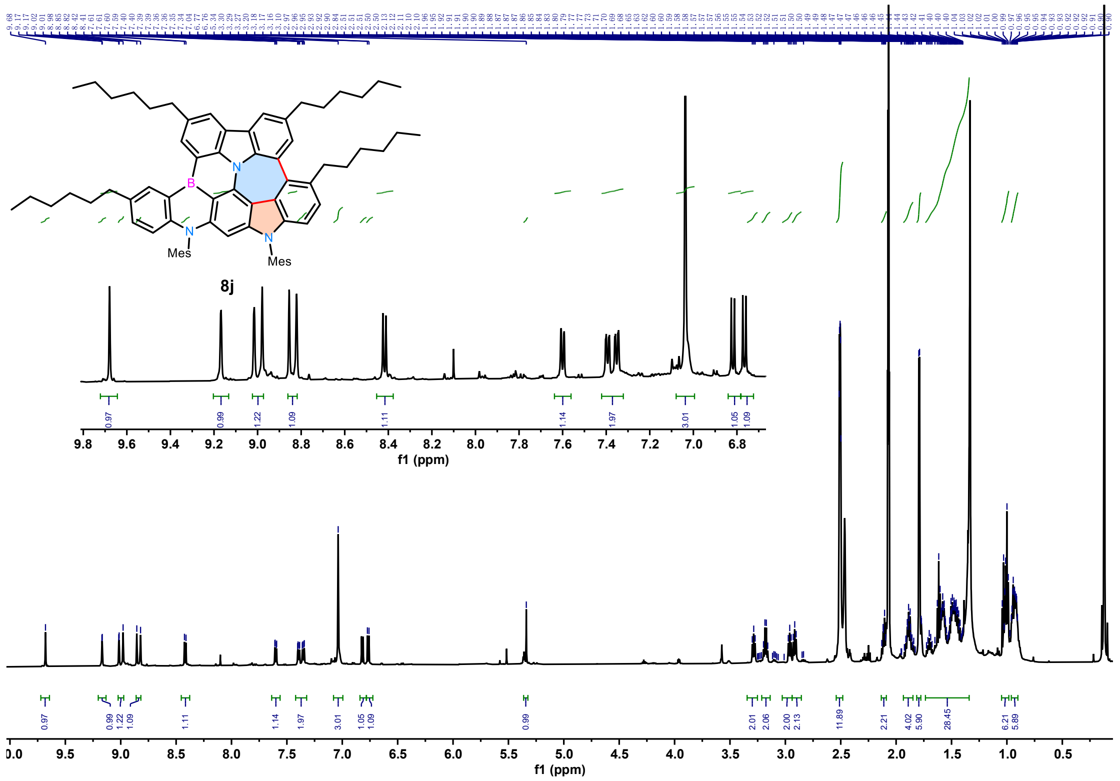

^{13}C NMR spectrum (151 MHz, Acetone-*d6*/CS_2, 298 K) of compound **8j**

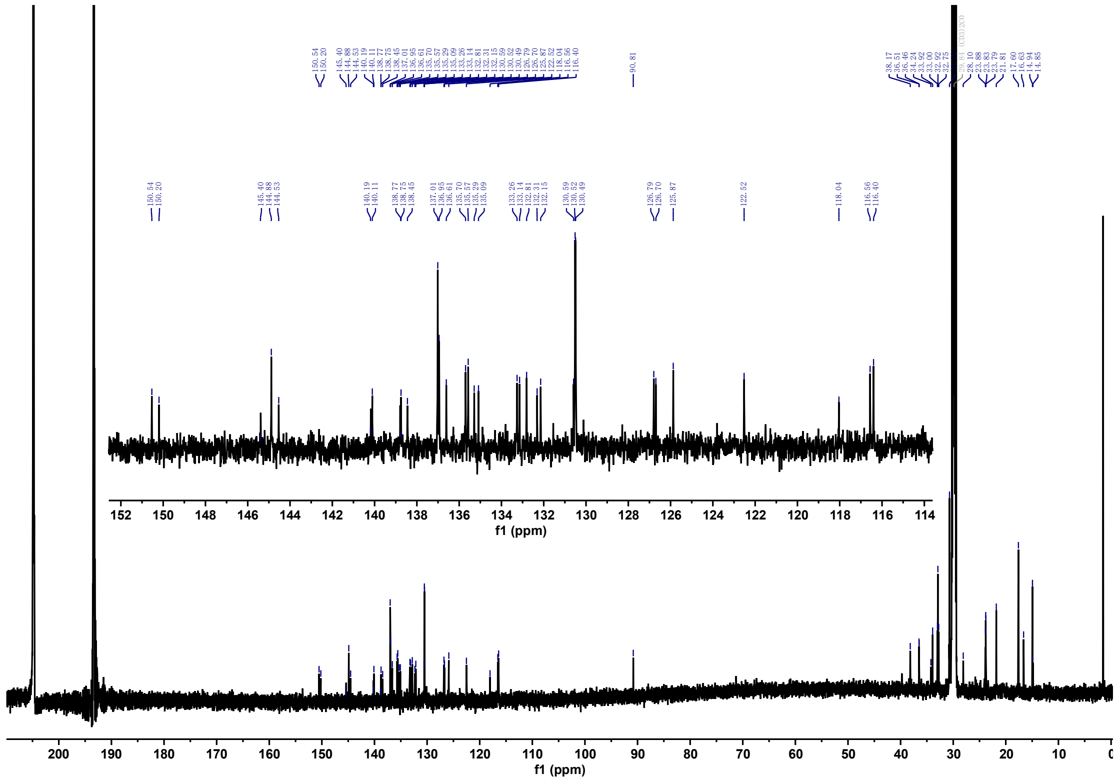

^{1}H NMR spectrum (400 MHz, $CDCl_3$, 298 K) of compound **8k**

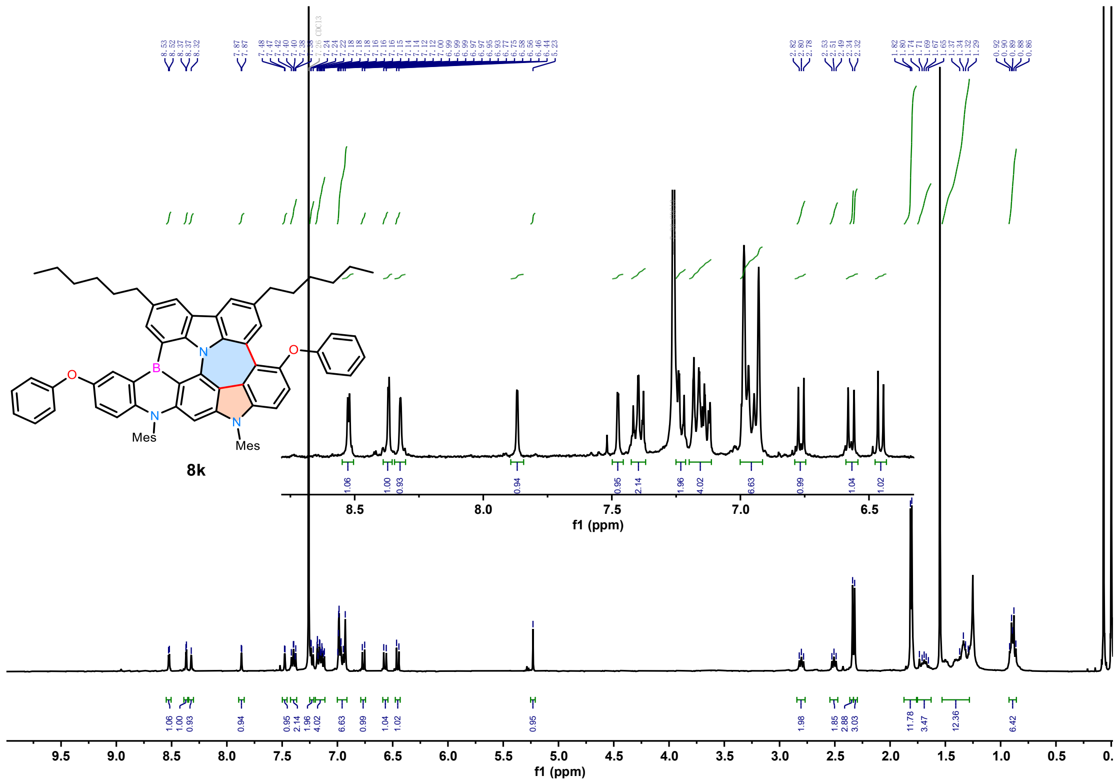

^{13}C NMR spectrum (151 MHz, $CDCl_3$, 298 K) of compound **8k**

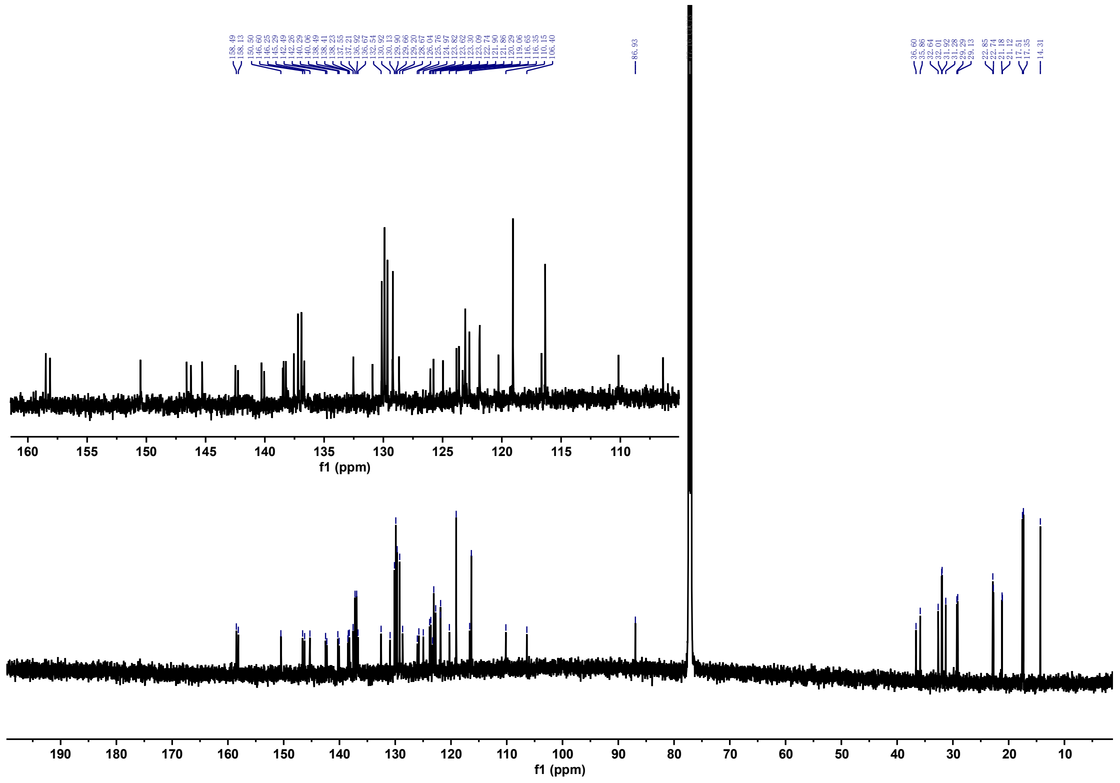

^{1}H NMR spectrum (400 MHz, $CDCl_3$, 298 K) of compound **8l**

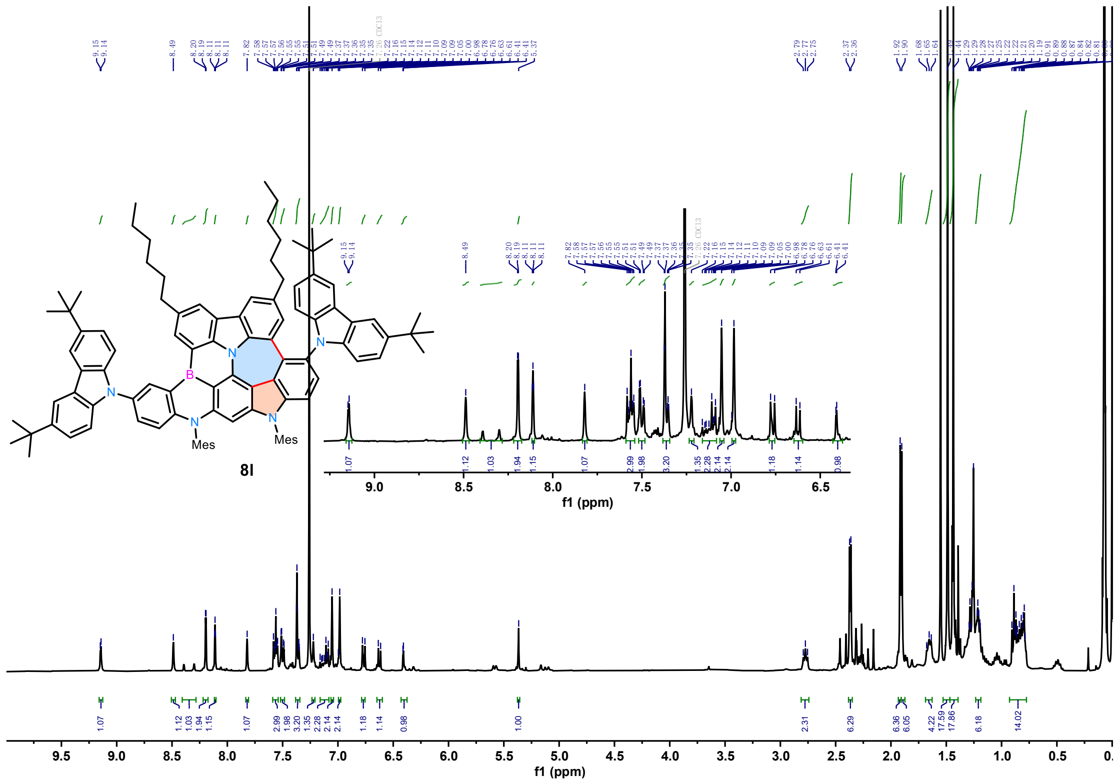

^{13}C NMR spectrum (151 MHz, Acetone-*d6*/CS_2, 298 K) of compound **8l**

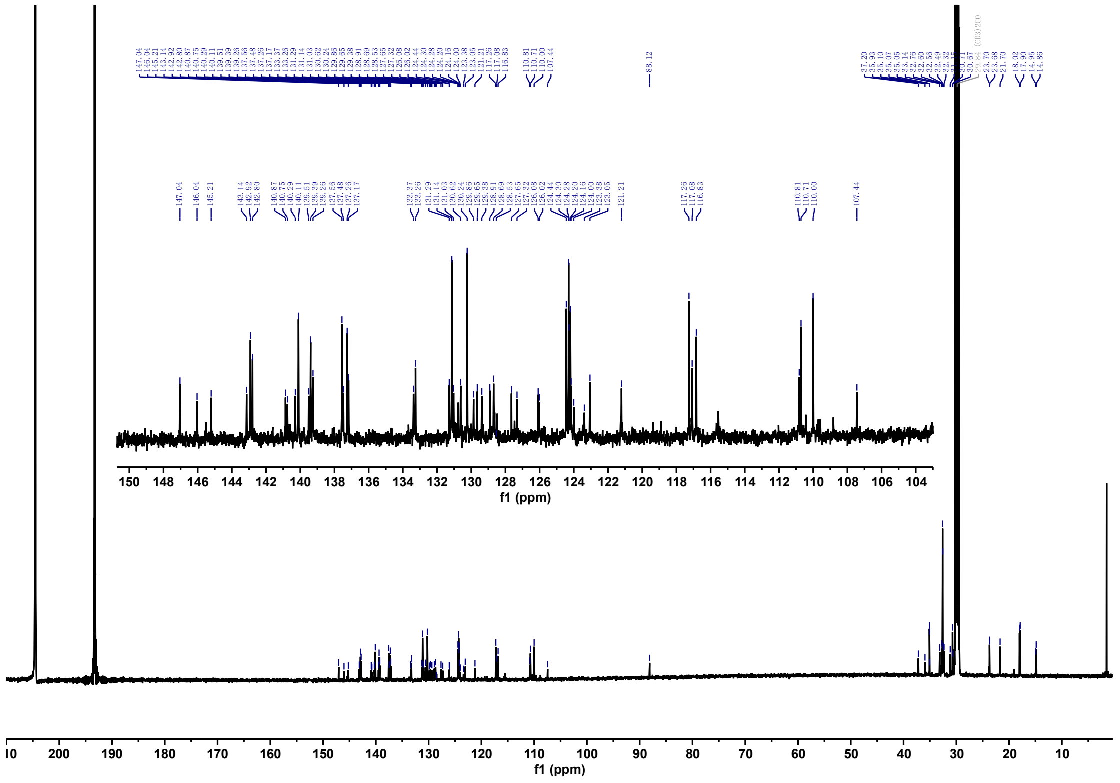

^{1}H NMR spectrum (600 MHz, DMSO-*d6*, 298 K) of compound **8m**

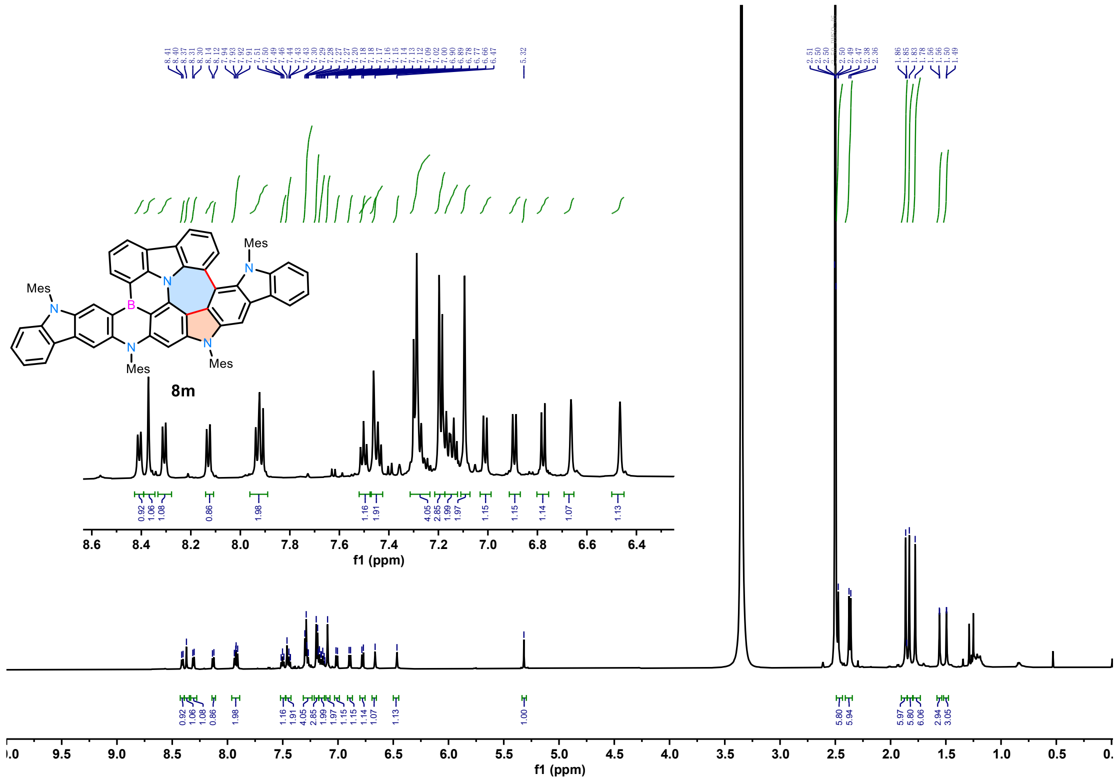

^{13}C NMR spectrum (151 MHz, DMSO-*d6*, 298 K) of compound **8m**

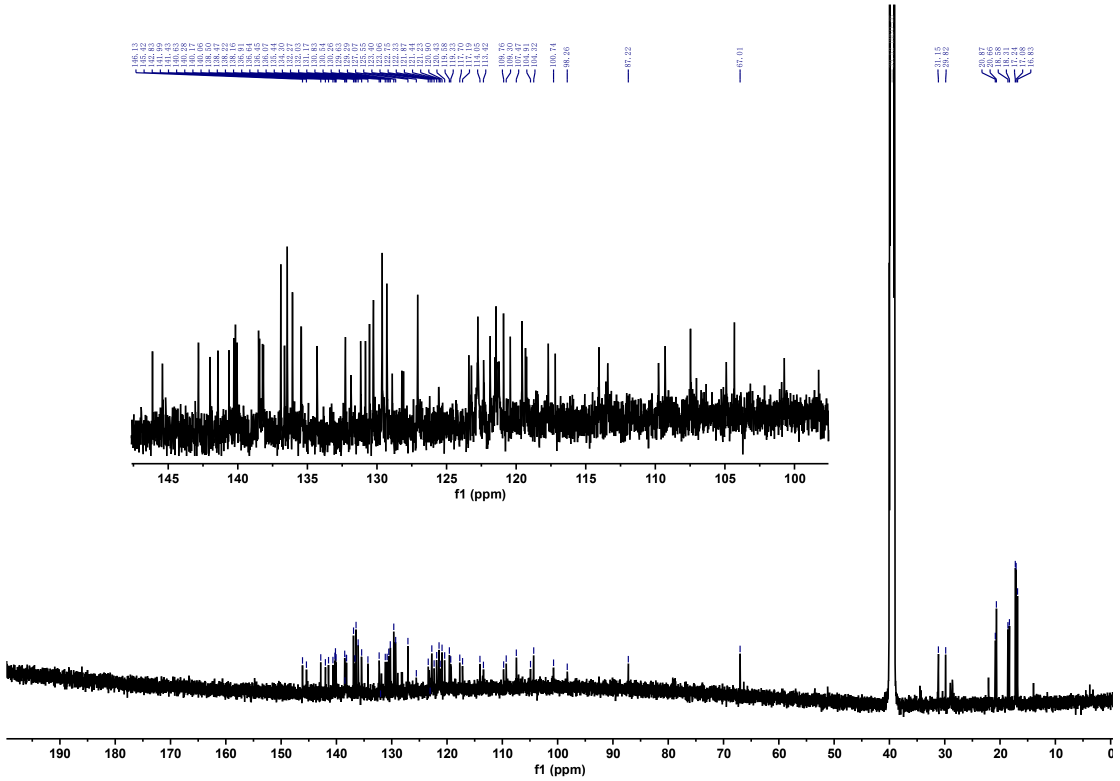

^{1}H NMR spectrum (400 MHz, $CDCl_3$, 298 K) of compound **8n**

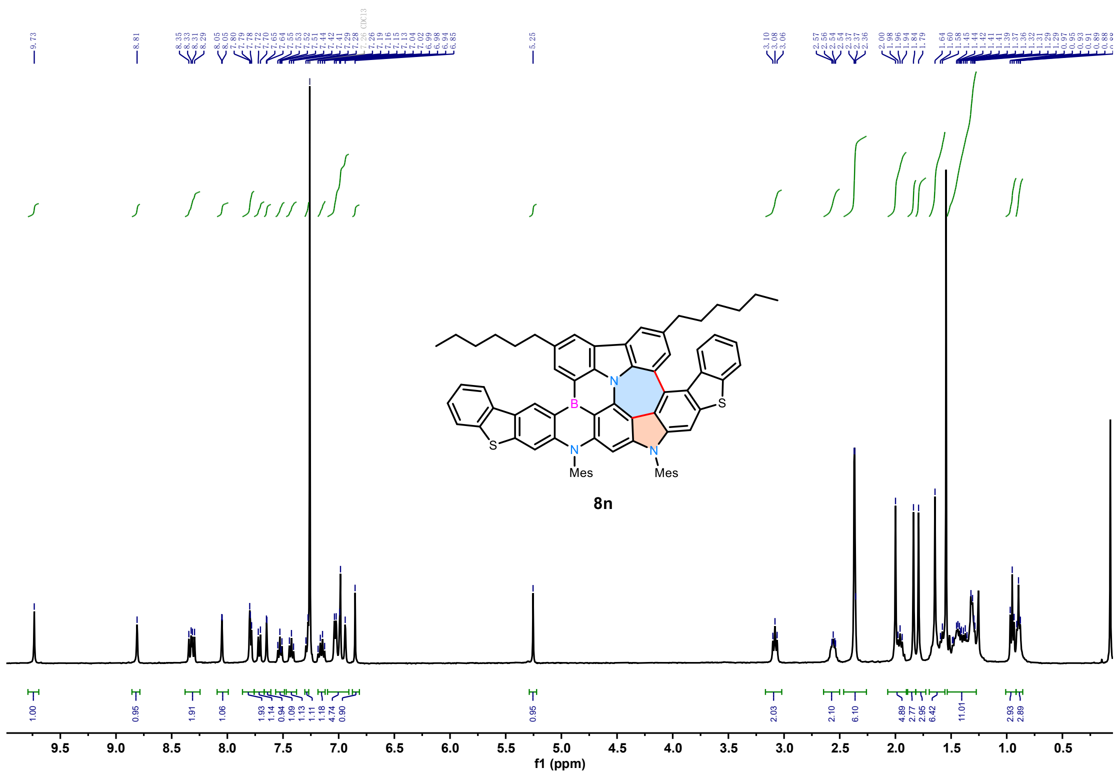

^{13}C NMR spectrum (101 MHz, $CDCl_3$, 298 K) of compound **8n**

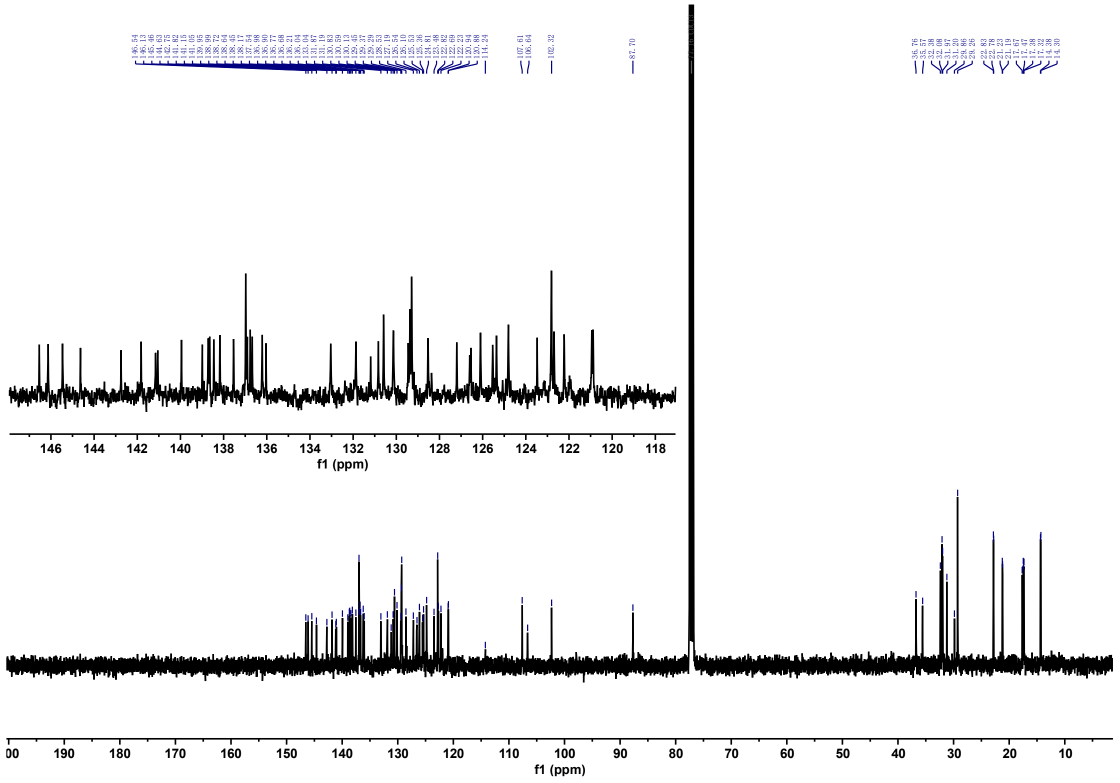

^{1}H NMR spectrum (400 MHz, $CDCl_3$, 298 K) of compound **8o**

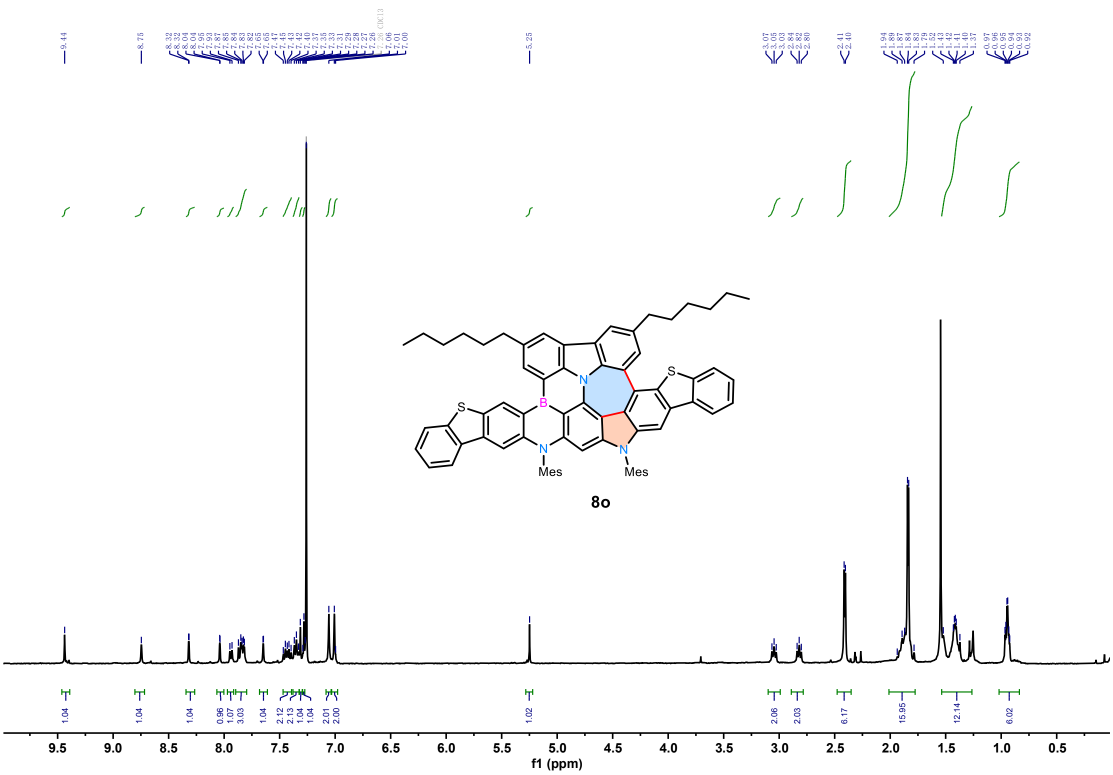

^{13}C NMR spectrum (101 MHz, $CDCl_3$, 298 K) of compound **8o**

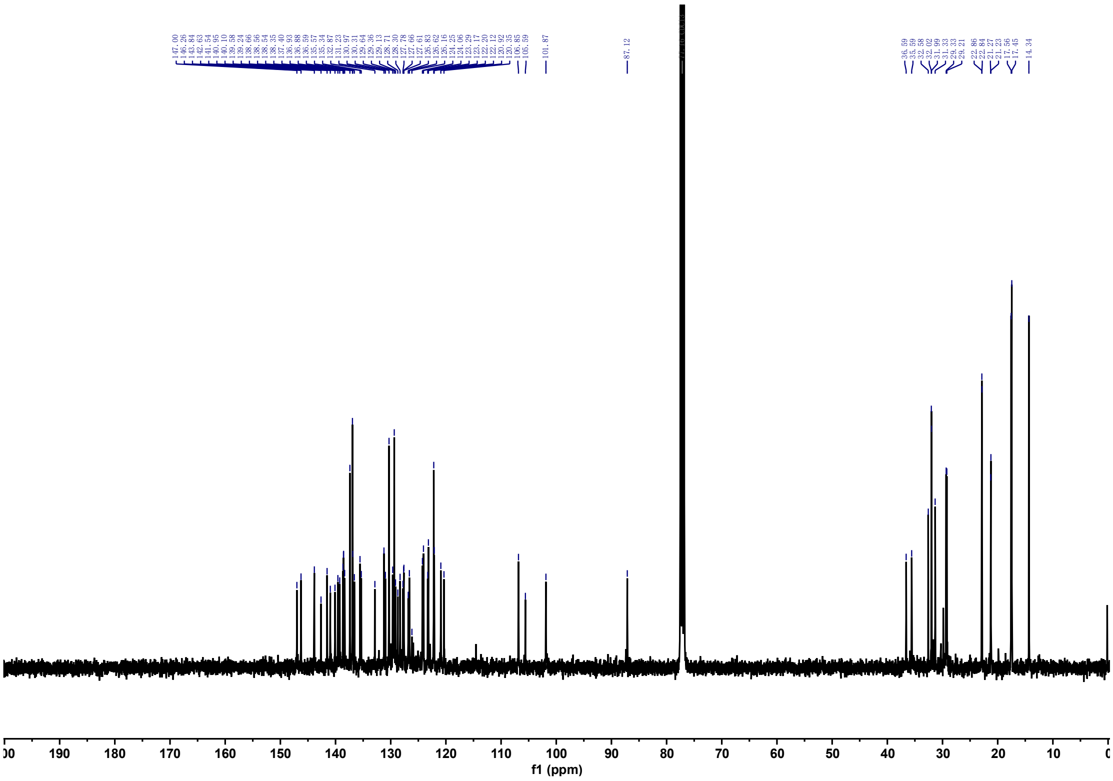

^{1}H NMR spectrum (600 MHz, $CDCl_3$, 298 K) of compound **8p**

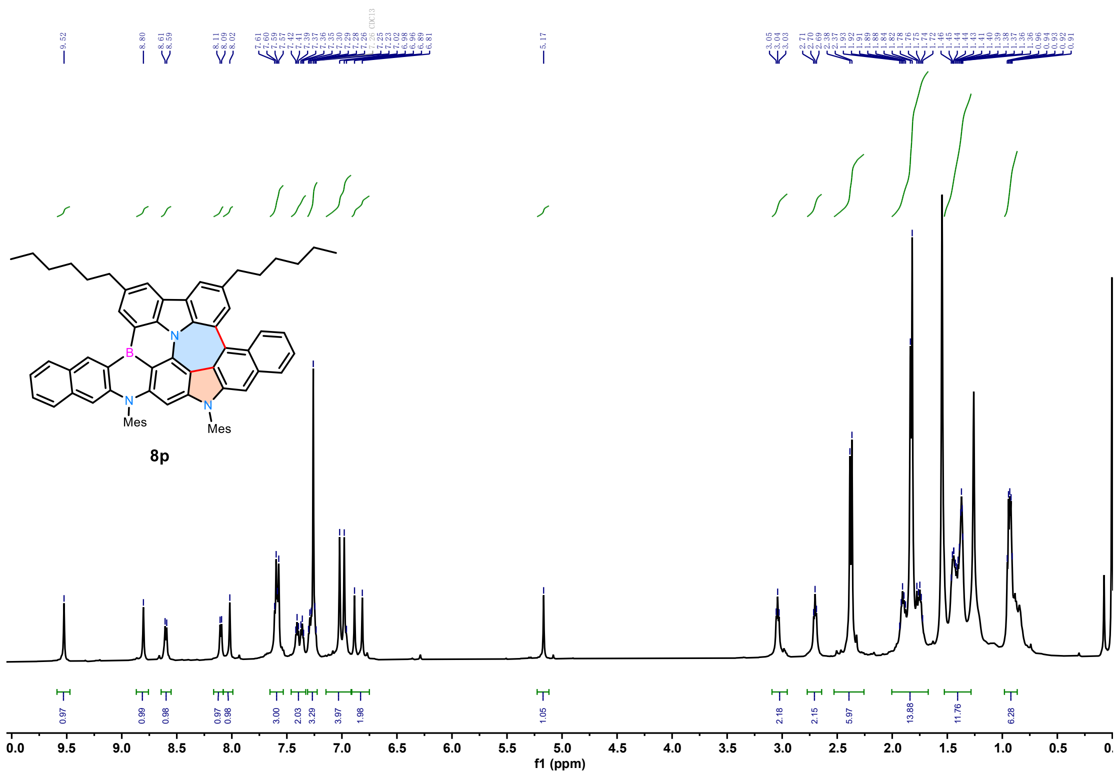

^{13}C NMR spectrum (151 MHz, $CDCl_3$, 298 K) of compound **8p**

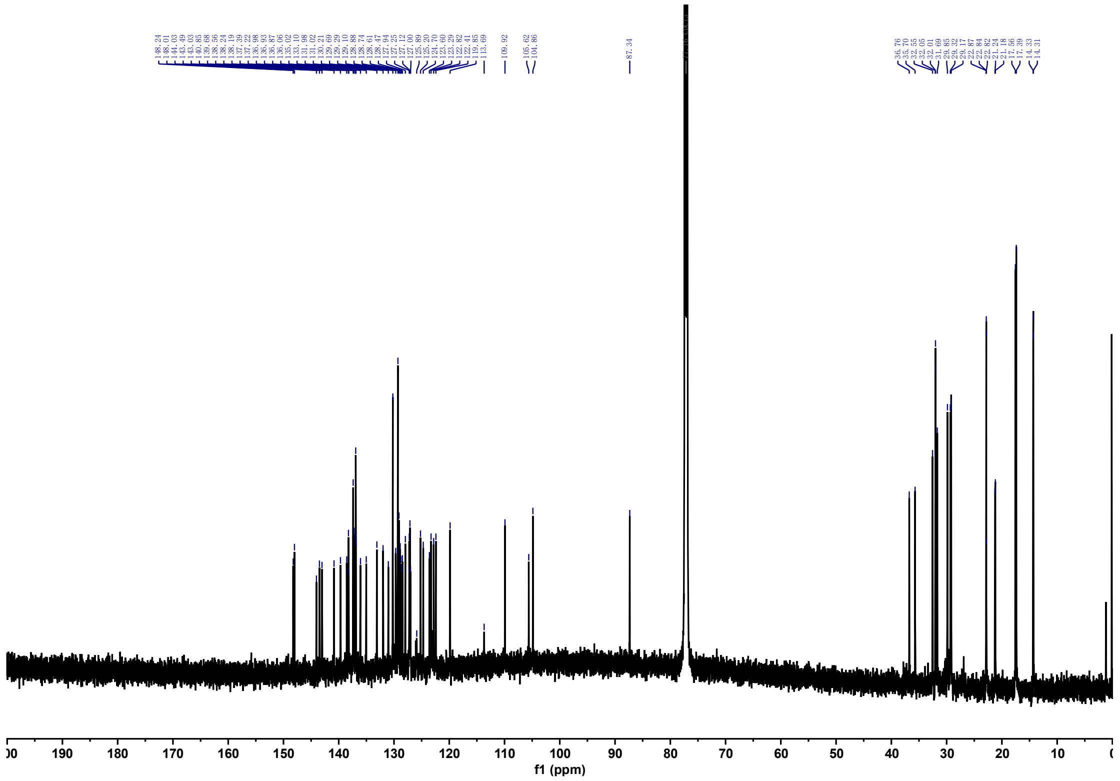

^{1}H NMR spectrum (400 MHz, $CDCl_3$, 298 K) of compound **9a**

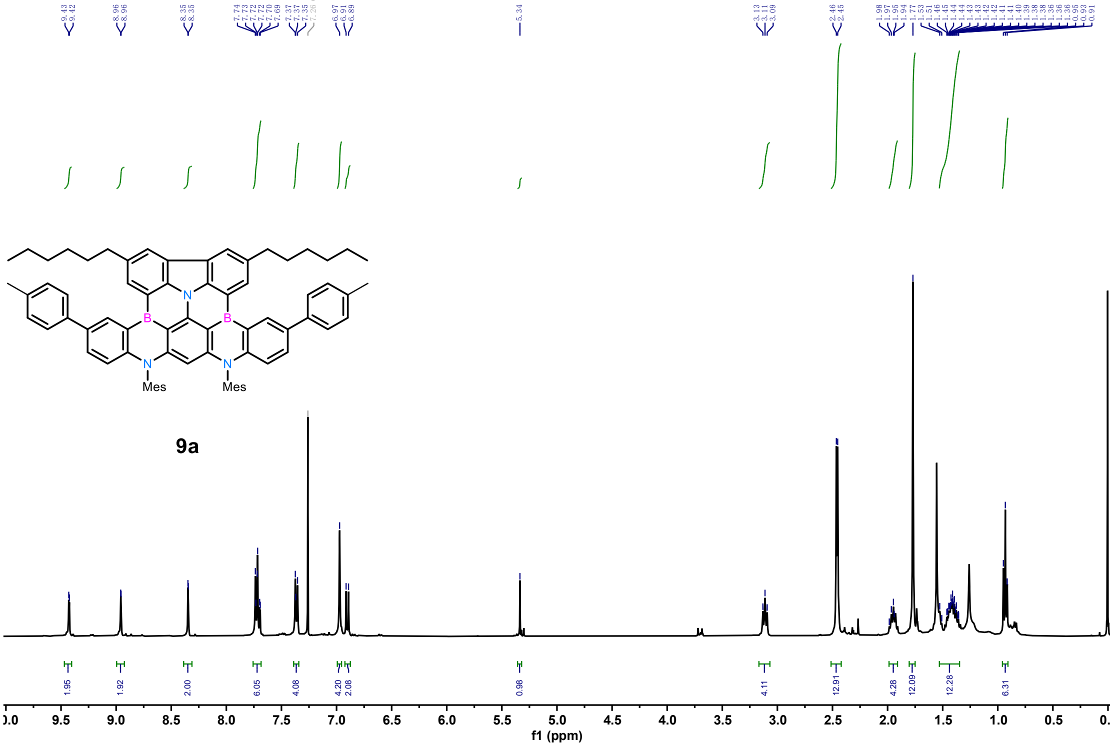

^{13}C NMR spectrum (101 MHz, $CDCl_3$, 298 K) of compound **9a**

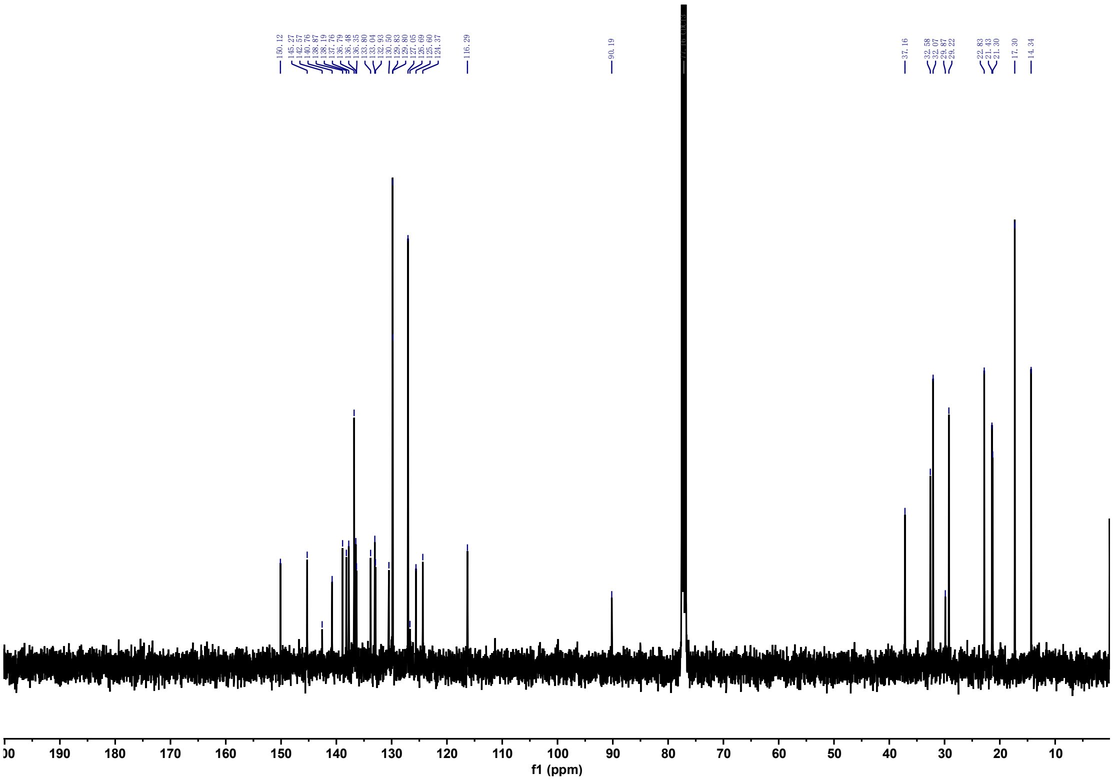

^{1}H NMR spectrum (400 MHz, $CDCl_3$, 298 K) of compound **9b**

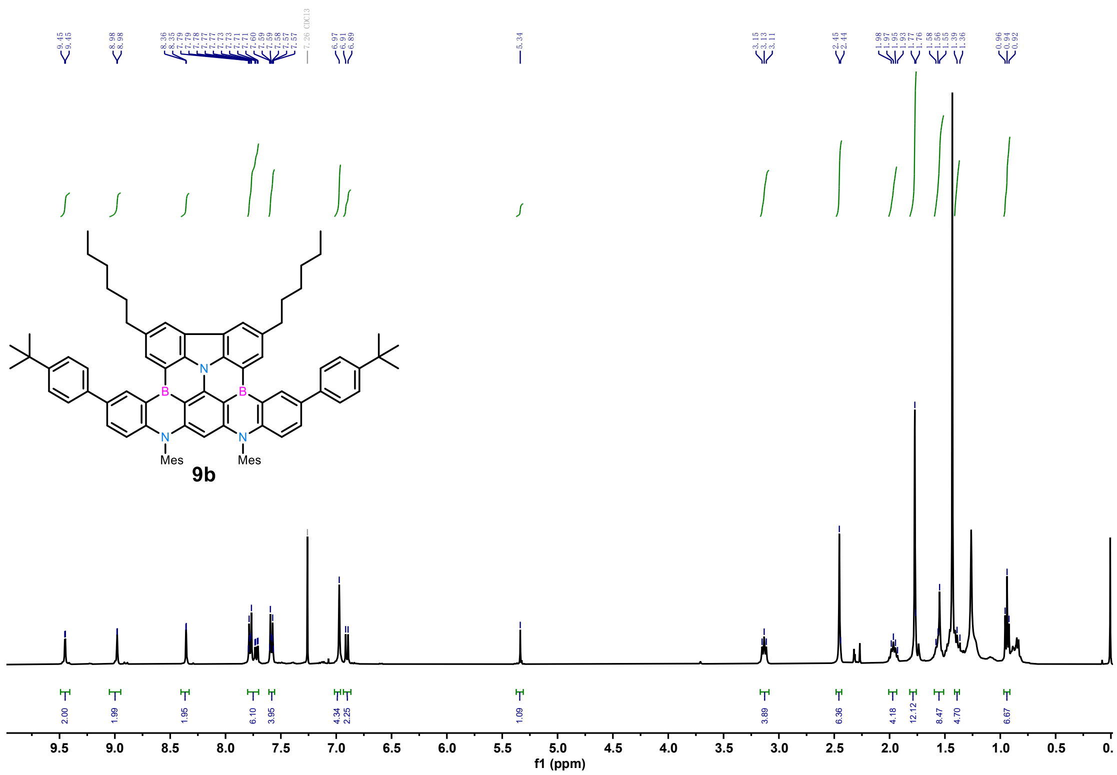

^{13}C NMR spectrum (101 MHz, $CDCl_3$, 298 K) of compound **9b**

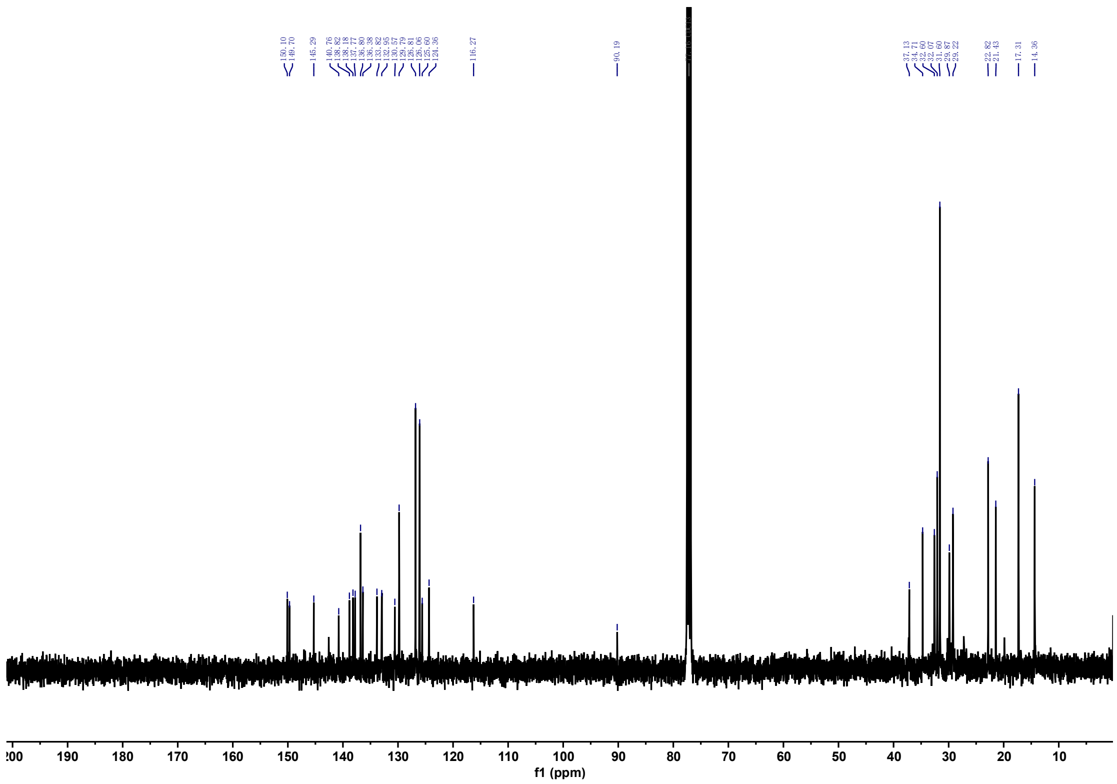

^{1}H NMR spectrum (400 MHz, $CDCl_3$, 298 K) of compound **9c**

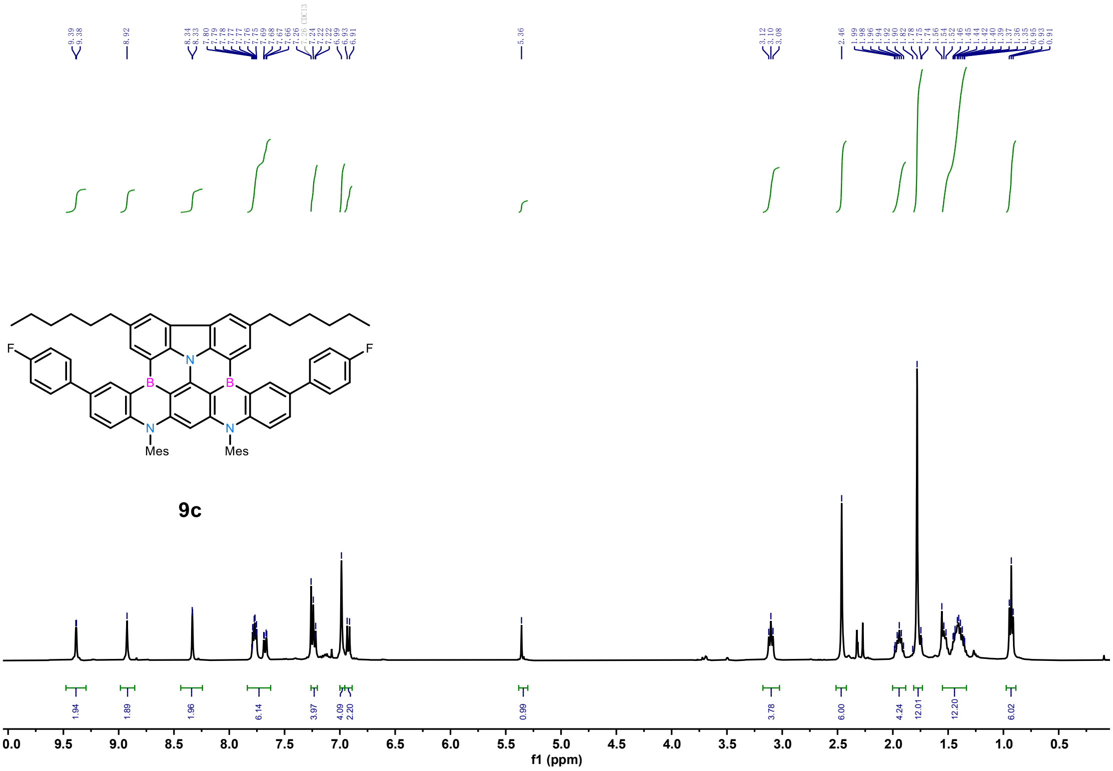

^{13}C NMR spectrum (101 MHz, $CDCl_3$, 298 K) of compound **9c**

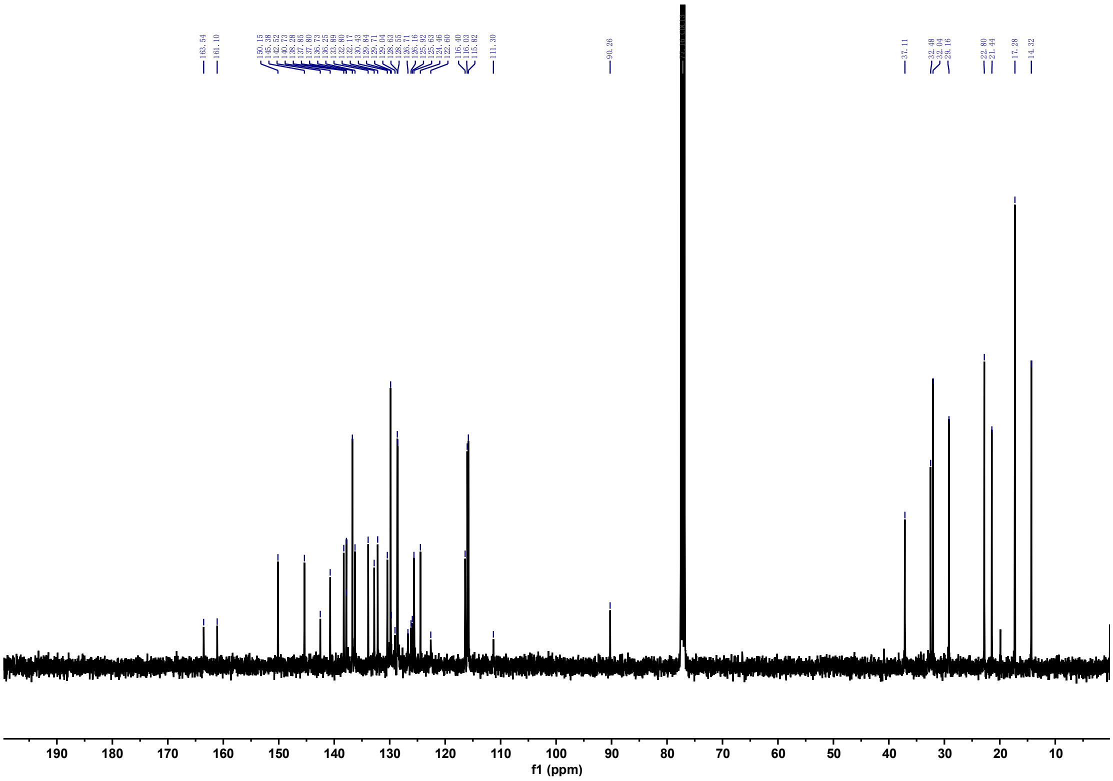

^{1}H NMR spectrum (400 MHz, $CDCl_3$, 298 K) of compound **9d**

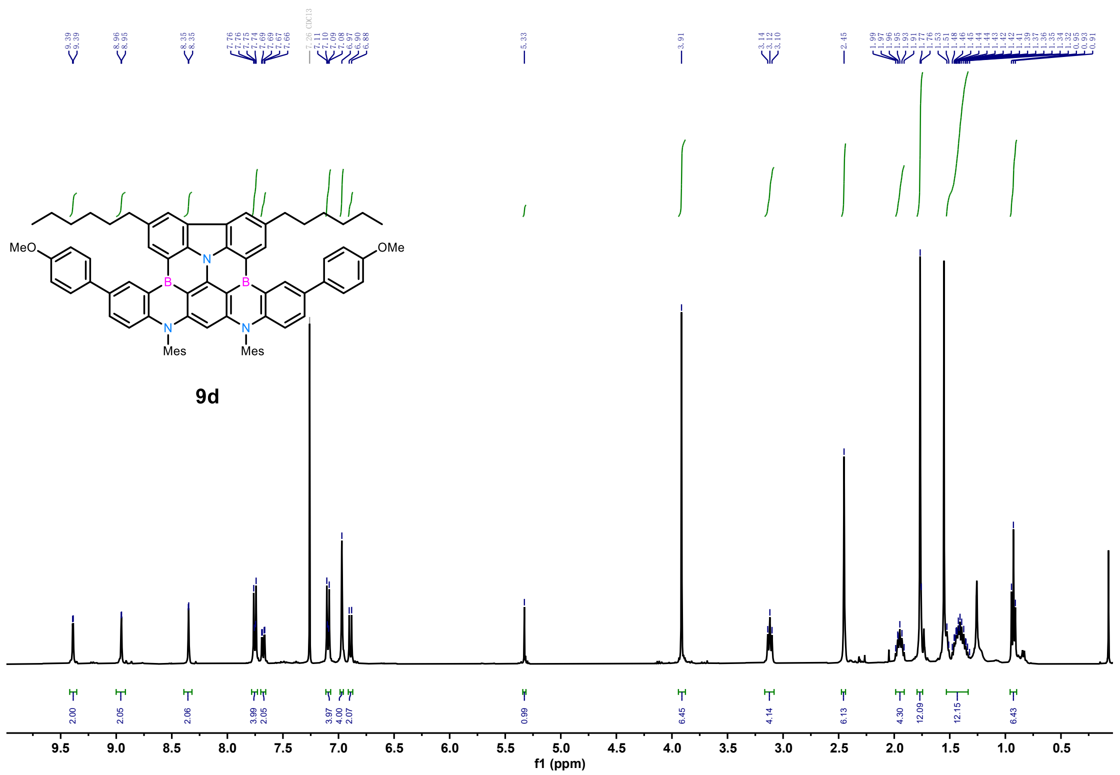

^{13}C NMR spectrum (101 MHz, $CDCl_3$, 298 K) of compound **9d**

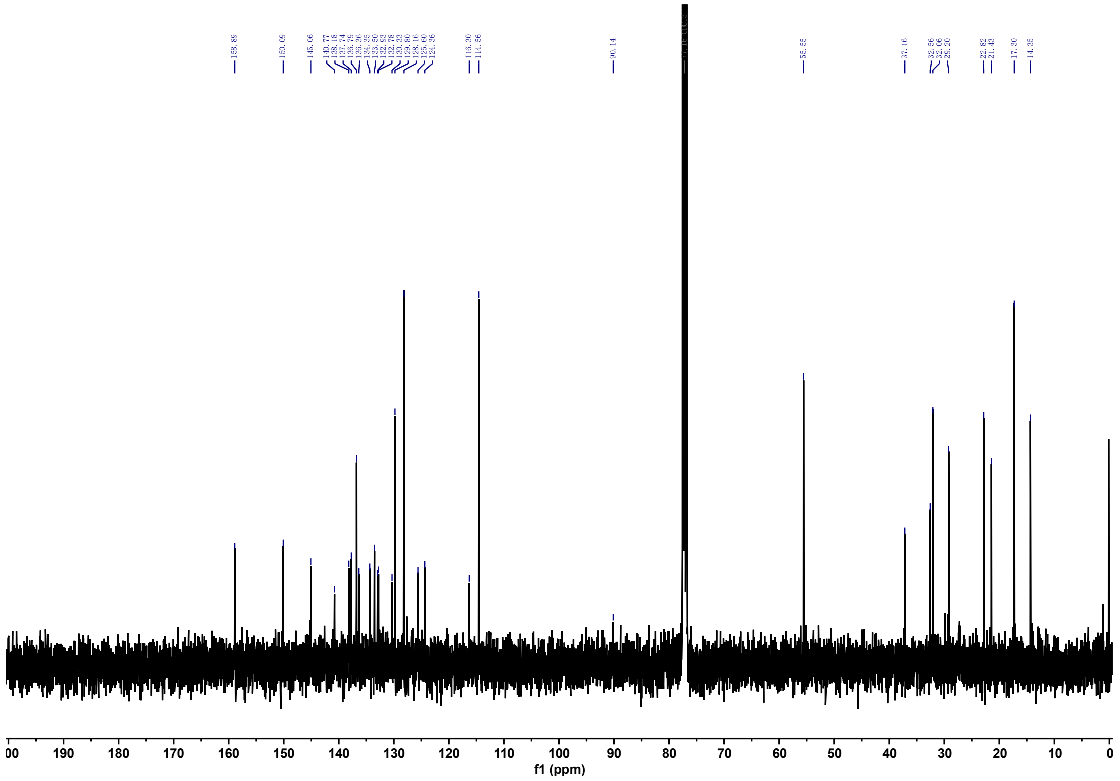

^{1}H NMR spectrum (400 MHz, $CDCl_3$, 298 K) of compound **9e**

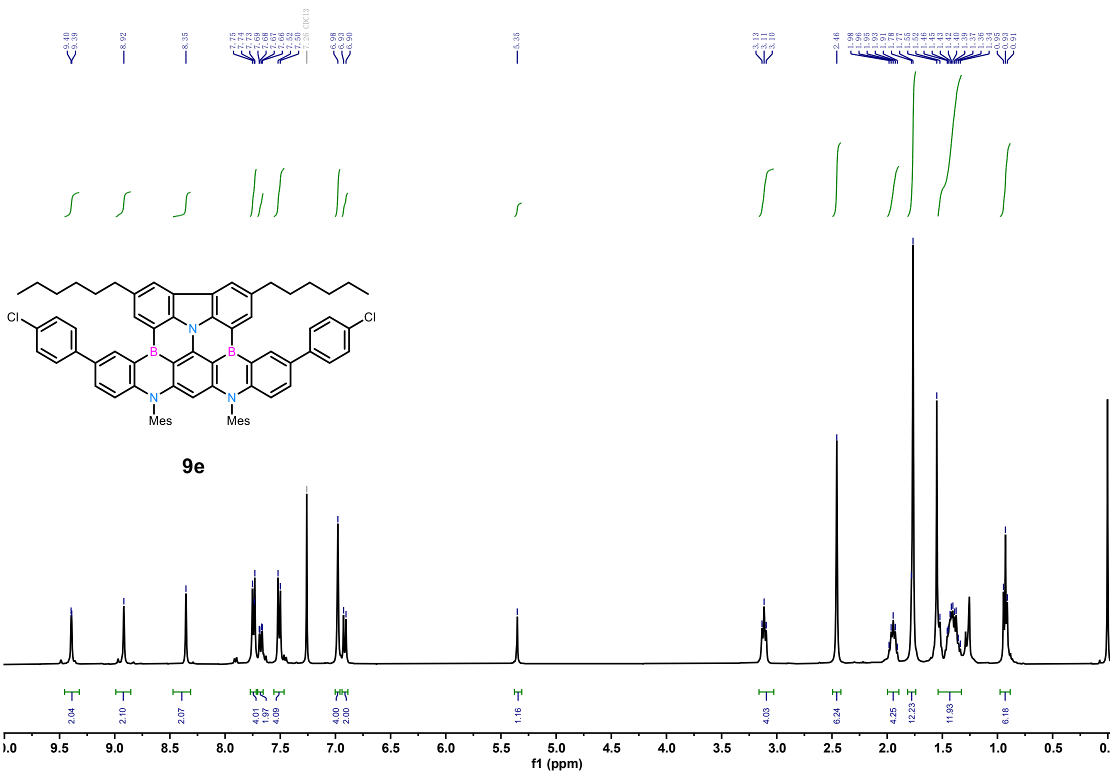

^{13}C NMR spectrum (101 MHz, $CDCl_3$, 298 K) of compound **9e**

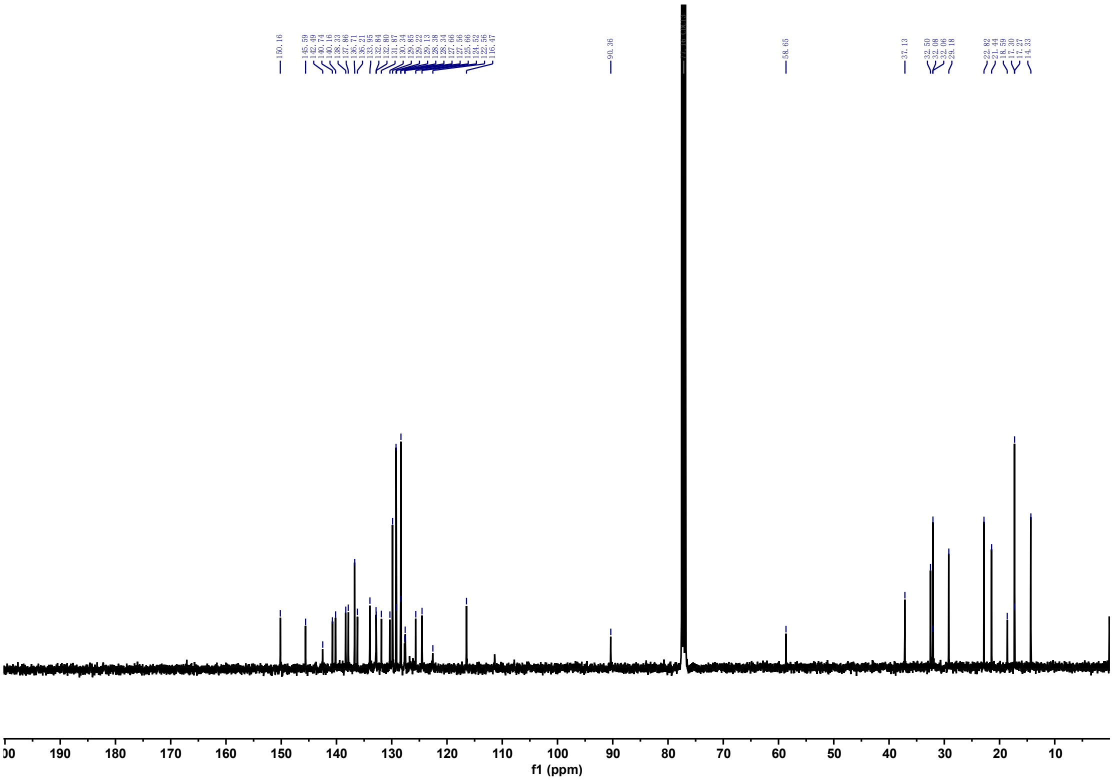

^{1}H NMR spectrum (400 MHz, $CDCl_3$, 298 K) of compound **9f**

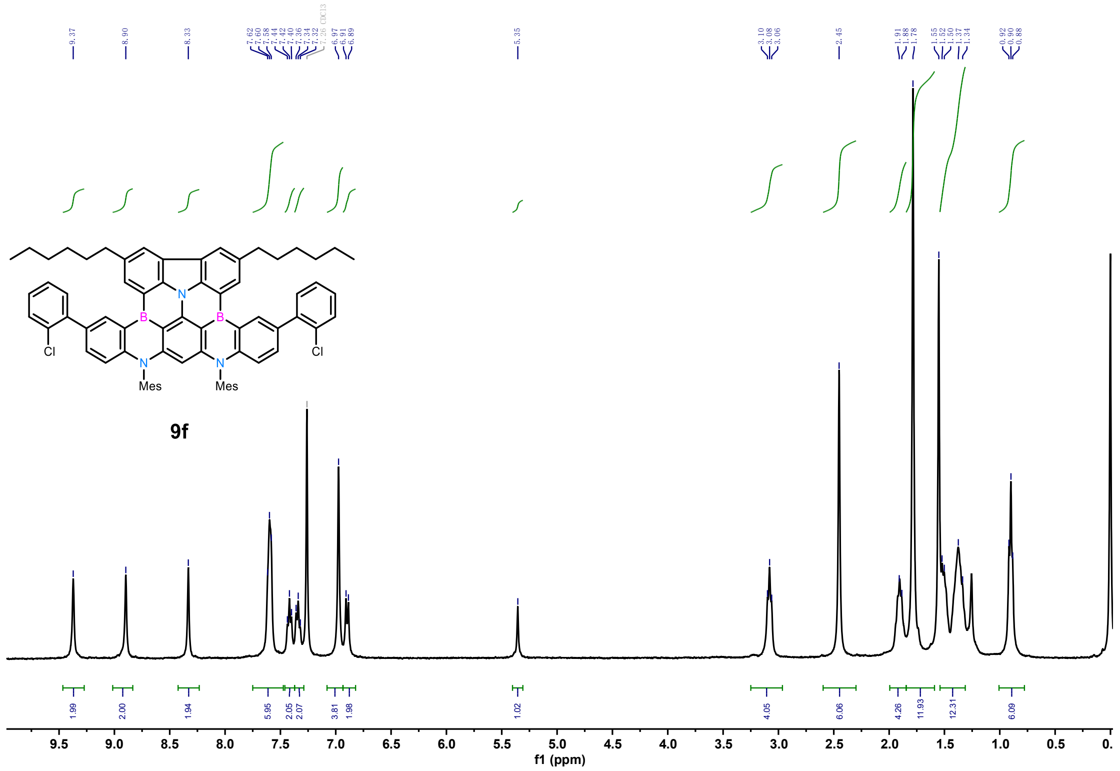

^{13}C NMR spectrum (101 MHz, $CDCl_3$, 298 K) of compound **9f**

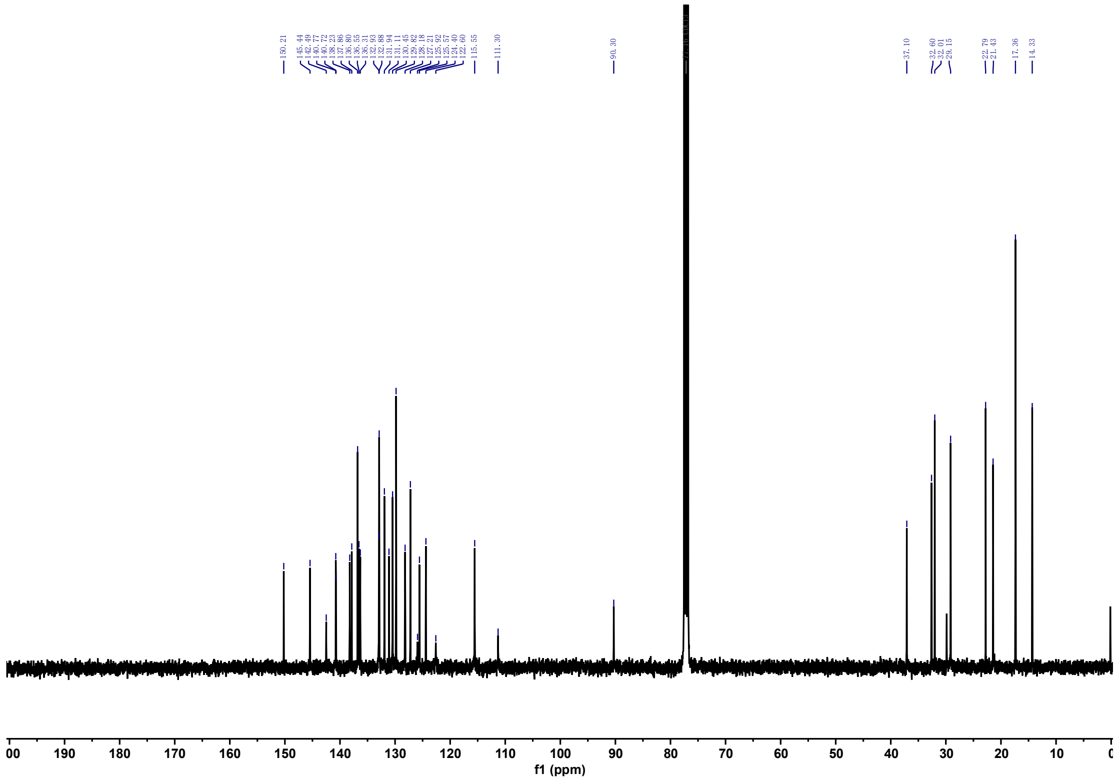

^{1}H NMR spectrum (400 MHz, $CDCl_3$, 298 K) of compound **9g**

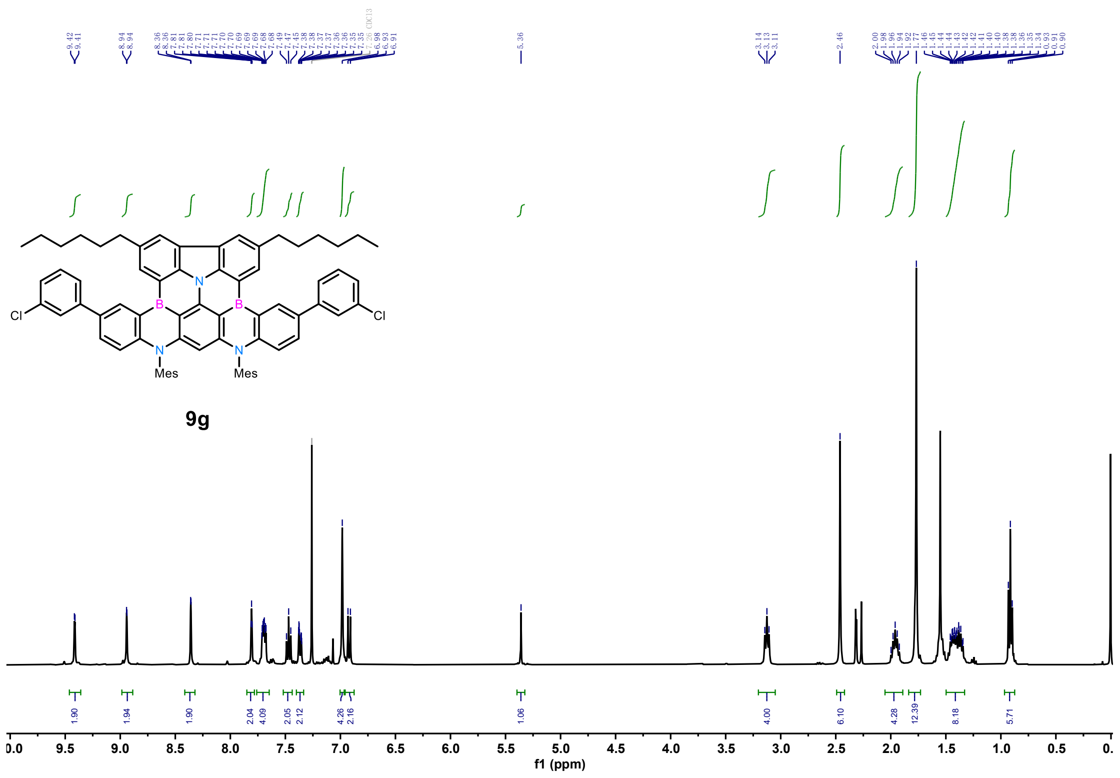

^{13}C NMR spectrum (101 MHz, $CDCl_3$, 298 K) of compound **9g**

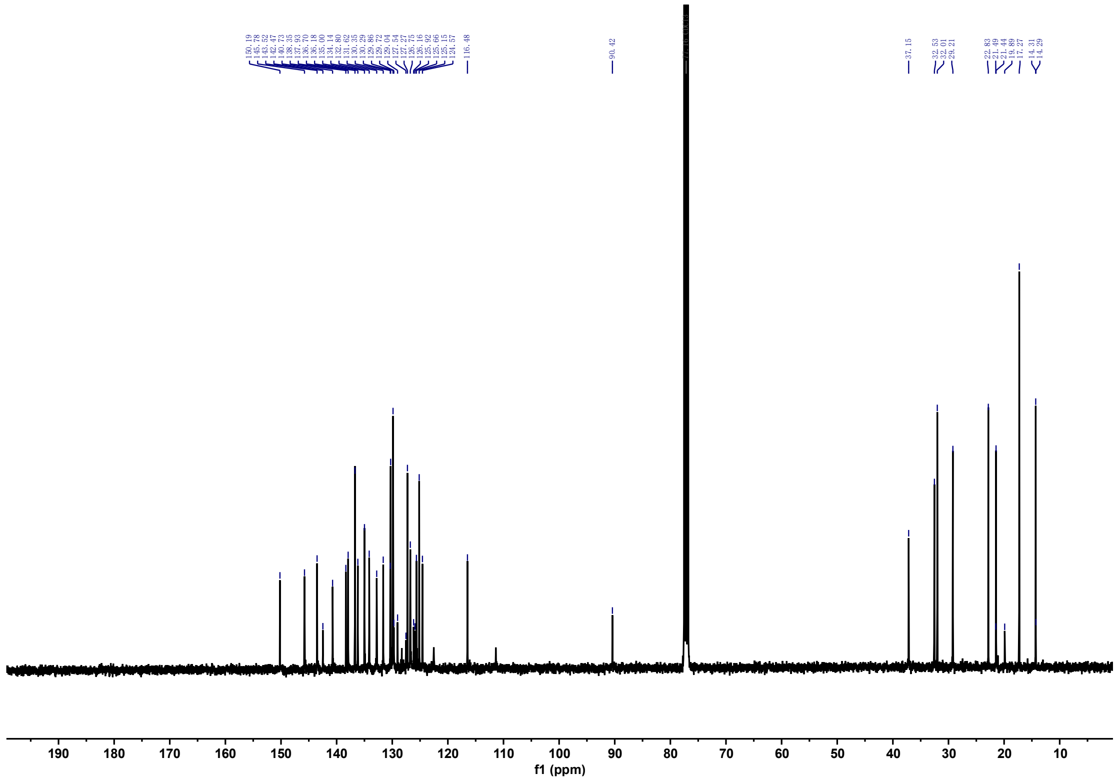

^{1}H NMR spectrum (500 MHz, $CDCl_3$, 298 K) of compound **9h**

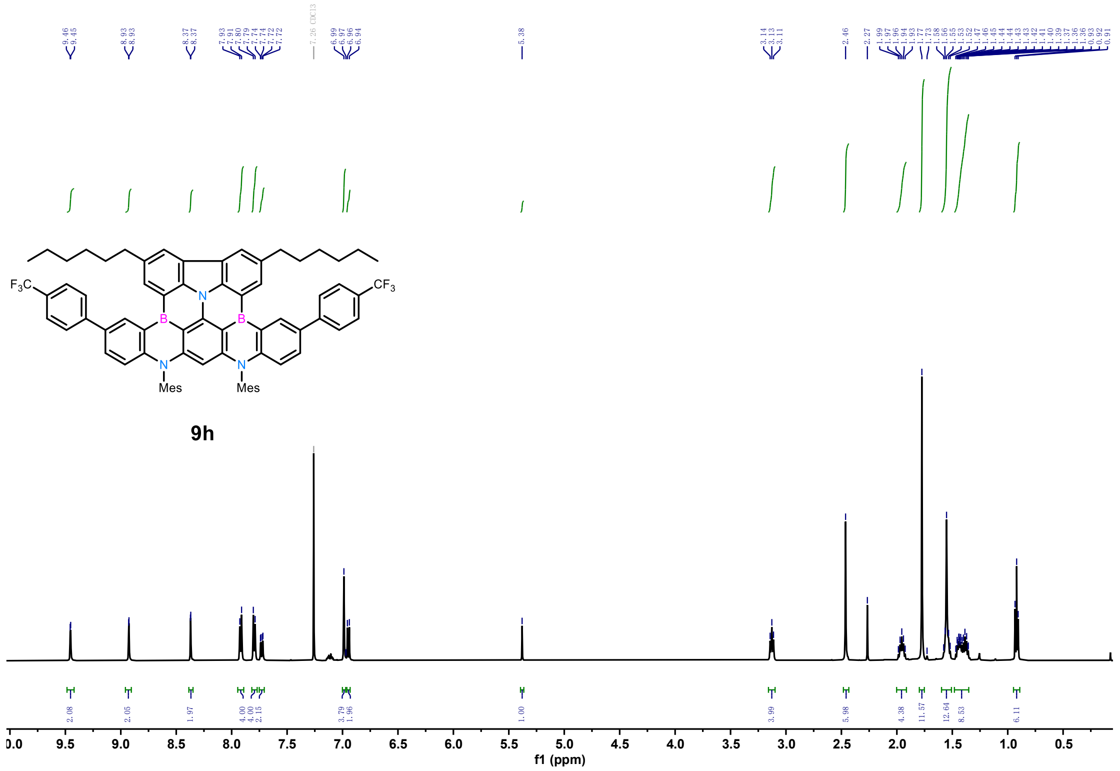

^{13}C NMR spectrum (101 MHz, $CDCl_3$, 298 K) of compound **9h**

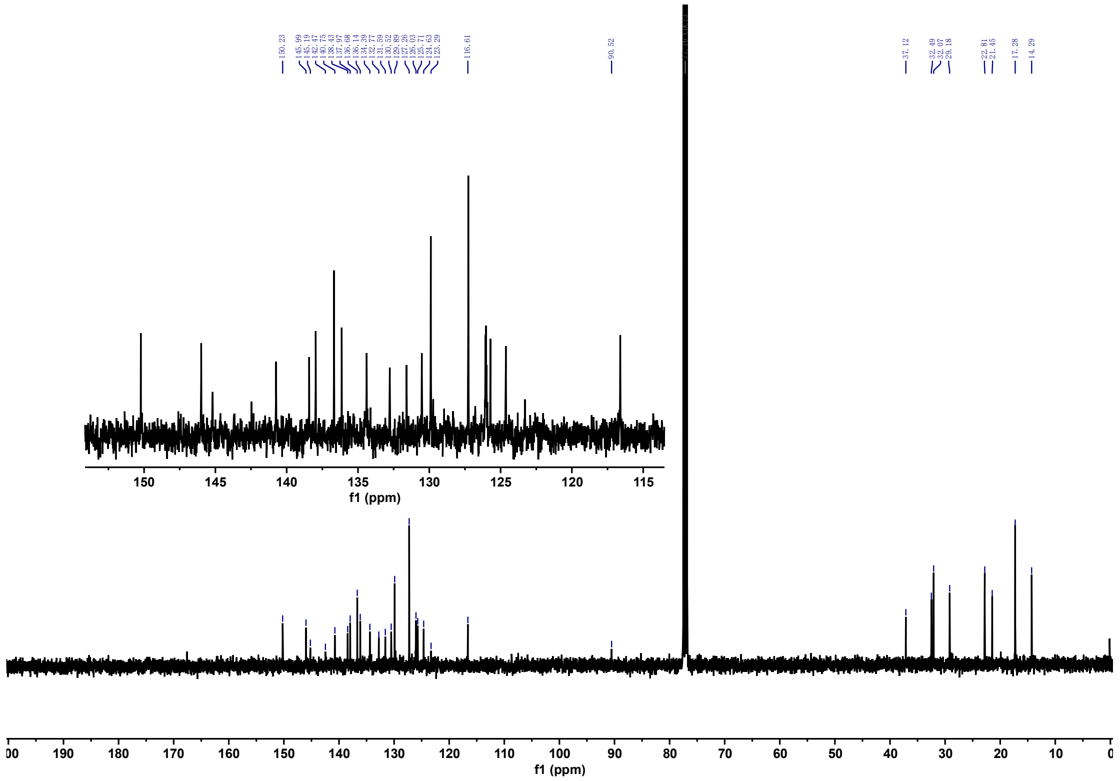

^{1}H NMR spectrum (400 MHz, $CDCl_3$, 298 K) of compound **9i**

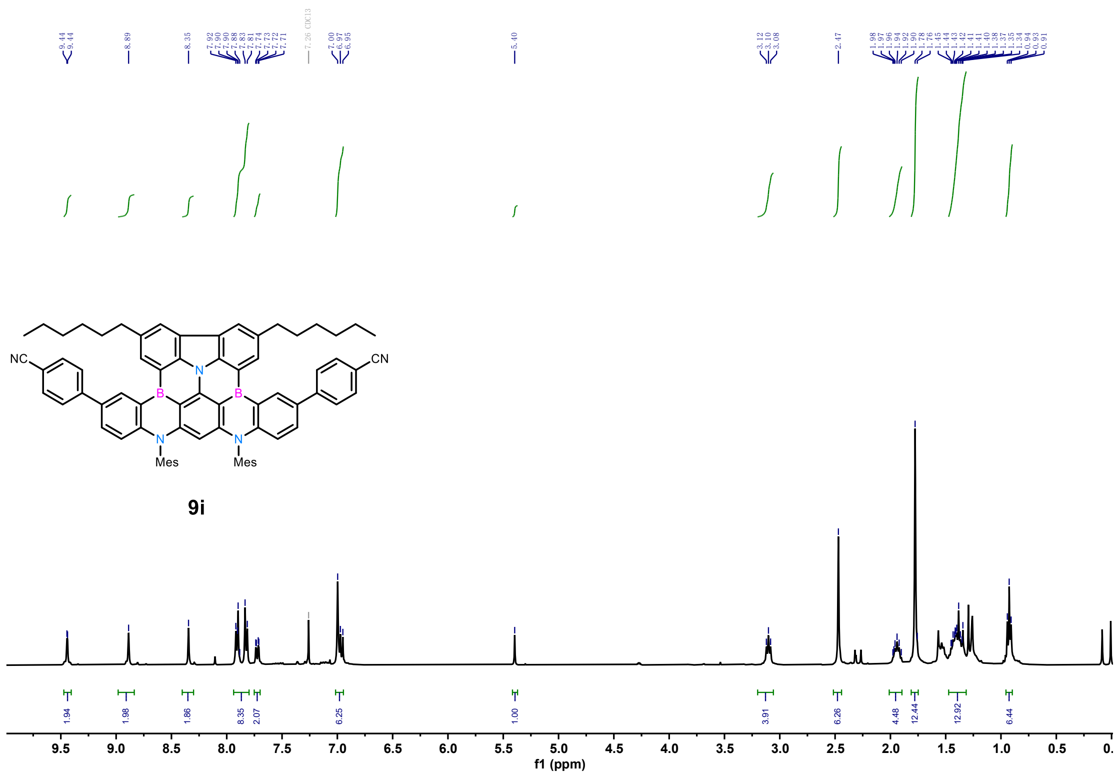

9i

^{13}C NMR spectrum (101 MHz, $CDCl_3$, 298 K) of compound **9i**

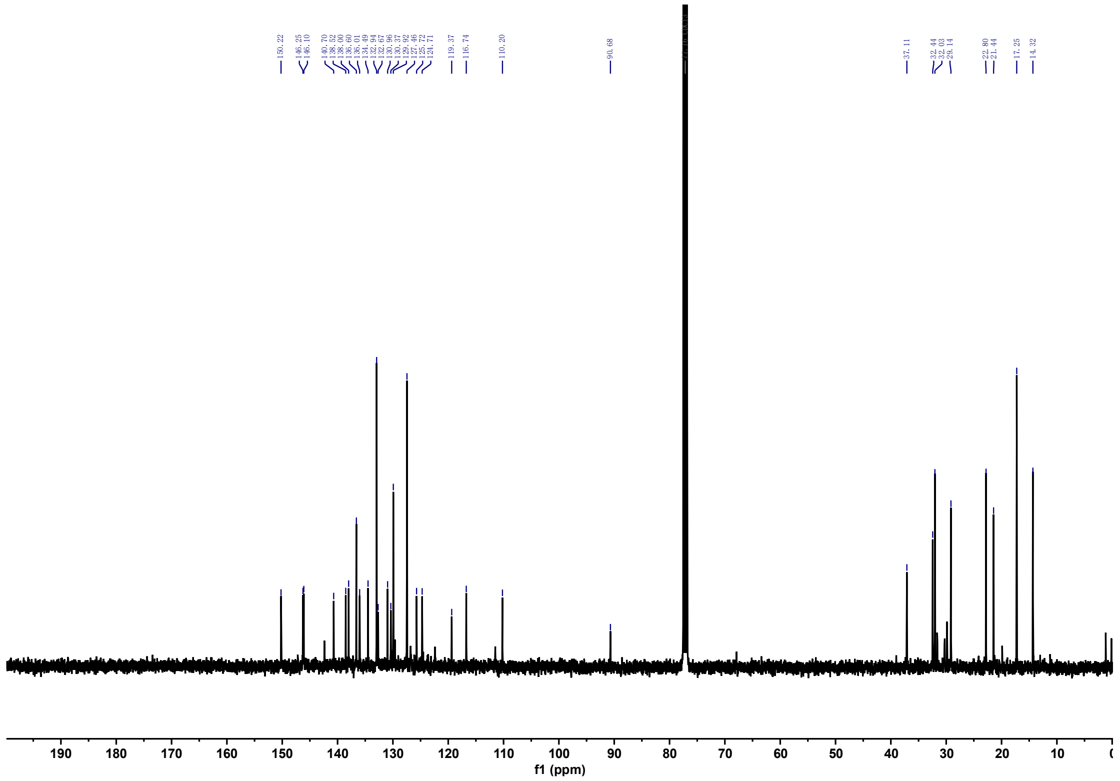

^{1}H NMR spectrum (400 MHz, $CDCl_3$, 298 K) of compound **9j**

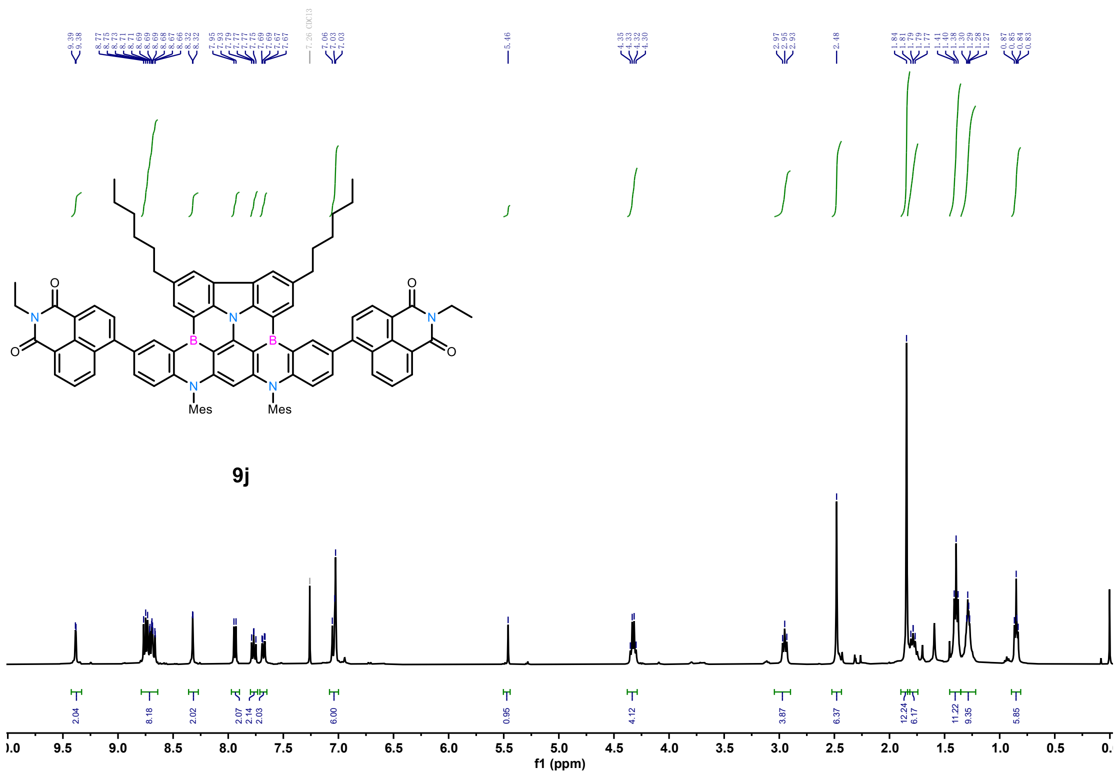

^{13}C NMR spectrum (101 MHz, $CDCl_3$, 298 K) of compound **9j**

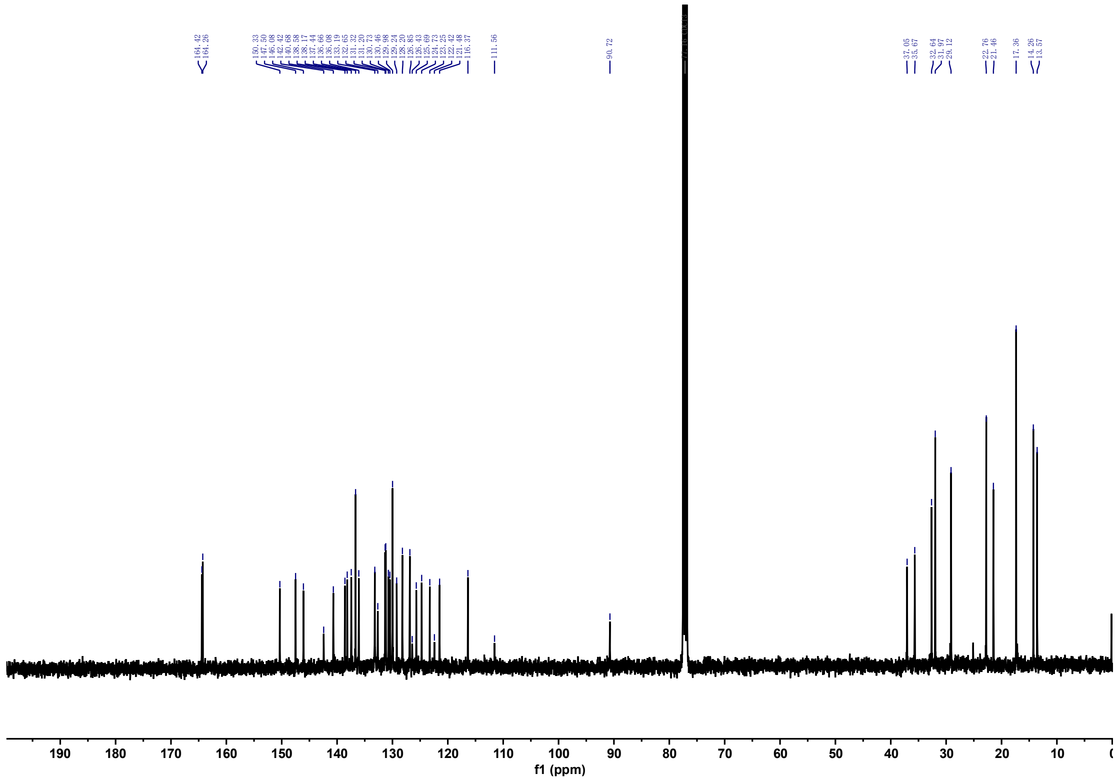

^{1}H NMR spectrum (400 MHz, $CDCl_3$, 298 K) of compound **9k**

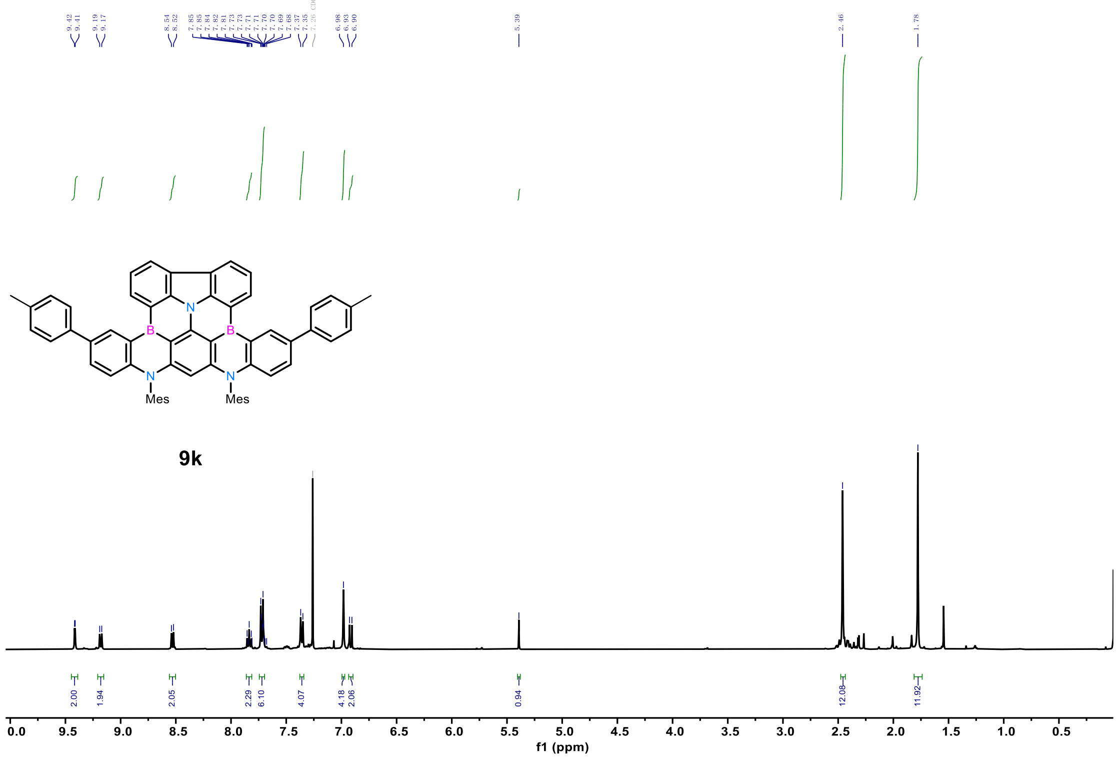

^{13}C NMR spectrum (101 MHz, $CDCl_3$, 298 K) of compound **9k**

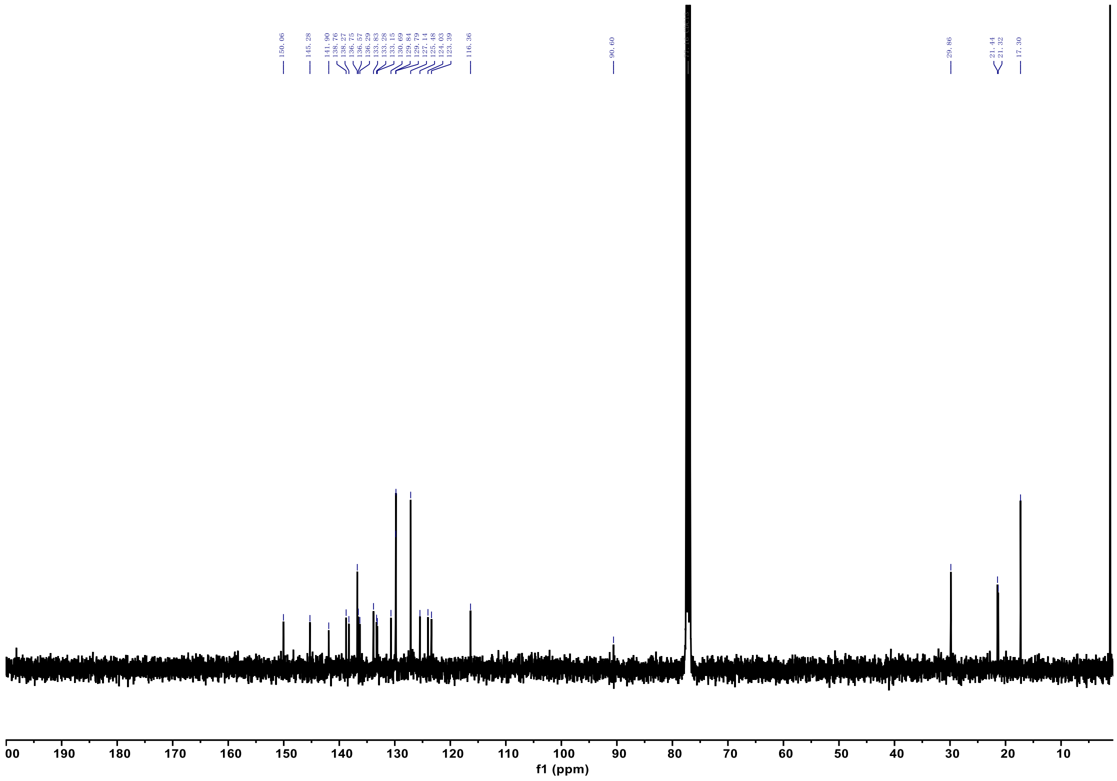

^{1}H NMR spectrum (400 MHz, $CDCl_3$, 298 K) of compound **10a**

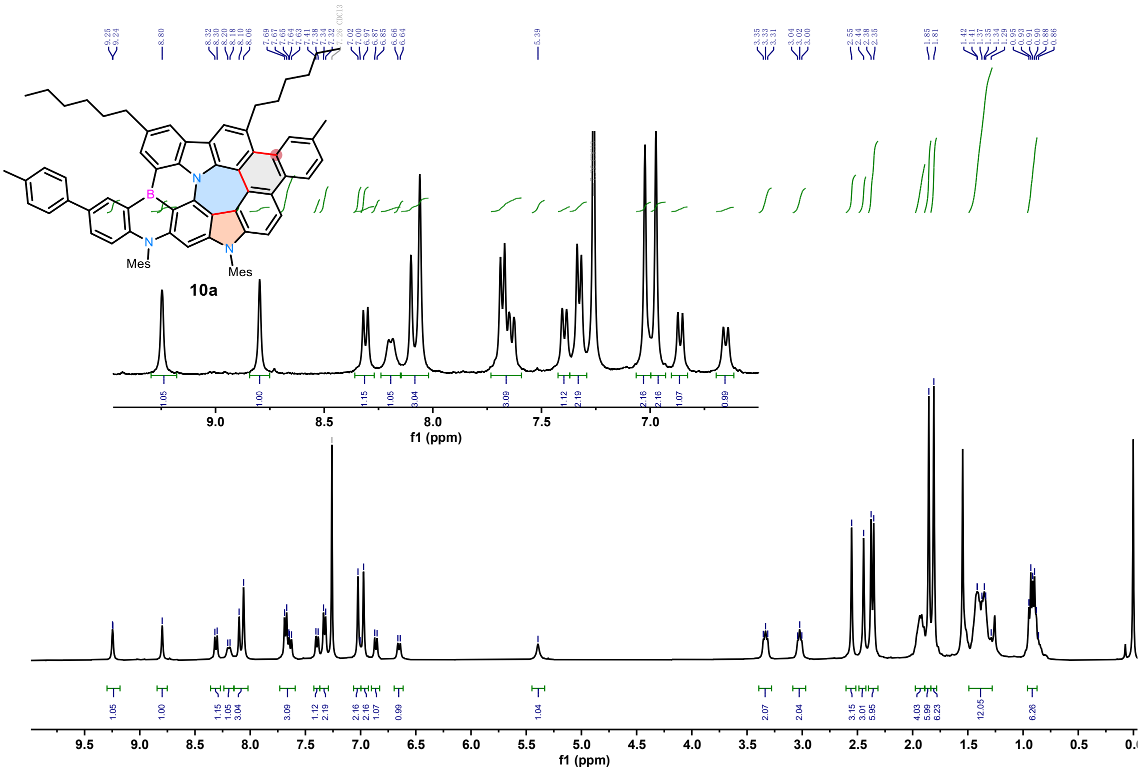

^{13}C NMR spectrum (101 MHz, $CDCl_3$, 298 K) of compound **10a**

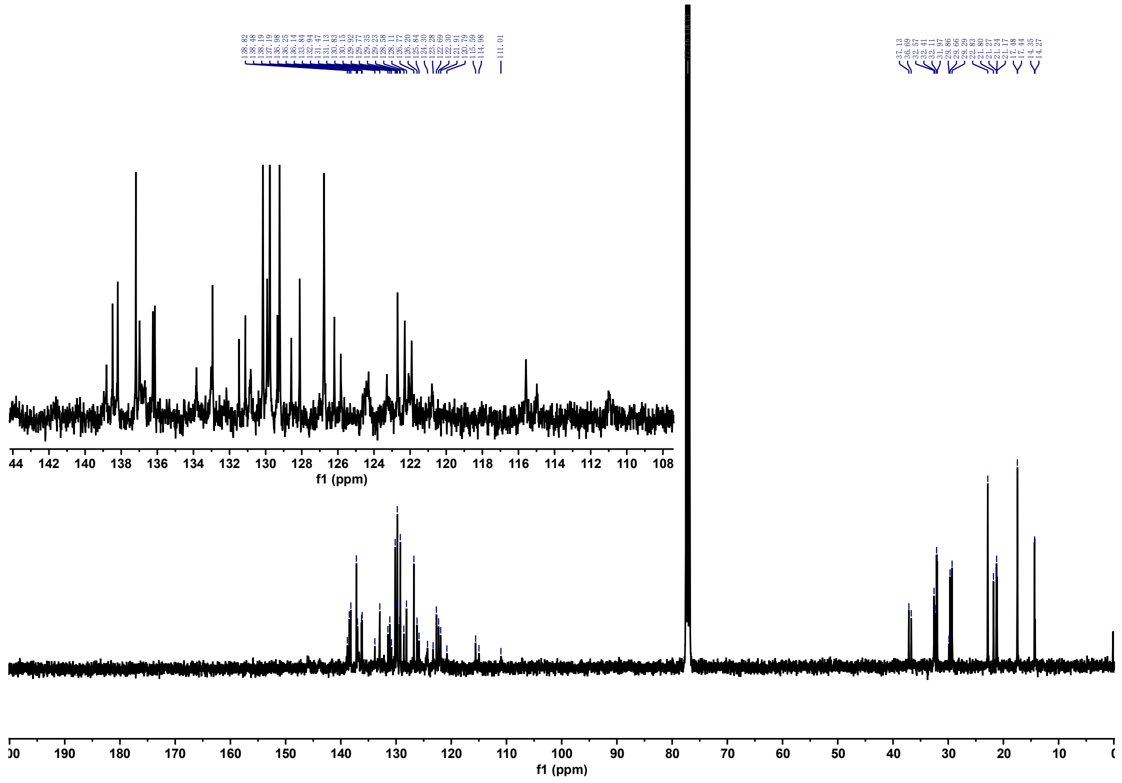

^{1}H NMR spectrum (400 MHz, $CDCl_3$, 298 K) of compound **10b**

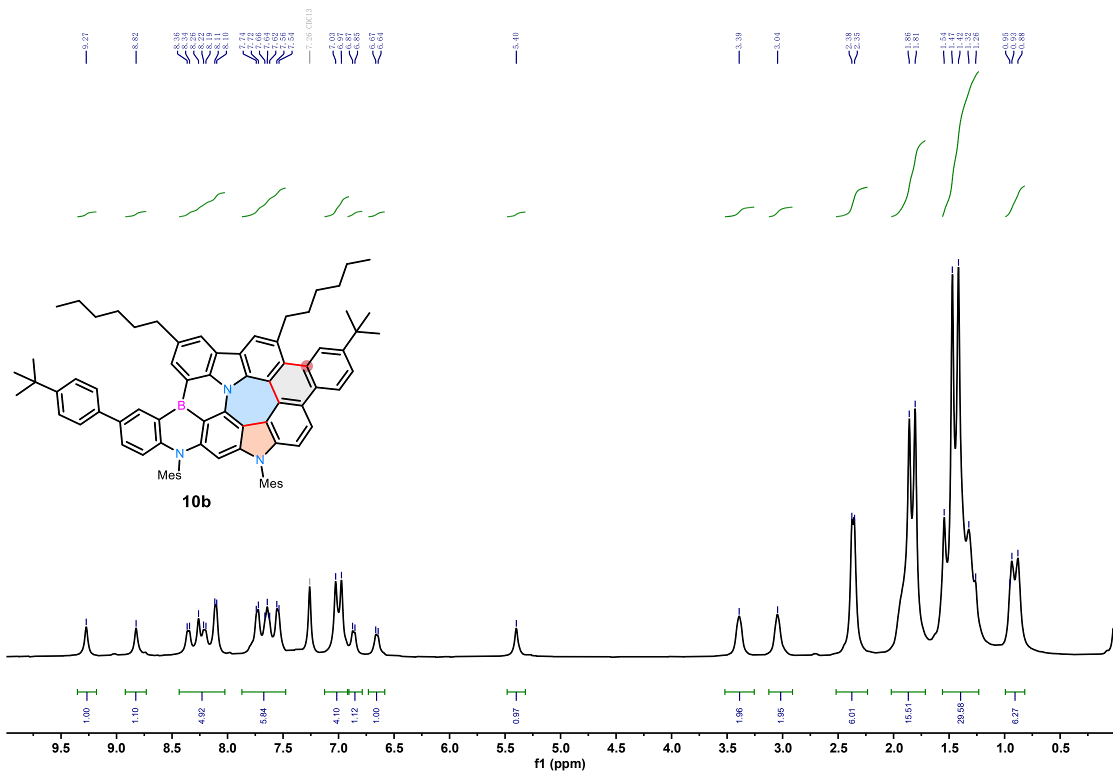

^{13}C NMR spectrum (101 MHz, $CDCl_3$, 298 K) of compound **10b**

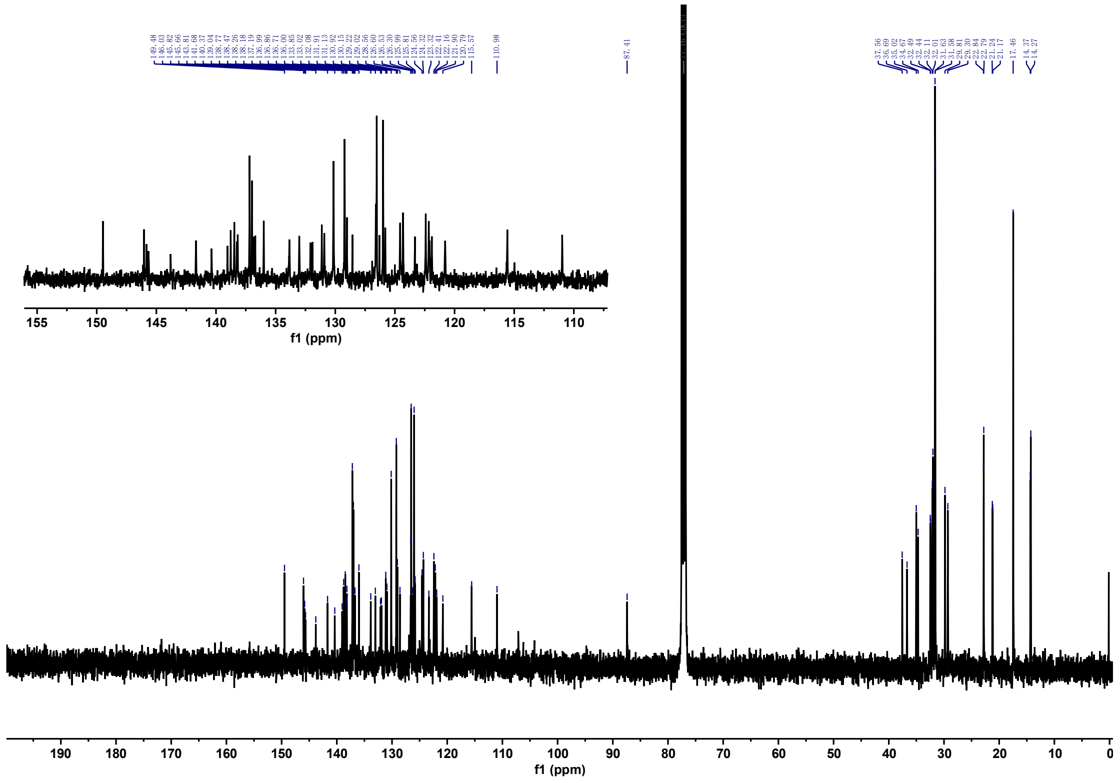

^{1}H NMR spectrum (400 MHz, $CDCl_3$, 298 K) of compound **10c**

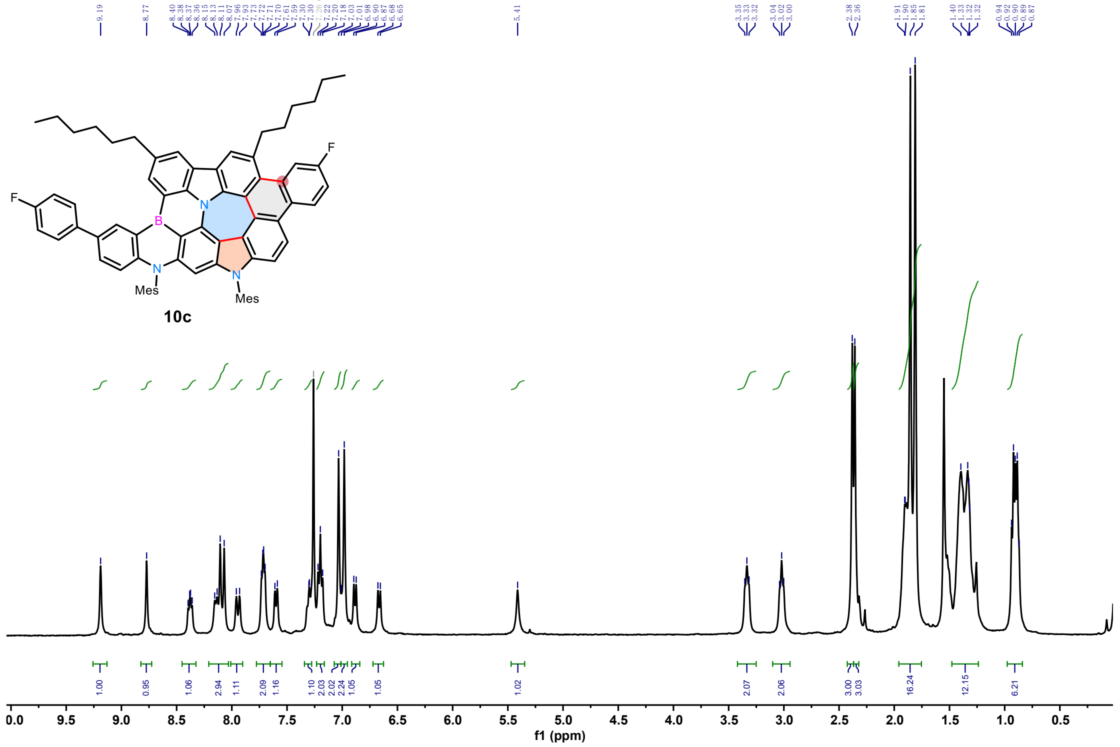

^{13}C NMR spectrum (101 MHz, $CDCl_3$, 298 K) of compound **10c**

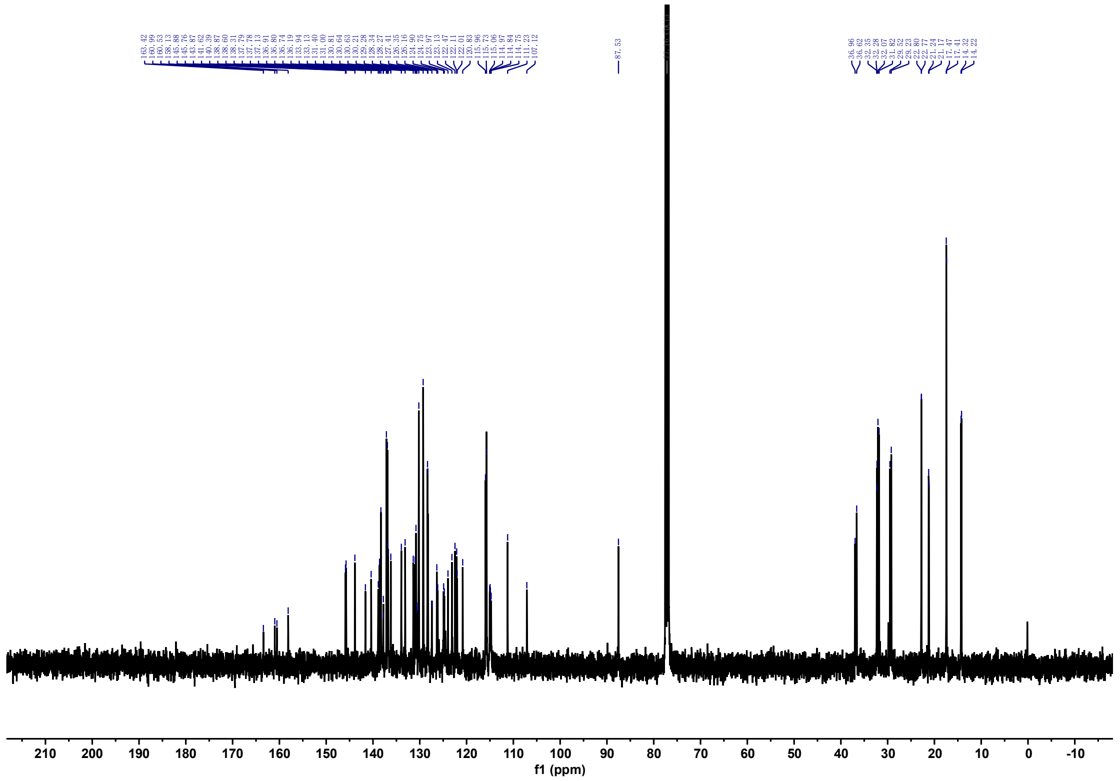

^{1}H NMR spectrum (400 MHz, $CDCl_3$, 298 K) of compound **10d**

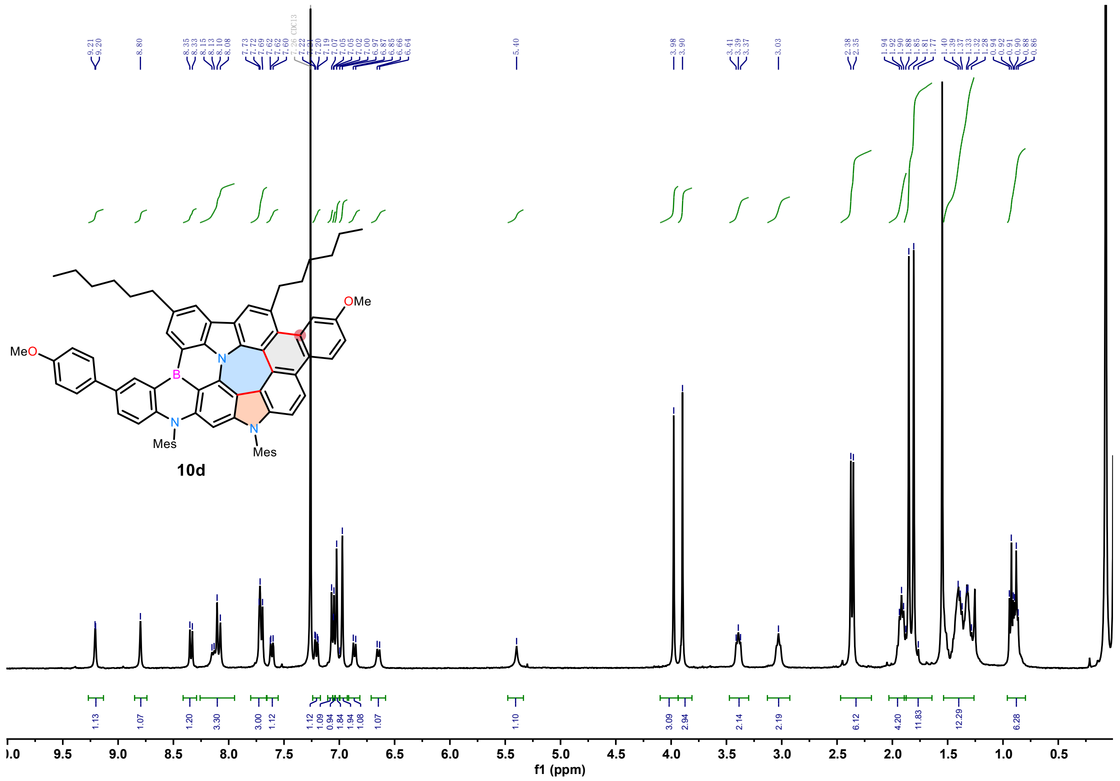

^{13}C NMR spectrum (101 MHz, $CDCl_3$, 298 K) of compound **10d**

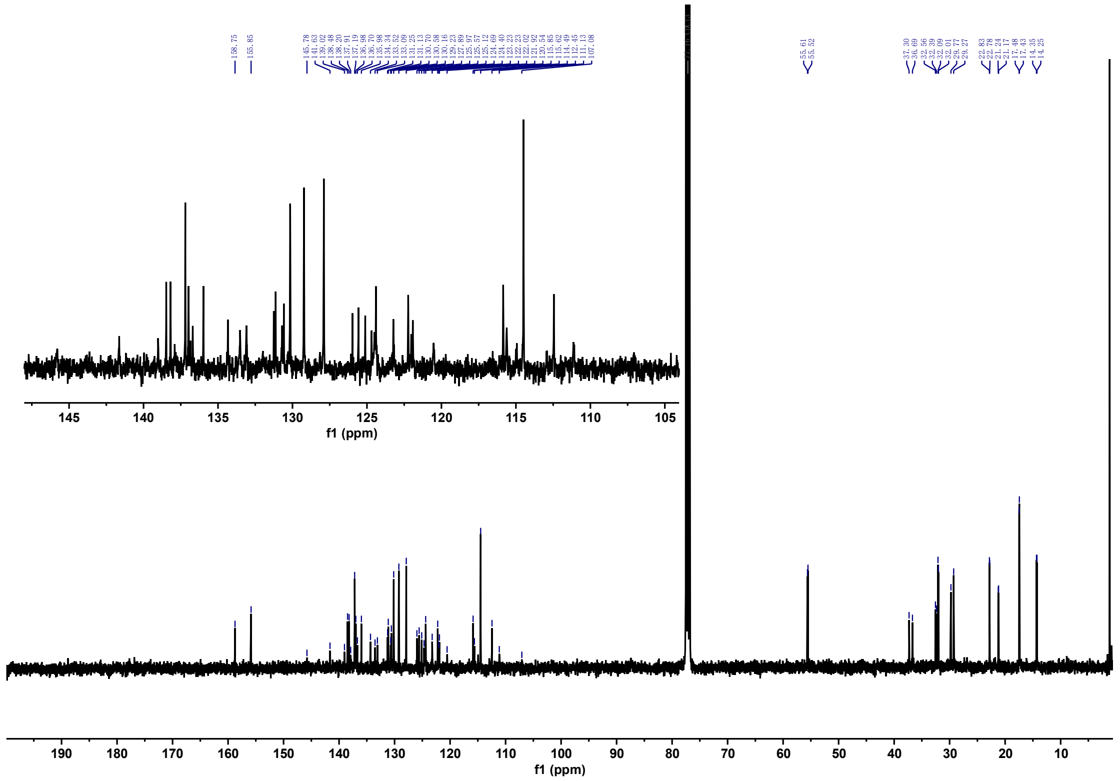

^{1}H NMR spectrum (400 MHz, $CDCl_3$, 298 K) of compound **10e**

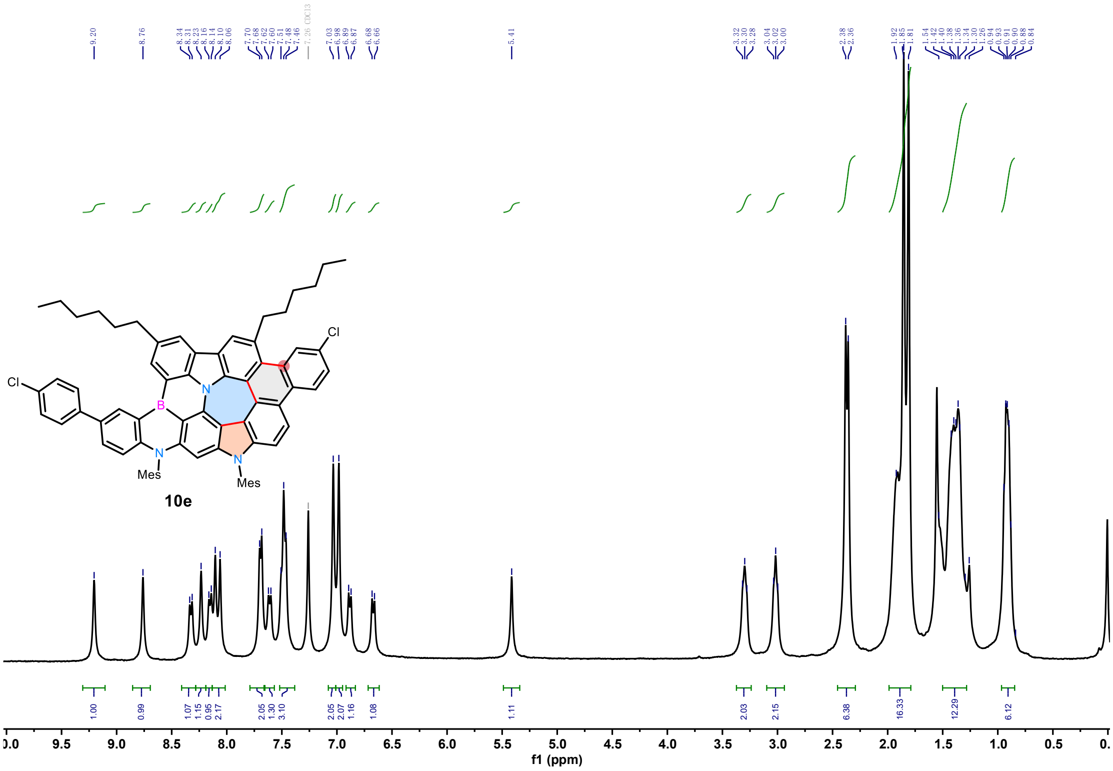

^{13}C NMR spectrum (101 MHz, $CDCl_3$, 298 K) of compound **10e**

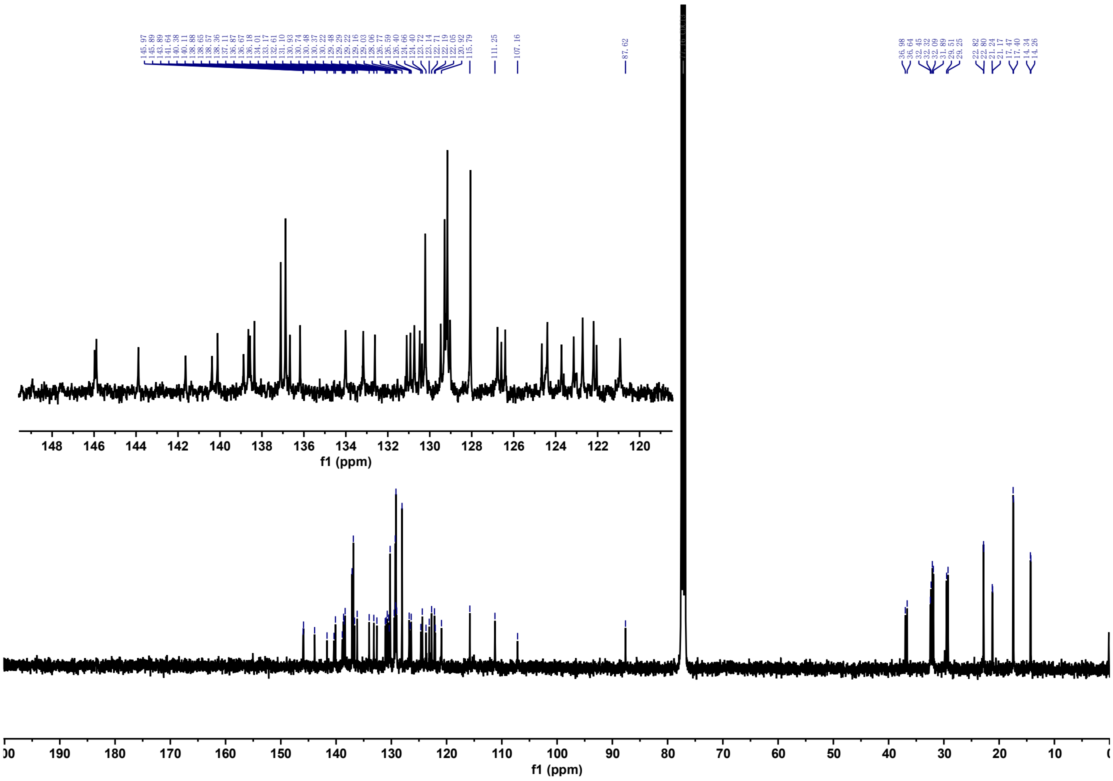

^{1}H NMR spectrum (400 MHz, $CDCl_3$, 298 K) of compound **10f**

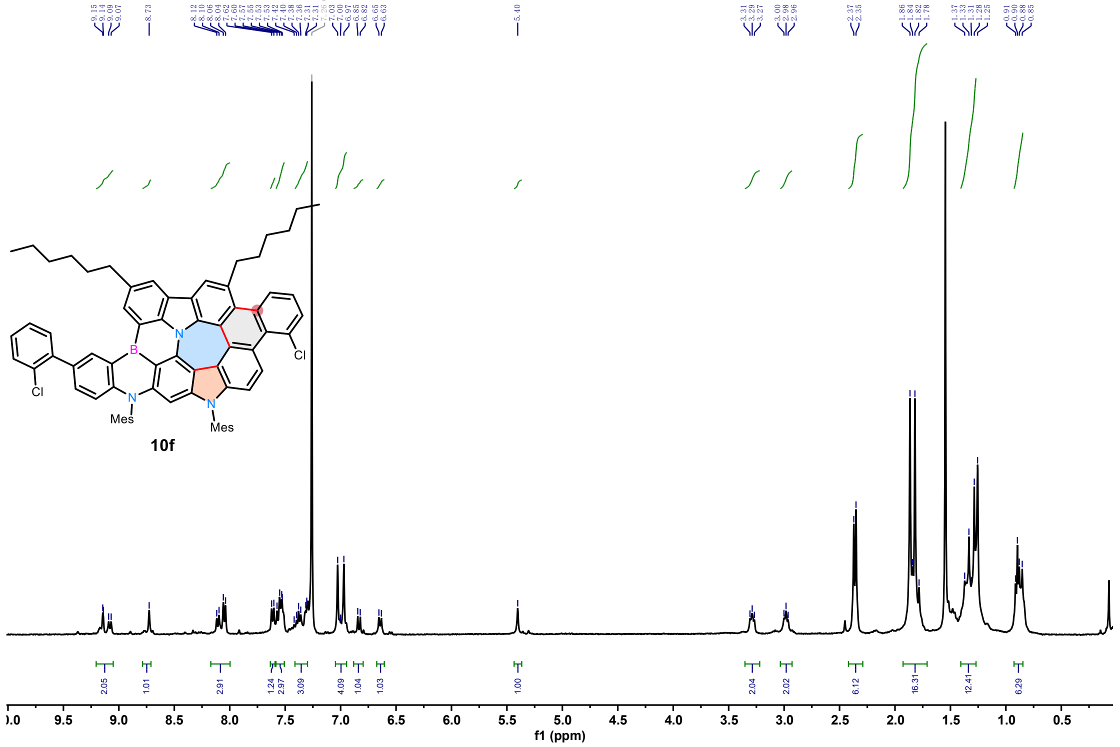

^{13}C NMR spectrum (101 MHz, $CDCl_3$, 298 K) of compound **10f**

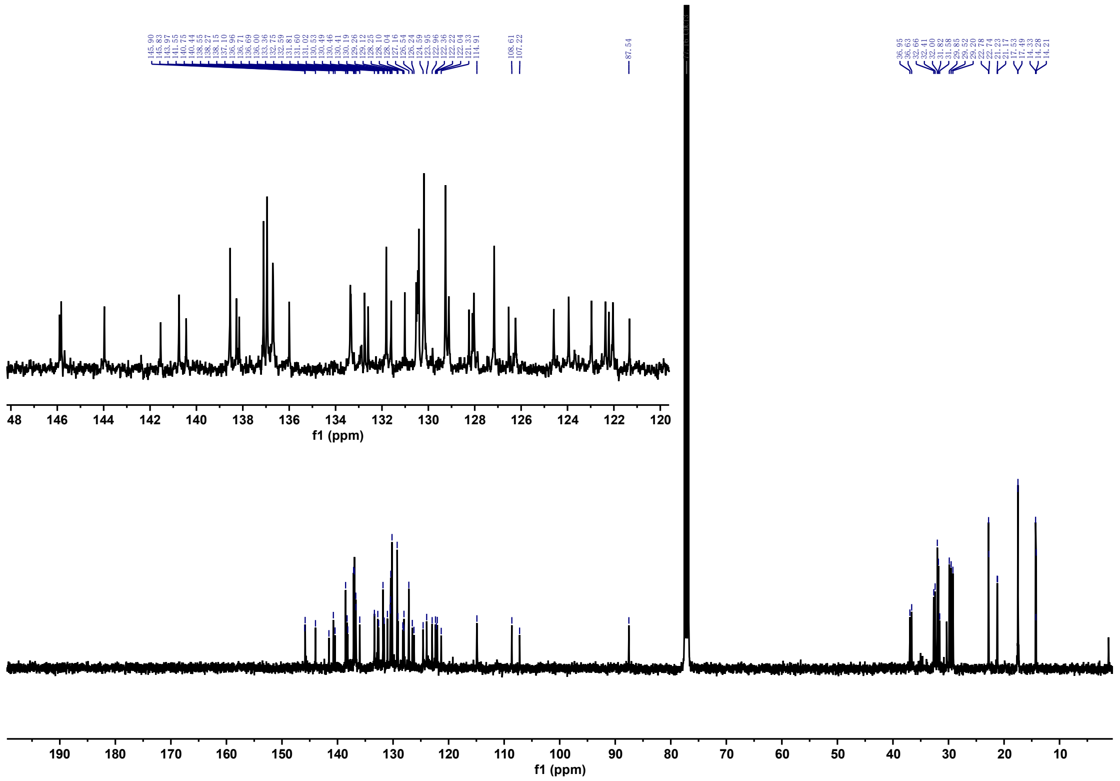

^{1}H NMR spectrum (400 MHz, $CDCl_3$, 298 K) of compound **10g**

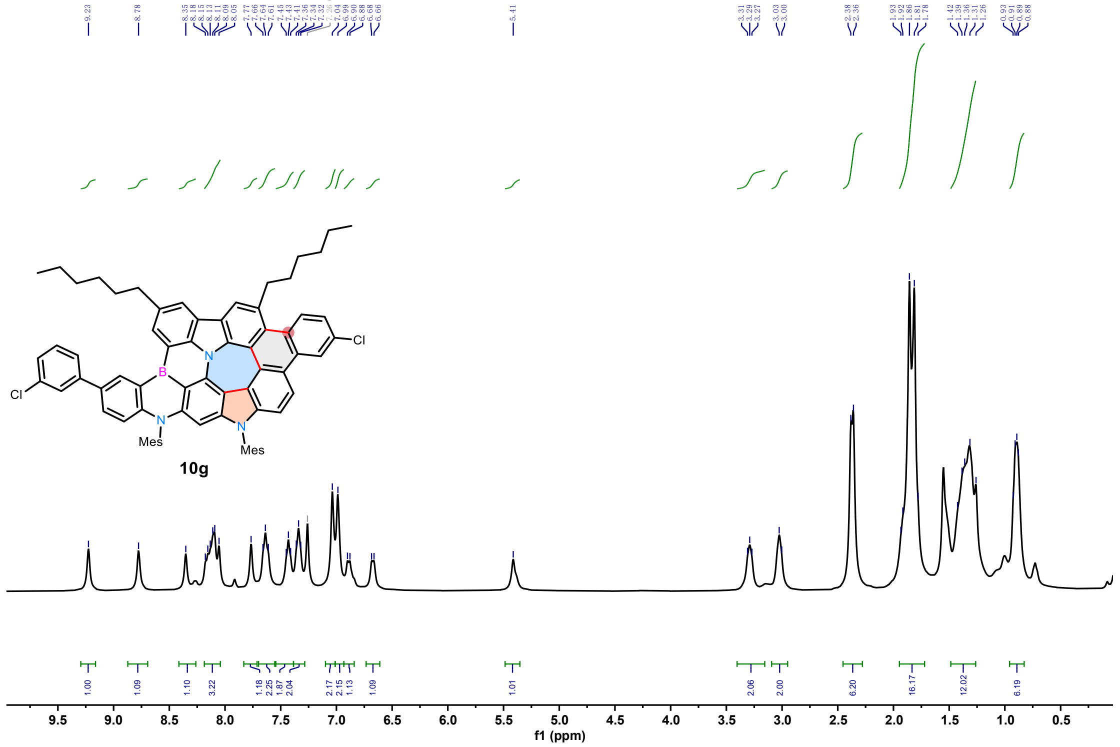

^{13}C NMR spectrum (101 MHz, $CDCl_3$, 298 K) of compound **10g**

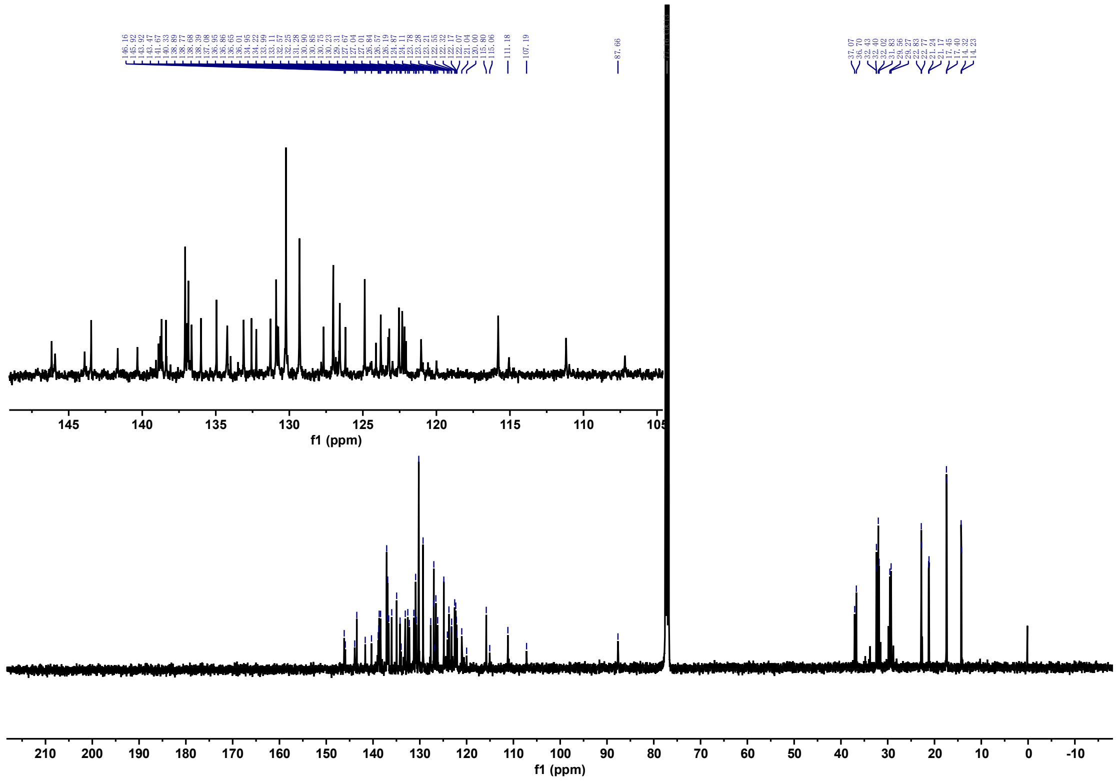

^{1}H NMR spectrum (400 MHz, $CDCl_3$, 298 K) of compound **10h**

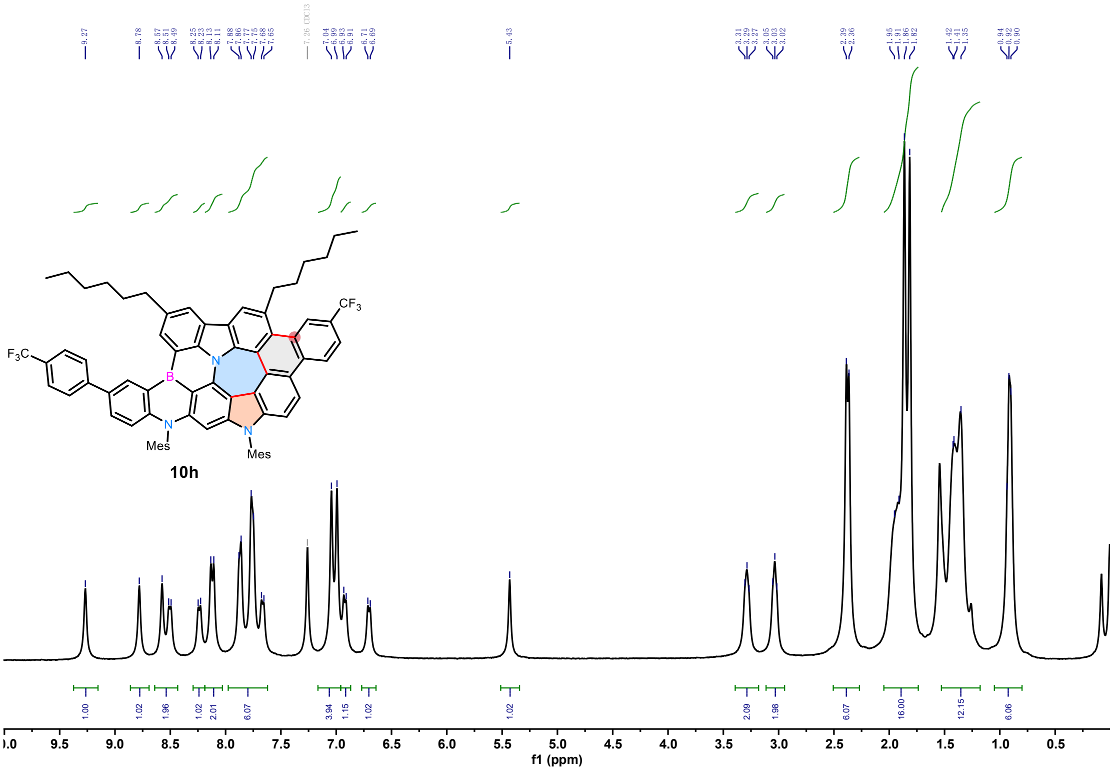

^{13}C NMR spectrum (101 MHz, $CDCl_3$, 298 K) of compound **10h**

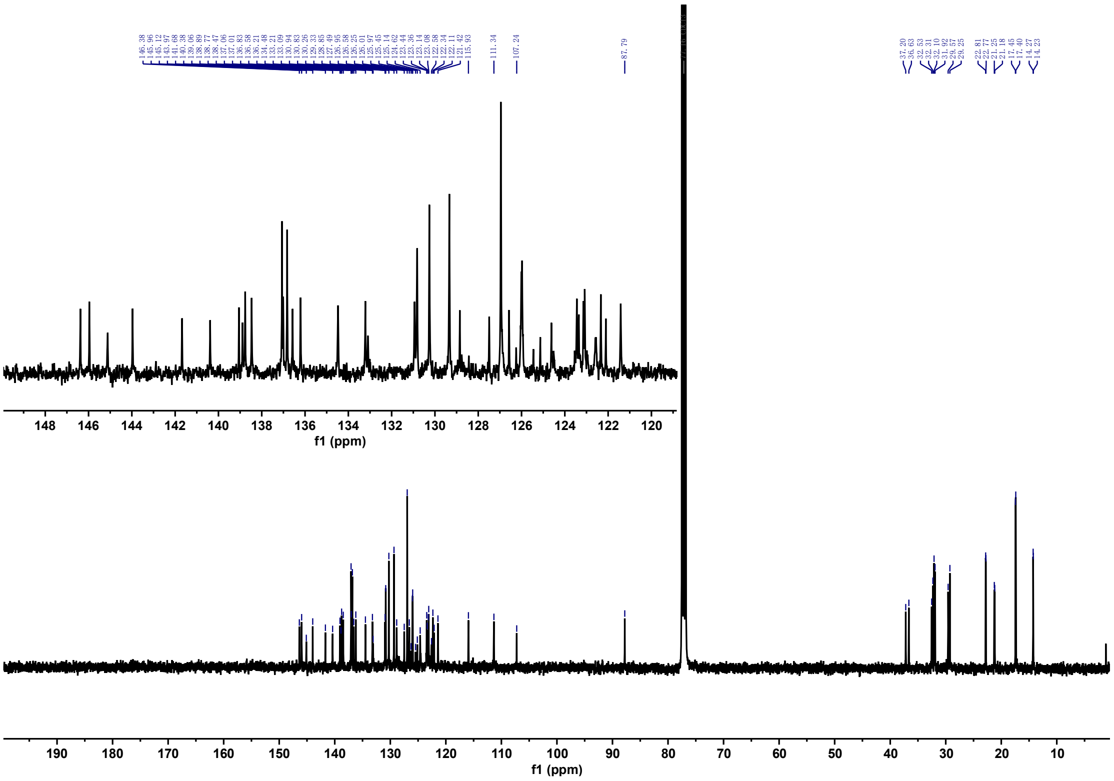

^{1}H NMR spectrum (400 MHz, $CDCl_3$, 298 K) of compound **10i**

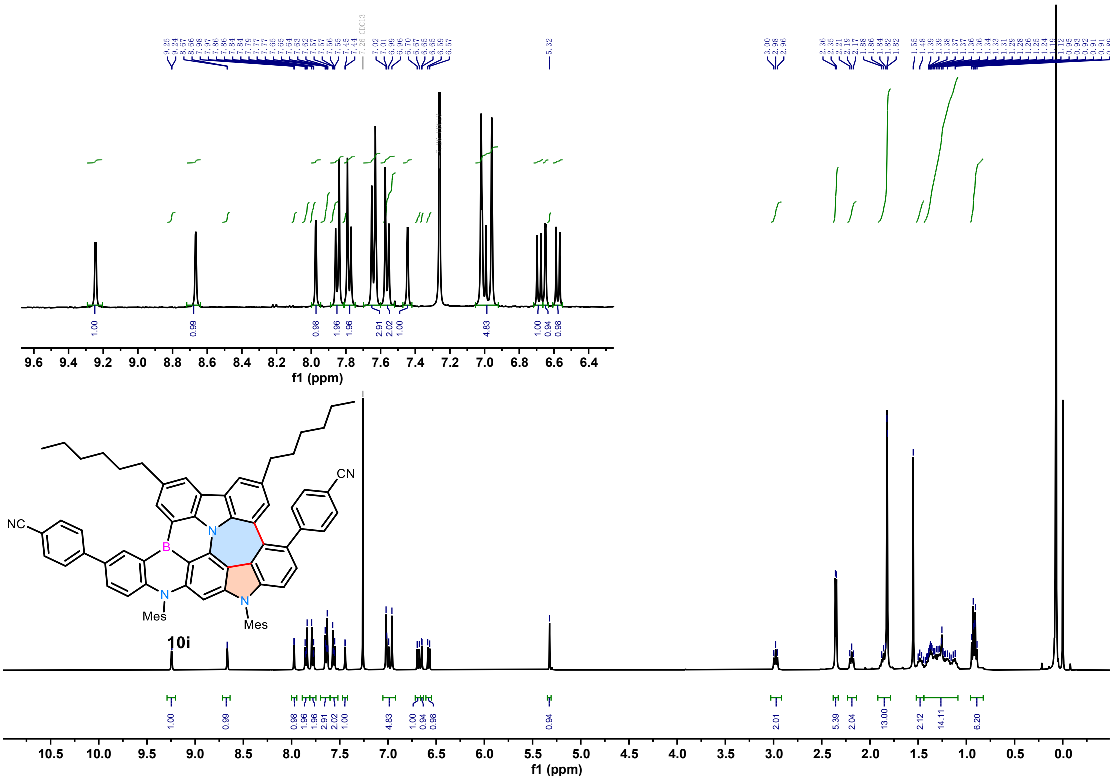

^{13}C NMR spectrum (101 MHz, $CDCl_3$, 298 K) of compound **10i**

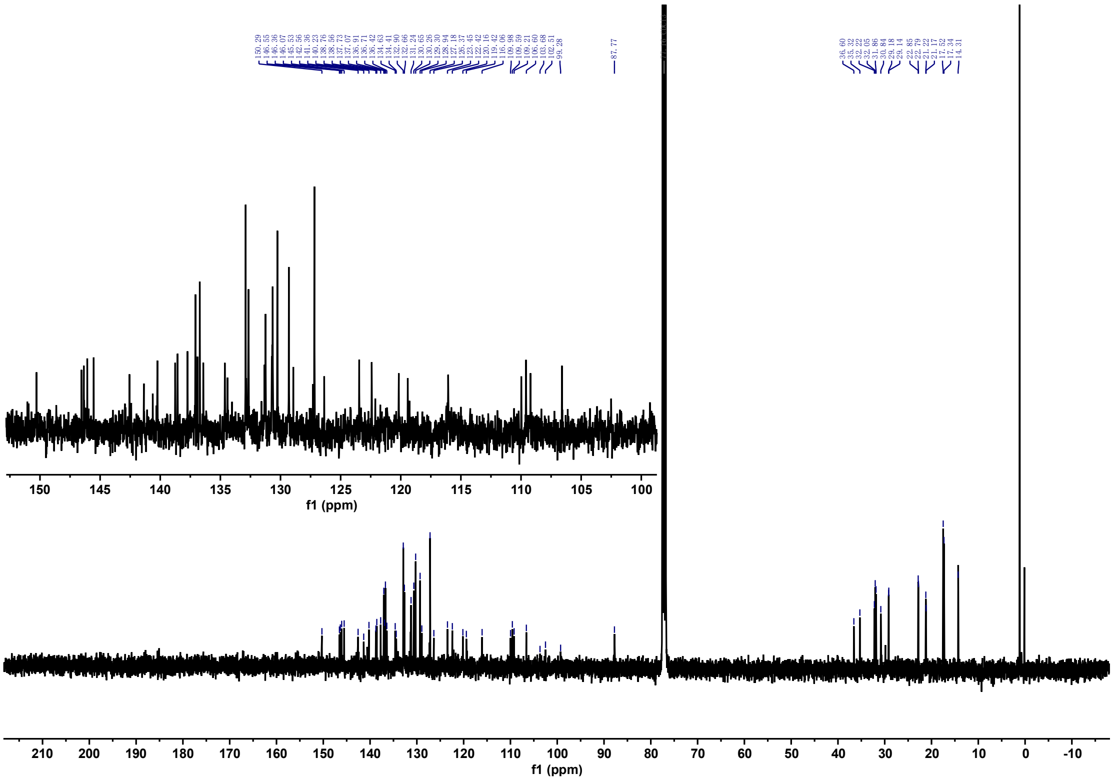

^{1}H NMR spectrum (600 MHz, Acetone-*d6*/CS_2, 298 K) of compound **10j**

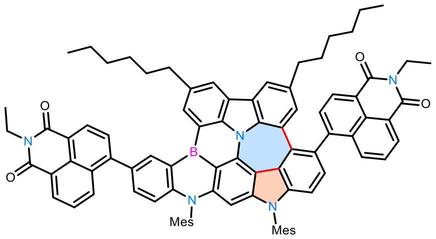

10j

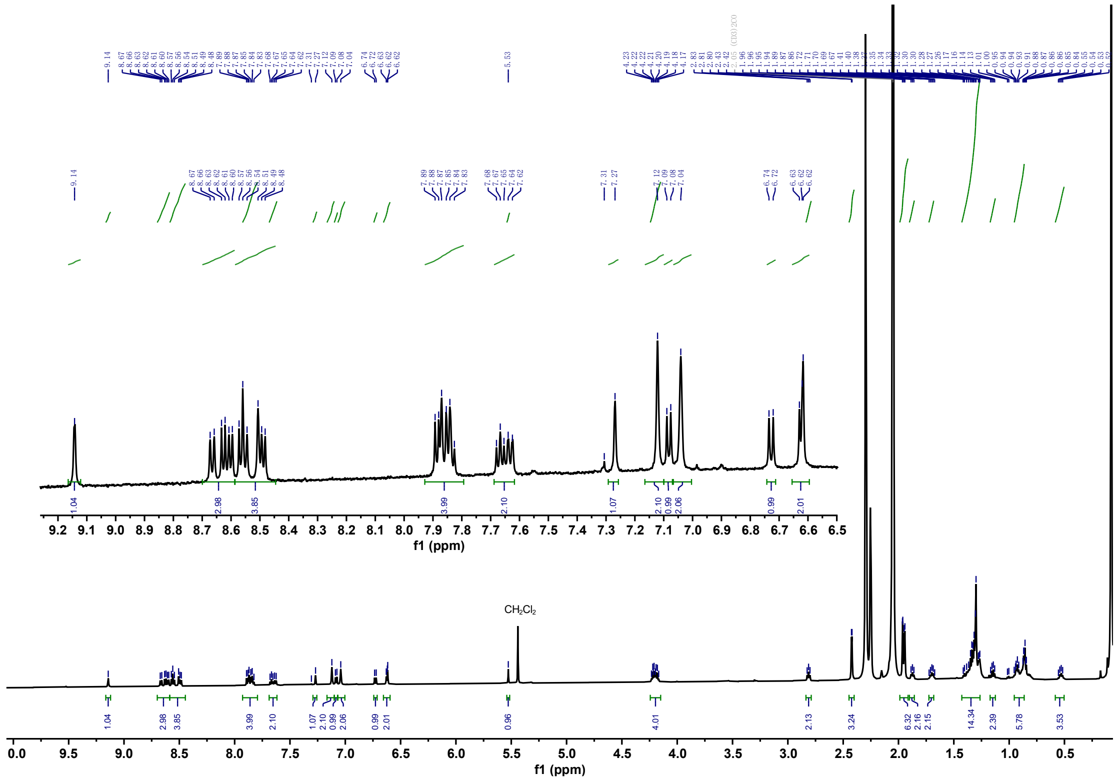

^{1}H NMR spectrum (400 MHz, Acetone-*d6*/CS_2, 298 K) of compound **10k**

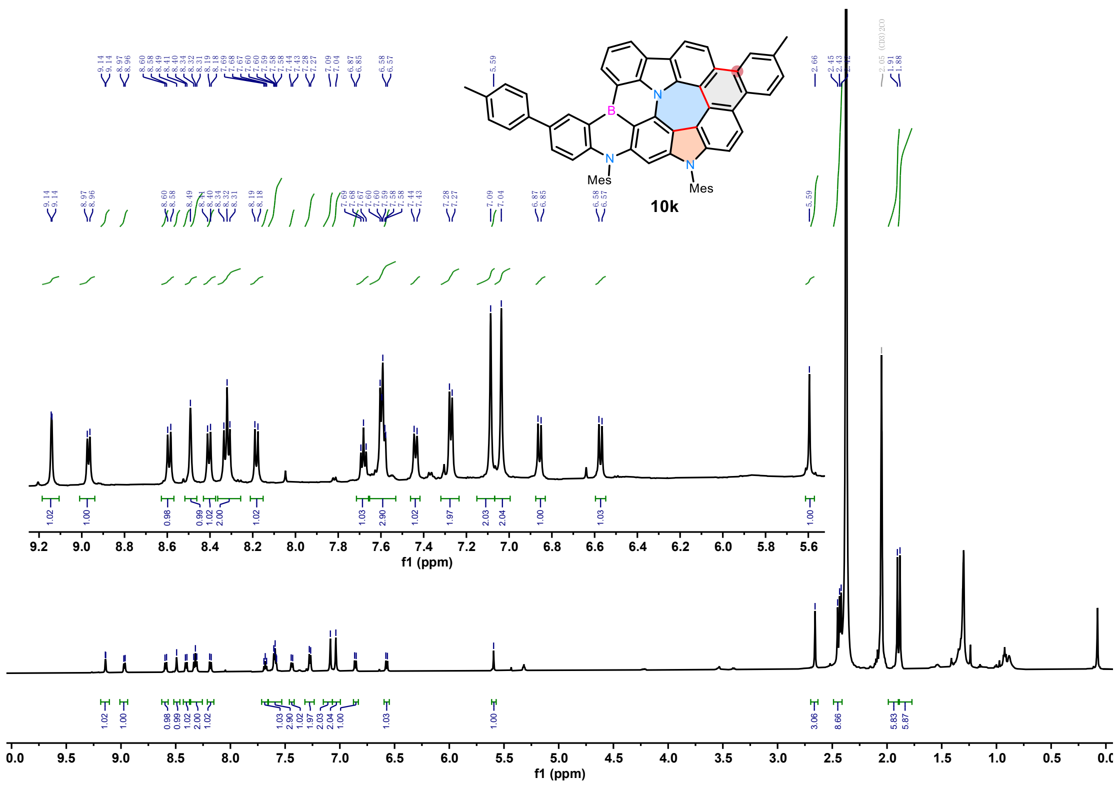

^{13}C NMR spectrum (151 MHz, Acetone-*d6*/CS_2, 298 K) of compound **10k**

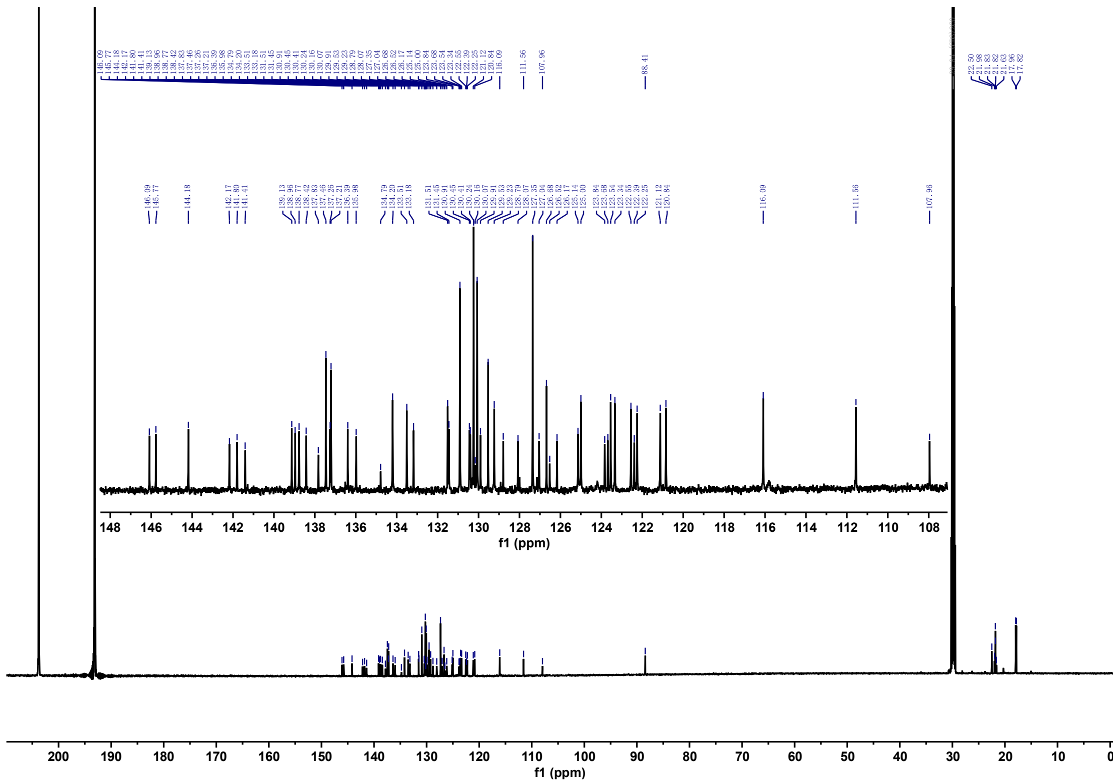

^{1}H NMR spectrum (400 MHz, $CDCl_3$, 298 K) of compound **11**

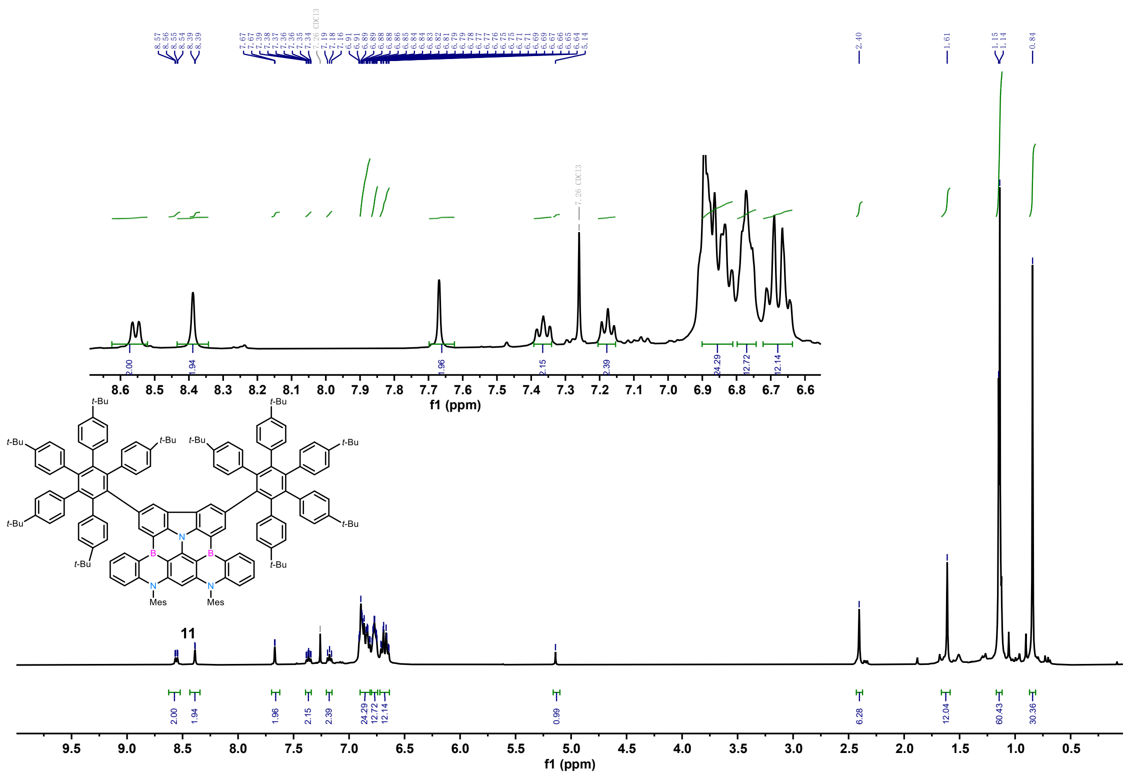

^{13}C NMR spectrum (101 MHz, $CDCl_3$, 298 K) of compound **11**

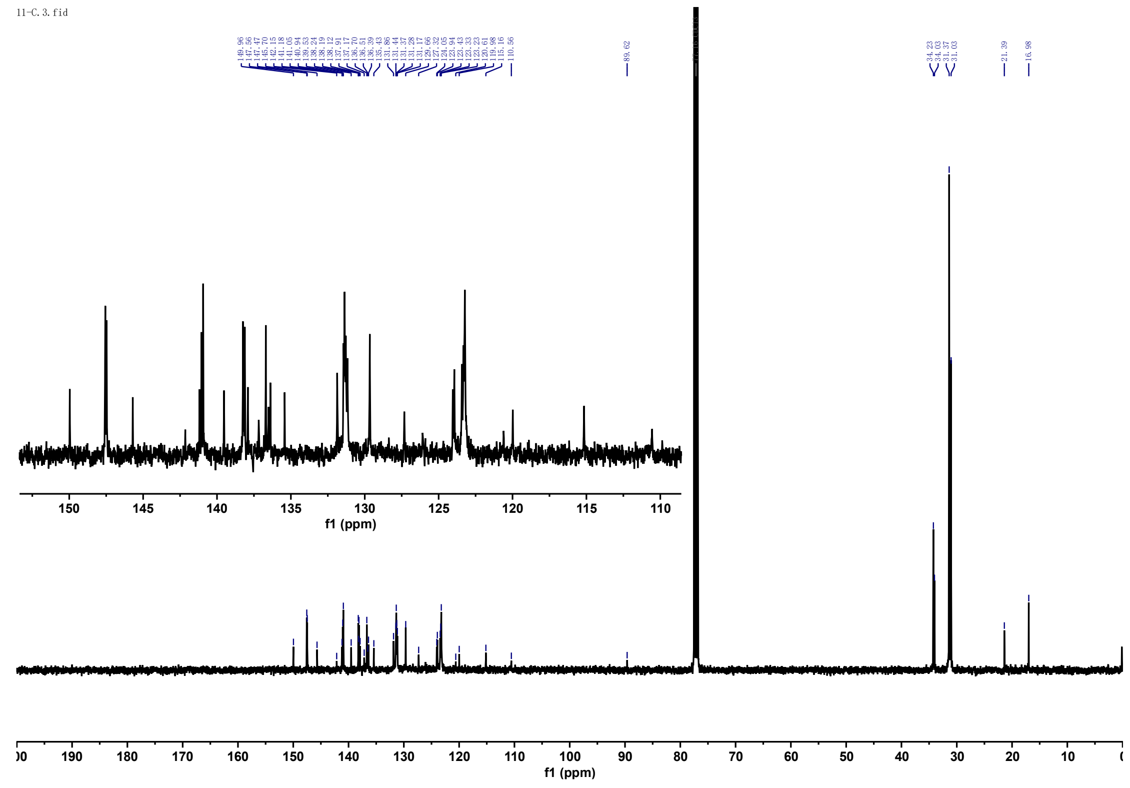

^{1}H NMR spectrum (600 MHz, Acetone-*d6*/CS_2, 298 K) of compound **12**

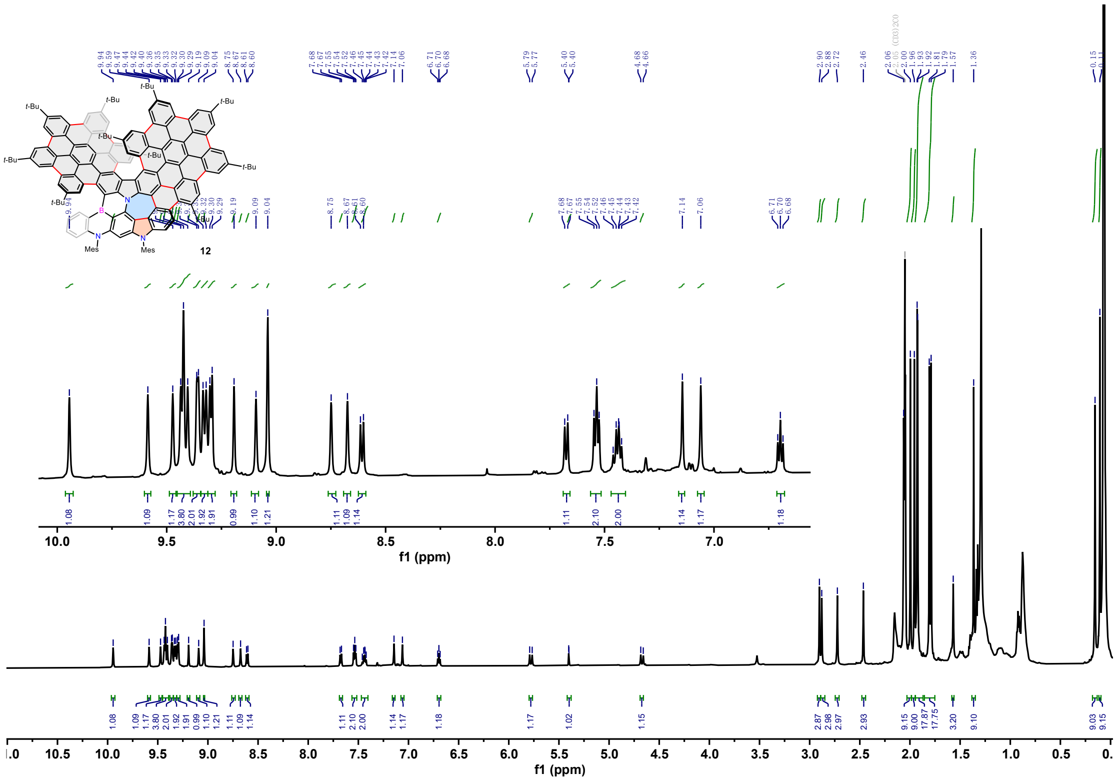

^{13}C NMR spectrum (151 MHz, Acetone-*d6*/CS_2, 298 K) of compound **12**

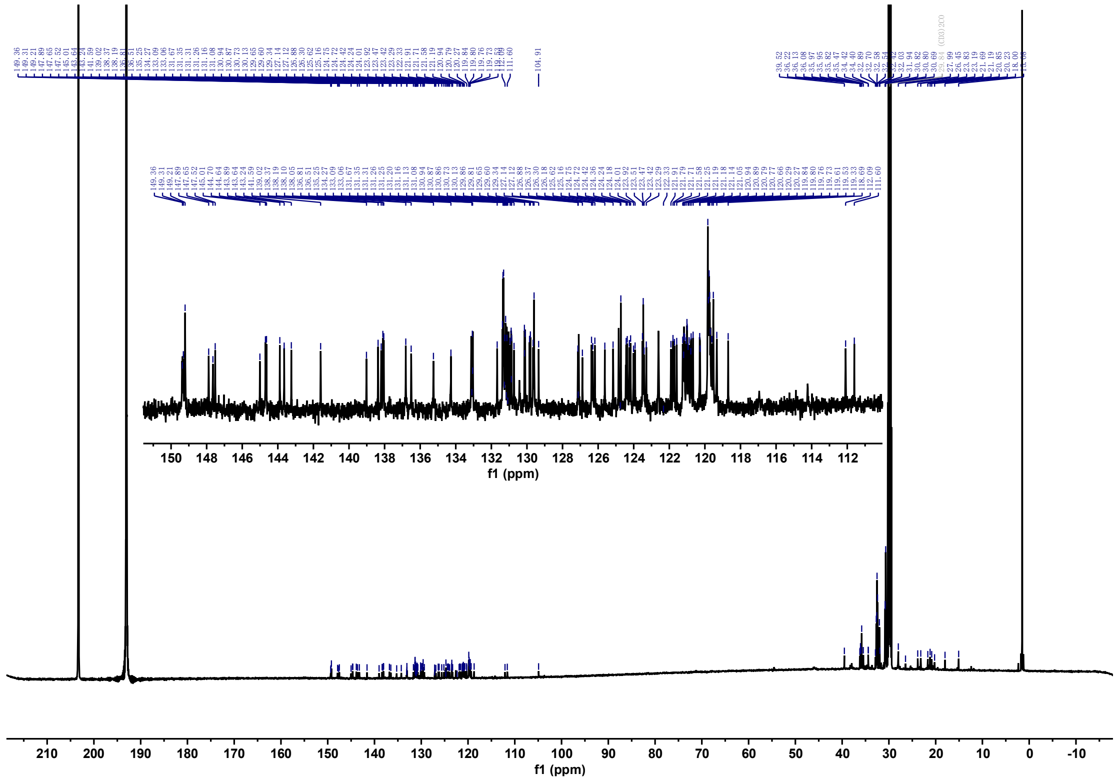

^{1}H NMR spectrum (400 MHz, $CDCl_3$, 298 K) of compound **13**

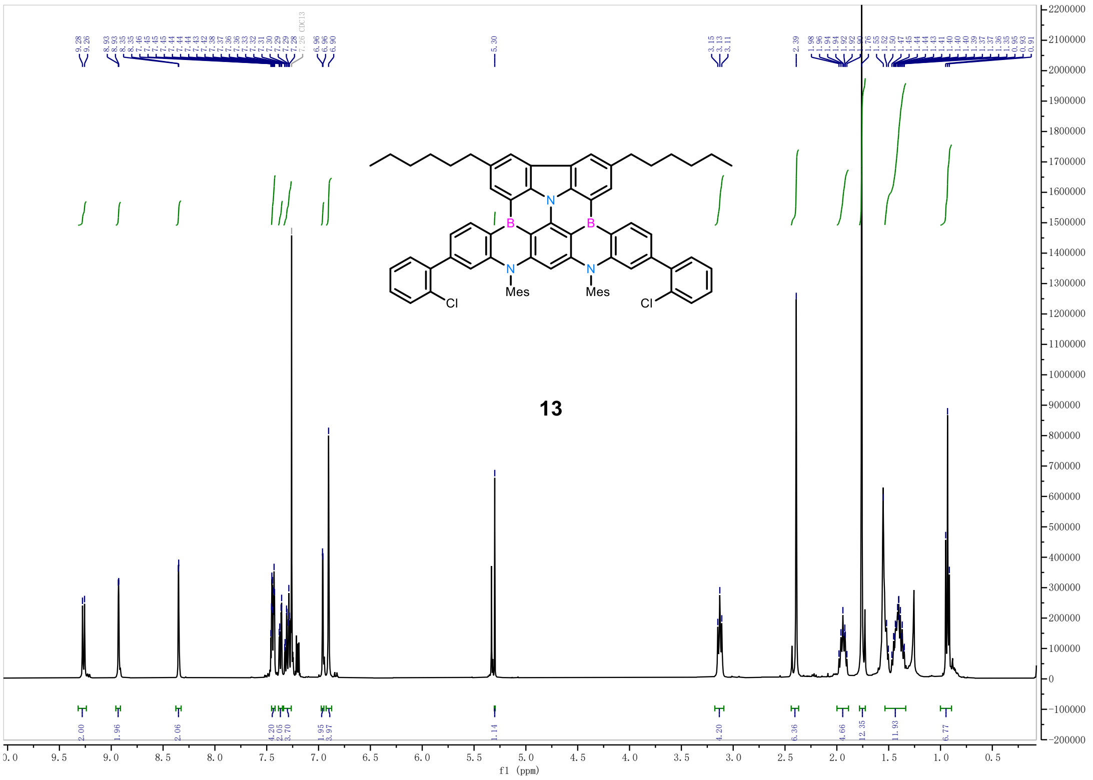

^{13}C NMR spectrum (101 MHz, $CDCl_3$, 298 K) of compound **13**

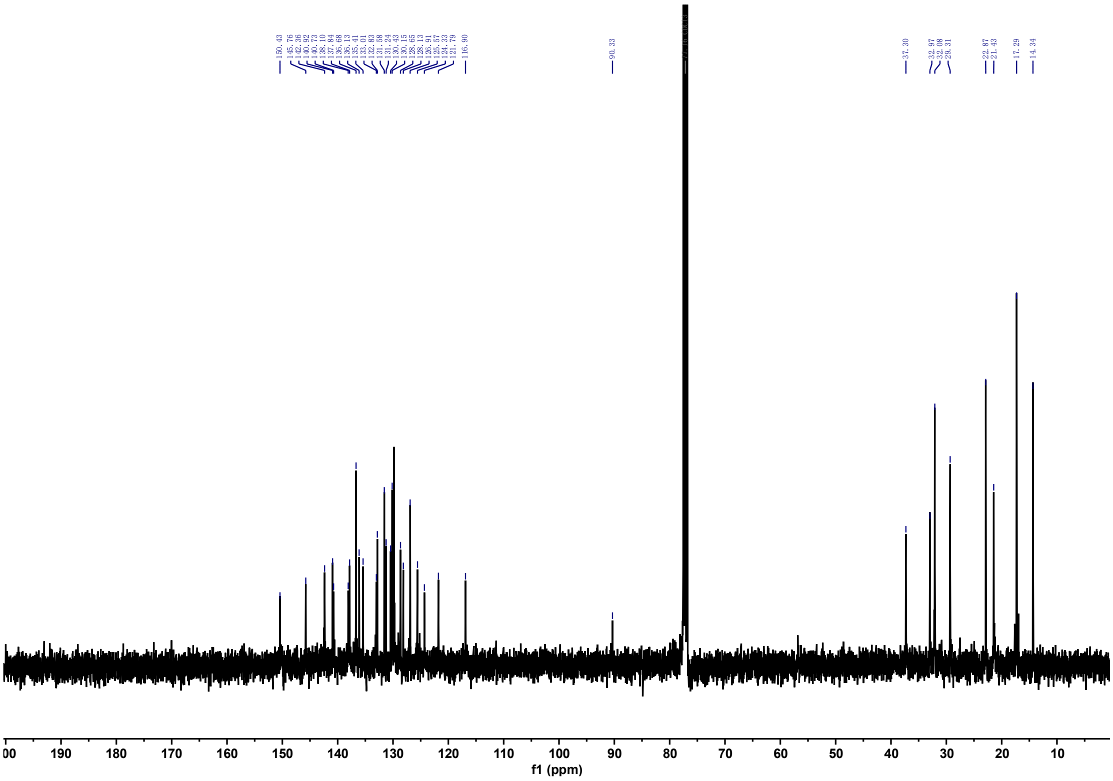

^{1}H NMR spectrum (600 MHz, $CDCl_3$, 298 K) of compound **14**

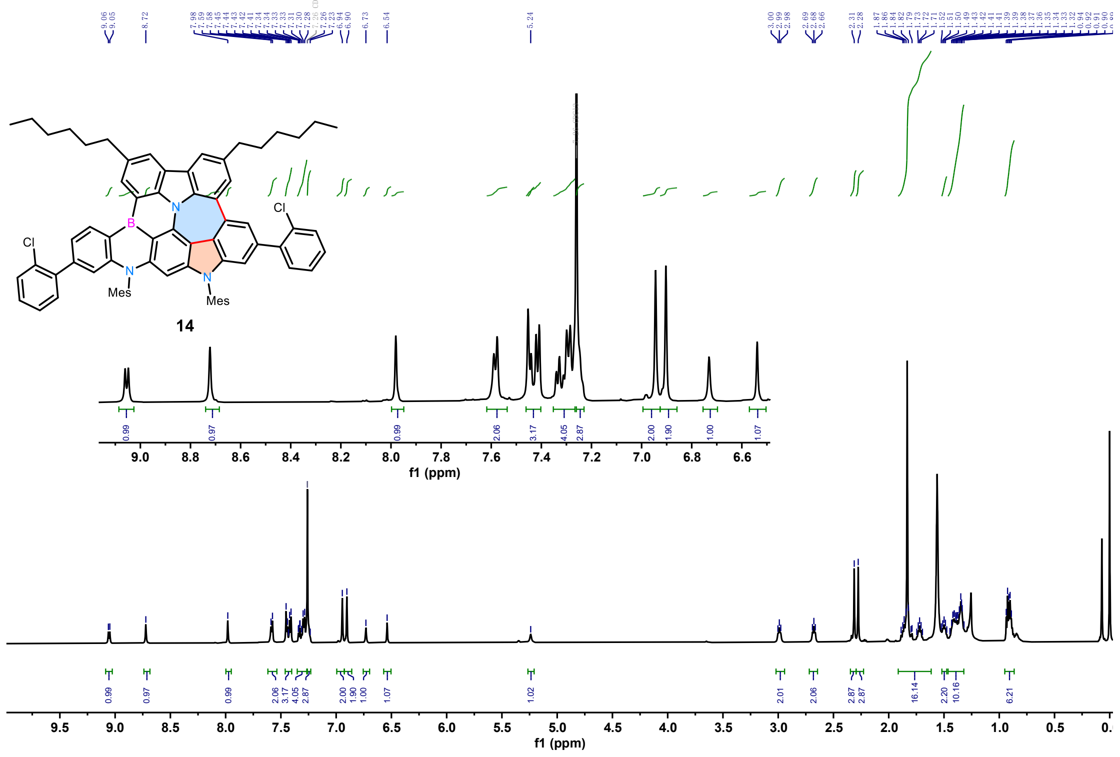

^{13}C NMR spectrum (151 MHz, $CDCl_3$, 298 K) of compound **14**

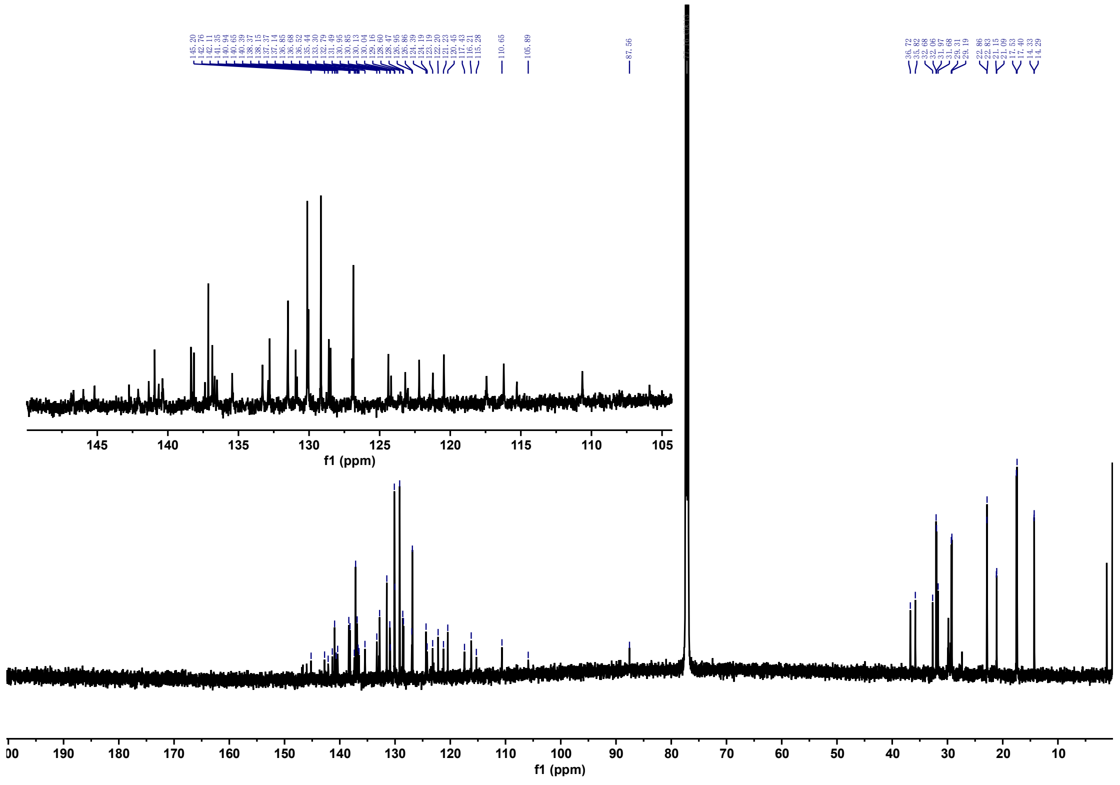

^{1}H NMR spectrum (400 MHz, $CDCl_3$, 298 K) of compound **15a**

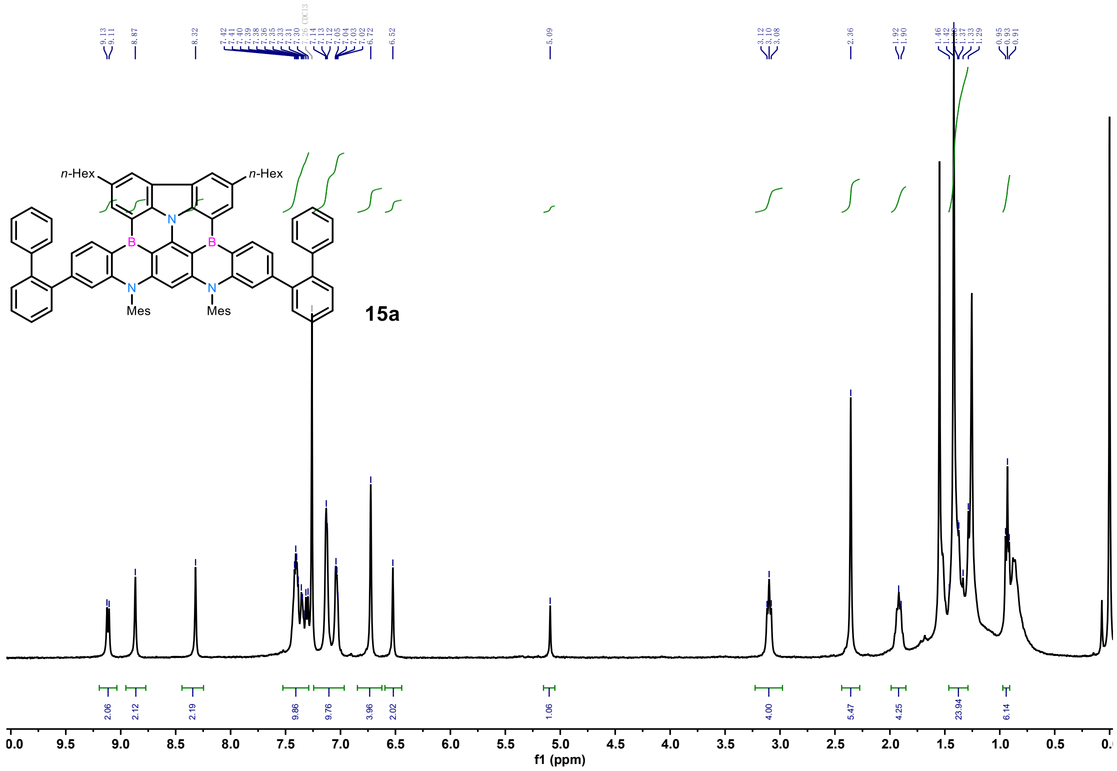

^{13}C NMR spectrum (101 MHz, $CDCl_3$, 298 K) of compound **15a**

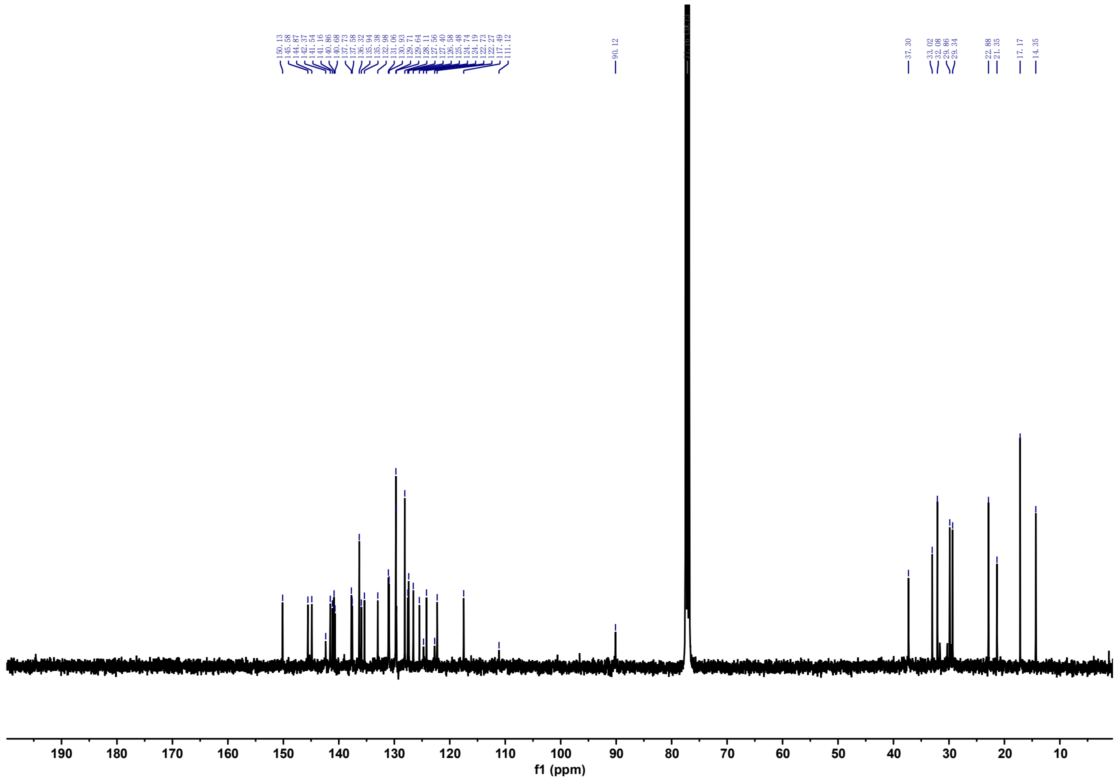

^{1}H NMR spectrum (400 MHz, $CDCl_3$, 298 K) of compound **15b**

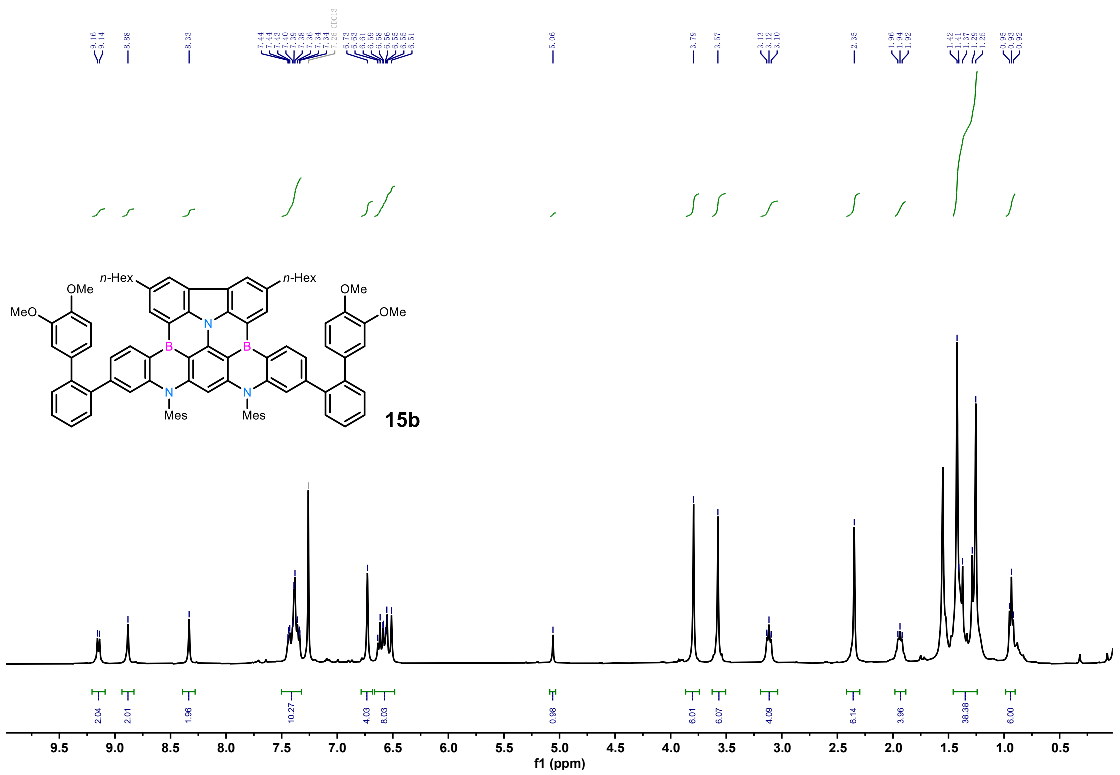

^{13}C NMR spectrum (101 MHz, $CDCl_3$, 298 K) of compound **15b**

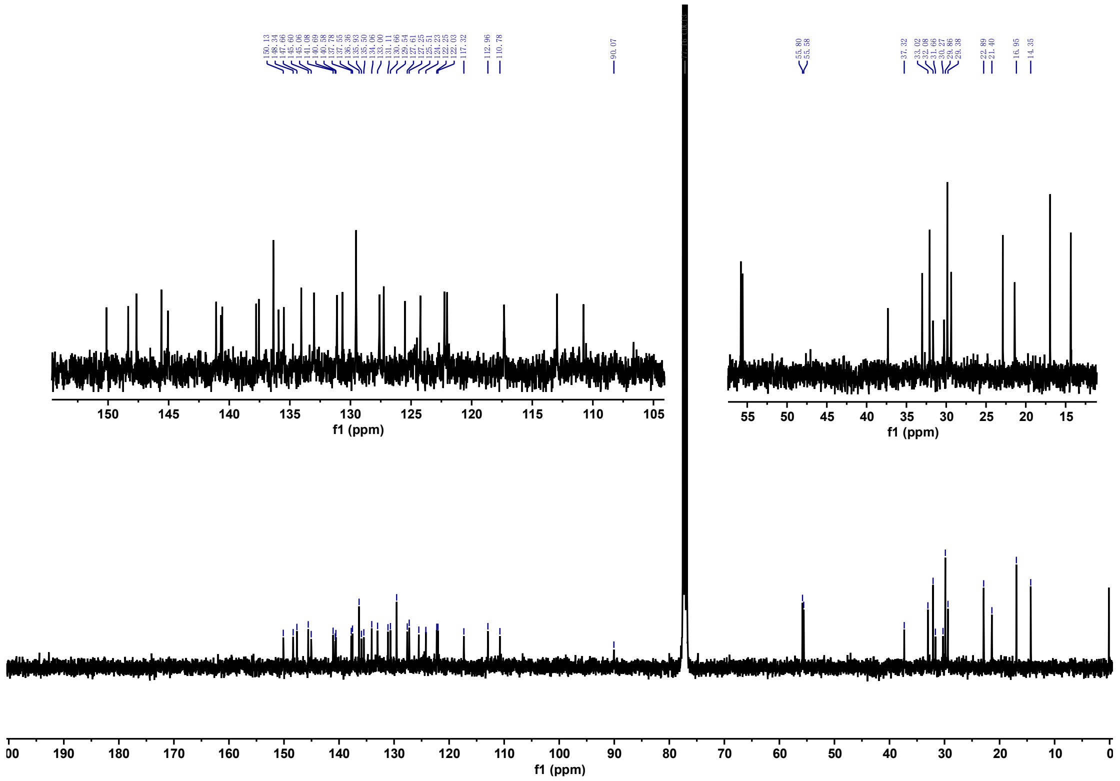

^{1}H NMR spectrum (600 MHz, Acetone-*d6*/CS_2, 298 K) of compound **16a**

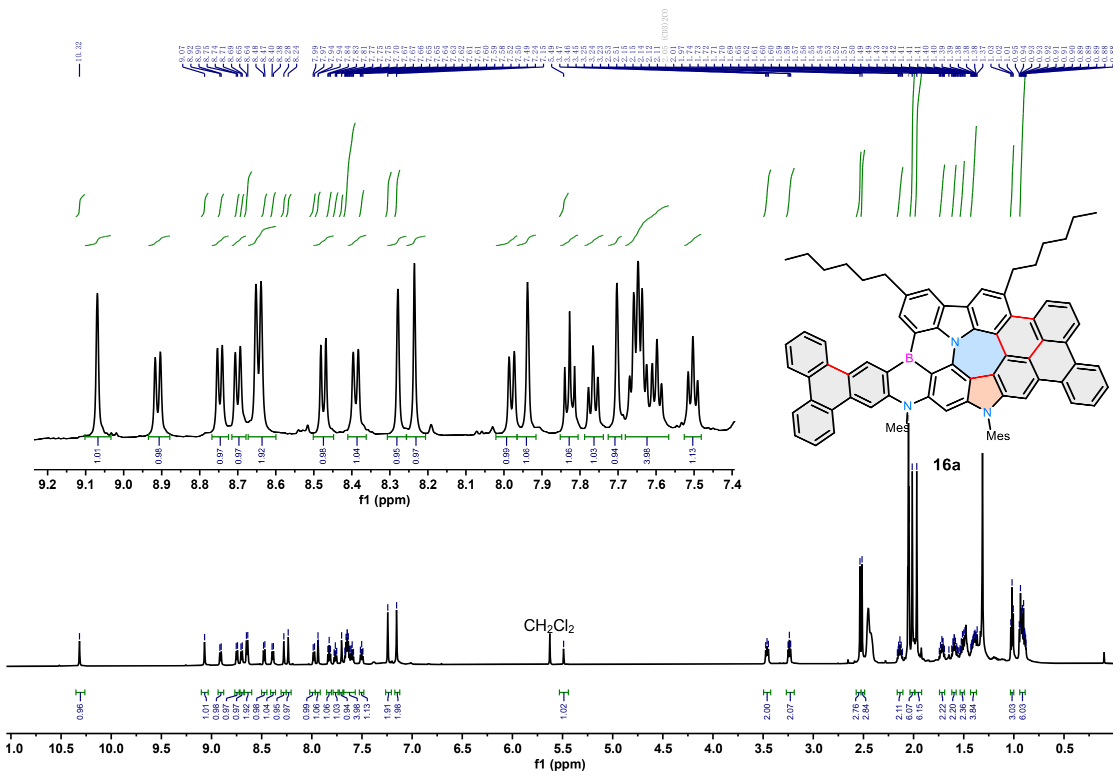

^{13}C NMR spectrum (151 MHz, Acetone-*d6*/CS_2, 298 K) of compound **16a**

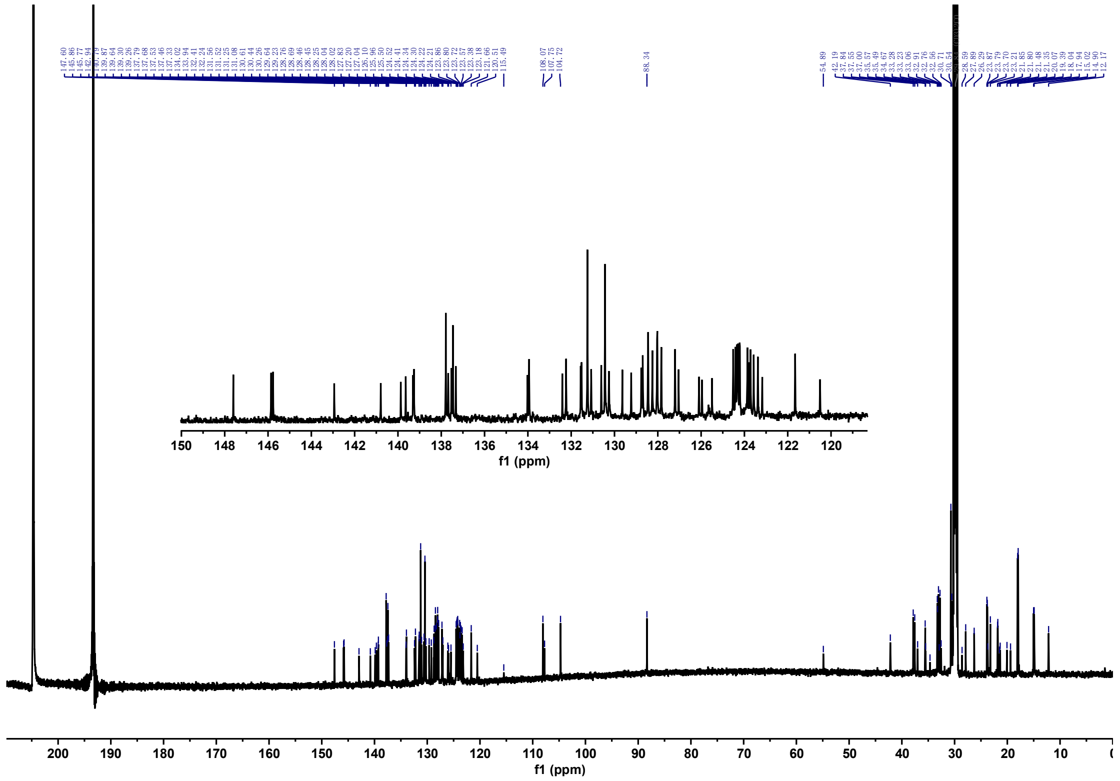

^{1}H NMR spectrum (600 MHz, Acetone-*d6*/CS_2, 298 K) of compound **16b**

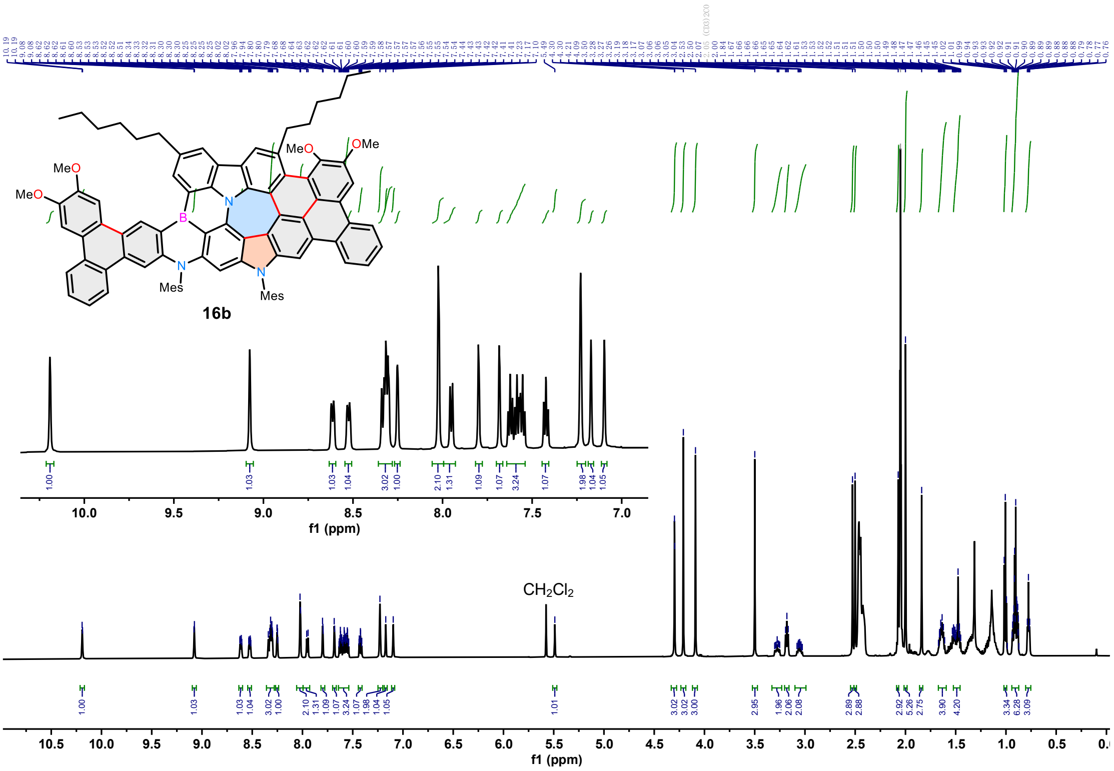

^{13}C NMR spectrum (151 MHz, Acetone-*d6*/CS_2, 298 K) of compound **16b**

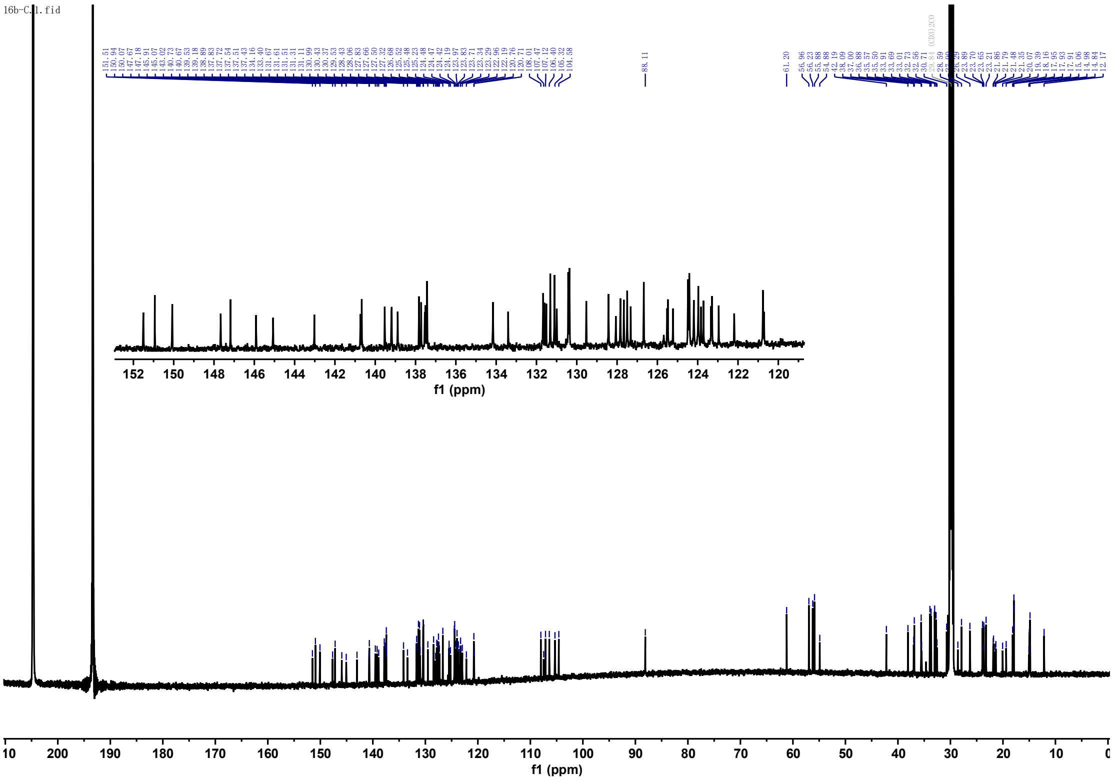

^{1}H NMR spectrum (400 MHz,$CDCl_3$, 298 K) of compound **17**

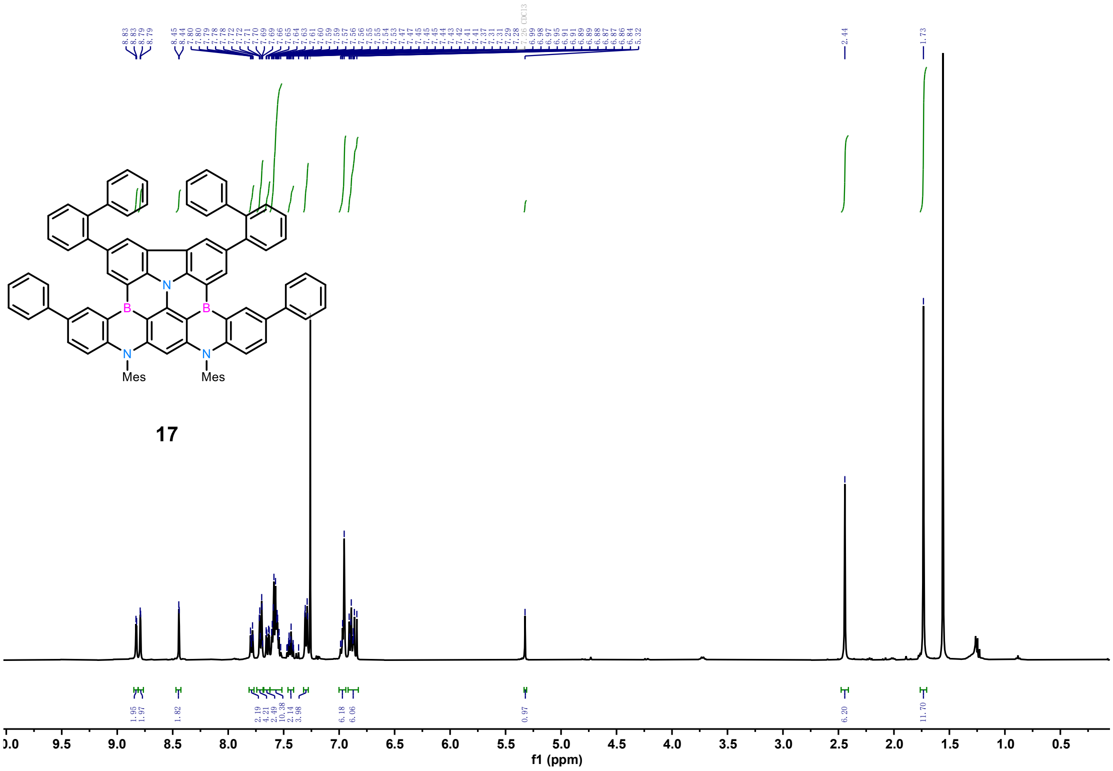

^{13}C NMR spectrum (101 MHz, $CDCl_3$, 298 K) of compound **17**

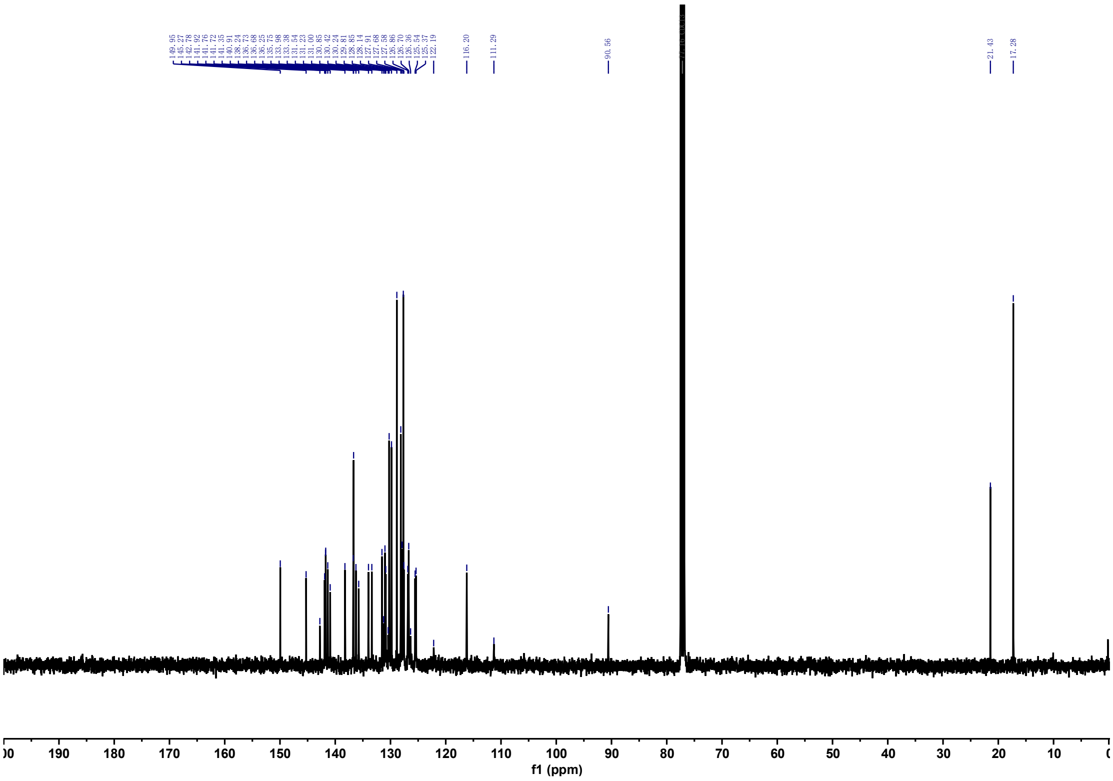

^{1}H NMR spectrum (600 MHz, Acetone-*d6*/CS_2, 298 K) of compound **18**

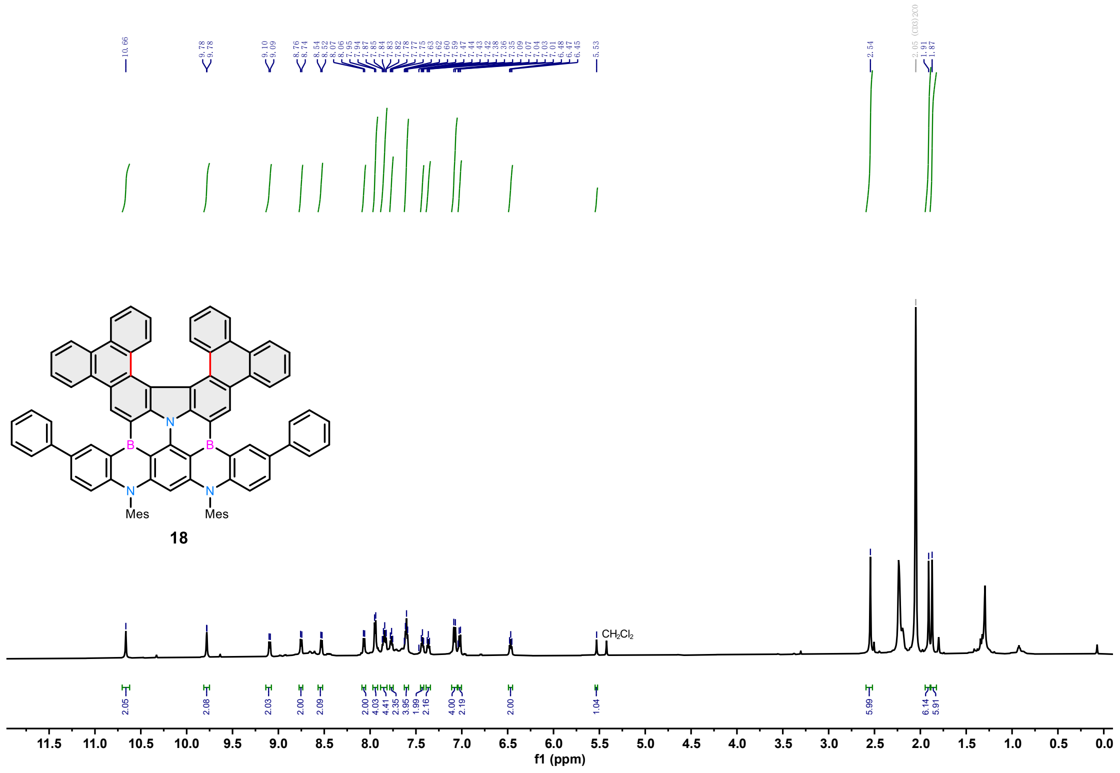

^{13}C NMR spectrum (151 MHz, Acetone-*d6*/CS_2, 298 K) of compound **18**

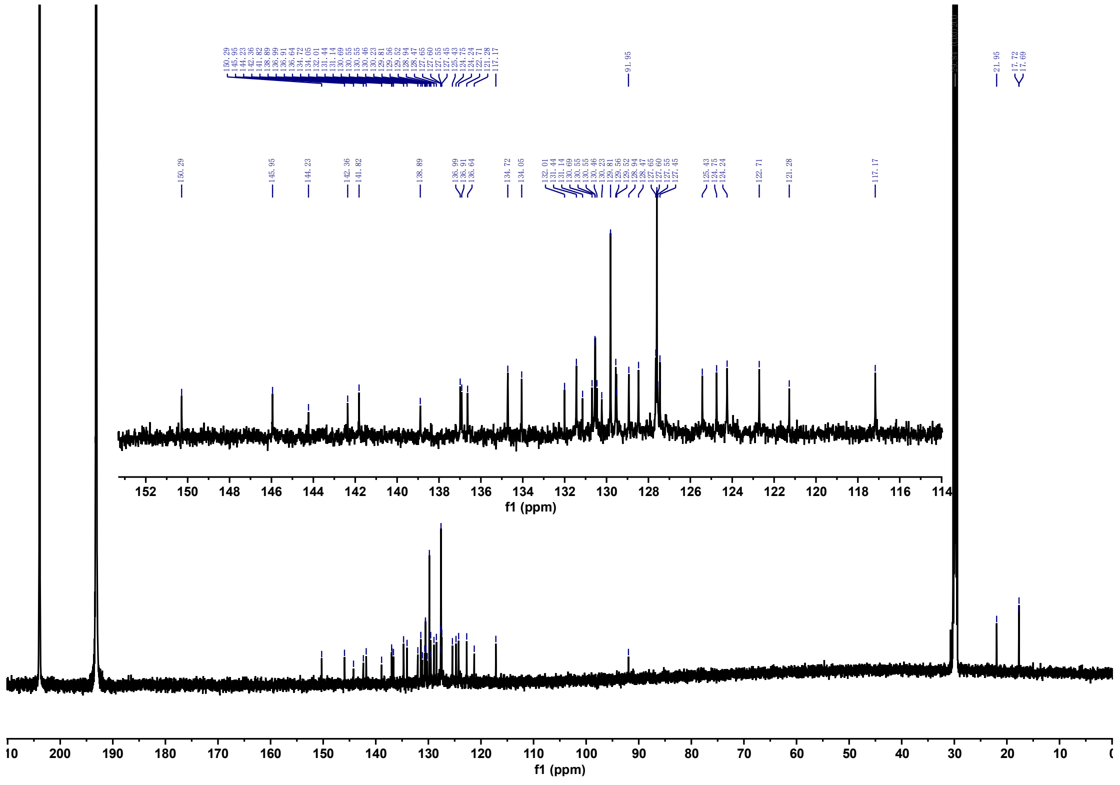

^{1}H NMR spectrum (600 MHz, Acetone-*d6*/CS_2, 298 K) of compound **19**

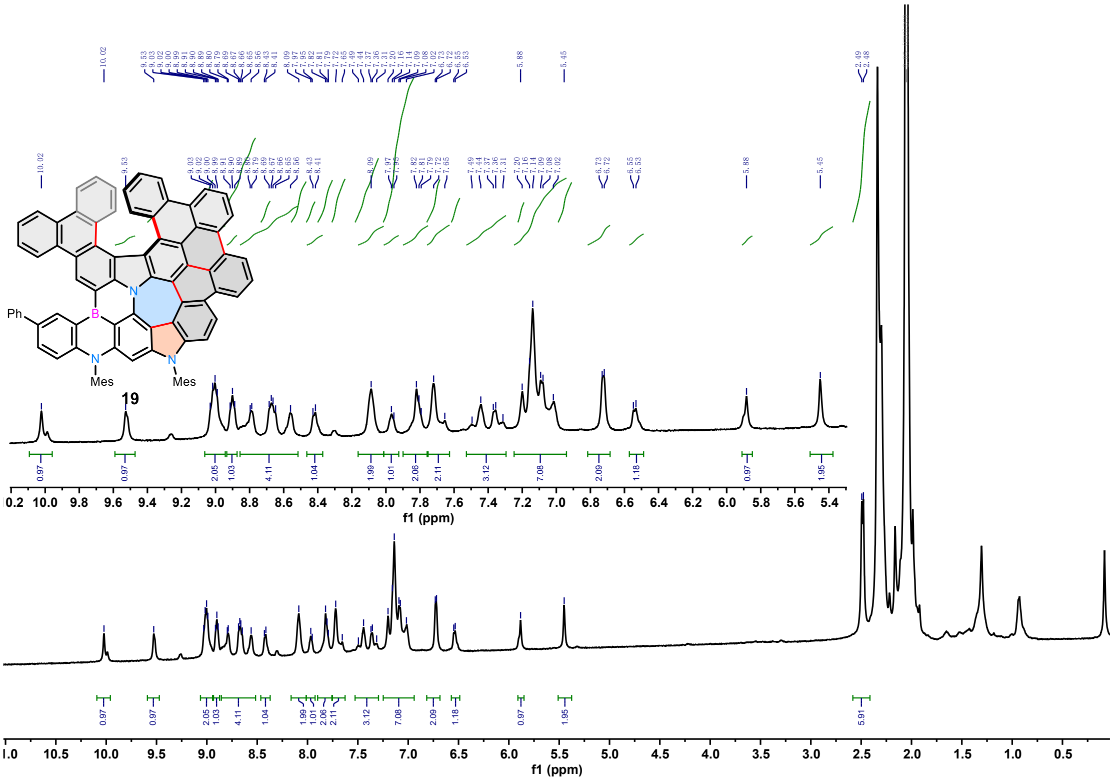

^{13}C NMR spectrum (151 MHz, Acetone-*d6*/CS_2, 298 K) of compound **19**

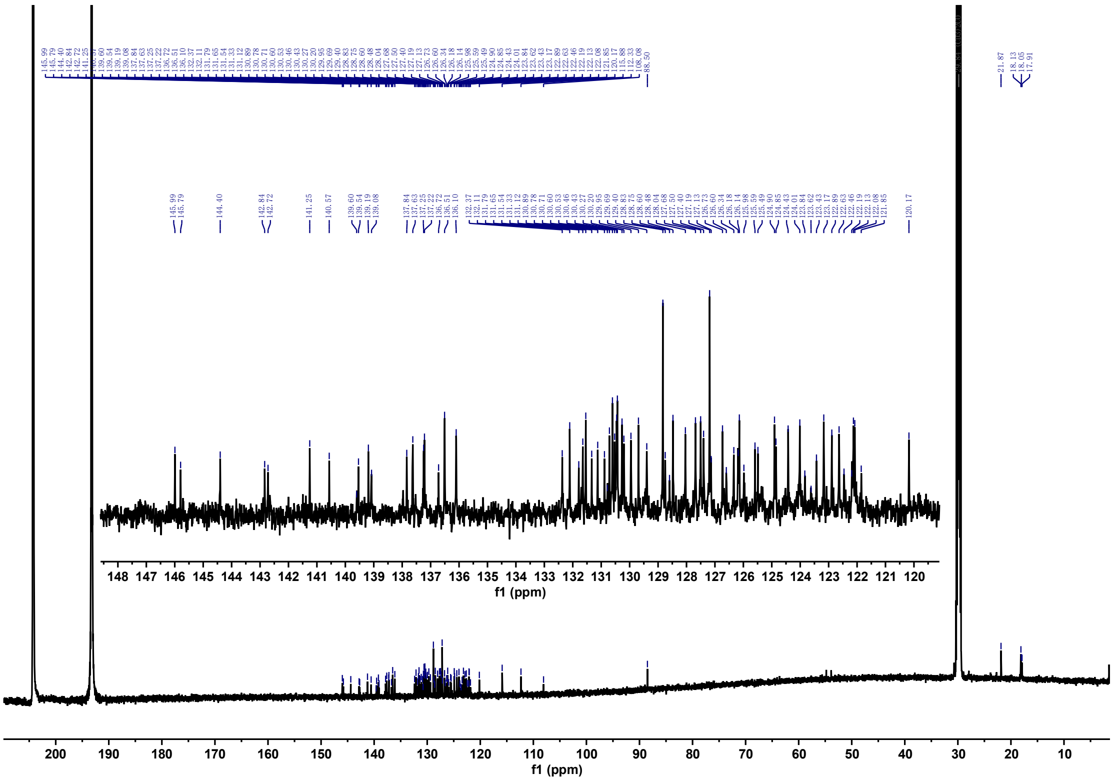

^{1}H NMR spectrum (400 MHz, $CDCl_3$, 298 K) of compound **20**

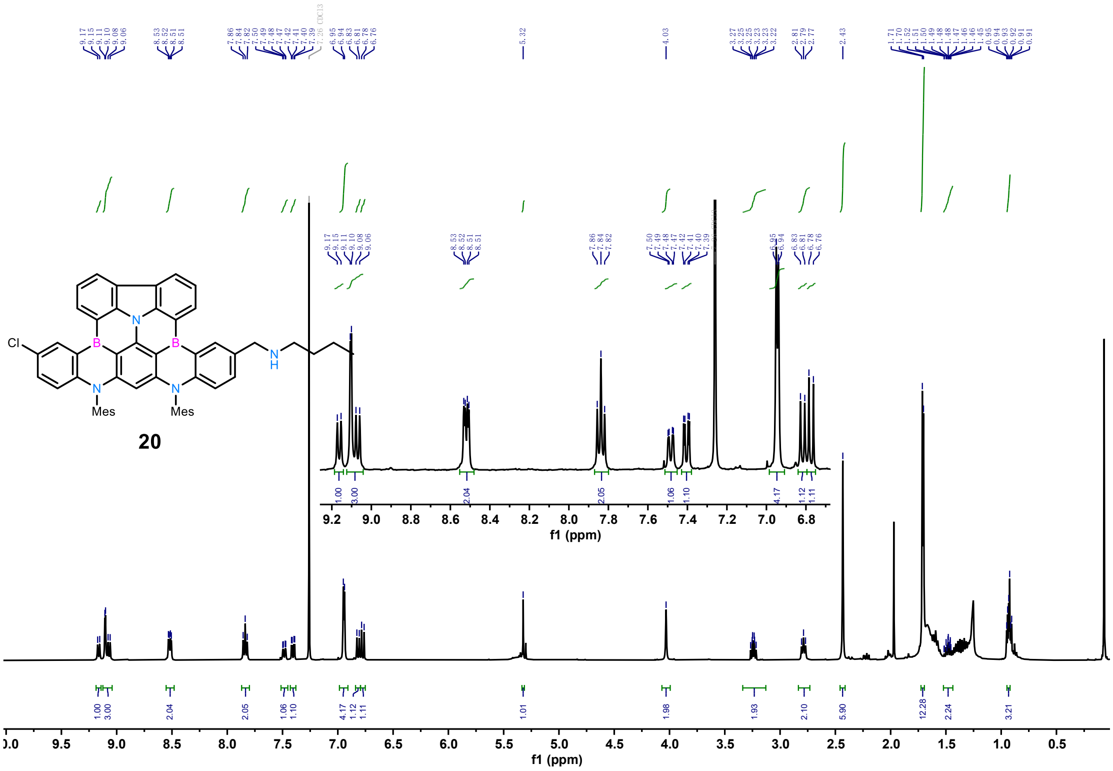

^{13}C NMR spectrum (151 MHz, DMSO-*d6*, 298 K) of compound **20**

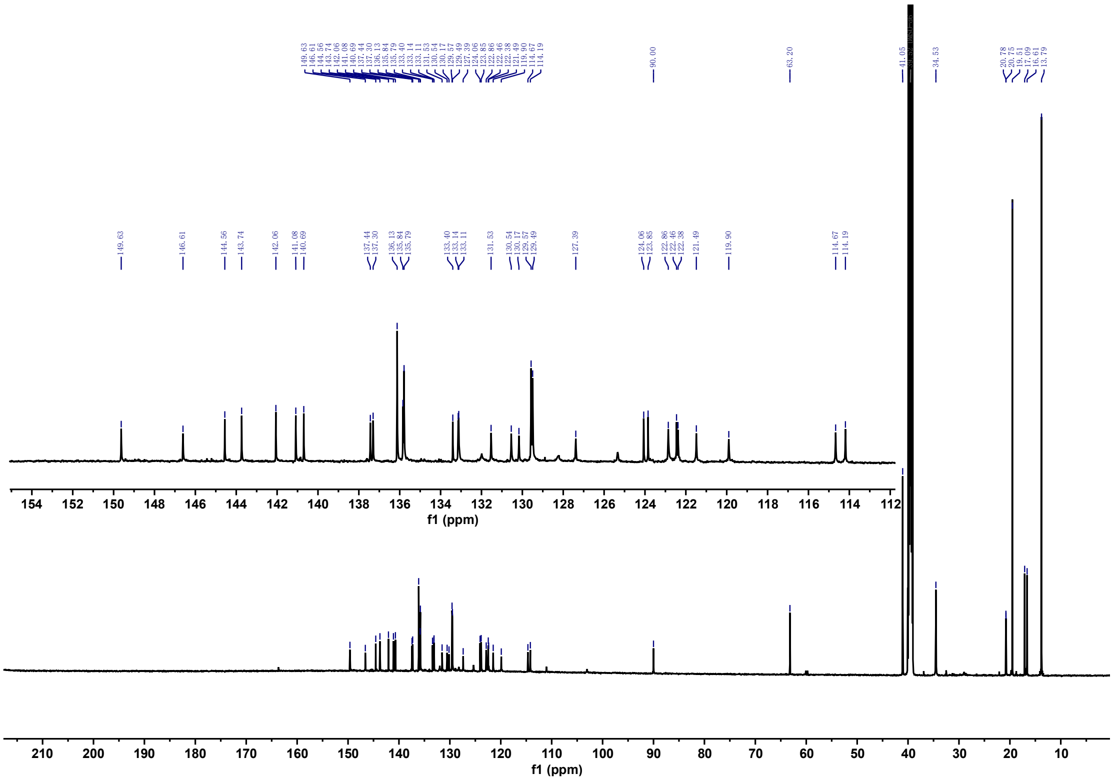

^{1}H NMR spectrum (400 MHz, $CDCl_3$, 298 K) of compound **21**

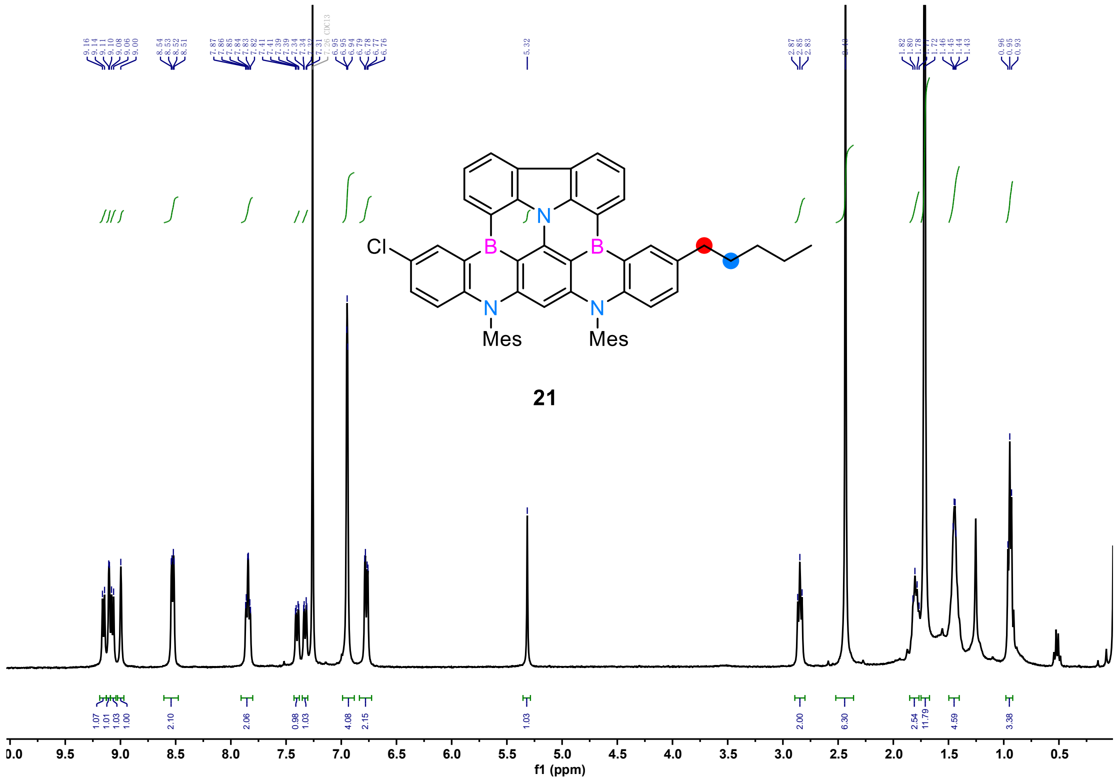

^{13}C NMR spectrum (101 MHz, $CDCl_3$, 298 K) of compound **21**

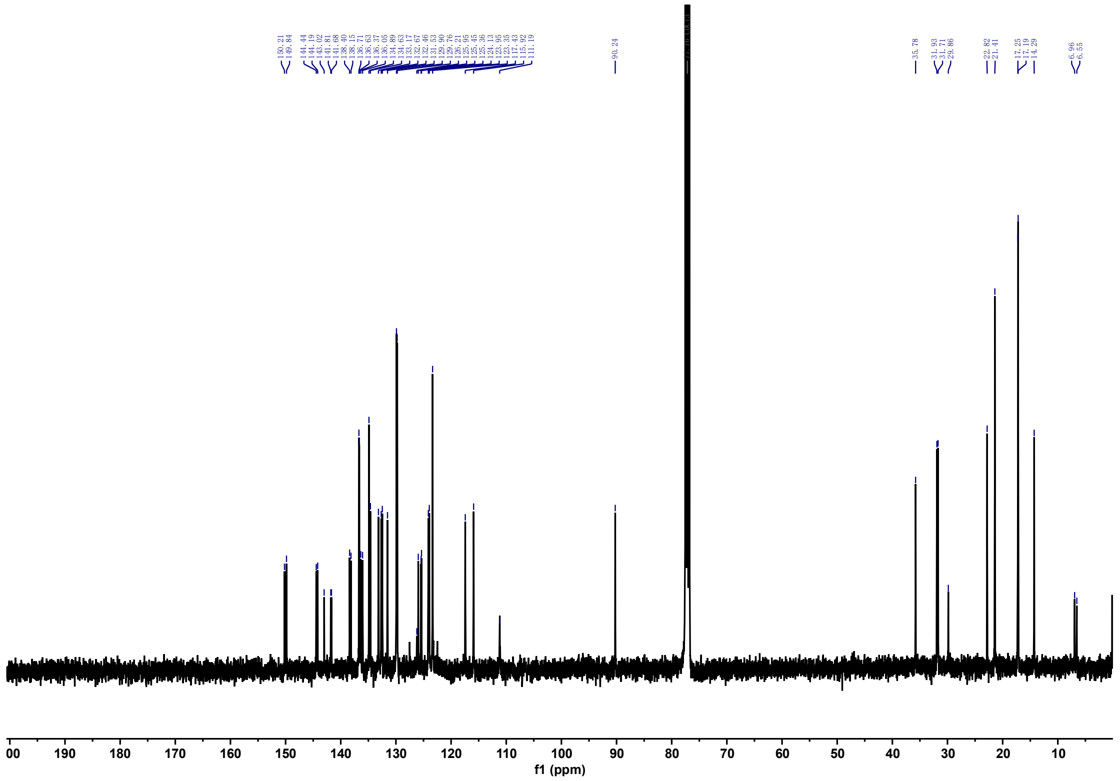

^{1}H NMR spectrum (400 MHz, $CDCl_3$, 298 K) of compound **22**

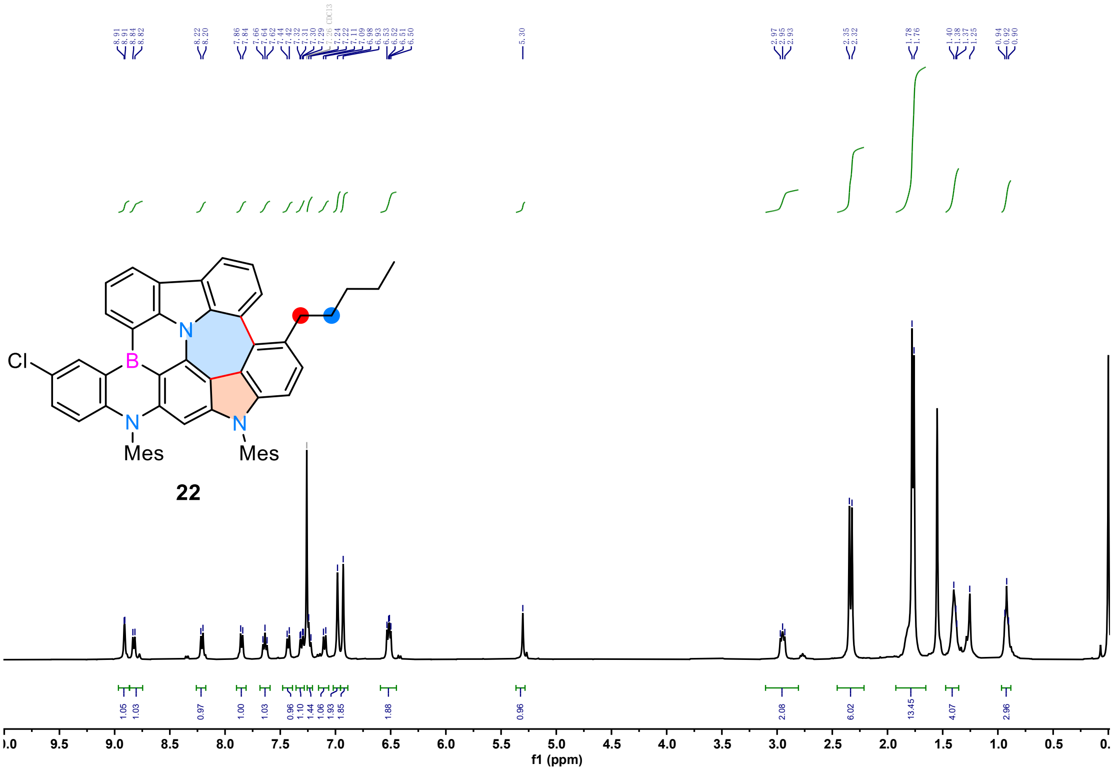

^{13}C NMR spectrum (101 MHz, $CDCl_3$, 298 K) of compound **22**

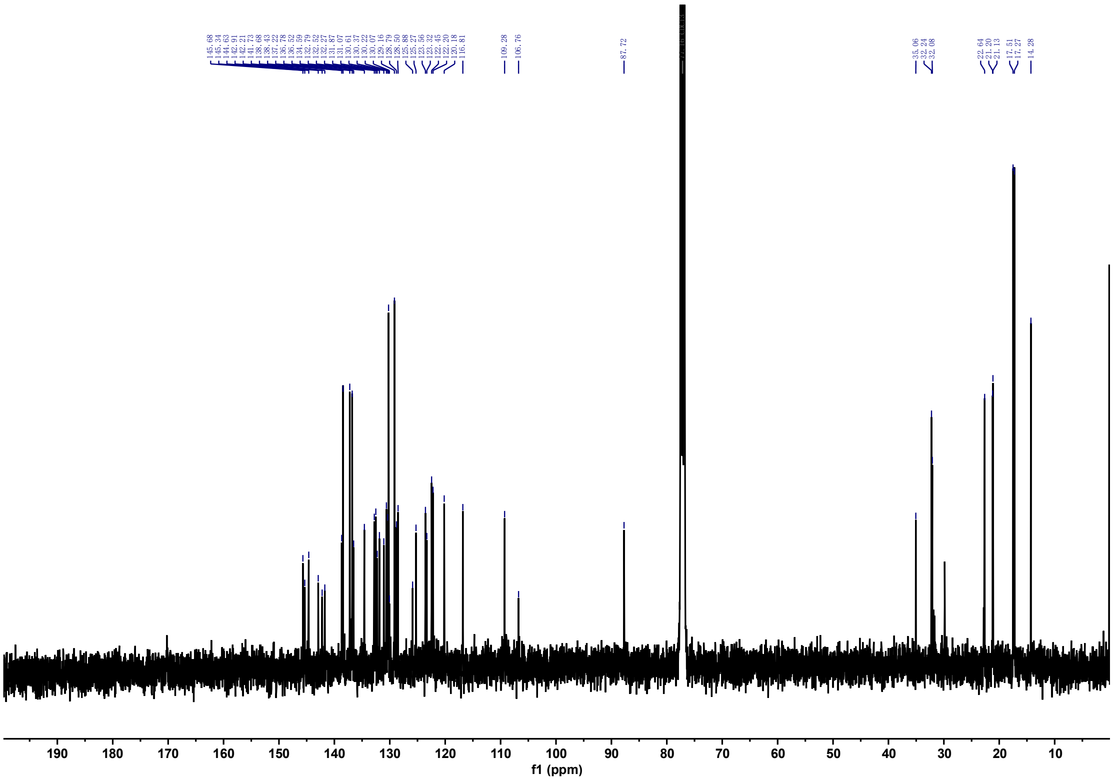

^{1}H NMR spectrum (600 MHz, Acetone-*d6*/CS_2, 298 K) of compound **23**

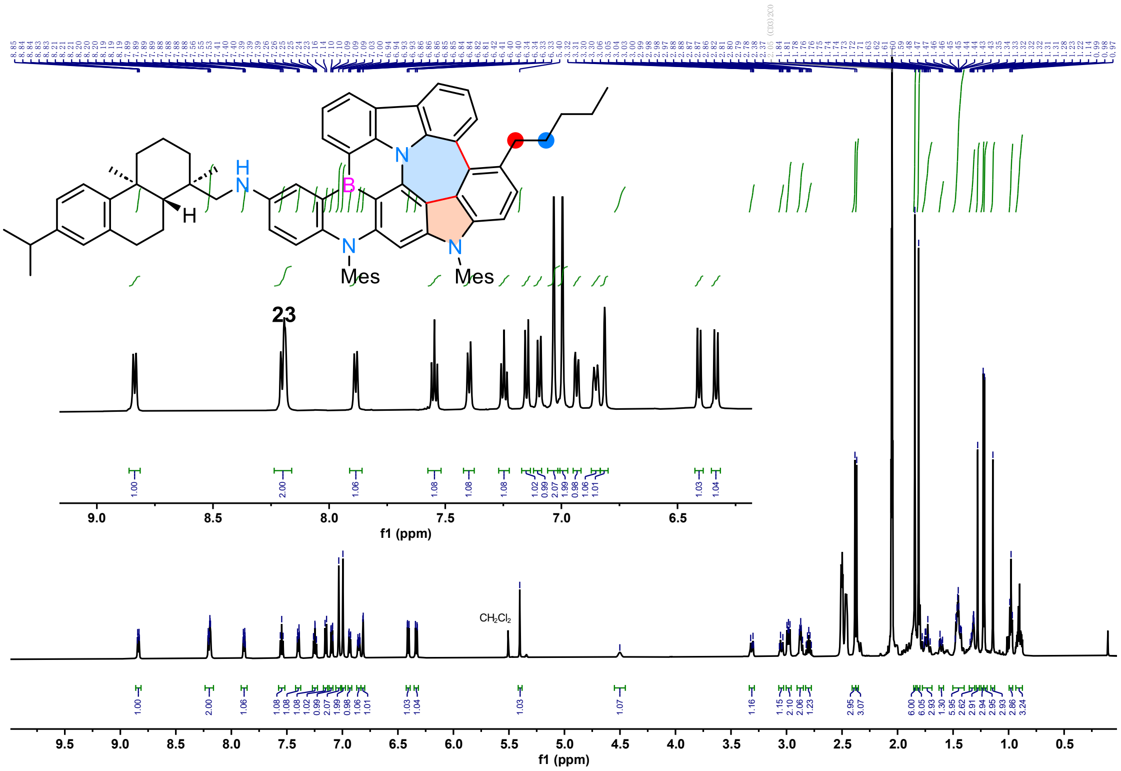

^{13}C NMR spectrum (151 MHz, Acetone-*d6*/CS_2, 298 K) of compound **23**

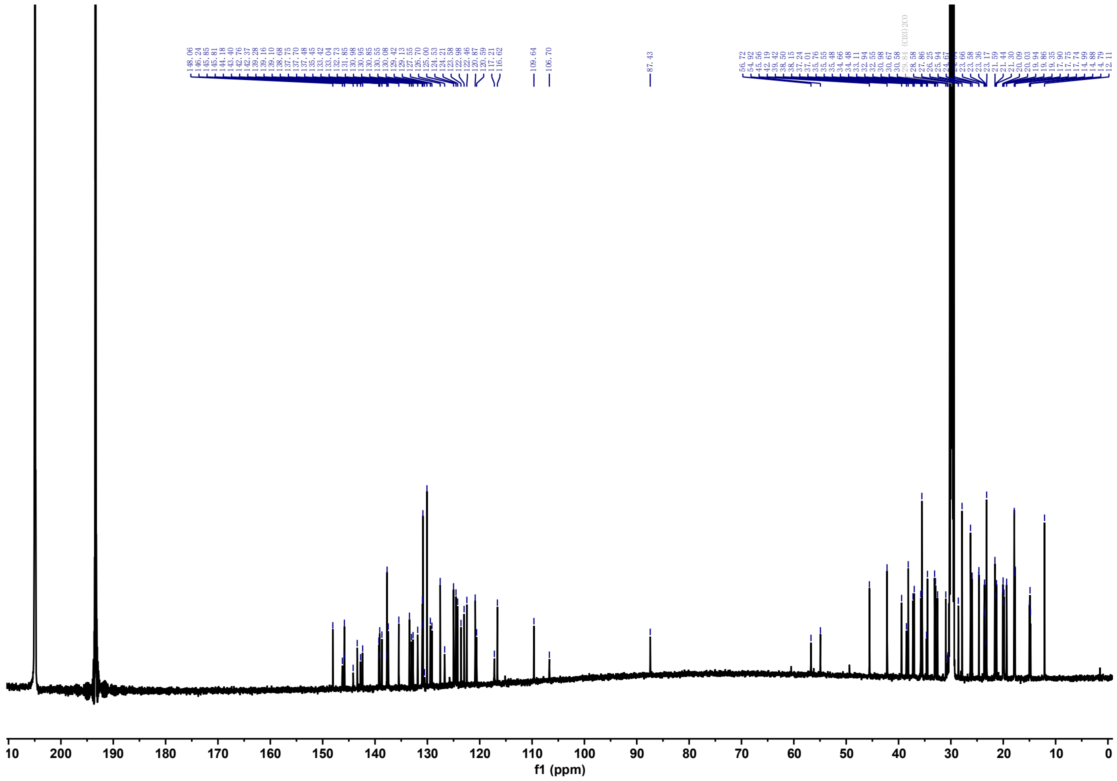

^{1}H NMR spectrum (400 MHz, $CDCl_3$, 298 K) of compound **24a**

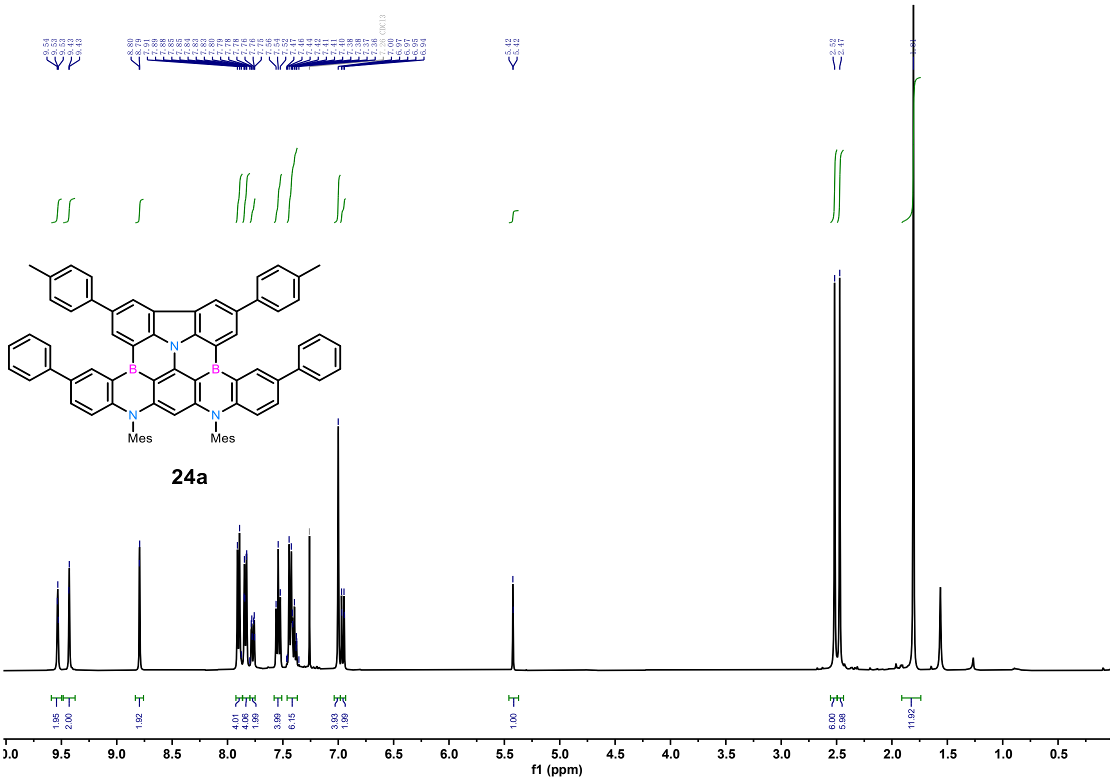

^{13}C NMR spectrum (101 MHz, $CDCl_3$, 298 K) of compound **24a**

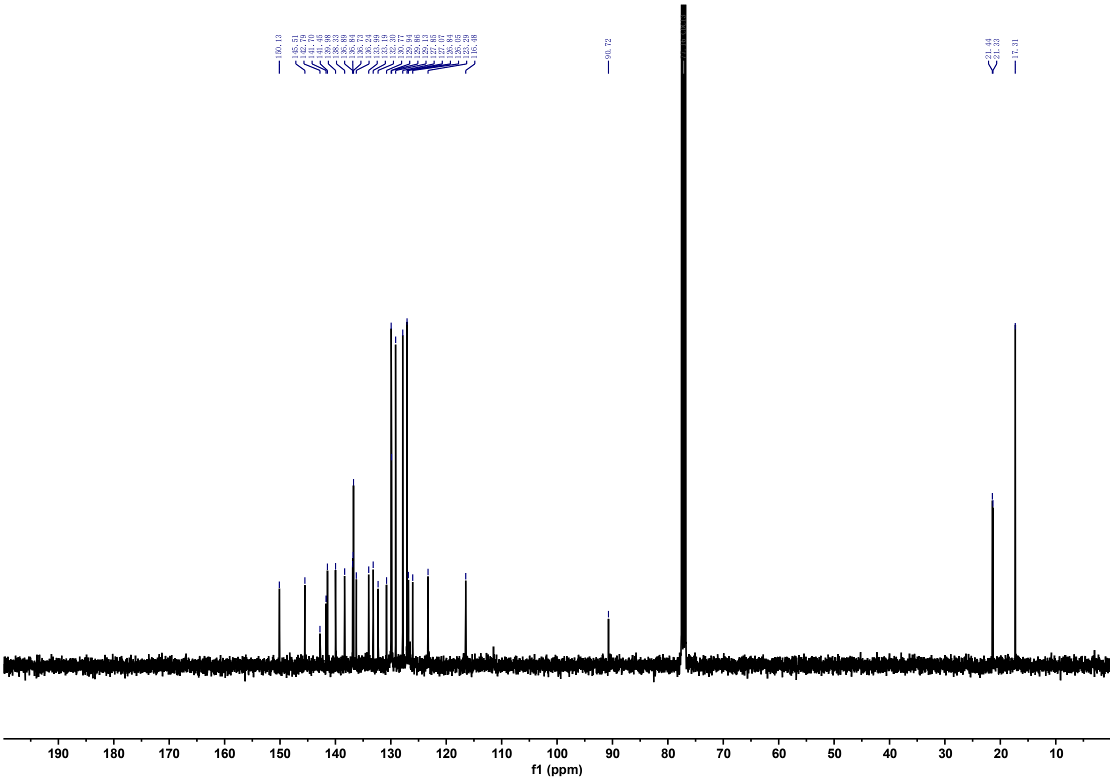

^{1}H NMR spectrum (400 MHz, $CDCl_3$, 298 K) of compound **24b**

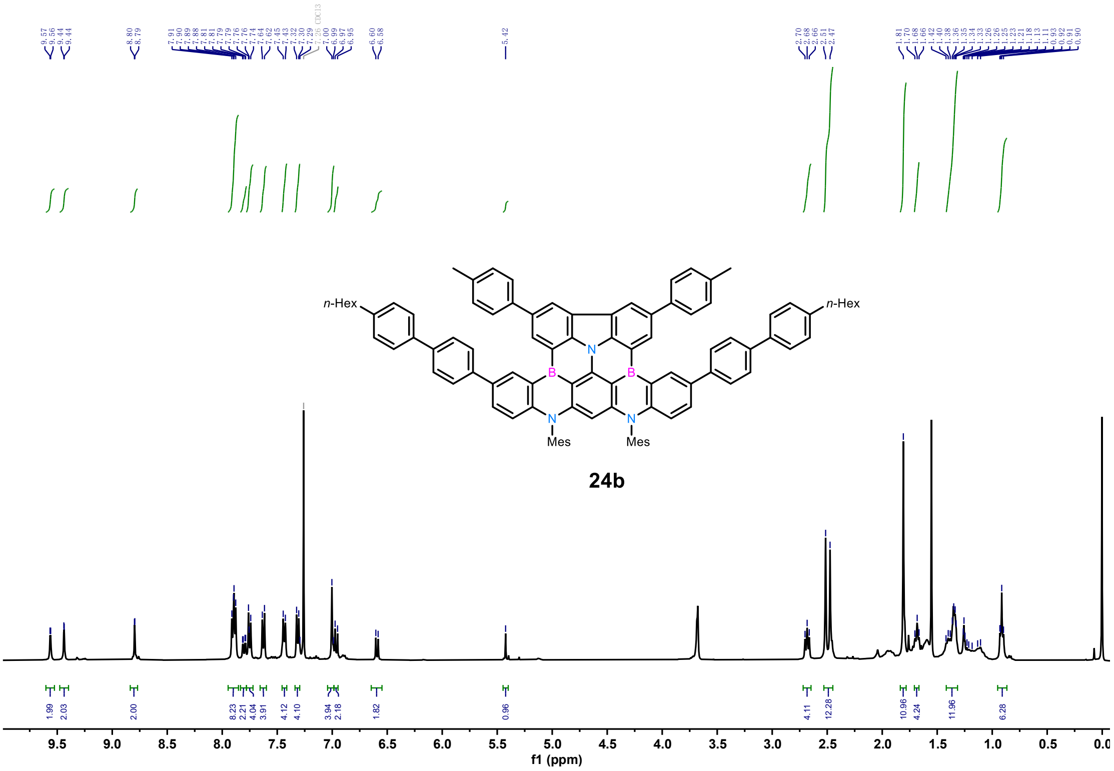

^{13}C NMR spectrum (101 MHz, $CDCl_3$, 298 K) of compound **24b**

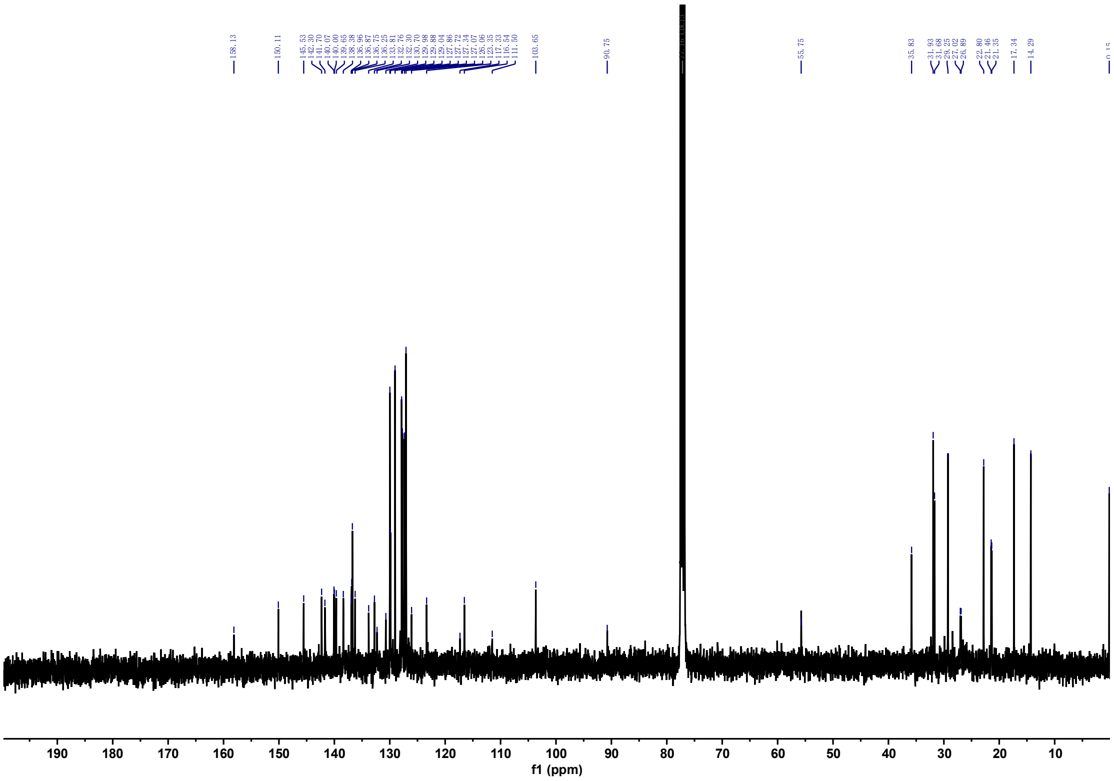

^{1}H NMR spectrum (600 MHz, Acetone-*d6*/CS_2, 298 K) of compound **25a**

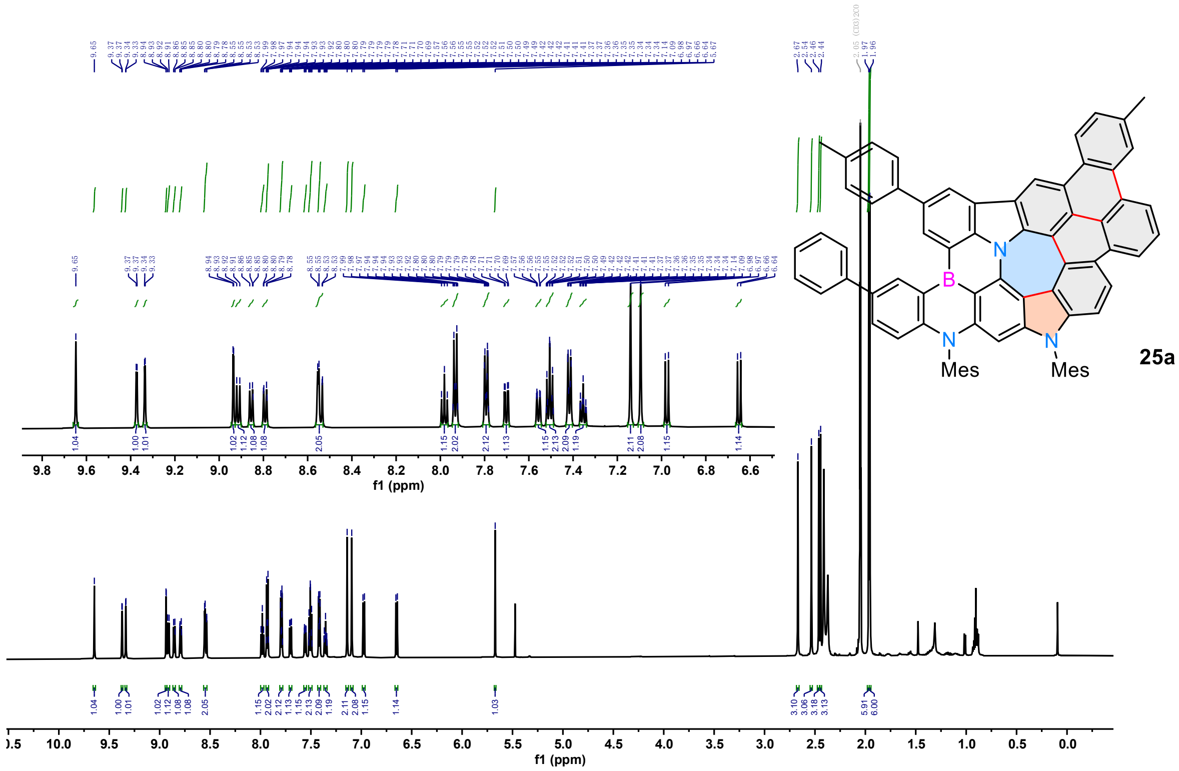

^{13}C NMR spectrum (151 MHz, Acetone-*d6*/CS_2, 298 K) of compound **25a**

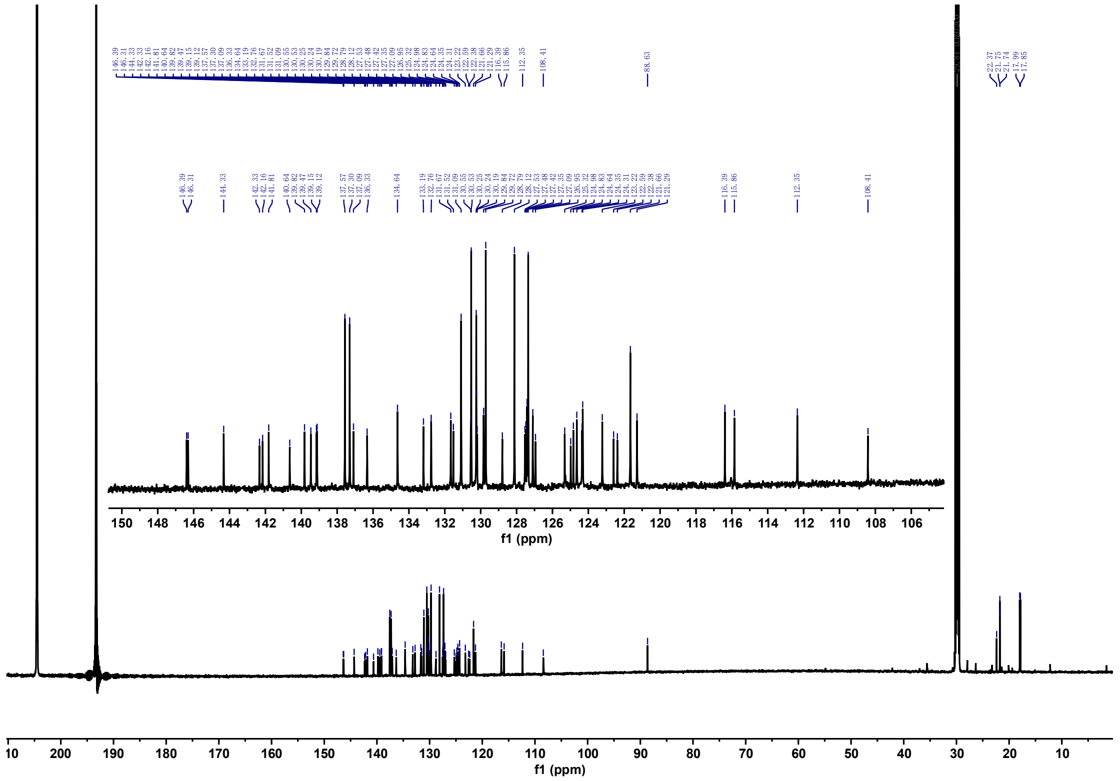

^{1}H NMR spectrum (600 MHz, Acetone-*d6*/CS_2, 298 K) of compound **25b**

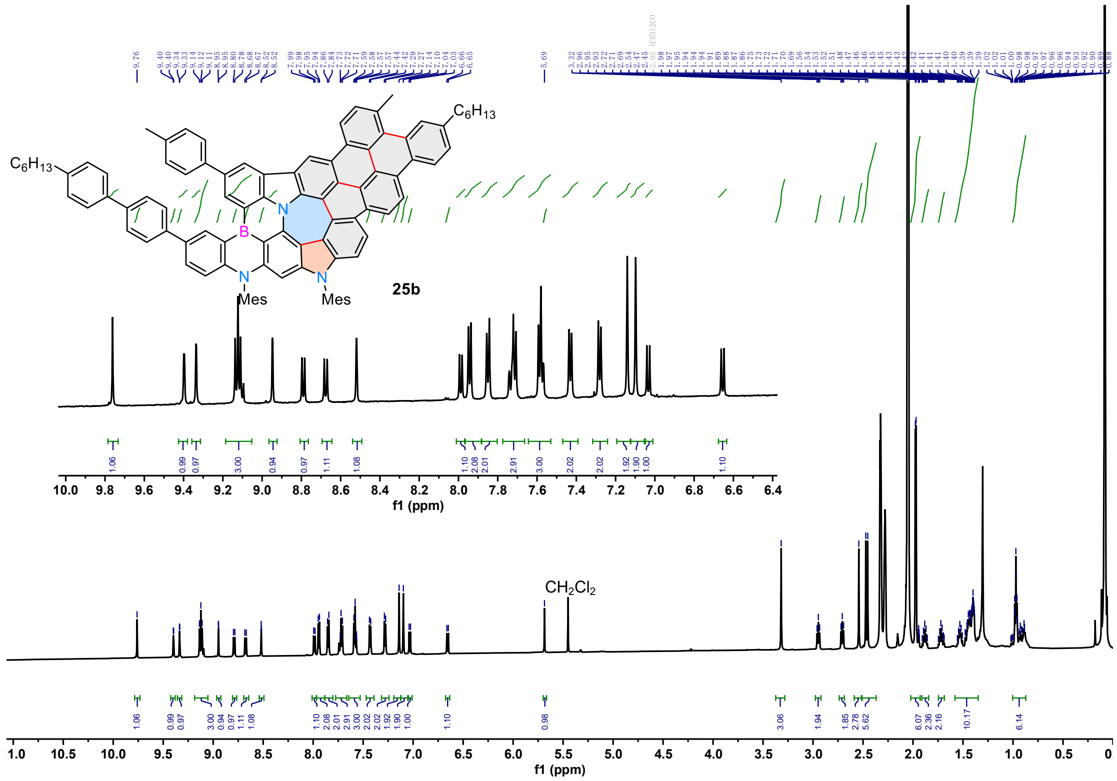

^{13}C NMR spectrum (151 MHz, Acetone-*d6*/CS_2, 298 K) of compound **25b**

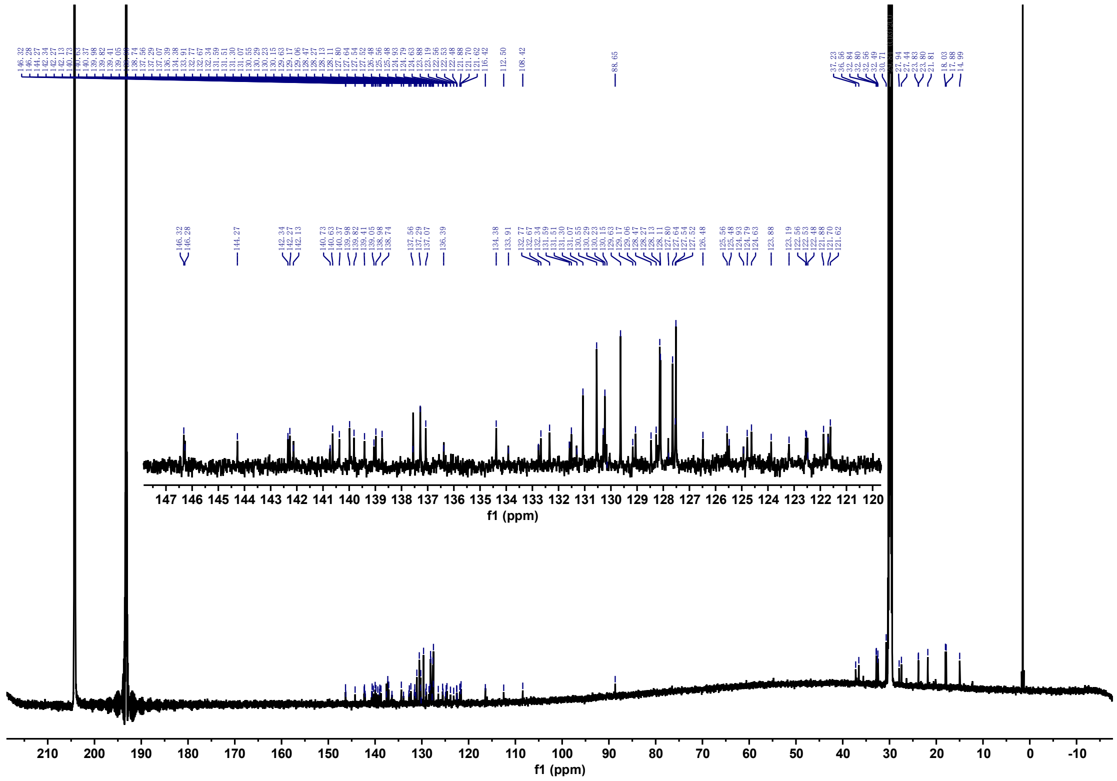

^{1}H NMR spectrum (600 MHz, C_6D_6, 298 K) of compound **26**

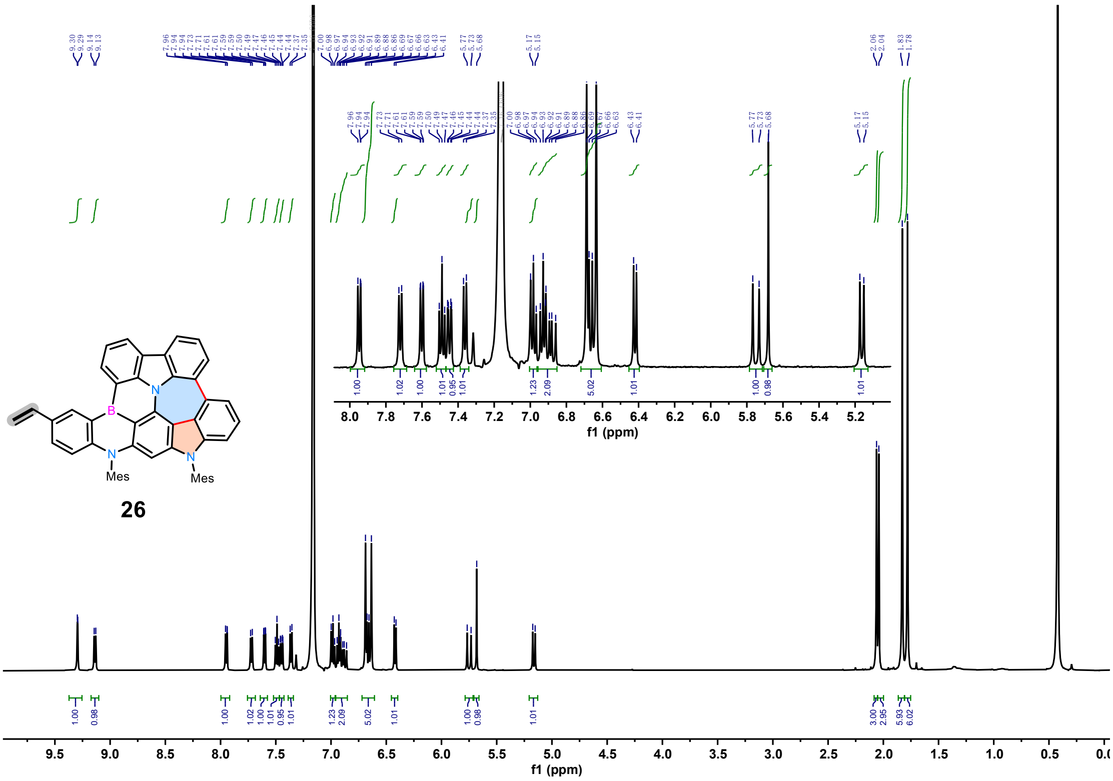

^{13}C NMR spectrum (151 MHz, C_6D_6, 298 K) of compound **26**

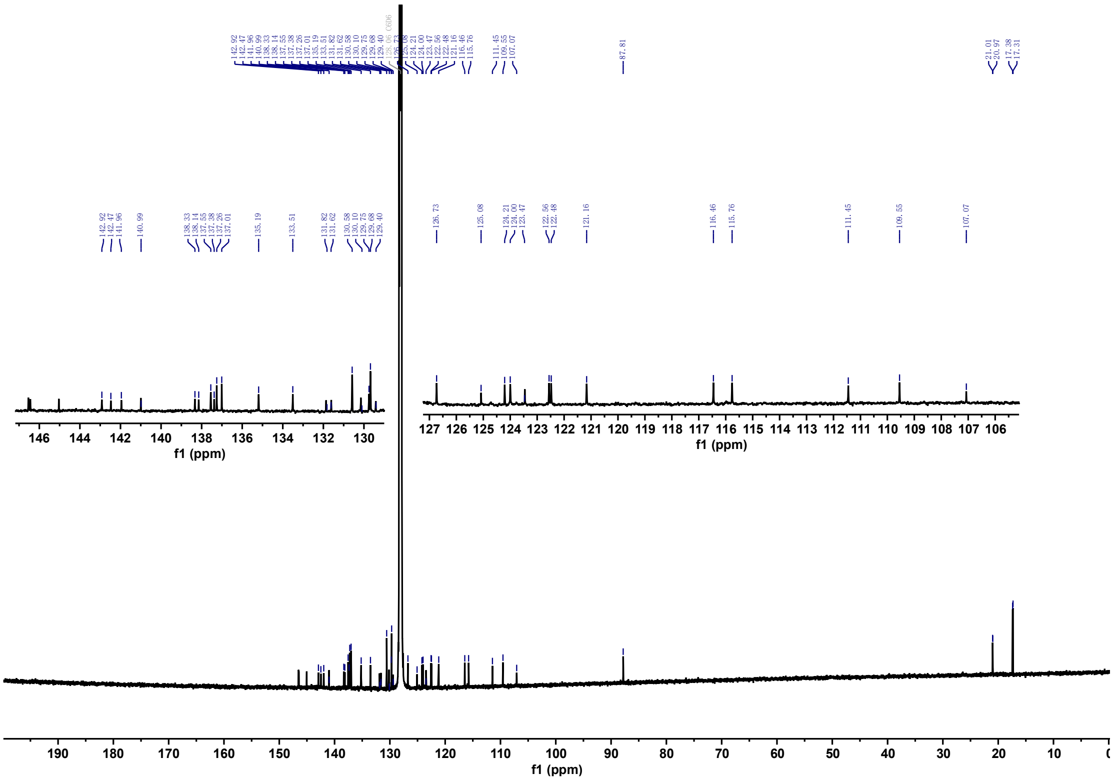

^{1}H NMR spectrum (400 MHz, $CDCl_3$, 298 K) of compound **27**

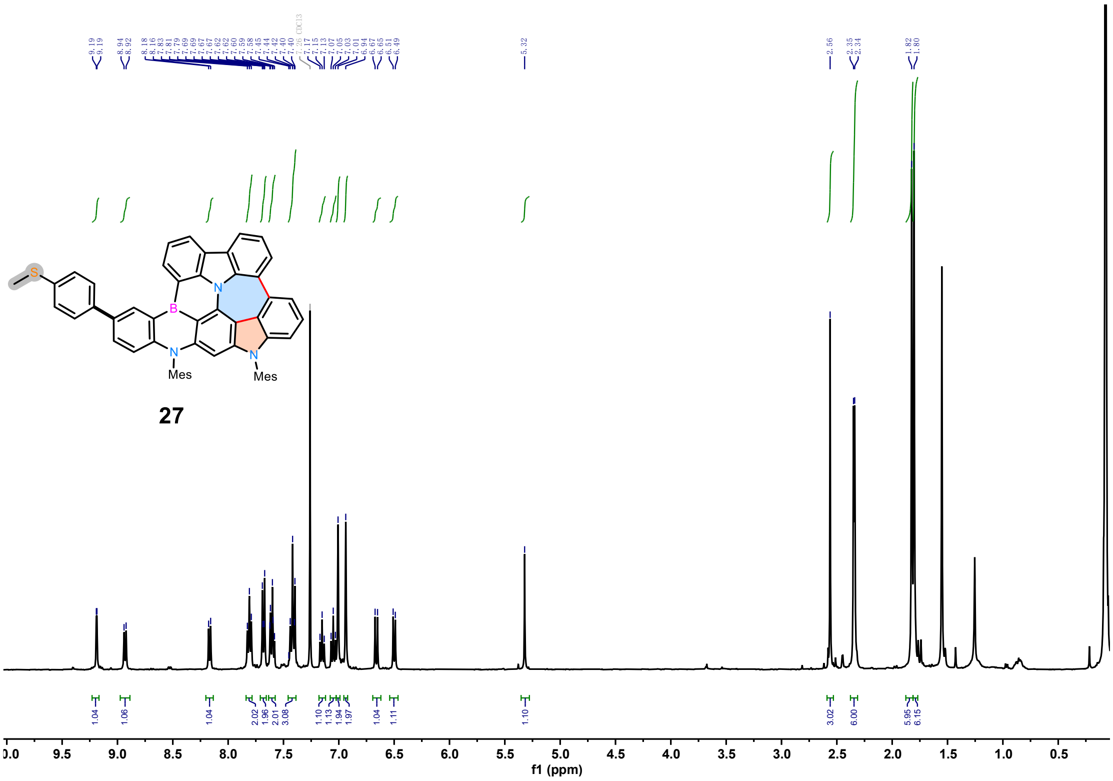

^{13}C NMR spectrum (101 MHz, $CDCl_3$, 298 K) of compound **27**

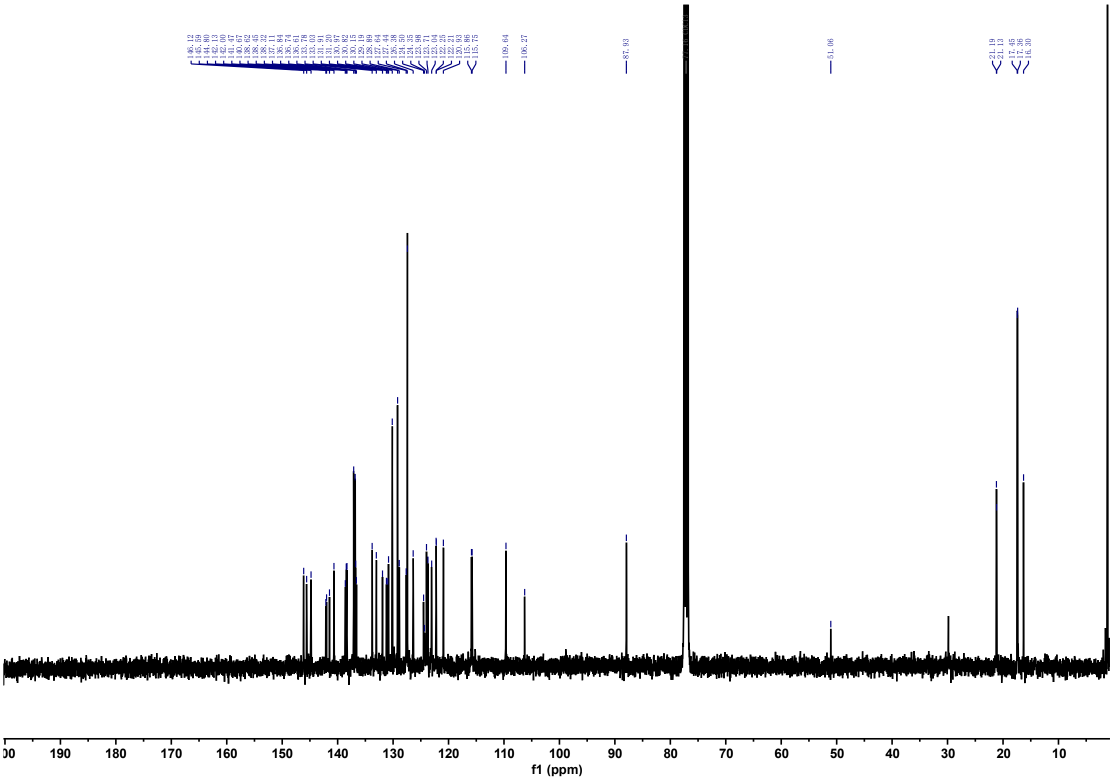

^{1}H NMR spectrum (600 MHz, Acetone-*d6*/CS_2, 298 K) of compound **28**

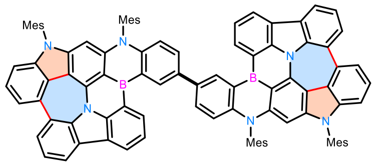

28

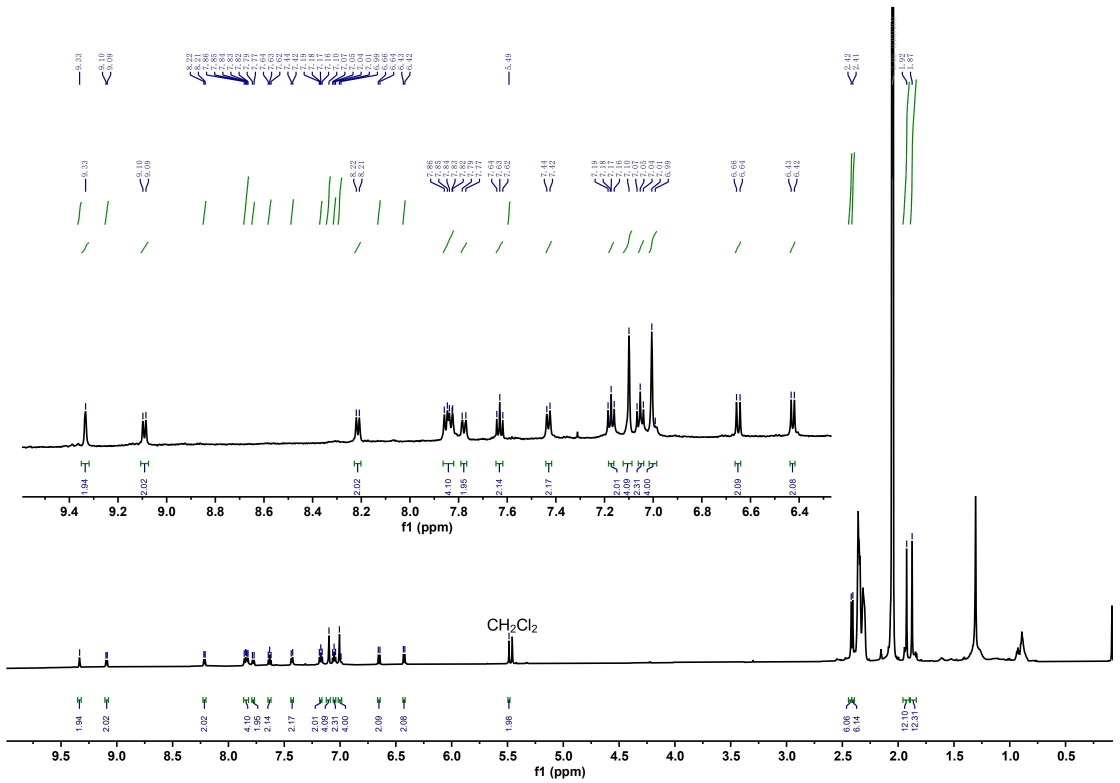

^{1}H NMR spectrum (400 MHz, DMSO-*d6*, 298 K) of compound **29a**

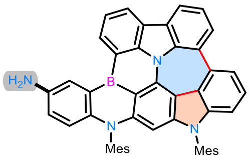

29a

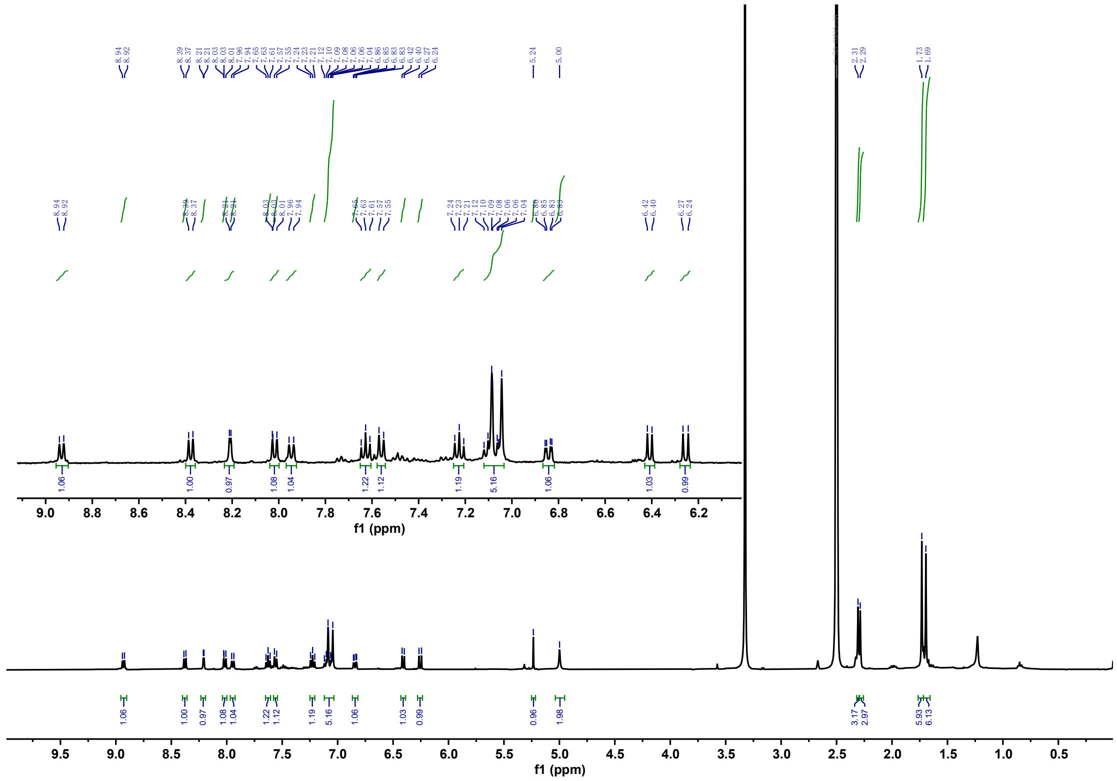

^{1}H NMR spectrum (400 MHz, Acetone-*d6*/DMSO-*d6*/CS_2, 298 K) of compound **29b**

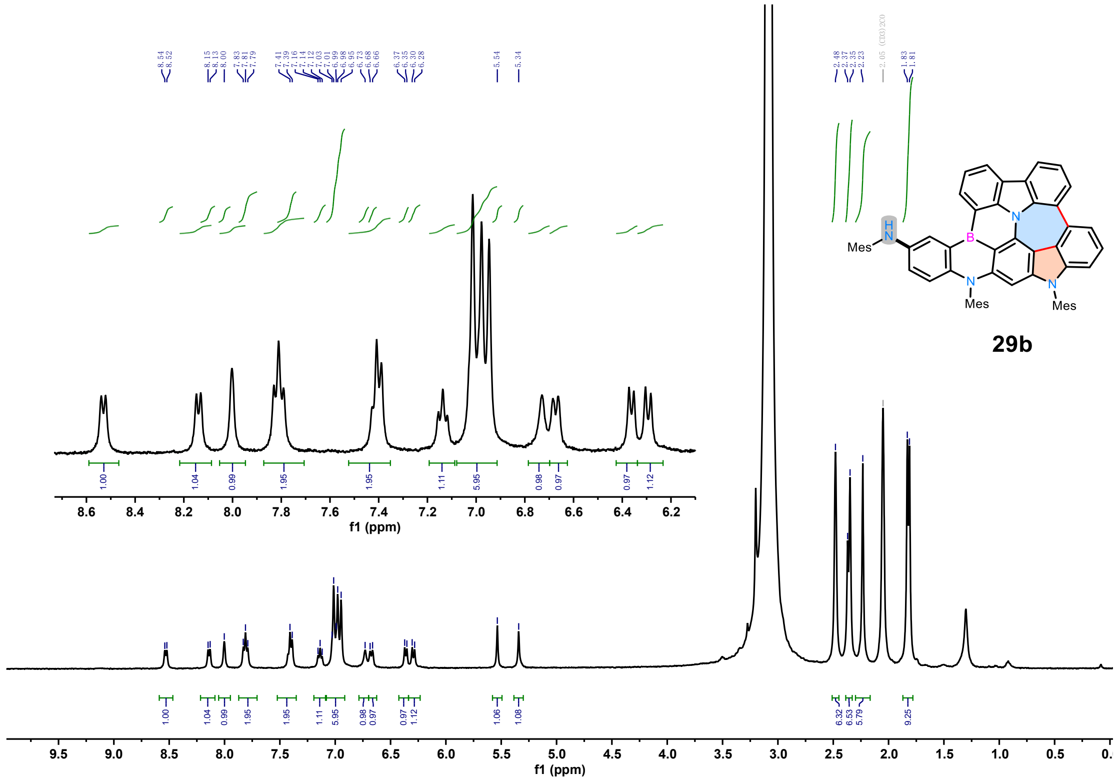

^{13}C NMR spectrum (151 MHz, Acetone-*d6*/DMSO-*d6*/CS_2, 298 K) of compound **29b**

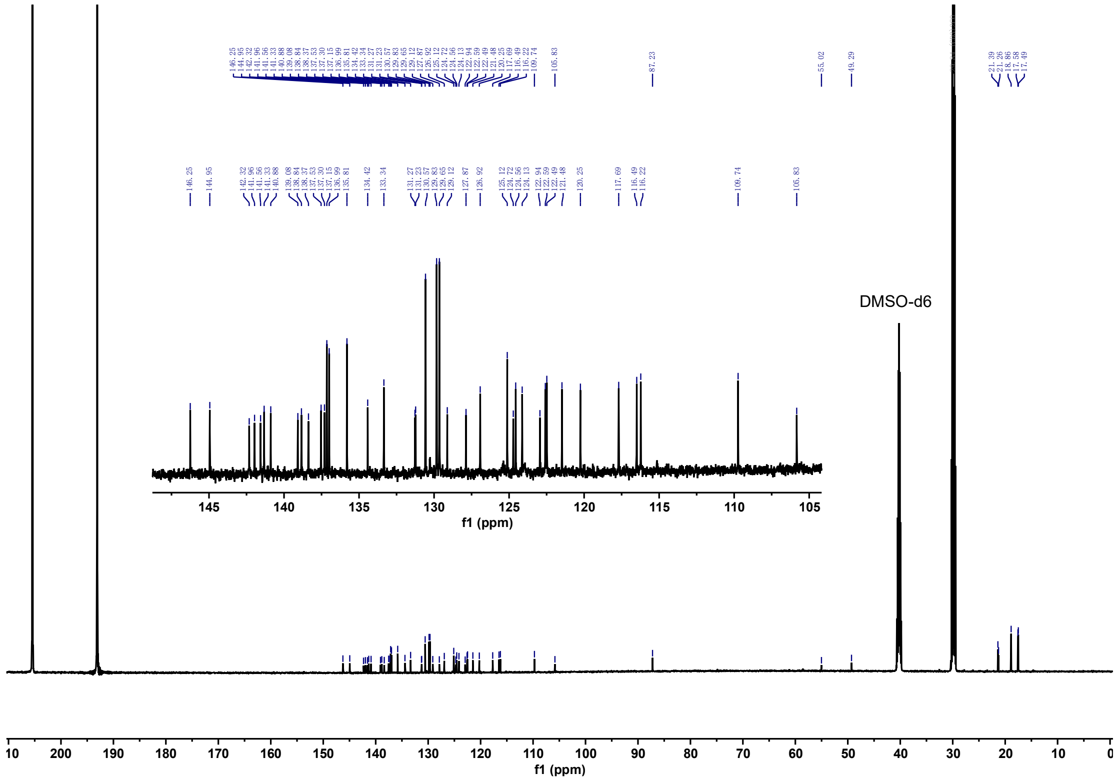

^{1}H NMR spectrum (400 MHz, Acetone-*d6*/CS_2, 298 K) of compound **30**

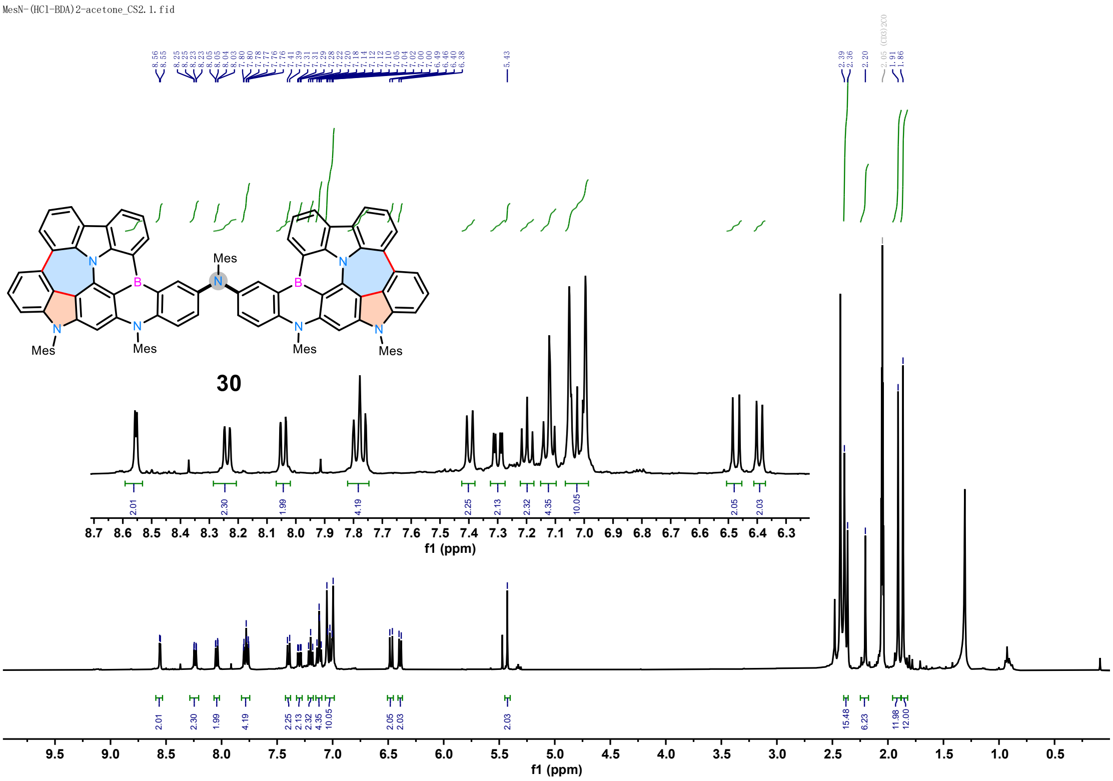

^{13}C NMR spectrum (151 MHz, Acetone-*d6*/CS_2, 298 K) of compound **30**

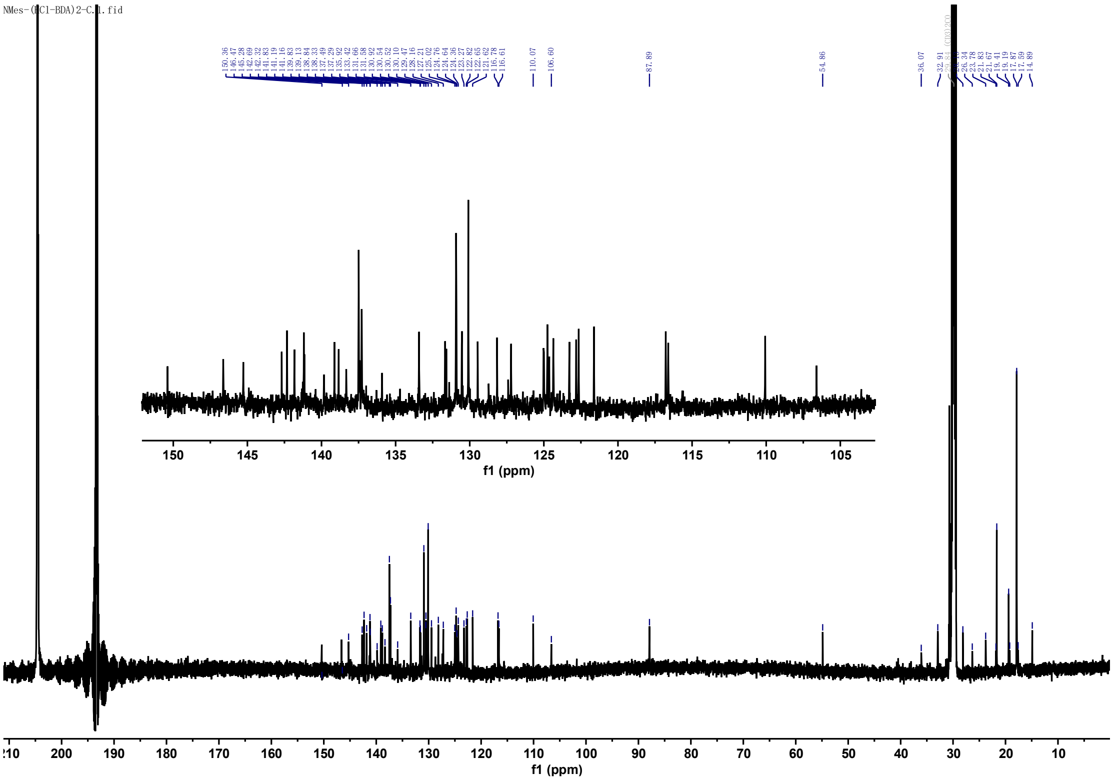

^{1}H NMR spectrum (400 MHz, $CDCl_3$, 298 K) of compound **31**

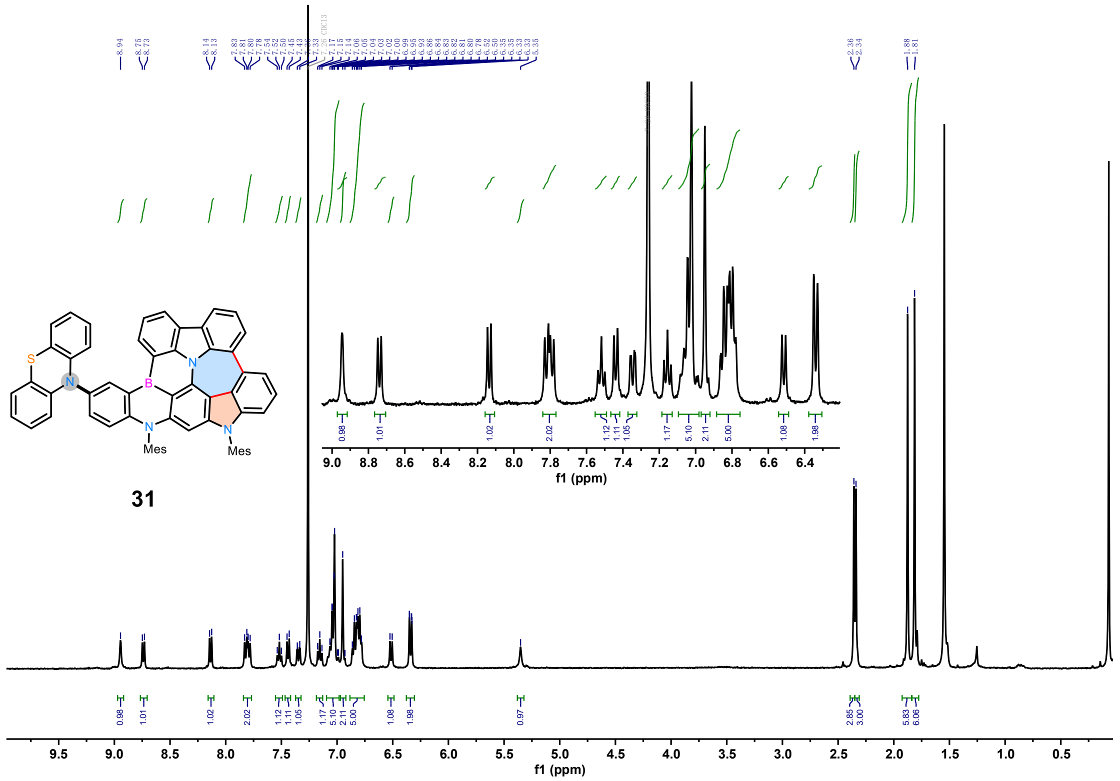

^{13}C NMR spectrum (151 MHz, Acetone-*d6*/CS_2, 298 K) of compound **31**

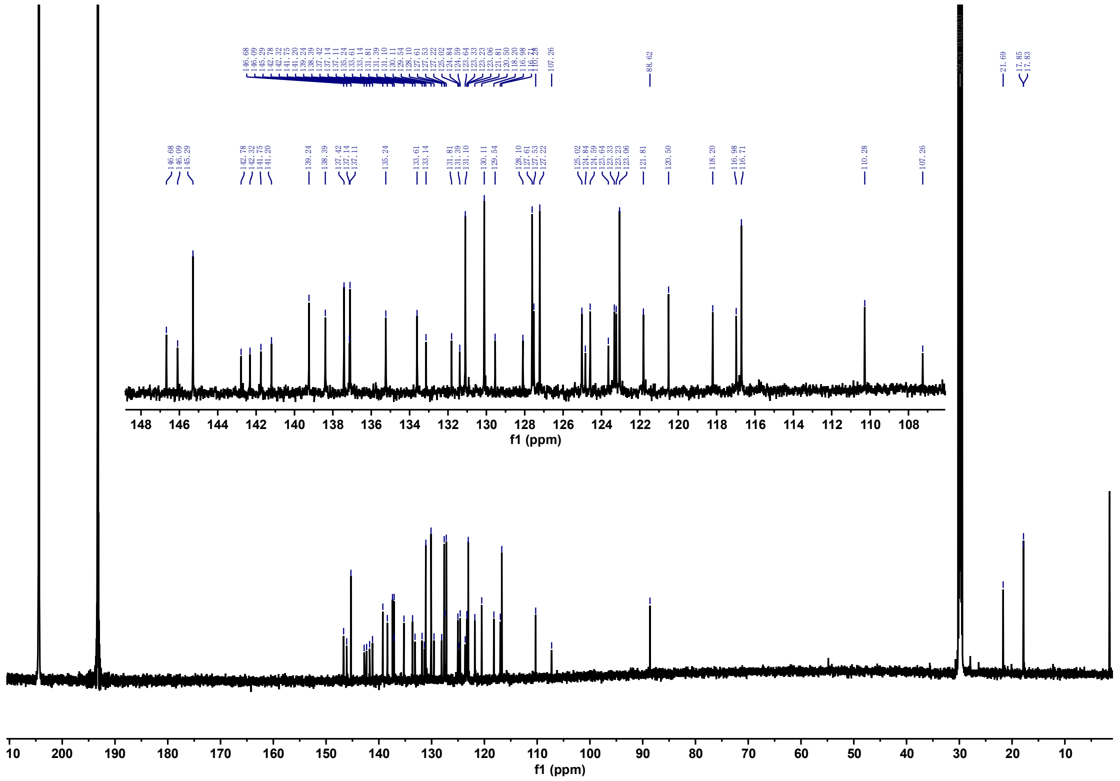

^{1}H NMR spectrum (600 MHz, Acetone-*d6*/CS_2, 298 K) of compound **32**

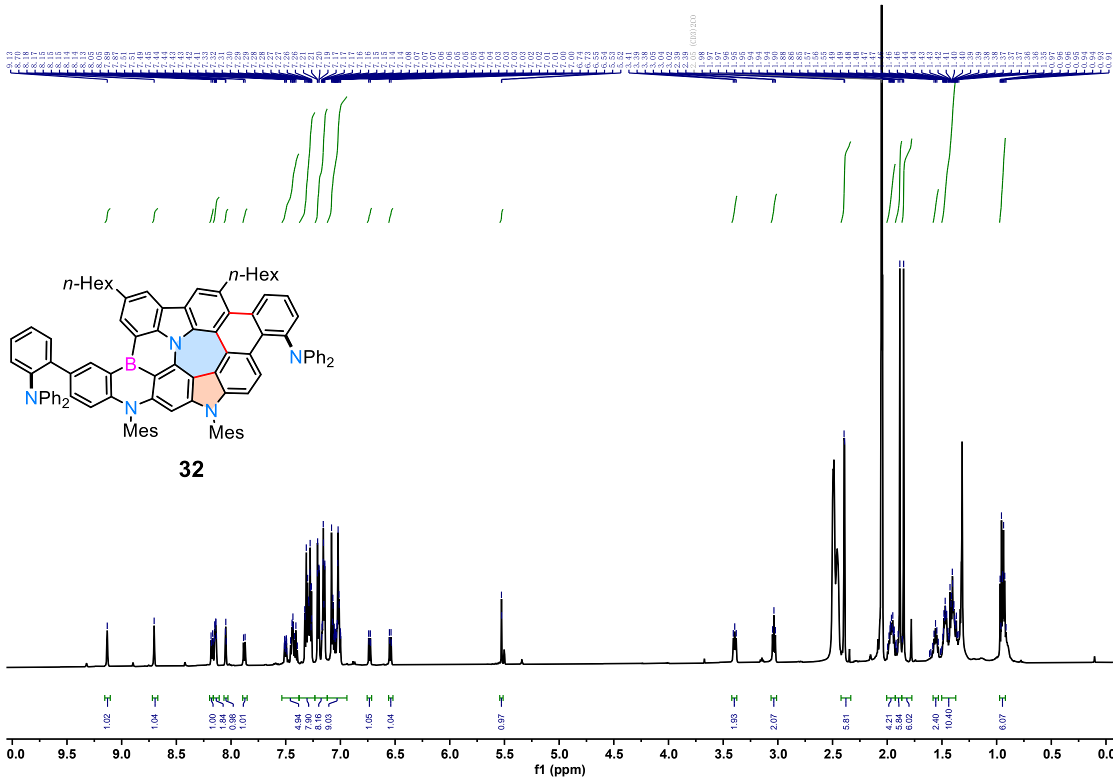

^{13}C NMR spectrum (151 MHz, Acetone-*d6*/CS_2, 298 K) of compound **32**

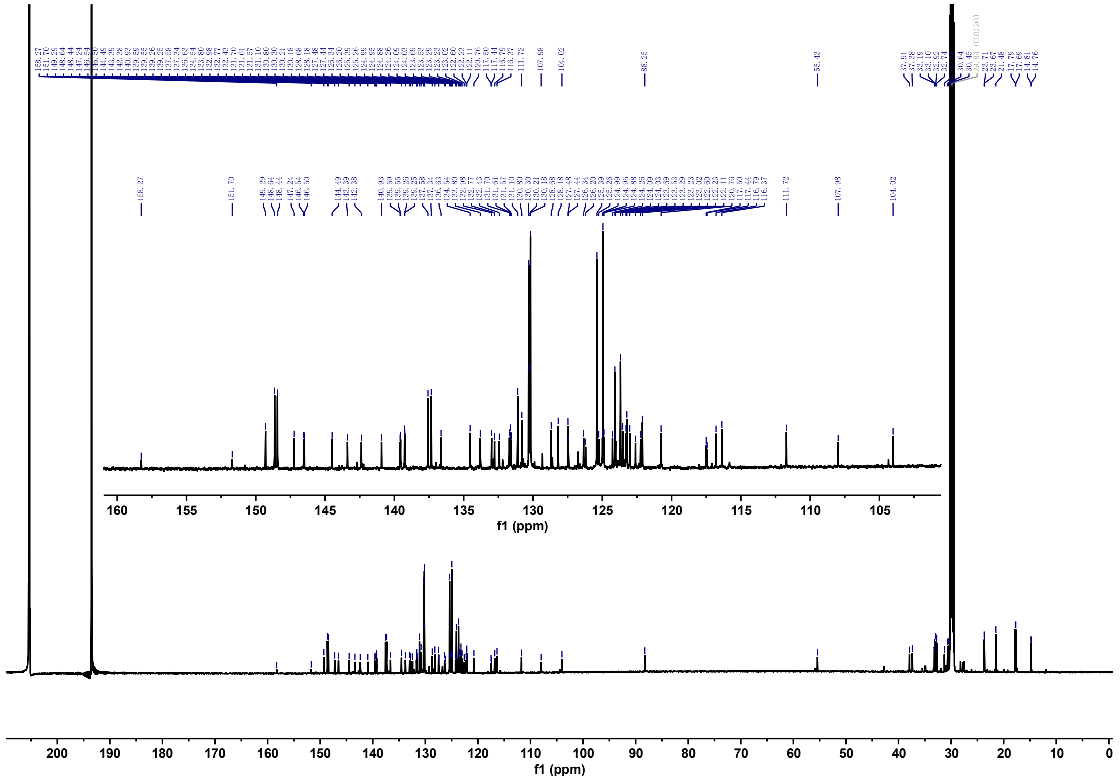

^{1}H NMR spectrum (600 MHz, Acetone-*d6*/CS_2, 298 K) of compound **33**

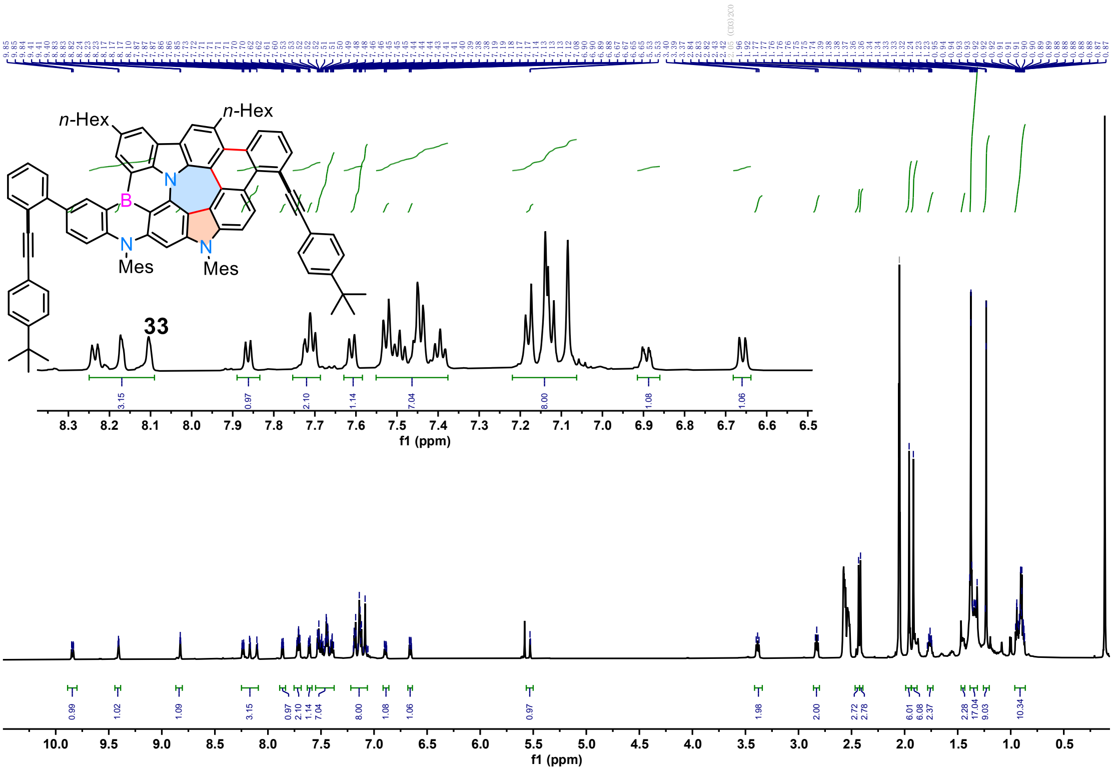

^{13}C NMR spectrum (151 MHz, Acetone-*d6*/CS_2, 298 K) of compound **33**

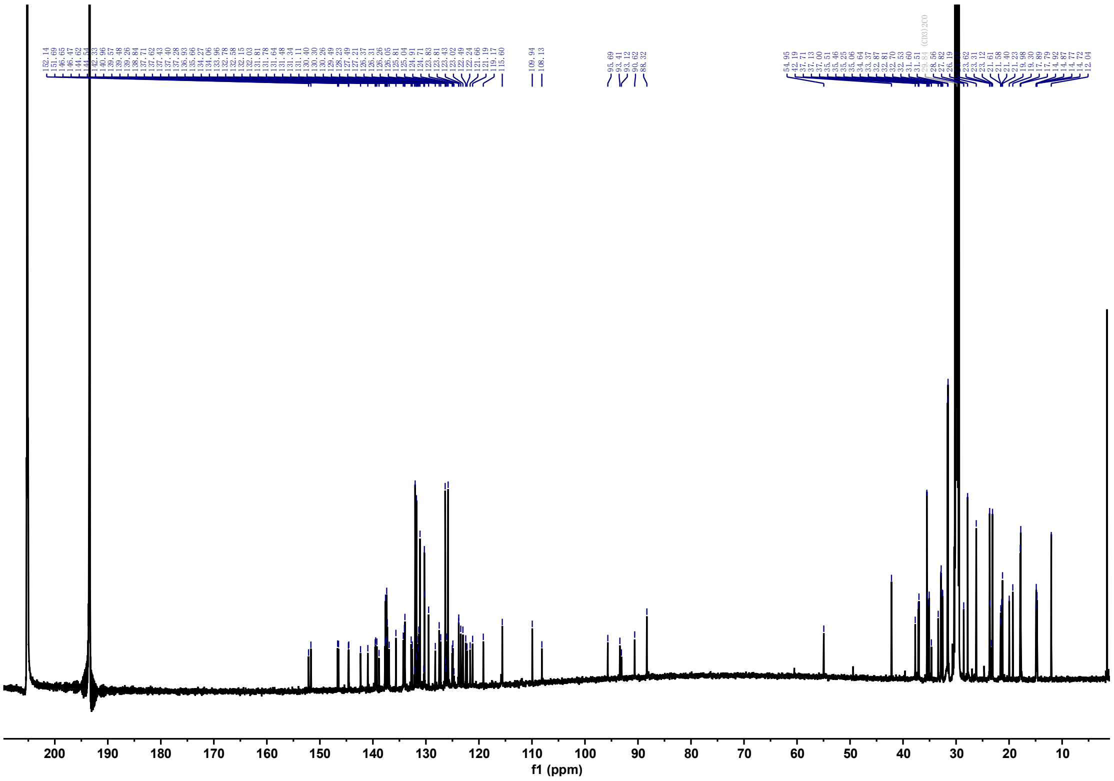

^{1}H NMR spectrum (600 MHz, Acetone-*d6*/CS_2, 298 K) of compound **34**

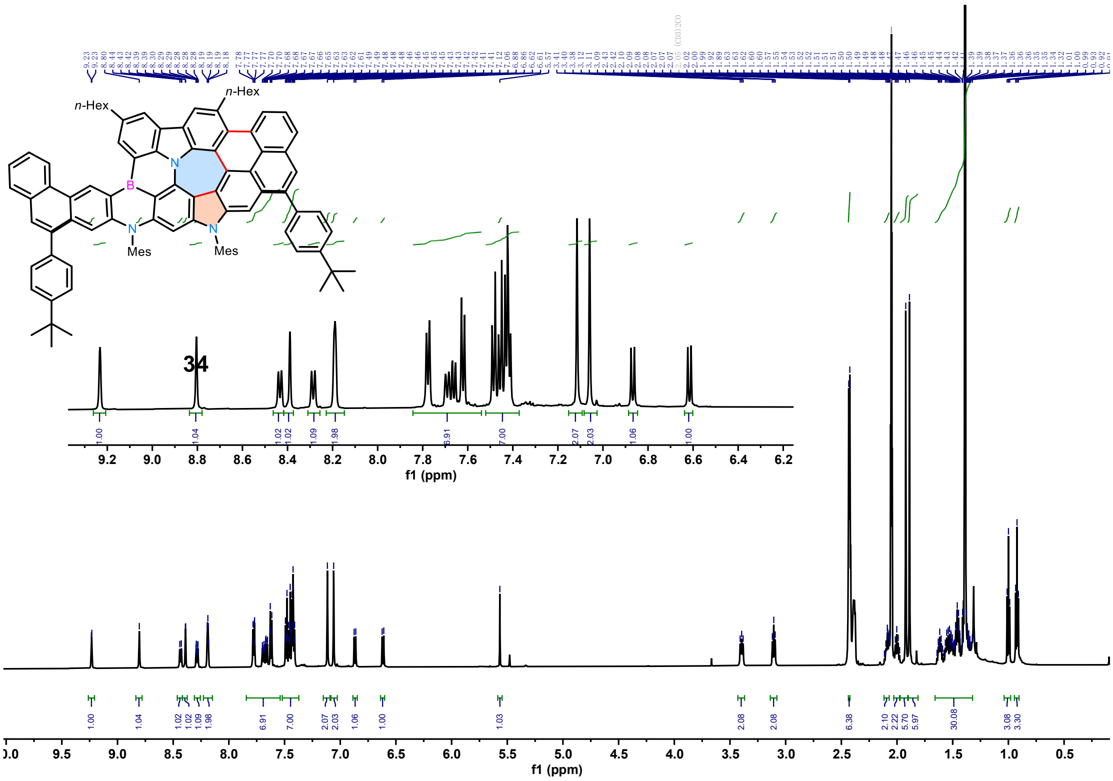

^{13}C NMR spectrum (151 MHz, Acetone-*d6*/CS_2, 298 K) of compound **34**

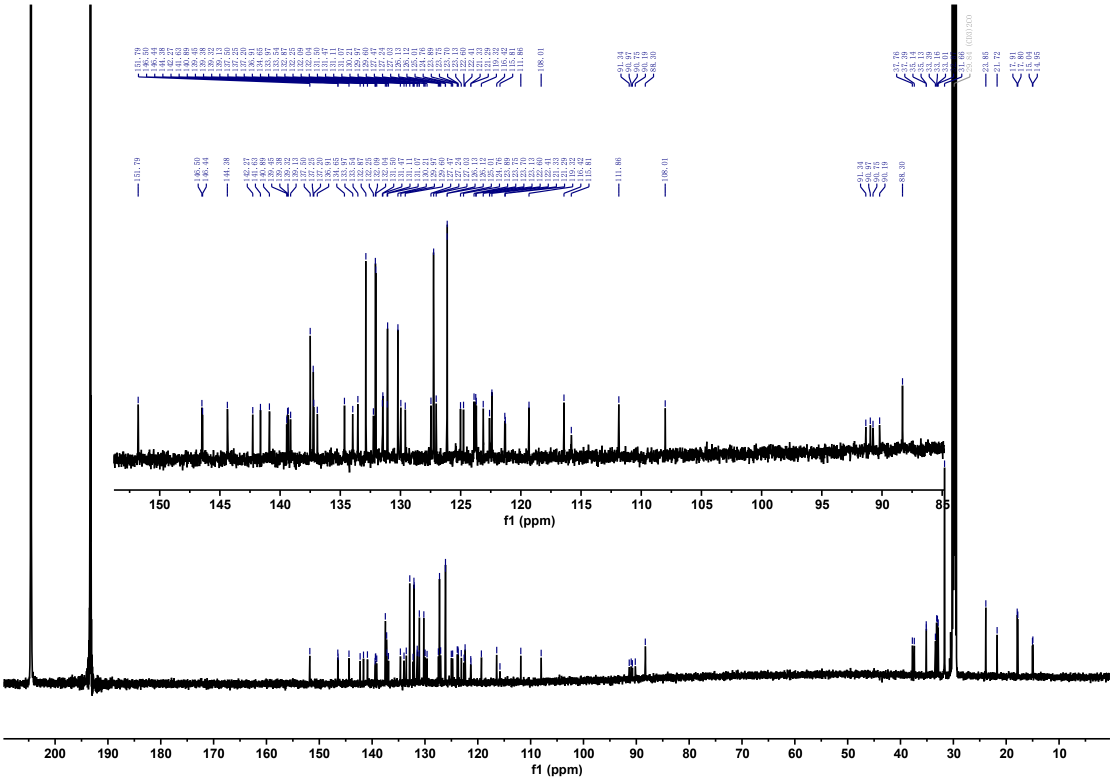

^{1}H NMR spectrum (600 MHz, Acetone-*d6*/CS_2, 298 K) of compound **35**

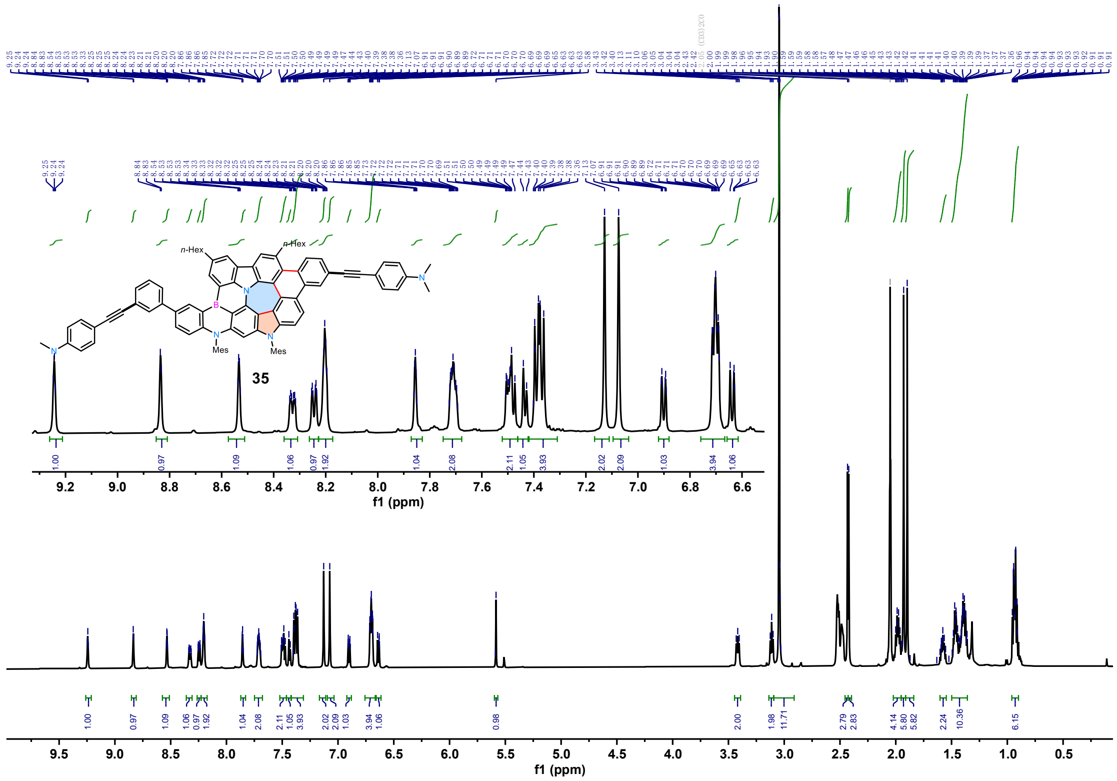

^{13}C NMR spectrum (151 MHz, Acetone-*d6*/CS_2, 298 K) of compound **35**

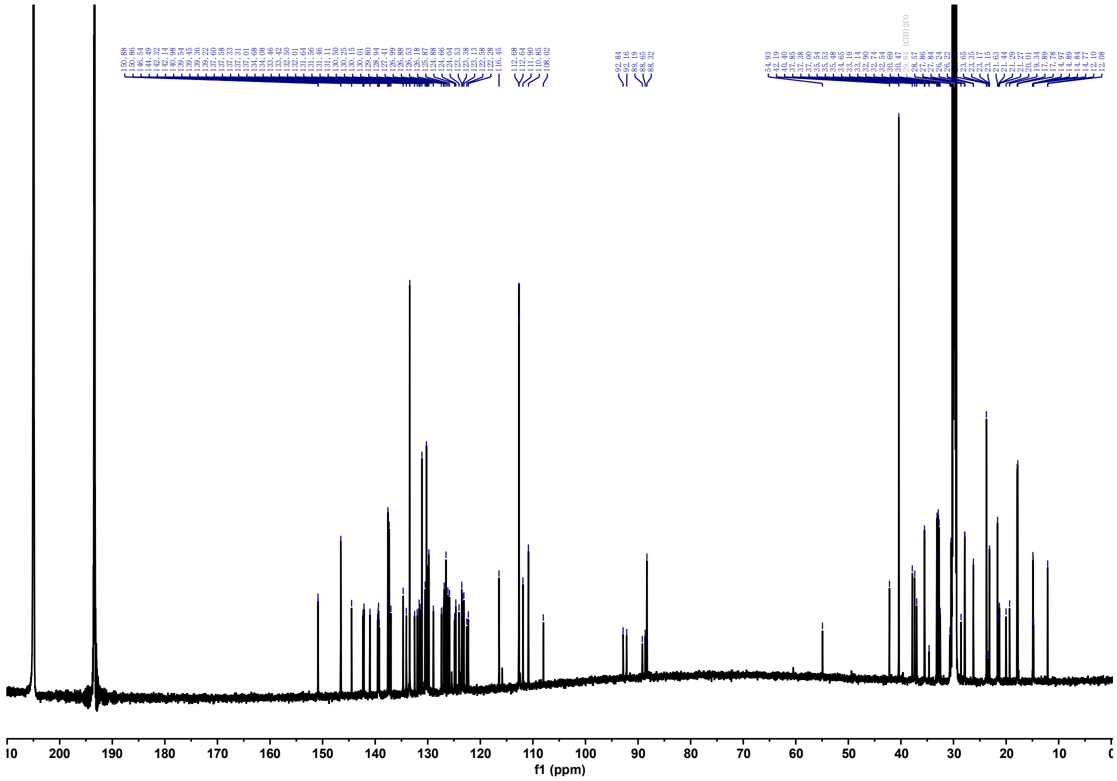

^{1}H NMR spectrum (400 MHz, $CDCl_3$, 298 K) of compound **36**

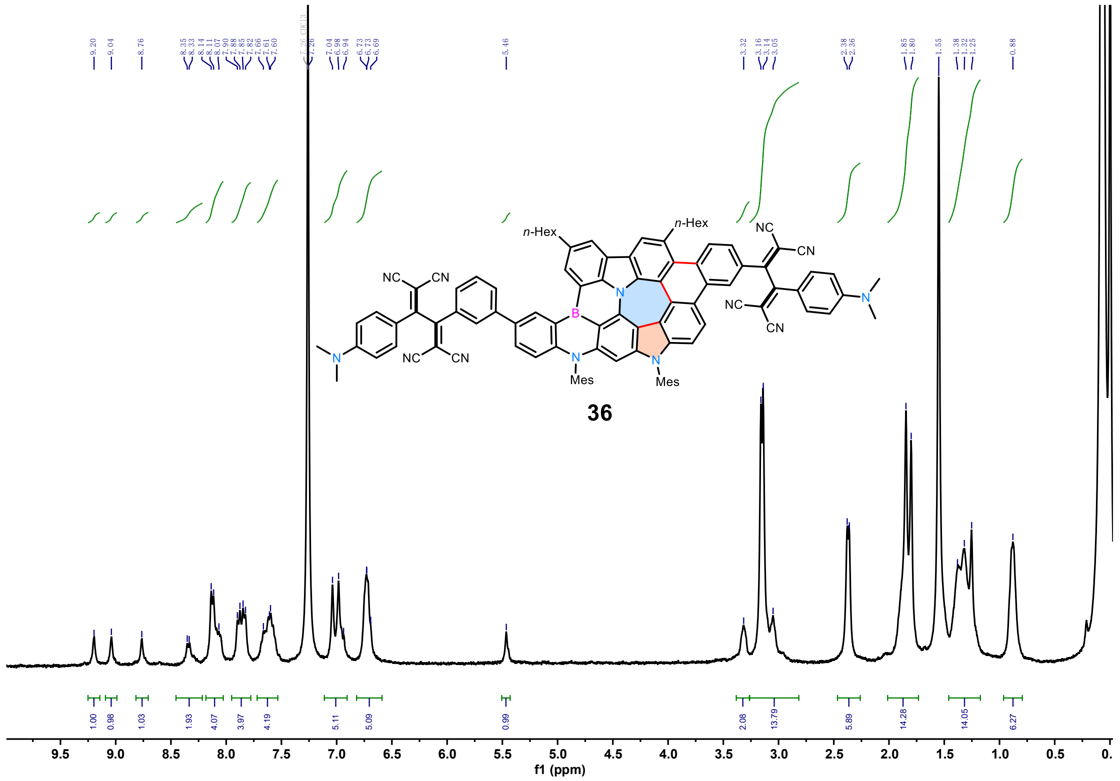

^{13}C NMR spectrum (151 MHz, Acetone-*d6*/CS_2, 298 K) of compound **36**

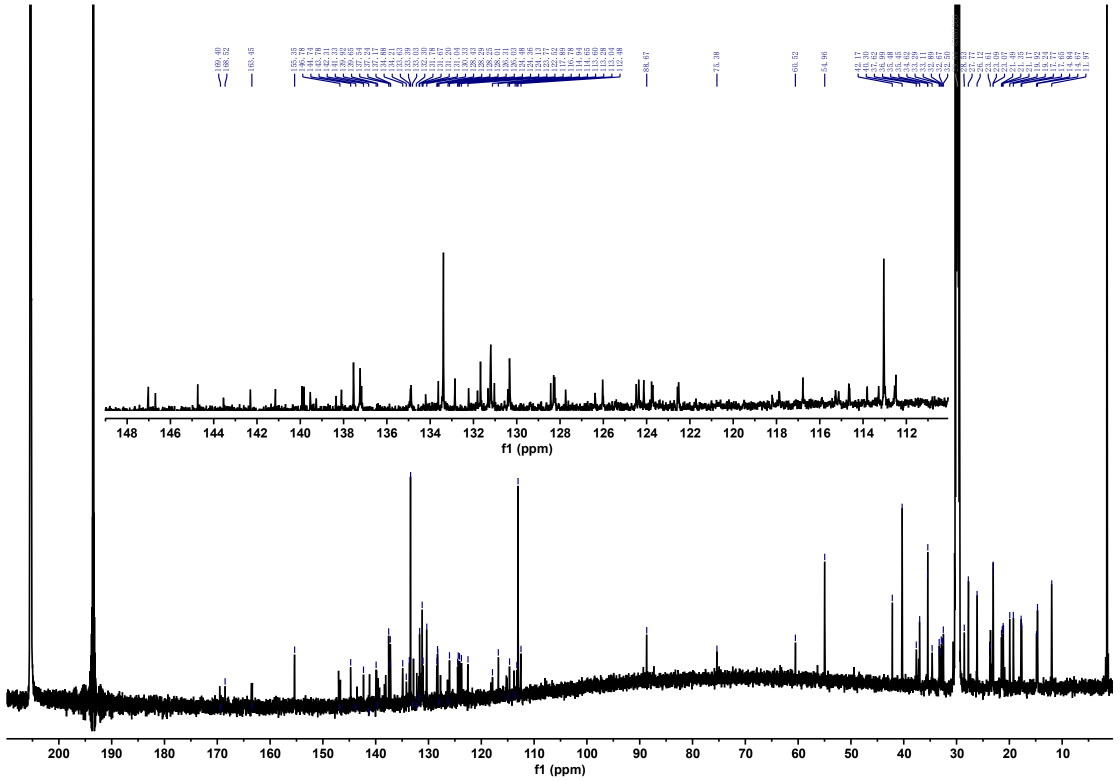

^{19}F NMR spectrum (471 MHz, $CDCl_3$, 298 K) of compound **3d**

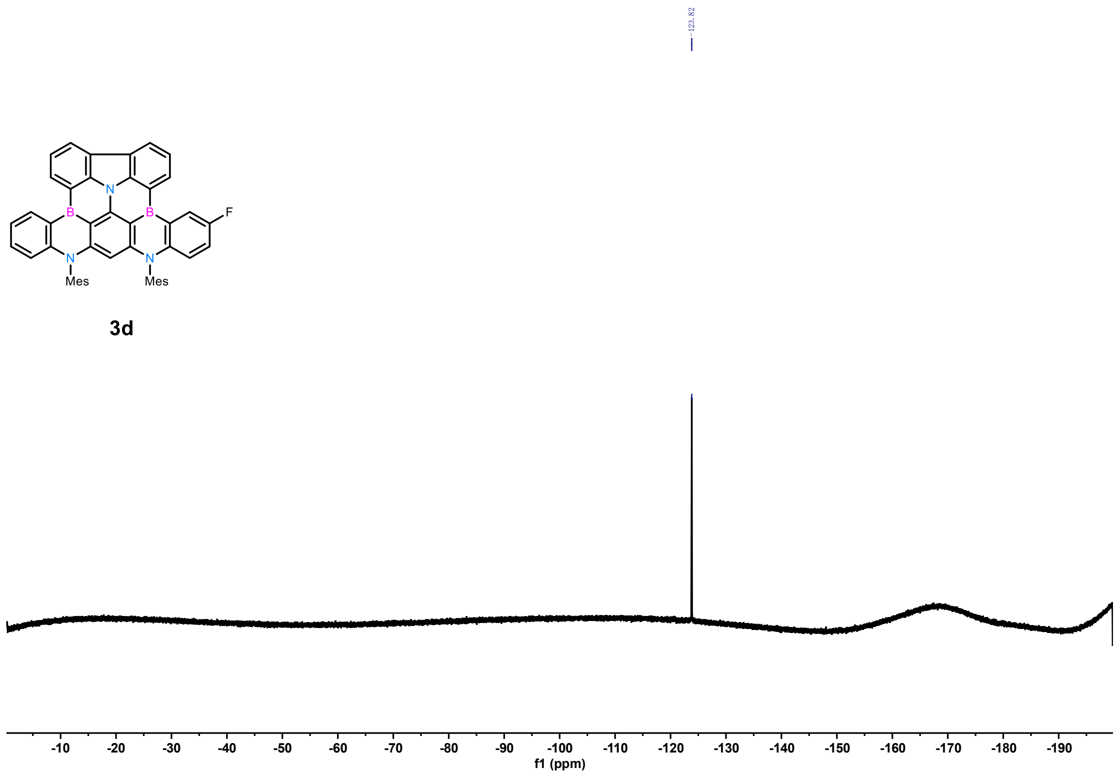

^{19}F NMR spectrum (565 MHz, Acetone-/CS_2, 298 K) of compound **5d**

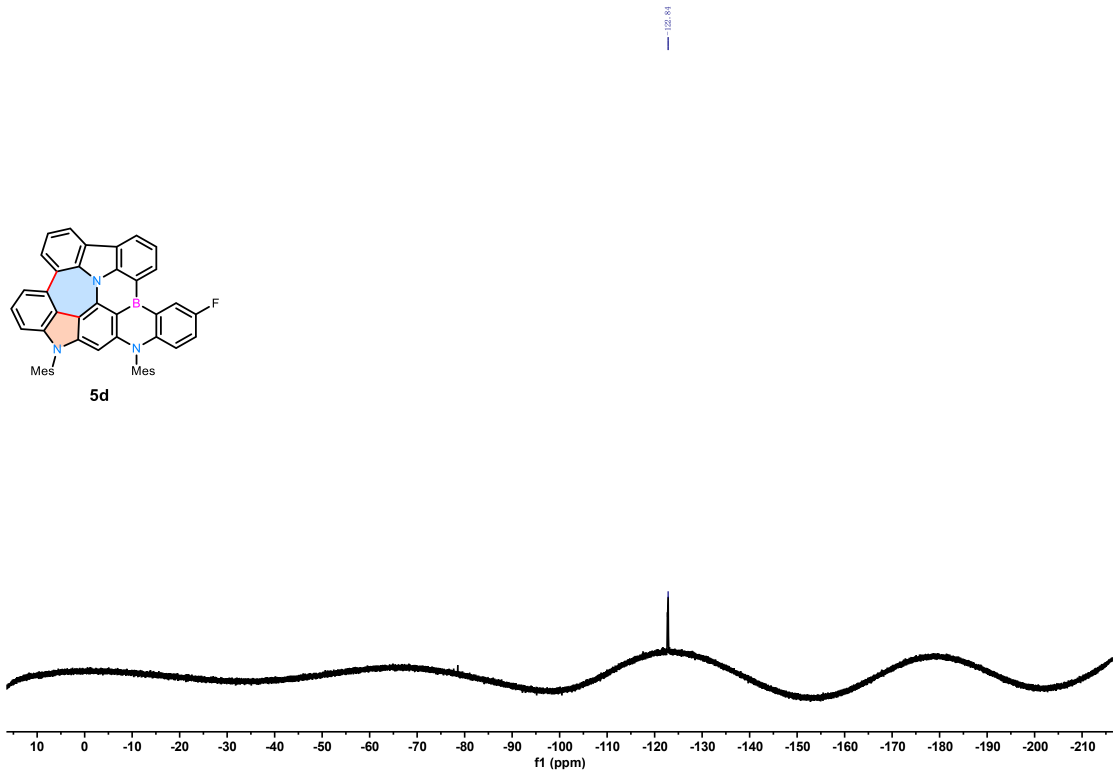

^{19}F NMR spectrum (377 MHz, CDCl$_3$, 298 K) of compound **9c**

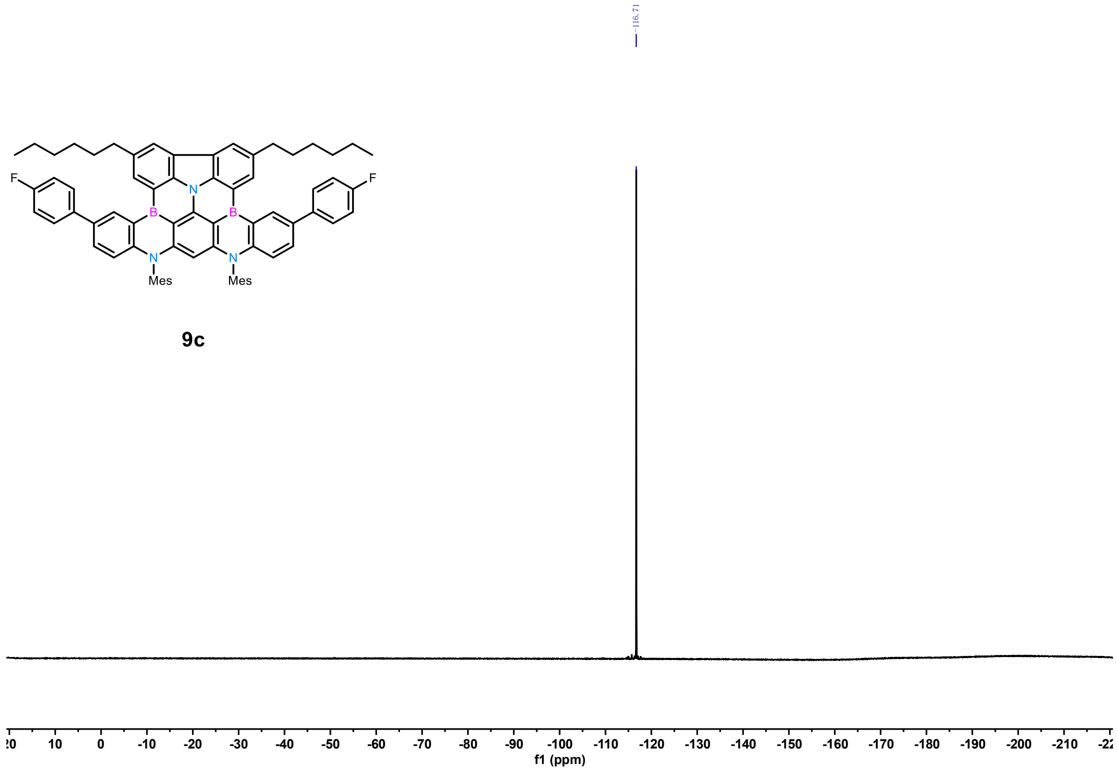

^{19}F NMR spectrum (377 MHz, CDCl$_3$, 298 K) of compound **10c**

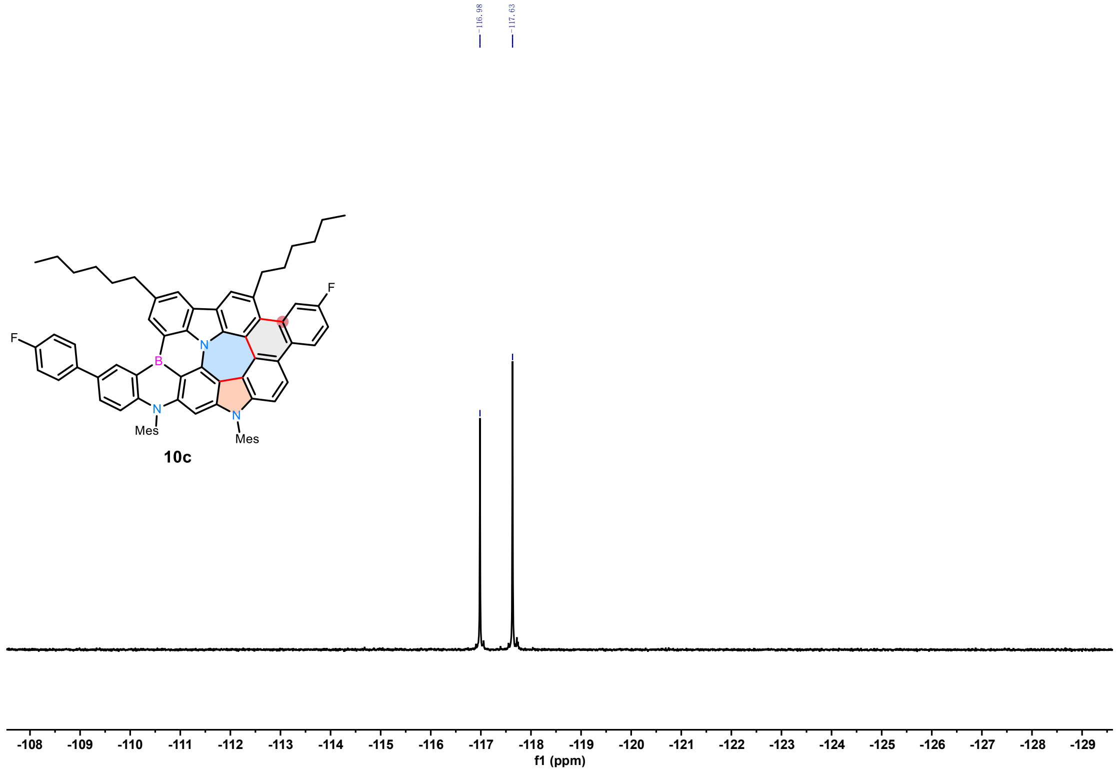

^{19}F NMR spectrum (377 MHz, $CDCl_3$, 298 K) of compound **9h**

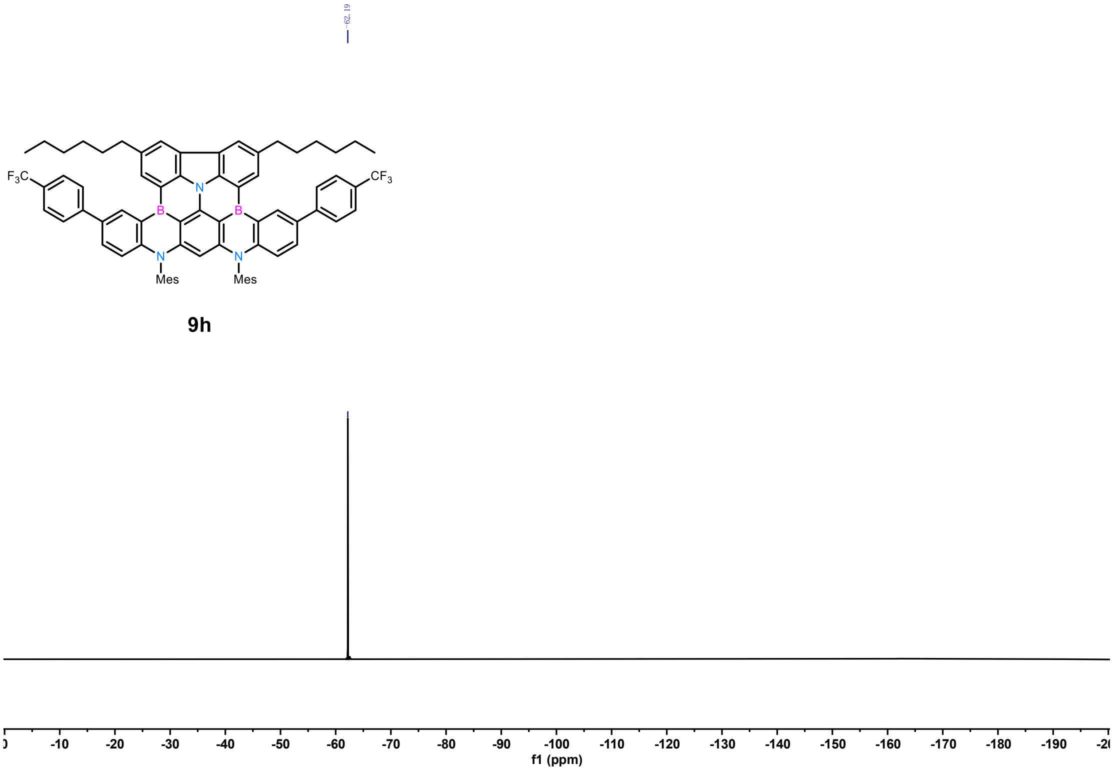

^{19}F NMR spectrum (377 MHz, $CDCl_3$, 298 K) of compound **10h**

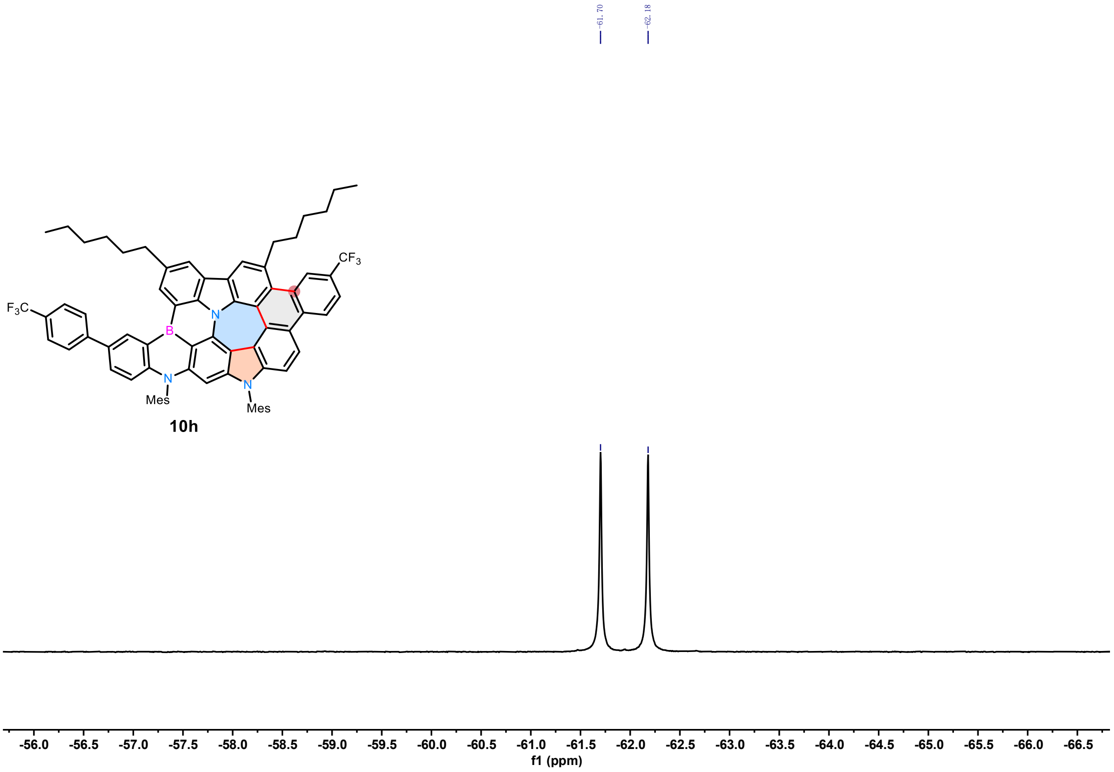

^{19}F NMR spectrum (565 MHz, $CDCl_3$, 298 K) of compound **S8**

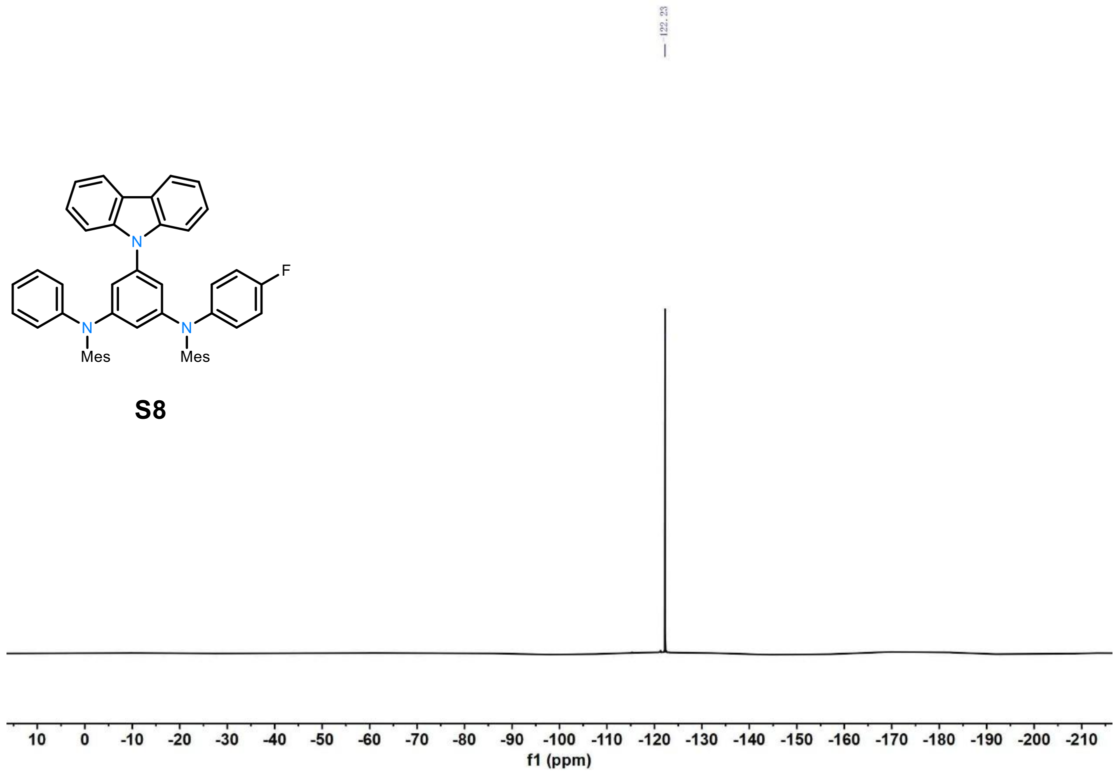

^{11}B NMR spectrum (128 MHz, $CDCl_3$, 298 K) of compound **2**

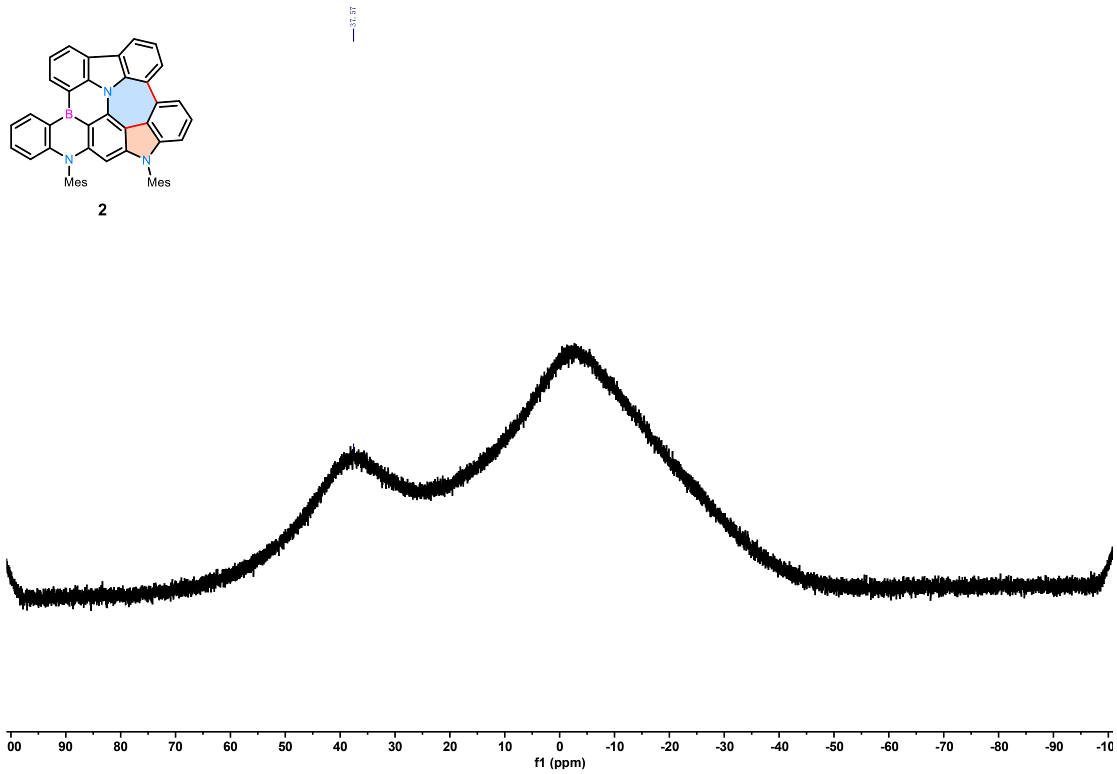

^{11}B NMR spectrum (128 MHz, $CDCl_3$, 298 K) of compound **4b’**

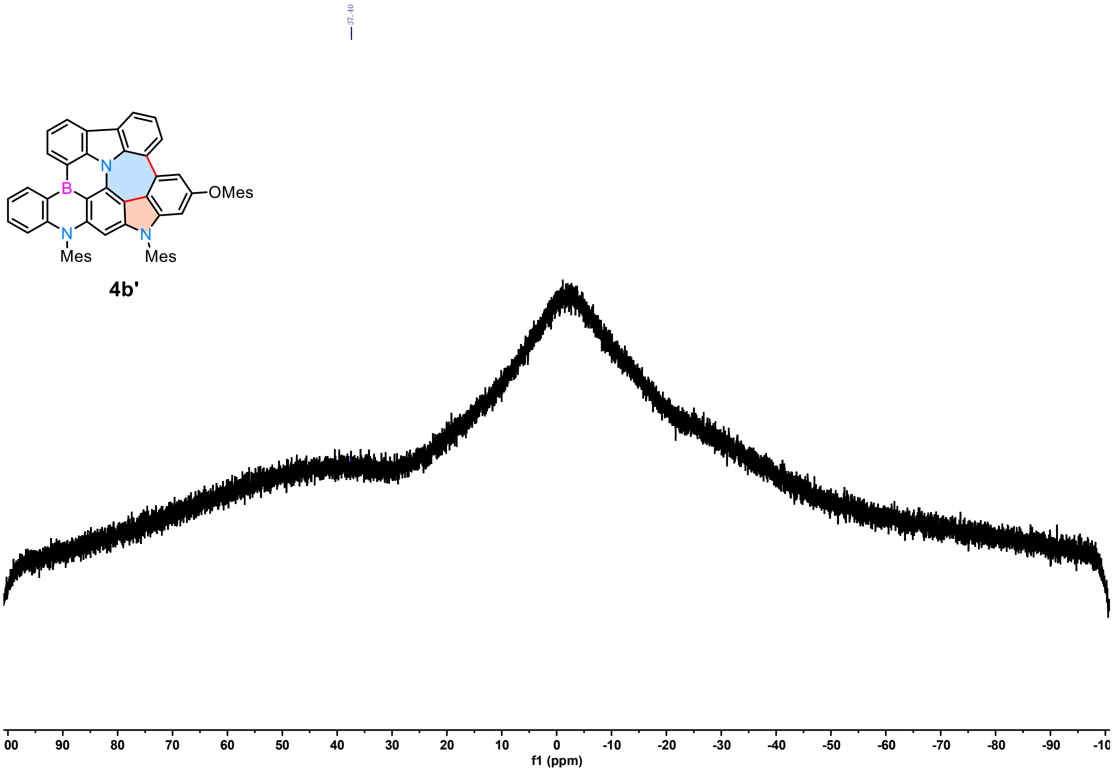

^{11}B NMR spectrum (128 MHz, $CDCl_3/CS_2$, 298 K) of compound **5b**

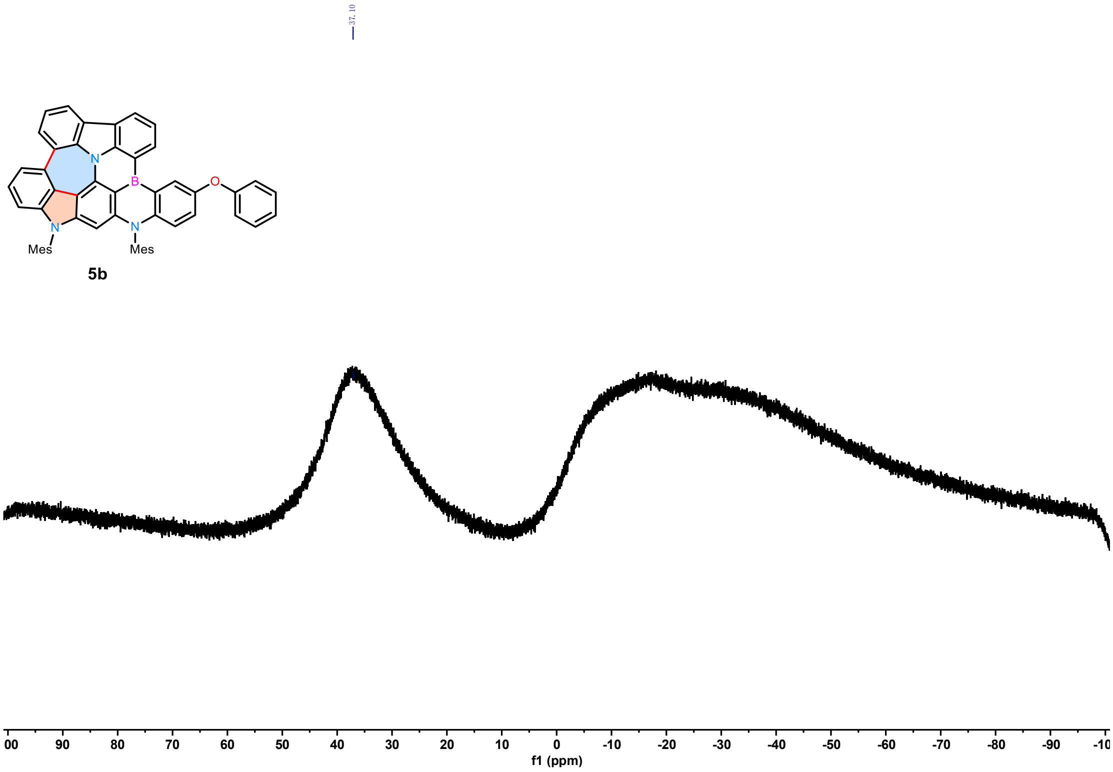

^{11}B NMR spectrum (128 MHz, $CDCl_3/CS_2$, 298 K) of compound **5c**

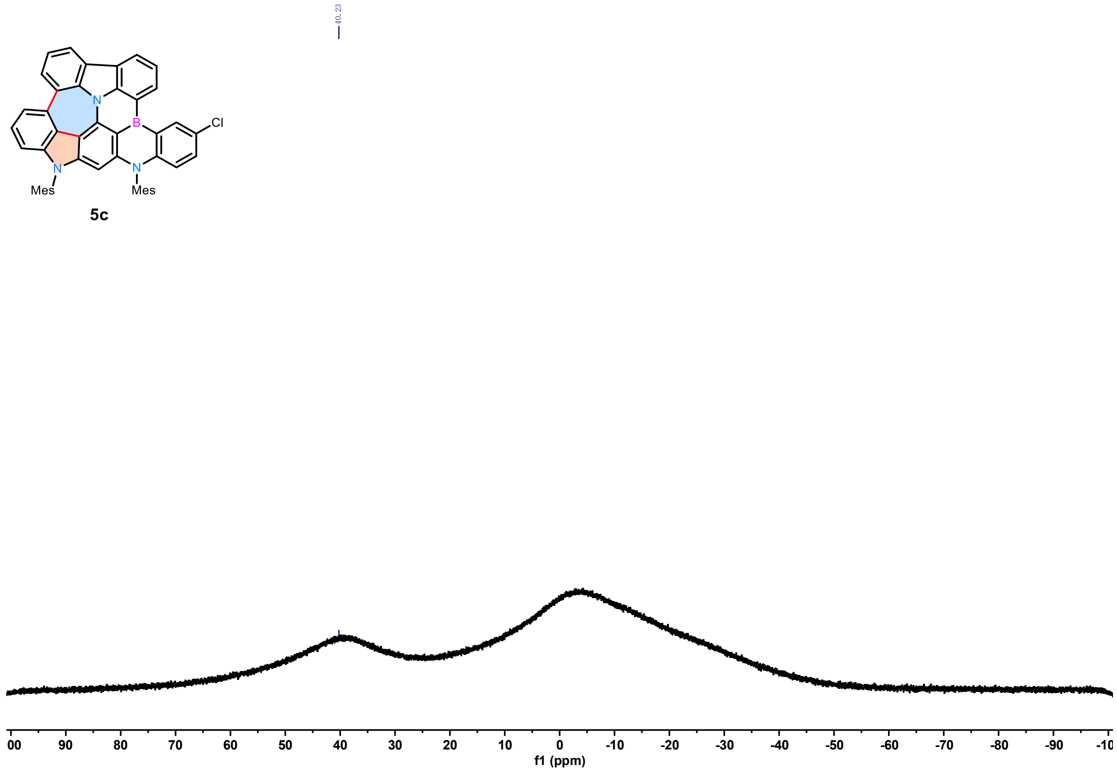

^{11}B NMR spectrum (128 MHz, $CDCl_3$, 298 K) of compound **5d**

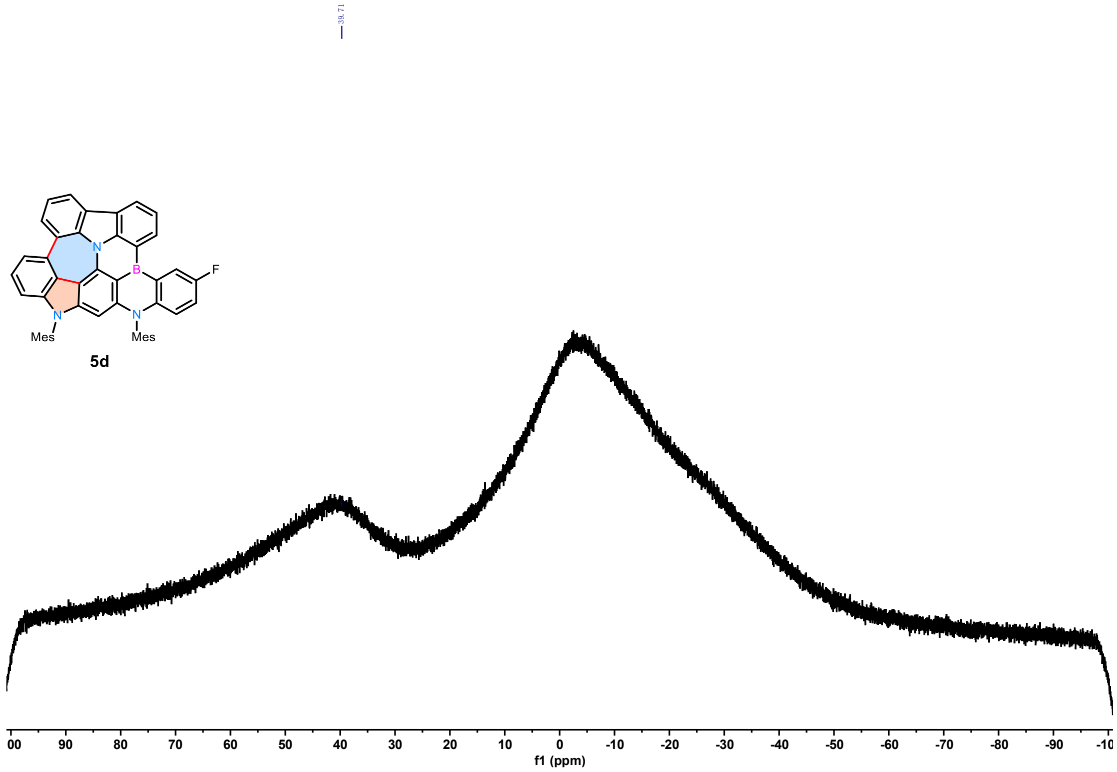

^{11}B NMR spectrum (128 MHz, $CDCl_3$, 298 K) of compound **8a**

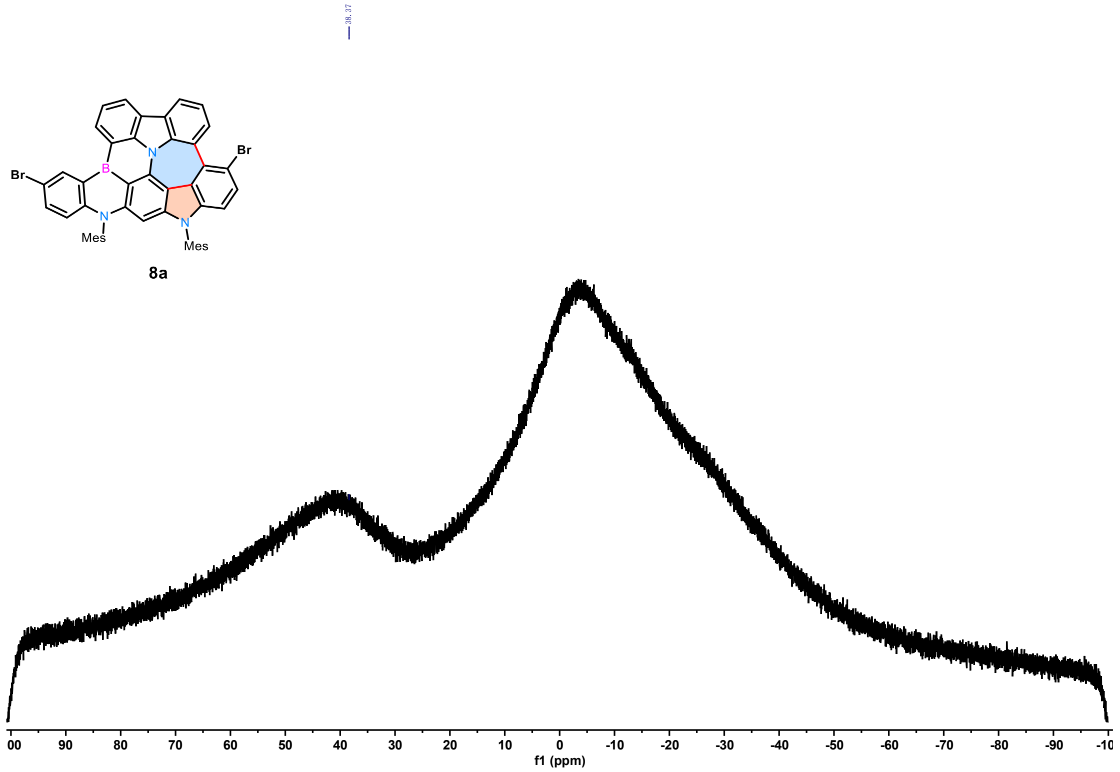

^{11}B NMR spectrum (128 MHz, $CDCl_3/CS_2$, 298 K) of compound **8b**

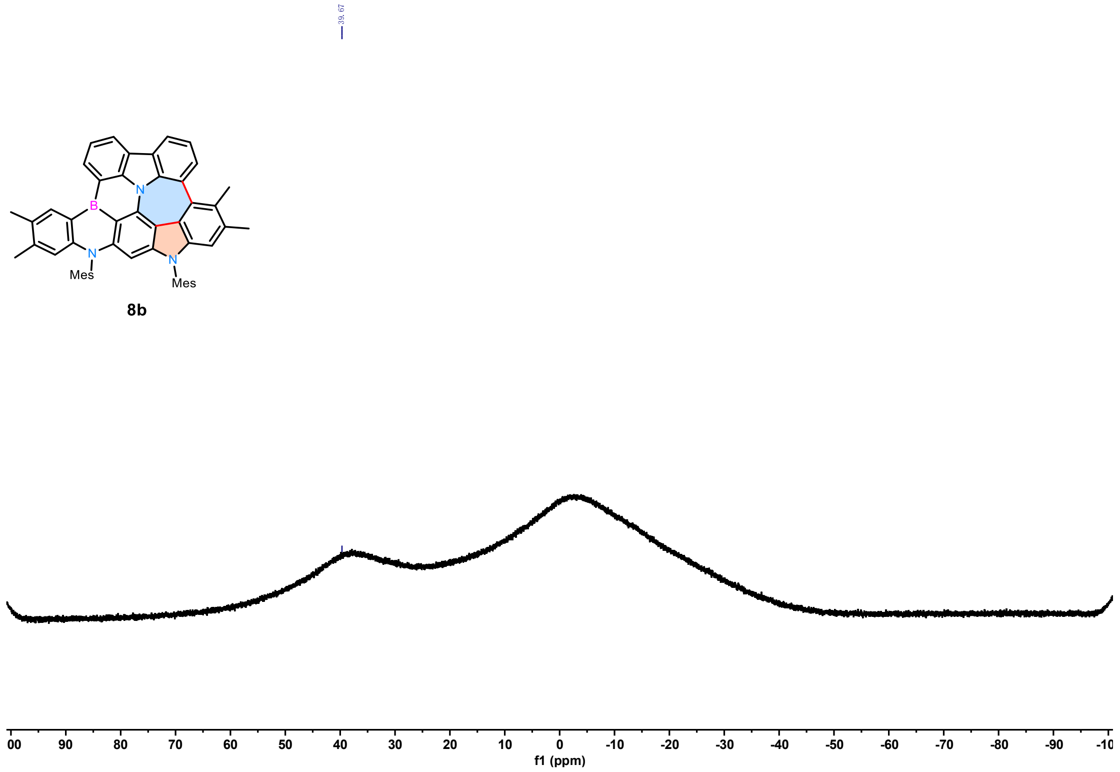

^{11}B NMR spectrum (128 MHz, $CDCl_3/CS_2$, 298 K) of compound **8c**

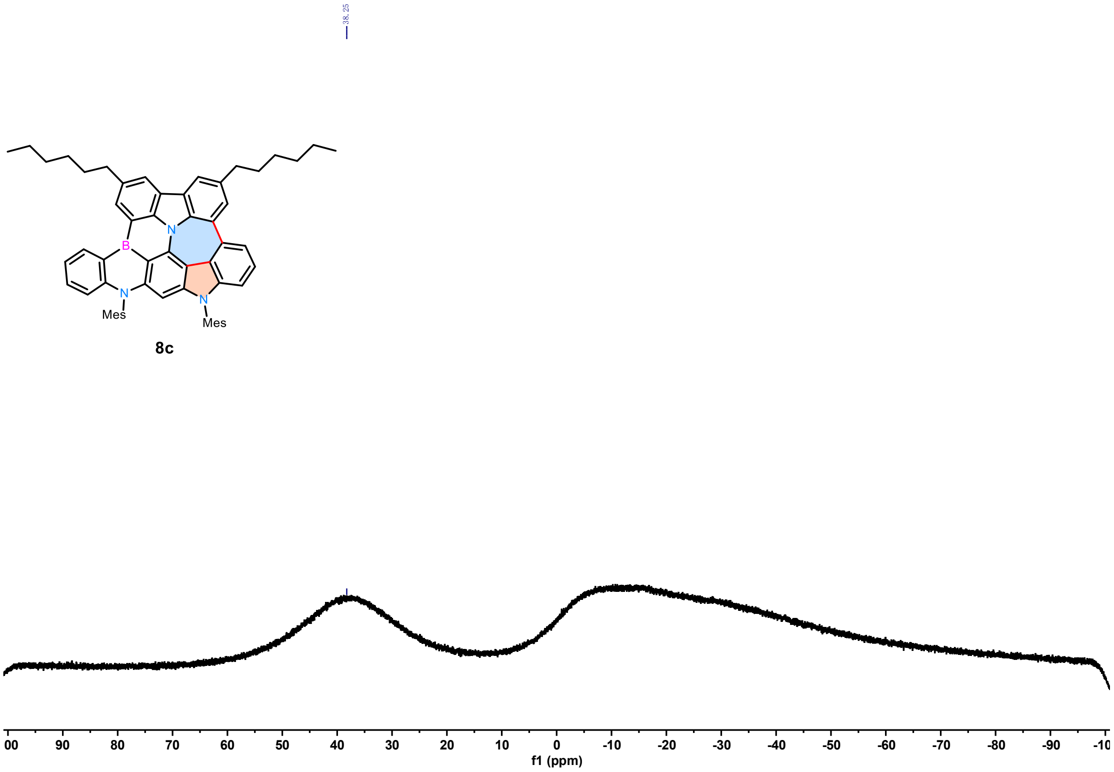

8c

^{11}B NMR spectrum (128 MHz, $CDCl_3/CS_2$, 298 K) of compound **8d**

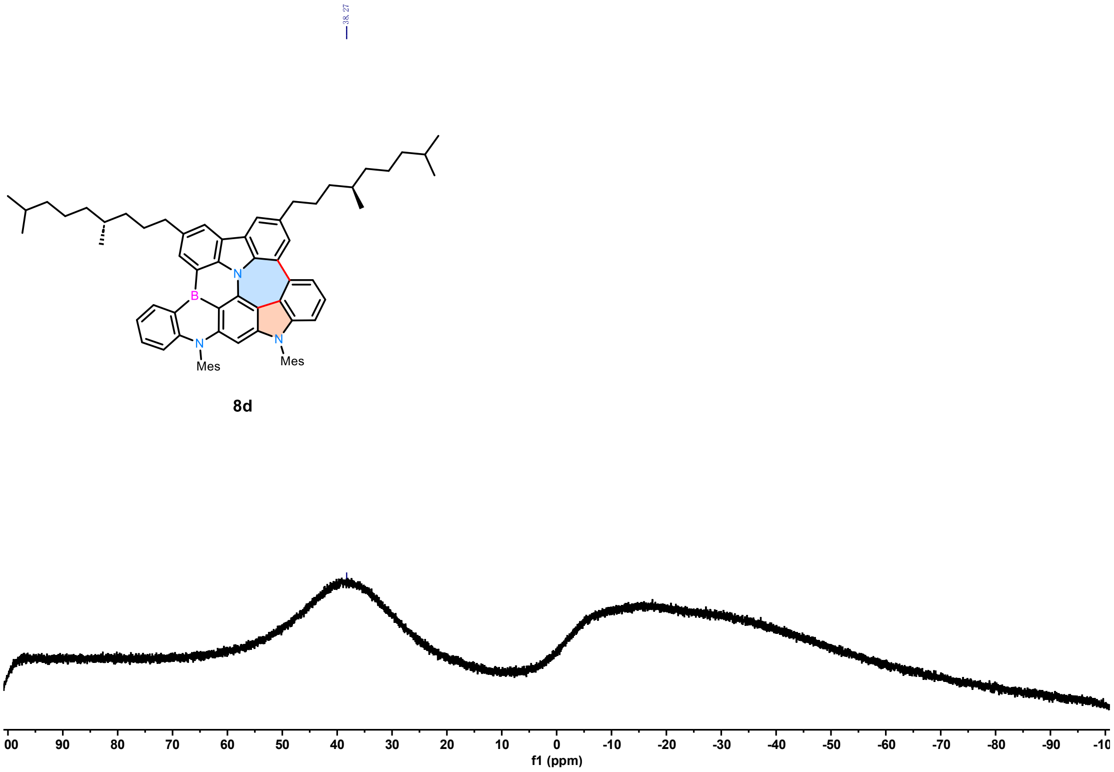

8d

^{11}B NMR spectrum (128 MHz, $CDCl_3/CS_2$, 298 K) of compound **8e**

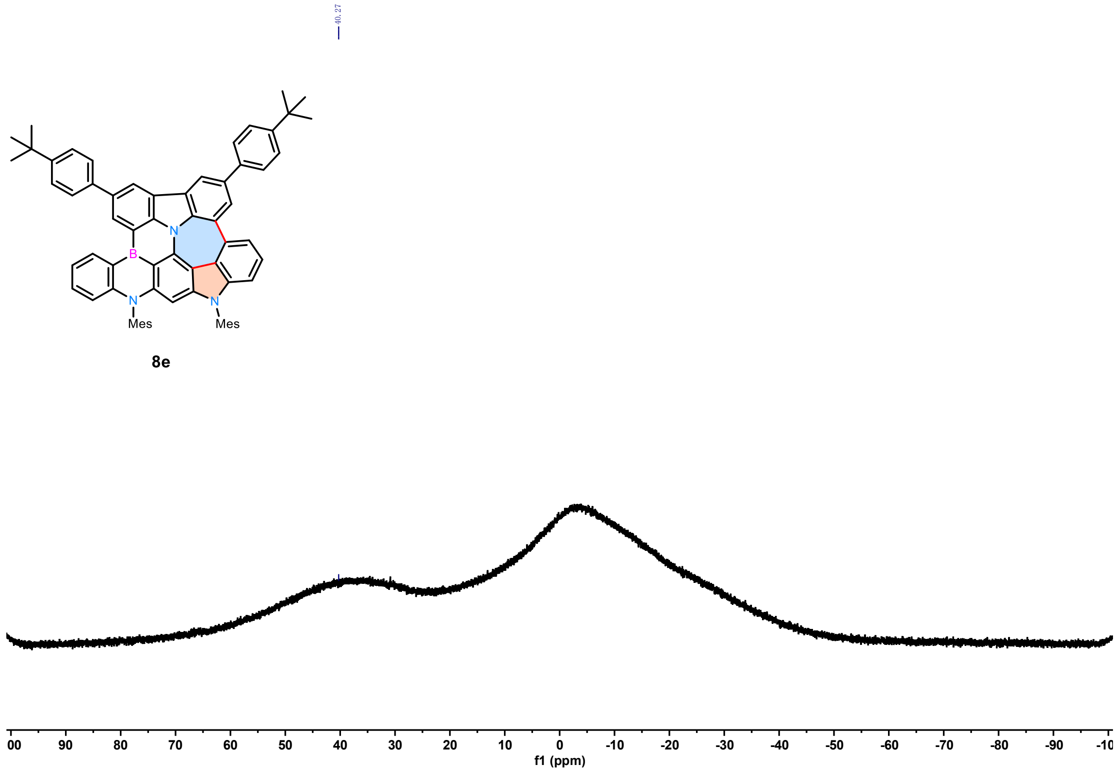

^{11}B NMR spectrum (128 MHz, $CDCl_3/CS_2$, 298 K) of compound **8f**

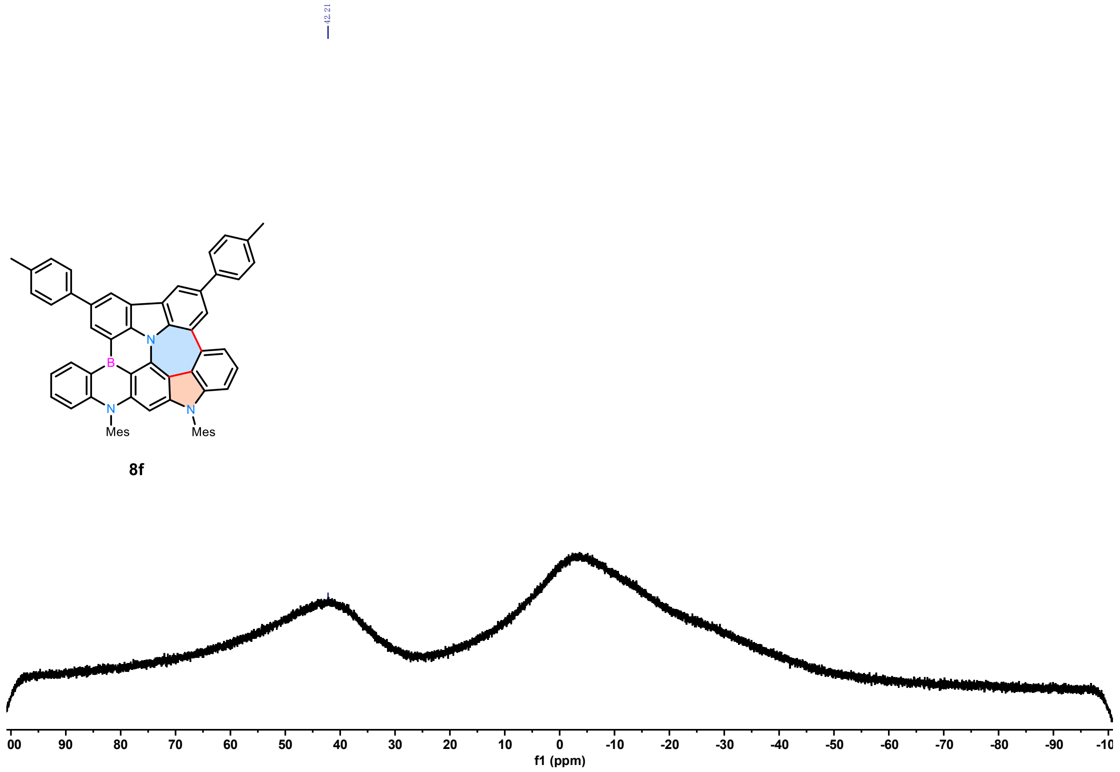

^{11}B NMR spectrum (128 MHz, $CDCl_3$, 298 K) of compound **8g**

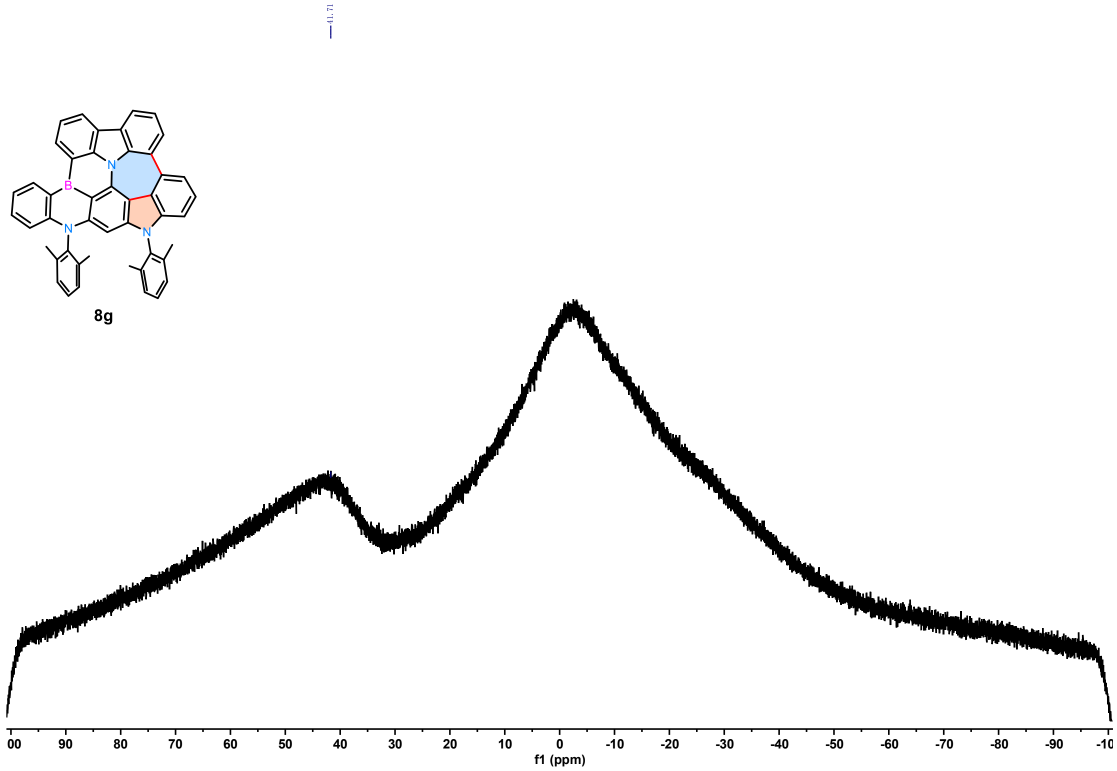

^{11}B NMR spectrum (128 MHz, $CDCl_3/CS_2$, 298 K) of compound **8h**

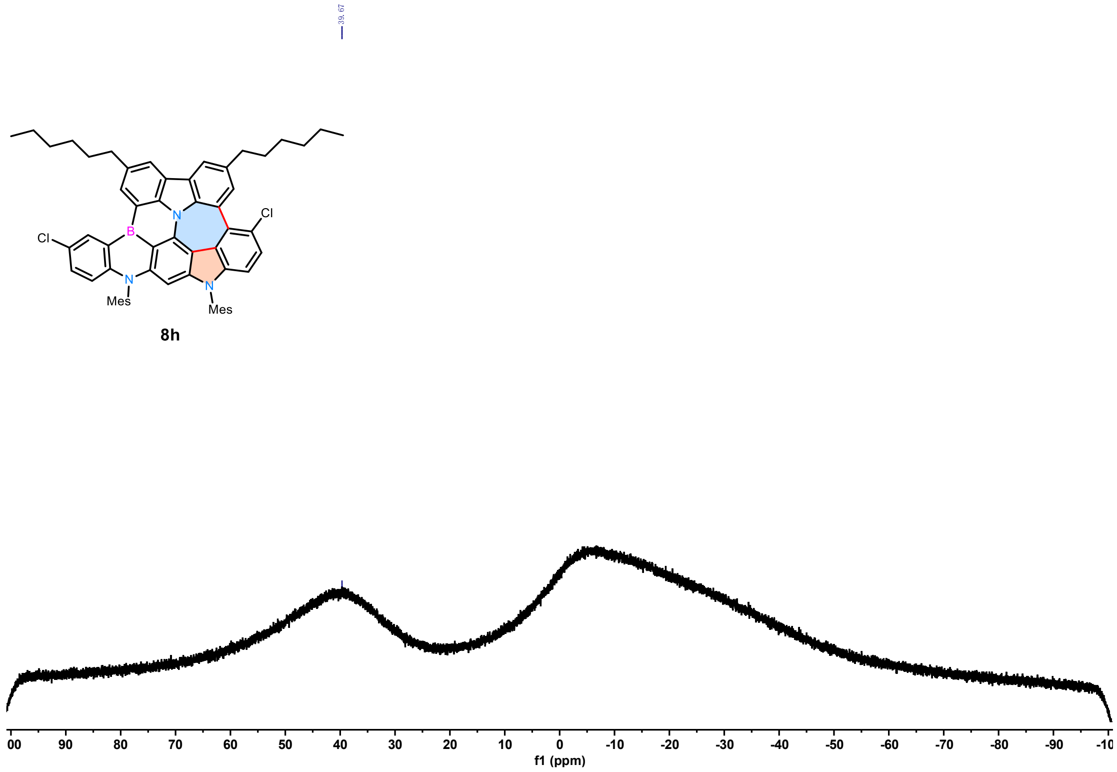

^{11}B NMR spectrum (128 MHz, $CDCl_3/CS_2$, 298 K) of compound **8i**

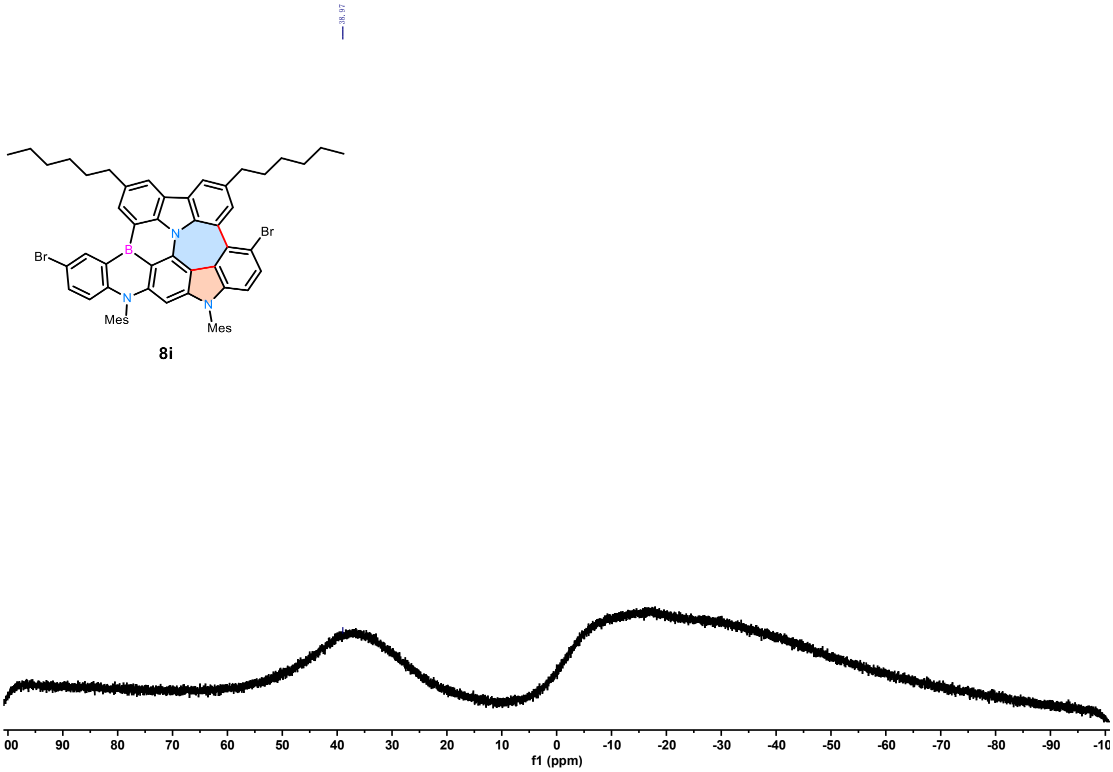

^{11}B NMR spectrum (128 MHz, $CDCl_3/CS_2$, 298 K) of compound **8j**

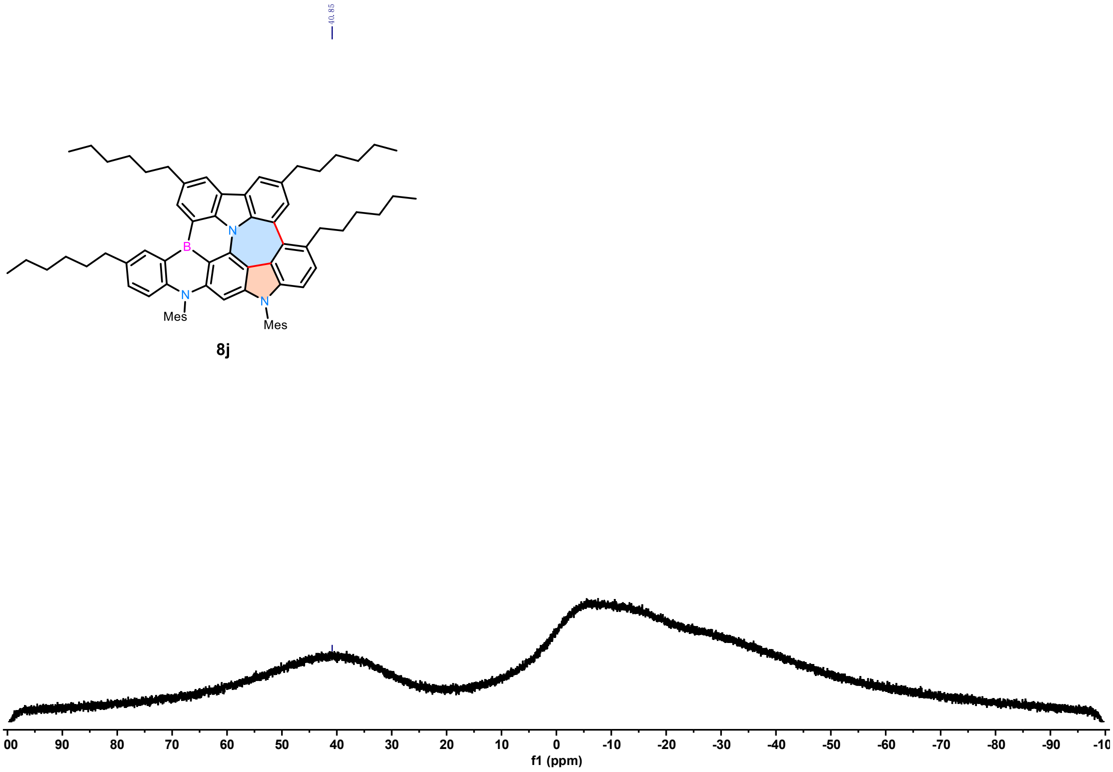

^{11}B NMR spectrum (128 MHz, $CDCl_3/CS_2$, 298 K) of compound **8k**

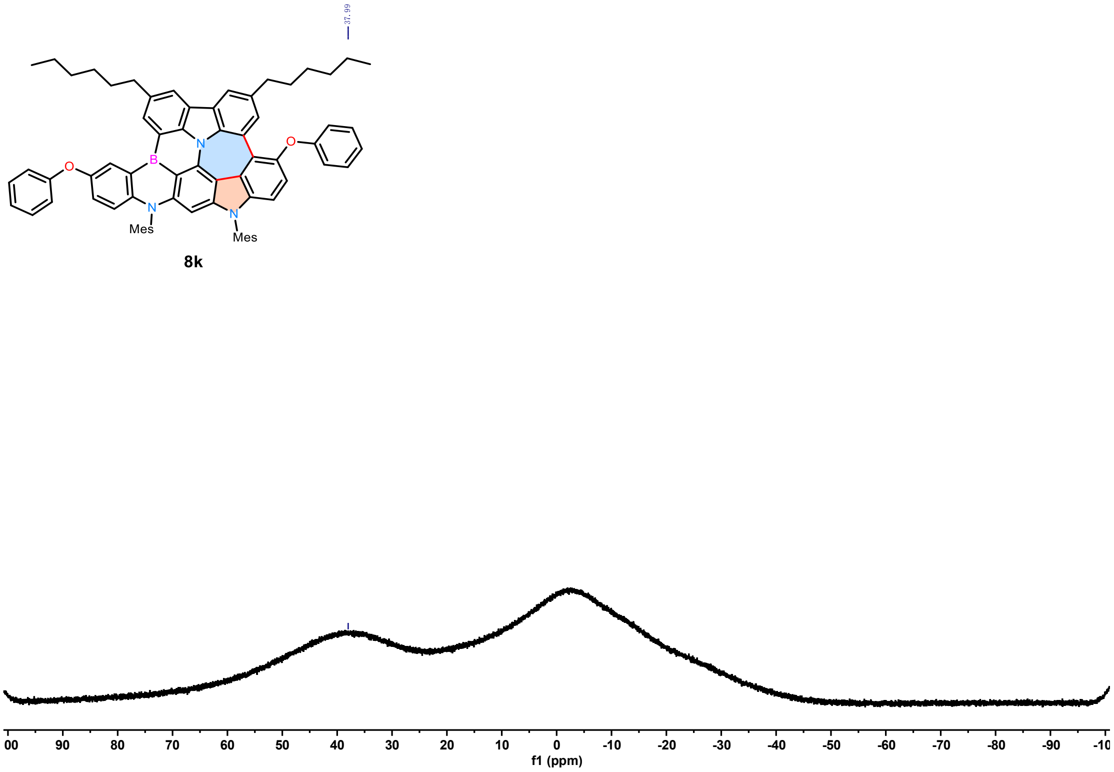

^{11}B NMR spectrum (128 MHz, $CDCl_3/CS_2$, 298 K) of compound **8l**

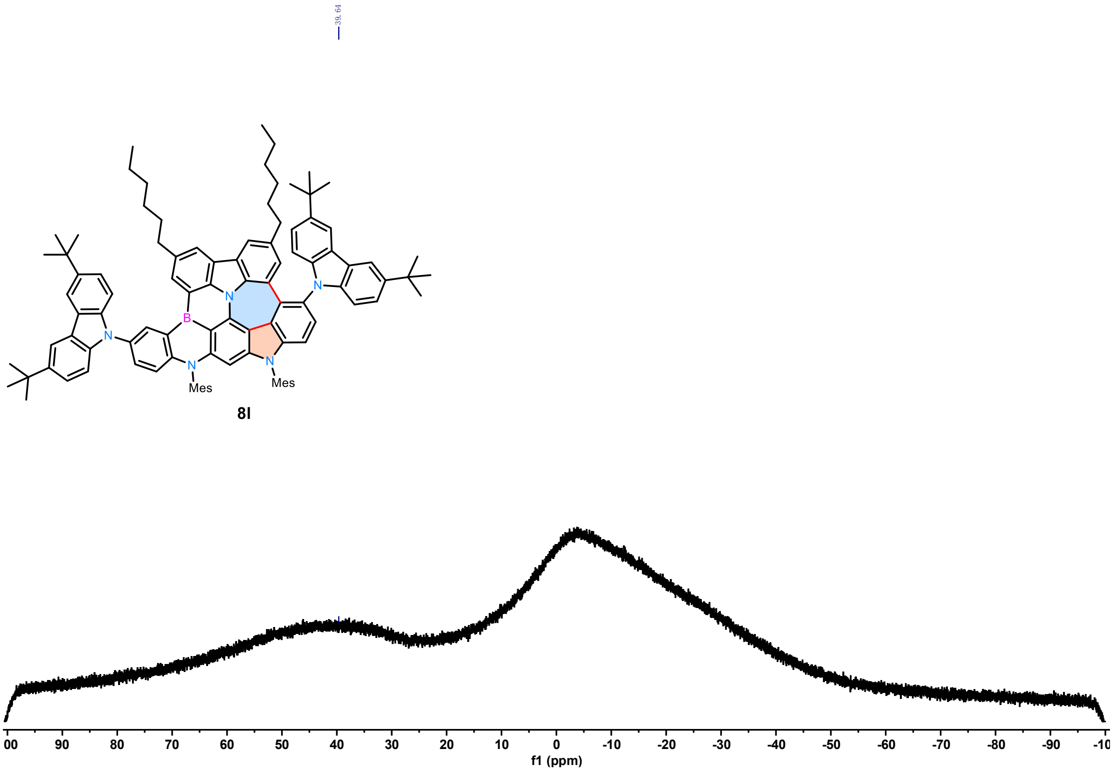

^{11}B NMR spectrum (128 MHz, $CDCl_3/CS_2$, 298 K) of compound **8m**

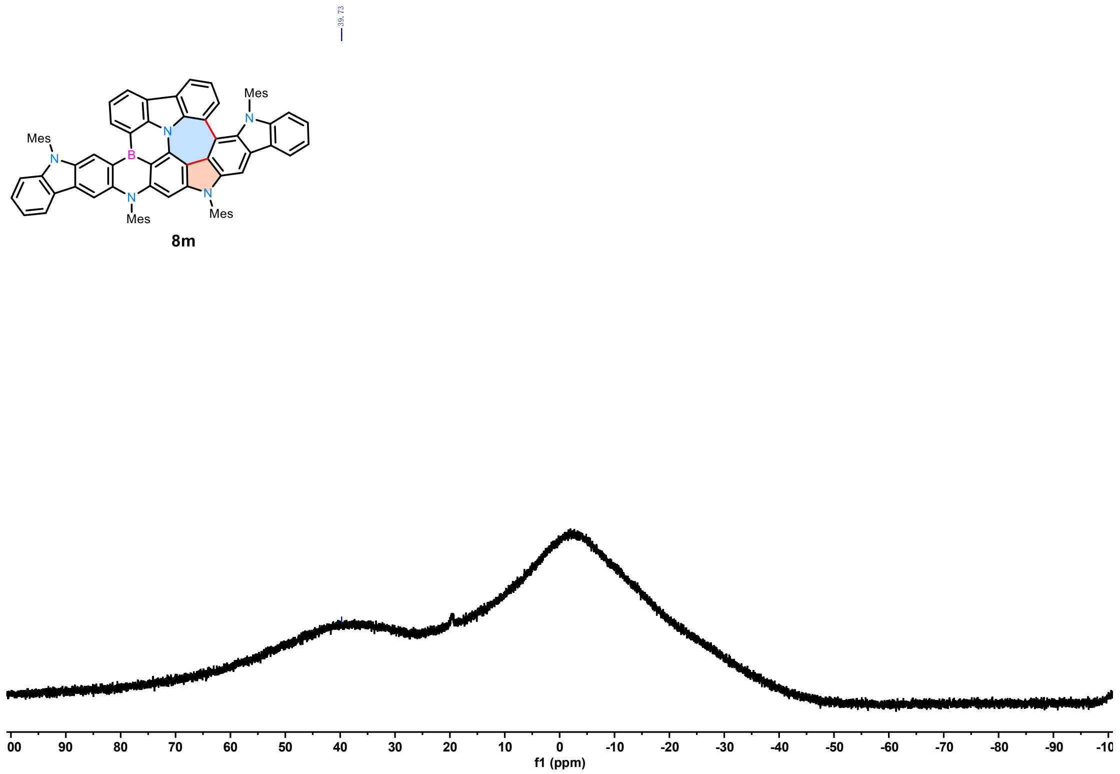

^{11}B NMR spectrum (128 MHz, $CDCl_3/CS_2$, 298 K) of compound **8n**

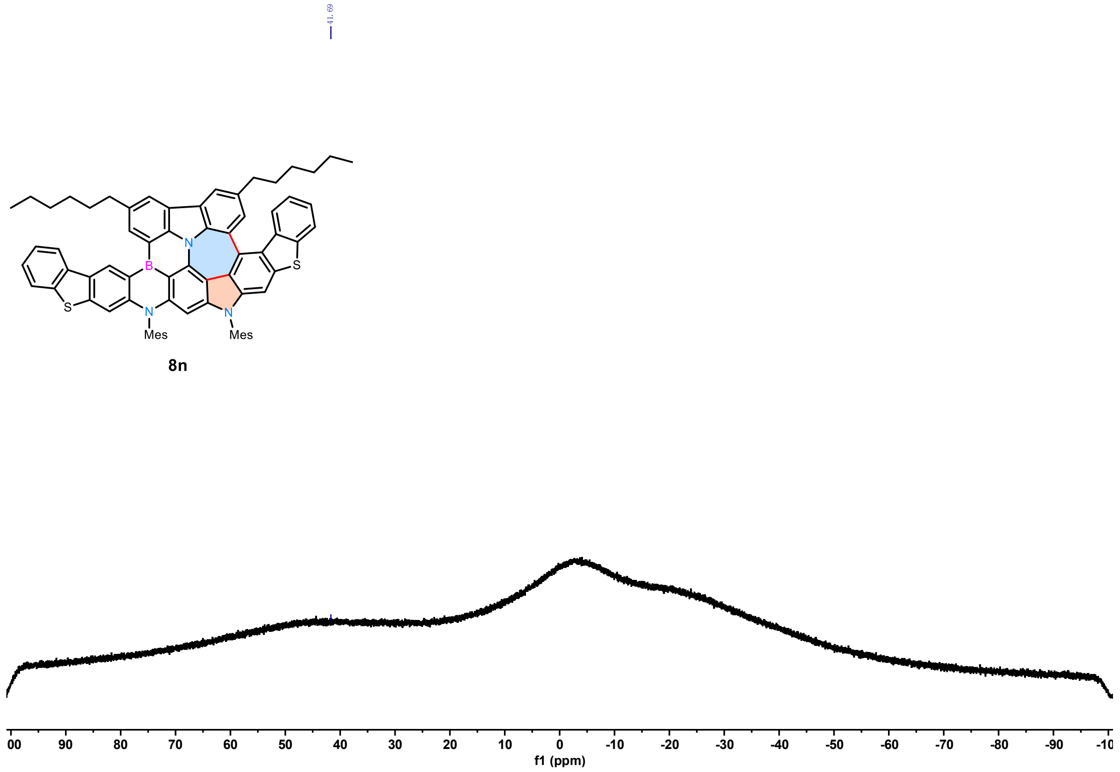

^{11}B NMR spectrum (128 MHz, $CDCl_3/CS_2$, 298 K) of compound **8o**

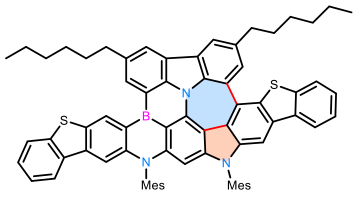

8o

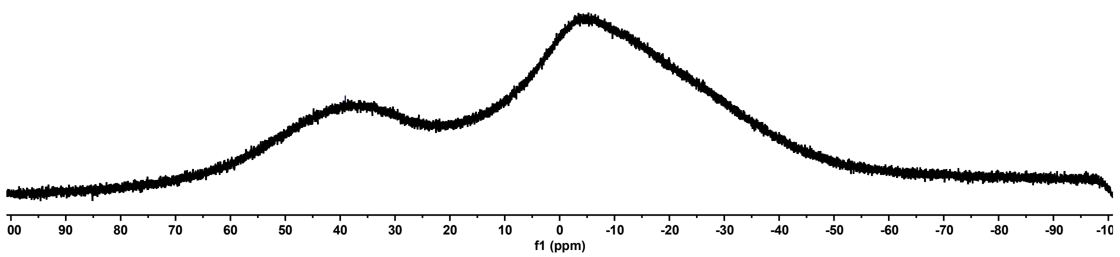

^{11}B NMR spectrum (128 MHz, $CDCl_3$, 298 K) of compound **8p**

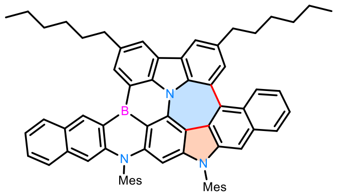

8p

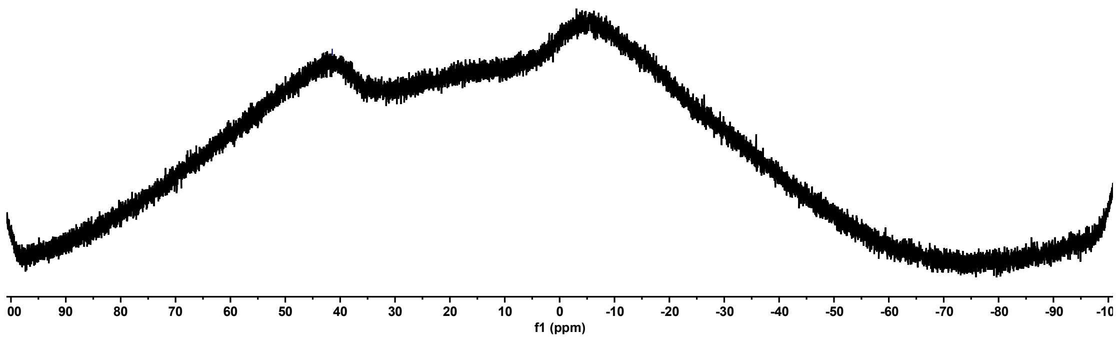

^{11}B NMR spectrum (128 MHz, $CDCl_3/CS_2$, 298 K) of compound **10a**

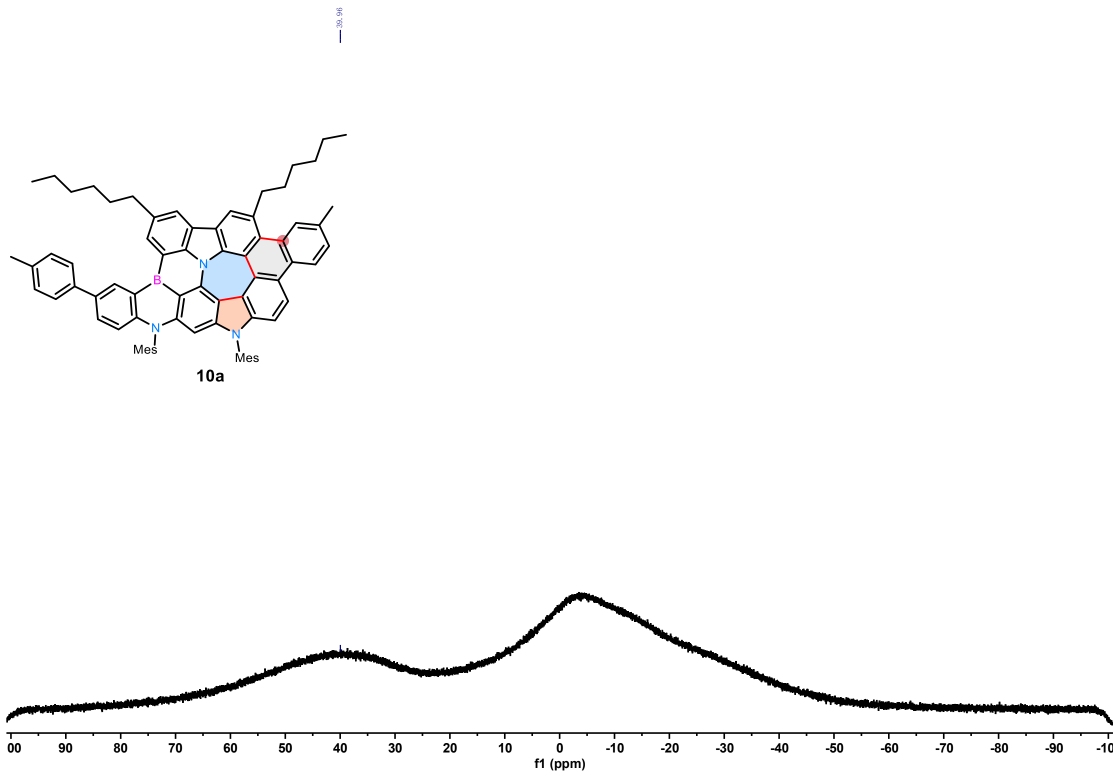

^{11}B NMR spectrum (128 MHz, $CDCl_3/CS_2$, 298 K) of compound **10b**

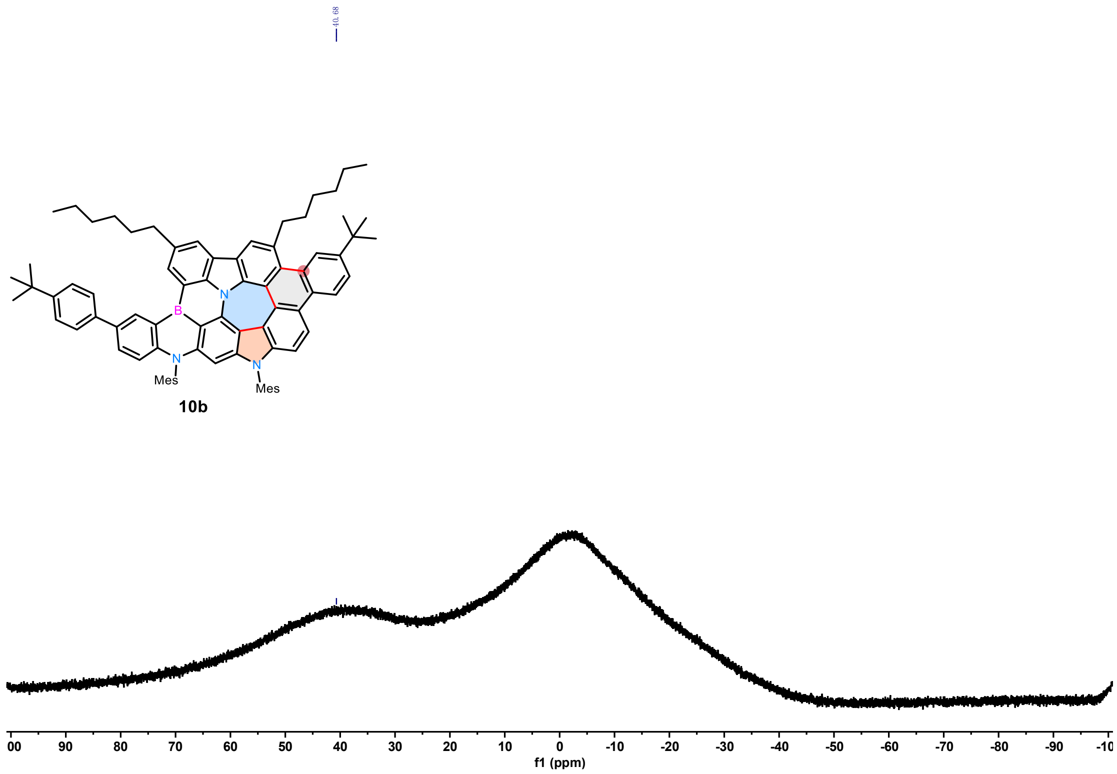

^{11}B NMR spectrum (128 MHz, $CDCl_3/CS_2$, 298 K) of compound **10c**

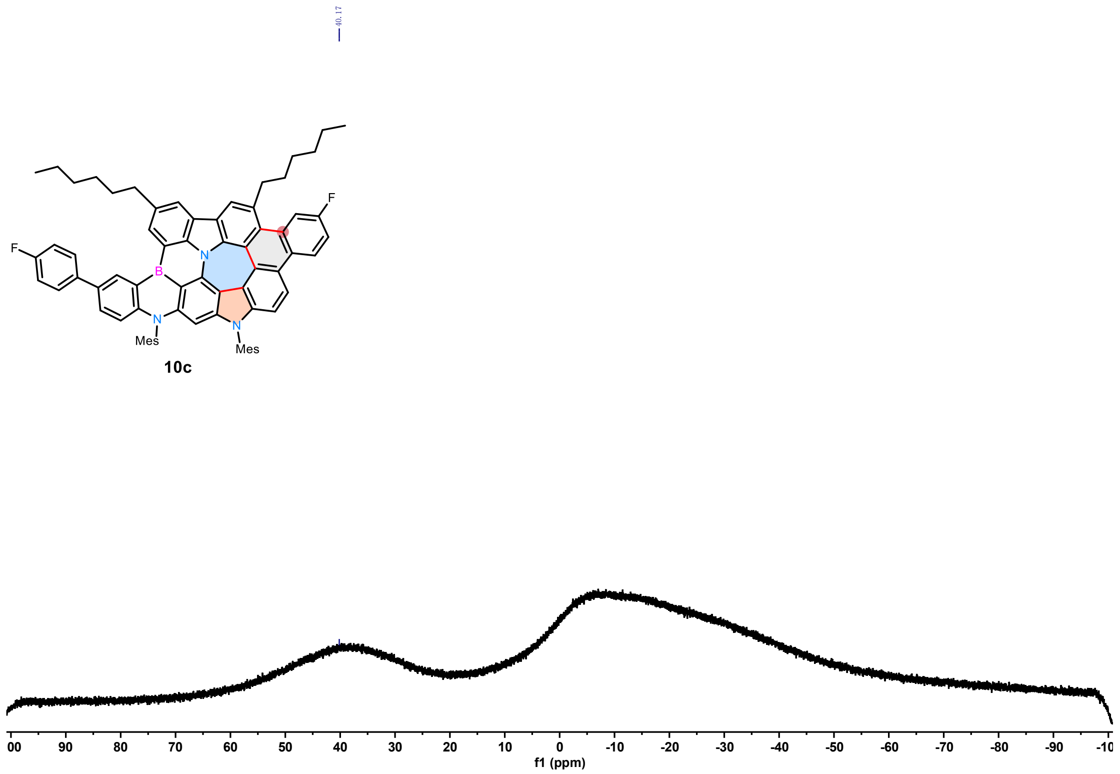

^{11}B NMR spectrum (128 MHz, $CDCl_3/CS_2$, 298 K) of compound **10d**

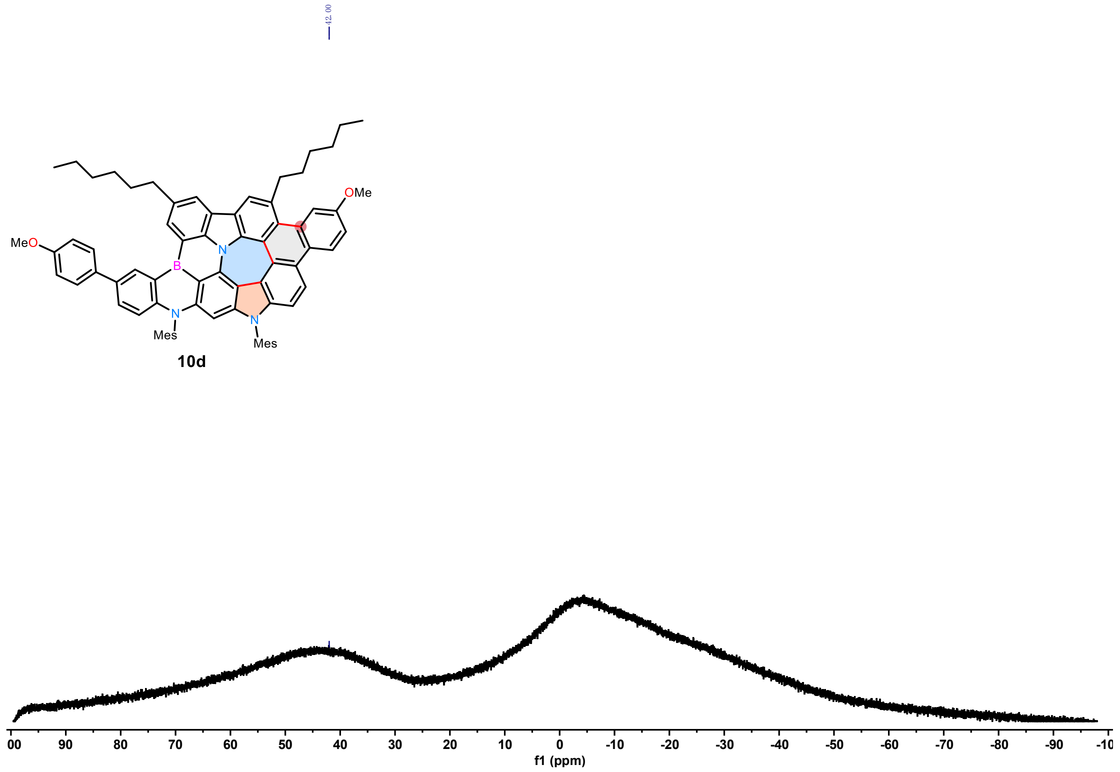

^{11}B NMR spectrum (128 MHz, $CDCl_3/CS_2$, 298 K) of compound **10e**

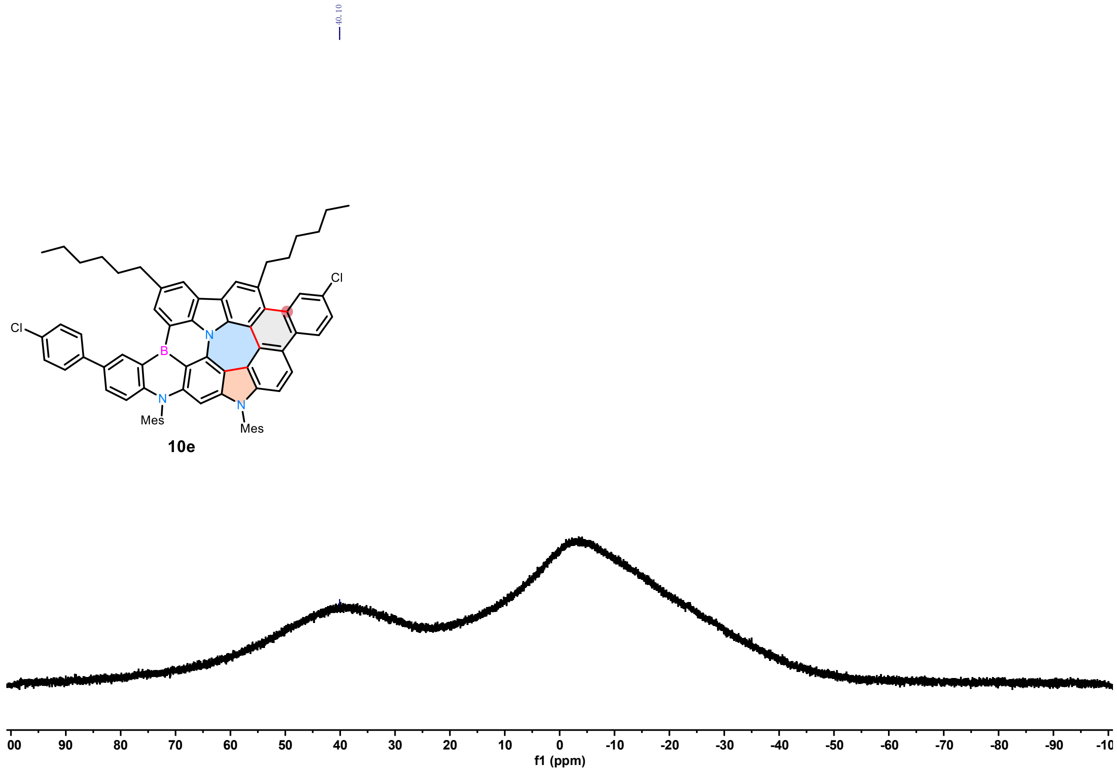

^{11}B NMR spectrum (128 MHz, $CDCl_3/CS_2$, 298 K) of compound **10f**

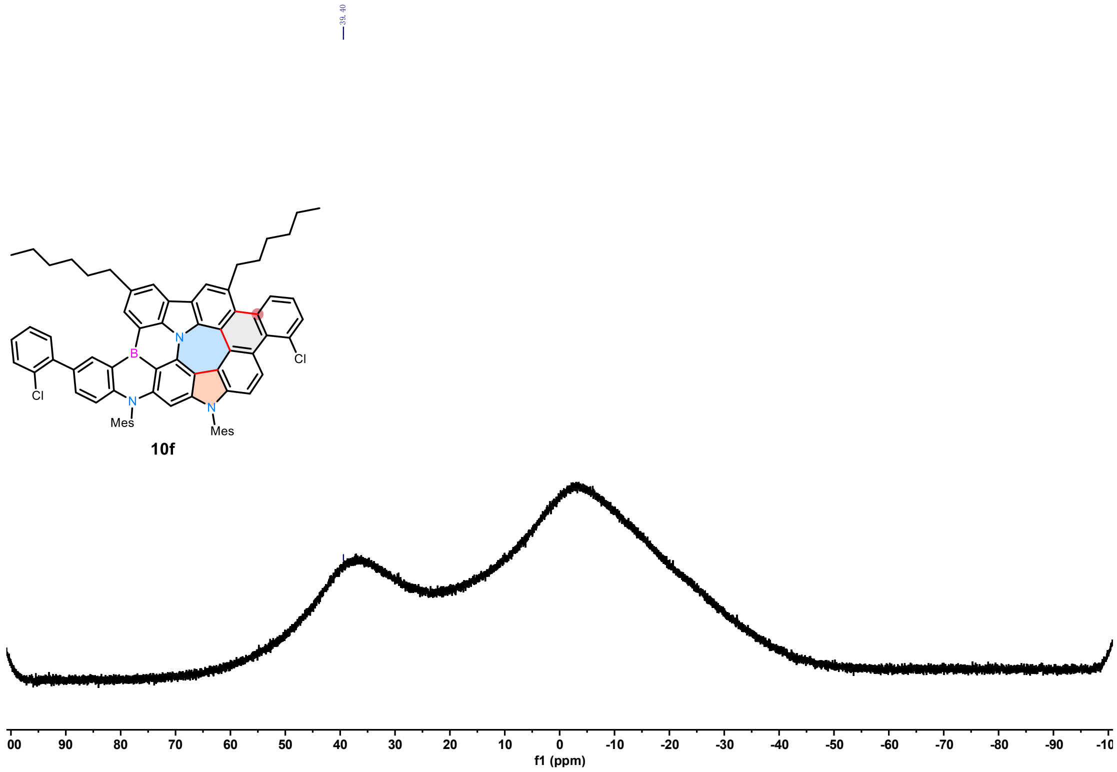

^{11}B NMR spectrum (128 MHz, $CDCl_3/CS_2$, 298 K) of compound **10g**

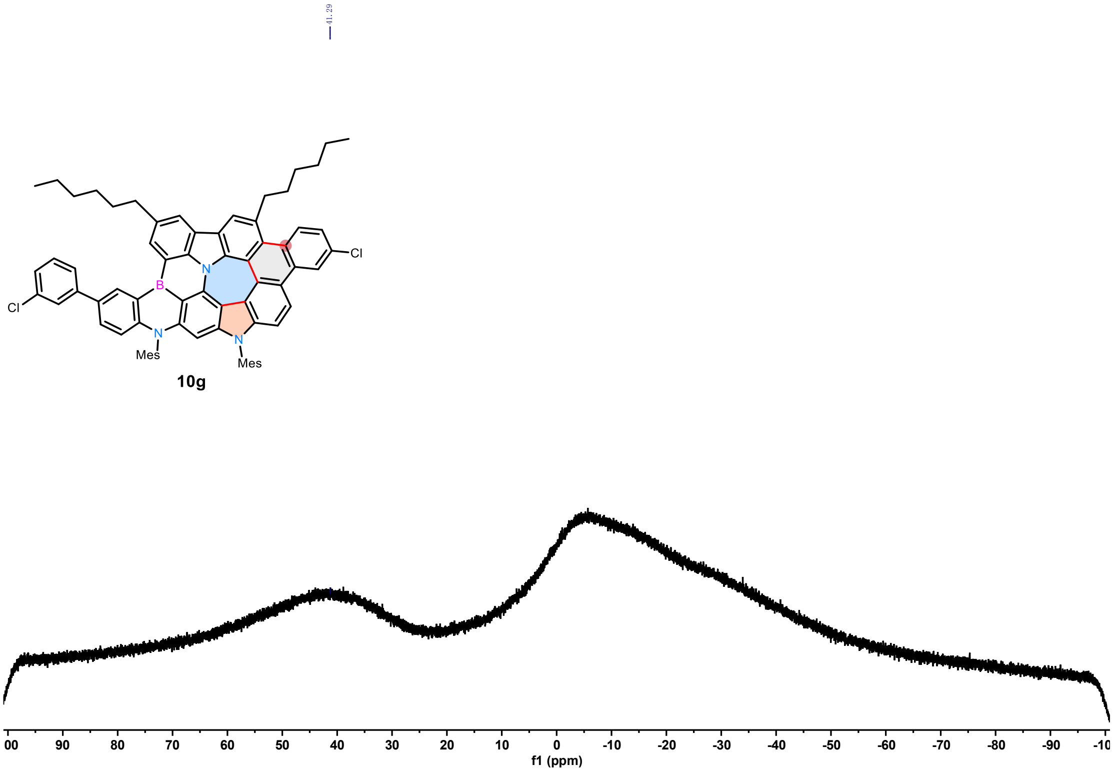

^{11}B NMR spectrum (128 MHz, $CDCl_3/CS_2$, 298 K) of compound **10h**

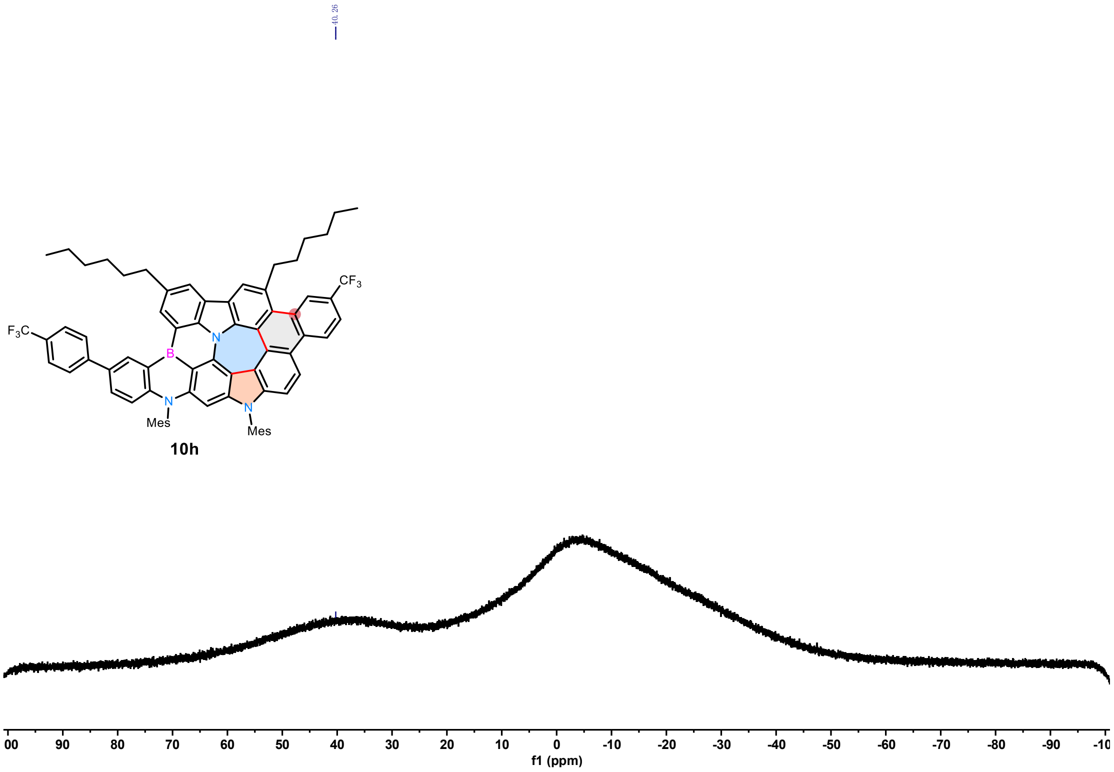

^{11}B NMR spectrum (128 MHz, $CDCl_3/CS_2$, 298 K) of compound **10i**

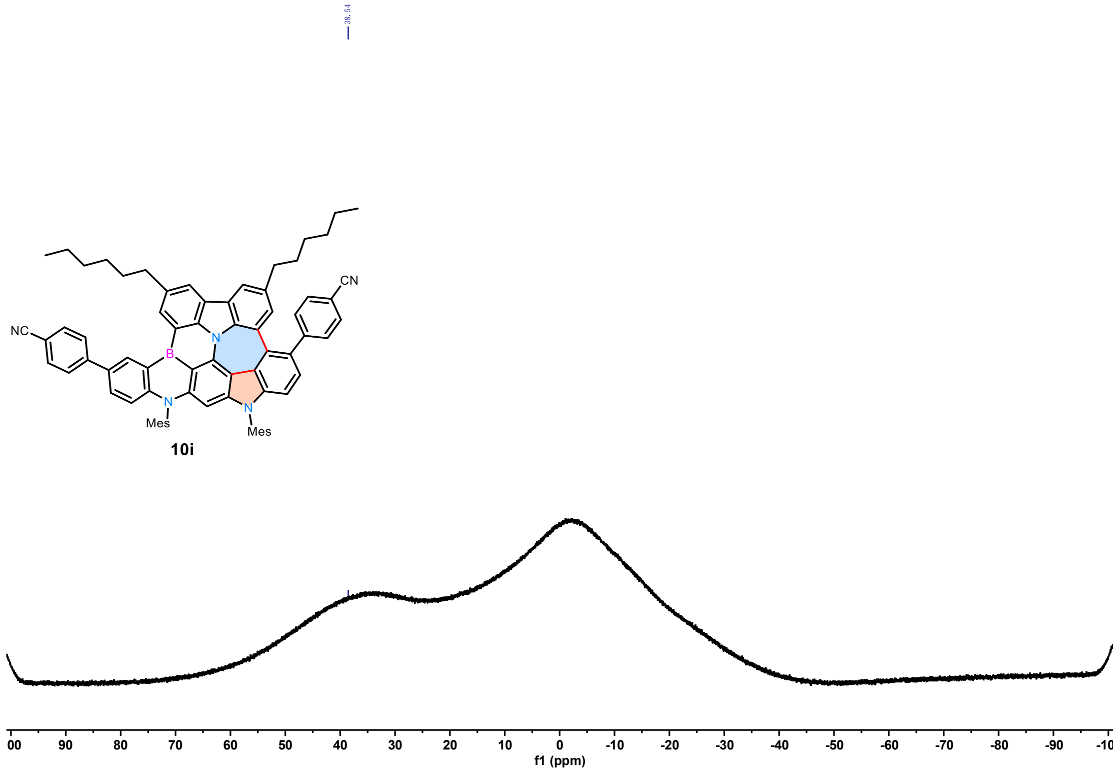

^{11}B NMR spectrum (128 MHz, $CDCl_3/CS_2$, 298 K) of compound **10j**

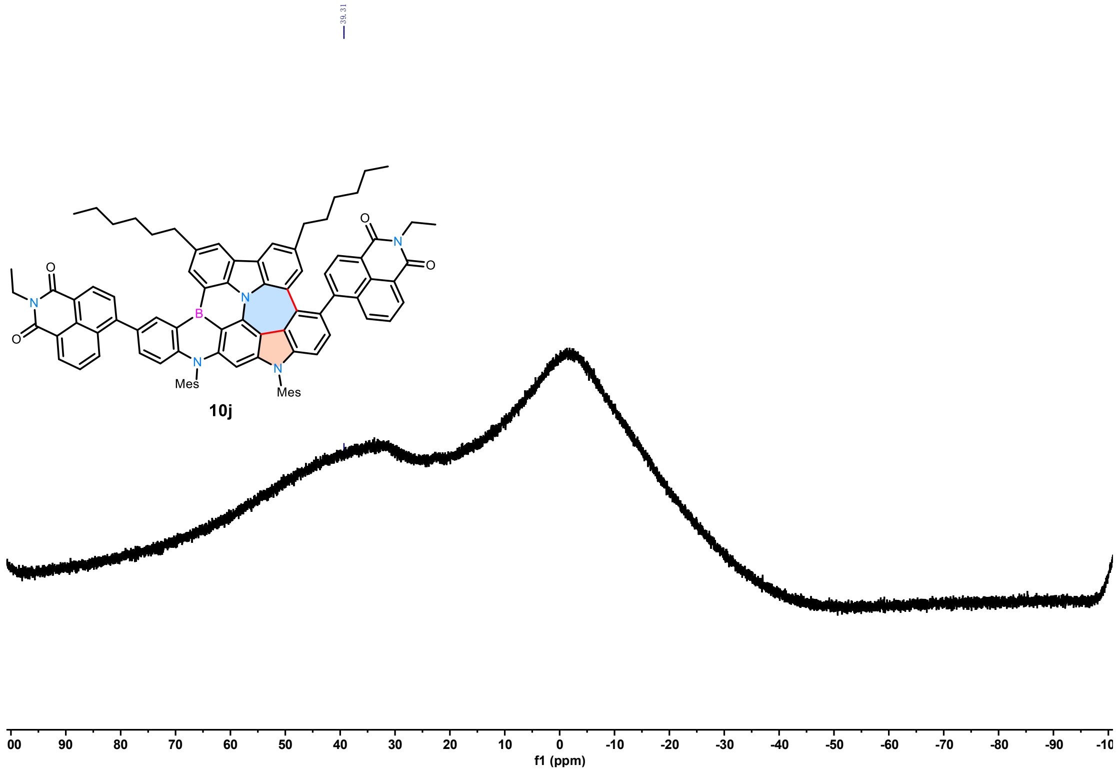

^{11}B NMR spectrum (128 MHz, $CDCl_3$, 298 K) of compound **10k**

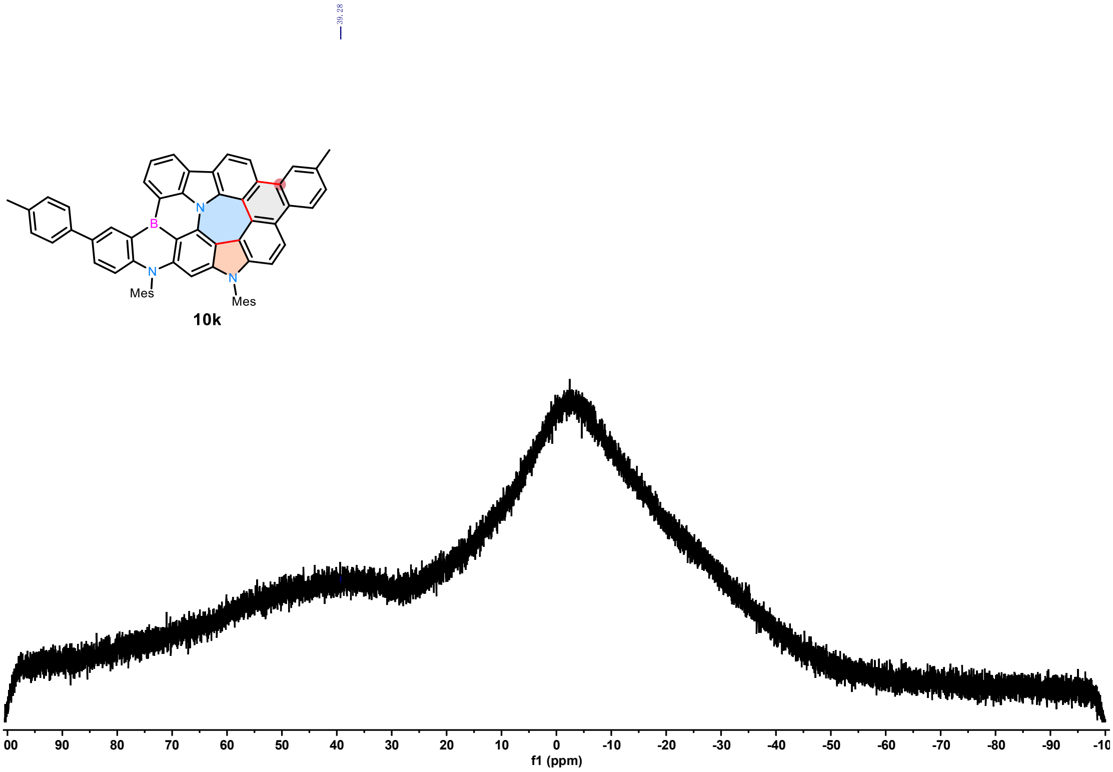

^{11}B NMR spectrum (128 MHz, $CDCl_3/CS_2$, 298 K) of compound **12**

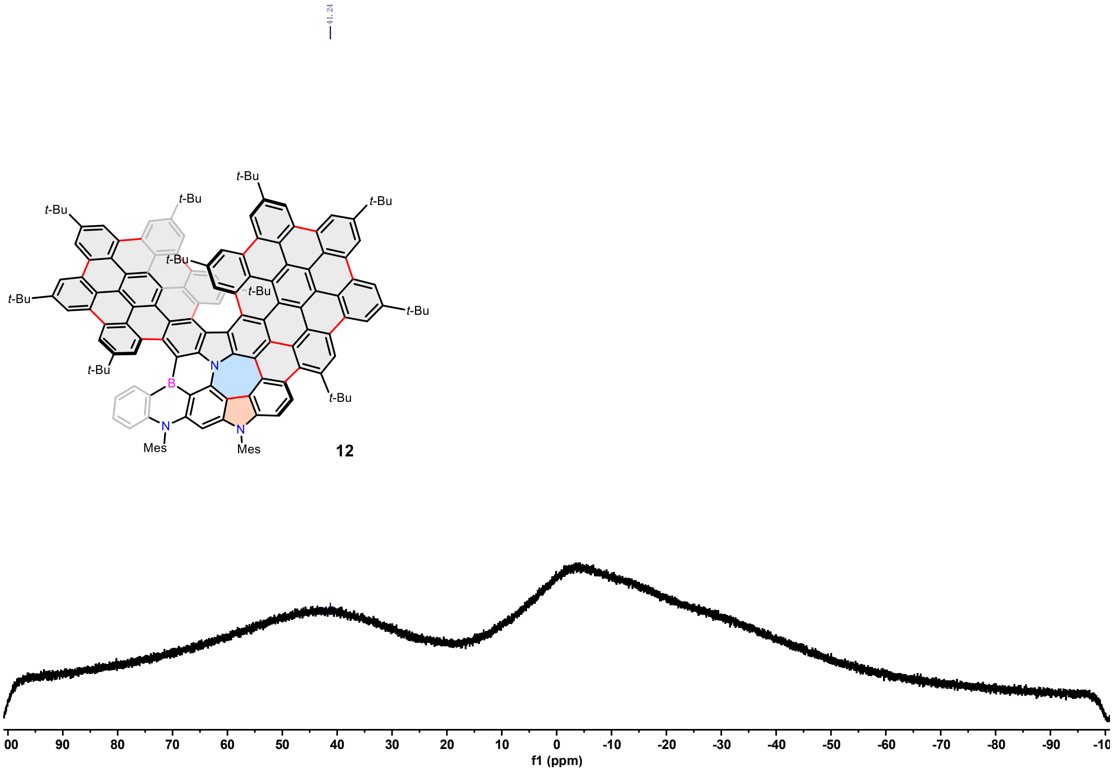

^{11}B NMR spectrum (128 MHz, $CDCl_3/CS_2$, 298 K) of compound **14**

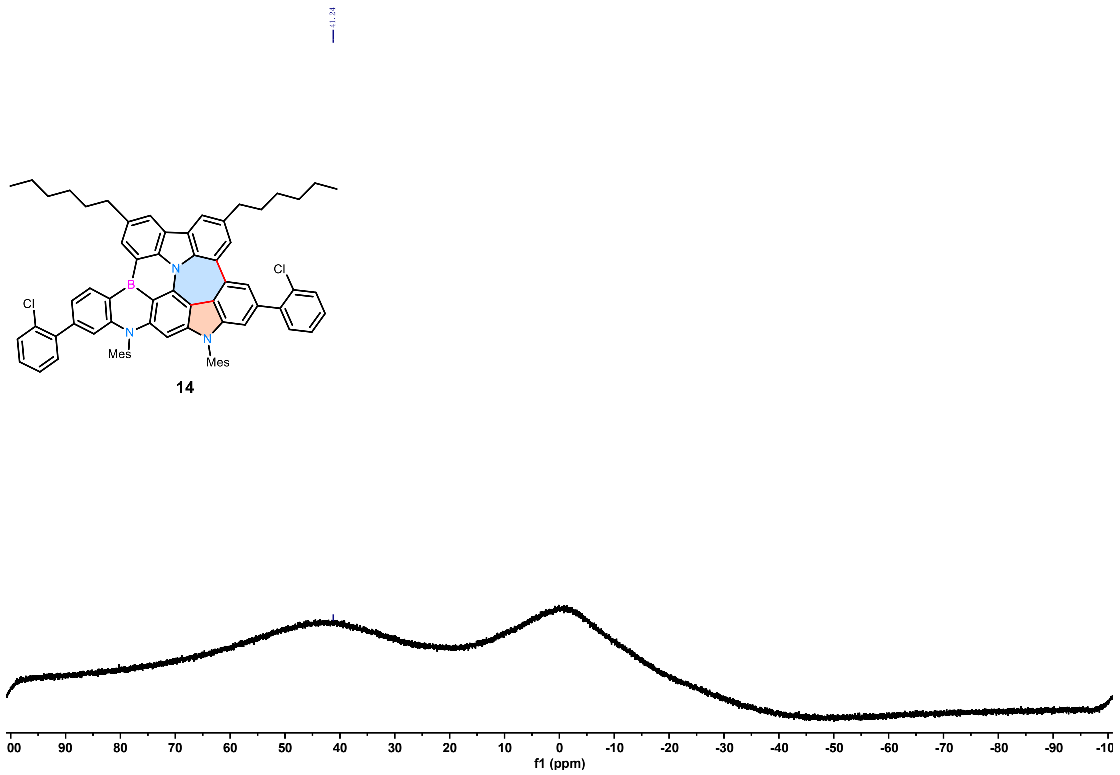

^{11}B NMR spectrum (128 MHz, $CDCl_3/CS_2$, 298 K) of compound **16a**

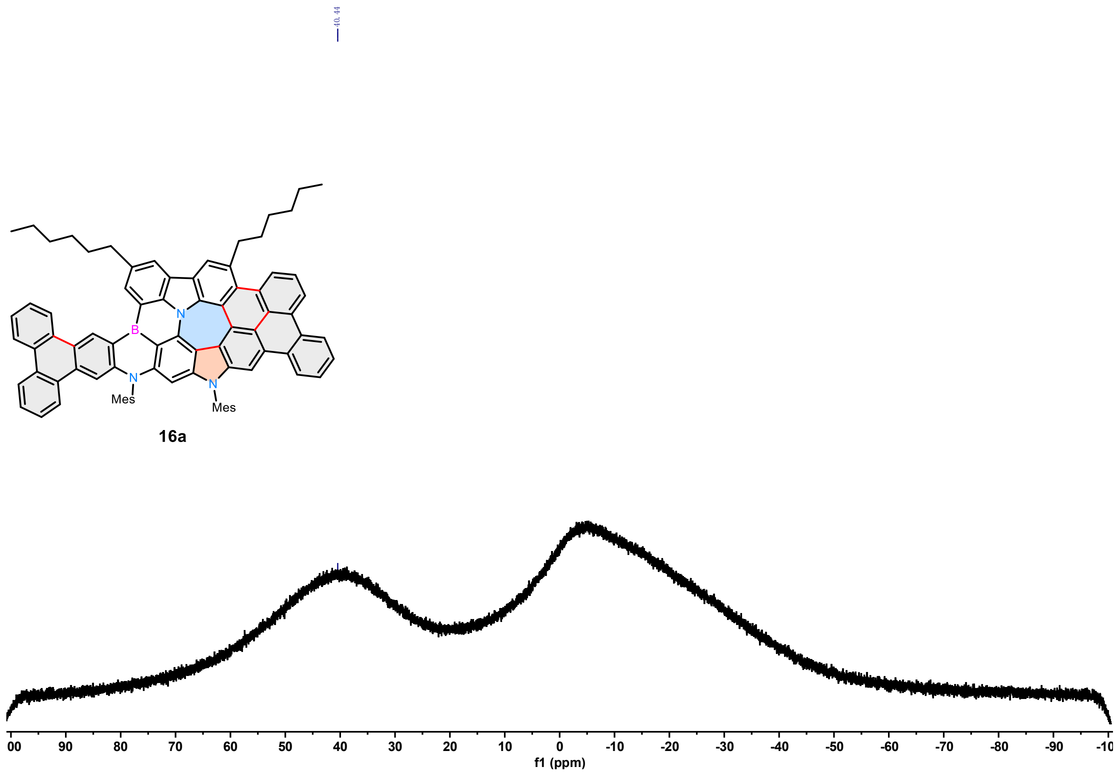

^{11}B NMR spectrum (128 MHz, $CDCl_3/CS_2$, 298 K) of compound **16b**

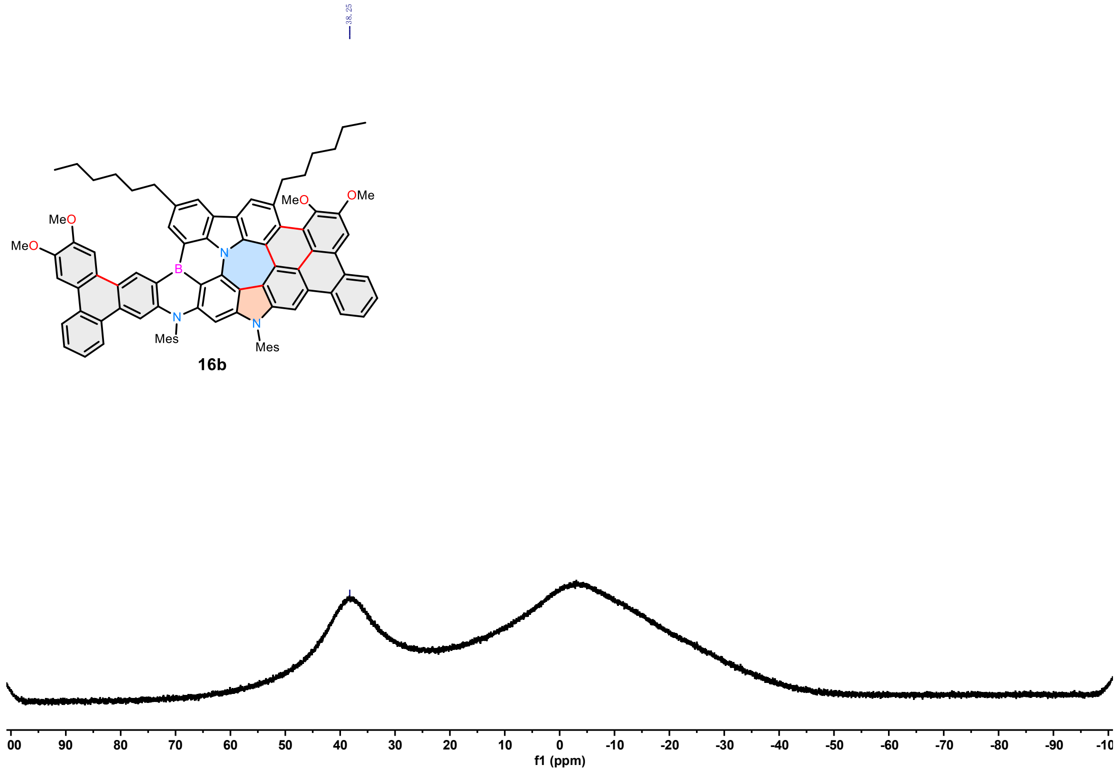

^{11}B NMR spectrum (128 MHz, $CDCl_3/CS_2$, 298 K) of compound **19**

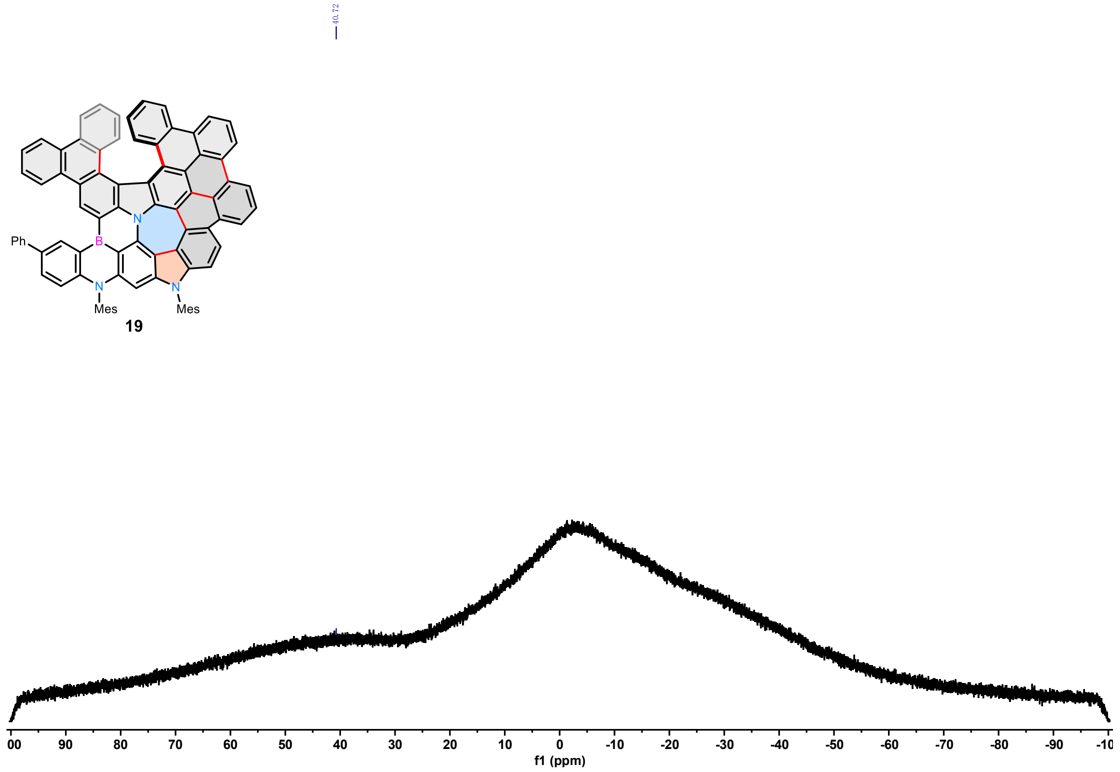

^{11}B NMR spectrum (128 MHz, $CDCl_3/CS_2$, 298 K) of compound **22**

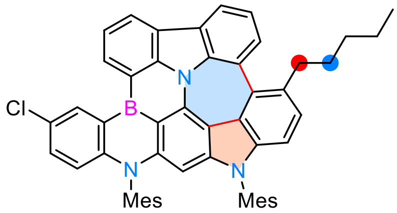

22

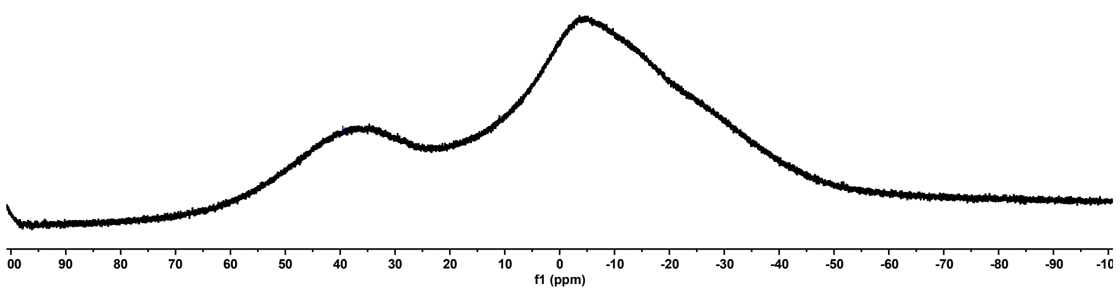

^{11}B NMR spectrum (128 MHz, $CDCl_3/CS_2$, 298 K) of compound **23**

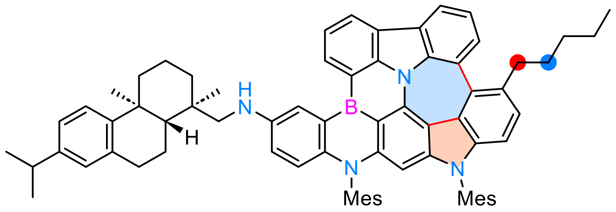

23

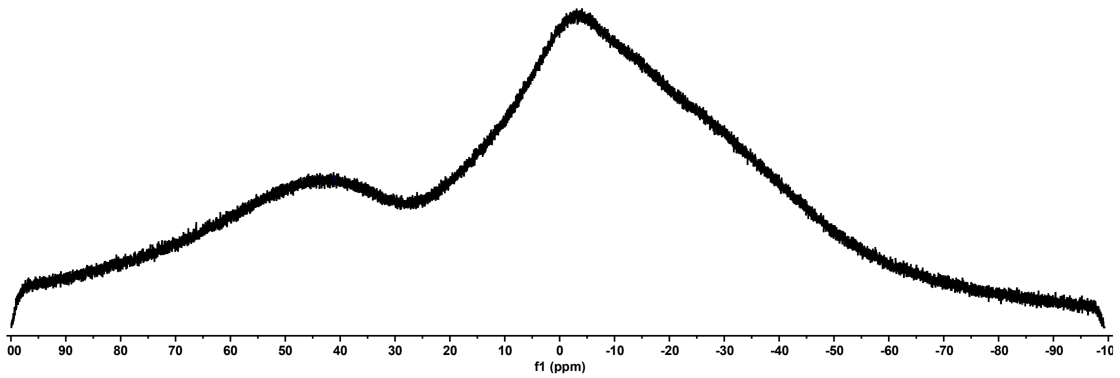

^{11}B NMR spectrum (128 MHz, $CDCl_3/CS_2$, 298 K) of compound **25a**

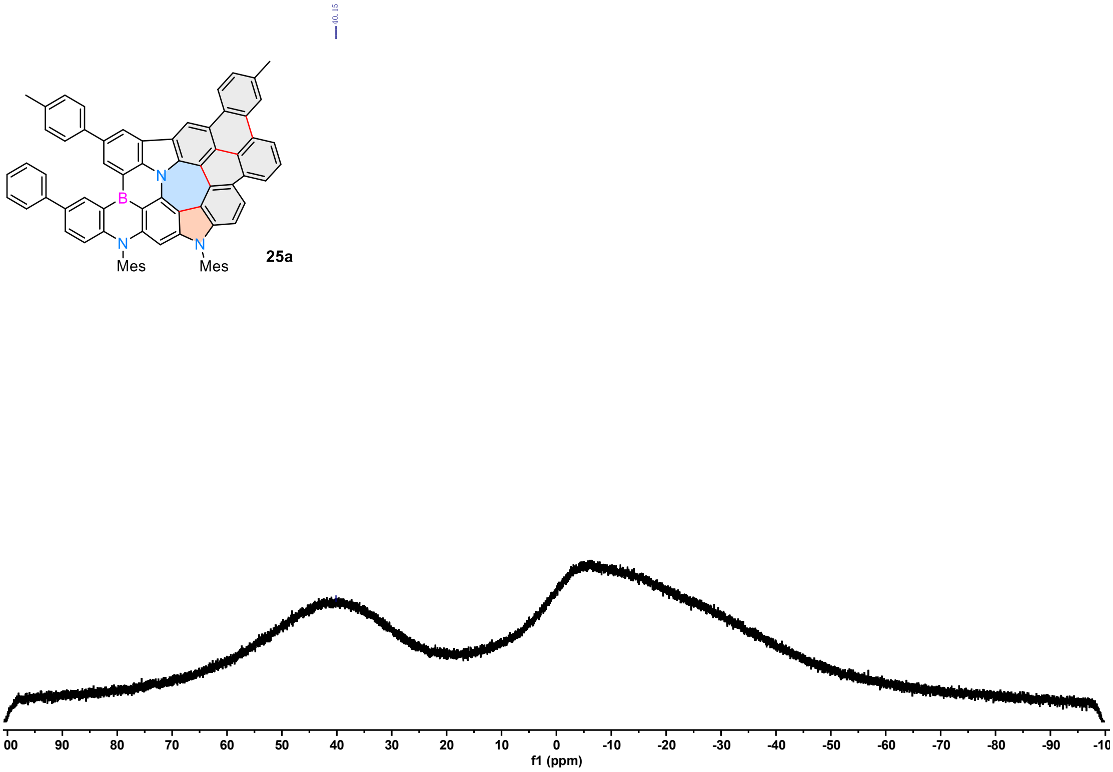

^{11}B NMR spectrum (128 MHz, $CDCl_3/CS_2$, 298 K) of compound **25b**

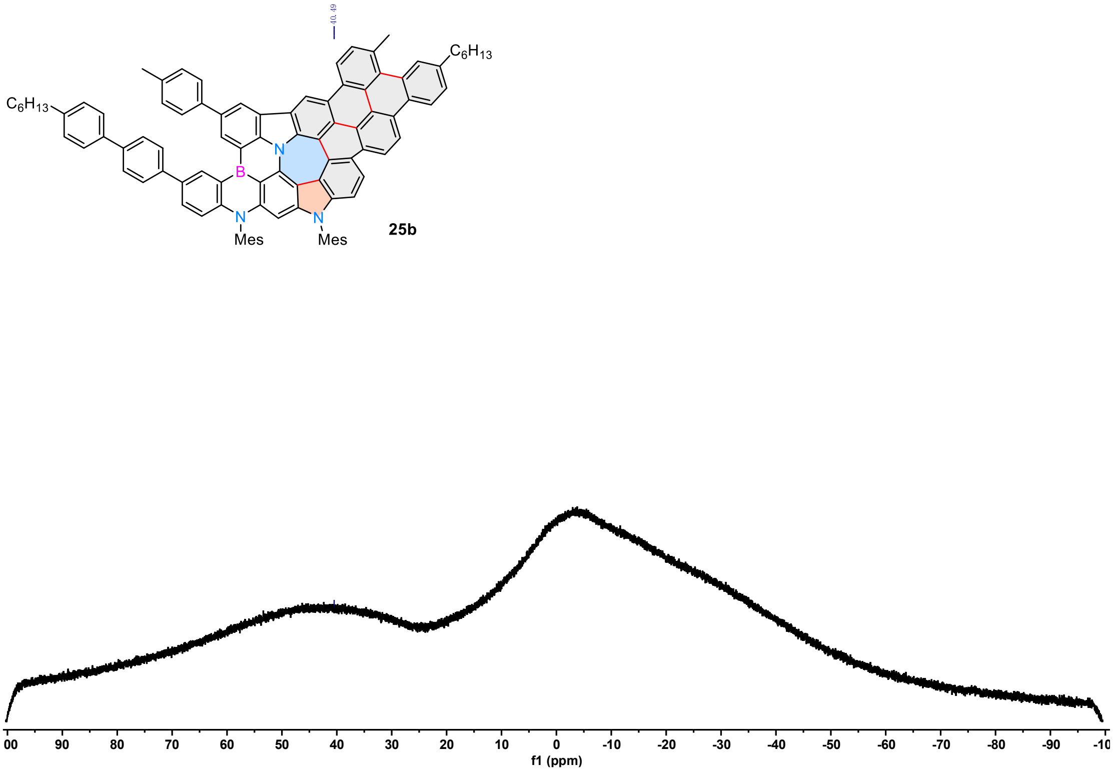

^{11}B NMR spectrum (128 MHz, $CDCl_3/CS_2$, 298 K) of compound **26**

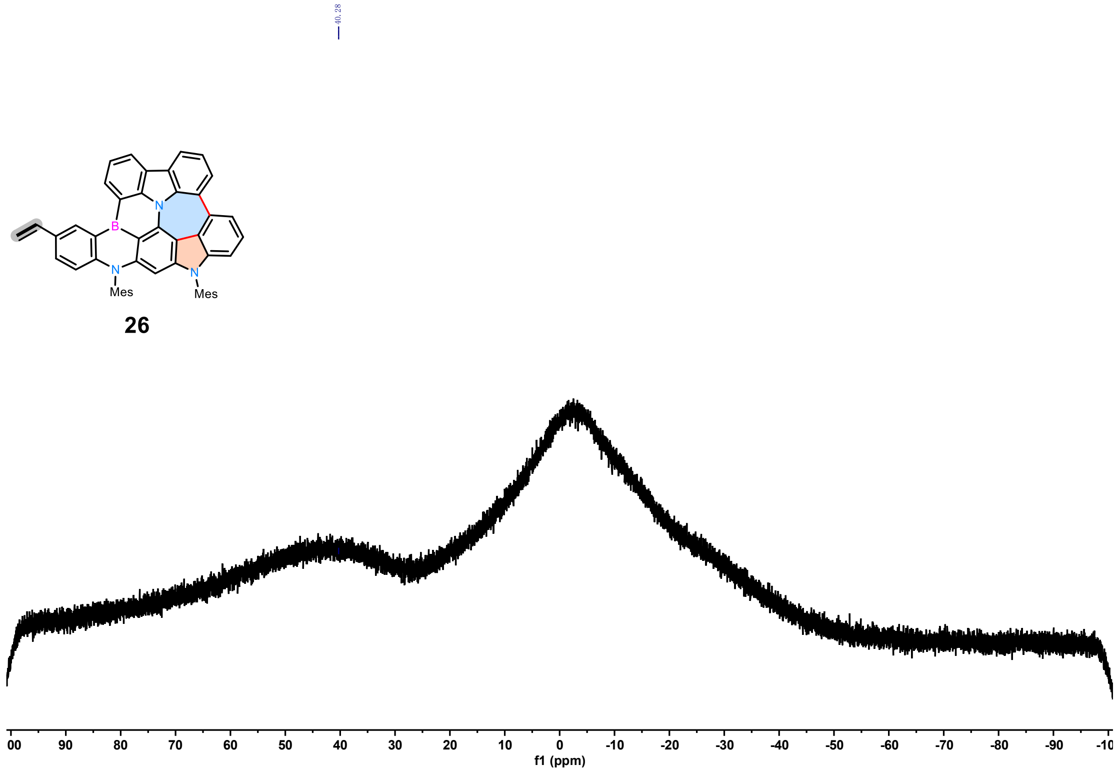

^{11}B NMR spectrum (128 MHz, $CDCl_3/CS_2$, 298 K) of compound **27**

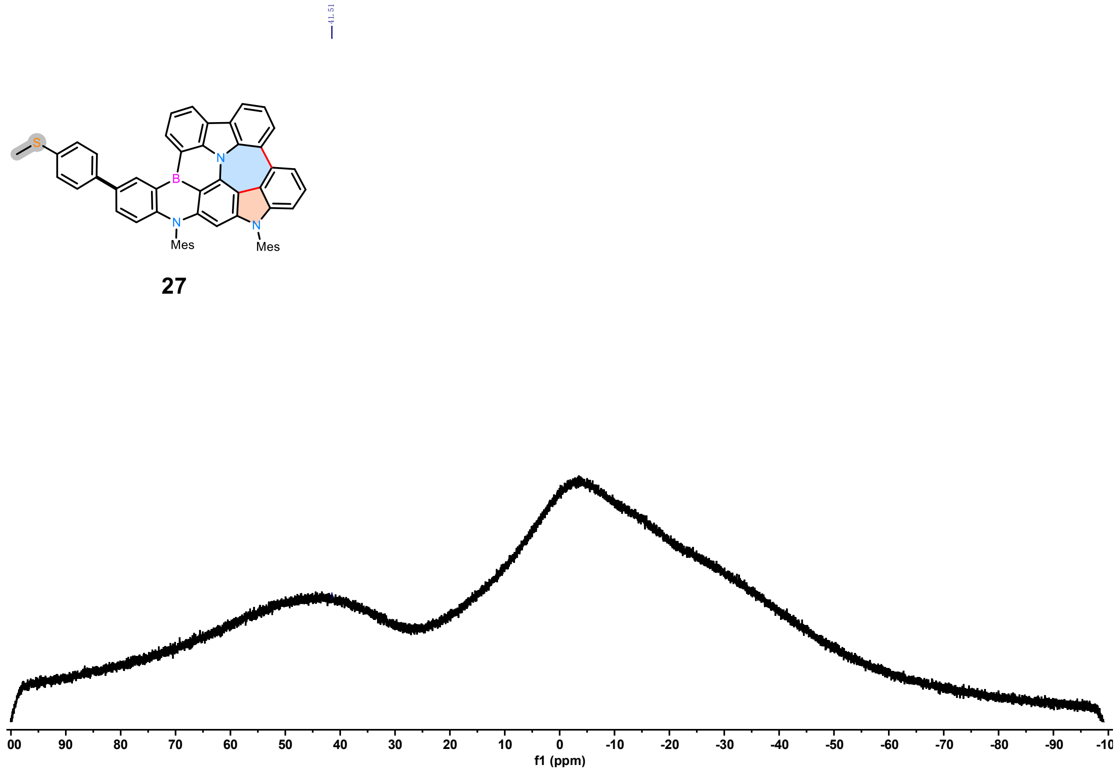

^{11}B NMR spectrum (128 MHz, $CDCl_3/CS_2$, 298 K) of compound **28**

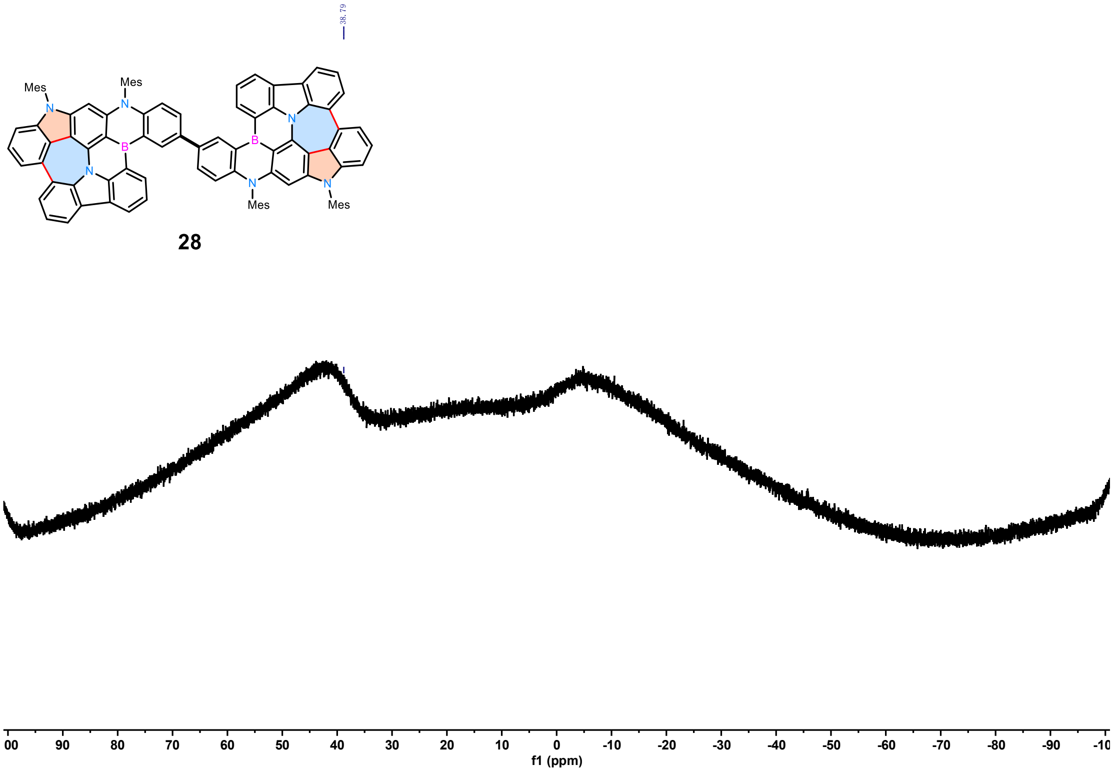

28

^{11}B NMR spectrum (128 MHz, $CDCl_3/CS_2$, 298 K) of compound **29a**

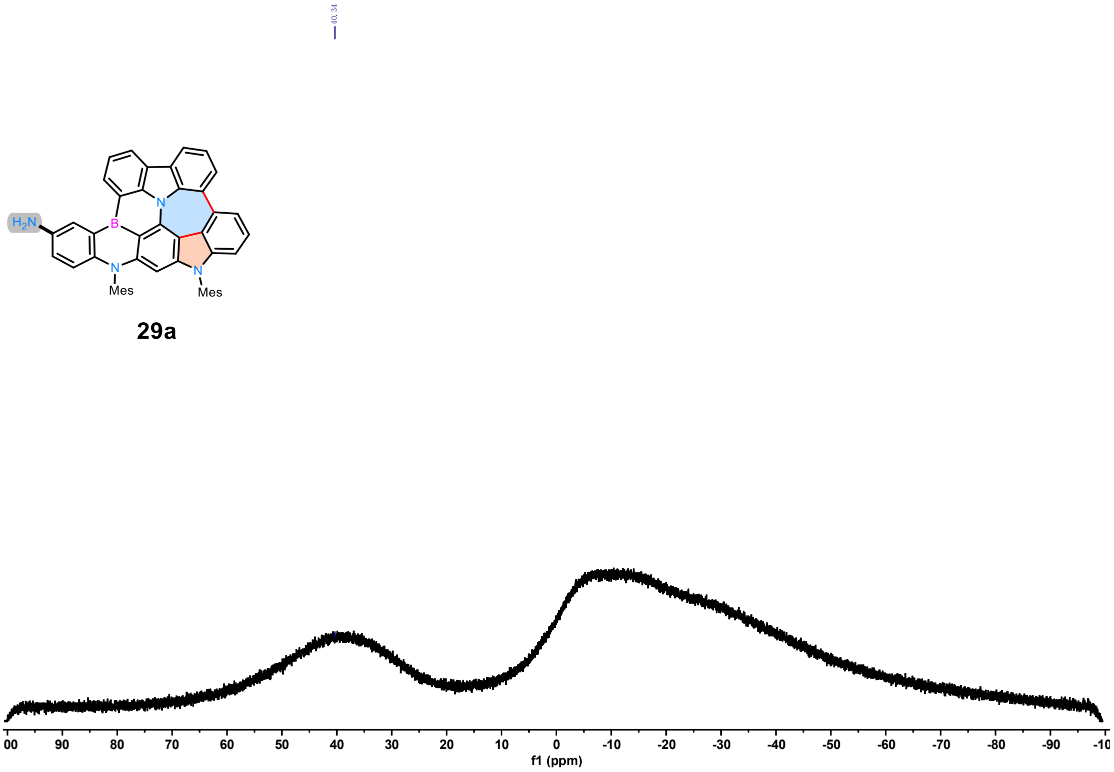

29a

^{11}B NMR spectrum (128 MHz, $CDCl_3/CS_2$, 298 K) of compound **29b**

40.74

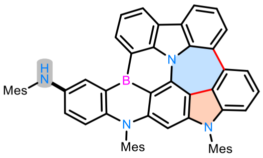

29b

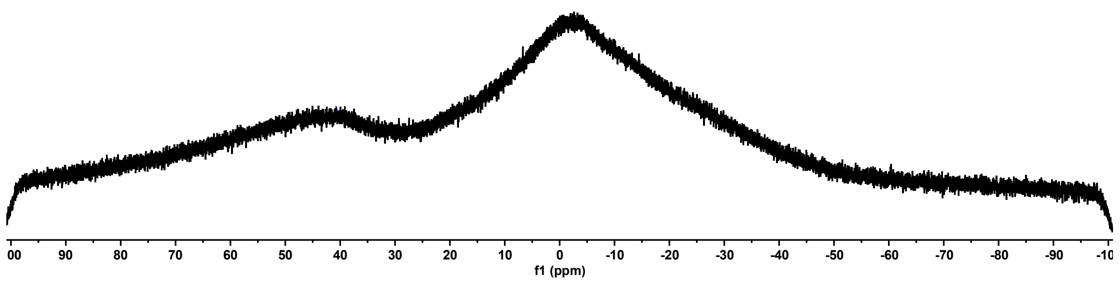

^{11}B NMR spectrum (128 MHz, $CDCl_3/CS_2$, 298 K) of compound **30**

40.30

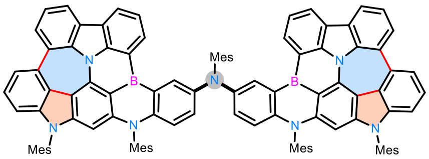

30

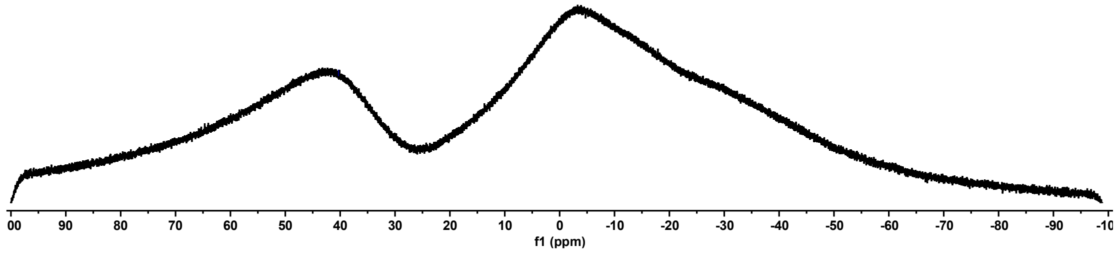

^{11}B NMR spectrum (128 MHz, $CDCl_3/CS_2$, 298 K) of compound **31**

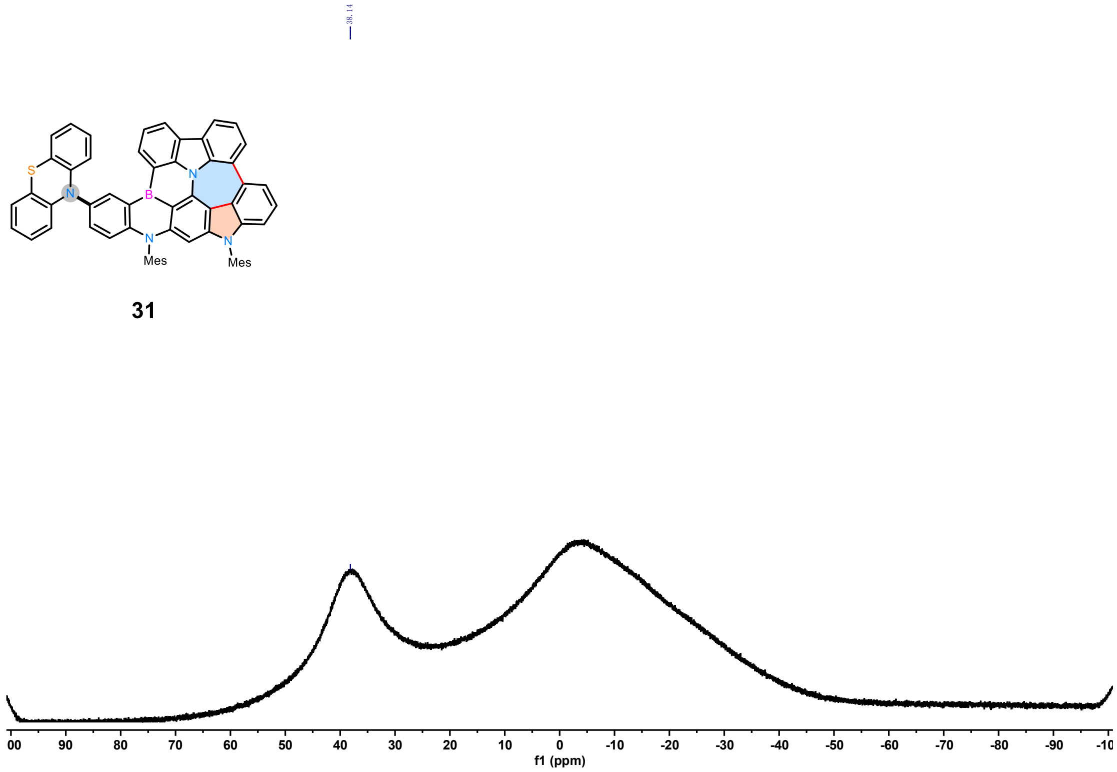

^{11}B NMR spectrum (128 MHz, $CDCl_3/CS_2$, 298 K) of compound **32**

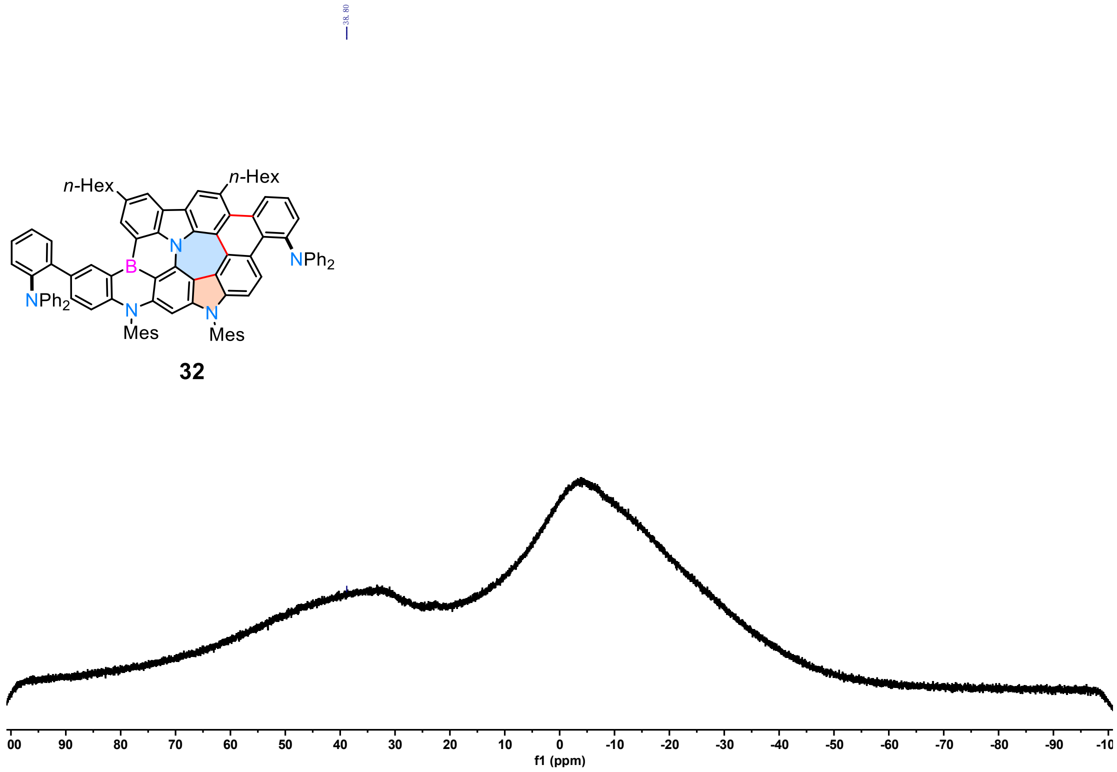

^{11}B NMR spectrum (128 MHz, $CDCl_3/CS_2$, 298 K) of compound **33**

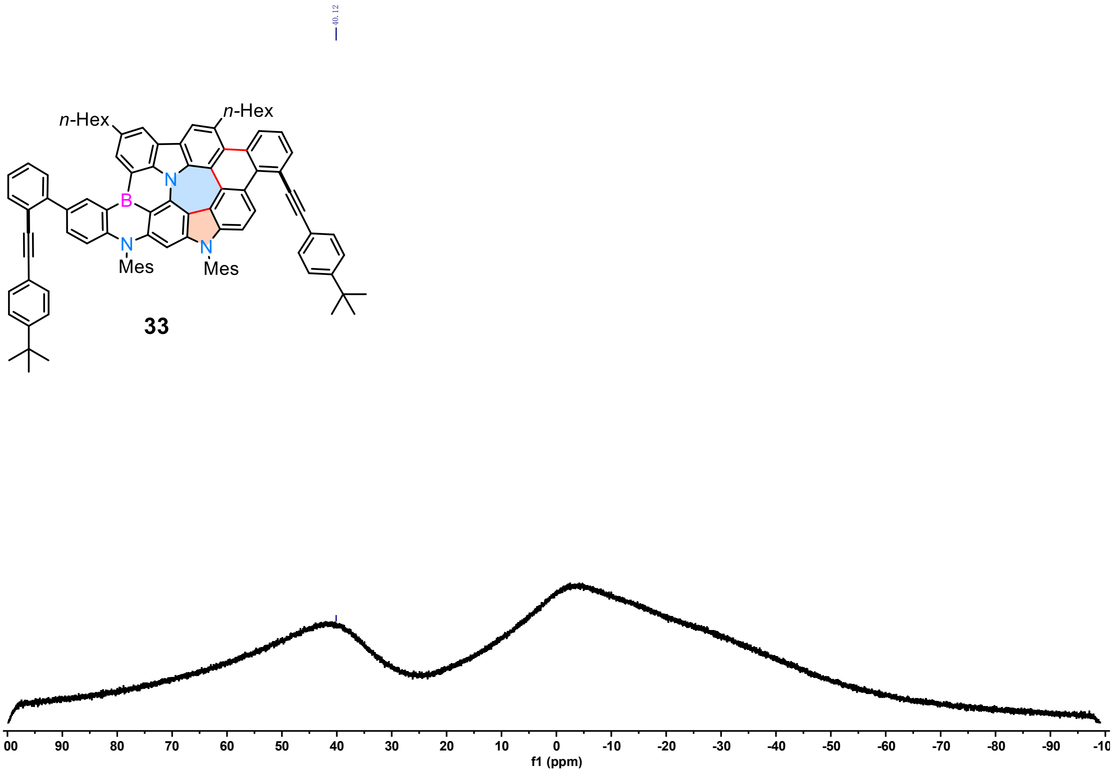

^{11}B NMR spectrum (128 MHz, $CDCl_3/CS_2$, 298 K) of compound **34**

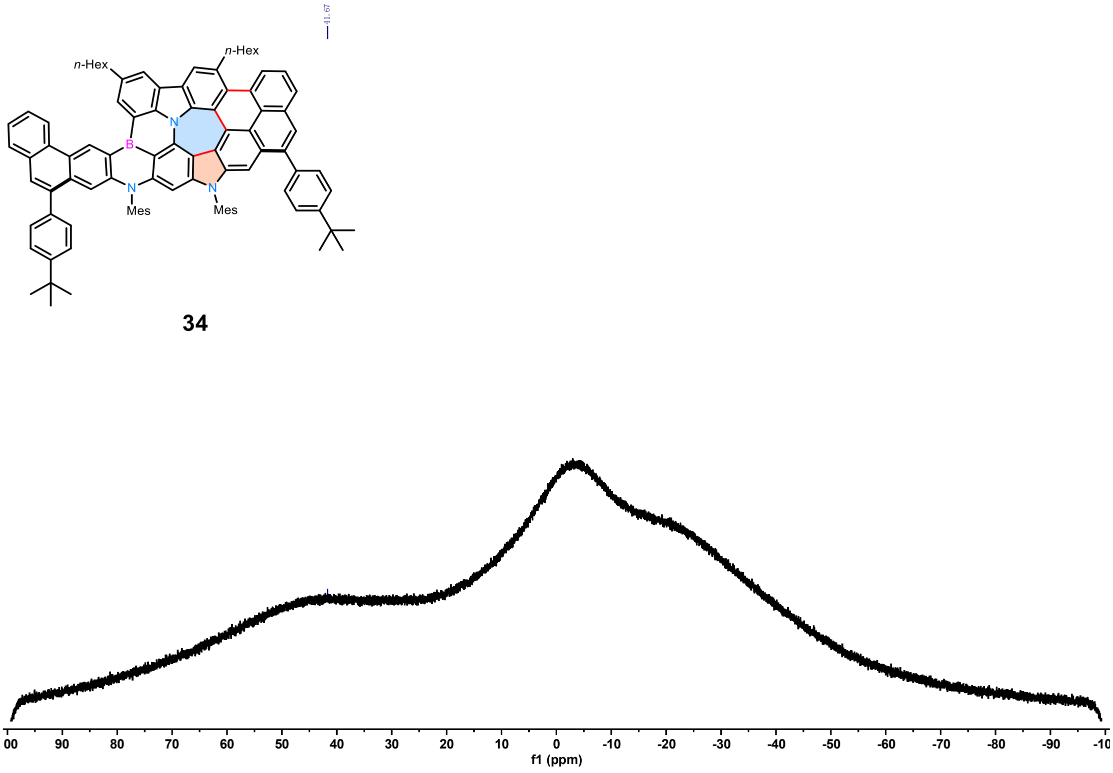

^{11}B NMR spectrum (128 MHz, $CDCl_3/CS_2$, 298 K) of compound **35**

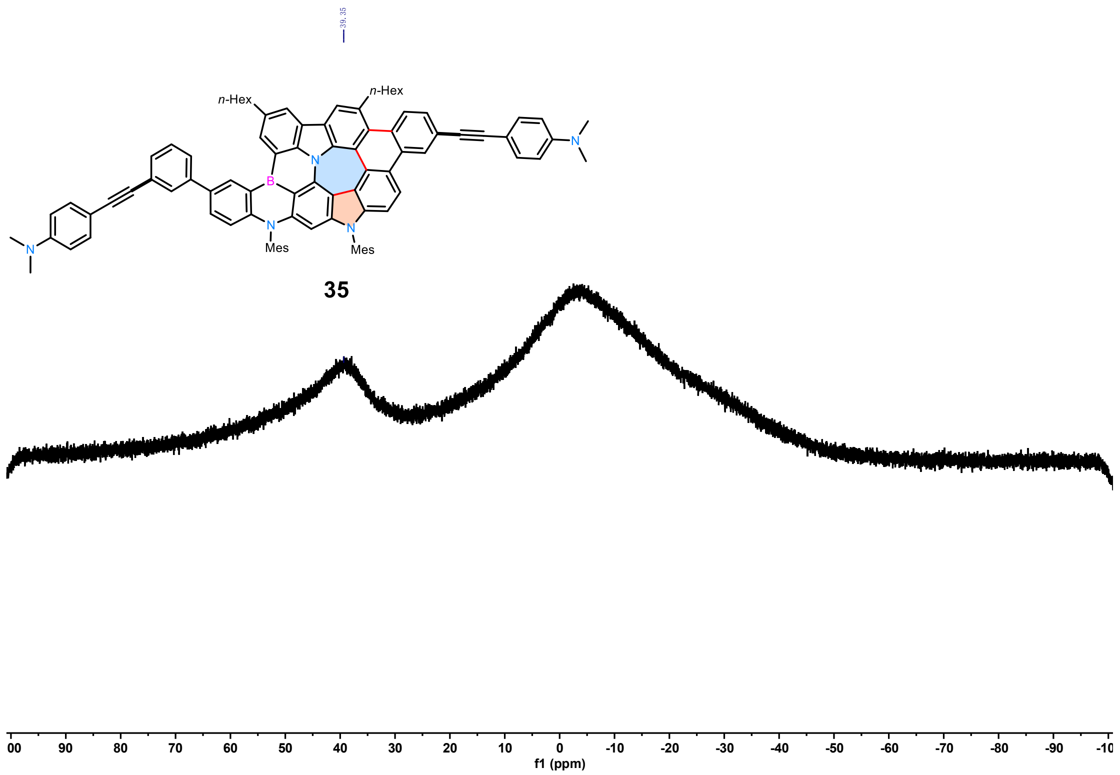

^{11}B NMR spectrum (128 MHz, $CDCl_3/CS_2$, 298 K) of compound **36**

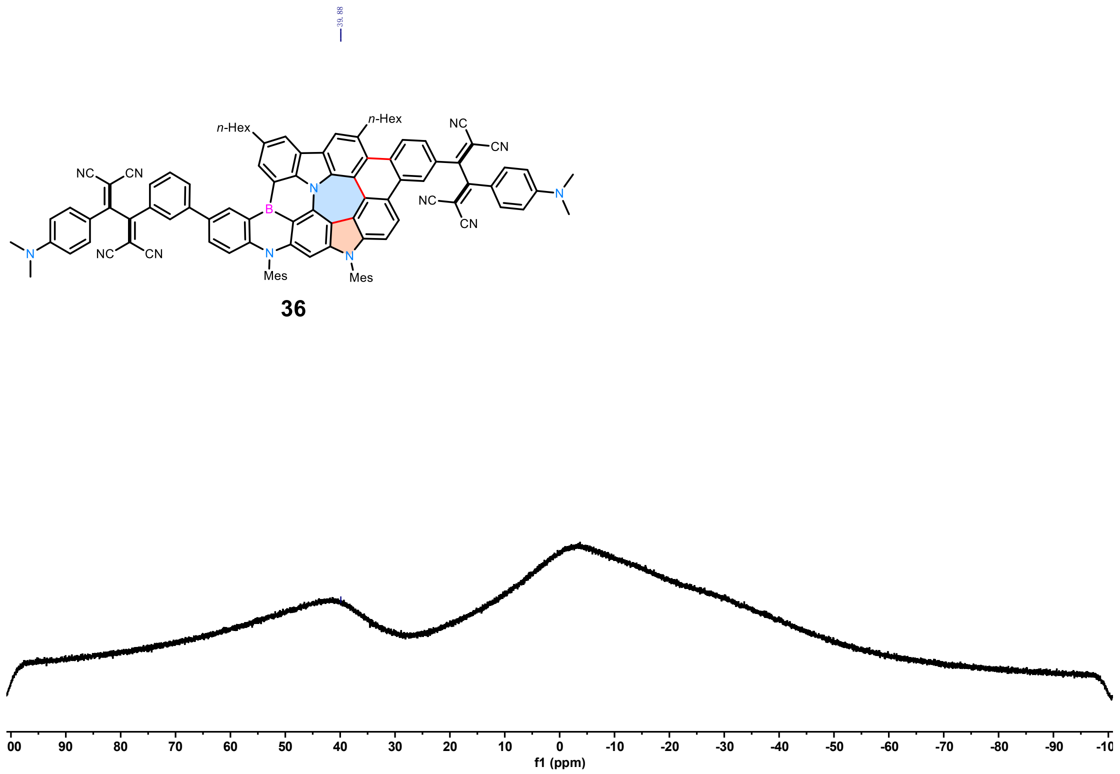

^{1}H NMR spectrum (400 MHz, $CDCl_3$, 298 K) of compound **S1**

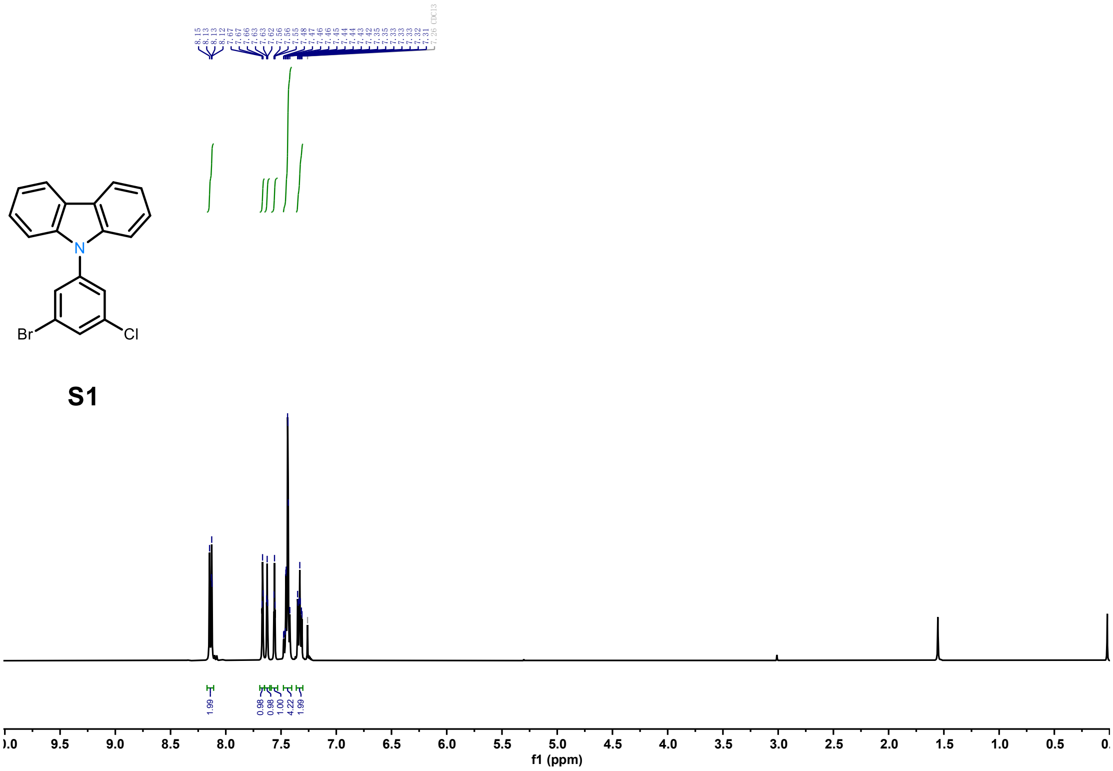

^{13}C NMR spectrum (101 MHz, $CDCl_3$, 298 K) of compound **S1**

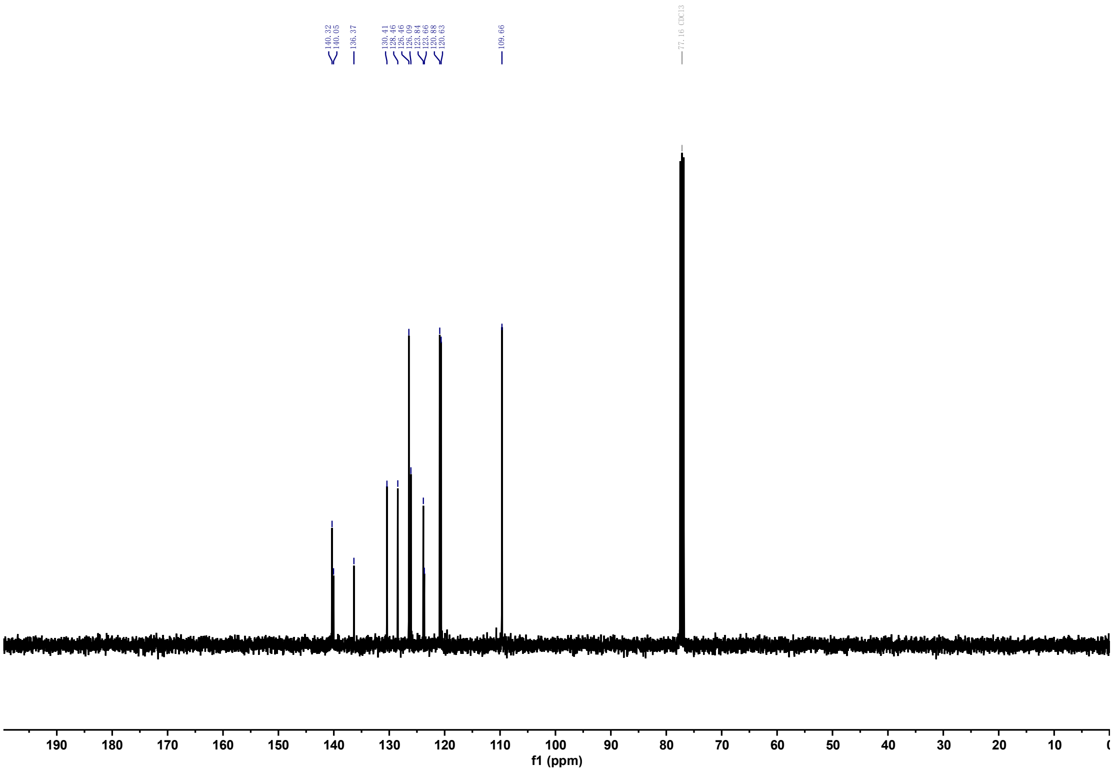

^{1}H NMR spectrum (400 MHz, $CDCl_3$, 298 K) of compound **S2**

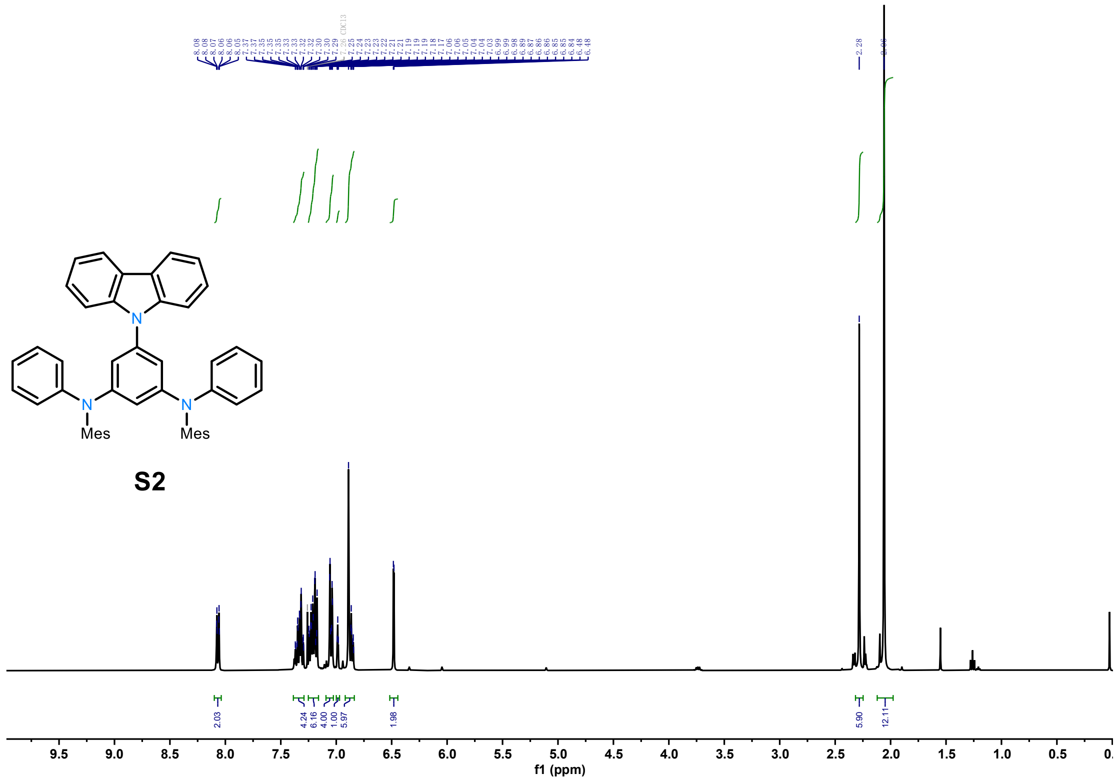

^{13}C NMR spectrum (101 MHz, $CDCl_3$, 298 K) of compound **S2**

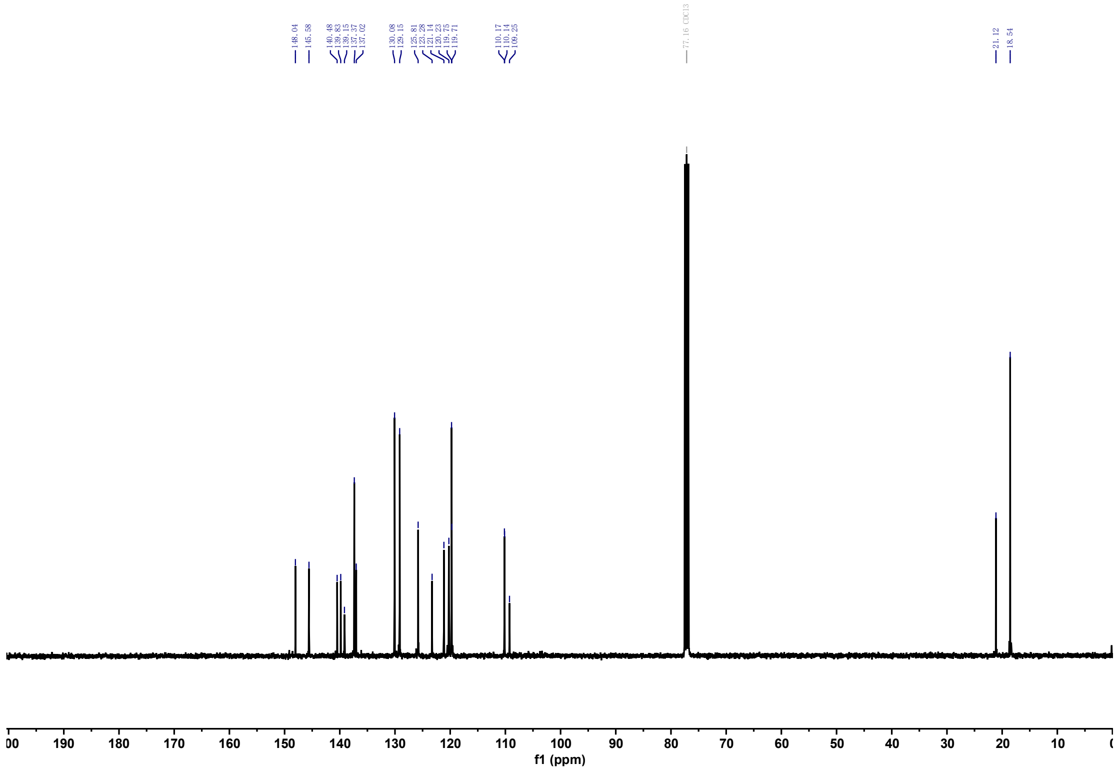

^{1}H NMR spectrum (400 MHz, $CDCl_3$, 298 K) of compound **S3**

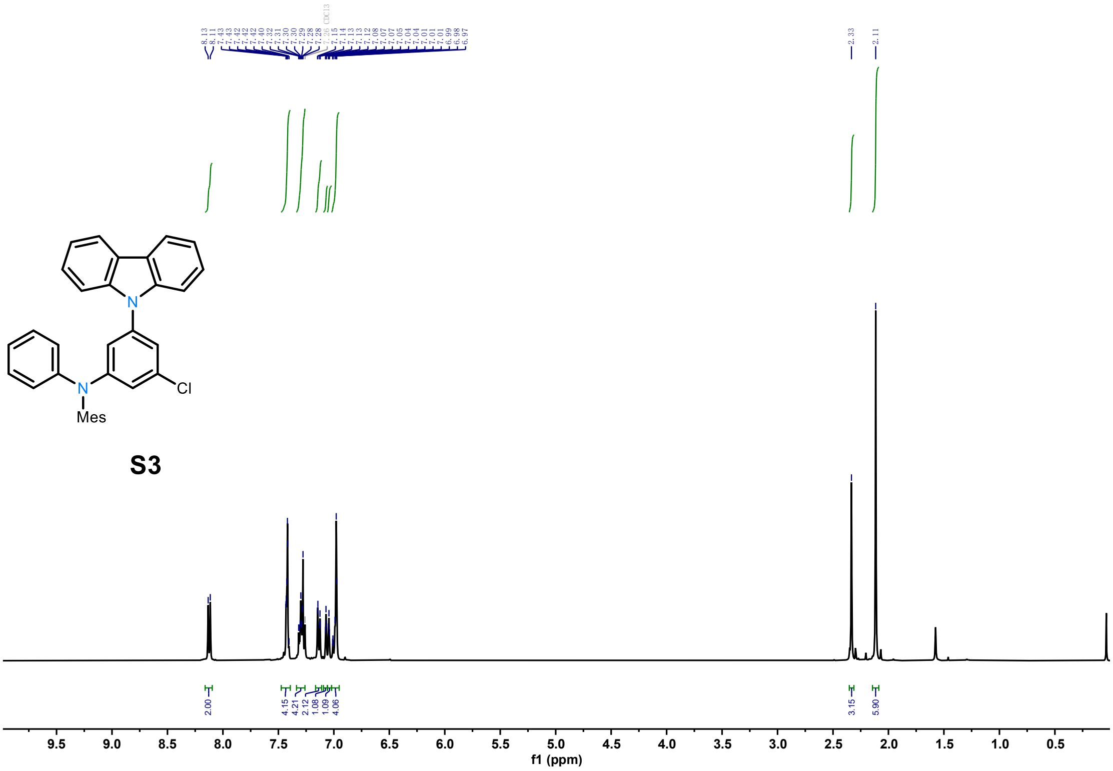

^{13}C NMR spectrum (101 MHz, $CDCl_3$, 298 K) of compound **S3**

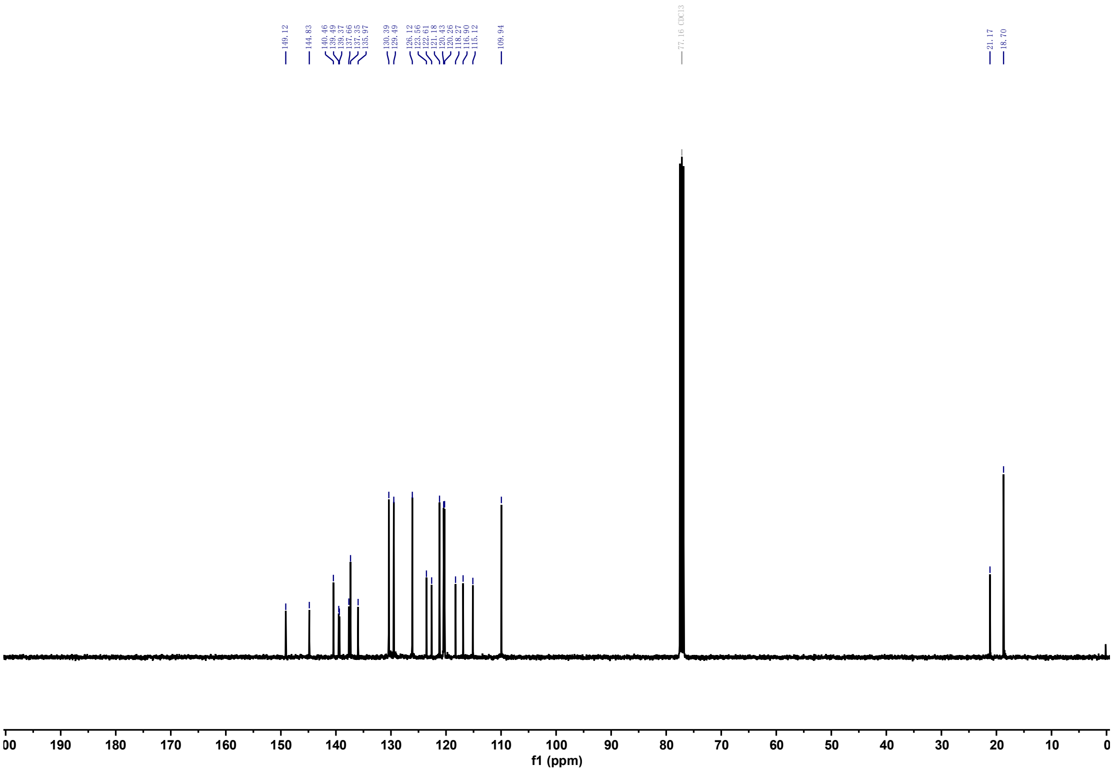

^{1}H NMR spectrum (400 MHz, $CDCl_3$, 298 K) of compound **S4**

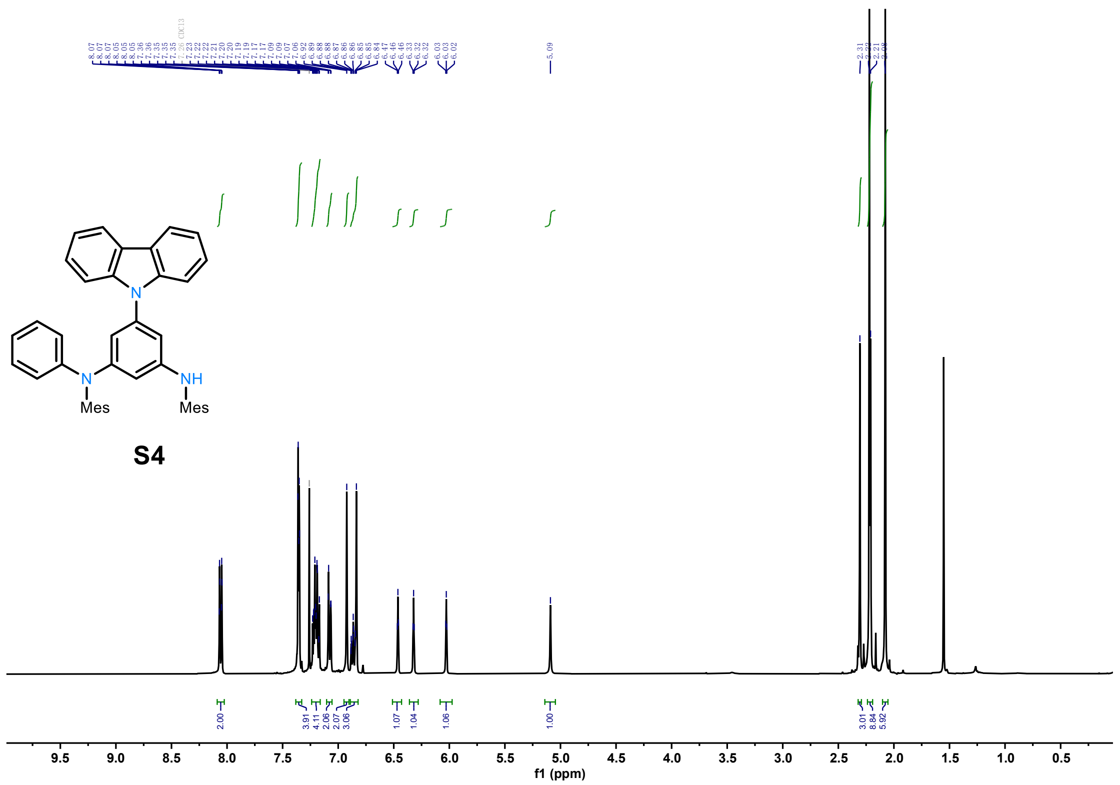

^{13}C NMR spectrum (101 MHz, $CDCl_3$, 298 K) of compound **S4**

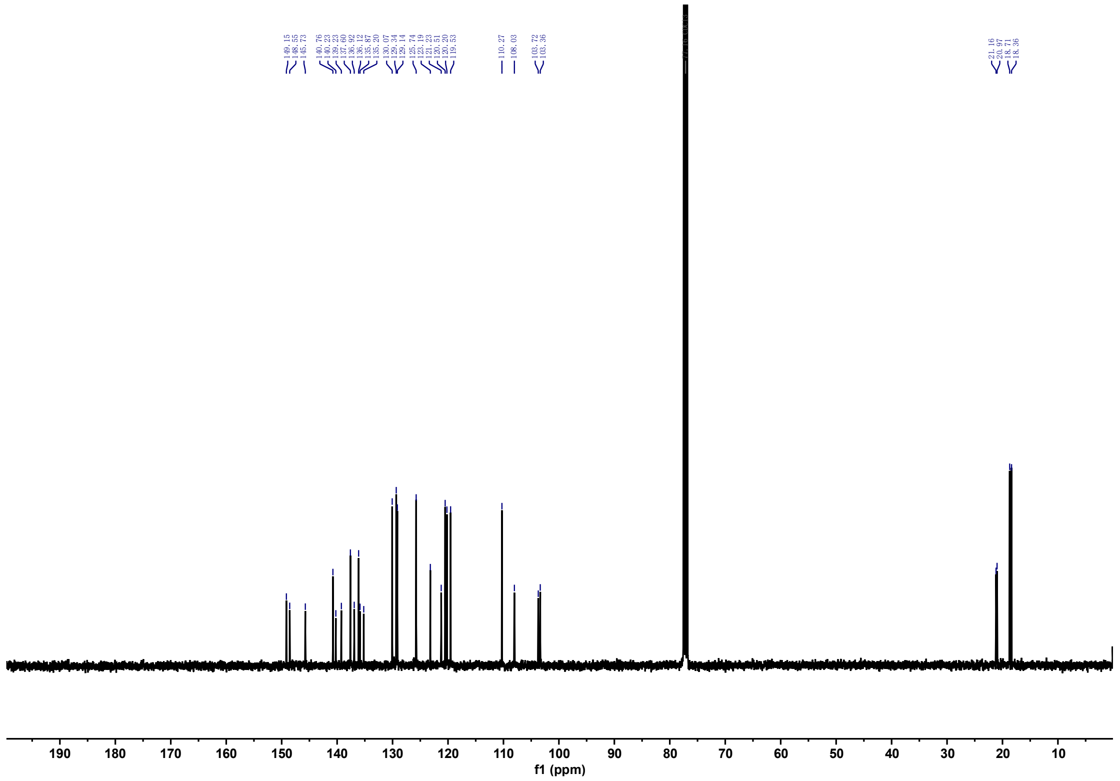

^{1}H NMR spectrum (600 MHz, $CDCl_3$, 298 K) of compound **S5**

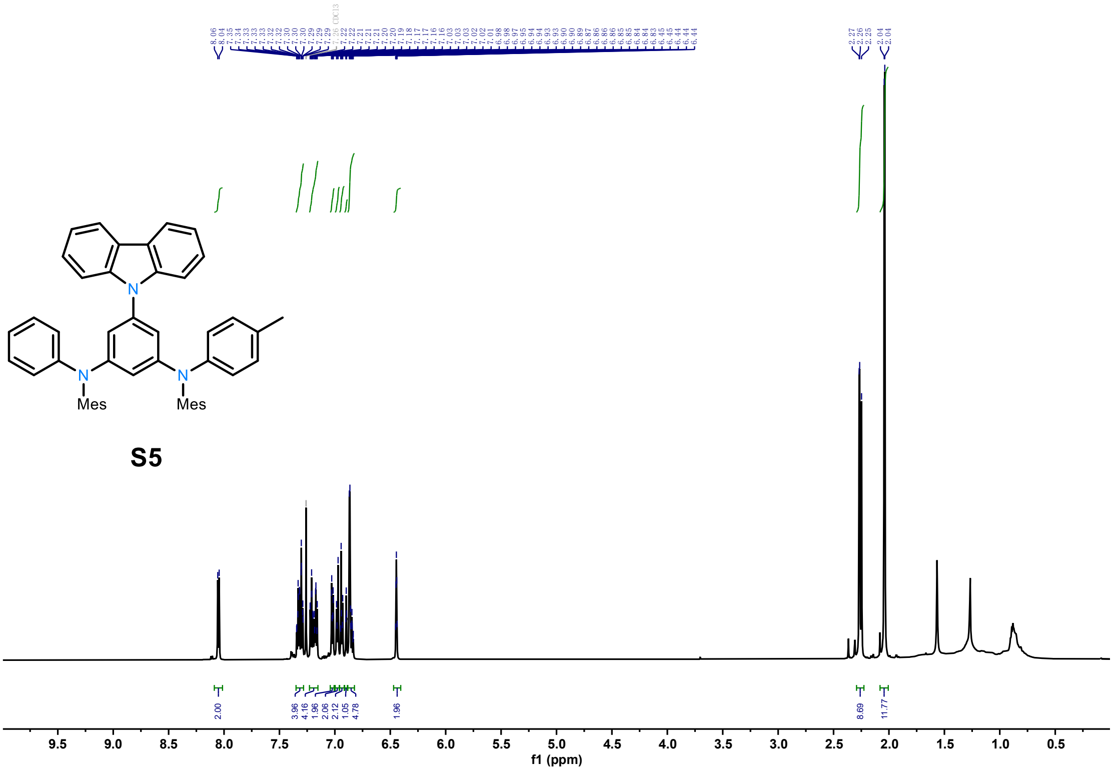

^{13}C NMR spectrum (151 MHz, $CDCl_3$, 298 K) of compound **S5**

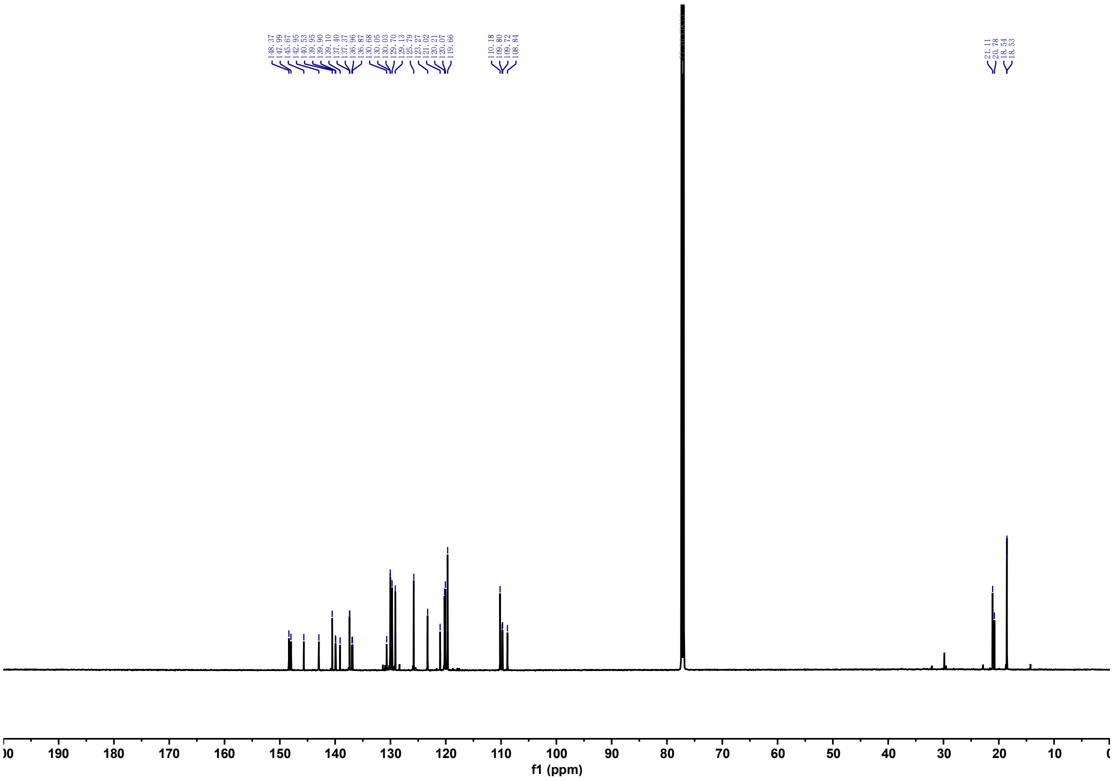

^{1}H NMR spectrum (400 MHz, $CDCl_3$, 298 K) of compound **S6**

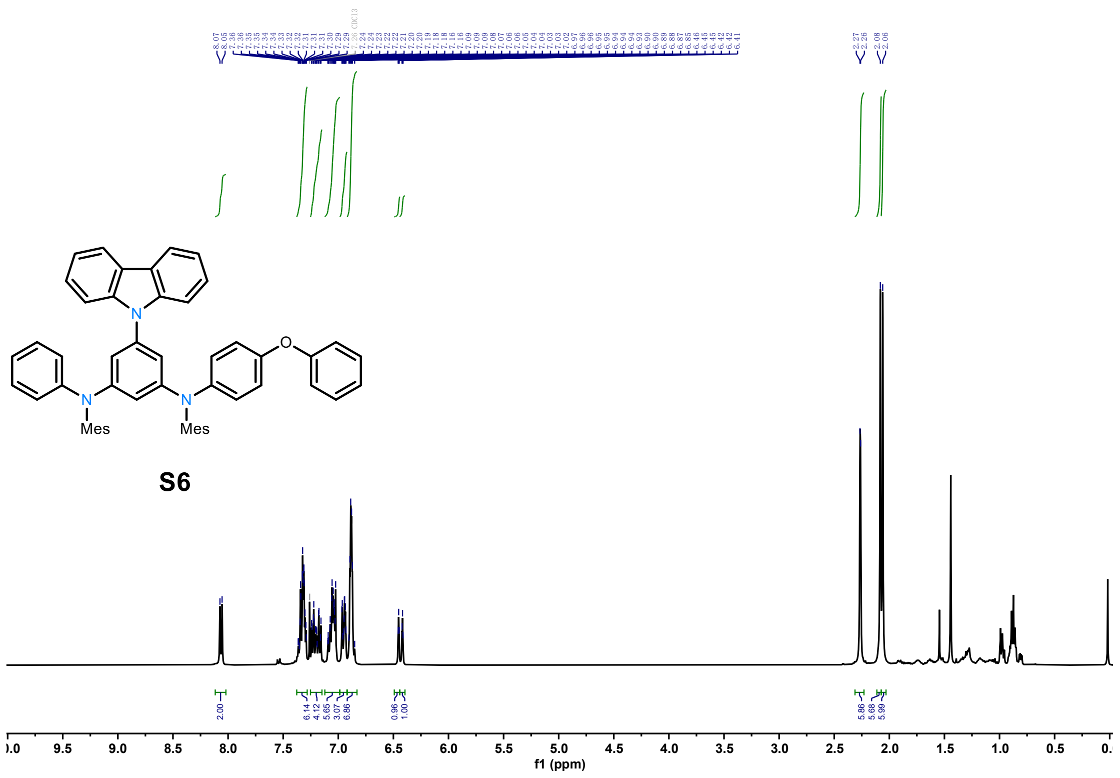

^{13}C NMR spectrum (101 MHz, $CDCl_3$, 298 K) of compound **S6**

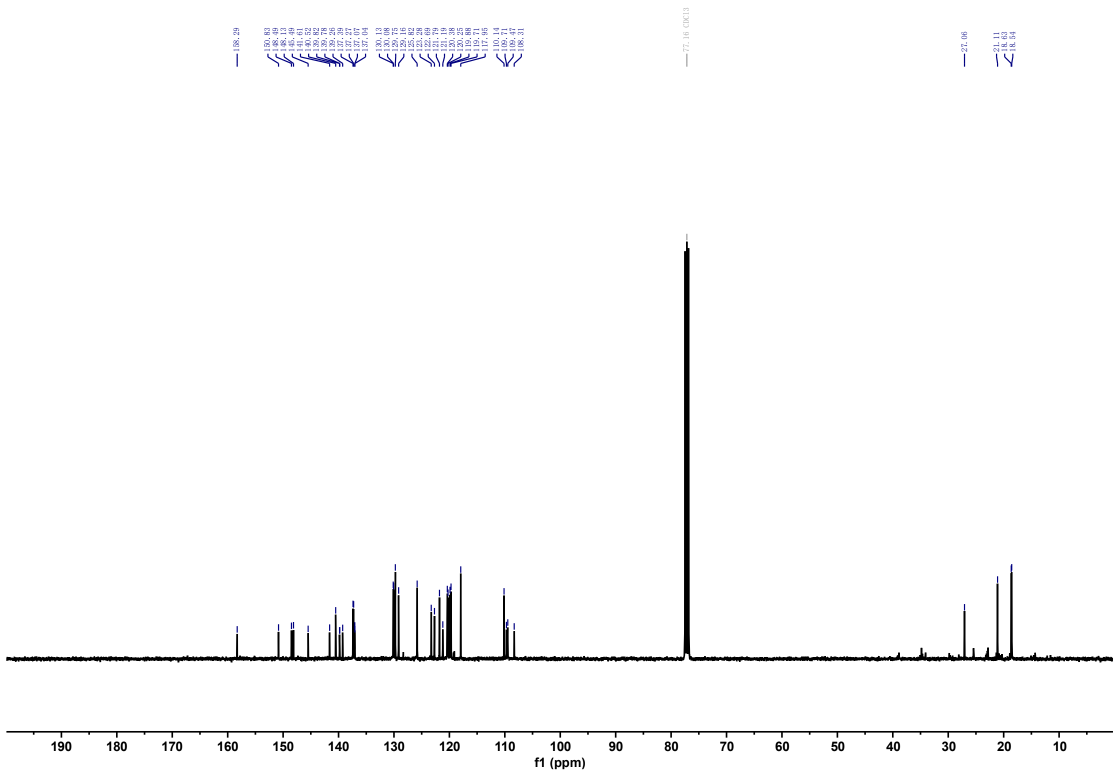

^{1}H NMR spectrum (600 MHz, $CDCl_3$, 298 K) of compound **S7**

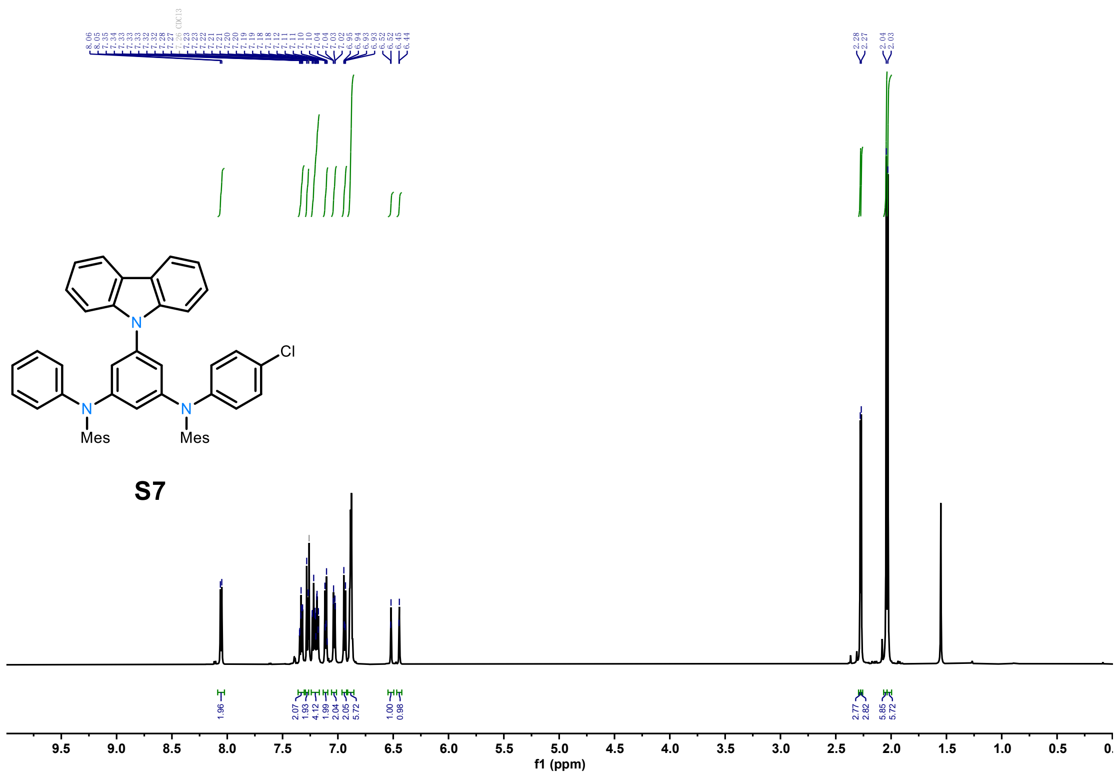

^{13}C NMR spectrum (151 MHz, $CDCl_3$, 298 K) of compound **S7**

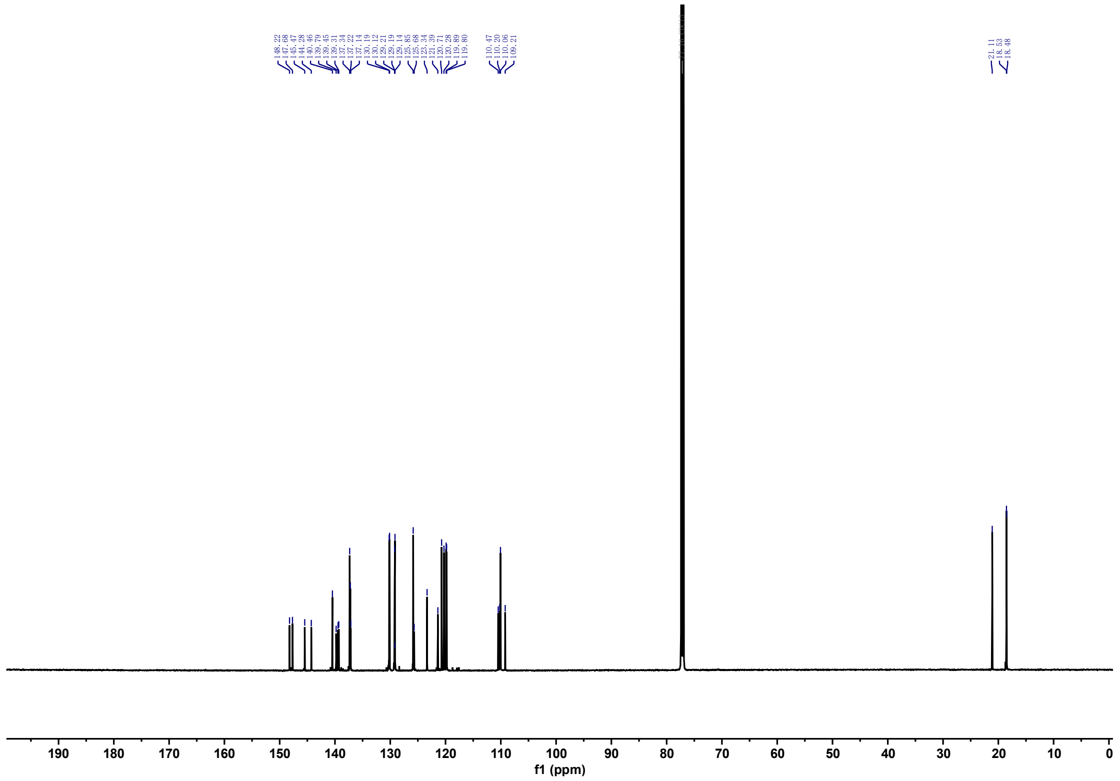

^{1}H NMR spectrum (600 MHz, $CDCl_3$, 298 K) of compound **S8**

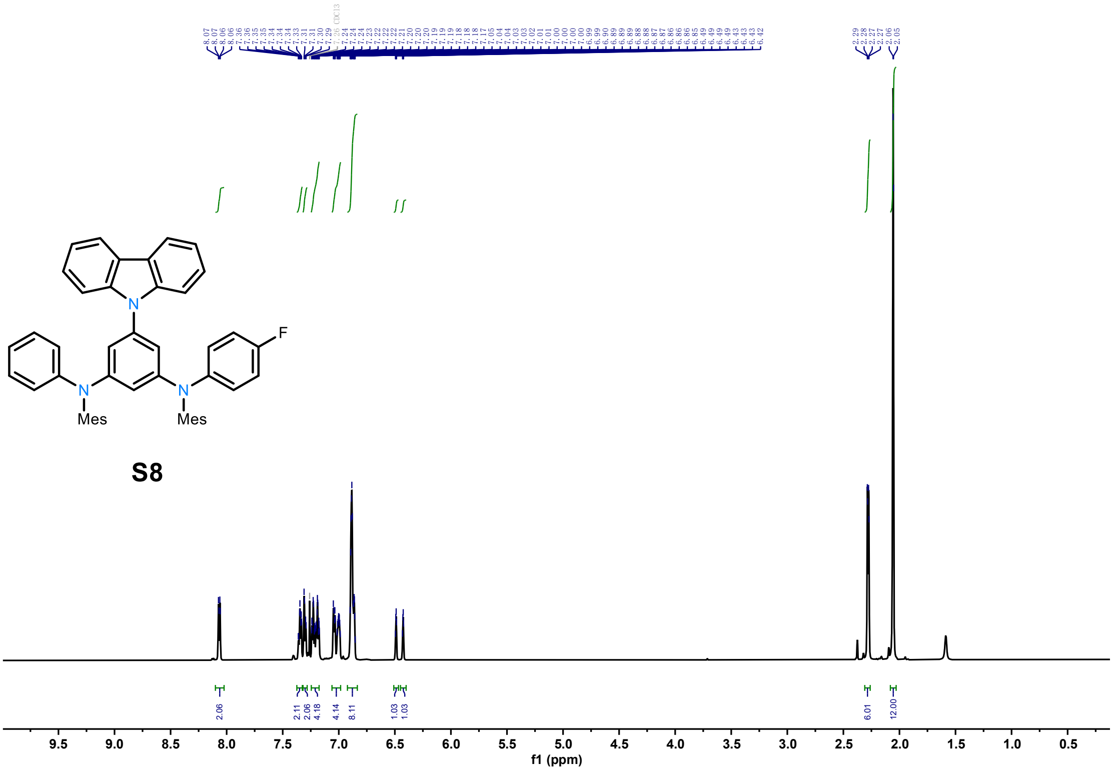

^{13}C NMR spectrum (151 MHz, $CDCl_3$, 298 K) of compound **S8**

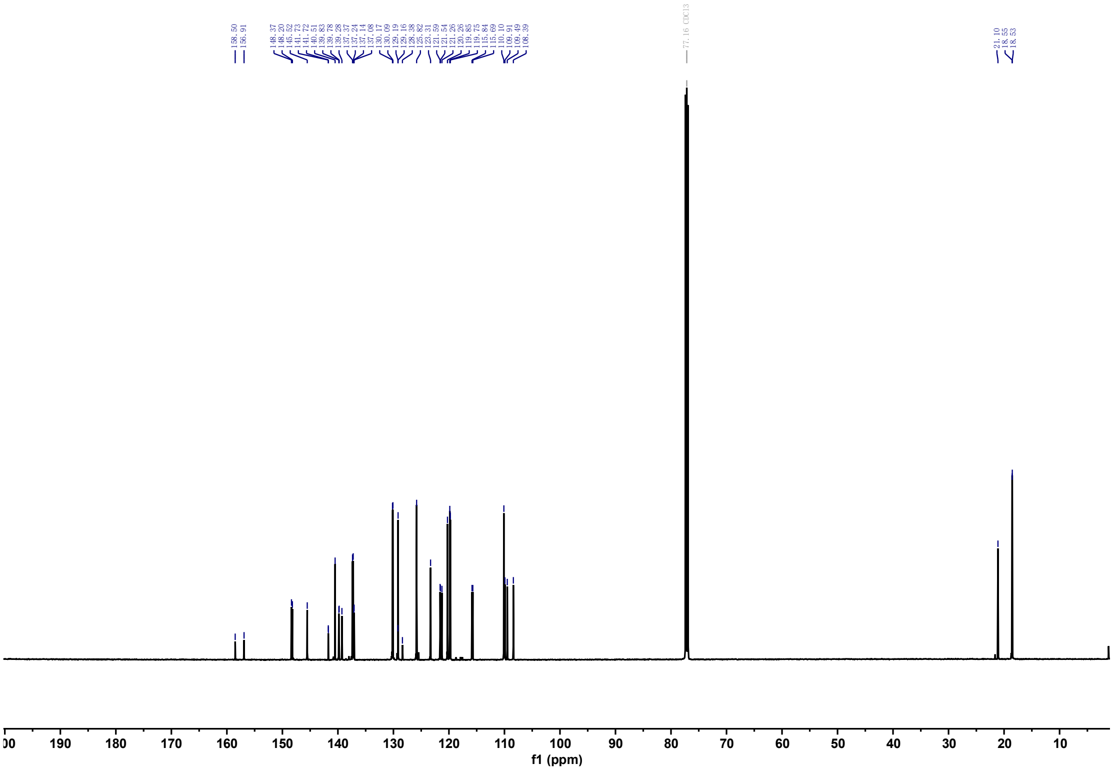

^{1}H NMR spectrum (600 MHz, Acetone-*d6*, 298 K) of compound **S9**

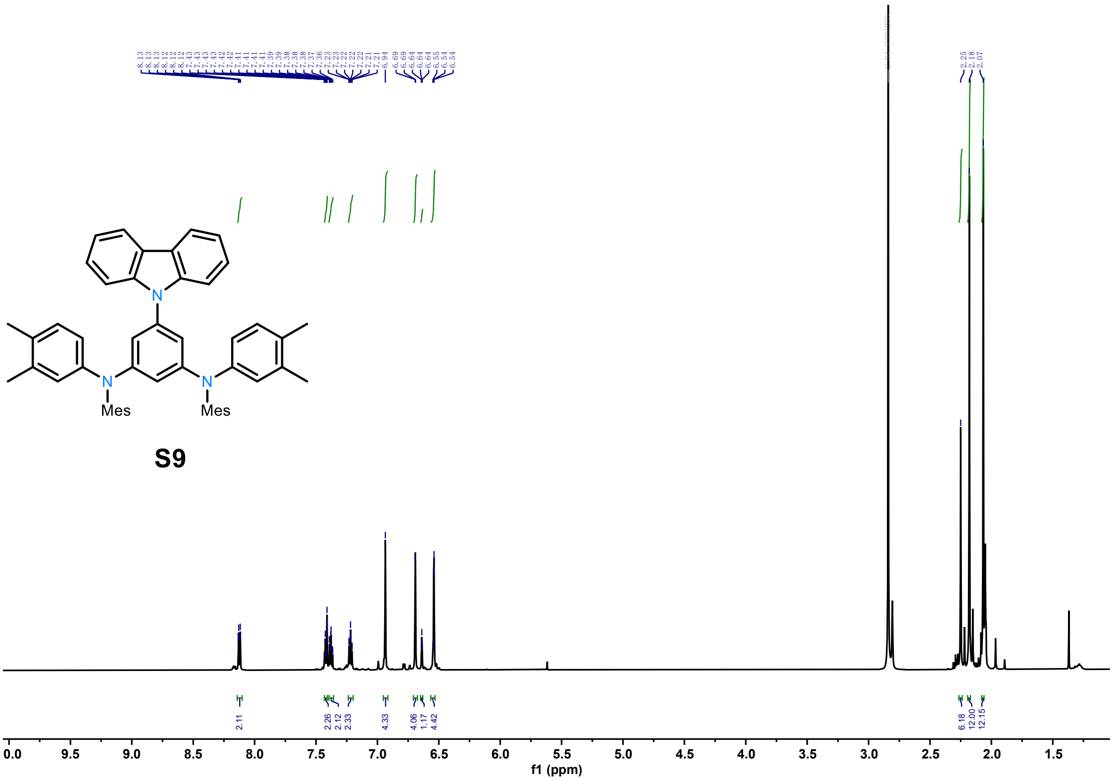

^{13}C NMR spectrum (151 MHz, Acetone-*d6*, 298 K) of compound **S9**

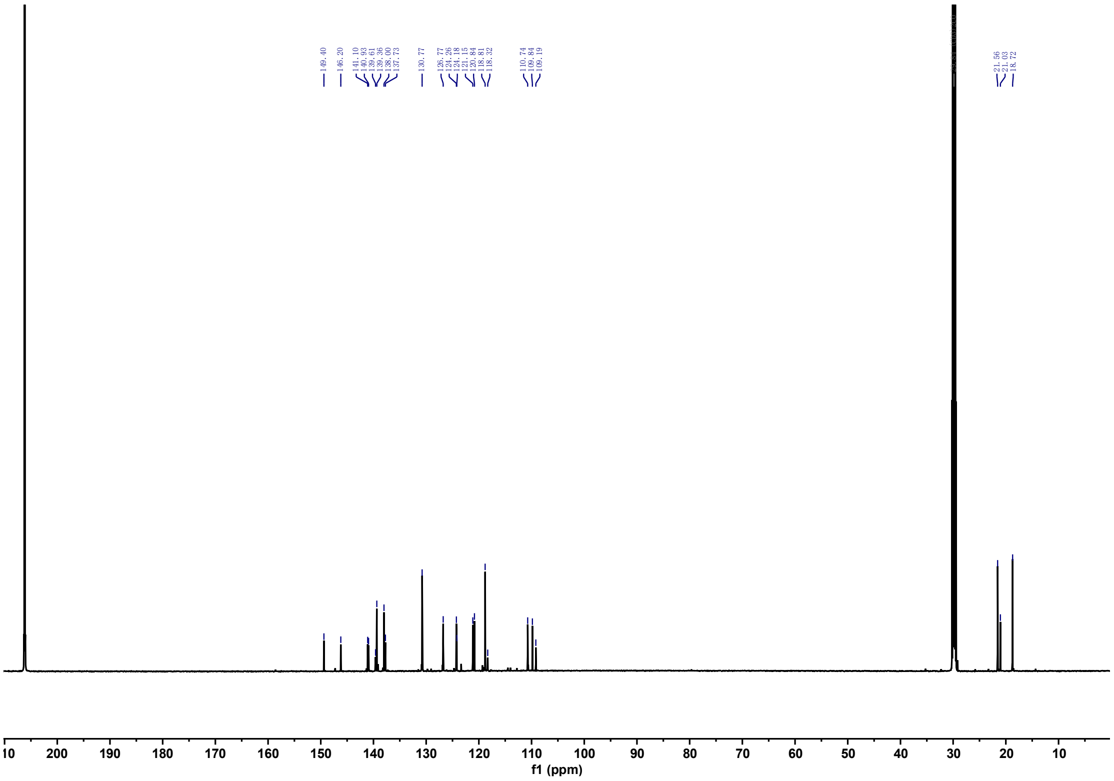

^{1}H NMR spectrum (400 MHz, $CDCl_3$, 298 K) of compound **S10**

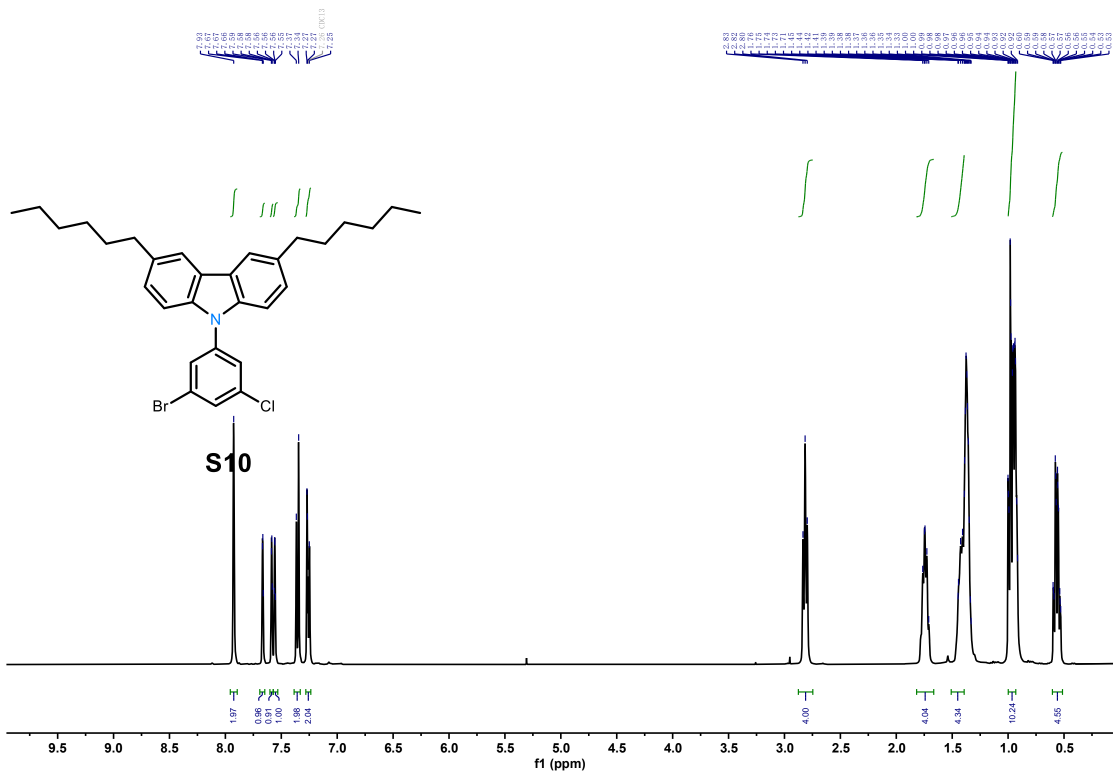

^{13}C NMR spectrum (101 MHz, $CDCl_3$, 298 K) of compound **S10**

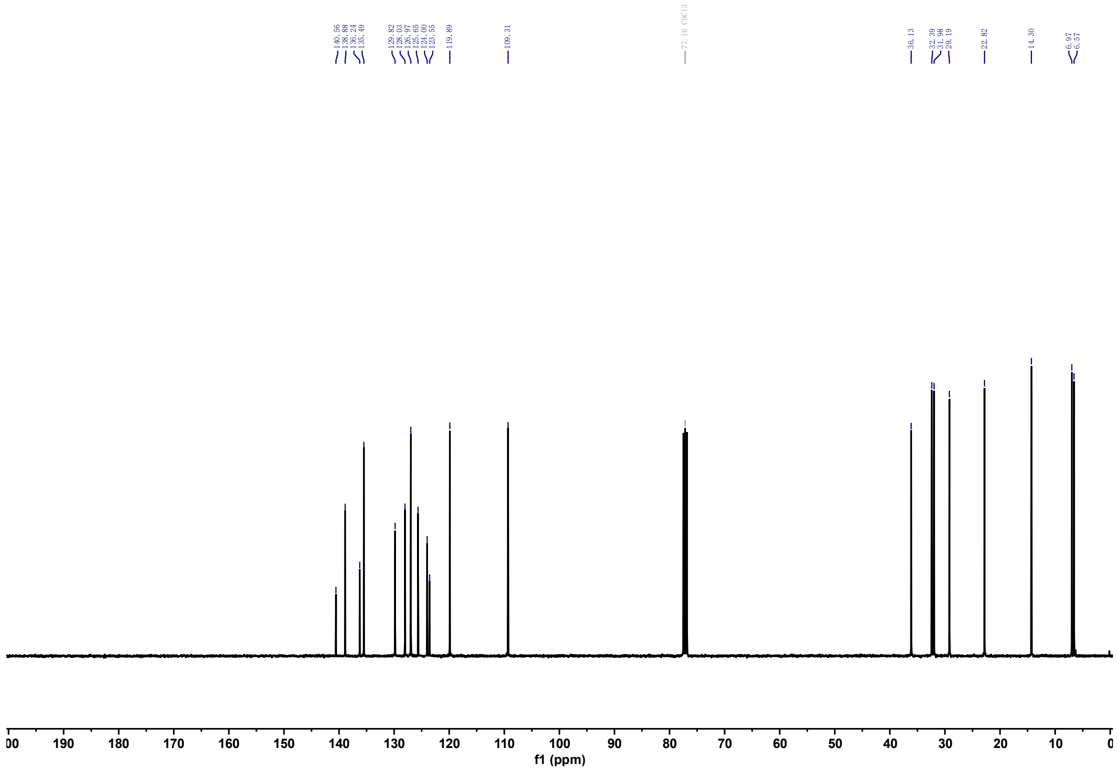

^{1}H NMR spectrum (400 MHz, $CDCl_3$, 298 K) of compound **S11**

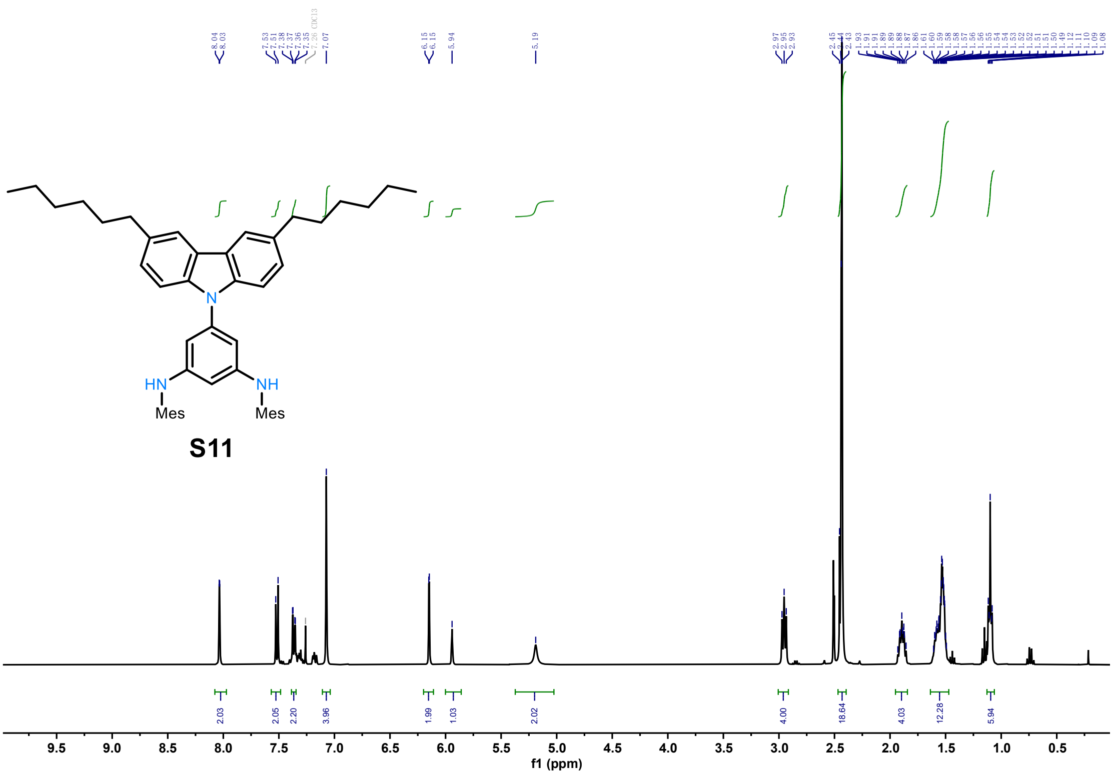

^{13}C NMR spectrum (101 MHz, $CDCl_3$, 298 K) of compound **S11**

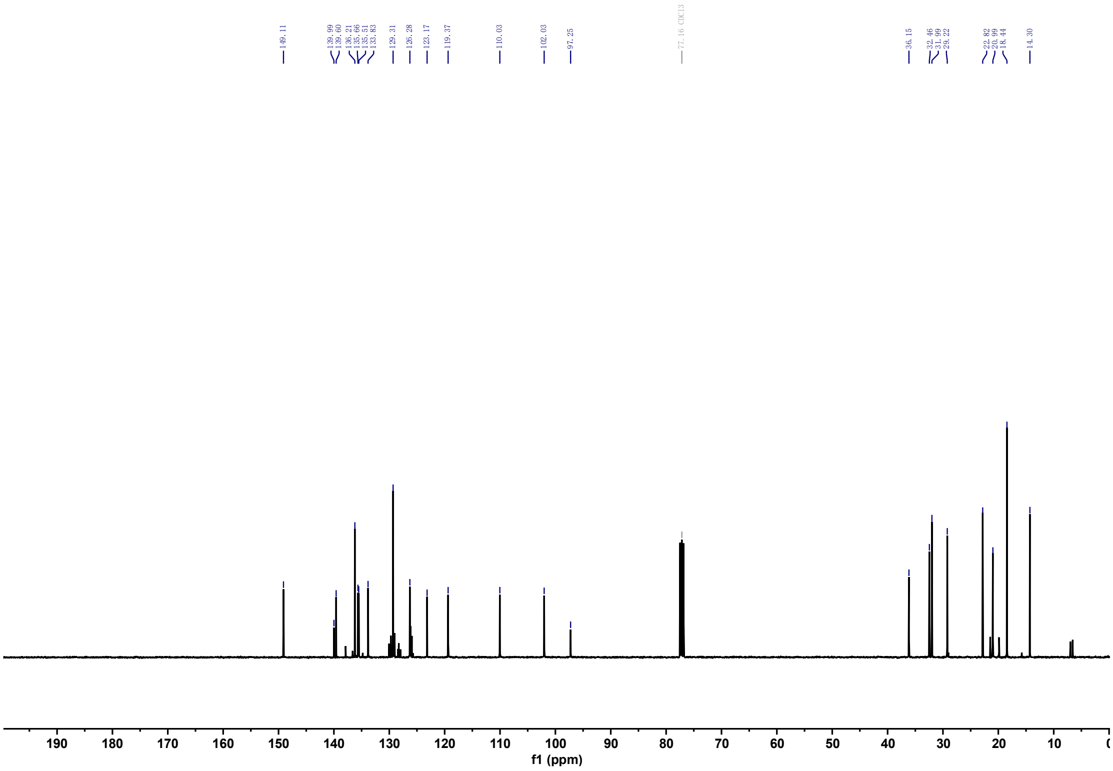

^{1}H NMR spectrum (400 MHz, $CDCl_3$, 298 K) of compound **S12**

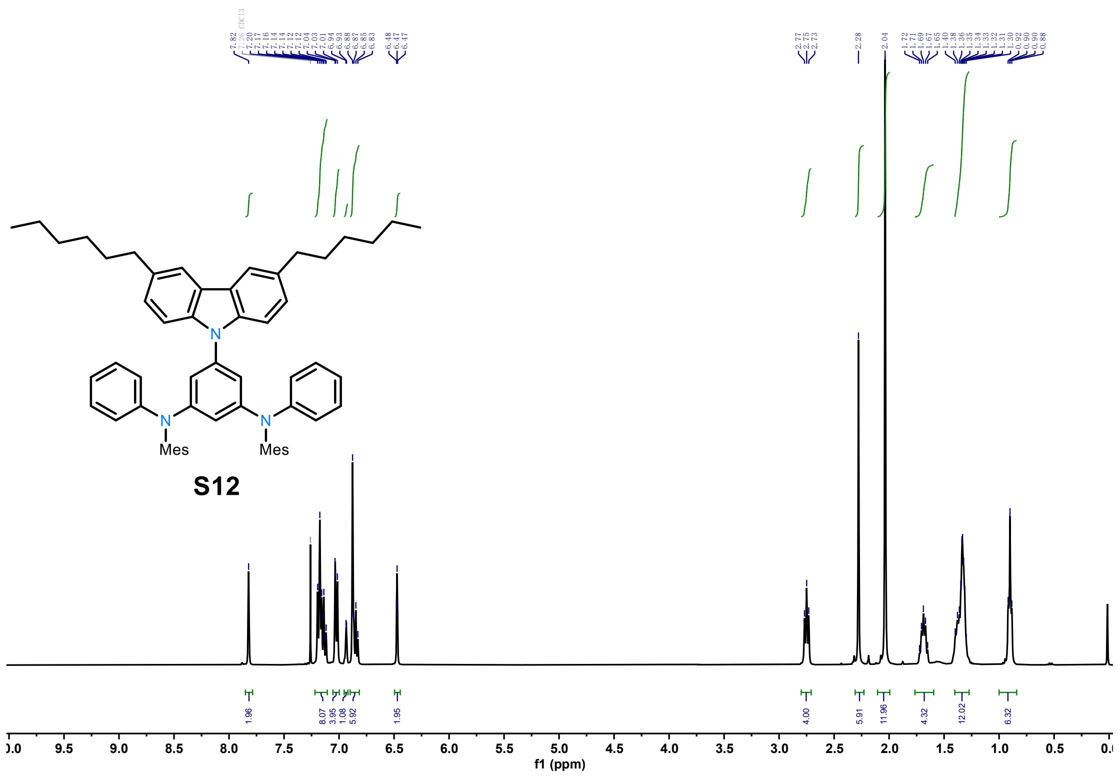

^{13}C NMR spectrum (101 MHz, $CDCl_3$, 298 K) of compound **S12**

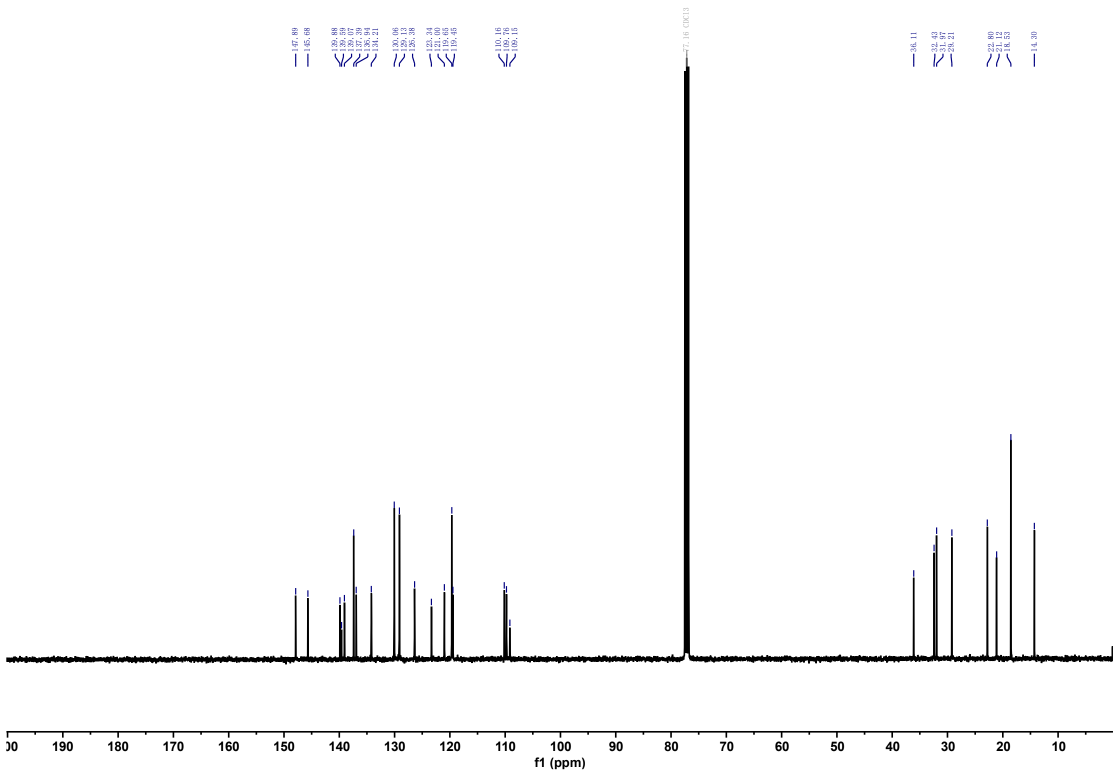

^{1}H NMR spectrum (600 MHz, $CDCl_3$, 298 K) of compound **S13**

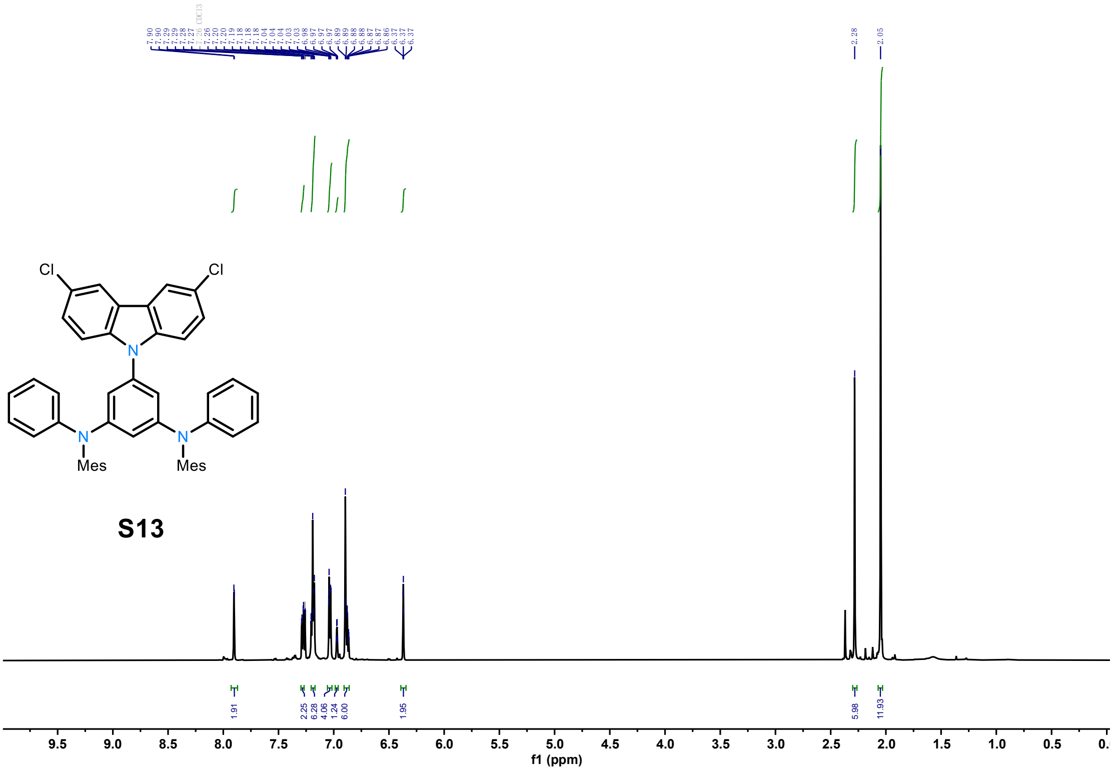

^{13}C NMR spectrum (151 MHz, $CDCl_3$, 298 K) of compound **S13**

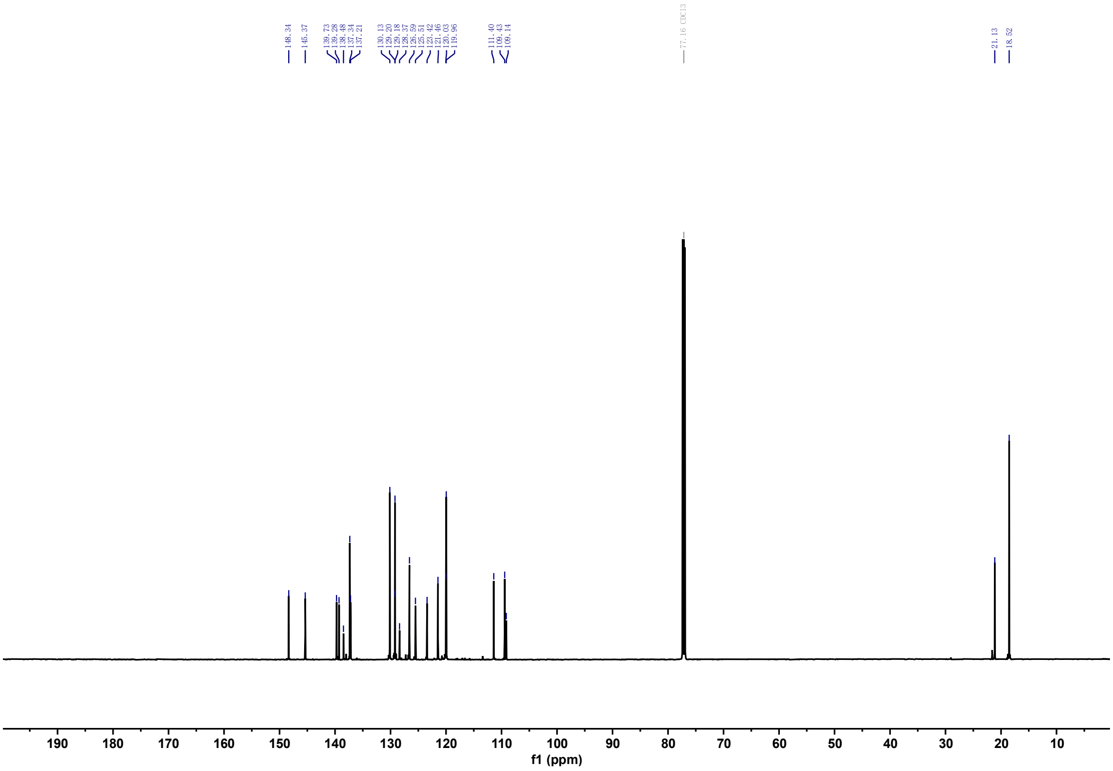

^{1}H NMR spectrum (400 MHz, $CDCl_3$, 298 K) of compound **S14**

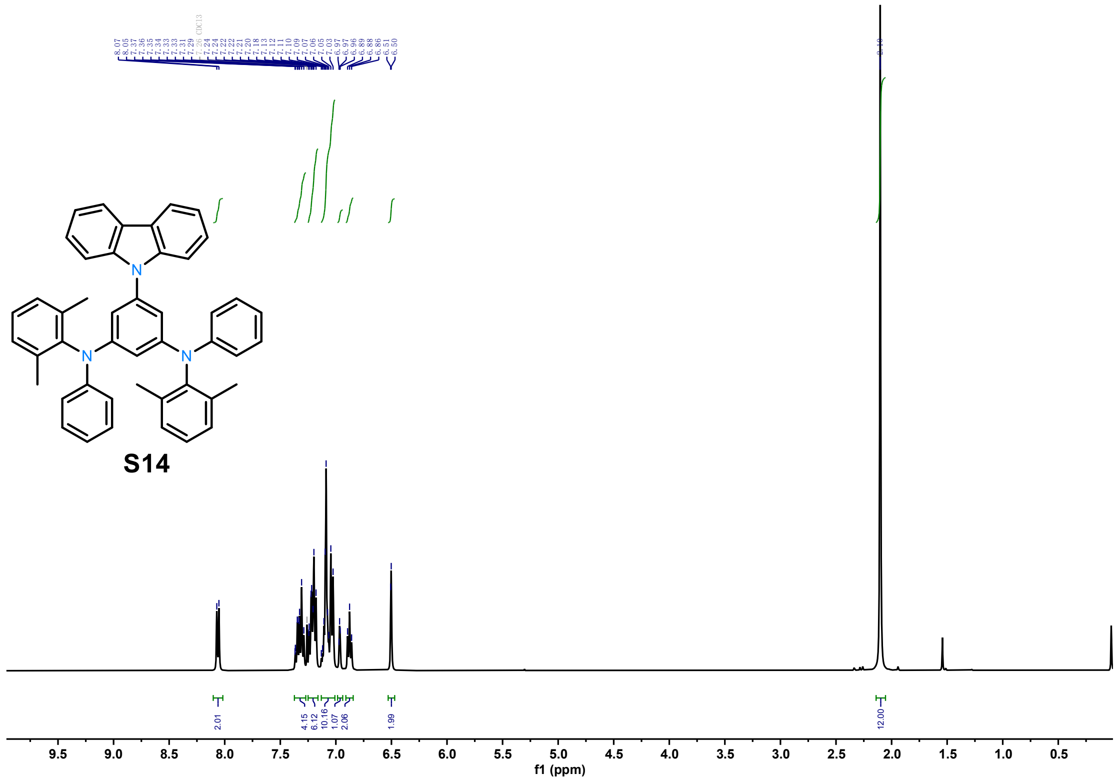

^{13}C NMR spectrum (101 MHz, $CDCl_3$, 298 K) of compound **S14**

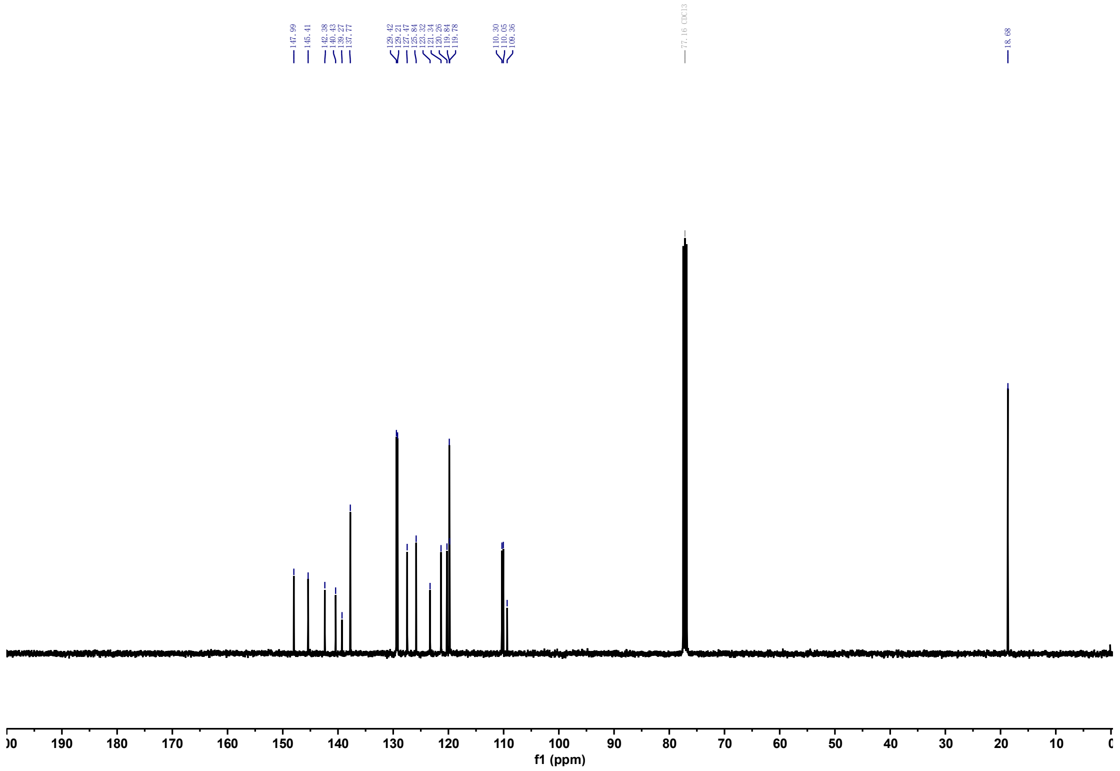

^{1}H NMR spectrum (600 MHz, $CDCl_3$, 298 K) of compound **S15**

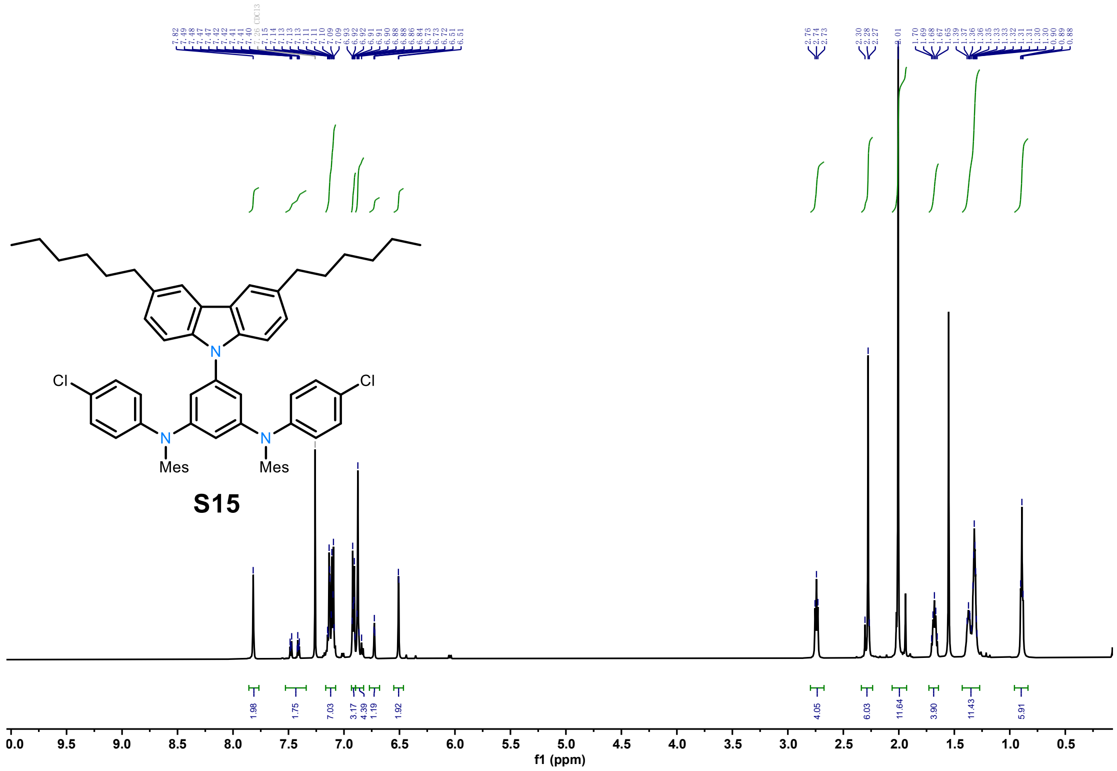

^{13}C NMR spectrum (151 MHz, $CDCl_3$, 298 K) of compound **S15**

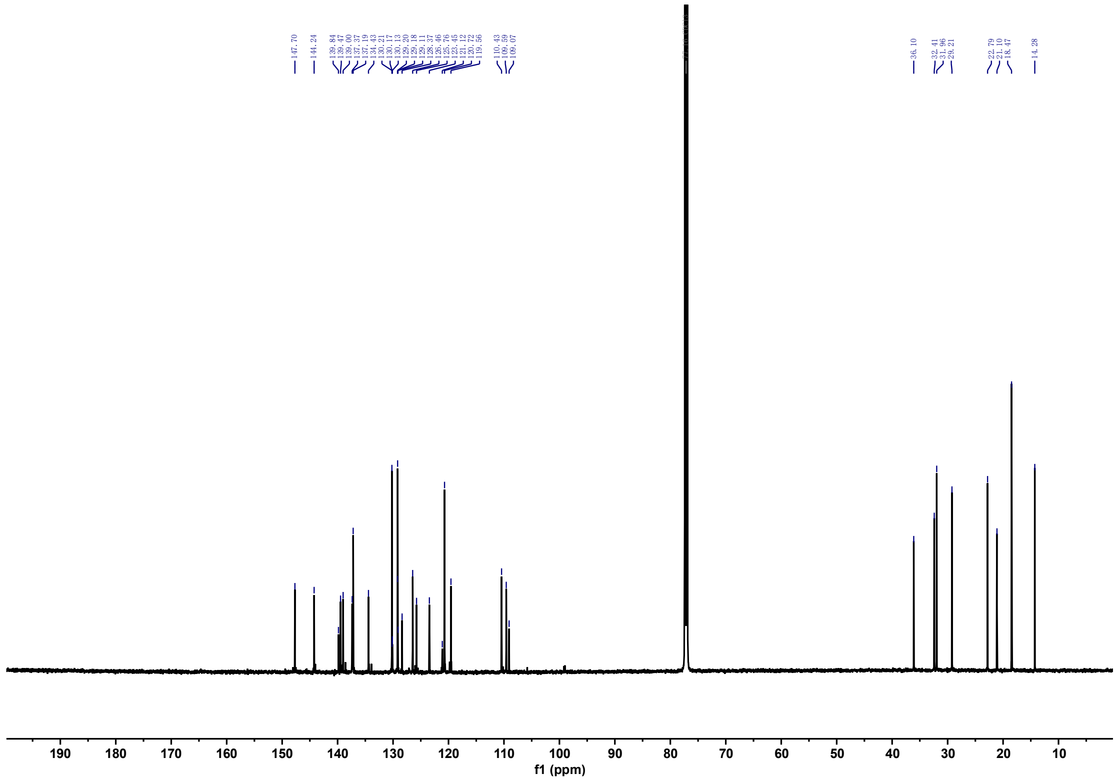

^{1}H NMR spectrum (400 MHz, DMSO-*d6*, 298 K) of compound **S16**

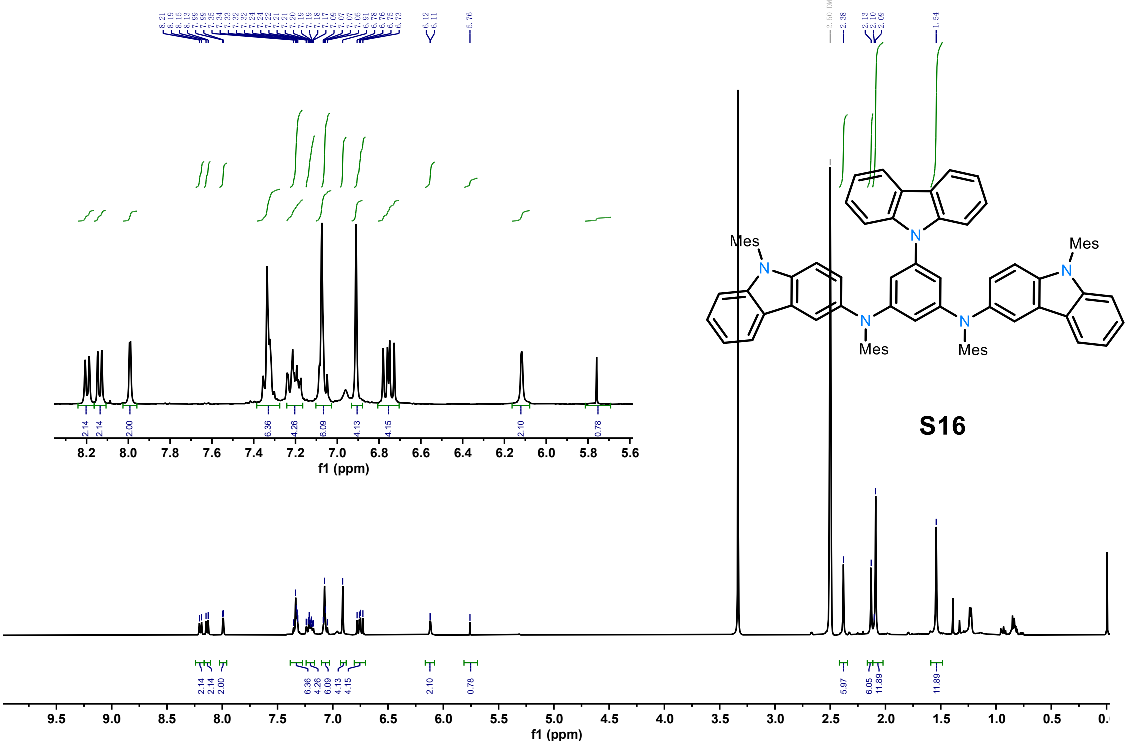

^{13}C NMR spectrum (151 MHz, Acetone-*d6*, 298 K) of compound **S16**

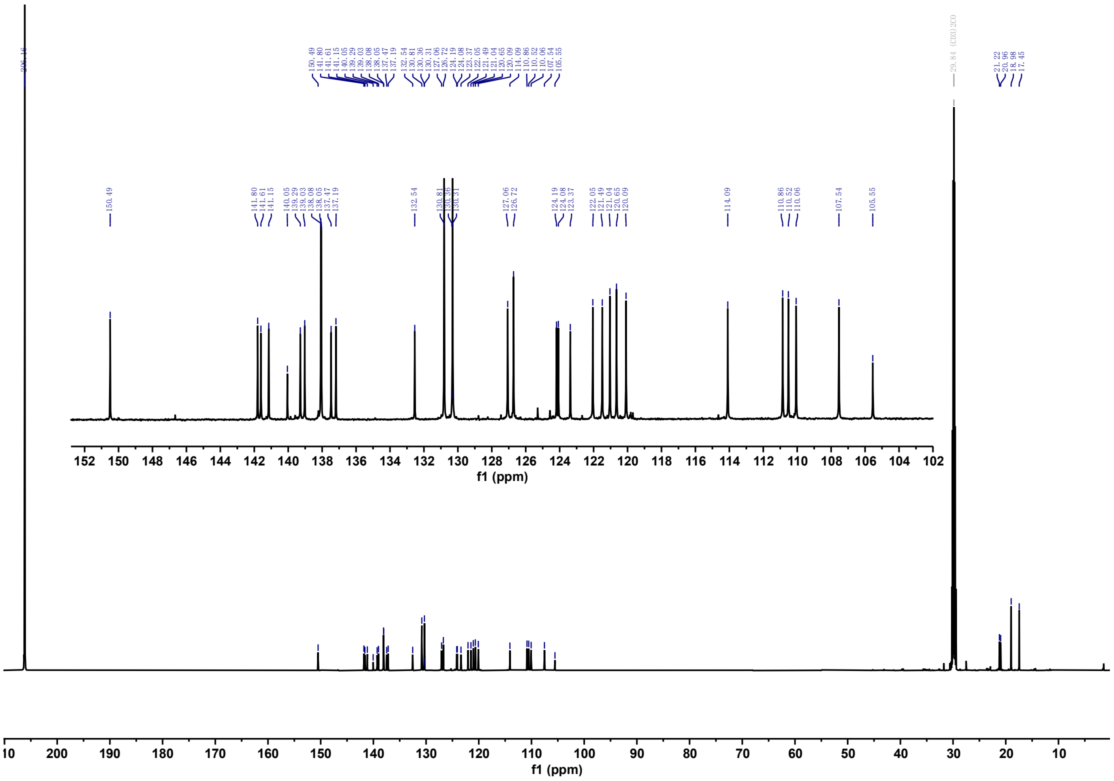

^{1}H NMR spectrum (400 MHz, $CDCl_3$, 298 K) of compound **S17**

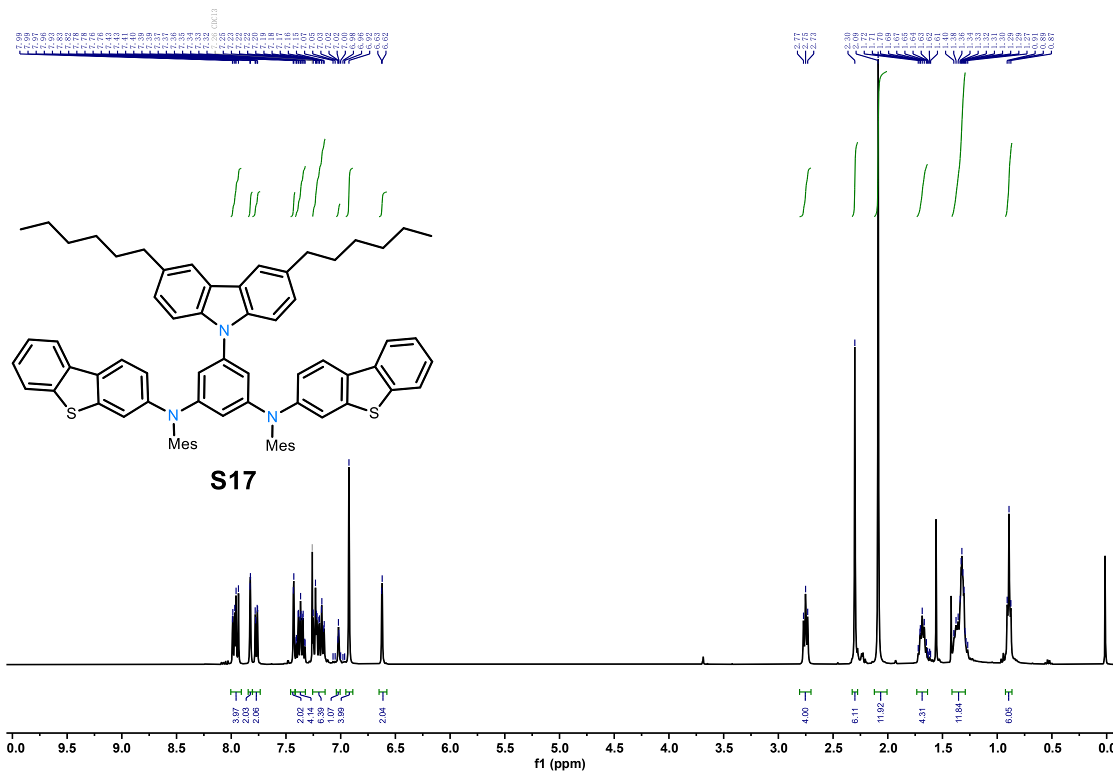

^{13}C NMR spectrum (101 MHz, $CDCl_3$, 298 K) of compound **S17**

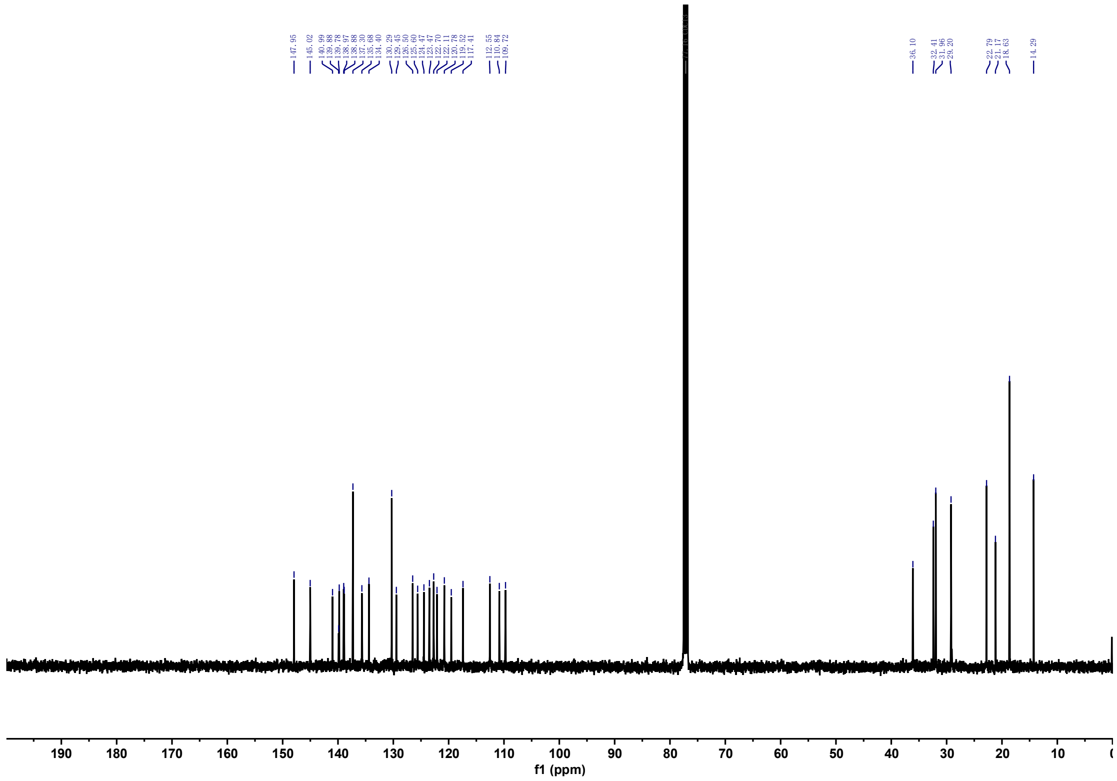

^{1}H NMR spectrum (400 MHz, $CDCl_3$, 298 K) of compound **S18**

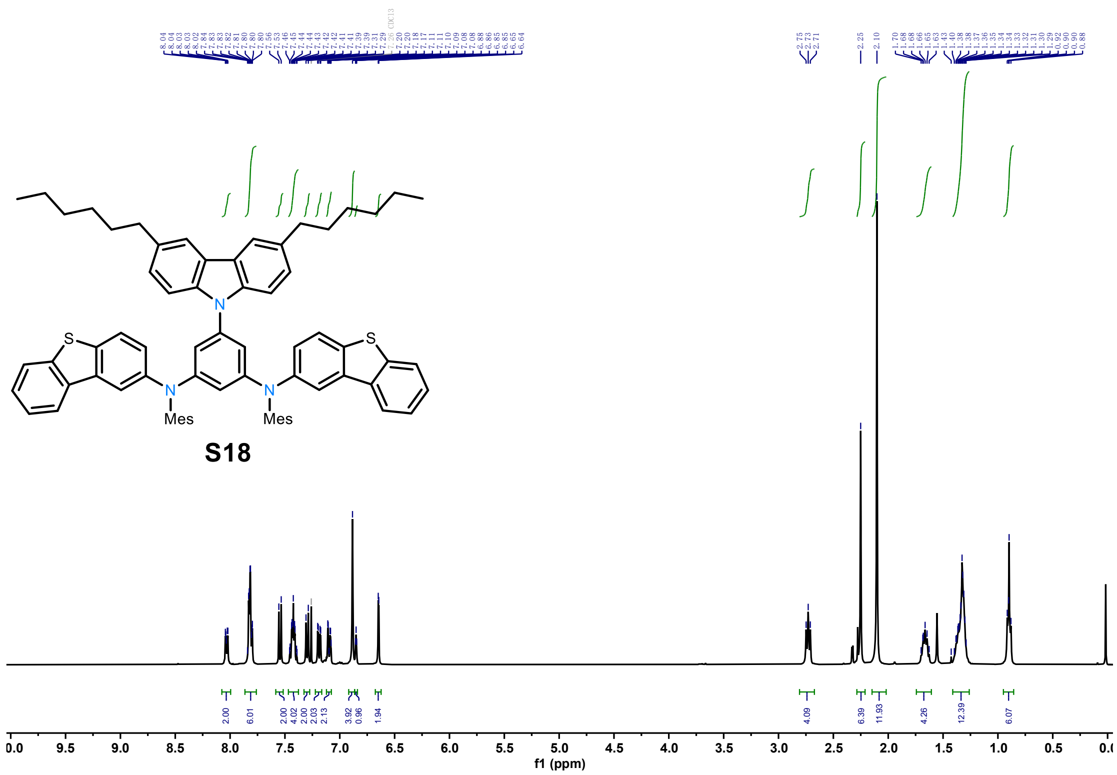

^{13}C NMR spectrum (101 MHz, $CDCl_3$, 298 K) of compound **S18**

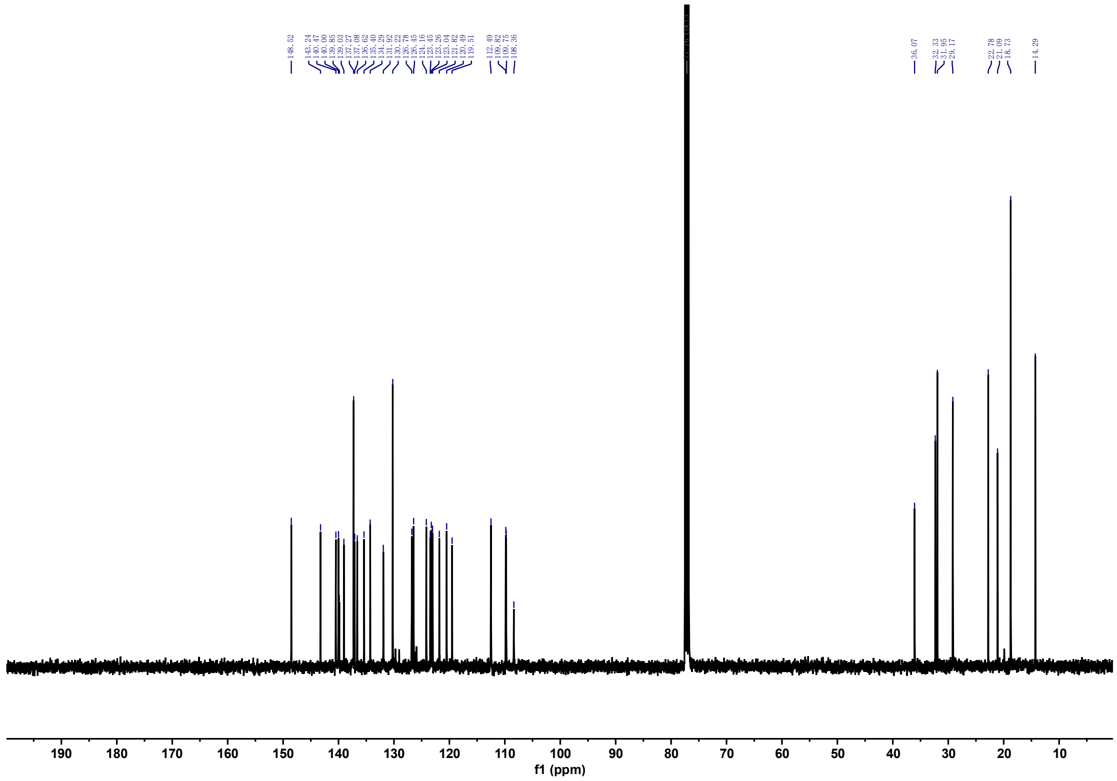

^{1}H NMR spectrum (600 MHz, $CDCl_3$, 298 K) of compound **S19**

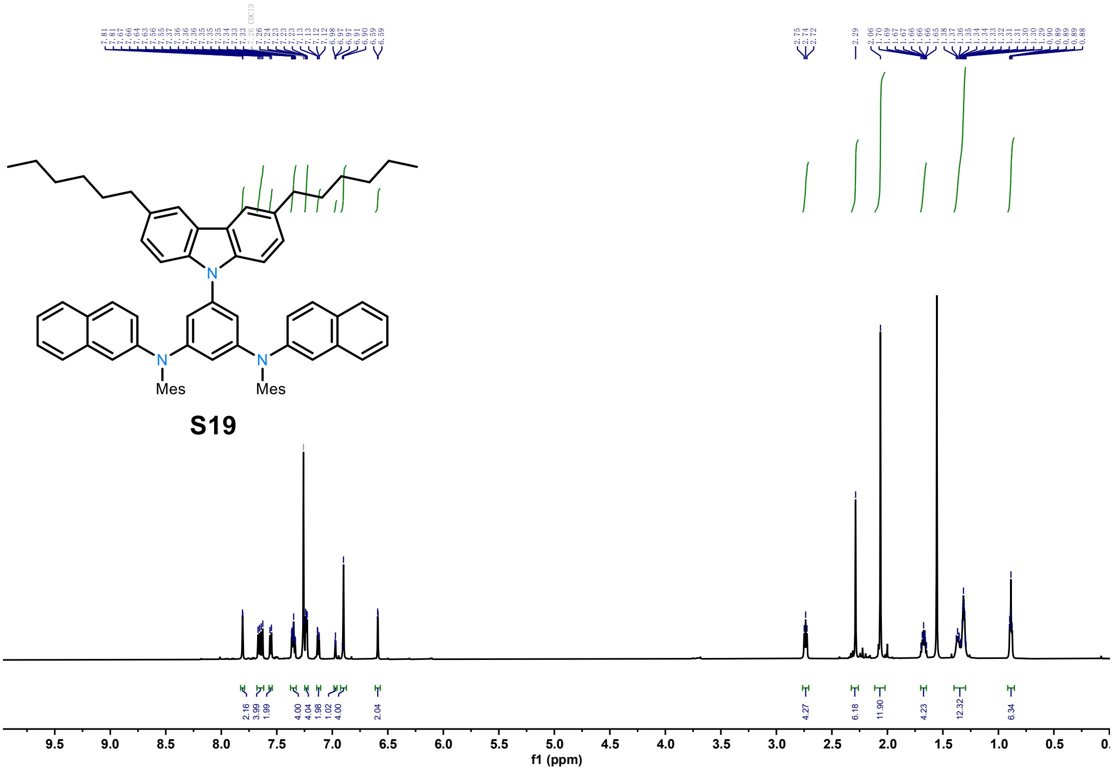

^{13}C NMR spectrum (151 MHz, $CDCl_3$, 298 K) of compound **S19**

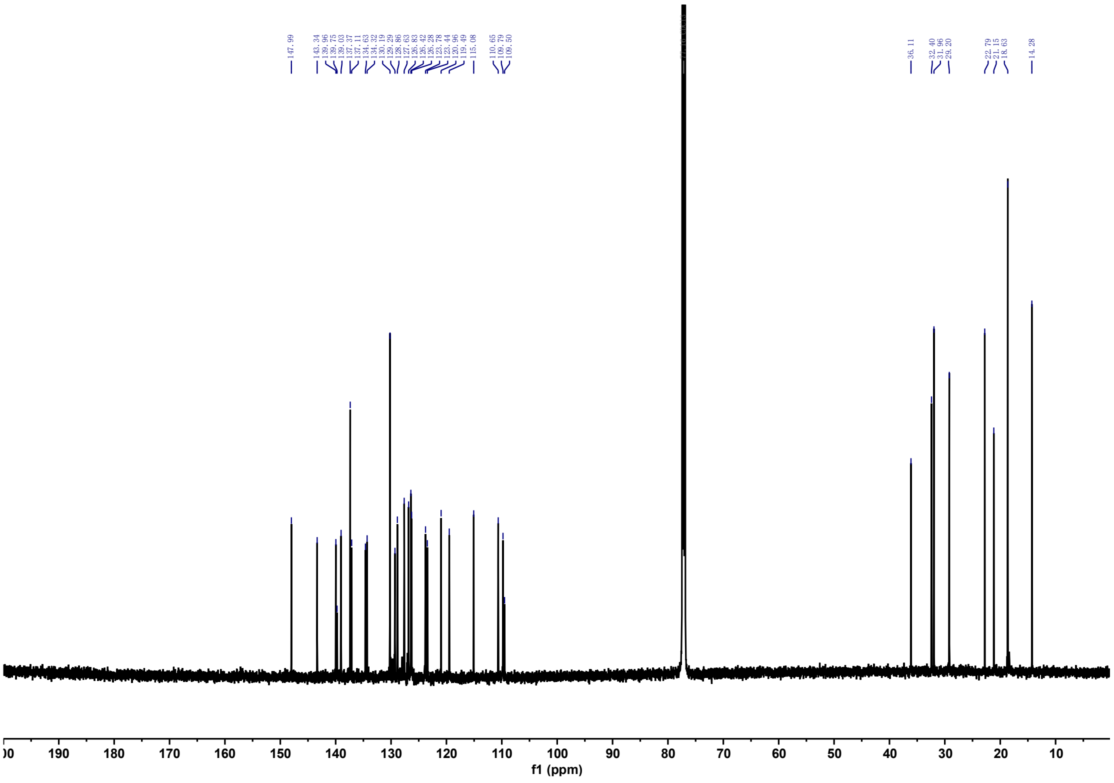

^{1}H NMR spectrum (600 MHz, $CDCl_3$, 298 K) of compound **S20**

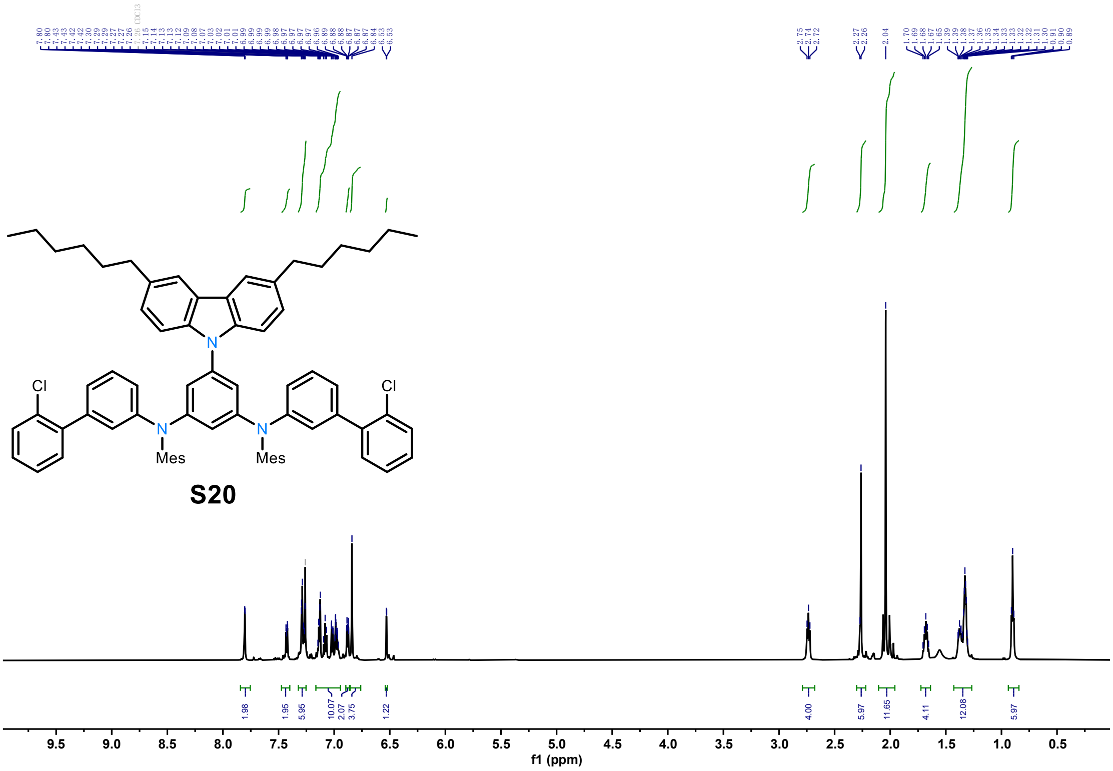

^{13}C NMR spectrum (151 MHz, $CDCl_3$, 298 K) of compound **S20**

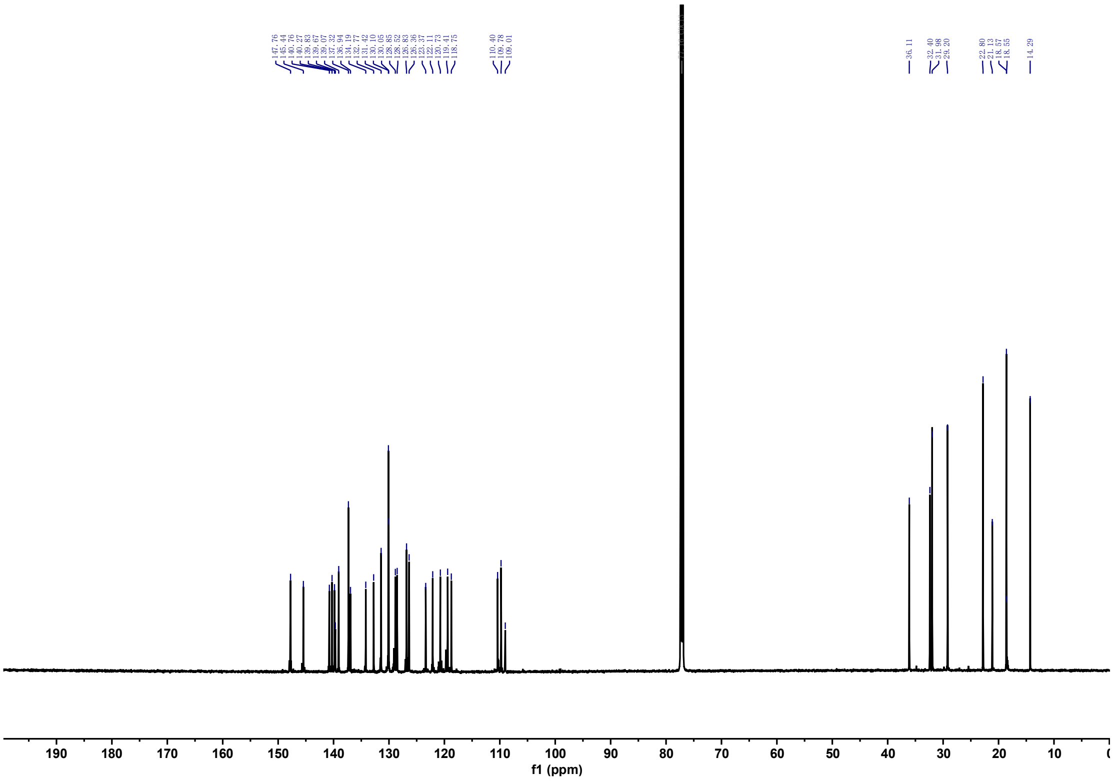

^{1}H NMR spectrum (400 MHz, $CDCl_3$, 298 K) of compound **S21**

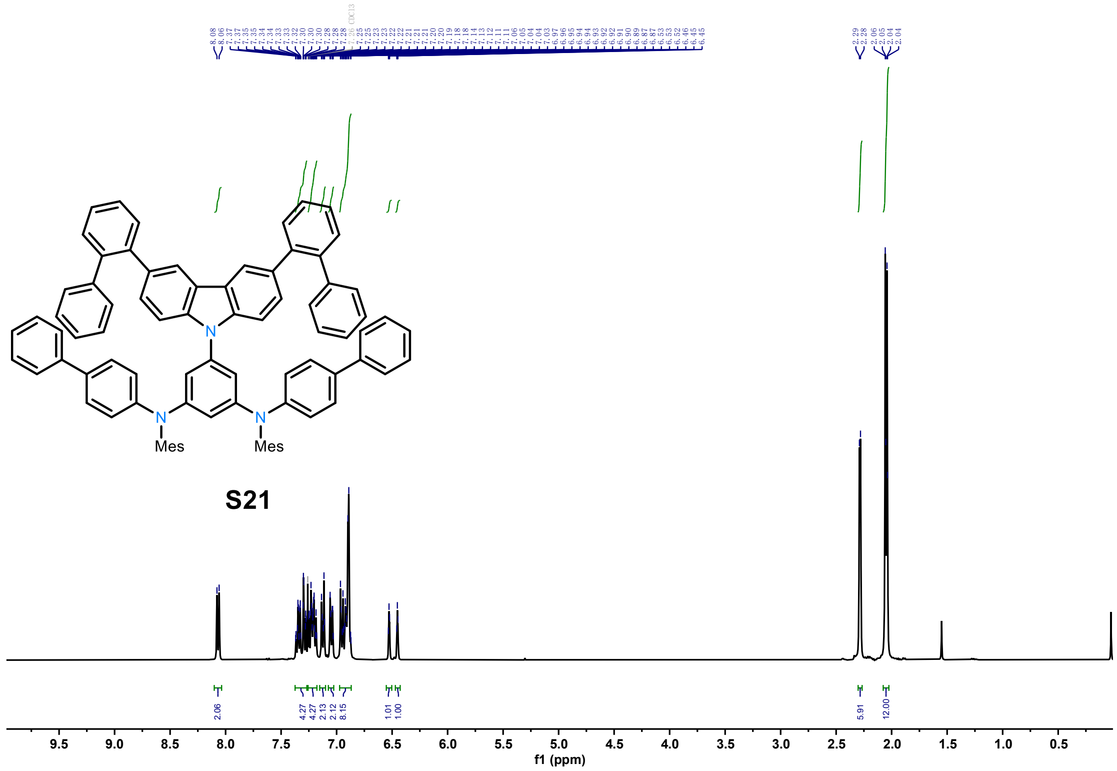

^{13}C NMR spectrum (101 MHz, $CDCl_3$, 298 K) of compound **S21**

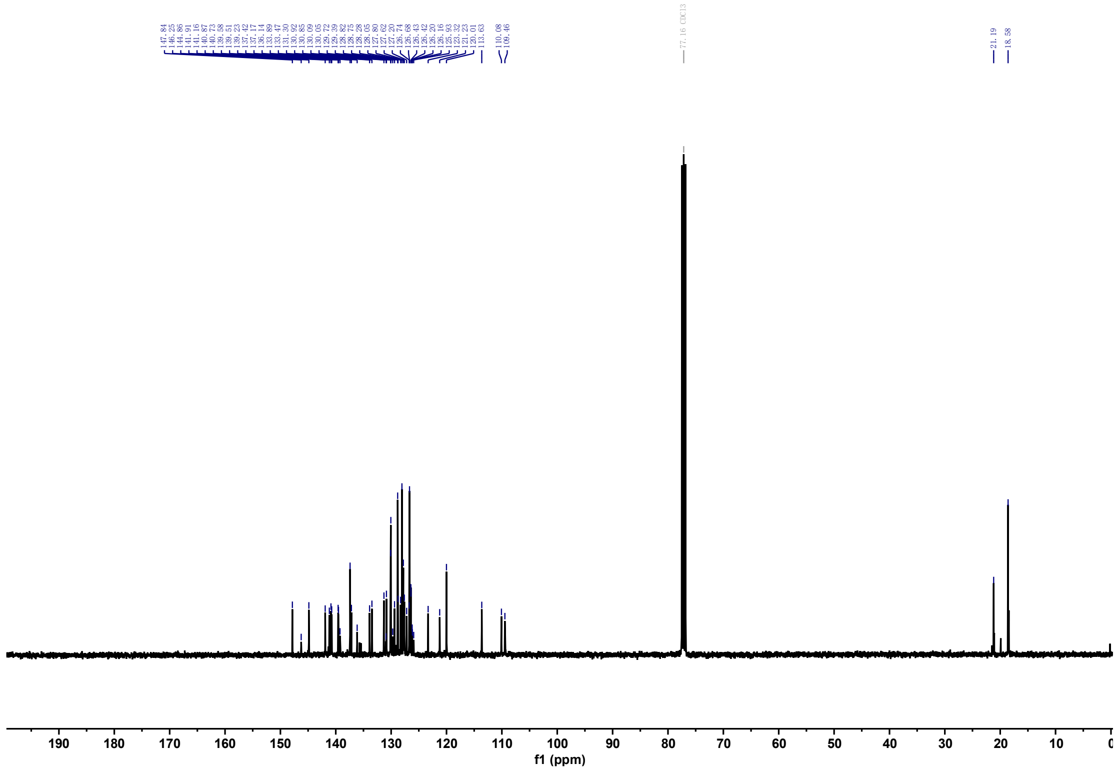

^{1}H NMR spectrum (400 MHz, $CDCl_3$, 298 K) of compound **S22**

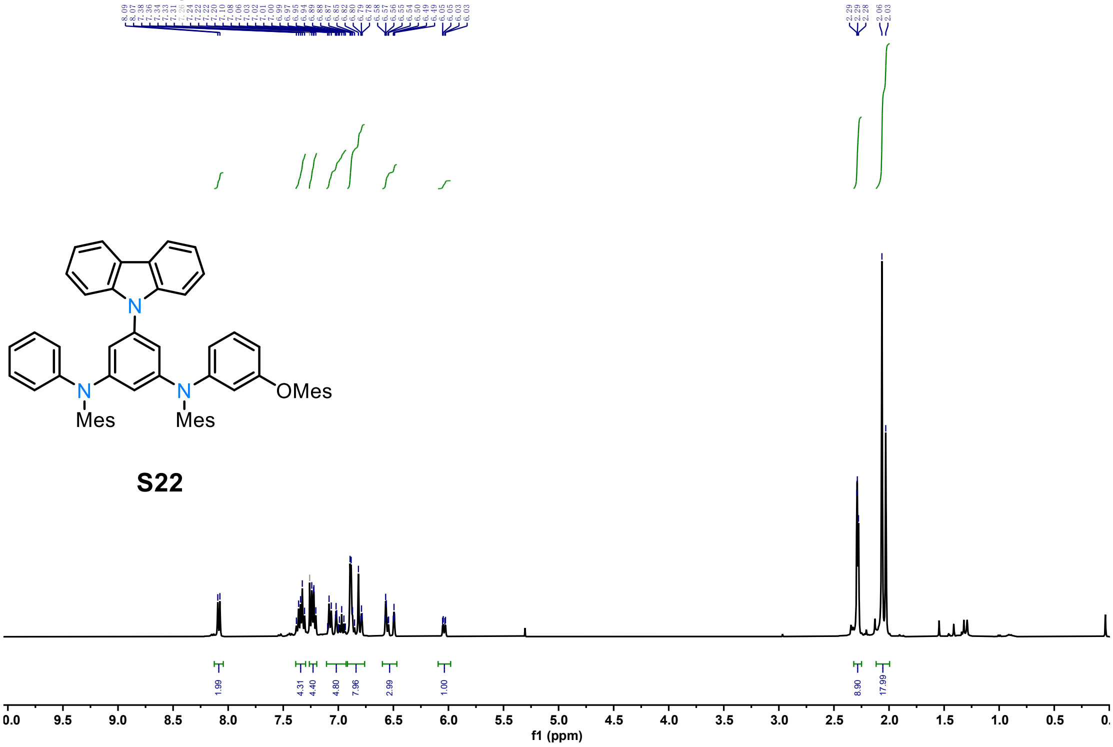

S22

^{13}C NMR spectrum (101 MHz, $CDCl_3$, 298 K) of compound **S22**

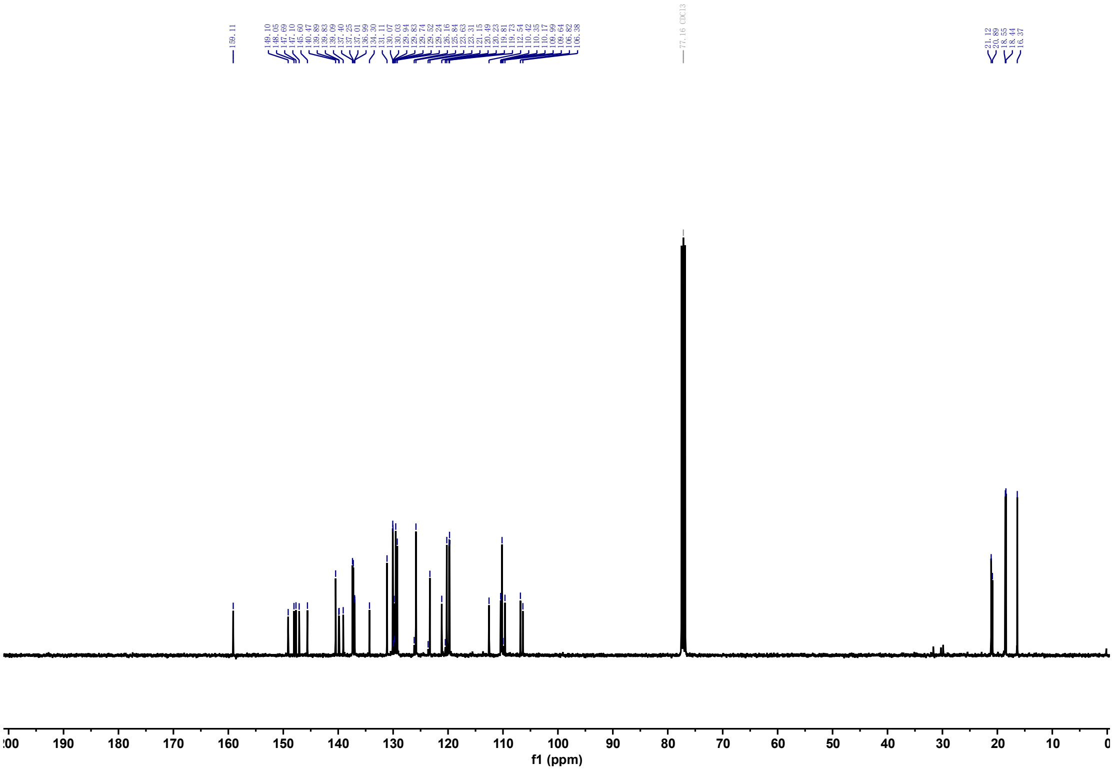

12. Mass spectrum

HR-MS (MALDI-TOF) spectrum of compound **1**

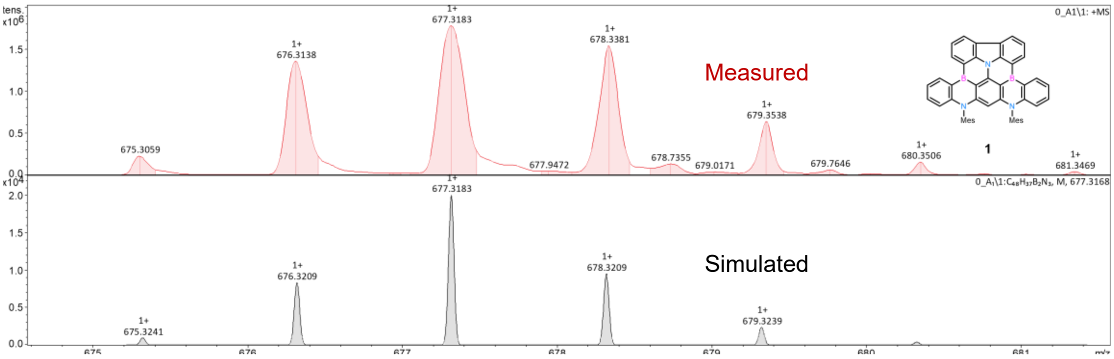

HR-MS (MALDI-TOF) spectrum of compound **2**

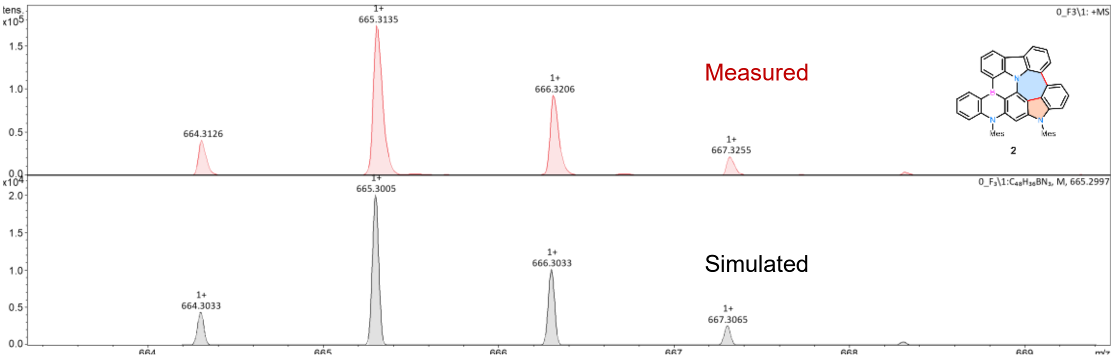

HR-MS (MALDI-TOF) spectrum of compound **3a**

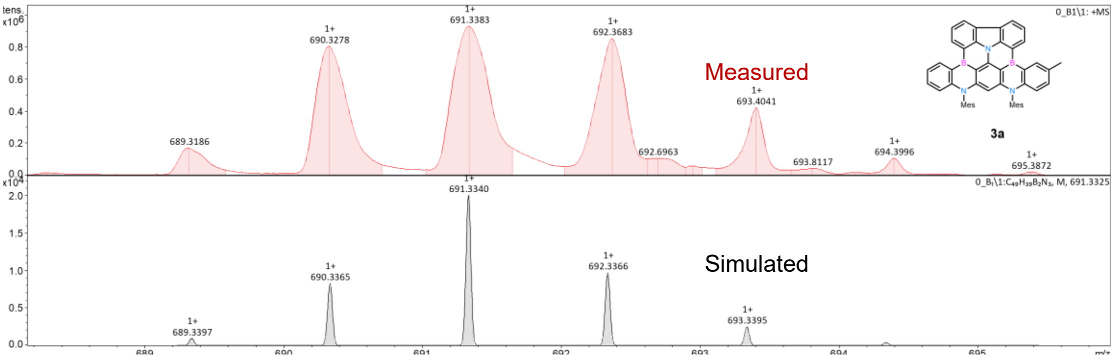

HR-MS (MALDI-TOF) spectrum of compound **3b**

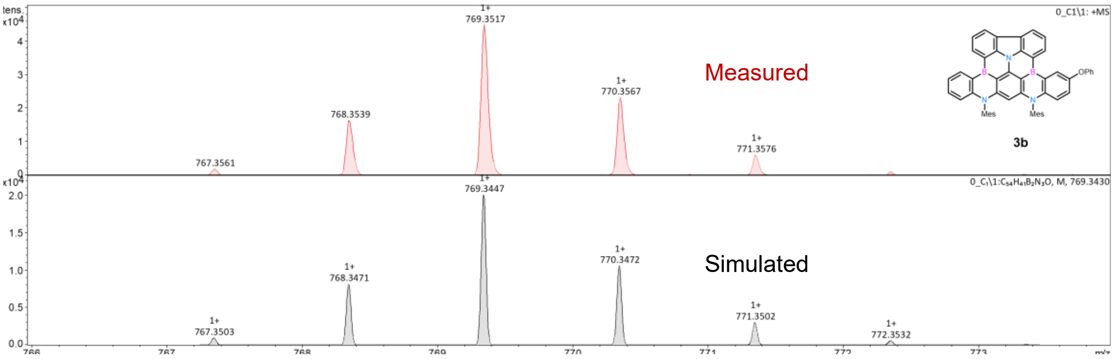

HR-MS (MALDI-TOF) spectrum of compound **3b’**

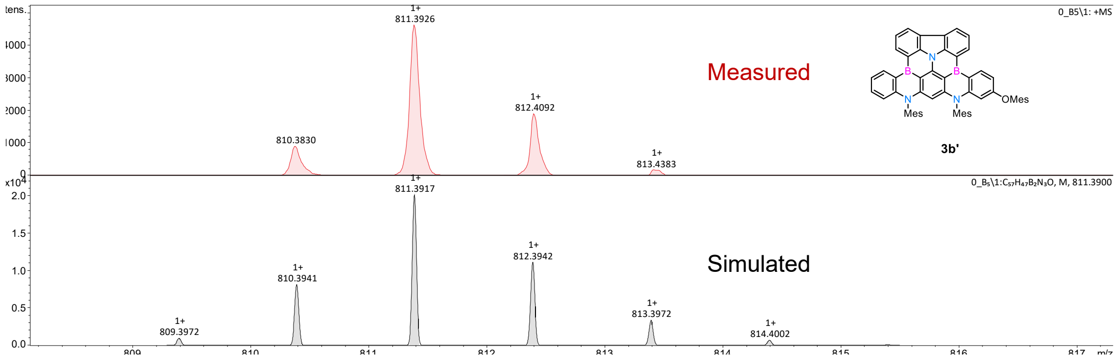

HR-MS (MALDI-TOF) spectrum of compound **3c**

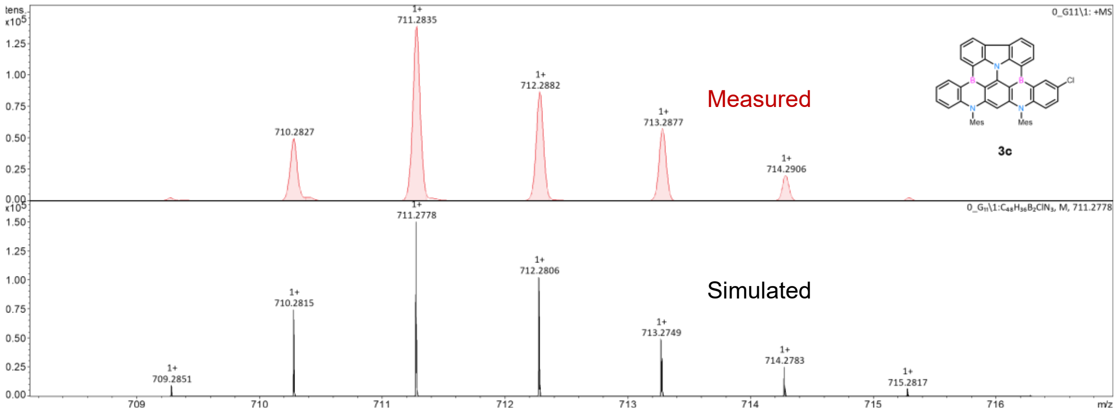

HR-MS (MALDI-TOF) spectrum of compound **3d**

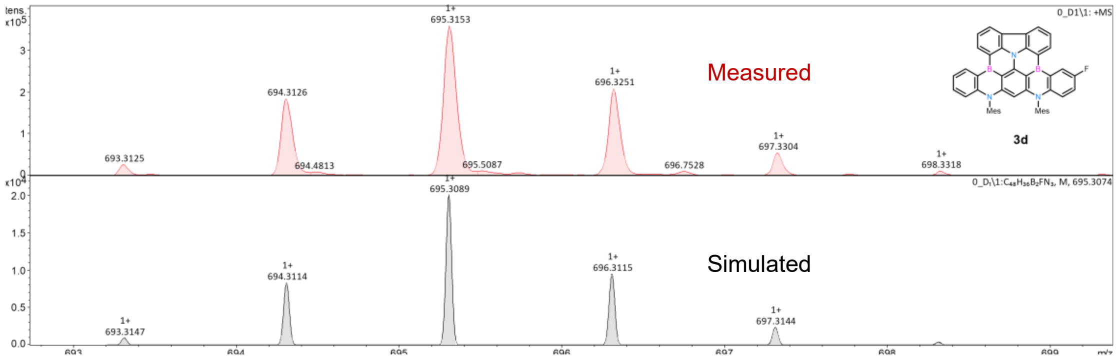

HR-MS (MALDI-TOF) spectrum of compound **3e**

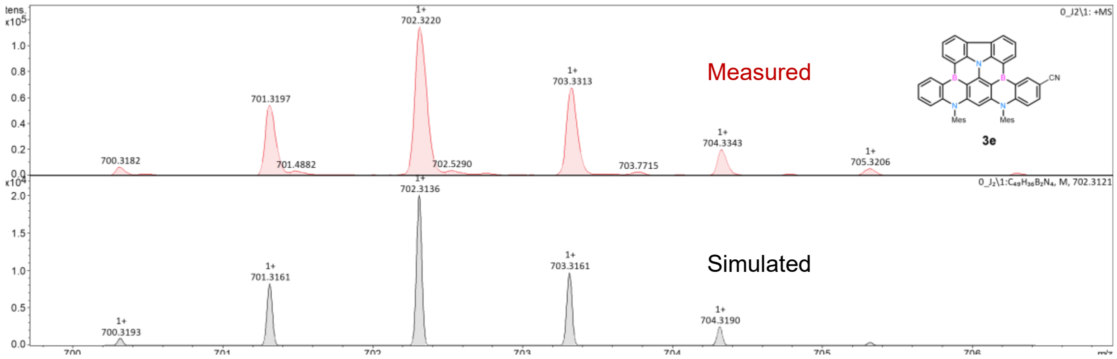

HR-MS (MALDI-TOF) spectrum of compound **4a/5a**

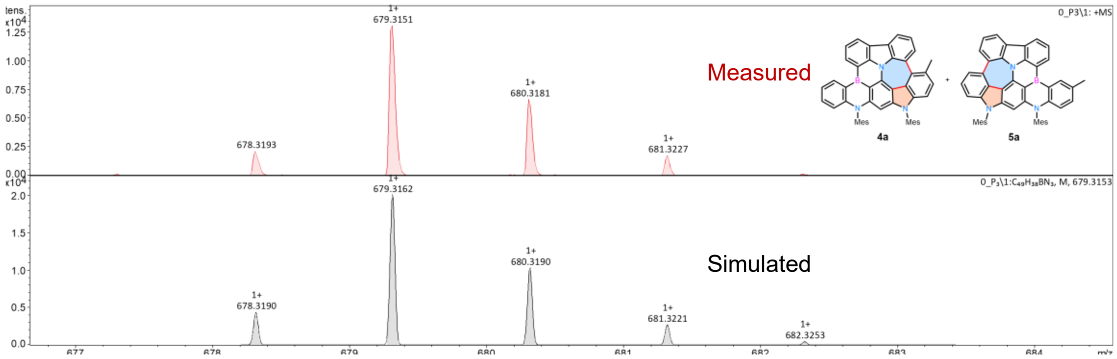

HR-MS (MALDI-TOF) spectrum of compound **4b’**

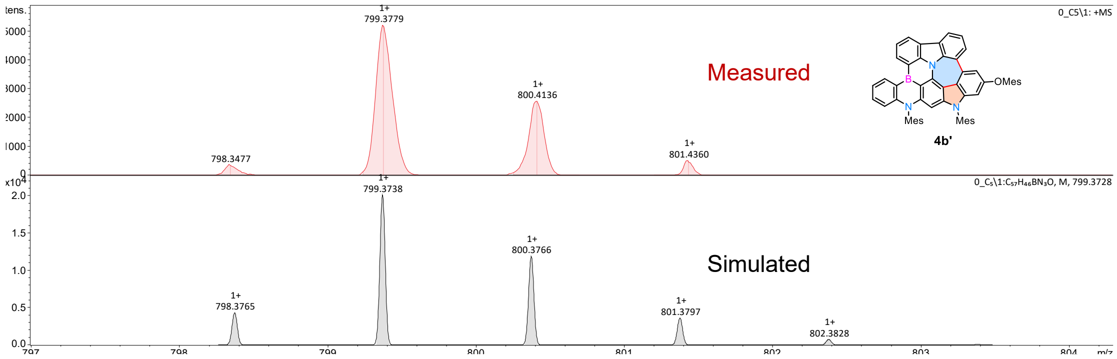

HR-MS (MALDI-TOF) spectrum of compound **5b**

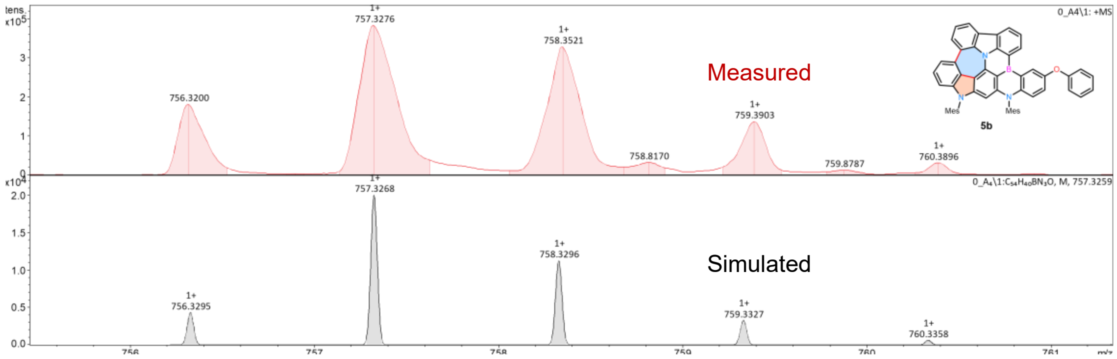

HR-MS (MALDI-TOF) spectrum of compound **5c**

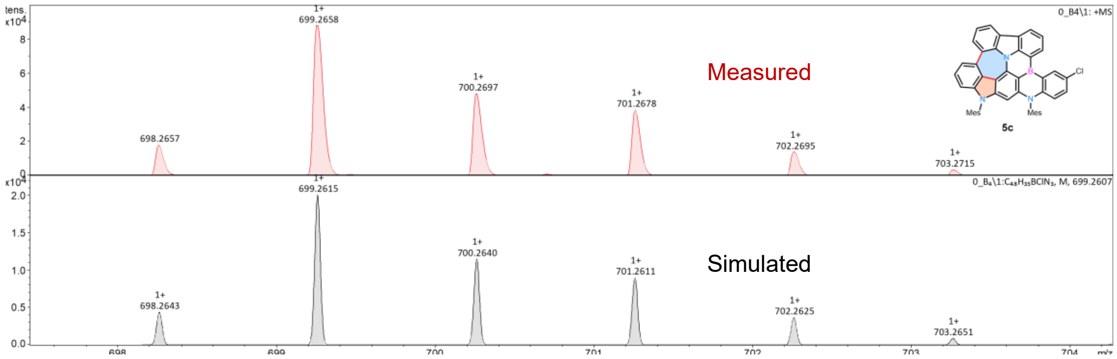

HR-MS (MALDI-TOF) spectrum of compound **5d**

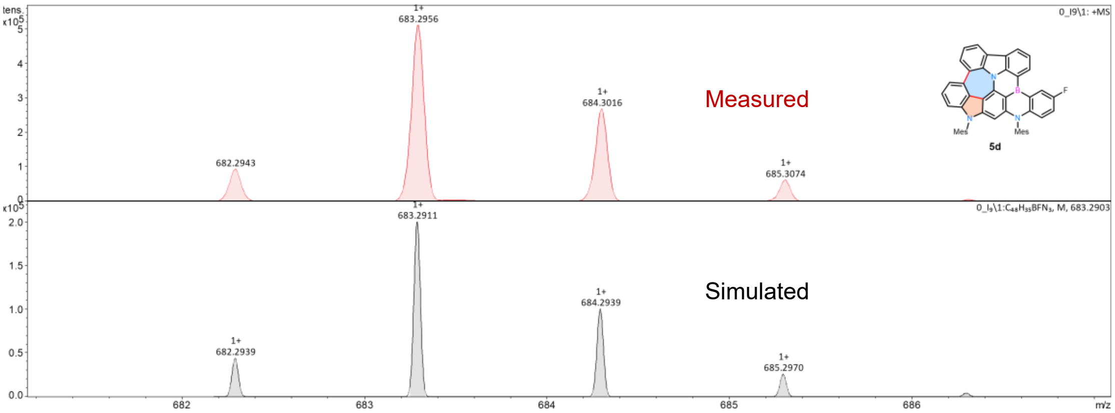

HR-MS (MALDI-TOF) spectrum of compound **7a**

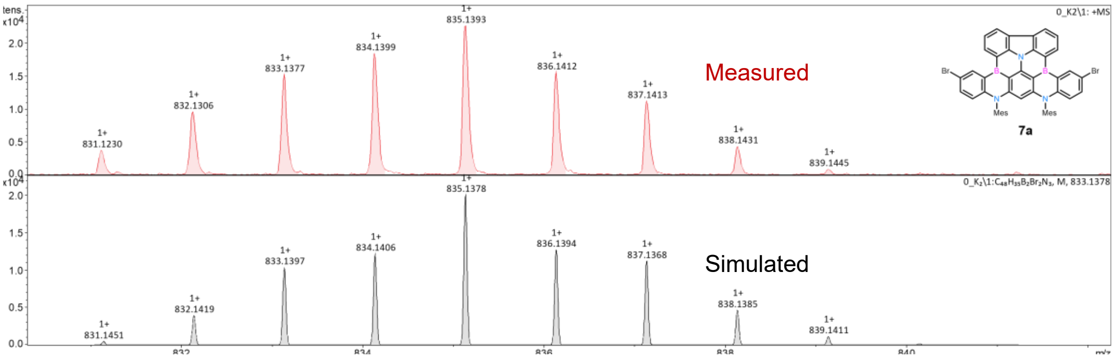

HR-MS (MALDI-TOF) spectrum of compound **7b**

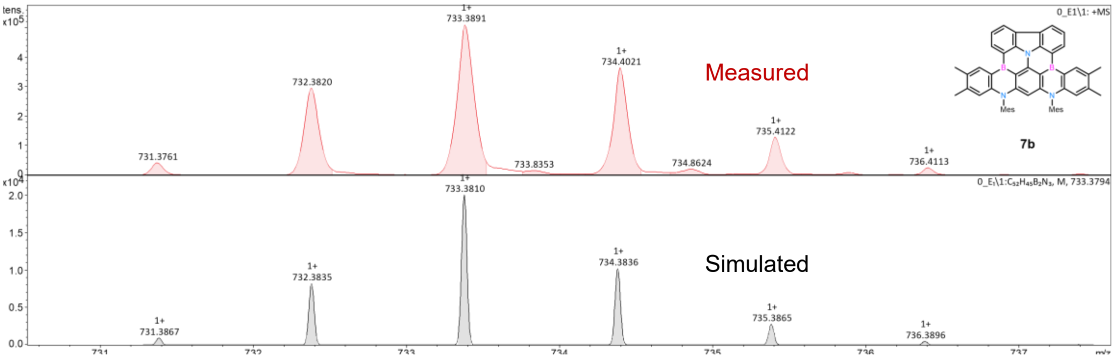

HR-MS (MALDI-TOF) spectrum of compound **7c**

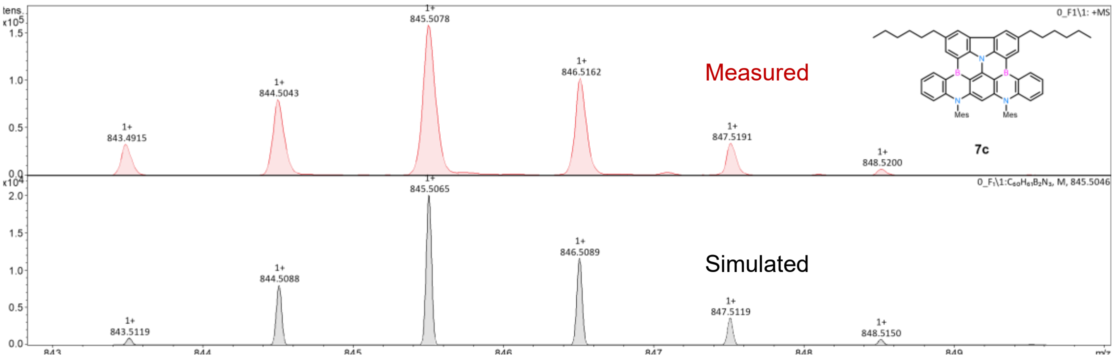

HR-MS (MALDI-TOF) spectrum of compound **7d**

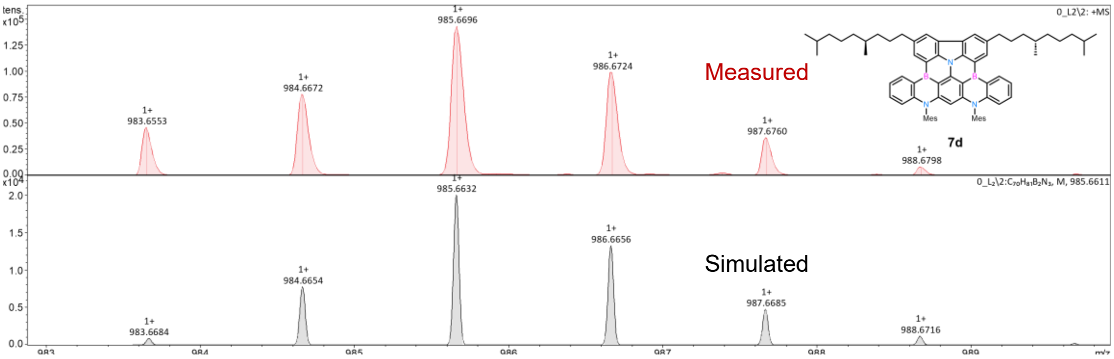

HR-MS (MALDI-TOF) spectrum of compound **7e**

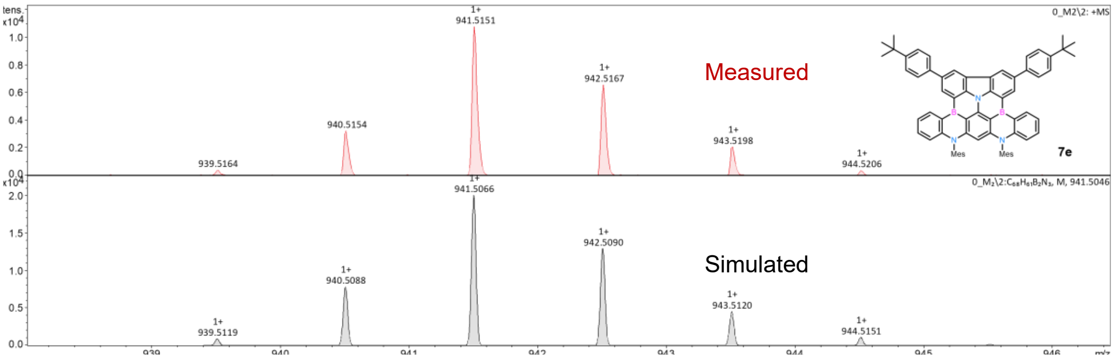

HR-MS (MALDI-TOF) spectrum of compound **7f**

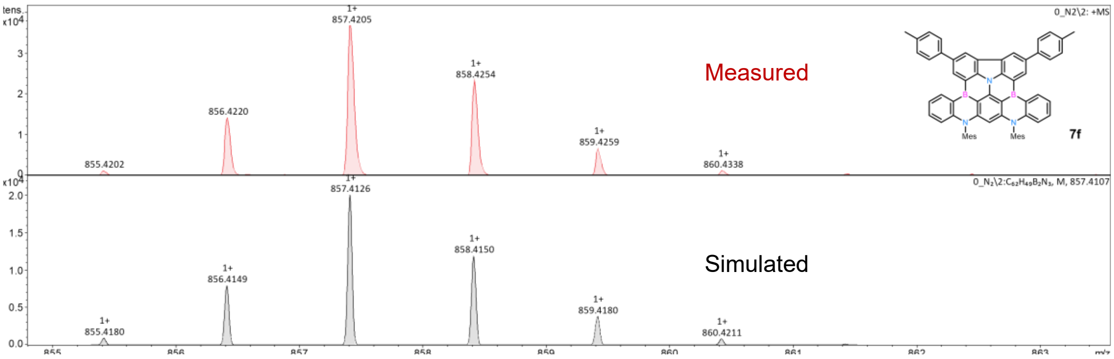

HR-MS (MALDI-TOF) spectrum of compound **7g**

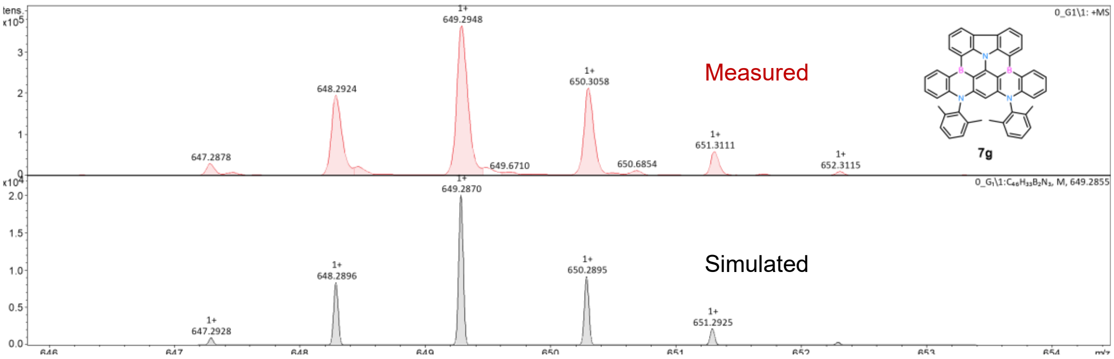

HR-MS (MALDI-TOF) spectrum of compound **7h**

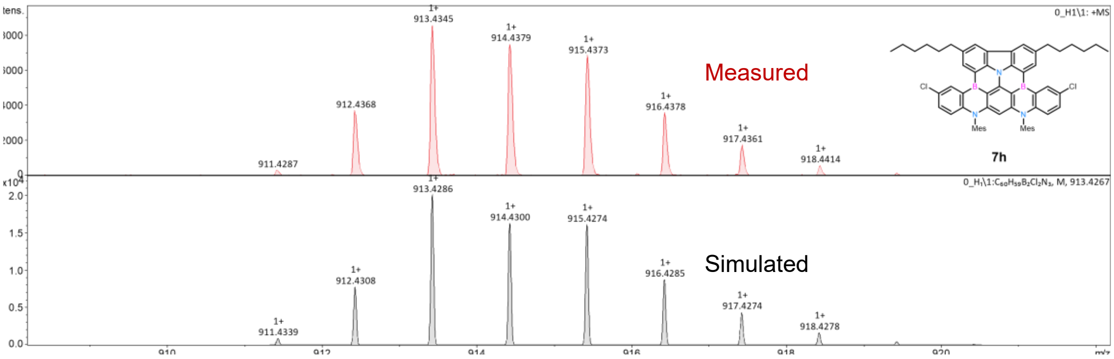

HR-MS (MALDI-TOF) spectrum of compound **7i**

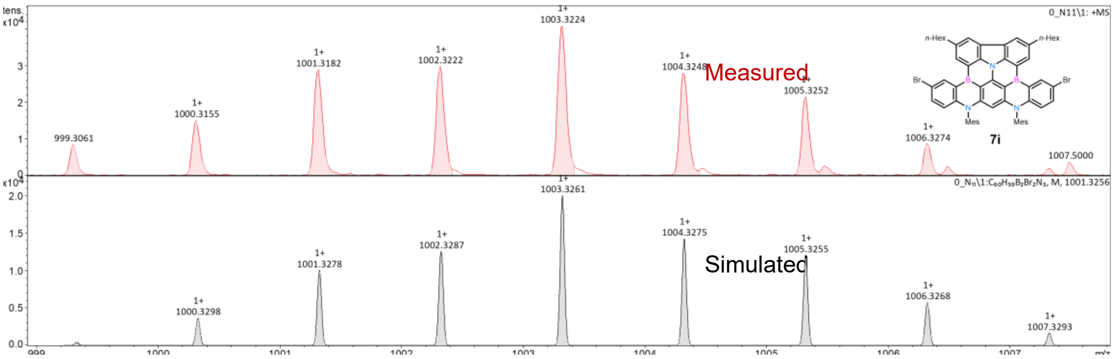

HR-MS (MALDI-TOF) spectrum of compound **7j**

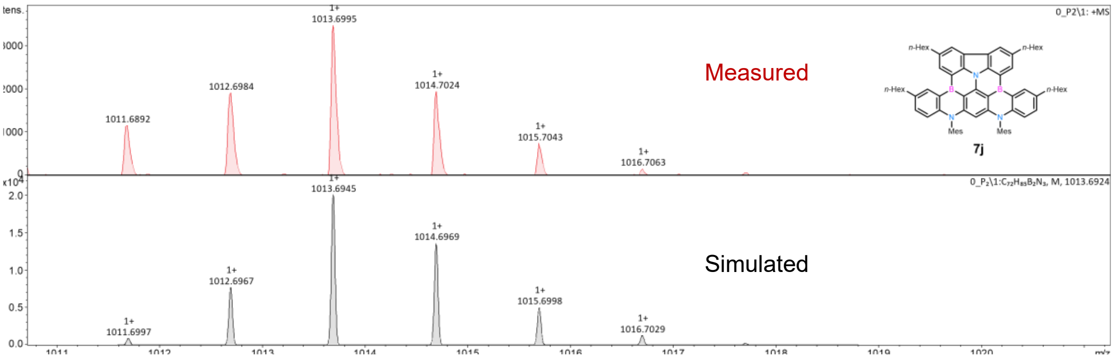

HR-MS (MALDI-TOF) spectrum of compound **7k**

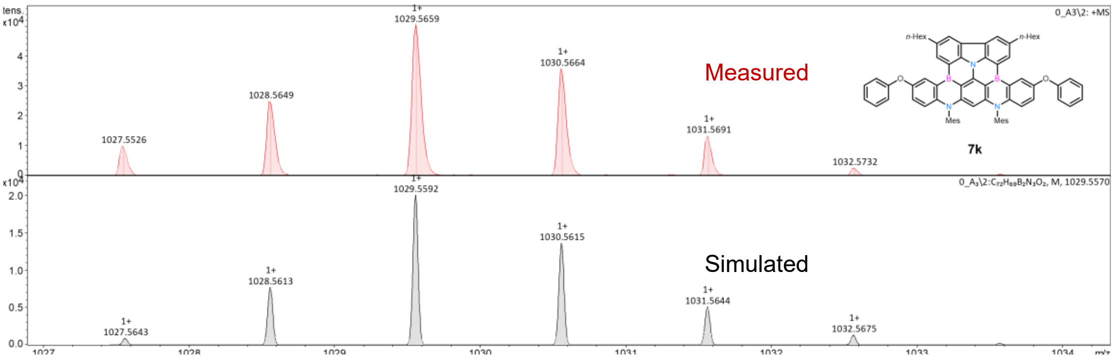

HR-MS (MALDI-TOF) spectrum of compound **7l**

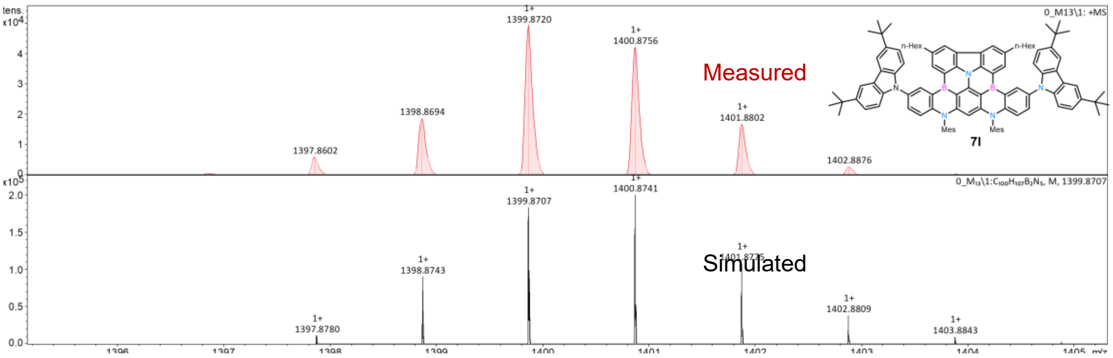

HR-MS (MALDI-TOF) spectrum of compound **7m**

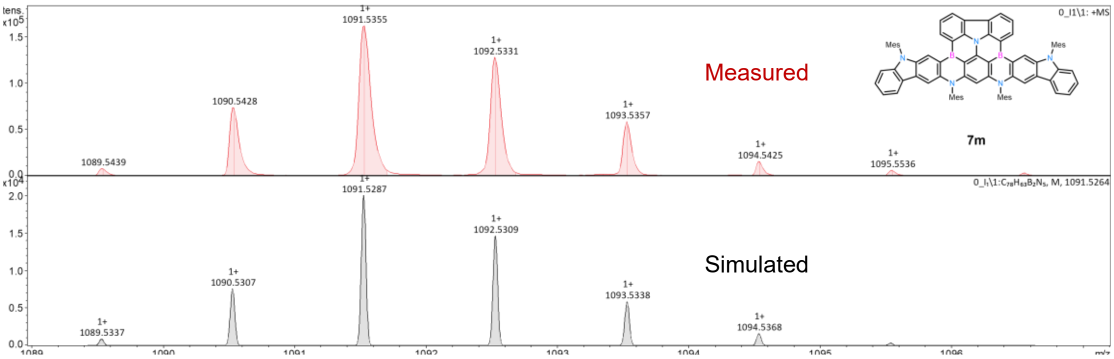

HR-MS (MALDI-TOF) spectrum of compound **7n**

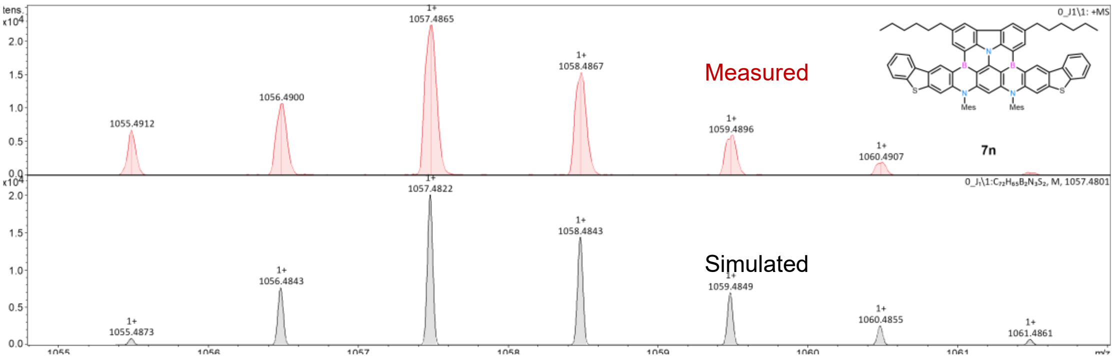

HR-MS (MALDI-TOF) spectrum of compound **7o**

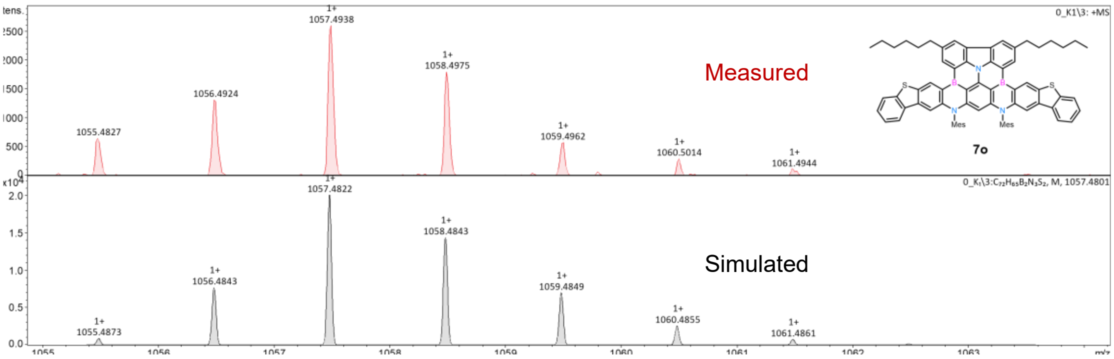

HR-MS (MALDI-TOF) spectrum of compound **7p**

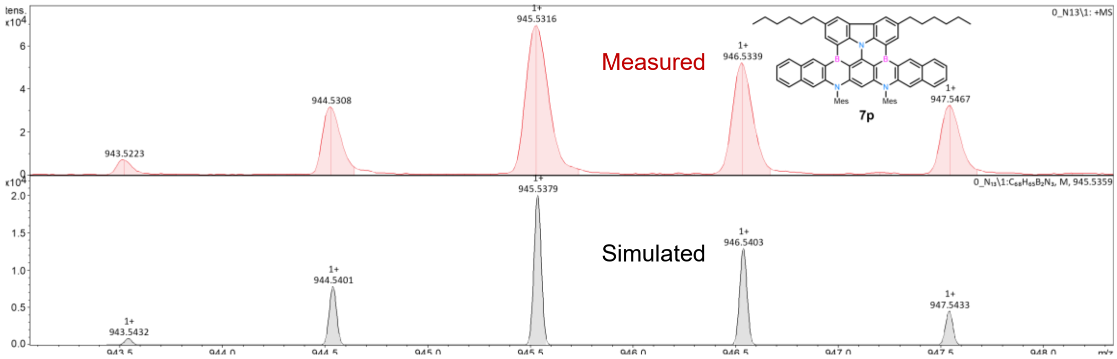

HR-MS (MALDI-TOF) spectrum of compound **7q**

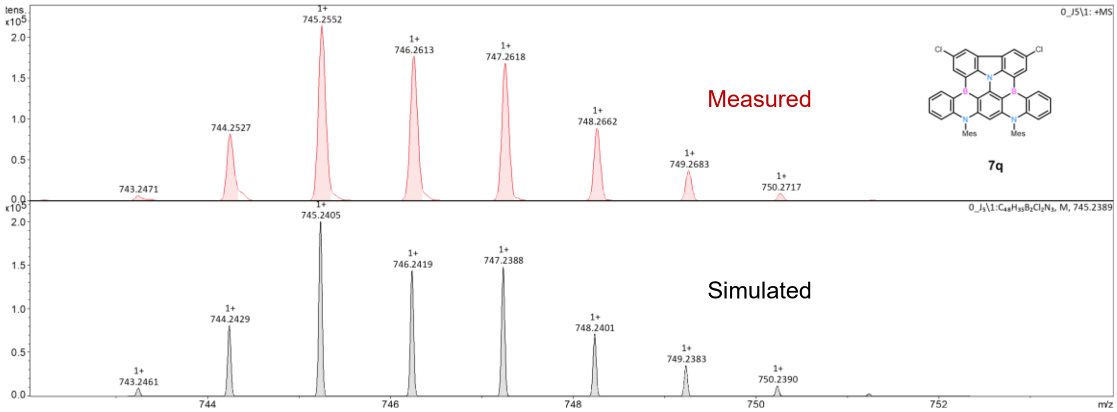

HR-MS (MALDI-TOF) spectrum of compound **8a**

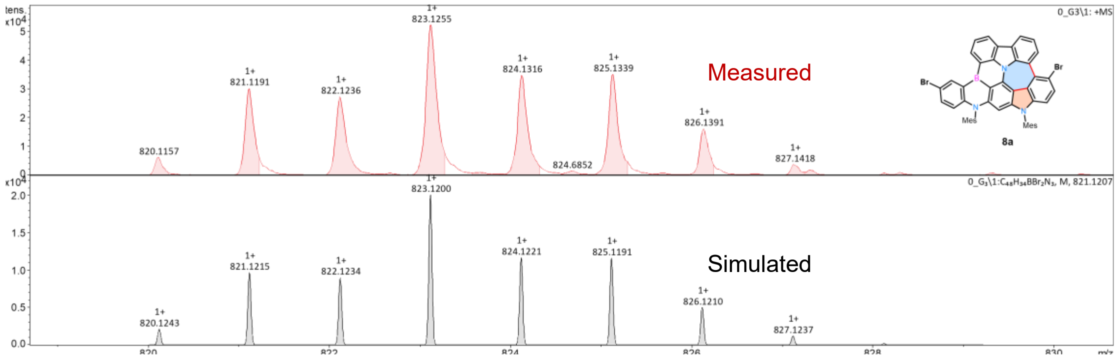

HR-MS (MALDI-TOF) spectrum of compound **8b**

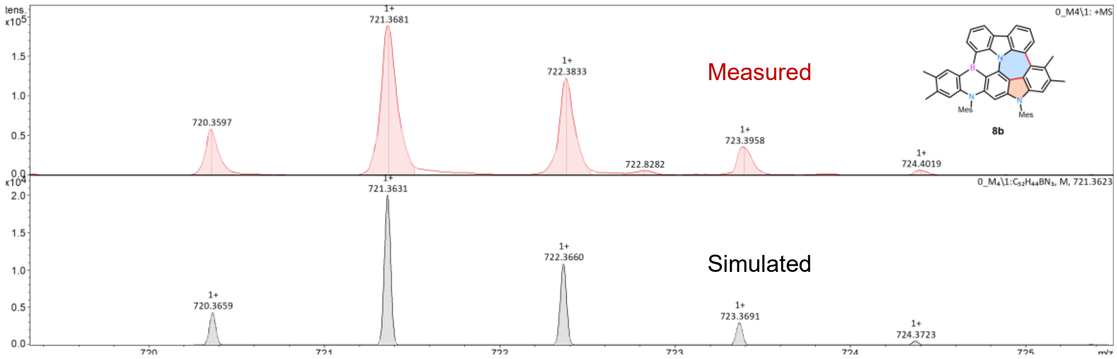

HR-MS (MALDI-TOF) spectrum of compound **8c**

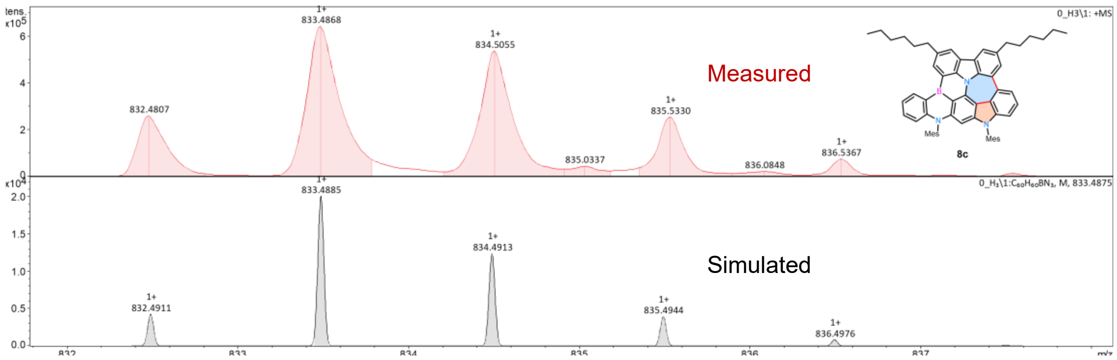

HR-MS (MALDI-TOF) spectrum of compound **8d**

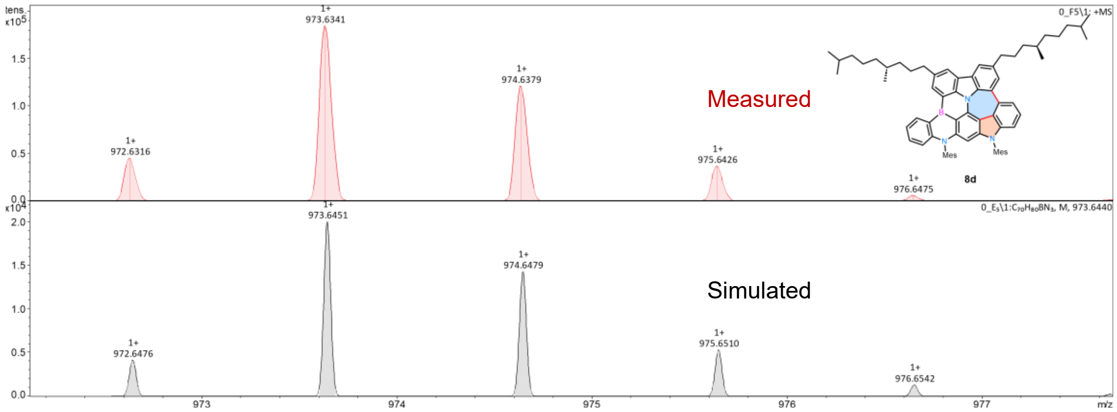

HR-MS (MALDI-TOF) spectrum of compound **8e**

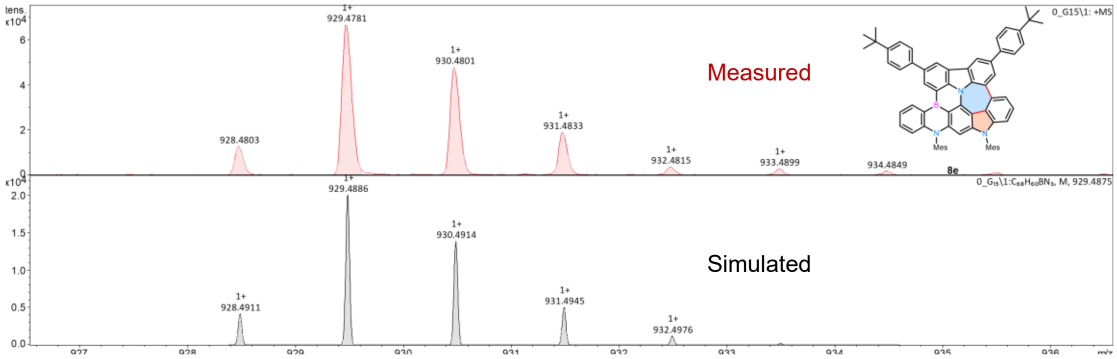

HR-MS (MALDI-TOF) spectrum of compound **8f**

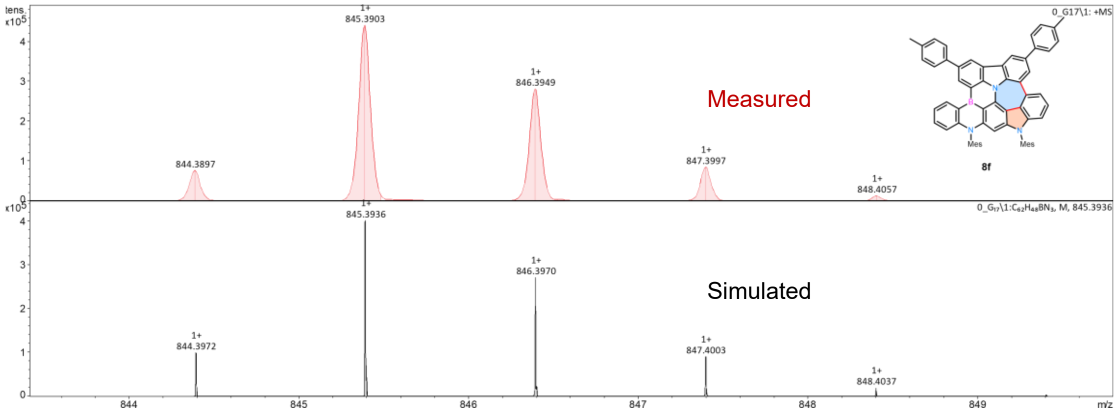

HR-MS (MALDI-TOF) spectrum of compound **8g**

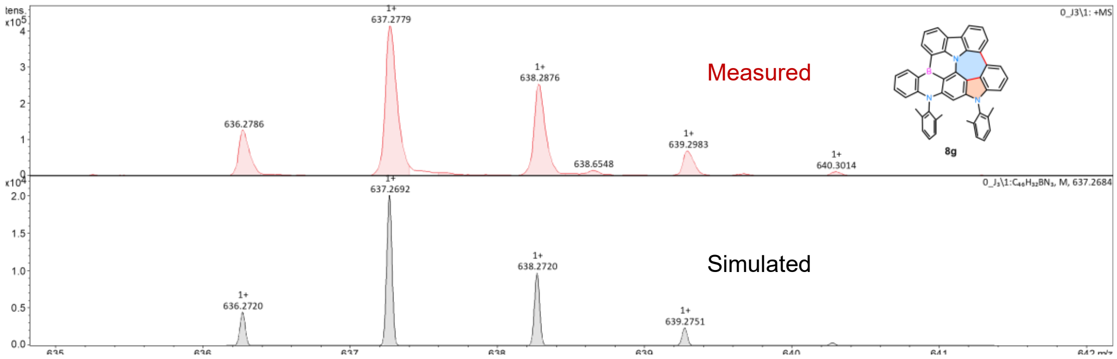

HR-MS (MALDI-TOF) spectrum of compound **8h**

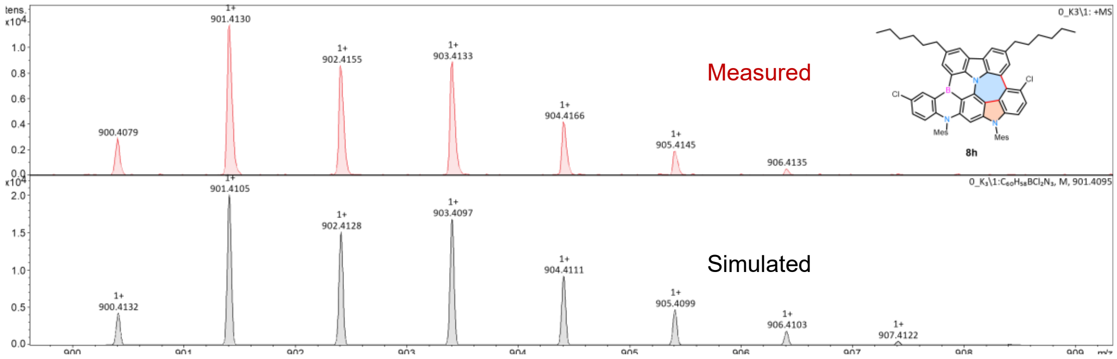

HR-MS (MALDI-TOF) spectrum of compound **8i**

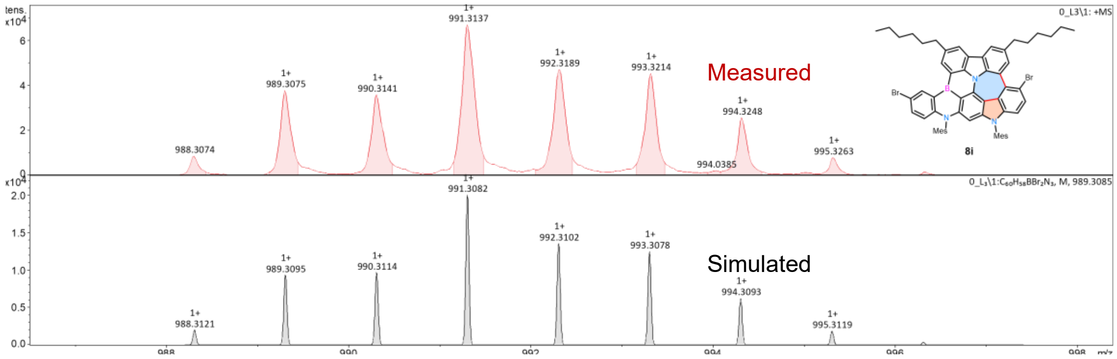

HR-MS (MALDI-TOF) spectrum of compound **8j**

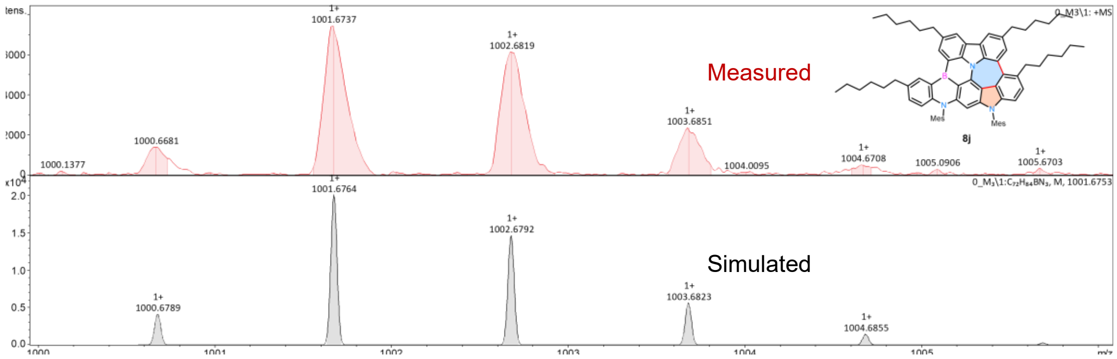

HR-MS (MALDI-TOF) spectrum of compound **8k**

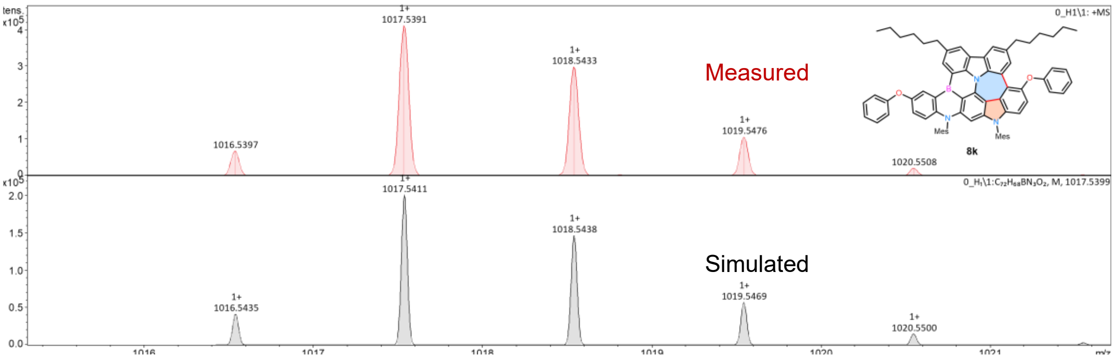

HR-MS (MALDI-TOF) spectrum of compound **8l**

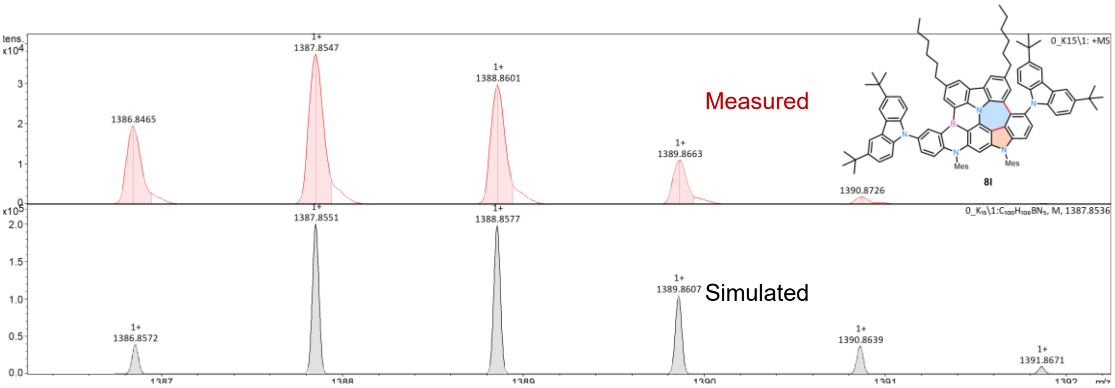

HR-MS (MALDI-TOF) spectrum of compound **8m**

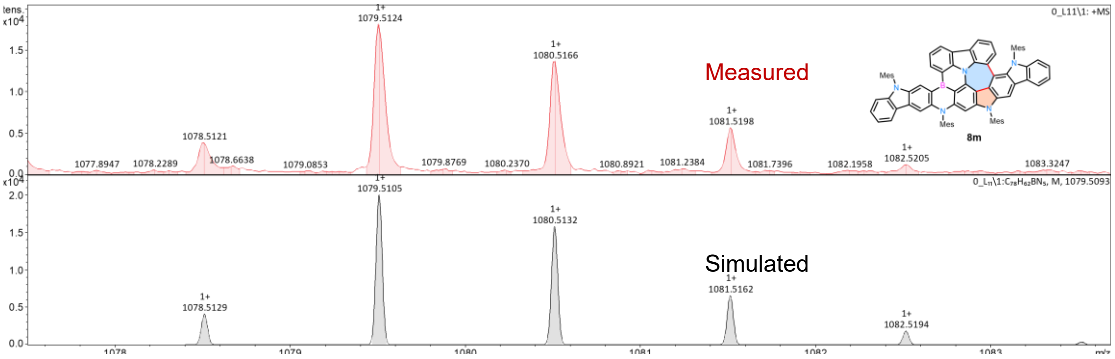

HR-MS (MALDI-TOF) spectrum of compound **8n**

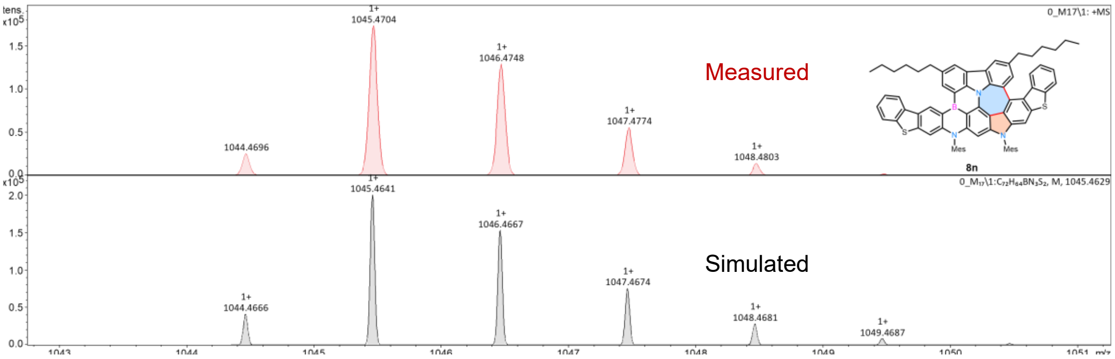

HR-MS (MALDI-TOF) spectrum of compound **8o**

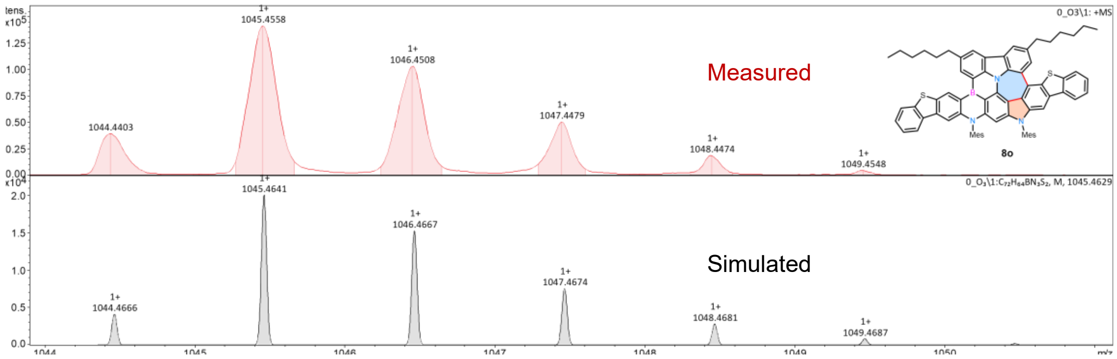

HR-MS (MALDI-TOF) spectrum of compound **8p**

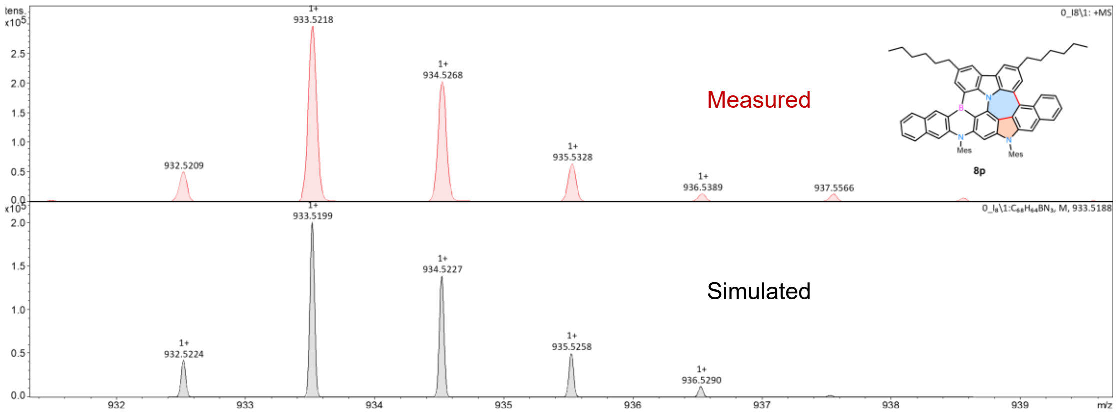

HR-MS (MALDI-TOF) spectrum of compound **9a**

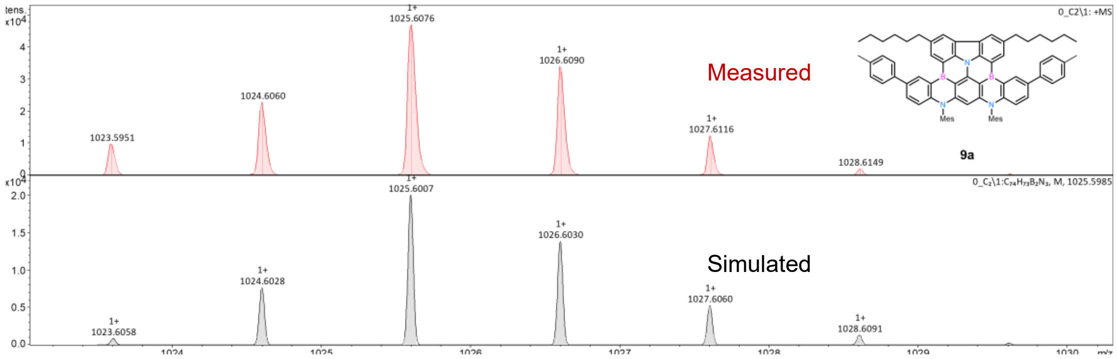

HR-MS (MALDI-TOF) spectrum of compound **9b**

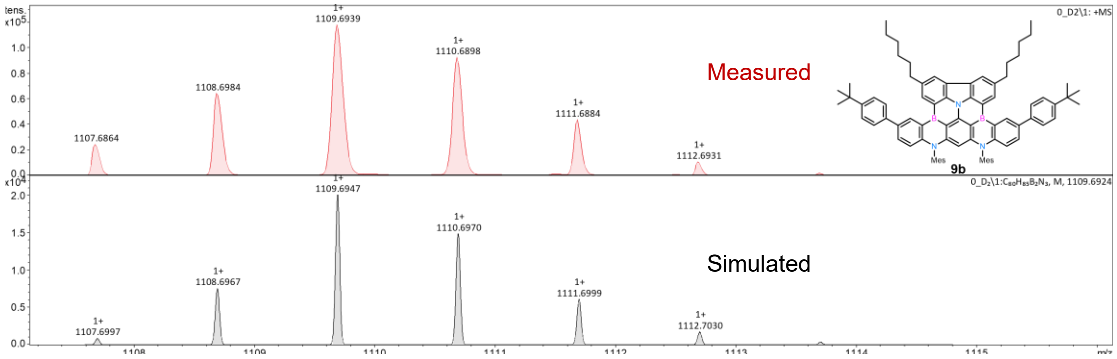

HR-MS (MALDI-TOF) spectrum of compound **9c**

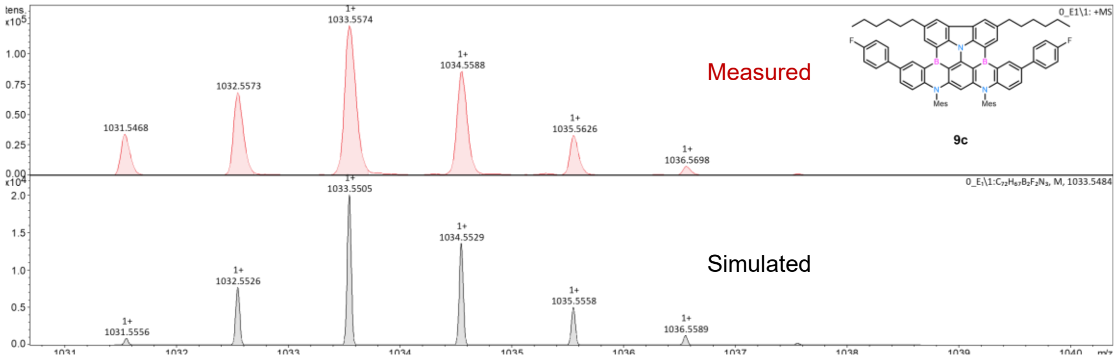

HR-MS (MALDI-TOF) spectrum of compound **9d**

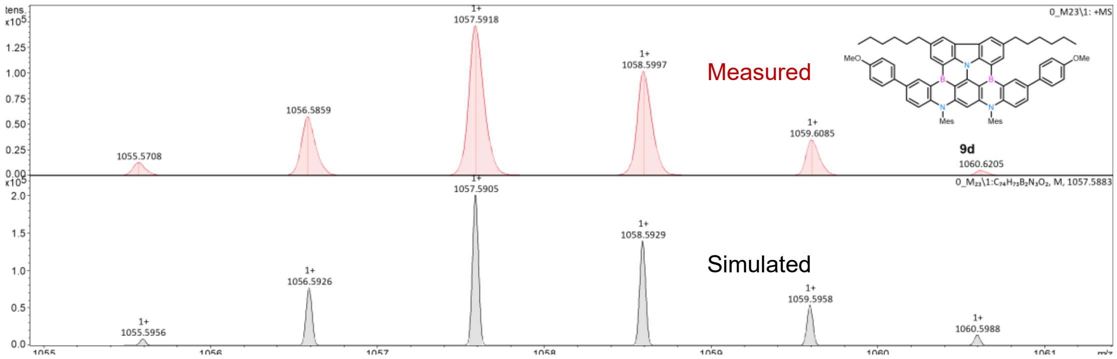

HR-MS (MALDI-TOF) spectrum of compound **9e**

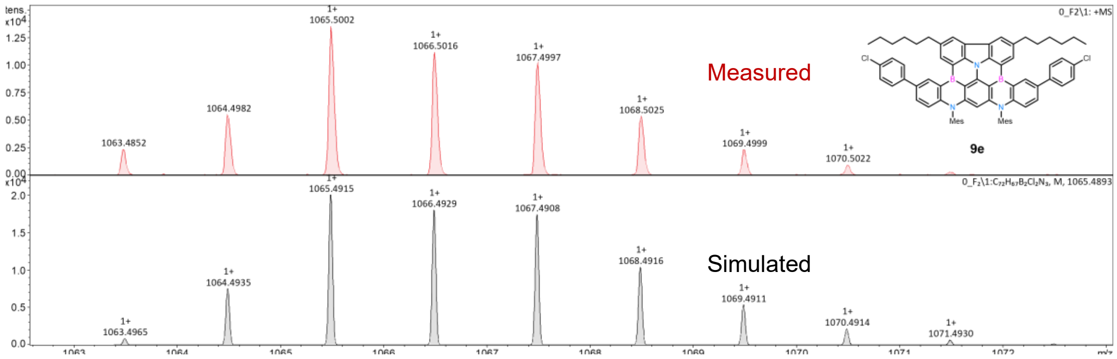

HR-MS (MALDI-TOF) spectrum of compound **9f**

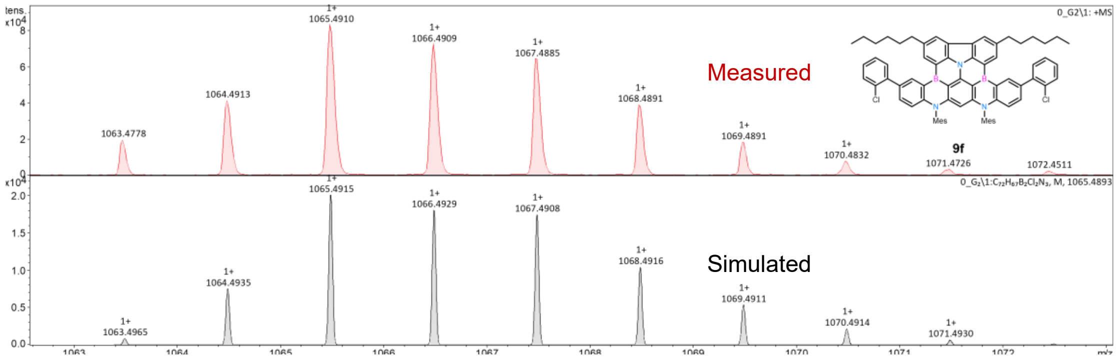

HR-MS (MALDI-TOF) spectrum of compound **9g**

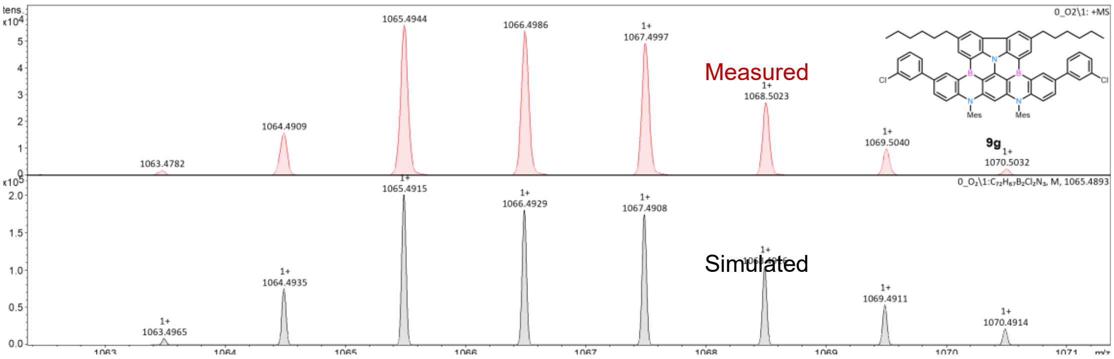

HR-MS (MALDI-TOF) spectrum of compound **9h**

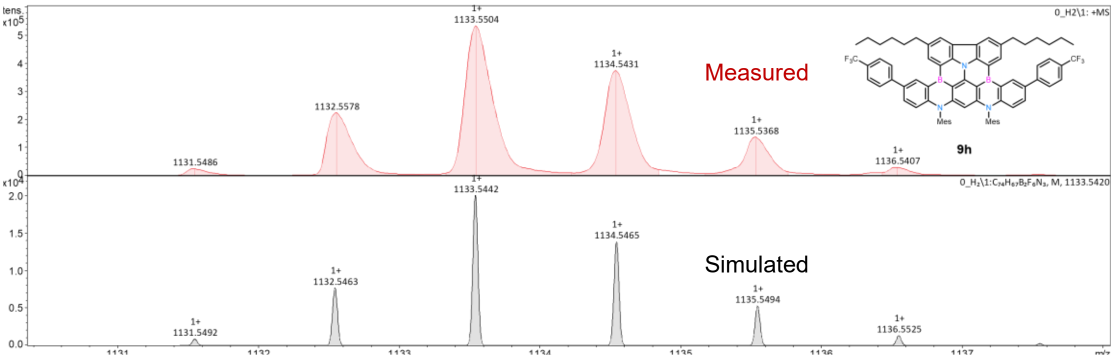

HR-MS (MALDI-TOF) spectrum of compound **9i**

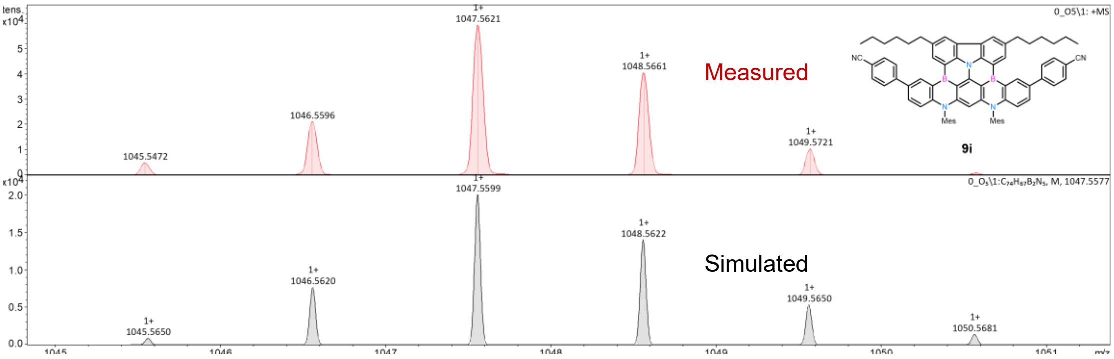

HR-MS (MALDI-TOF) spectrum of compound **9j**

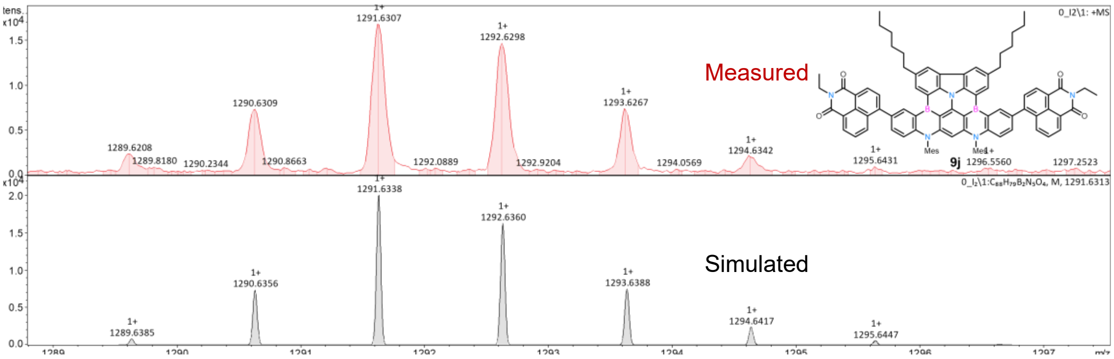

HR-MS (MALDI-TOF) spectrum of compound **9k**

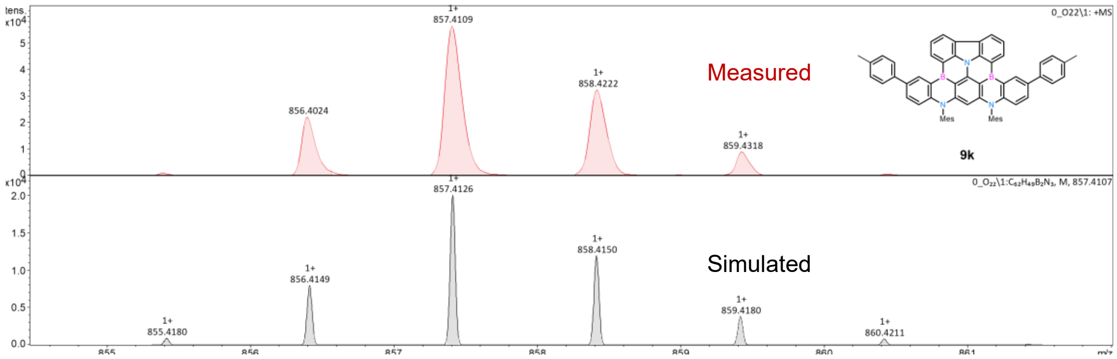

HR-MS (MALDI-TOF) spectrum of compound **10a**

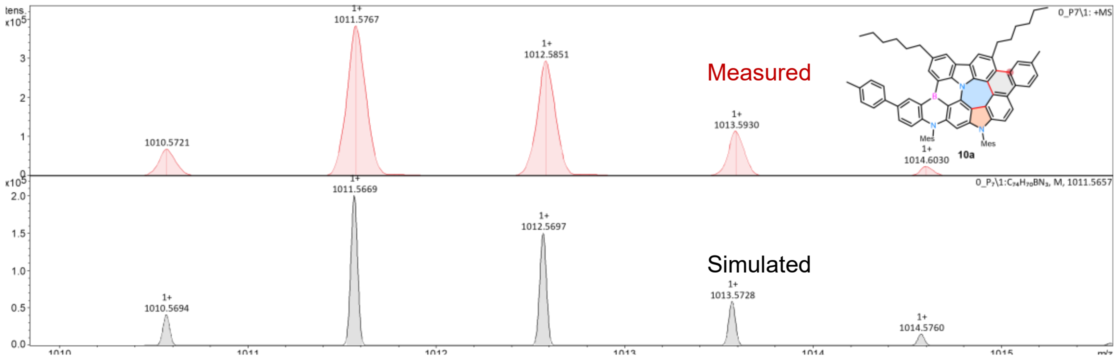

R-MS (MALDI-TOF) spectrum of compound **10b**

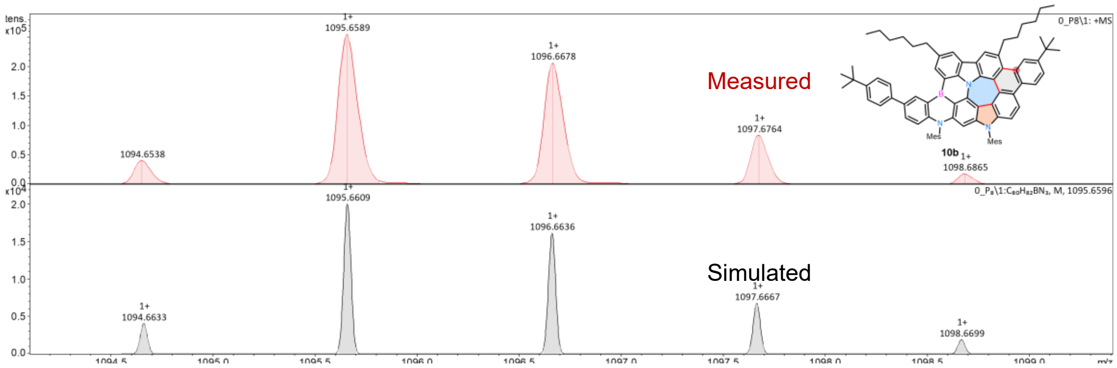

HR-MS (MALDI-TOF) spectrum of compound **10c**

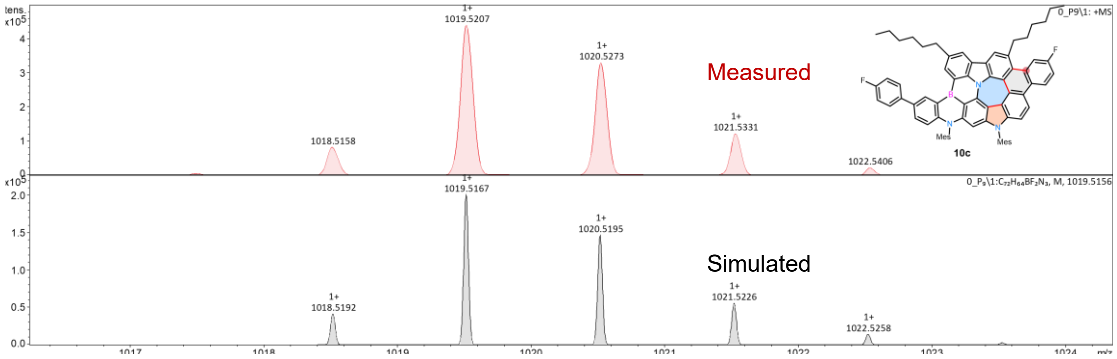

HR-MS (MALDI-TOF) spectrum of compound **10d**

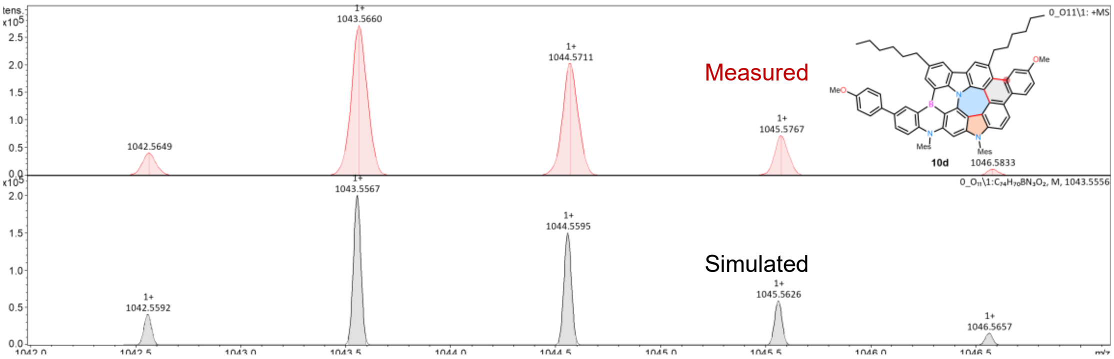

HR-MS (MALDI-TOF) spectrum of compound **10e**

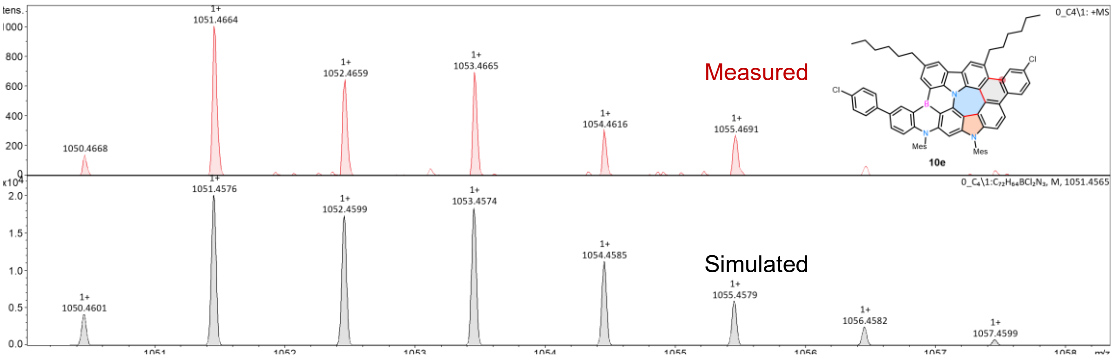

HR-MS (MALDI-TOF) spectrum of compound **10f**

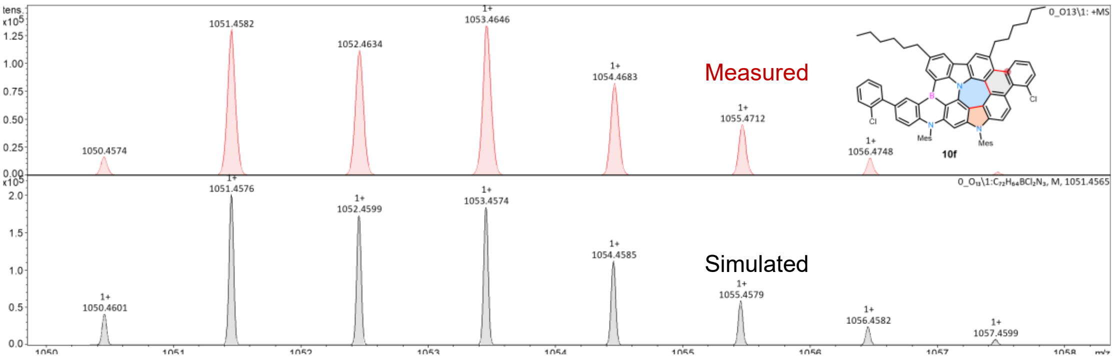

HR-MS (MALDI-TOF) spectrum of compound **10g**

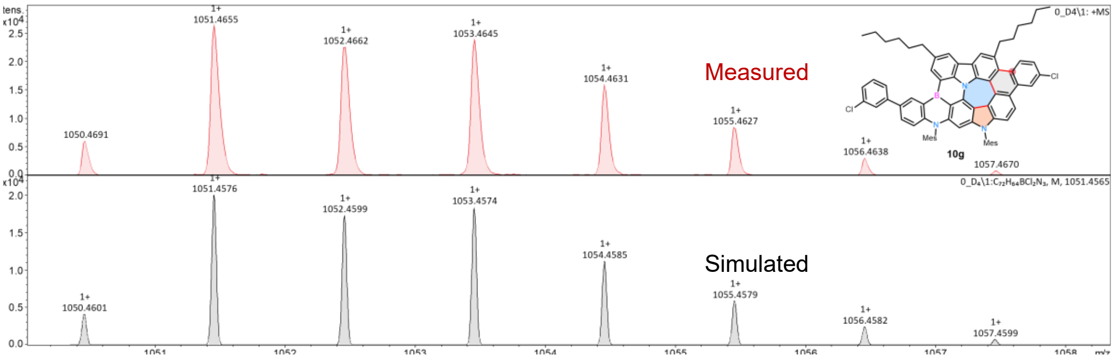

HR-MS (MALDI-TOF) spectrum of compound **10h**

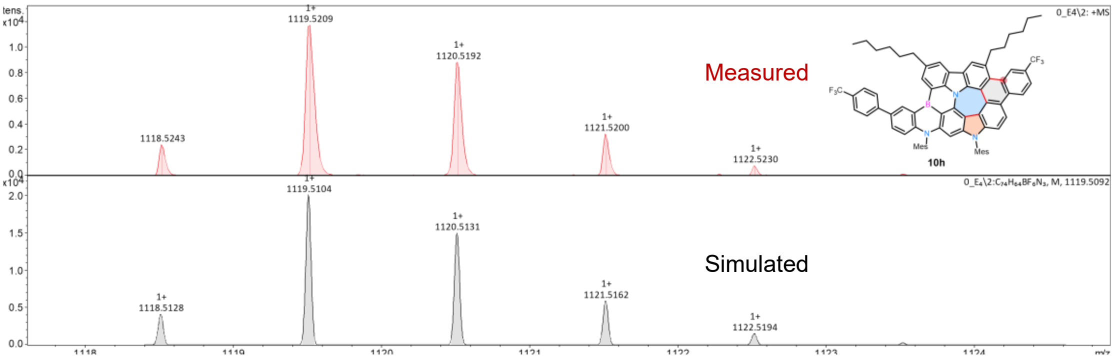

HR-MS (MALDI-TOF) spectrum of compound **10i**

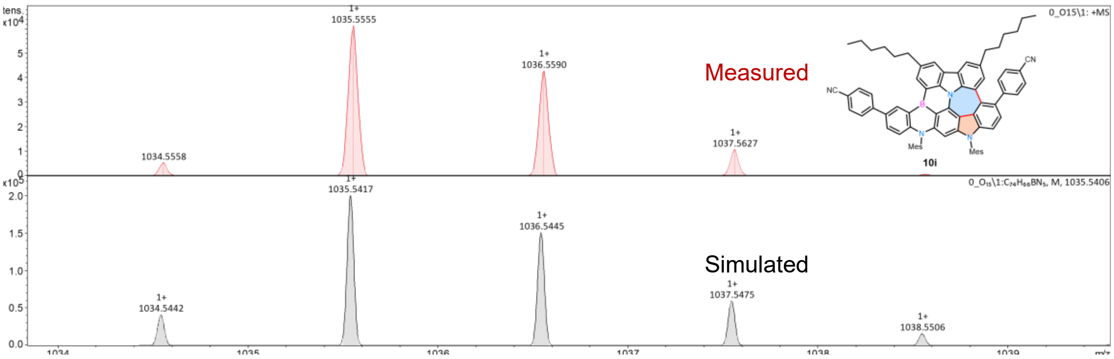

HR-MS (MALDI-TOF) spectrum of compound **10j**

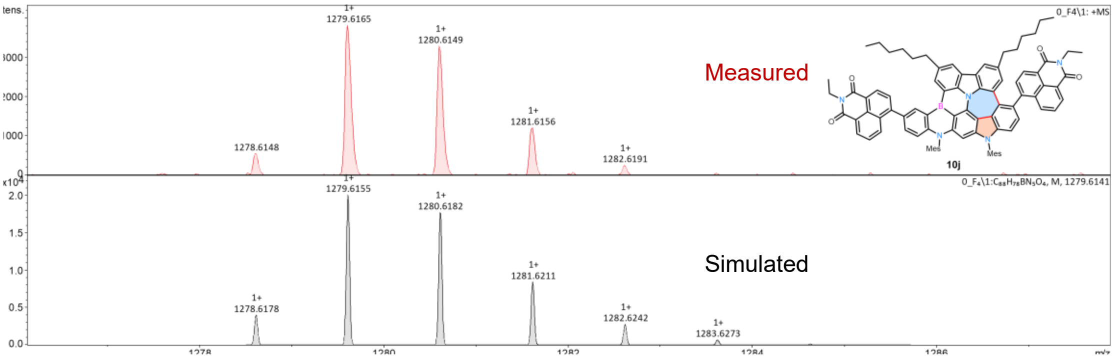

HR-MS (MALDI-TOF) spectrum of compound **10k**

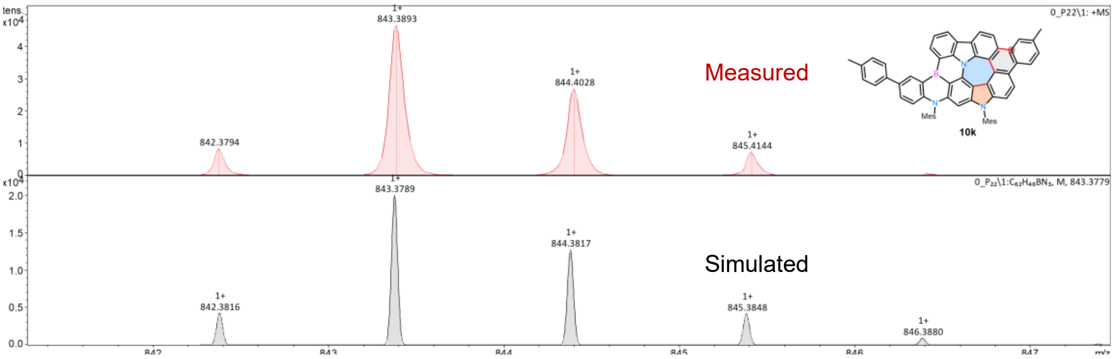

HR-MS (MALDI-TOF) spectrum of compound **11**

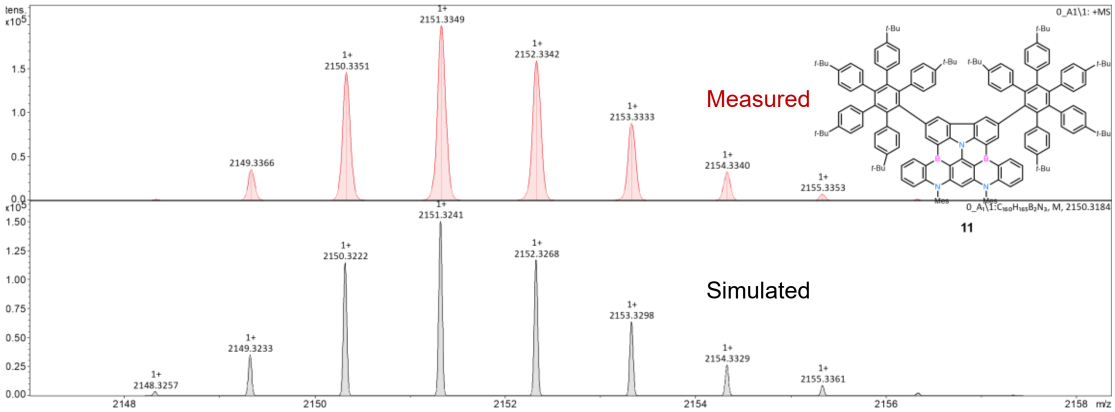

HR-MS (MALDI-TOF) spectrum of compound **12**

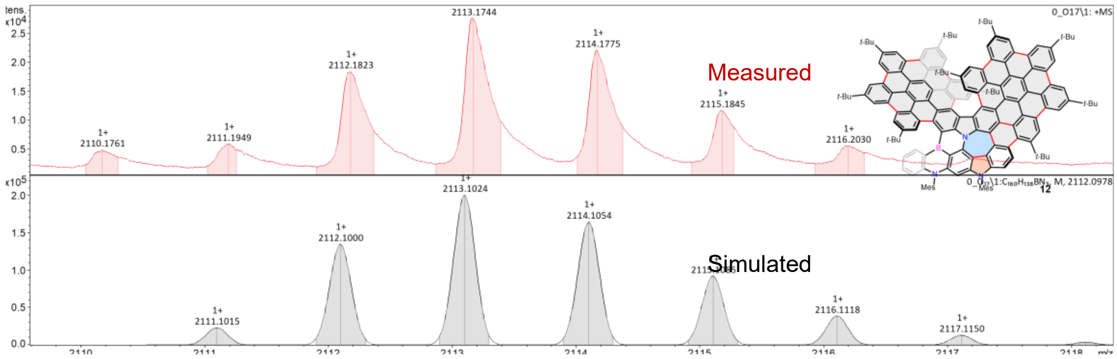

HR-MS (MALDI-TOF) spectrum of compound **13**

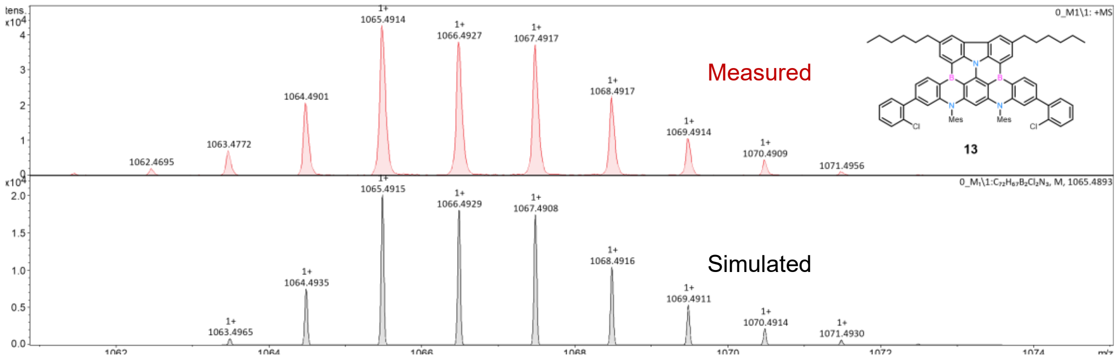

HR-MS (MALDI-TOF) spectrum of compound **14**

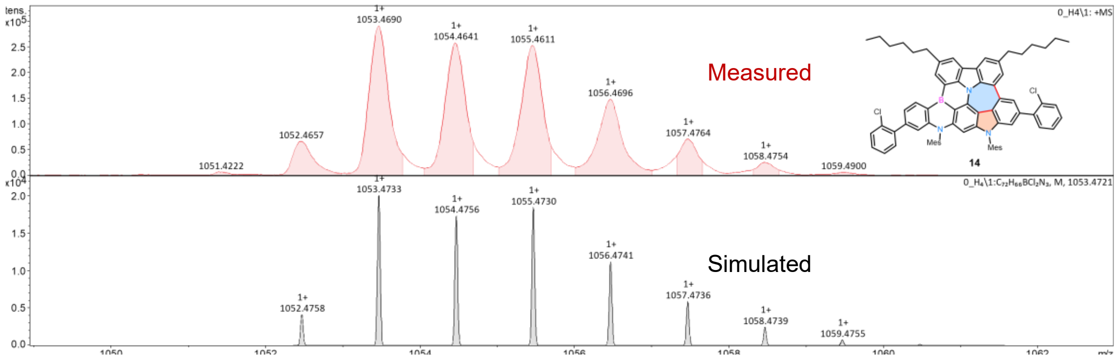

HR-MS (MALDI-TOF) spectrum of compound **15a**

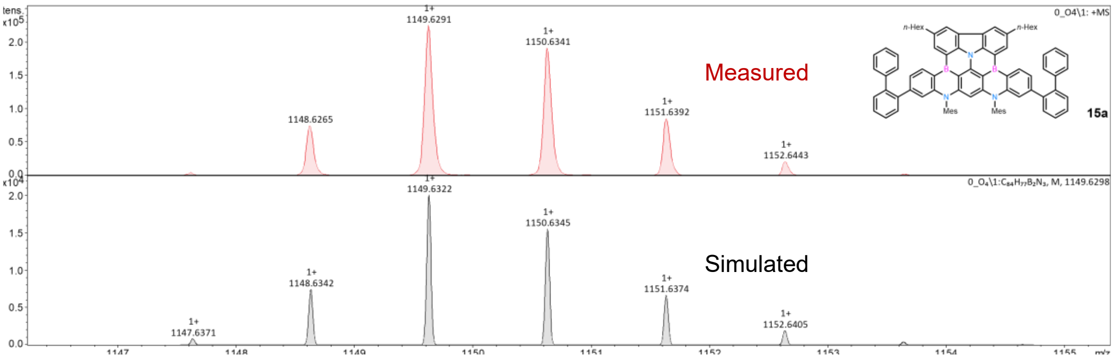

HR-MS (MALDI-TOF) spectrum of compound **15b**

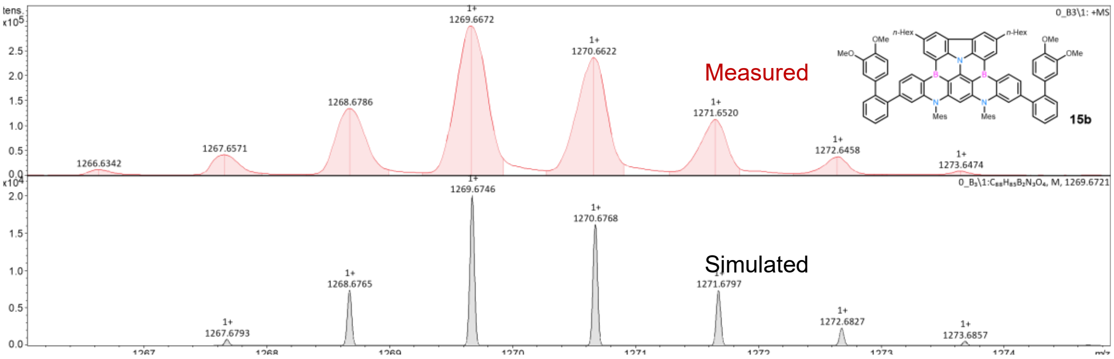

HR-MS (MALDI-TOF) spectrum of compound **16a**

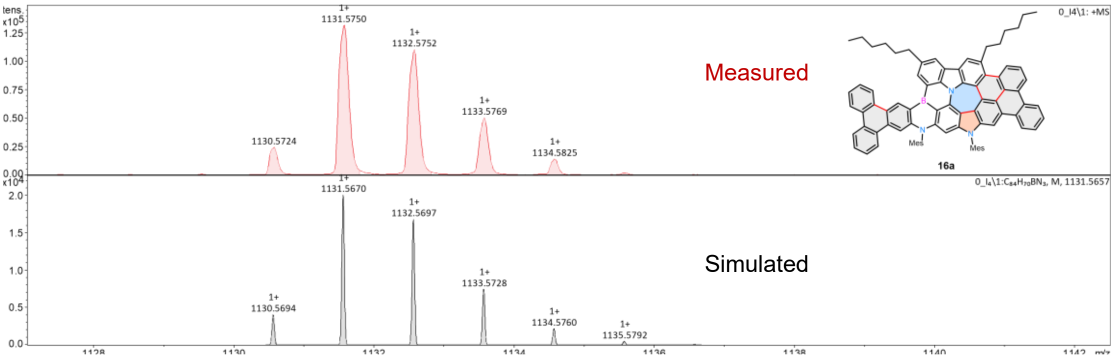

HR-MS (MALDI-TOF) spectrum of compound **16b**

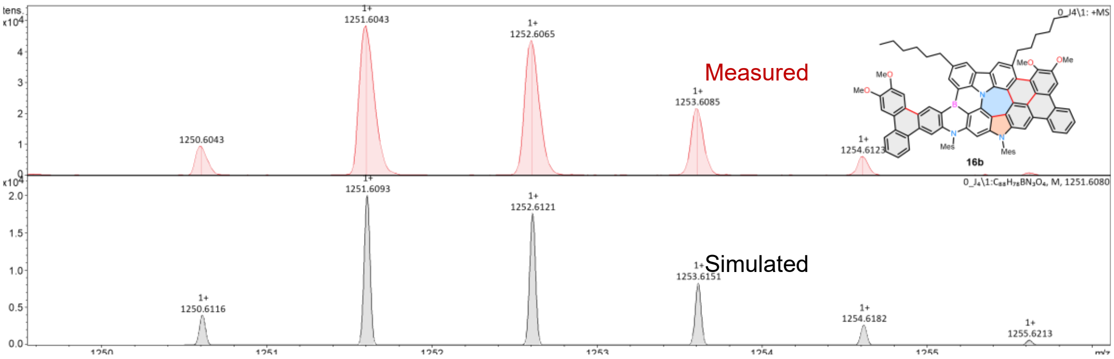

HR-MS (MALDI-TOF) spectrum of compound **17**

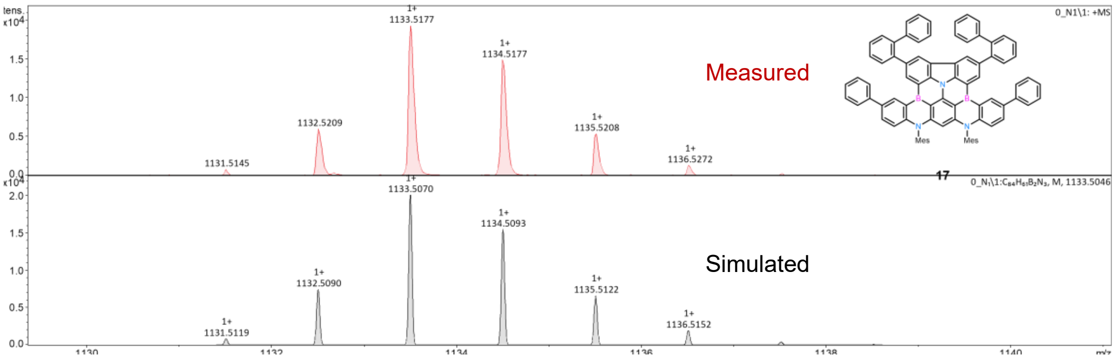

HR-MS (MALDI-TOF) spectrum of compound **18**

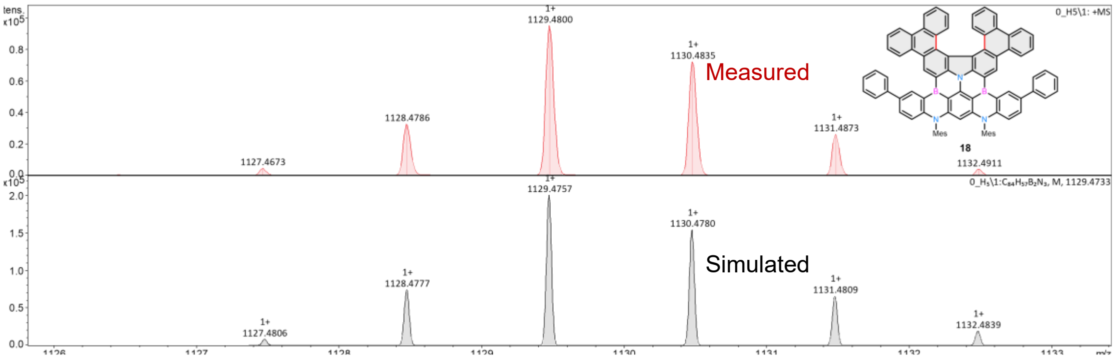

HR-MS (MALDI-TOF) spectrum of compound **19**

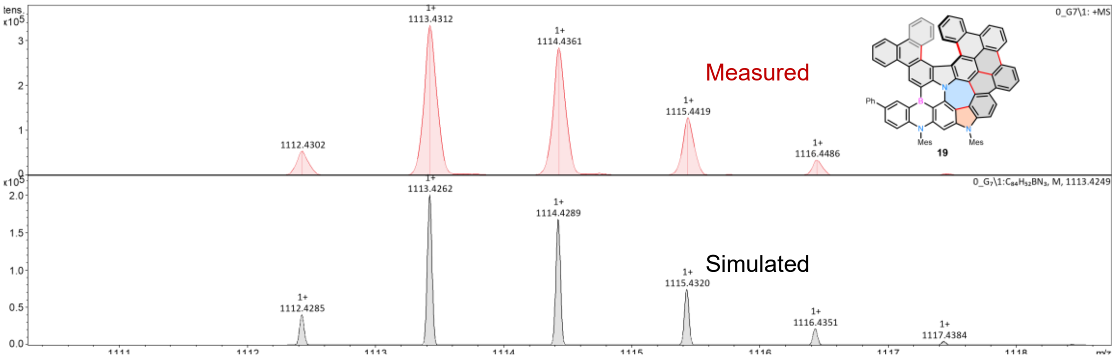

HR-MS (MALDI-TOF) spectrum of compound **20**

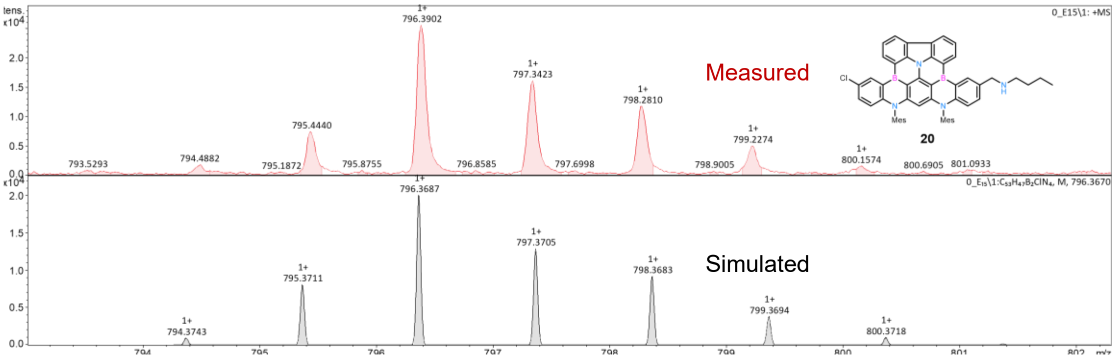

HR-MS (MALDI-TOF) spectrum of compound **21**

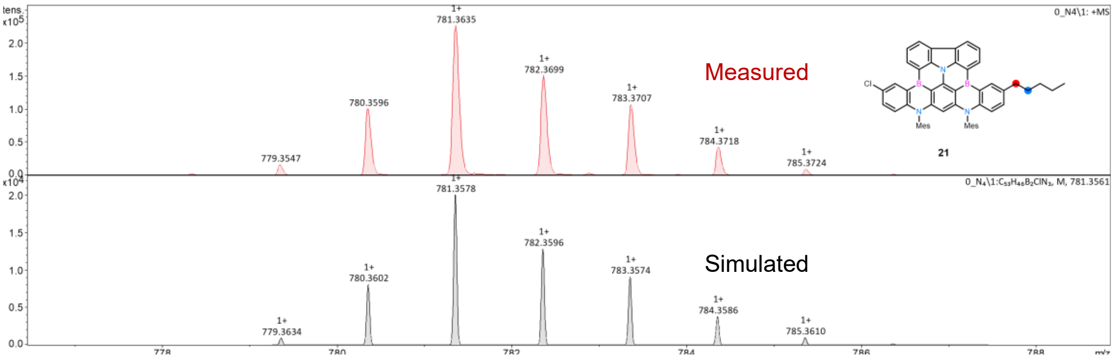

HR-MS (MALDI-TOF) spectrum of compound **22**

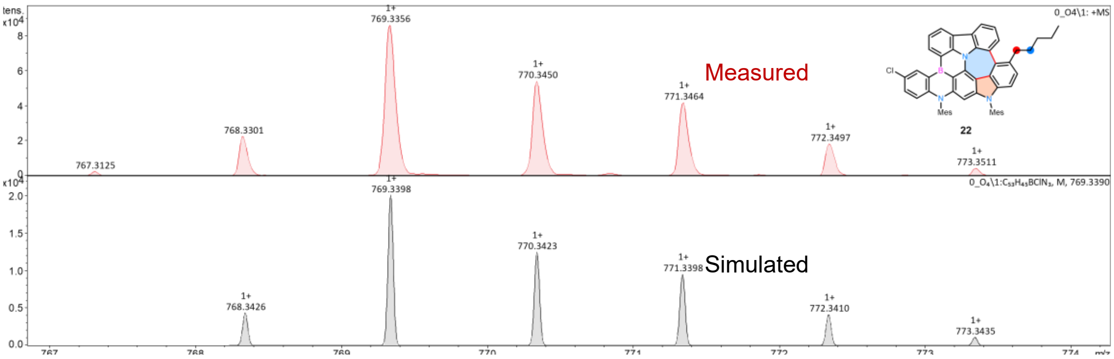

HR-MS (MALDI-TOF) spectrum of compound **23**

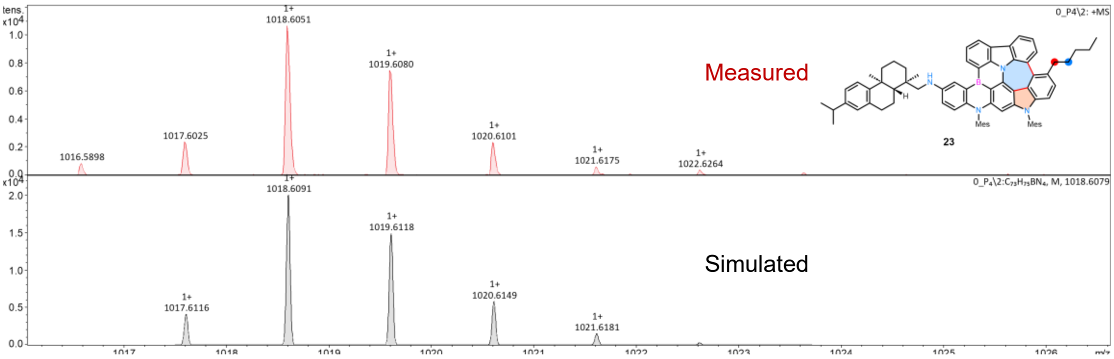

HR-MS (MALDI-TOF) spectrum of compound **24a**

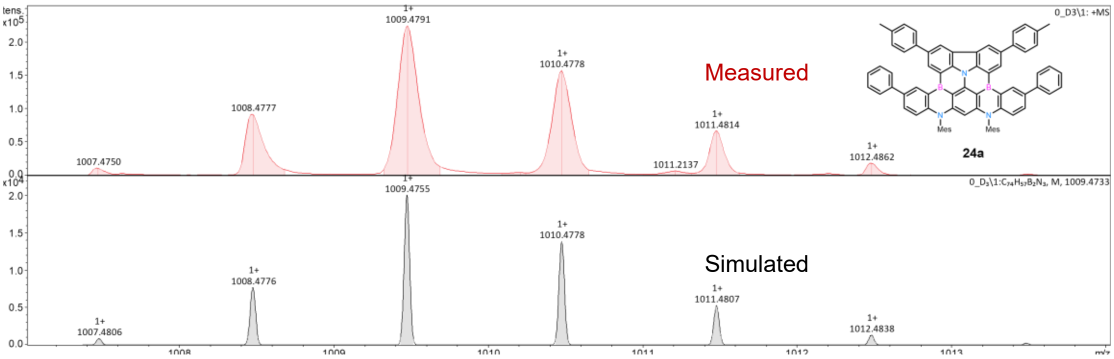

HR-MS (MALDI-TOF) spectrum of compound **24b**

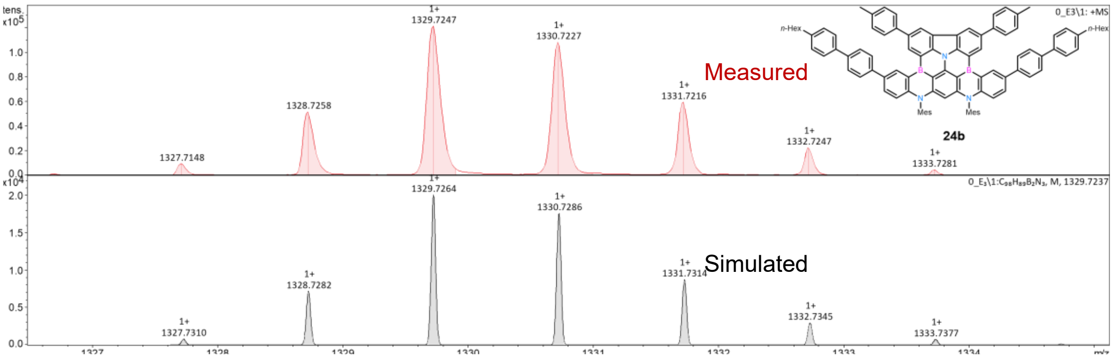

HR-MS (MALDI-TOF) spectrum of compound **25a**

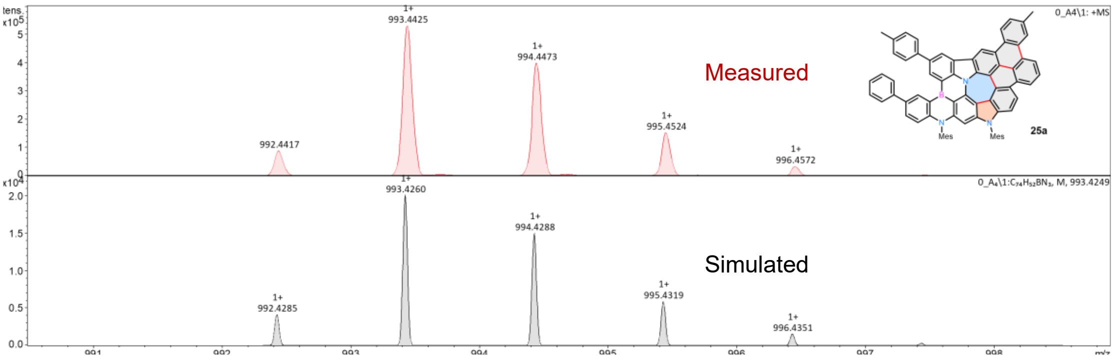

HR-MS (MALDI-TOF) spectrum of compound **25b**

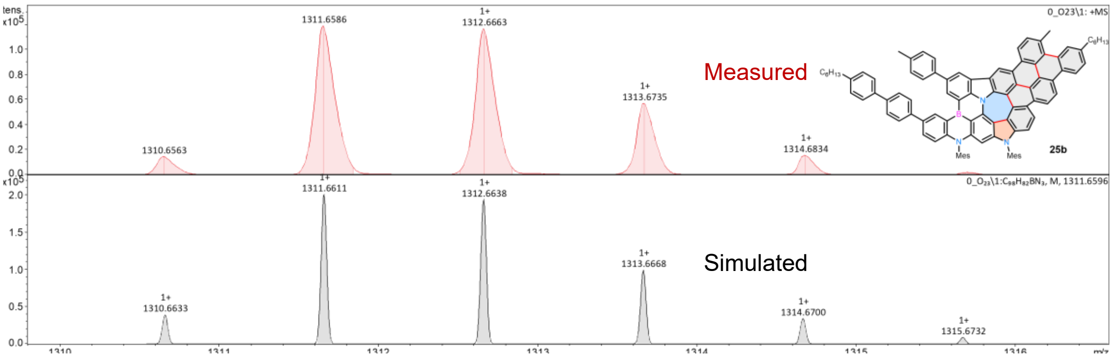

HR-MS (MALDI-TOF) spectrum of compound **26**

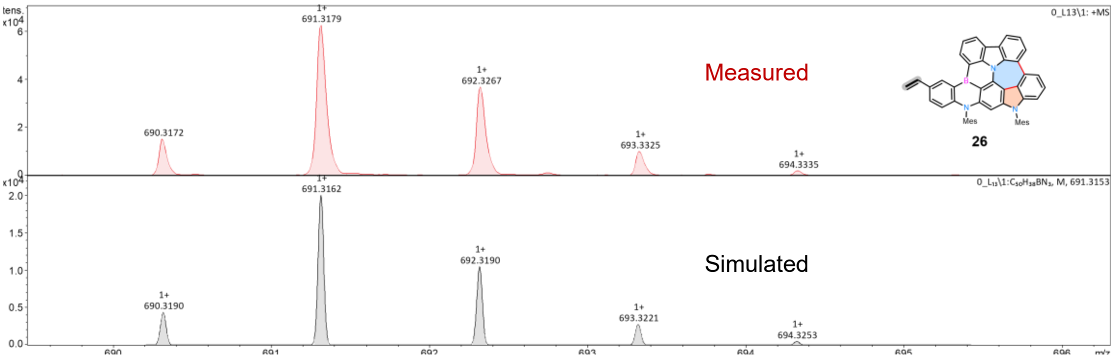

HR-MS (MALDI-TOF) spectrum of compound **27**

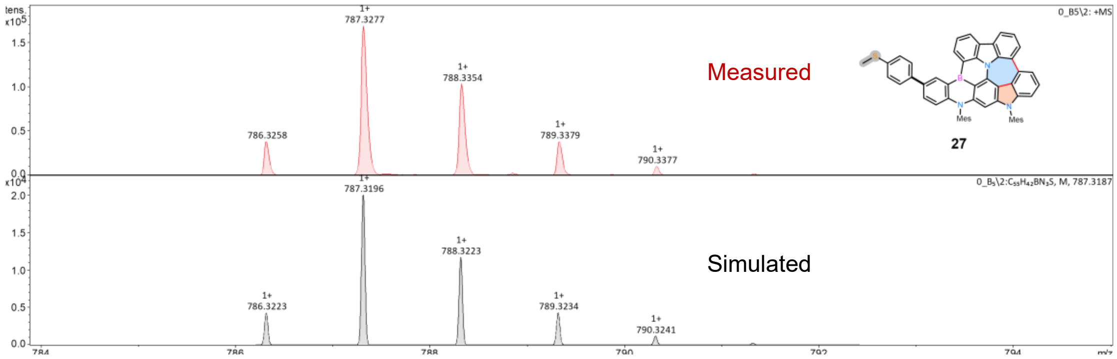

HR-MS (MALDI-TOF) spectrum of compound **28**

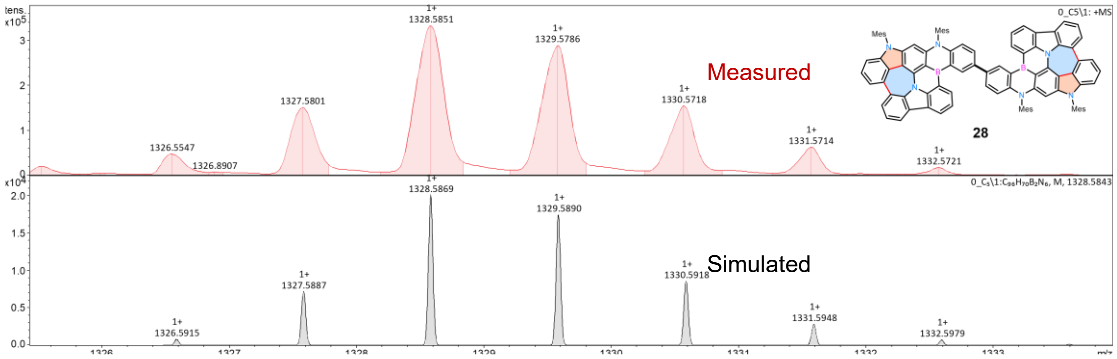

HR-MS (MALDI-TOF) spectrum of compound **29a**

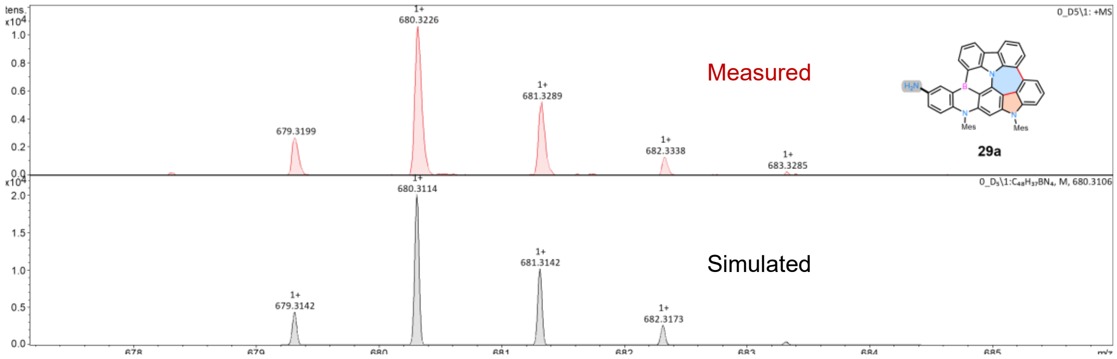

HR-MS (MALDI-TOF) spectrum of compound **29b**

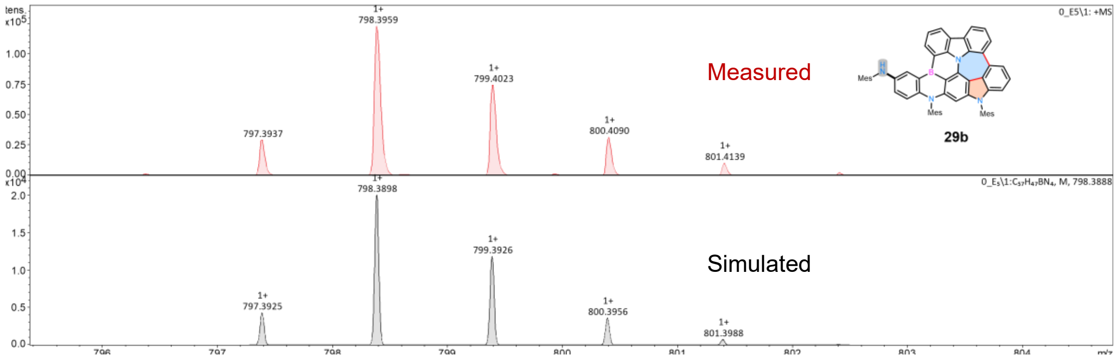

HR-MS (MALDI-TOF) spectrum of compound **30**

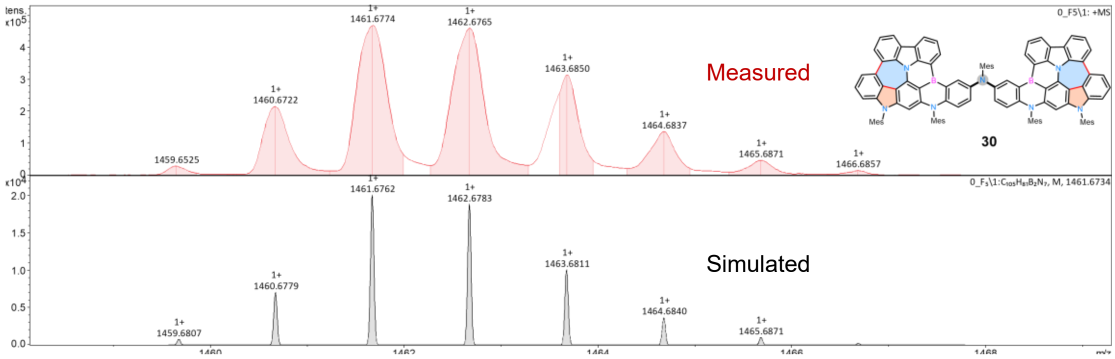

HR-MS (MALDI-TOF) spectrum of compound **31**

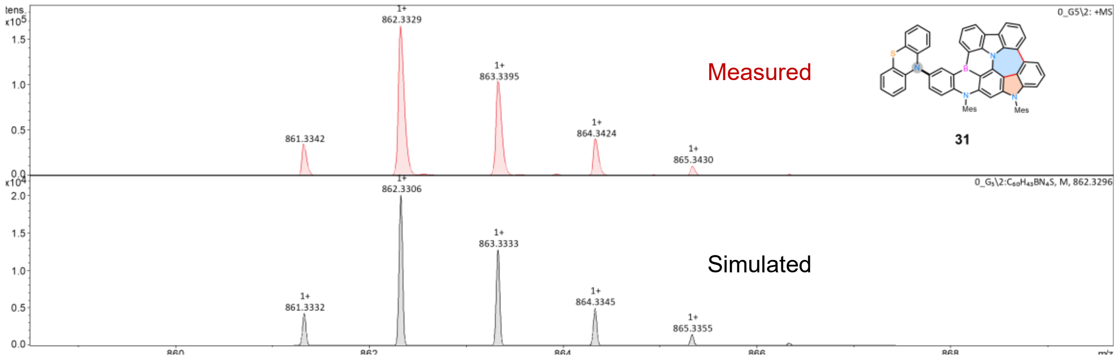

HR-MS (MALDI-TOF) spectrum of compound **32**

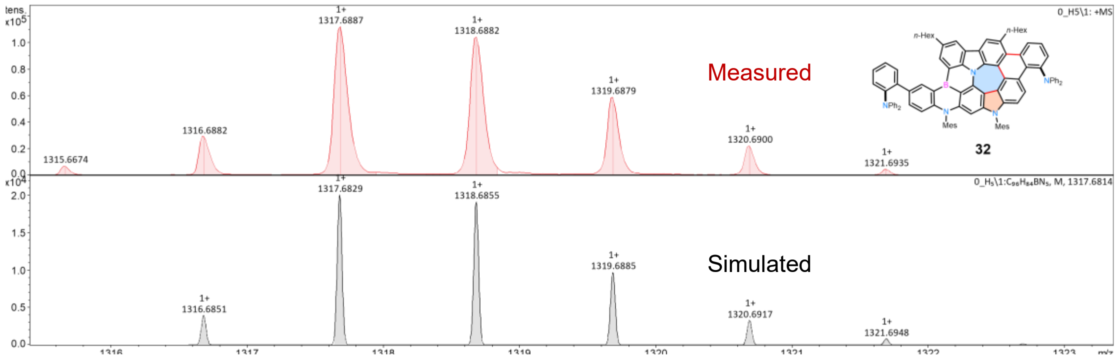

HR-MS (MALDI-TOF) spectrum of compound **33**

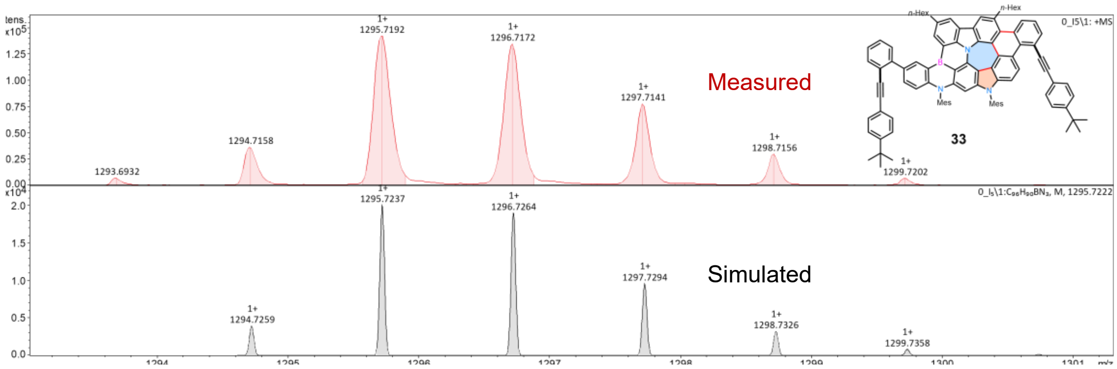

HR-MS (MALDI-TOF) spectrum of compound **34**

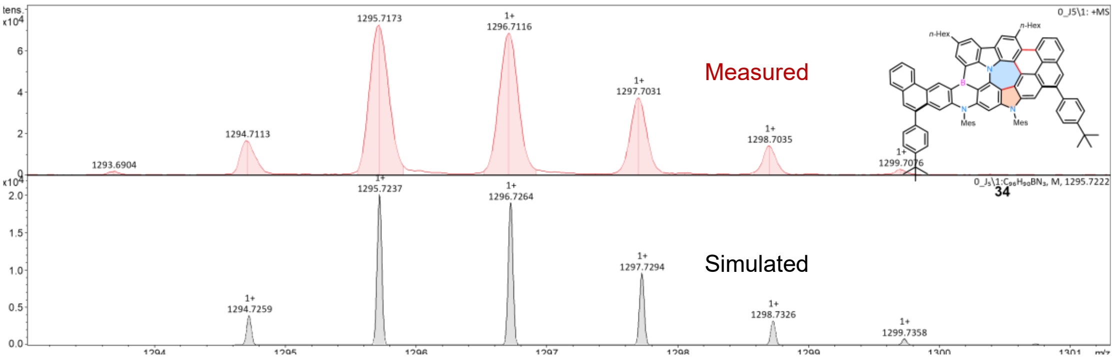

HR-MS (MALDI-TOF) spectrum of compound **35**

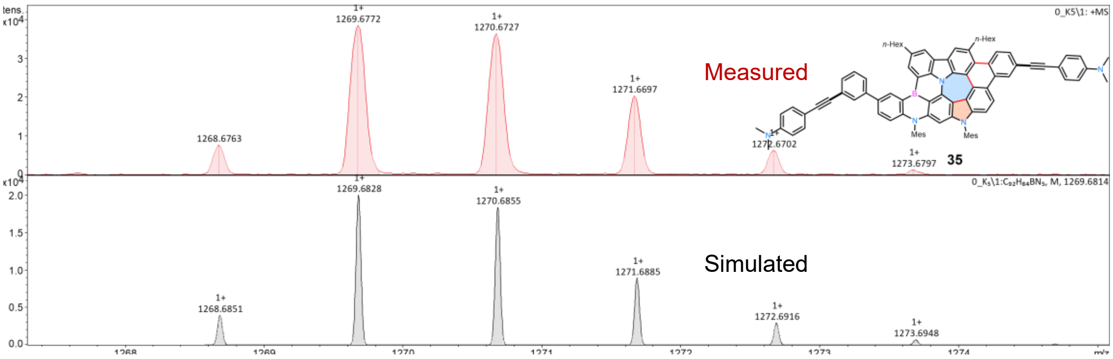

HR-MS (MALDI-TOF) spectrum of compound **36**

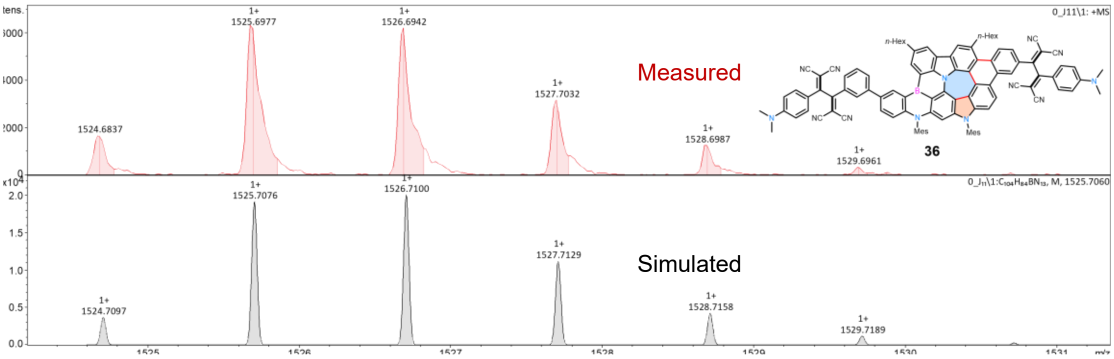

HR-MS (MALDI-TOF) spectrum of compound **S2**

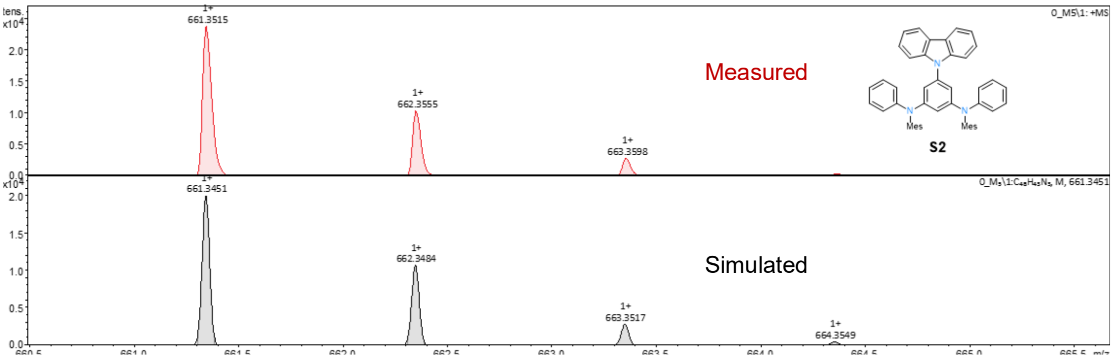

HR-MS (MALDI-TOF) spectrum of compound **S3**

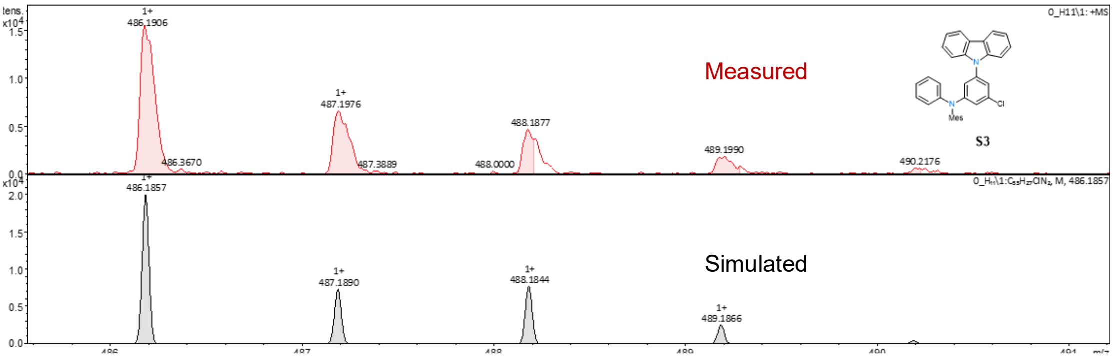

HR-MS (MALDI-TOF) spectrum of compound **S4**

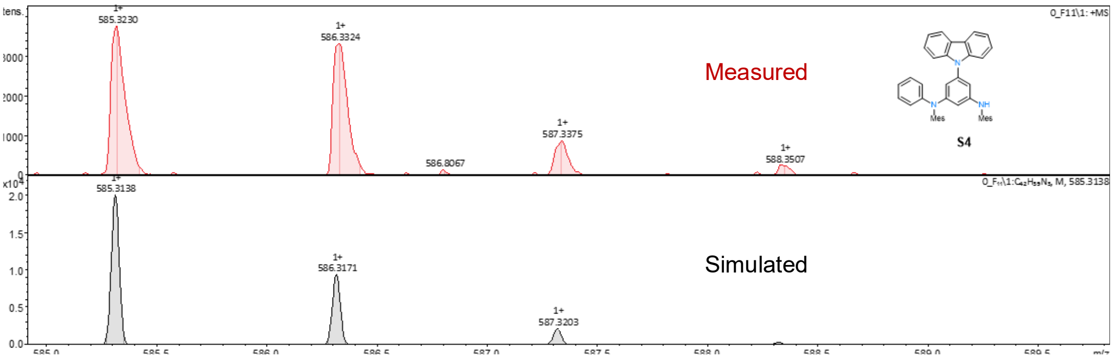

HR-MS (MALDI-TOF) spectrum of compound **S5**

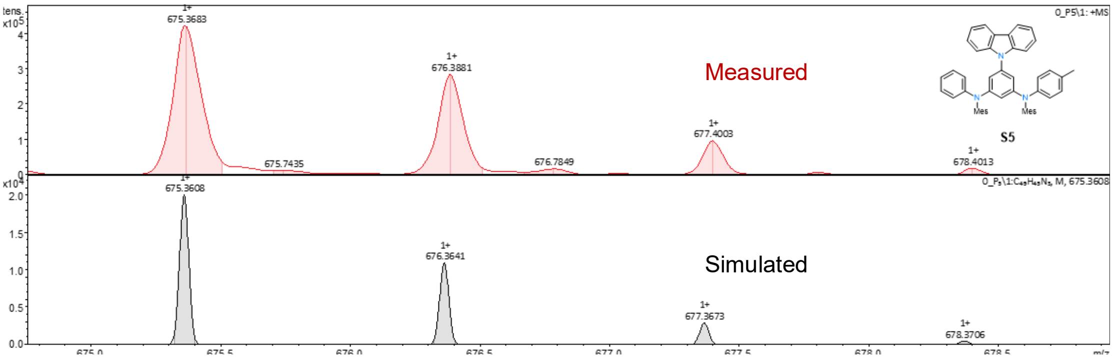

HR-MS (MALDI-TOF) spectrum of compound **S6**

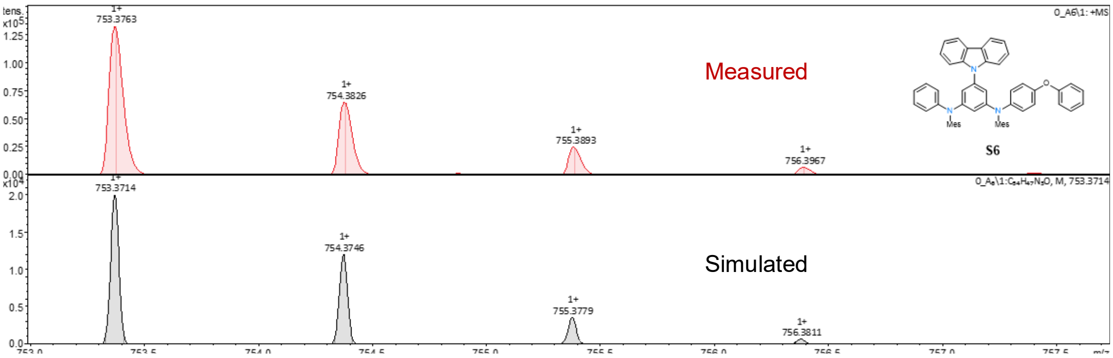

HR-MS (MALDI-TOF) spectrum of compound **S7**

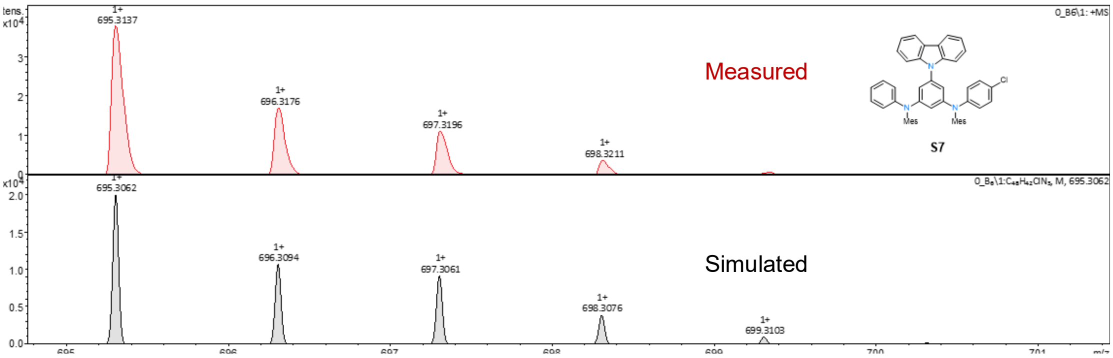

HR-MS (MALDI-TOF) spectrum of compound **S8**

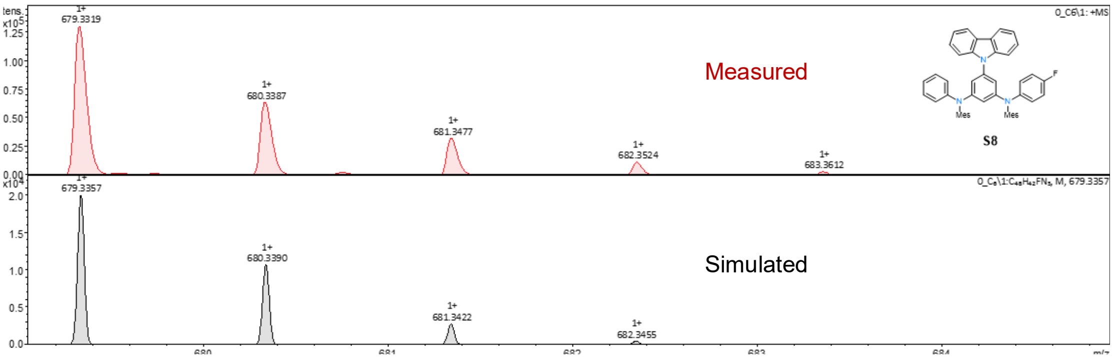

HR-MS (MALDI-TOF) spectrum of compound **S9**

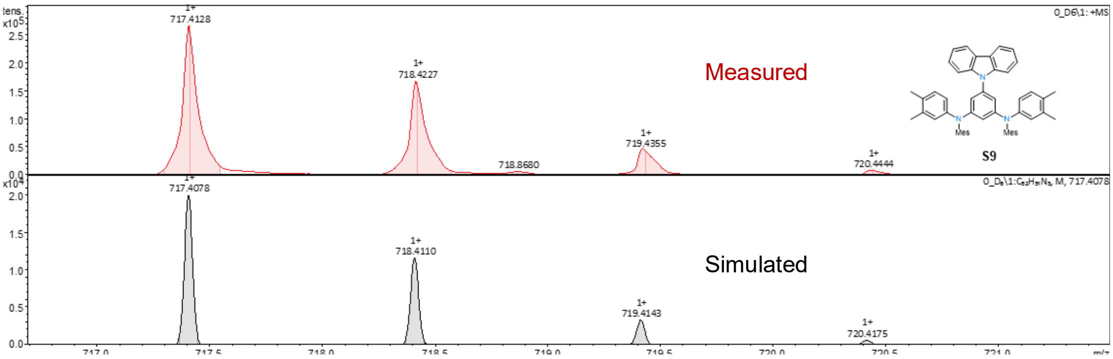

HR-MS (MALDI-TOF) spectrum of compound **S10**

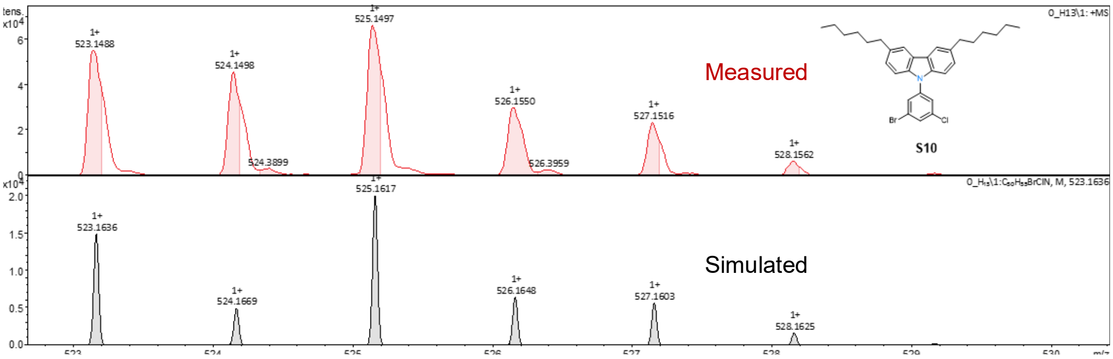

HR-MS (MALDI-TOF) spectrum of compound **S11**

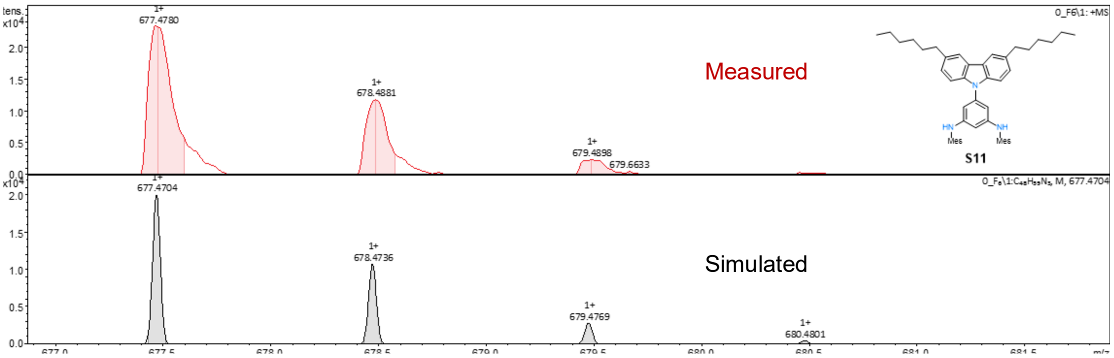

HR-MS (MALDI-TOF) spectrum of compound **S12**

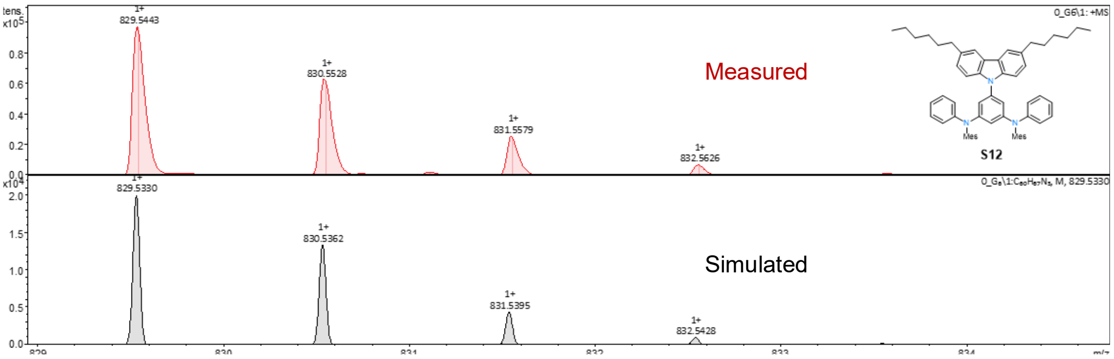

HR-MS (MALDI-TOF) spectrum of compound **S13**

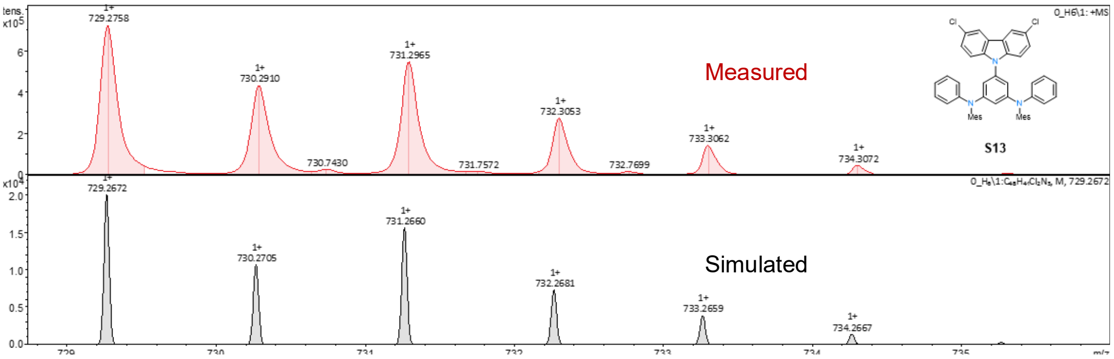

HR-MS (MALDI-TOF) spectrum of compound **S14**

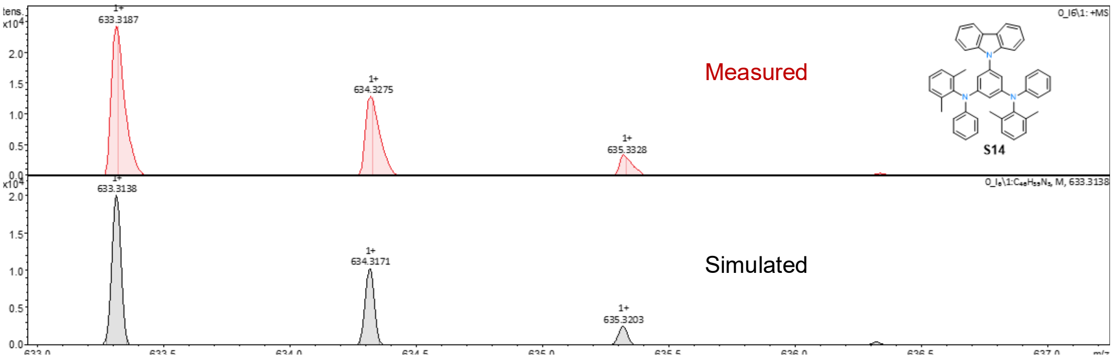

HR-MS (MALDI-TOF) spectrum of compound **S15**

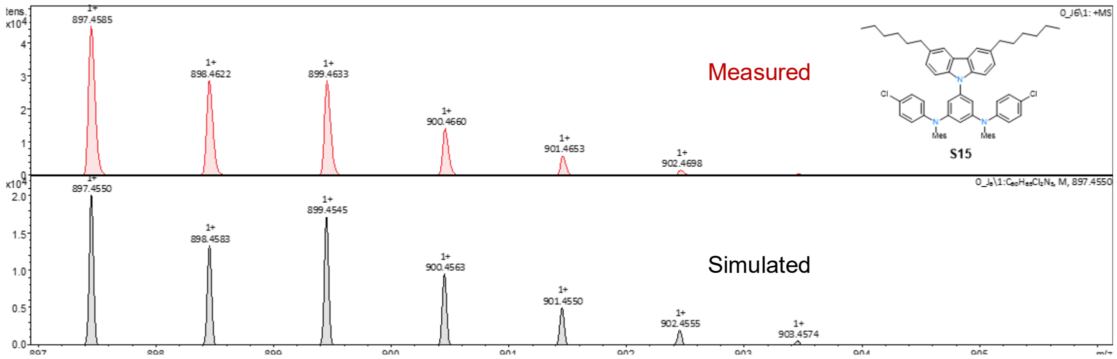

HR-MS (MALDI-TOF) spectrum of compound **S16**

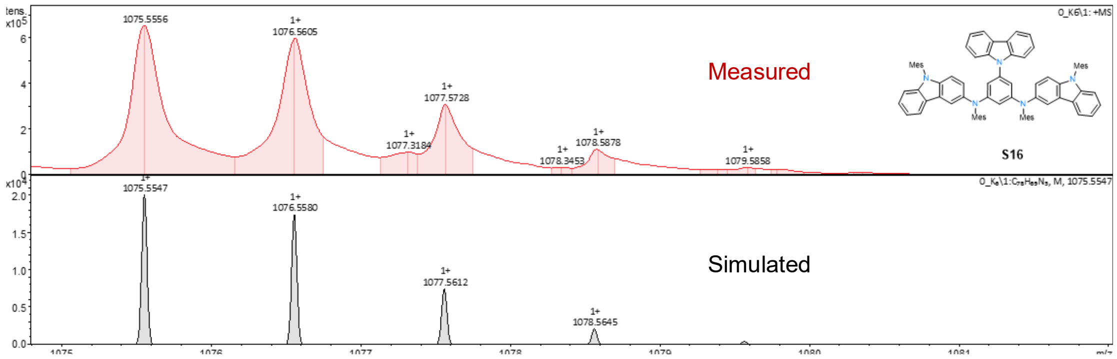

HR-MS (MALDI-TOF) spectrum of compound **S17**

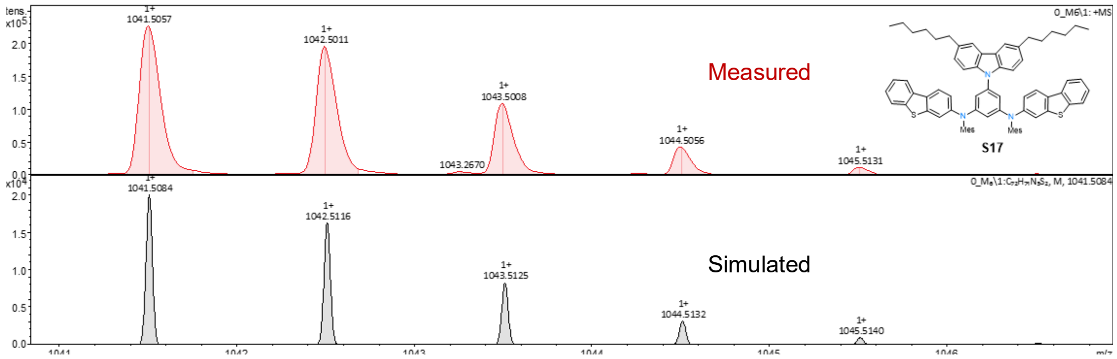

HR-MS (MALDI-TOF) spectrum of compound **S18**

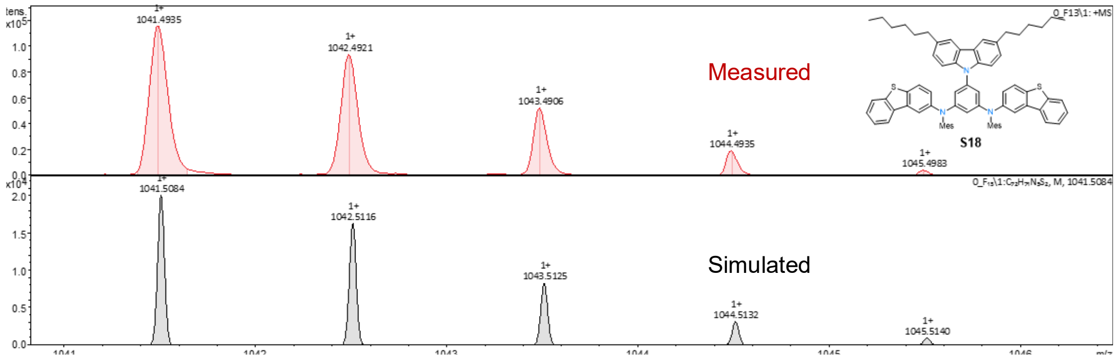

HR-MS (MALDI-TOF) spectrum of compound **S19**

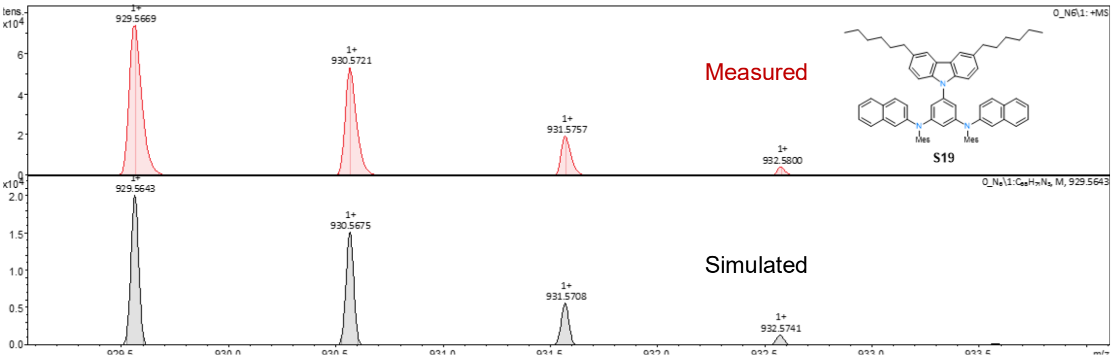

HR-MS (MALDI-TOF) spectrum of compound **S20**

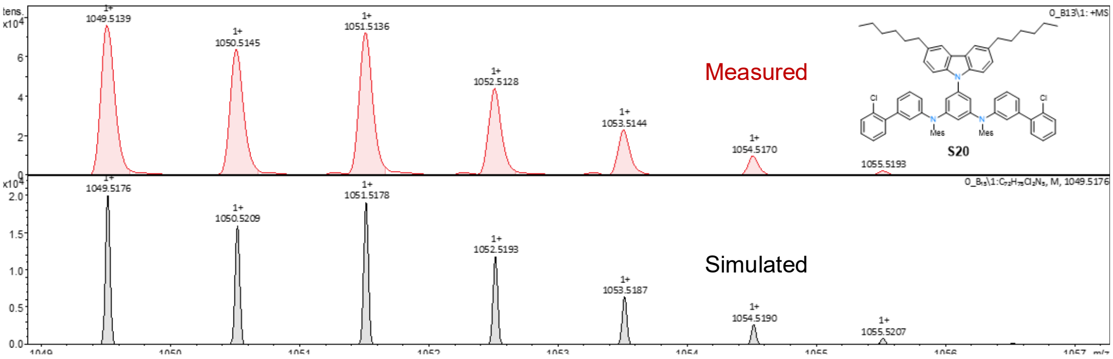

HR-MS (MALDI-TOF) spectrum of compound **S21**

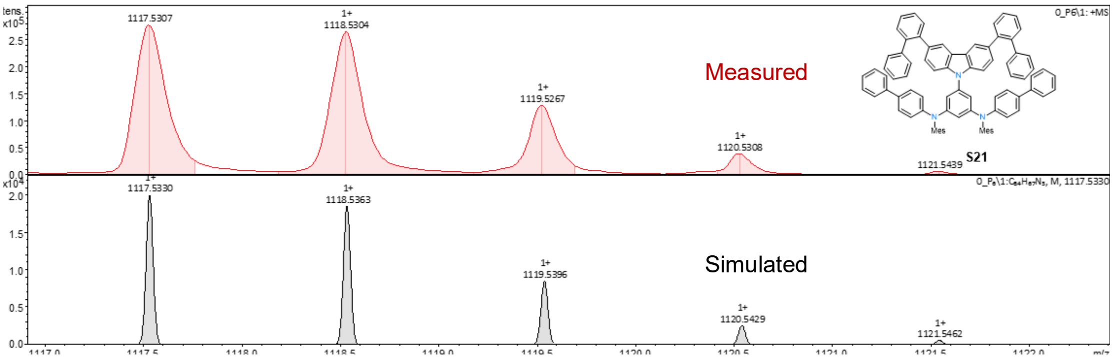

HR-MS (MALDI-TOF) spectrum of compound **S22**

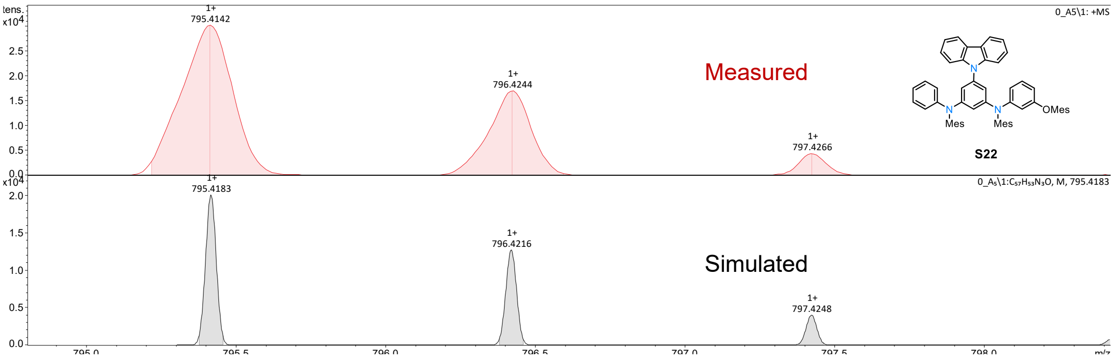

13. Reference

[S1] S. Oda, W. Kumano, T. Hama, R. Kawasumi, K. Yoshiura, T. Hatakeyama, *Angew. Chem. Int. Ed.* 2021, 60, 2882–2886.

[S2] C. Maeda, K. Nagahata, T. Shirakawa, T. Ema, *Angew. Chem. Int. Ed.* **2020**, *59*, 7813–7817.

[S3] F. Zhang, S. Das, A. J. Walkinshaw, A. Casitas, M. Taylor, M. G. Suero, M. J. Gaunt, *J. Am. Chem. Soc.* **2014**, *136*, 8851–8854.

[S4] Gaussian 16, Revision C.01, M. J. Frisch, G. W. Trucks, H. B. Schlegel, G. E. Scuseria, M. A. Robb, J. R. Cheeseman, G. Scalmani, V. Barone, G. A. Petersson, H. Nakatsuji, X. Li, M. Caricato, A. V. Marenich, J. Bloino, B. G. Janesko, R. Gomperts, B. Mennucci, H. P. Hratchian, J. V. Ortiz, A. F. Izmaylov, J. L. Sonnenberg, D. Williams-Young, F. Ding, F. Lipparini, F. Egidi, J. Goings, B. Peng, A. Petrone, T. Henderson, D. Ranasinghe, V. G. Zakrzewski, J. Gao, N. Rega, G. Zheng, W. Liang, M. Hada, M. Ehara, K. Toyota, R. Fukuda, J. Hasegawa, M. Ishida, T. Nakajima, Y. Honda, O. Kitao, H. Nakai, T. Vreven, K. Throssell, J. A. Montgomery, Jr., J. E. Peralta, F. Ogliaro, M. J. Bearpark, J. J. Heyd, E. N. Brothers, K. N. Kudin, V. N. Staroverov, T. A. Keith, R. Kobayashi, J. Normand, K. Raghavachari, A. P. Rendell, J. C. Burant, S. S. Iyengar, J. Tomasi, M. Cossi, J. M. Millam, M. Klene, C. Adamo, R. Cammi, J. W. Ochterski, R. L. Martin, K. Morokuma, O. Farkas, J. B. Foresman, and D. J. Fox, Gaussian, Inc., Wallingford CT, **2016**.

[S5] Y. Zhao, D. G. Truhlar, *Theor. Chem. Acc.* **2008**, *120*, 215–241.

[S6] A. V. Marenich, C. J. Cramer, D. G. Truhlar, *J. Chem. Theory Comput.* 2013, 9, 609–620.

[S7] F. Weigend, R. Ahlrichs, *Phys. Chem. Chem. Phys.* **2005**, *7*, 3297–3305.

[S8] F. Weigend, *Phys. Chem. Chem. Phys.* **2006**, *8*, 1057–1065.

[S9] K. Fukui, *Acc. Chem. Res.* **1981**, *14*, 363–368.

[S10] F. Sheong, J. X. Zhang, Z. Lin, *J. Comput. Chem.* **2019**, *40*, 1172–1184.

[S11] NBO 7.0. E. D. Glendening, J, K. Badenhoop, A. E. Reed, J. E. Carpenter, J. A. Bohmann, C. M. Morales, P. Karafiloglou, C. R. Landis, and F. Weinhold, Theoretical Chemistry Institute, University of Wisconsin, Madison. **2018**.

[S12] J. Tomasi, B. Mennucci, R. Cammi, *Chem. Rev.* **2005**, *105*, 2999–3093.

[S13] M. E. Casida, C. Jamorski, K. C. Casida, D. R. Salahub, *J. Chem. Phys*. **1998**, *108*, 4439–4449.

[S14] R. E. Stratmann, G. E. Scuseria, M. J. Frisch, *J. Chem. Phys.* **1998**, *109*, 8218–8224.

[S15] A. D. Becke, *J. Chem. Phys.* **1993**, *98*, 5648–5652.

[S16] C. Lee, W. Yang, R. G. Parr, *Phys. Rev. B* **1988**, *37*, 785.

[S17] T. A. Keith, R. F. W Bader, *Chem. Phys. Lett.* **1992**, *194*, 1–8.

[S18] T. A. Keith, R. F. W. Bader, *J. Chem. Phys.* **1993**, *99*, 3669–3682.

[S19] W. Humphrey, A. Dalke, K. Schulten. *J. Mol. Graph.* **1996**, *14*, 33–38.

[S20] N. M. O'Boyle, A. L. Tenderholt, K. M. Langner, *J. Comp. Chem.* **2008**, *29*, 839–845.

[S21] CYLview, 1.0b; Legault, C. Y., Université de Sherbrooke, 2009 (http://www.cylview.org).

[S22] S. Fukuzumi, Y. Yoshida, K. Okamoto, H. Imahori, Y. Araki, O. Ito, *J. Am. Chem. Soc.* **2002**, *124*, 6794–6795.

[S23] S. Fukuzumi, H. Kitaguchi, T. Suenobu, S. Ogo, *Chem. Commun.* **2002**, 1984–1985.
